# Facile Synthesis of Indole–, Pyrazole–, and Indazole–Pyrrolidine Hybrids as Novel Chiral Heterocyclic Building Blocks

**DOI:** 10.3390/molecules31152736

**Published:** 2026-08-06

**Authors:** Rokas Jankauskas, Neringa Kleizienė, Greta Račkauskienė, Aurimas Bieliauskas, Miglė Dagilienė, Sonata Krikštolė, Sergey Belyakov, Frank A. Sløk, Algirdas Šačkus

**Affiliations:** 1 Institute of Synthetic Chemistry, Kaunas University of Technology, K. Baršausko g. 59, LT-51423 Kaunas, Lithuania; neringa.kleiziene@ktu.lt (N.K.); greta.ragaite@ktu.lt (G.R.); aurimas.bieliauskas@ktu.lt (A.B.); migle.burinskaite@ktu.lt (M.D.); 2 Department of Organic Chemistry, Kaunas University of Technology, Radvilėnų pl. 19, LT-50254 Kaunas, Lithuania; sonata.krikstole@ktu.lt; 3 Latvian Institute of Organic Synthesis, Aizkraukles 21, LV-1006 Riga, Latvia; serg@osi.lv; 4 Vipergen ApS, Gammel Kongevej 23A, DK-1610 Copenhagen, Denmark; fas@vipergen.com

**Keywords:** biheterocycles, pyrrolidine, *N*-alkylation, building blocks, heterocyclic synthesis, rotamers

## Abstract

An efficient and stereoselective method for the synthesis of chiral biheterocyclic *N*-Boc-protected pyrrolidine derivatives bearing indole, pyrazole, and indazole moieties is presented. In this approach, nucleophilic substitution of heterocyclic carboxylates with enantiomerically pure *N*-Boc-3-methanesulfonyloxypyrrolidines is used to afford *N*-(pyrrolidin-3-yl) derivatives in high yields with an inverted configuration. The methodology accommodates a range of substrates, enabling structural diversity. Reactions with pyrazole- and indazolecarboxylates generate regioisomeric products. Representative peptide-coupling reactions further demonstrated the synthetic utility of the synthesized chiral biheterocyclic pyrrolidine derivatives containing protected amino and carboxyl functionalities as building blocks for peptide synthesis. Halogenated indole and pyrazole derivatives underwent further functionalization via palladium-catalyzed cross-coupling to introduce aryl, heteroaryl, and alkynyl substituents. All of the *N*-Boc-substituted biheterocycle–pyrrolidine carboxylates displayed NMR spectra with two sets of signals, notable signal broadening, or both. These effects are due to the dynamic equilibrium between two conformers in a deuterated solvent and were studied in depth. The structures and stereochemistry of the synthesized compounds were confirmed by means of chiral HPLC, single-crystal X-ray diffraction, and advanced NMR analyses. This synthetic strategy provides access to chiral heterocyclic amino acid-like building blocks for peptide synthesis and medicinal chemistry.

## 1. Introduction

**Figure 1:**
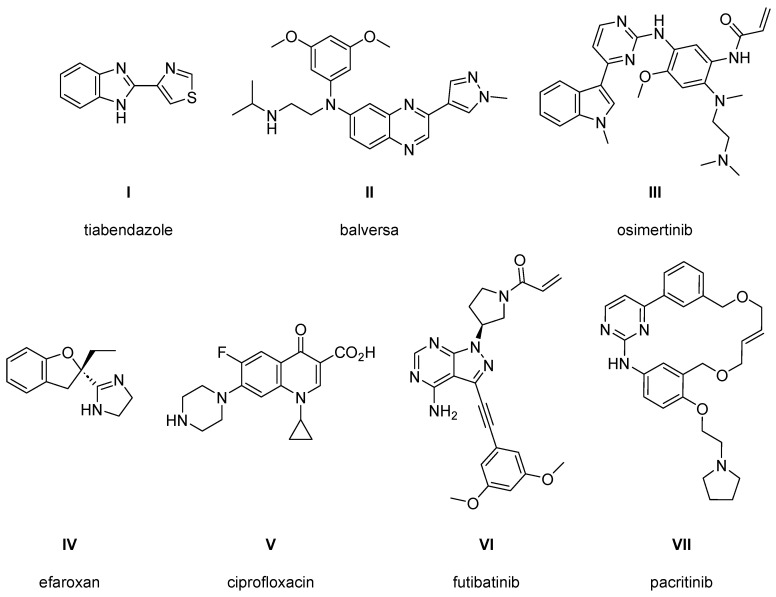
Examples of selected biheterocyclic derivatives.

Biheterocycles are an essential class of heterocyclic compounds in medicinal chemistry. Their design involves combining two pharmacophoric scaffolds into a single molecule. This combination enhances pharmacological potency, often exceeding the effects of each component alone [1,2]. Biheterocycles exhibit a variety of complex frameworks, including linear [3], angular [4], spiro [5], fused [6], and bridged [7,8] heterocyclic rings, and frequently serve as scaffolds for the development of new biologically active molecules [9]. Linear biheterocycles can be symmetrical or unsymmetrical and connect two heterocyclic rings with a single bond. These structures provide a valuable platform for developing drugs targeting various diseases. They are commonly used as antitumor [10,11], antidiabetic [12], antifungal or antihelminthic [13], anti-inflammatory [14,15], antimicrobial [16], and antidepressant [17] agents as well as for other therapeutic purposes [18]. Many commercially available drugs contain a biheterocyclic moiety. For example, the linear biheterocycle tiabendazole (**I**) serves as an effective antifungal and antiparasitic agent [19] (Figure 1). Balversa (**II**) functions as a pan-fibroblast growth factor receptor inhibitor for the treatment of locally advanced or metastatic urothelial carcinoma [20]. The 1*H*-indole derivative osimertinib (**III**) is used to treat non-small-cell lung cancers with specific mutations [21]. Efaroxan (**IV**), with its imidazoline-benzofuran ring system, is a well-known α2-adrenergic receptor antagonist with antihyperglycemic effects [22]. Additionally, antibiotics such as norfloxacin and ciprofloxacin (**V**), which are fluoroquinolone-pyrazine derivatives, are broad-spectrum antimicrobial agents commonly used to treat infections [23]. There are also biheterocyclic pyrrolidine derivatives that have been approved as active drugs. For example, futibatinib (**VI**) is approved as an anticancer medication for treating a specific type of bile duct cancer [24], while pacritinib (**VII**) is approved for certain forms of myelofibrosis, a type of bone marrow cancer [25].

Linear biheterocycles often contain functional groups such as halogen, vinyl, alkynyl, hydroxyl, carbonyl, and primary or secondary amino groups, making them useful as reactive precursors for the synthesis of more complex biologically active molecules [18,26,27,28]. For example, triheterocyclic anticancer agents targeting colorectal lines were smoothly prepared by the Suzuki–Miyaura cross-coupling of brominated tetrahydroquinoline–indole hybrids with an aminopyrimidinyl boronic acid [29]. Similarly, the Knoevenagel condensation of purine–pyrazolecarbaldehydes with rhodanine-3-acetic acid derivatives under ammonium acetate catalysis yielded complex biheterocyclic scaffolds possessing potent antioxidant and antitumor properties [30]. Heterocycles conjugated to amino acids and peptides represent an impactful class of therapeutic agents [31,32]. Gowda’s group appended piperidinyl-benzisoxazole and piperazinyl-benzisothiazole platforms to distinct peptide fragments via solution-phase Boc chemistry to evaluate their inhibitory potential against pathogenic bacteria [33,34]. Concurrently, Katritzky’s team utilized benzotriazole chemistry to efficiently tether fluoroquinolones—such as norfloxacin and ciprofloxacin—to amino acid linkers, providing hybrid bis-conjugates with antibacterial activities matching those of the parent drugs [35].

Heterocyclic unnatural amino acids are also widely deployed as rigid structural building blocks in peptide engineering [32,36,37,38]. For instance, isoxazole–cycloamine frameworks have been synthesized and seamlessly incorporated into rigidified peptidomimetics [39,40]. Recently, our group developed robust methods combining simple NH-heterocyclic carboxylic esters with functionalized azetidines to generate achiral biheterocyclic building blocks aimed at similar peptidomimetic applications [41]. In the present work, this concept is further expanded to stereochemically defined biheterocyclic *N*-Boc amino esters, whose synthetic utility as peptide building blocks is demonstrated through representative peptide-coupling reactions.

This paper describes the design and synthesis of biheterocyclic hybrids derived from pyrazole-, indazole-, and indolecarboxylates, featuring functionalized pyrrolidine as novel chiral heterocyclic Boc-amino esters. Pyrrolidine is a key synthetic fragment in drug development that is critical in medicinal chemistry and the pharmaceutical industry [42,43]. Therefore, the newly developed biheterocycle–pyrrolidine hybrids may serve as useful isosteres and conformationally rigid amino acid-like units, with potential applications as biologically active compounds and peptidomimetics, including as chiral building blocks for synthesizing DNA-encoded compound libraries [44,45,46].

## 2. Results and Discussion

### 2.1. Chemistry

**Scheme 1:**
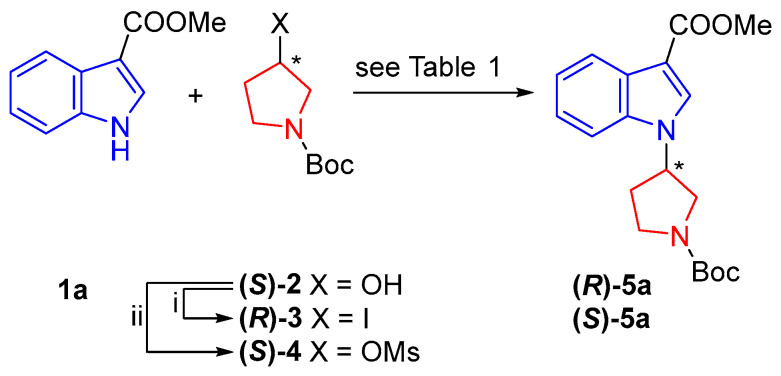
Preparation of 3-functionalized *N*-Boc-pyrrolidines and their coupling with methyl 1*H*-indole-3-carboxylate **1a**. Reaction conditions are as follows. i: I_2_, imidazole, PPh_3_, toluene, 100 °C, 4 h; ii: Methanesulfonyl chloride, TEA, DCM, 0 °C to rt, 3 h. Asterisk (*) denotes a stereocenter.

The development of a coupling reaction to access biheterocycle–pyrrolidine hybrids is outlined in Scheme 1. *N*-Boc-(*S*)-3-hydroxypyrrolidine (**(*S*)-2**) was selected as a stereochemically defined precursor for coupling with indole **1a** to afford the corresponding *N*-alkylated target compounds (Scheme 1). The secondary hydroxy group of **(*S*)-2** enables either direct coupling under Mitsunobu conditions or conversion into activated electrophilic derivatives suitable for nucleophilic substitution [47,48].

Based on our previous studies [41], iodide **(*R*)-3** and mesylate **(*S*)-4** were prepared from alcohol **(*S*)-2** to investigate leaving-group effects in the *N*-alkylation of indole **1a**. Iodination under Appel conditions [49] afforded iodide **(*R*)-3** with inversion at the stereogenic center, whereas mesylation with methanesulfonyl chloride [50] gave mesylate **(*S*)-4**. Compounds **(*S*)-2**, **(*R*)-3**, and **(*S*)-4** were subsequently examined in reactions with indole **1a** (Scheme 1).

The initial coupling of *N*-Boc-(*S*)-3-hydroxypyrrolidine (**(*S*)-2**) with indole **1a** under Mitsunobu conditions [51] (Table 1, entry 1) afforded **(*R*)-5a** in an 11% yield. This stereochemical inversion is consistent with the known stereochemical course of the Mitsunobu reaction [52,53]. Reacting **1a** with the iodide derivative **(*R*)-3** also resulted in low yields of **(*S*)-5a** (19% and 8%—Table 1, entries 2 and 3). In both cases, the low yields resulted from incomplete conversion of the starting materials, while no significant side reactions were observed. In contrast, coupling of **(*S*)-4** with **1a** under Cs_2_CO_3_/DMF conditions at 80 °C for 4 h afforded **(*R*)-5a** in 92% yield (Table 1, entry 4). Given the excellent performance of the mesylate **(*S*)-4**, no additional sulfonate leaving groups were investigated, and this method was selected for subsequent studies. Using the same mesylation procedure, the enantiomeric mesylate **(*R*)-4** was also prepared from the corresponding alcohol **(*R*)-2** and subsequently employed in the synthesis of the corresponding enantiomeric indole derivatives.

**Scheme 2:**
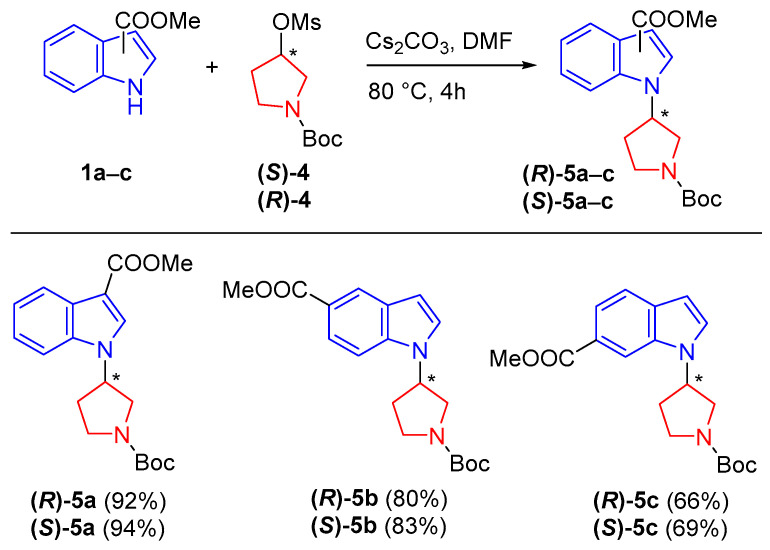
Alkylation of methyl indolecarboxylates **1a**–**c** with (*S*)-*N*-Boc-3-methanesulfonyloxypyrrolidine **(*S*)-4** or (*R*)-*N*-Boc-3-methanesulfonyloxypyrrolidine **(*R*)-4**. Asterisk (*) denotes a stereocenter.

Under the optimized conditions in Table 1 (entry 4), *N*-unsubstituted methyl indole-3-, -5-, and -6-carboxylates **1a**–**c** underwent *N*-alkylation with the chiral pyrrolidine mesylates **(*S*)-4** and **(*R*)-4** (Scheme 2). In all cases, nucleophilic substitution proceeded with inversion of configuration, yielding **(*R*)-5a**–**c** and **(*S*)-5a**–**c** in good yields (66–94%). These results demonstrate the general applicability of the developed S_N_2 *N*-alkylation protocol across differently substituted indole carboxylates.

**Figure 2:**
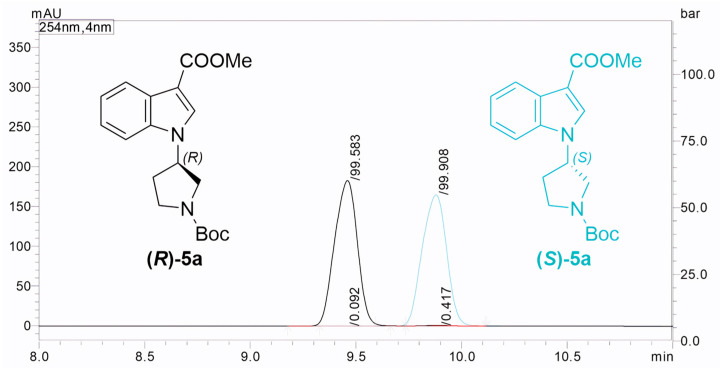
Superimposed chromatogram view showing chromatograms for nucleophilic substitution products **(*R*)-5a** (black line, ee 99.2%) and **(*S*)-5a** (cyan line, ee 99.8%). Conditions: CHIRAL ART Cellulose-SB (100 × 4.6 mm I.D., S-3 μm); mobile phase: H_2_O + 0.1% HCOOH/MeCN 70:30 to 50:50 in 16 min; T = 36 °C; flow rate 1.0 mL/min. Samples were prepared in methanol. λ_det_ = 245 nm.

The synthesized chiral *N*-(pyrrolidin-3-yl)-1*H*-indolecarboxylates **(*R*)-** and **(*S*)-5a**–**c** exhibited optical activity, with the corresponding (*R*)- and (*S*)-enantiomers rotating plane-polarized light in opposite directions. All compounds were analyzed using chiral HPLC, confirming their high enantiomeric purity (see the Supporting Information Figures S204–S227). As shown in Figure 2, **(*R*)-5a** and **(*S*)-5a** were obtained with excellent enantiomeric excesses of 99.2% and 99.8%, respectively. Nucleophilic substitution at secondary alkyl electrophiles can be challenging because of steric constraints and competing elimination pathways, and various strategies, including transition-metal-catalyzed approaches, have been developed to address limitations in reactivity and stereochemical control [54,55,56]. In the present study, *N*-alkylation of the heterocyclic substrates with enantiomerically pure secondary pyrrolidine mesylates proceeded efficiently under the selected conditions (Cs_2_CO_3_, DMF, 80 °C), affording the corresponding products in high yields with complete inversion of configuration and essentially no loss of enantiomeric purity. These results are consistent with a stereospecific S_N_2 substitution pathway.

**Figure 3:**
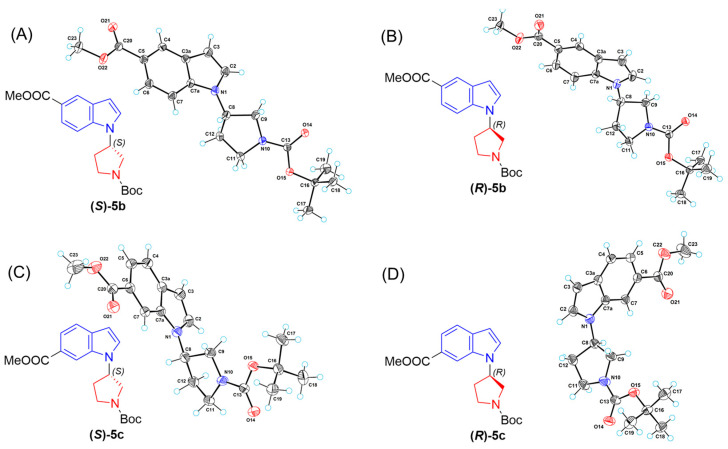
ORTEP view of crystals **(*S*)-5b**/**(*R*)-5b** and **(*S*)-5c**/**(*R*)-5c** and corresponding structural formulas: (**A**) methyl 1-[(3*S*)-1-(*tert*-butoxycarbonyl)pyrrolidin-3-yl]-1*H*-indole-5-carboxylate; (**B**) methyl 1-[(3*R*)-1-(*tert*-butoxycarbonyl)pyrrolidin-3-yl]-1*H*-indole-5-carboxylate; (**C**) methyl 1-[(3*S*)-1-(*tert*-butoxycarbonyl)pyrrolidin-3-yl]-1*H*-indole-6-carboxylate and (**D**) methyl 1-[(3*R*)-1-(*tert*-butoxycarbonyl)pyrrolidin-3-yl]-1*H*-indole-6-carboxylate.

Single-crystal X-ray diffraction analysis was performed to further confirm the molecular structures and absolute configurations of the synthesized compounds. Suitable single crystals of the representative enantiomeric pairs **(*S*)-5b**/**(*R*)-5b** and **(*S*)-5c**/**(*R*)-5c** were obtained via recrystallization from methanol (Figure 3).

**Figure 4:**
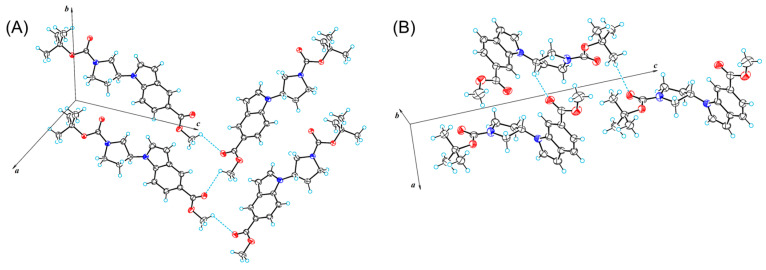
Crystal structure of (**A**) methyl 1-[(3*S*)-1-(*tert*-butoxycarbonyl)pyrrolidin-3-yl]-1*H*-indole-5-carboxylate **(*S*)-5b** and (**B**) methyl 1-[(3*S*)-1-(*tert*-butoxycarbonyl)pyrrolidin-3-yl]-1*H*-indole-6-carboxylate **(*S*)-5c**.

The pyrrolidine ring in **(*S*)-5b** adopts a typical envelope conformation, with the C12 atom 0.539(2) Å out of the plane of the other atoms. The crystal structure features a CH···O hydrogen bond between the C23-H23B group and the carbonyl oxygen O21. The methyl group’s carbon, attached to the ester oxygen, is more electronegative, leading to a moderate CH···O hydrogen bond, as observed in similar cases. The bond length is 3.114(2) Å (H23B···O21 = 2.66 Å, C23-H23B···O21 = 109°). This hydrogen bond forms molecular chains along the monoclinic axis (Figure 4A). Because **(*S*)-5b** and **(*R*)-5b** are mirror images, these structural features are also present in the **(*R*)-5b** structure.

Like the **(*S*)-5b** structure, the **(*S*)-5c** structure adopts an envelope conformation in the pyrrolidine ring. In this case, the C8 atom, rather than C12, protrudes by 0.541(3) Å from the plane defined by the other four atoms. The crystal structure displays relatively weak CH···O hydrogen bonds (Figure 4B). One such bond, C19-H19B···O14, measures 3.187(3) Å, with H19B···O14 = 2.87 Å and an angle of 100°. The second bond, C8-H8···O21, measures 3.300(3) Å; this interaction may contribute to the displacement of the C8 atom from the plane of the other pyrrolidine ring atoms. Its parameters are H8···O21 = 2.47 Å and an angle of 142°. These hydrogen bonds facilitate the formation of molecular networks or layers within the crystal structure, oriented perpendicular to the monoclinic axis. All these structural features are also present in the mirror-symmetric crystal structure of **(*R*)-5c**.

**Scheme 3:**
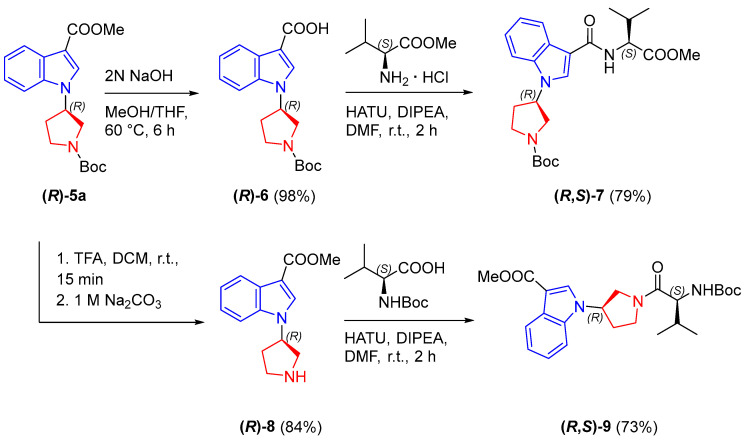
Synthesis of dipeptides **(*R*,*S*)-7** and **(*R*,*S*)-9**.

To demonstrate the synthetic utility of the synthesized chiral biheterocyclic building blocks, representative peptide-coupling reactions were performed (Scheme 3). Hydrolysis of methyl ester **(*R*)-5a** afforded the corresponding carboxylic acid **(*R*)-6**, which was subsequently coupled with *L*-valine methyl ester hydrochloride under standard HATU/DIPEA-mediated conditions [57] to furnish dipeptide **(*R*,*S*)-7** in 79% yield. In a complementary approach, Boc deprotection of **(*R*)-5a** provided the corresponding amine **(*R*)-8**, which underwent coupling with Boc-*L*-valine under analogous conditions to afford dipeptide **(*R*,*S*)-9** in 73% yield. In both cases, the products were isolated in good yield, and no additional diastereomeric signals were evident in the ^1^H NMR spectra. These complementary transformations demonstrate that both the amino and carboxyl functionalities of the synthesized pyrrolidine derivatives can be employed in peptide-bond formation. The successful preparation of representative dipeptides further demonstrates the potential of these chiral biheterocyclic derivatives as amino acid-like building blocks for peptide synthesis.

**Scheme 4:**
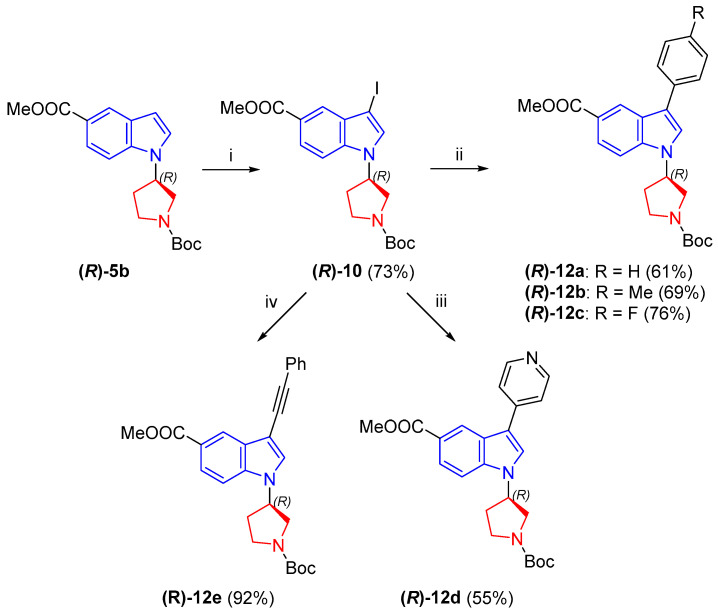
Synthesis of C-3-substituted indole derivatives via iodination and palladium-catalyzed cross-coupling reactions. Reaction conditions: (i) I_2_, KOH, DMF, r.t., 2 h; (ii) Suzuki–Miyaura coupling—ArB(OH)_2_ (**11a**–**c**), 5 mol% Pd(dppf)Cl_2_·DCM, K_3_PO_4_, dioxane/H_2_O, 80 °C, MW, 100 W, 15 min; (iii) Suzuki–Miyaura coupling—4-Py-B(OH)_2_ (**11d**), 5 mol% Pd(dppf)Cl_2_·DCM, K_3_PO_4_, dioxane/H_2_O, 80 °C, 1 h; (iv) Sonogashira coupling—Ph–C≡CH, 5 mol% Pd(PPh_3_)_2_Cl_2_, CuI, Et_3_N, DMF, 65 °C, 1 h.

Functionalization of heterocyclic compounds is a highly efficient and widely used strategy for expanding heterocyclic building-block libraries [58]. Palladium-catalyzed cross-coupling reactions are among the most powerful methods for modifying heteroaromatic systems, enabling the efficient and selective introduction of a wide range of substituents into the molecular framework [59,60,61]. The indole derivative **(*R*)-5b** was selected as a representative scaffold for further functionalization (Scheme 4). As reported in the literature, the indole core undergoes selective halogenation at the C-3 position [62]. The indole derivative **(*R*)-5b** was iodinated using iodine and potassium hydroxide in DMF according to a literature procedure [62,63] to afford compound **(*R*)-10**. The resulting 3-iodoindole derivative **(*R*)-10** provided a suitable substrate for palladium-catalyzed cross-coupling reactions. Suzuki–Miyaura coupling of **(*R*)-10** with phenylboronic acid **11a** was investigated using selected palladium catalysts (Table 2, entries 1–3). Progress was monitored via thin-layer chromatography, and the reaction mixture was quenched upon complete consumption of the starting material.

Under typical Suzuki–Miyaura conditions, using Pd(PPh_3_)_4_ as the catalyst and K_3_PO_4_ as the base in dioxane at 100 °C [61,64,65], the reaction between 3-iodoindole **(*R*)-10** and phenylboronic acid **11a** was evaluated. Under these conditions, compound **(*R*)-12a** was obtained in a 32% yield after 2 h (Table 2, entry 1). Although the desired product had formed, the moderate yield indicated that further optimization of the catalytic system and reaction parameters was required.

Given reports of the high catalytic activity of *N*-heterocyclic carbene (NHC)-based palladium complexes in cross-coupling reactions, particularly with heteroaryl halides [66,67,68], Pd-PEPPSI-IPr was subsequently evaluated as an alternative catalytic system. However, in our case, this catalyst did not result in the expected improvement, affording the desired product **(*R*)-12a** in only a 6% yield after 4 h at 100 °C (Table 2, entry 2). This result indicates that the Pd-PEPPSI-IPr system is unsuitable for coupling 3-iodoindole **(*R*)-10** under the conditions investigated.

Because the NHC-based catalyst did not provide satisfactory results, attention was then directed to phosphine-based ligand systems. Given reports that 1,1′-bis(diphenylphosphino)ferrocene (dppf) is an efficient ligand for Suzuki–Miyaura cross-coupling reactions involving heteroaryl halides [69,70,71], Pd(dppf)Cl_2_·DCM was examined as an alternative catalytic system. A significant improvement was observed when the reaction was carried out in a mixed dioxane/H_2_O solvent system in the presence of K_3_PO_4_. Under these conditions, at a lower temperature of 80 °C, the desired product **(*R*)-12a** was obtained in a 57% yield within 1 h (Table 2, entry 3), suggesting beneficial effects of the dppf ligand and the aqueous co-solvent on the efficiency of the coupling reaction.

Further enhancement was achieved by applying microwave irradiation under otherwise identical conditions. Heating at 100 W reduced the reaction time to 15 min and slightly increased the yield to 61% (Table 2, entry 4). Consequently, the combination of Pd(dppf)Cl_2_·DCM, K_3_PO_4_, and a dioxane/H_2_O solvent system under microwave irradiation was identified as the optimal set of conditions for the Suzuki–Miyaura coupling of 3-iodoindole **(*R*)-10** with phenylboronic acid **11a**.

Based on the screening results, the most efficient reaction conditions were selected for further studies. Under these conditions, 3-iodoindole **(*R*)-10** was reacted with substituted arylboronic acids to evaluate the reaction scope. In particular, *p*-tolylboronic acid (**11b**), bearing an electron-donating substituent, afforded compound **(*R*)-12b** in a 69% yield, whereas 4-fluorophenylboronic acid (**11c**), bearing an electron-withdrawing substituent, produced compound **(*R*)-12c** in a 76% yield (Scheme 4). These results demonstrate that arylboronic acids with different electronic properties are well tolerated under the reaction conditions employed.

Owing to the reactivity and stability challenges associated with the deborylation of 4-pyridinylboronic acid (**11d**) [72,73], the procedure was slightly modified to enable the synthesis of the heteroaryl-substituted derivative **(*R*)-12d**. The reaction was carried out under the optimized Suzuki–Miyaura catalytic conditions at 80 °C using conventional thermal heating. The reaction mixture containing **(*R*)-10** was heated to the reaction temperature, and 4-pyridinylboronic acid (**11d**) was added after 10 min; the reaction was then continued for 1 h, affording compound **(*R*)-12d** in a 55% yield (Scheme 4). This result suggests that the catalytic system is also applicable to heteroaryl substrates, although minor adjustments of the reaction protocol may be necessary.

In addition, the versatility of the iodinated intermediate **(*R*)-10** was further demonstrated by its use in a Sonogashira cross-coupling reaction. The reaction of 3-iodoindole **(*R*)-10** with phenylacetylene was carried out under standard Sonogashira conditions, using Pd(PPh_3_)_2_Cl_2_ and CuI as catalysts in the presence of Et_3_N in DMF at 65 °C [74]. Under these conditions, the desired 3-(2-phenylethynyl)-1*H*-indole derivative **(*R*)-12e** was obtained in a 92% yield (Scheme 4). This outcome highlights the high efficiency of the Sonogashira protocol for introducing alkynyl groups at the C-3 position of the indole scaffold.

Overall, these results demonstrate that the developed palladium-catalyzed cross-coupling protocols enable efficient introduction of aryl, heteroaryl, and alkynyl substituents at the C-3 position of the indole core. This approach is a robust, flexible strategy for systematically expanding the library of C-3-substituted indole derivatives and underscores the synthetic utility of 3-iodoindole **(*R*)-10** as a versatile heterocyclic building block.

**Scheme 5:**
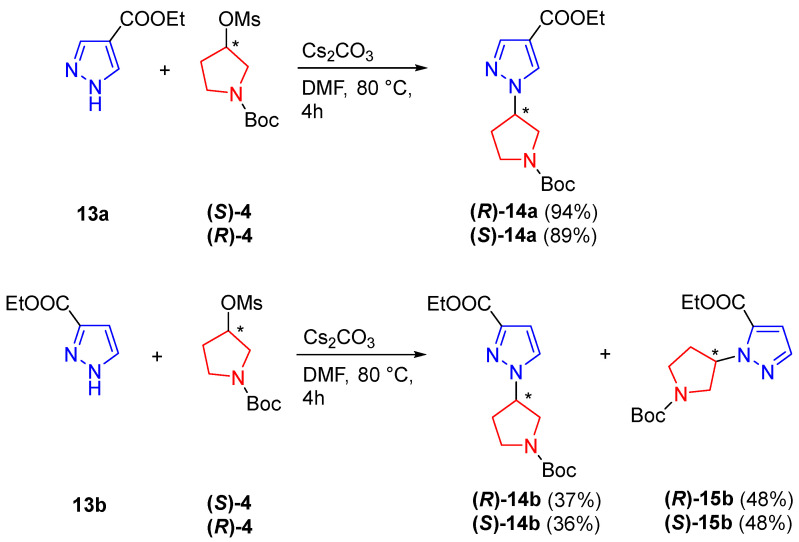
Alkylation of ethyl pyrazolecarboxylates **13a**,**b** with (*S*)-*N*-Boc-3-methanesulfonyloxypyrrolidine **(*S*)-4** or (*R*)-*N*-Boc-3-methanesulfonyloxypyrrolidine **(*R*)-4**. Asterisk (*) denotes a stereocenter.

The next objective of this study was to synthesize *N*-(pyrrolidin-3-yl)-1*H*-pyrazolecarboxylates to expand the compound library by introducing an additional heteroaromatic scaffold. Accordingly, *N*-alkylation of ethyl 1*H*-pyrazole-4-carboxylate **13a** with the chiral mesylates **(*S*)-4** or **(*R*)-4**, using Cs_2_CO_3_ in DMF at 80 °C, afforded the corresponding *N*-alkylated products **(*R*)-14a** and **(*S*)-14a** in high yields of 94% and 89%, respectively (Scheme 5).

The optimized alkylation conditions were subsequently applied to the unsymmetrical ethyl 1*H*-pyrazole-3-carboxylate **13b**. DFT calculations reported in the literature [75] show that the N1 and N2 atoms of 3-CO_2_Et pyrazole have nearly identical nucleophilicity, predicting low regioselectivity, whereas the electron-withdrawing 3-NO_2_ group makes N1 more nucleophilic and drives selective alkylation. In our case, this lower regioselectivity is desirable, as both isolated regioisomers expand the molecular diversity of the building block library. In this instance, reaction with mesylate **(*S*)-4** produced a mixture of two regioisomers, **(*R*)-14b** and **(*R*)-15b**, in an approximately 3:4 ratio. Separation by flash column chromatography afforded the corresponding products in 37% and 48% yields, respectively. Similarly, alkylation of pyrazole **13b** with mesylate **(*R*)-4** gave the analogous regioisomeric pair, **(*S*)-14b** and **(*S*)-15b**, in comparable yields (Scheme 5). Thus, alkylation of the pyrazole ring occurs at either nitrogen atom, yielding regioisomeric products.

**Scheme 6:**
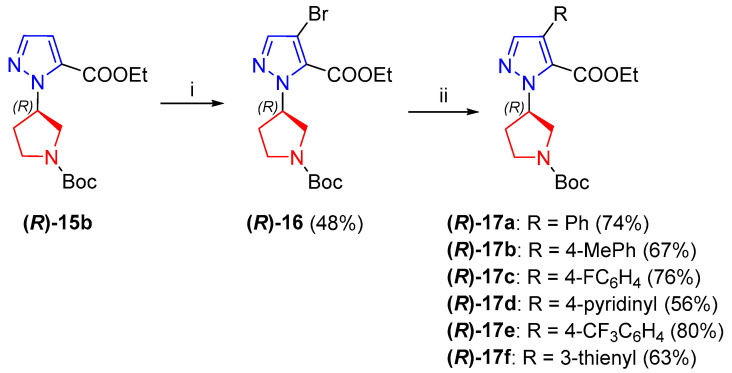
Synthesis of C-4-substituted pyrazole derivatives via bromination and Suzuki–Miyaura cross-coupling. Reaction conditions: (i) NBS, MeCN, r.t., 4h; (ii) R-B(OH)_2_ (**11a**–**f**), 5 mol% Pd(dppf)Cl_2_·DCM, K_3_PO_4_, dioxane/H_2_O, 80 °C, MW, 100 W, 15 min.

To evaluate the transferability of the optimized catalytic system to structurally distinct heterocyclic scaffolds, the *N*-(pyrrolidin-3-yl)-substituted pyrazole derivative **(*R*)-15b** was selected for further functionalization via Suzuki–Miyaura cross-coupling. According to a reported NBS-mediated bromination procedure [76], compound **(*R*)-15b** underwent selective bromination with *N*-bromosuccinimide (NBS) in acetonitrile at room temperature, yielding the corresponding monobrominated pyrazole **(*R*)-16** in a 48% yield (Scheme 6).

Subsequent Suzuki–Miyaura cross-coupling of intermediate **(*R*)-16** was performed under palladium-catalyzed conditions previously optimized for the indole series, namely, Pd(dppf)Cl_2_·DCM (5 mol%) as the catalyst, K_3_PO_4_ as the base, and a dioxane/H_2_O solvent system under microwave irradiation at 80 °C (100 W, 15 min). These conditions enabled the synthesis of aryl- and heteroaryl-substituted pyrazole derivatives **(*R*)-17a**–**f** from the corresponding boronic acids (**11a**–**f**) (Scheme 6). As observed for the indole series, synthesis of the 4-pyridinyl-substituted derivative **(*R*)-17d** required conventional heating at 80 °C for 1 h because of the limited stability of 4-pyridinylboronic acid under microwave conditions. The aryl-substituted products **(*R*)-17a**–**c** and **(*R*)-17e** were obtained in good to excellent yields of 67–80%, indicating that arylboronic acids bearing both electron-donating and electron-withdrawing substituents are well tolerated under the reaction conditions employed. In contrast, the heteroaryl-substituted derivatives **(*R*)-17d** and **(*R*)-17f**, derived from 4-pyridinylboronic acid (**11d**) and 3-thienylboronic acid (**11f**), were isolated in slightly lower yet still synthetically useful yields of 56% and 63%, respectively.

Overall, these results demonstrate that the Suzuki–Miyaura coupling protocol optimized for the indole scaffold is readily applicable to *N*-pyrrolidinyl-substituted pyrazoles. The method efficiently accommodates both aryl and heteroaryl boronic acids, underscoring the generality and robustness of the developed catalytic system for constructing structurally diverse chiral heterocyclic building blocks.

This study was further extended to the synthesis of a series of chiral *N*-(pyrrolidin-3-yl)-1*H*-indazolecarboxylates to evaluate the applicability of the developed alkylation protocol to another structurally distinct heterocyclic scaffold. Because the indazole ring contains two nonequivalent nitrogen atoms, *N*-alkylation can occur at either position, yielding *N*-substituted regioisomers and thereby significantly broadening the range of accessible chiral building blocks.

**Scheme 7:**
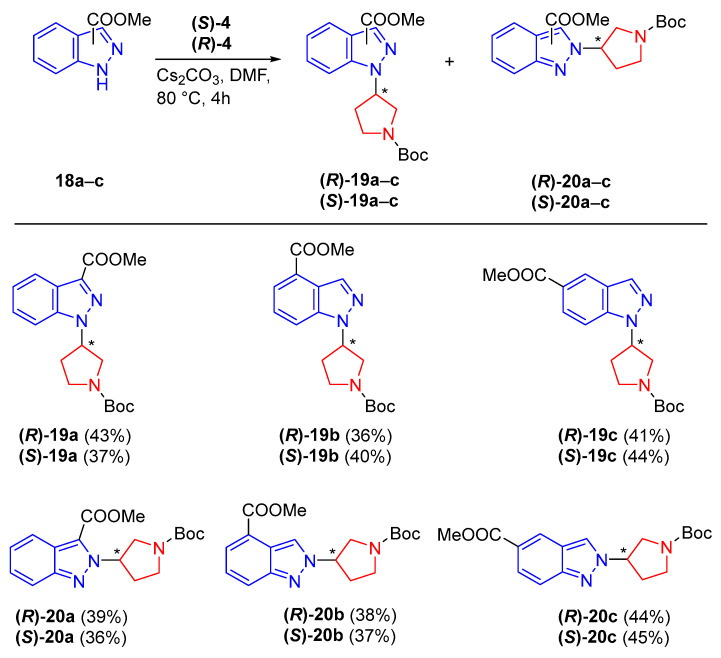
Alkylation of methyl indazole carboxylates **18a**–**c** with (*S*)-*N*-Boc-3-methanesulfonyloxypyrrolidine **(*S*)-4** or (*R*)-*N*-Boc-3-methanesulfonyloxypyrrolidine **(*R*)-4**. Asterisk (*) denotes a stereocenter.

Accordingly, methyl indazole-3-, 4-, and 5-carboxylates **18a**–**c** were alkylated with chiral *N*-(pyrrolidin-3-yl)mesylates in both (*S*)- and (*R*)-configurations under previously optimized nucleophilic substitution conditions (Cs_2_CO_3_, DMF, 80 °C). As shown in Scheme 7, these reactions afforded two series of regioisomeric products, namely the N1-alkylated derivatives **(*R*)-19a**–**c** and **(*S*)-19a**–**c**, and the N2-alkylated derivatives **(*R*)-20a**–**c** and **(*S*)-20a**–**c**. Formation of the N1- and N2-alkylated regioisomers occurred with little regioselectivity, and both products were obtained in comparable yields. This outcome further demonstrates that the developed alkylation protocol is readily transferable to indazole systems and provides efficient access to a diverse set of chiral *N*-substituted indazole building blocks.

### 2.2. NMR Spectroscopic Investigations

The structures of all major biheterocyclic pyrrolidines and their intermediates were confirmed using various spectroscopic techniques, including multinuclear NMR, infrared (IR) spectroscopy, and high-resolution mass spectrometry (HRMS). The main information for structure elucidation was obtained through a combination of standard NMR techniques—including ^1^H-^13^C HMBC, ^1^H-^13^C HSQC, ^1^H-^13^C H2BC, ^1^H-^15^N HMBC, ^1^H-^15^N HSQC, ^1^H-^1^H COSY, ^1^H-^1^H TOCSY, ^1^H-^1^H NOESY, 1D selective NOESY, and 1,1-ADEQUATE experiments—while in some challenging cases, advanced 2D band-selective ^1^H-^13^C HMBC experiments were also performed [77].

**Figure 5:**
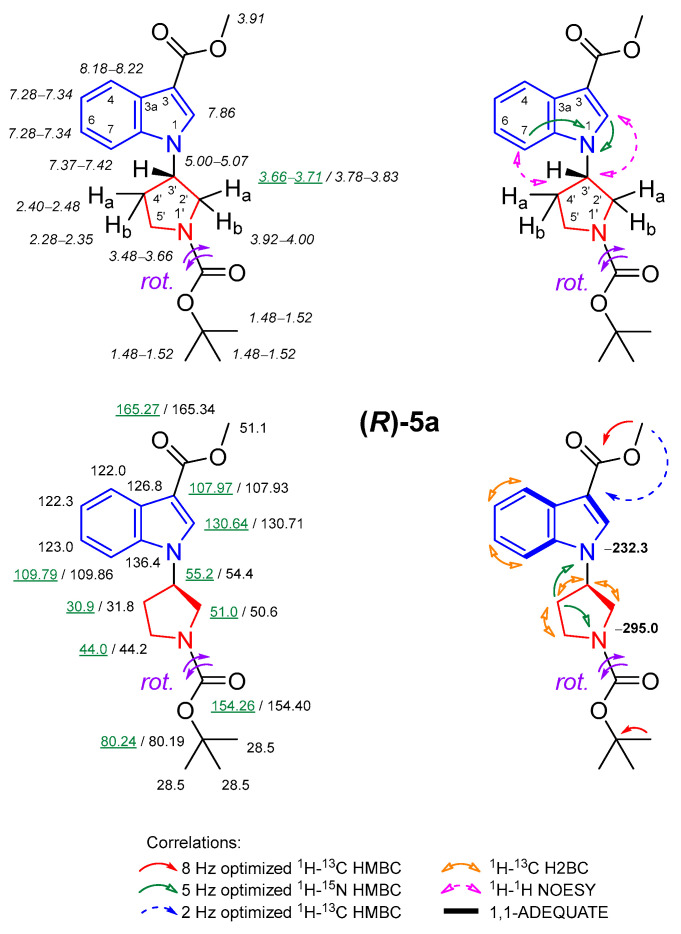
Relevant ^1^H-^13^C HMBC, ^1^H-^13^C H2BC, ^1^H-^15^N HMBC, 1,1-ADEQUATE, and ^1^H-^1^H NOESY correlations, along with ^1^H NMR (italic), ^13^C NMR, and ^15^N NMR (bold) chemical shifts for compound **(*R*)-5a**; in the case of the major rotamer, the chemical shift value is underlined.

For instance, a representative indole–pyrrolidine derivative **(*R*)-5a** was subjected to in-depth NMR analysis. Key structural information was obtained primarily from the ^1^H-^15^N HMBC and ^1^H-^1^H NOESY spectra (Figure 5). Long-range correlations in the ^1^H-^15^N HMBC spectrum unambiguously confirmed the formation of pyrrolidin-3-yl-1*H*-indole-3-carboxylate. The indole N-1 (δ –232.3 ppm) nitrogen showed correlations with the nearby indole methine protons H-2 (δ 7.86 ppm) and H-7 (δ 7.37–7.42 ppm), as well as with the pyrrolidine methylene protons H-4′ (δ 2.28–2.35 and 2.40–2.48 ppm). These H-4′ protons also correlated with the pyrrolidine nitrogen N-1′ (δ –295.0 ppm), which appeared significantly upfield relative to indole N-1, as expected. The ^1^H-^1^H NOESY spectrum of **(*R*)-5a** further elucidated connectivities based on through-space correlations, showing unambiguous NOEs between the pyrrolidine methine proton H-3′ (δ 5.00–5.07 ppm) and the indole methine protons H-2 (δ 7.86 ppm) and H-7 (δ 7.37–7.42 ppm), confirming their proximity and the formation of the indole–pyrrolidine biheterocyclic system.

Initial ^1^H, ^13^C, ^13^C DEPT-90, and ^13^C DEPT-135 NMR spectral analysis of compound **(*R*)-5a** in CDCl_3_ revealed that some ^1^H and ^13^C signals were broadened or doubled, with varying intensities. These signals likely indicate the presence of equilibrating conformers arising from rotation of the *tert*-butoxycarbonyl (Boc) group around a C-N single bond. The amide bond has a partial double-bond character, which restricts C-N rotation. As a result, different conformers remain kinetically stable and can be detected separately via NMR at room temperature. These conformers typically fall within the NMR timescale and appear as distinct signals. Many *N*-acetyl, *N*-trifluoroacetyl, and *N*-Boc-protected heterocyclic compounds, such as oxazolidines [78,79], piperidines [40,80,81], and pyrrolidines [40,80,81,82,83,84], display two sets of NMR signals in deuterated solvents due to a dynamic equilibrium between conformers arising from rotation of their respective nitrogen-protecting groups, with both *syn*- and *anti*-orientations present in these molecules.

A significant broadening or doubling of ^1^H and ^13^C signals, with varying intensities, was also observed in our recent synthesis of a series of 5-(*N*-Boc-cycloaminyl)-1,2-oxazole-4-carboxylates [40]. In that case, some derivatives were treated with trifluoroacetic acid in dichloromethane. This step removed the *N*-Boc protecting group, allowing us to investigate its role in the formation of complex NMR spectra in CDCl_3_ solutions. The reaction produced the corresponding trifluoroacetates, which exhibited only sharp, single ^1^H and ^13^C NMR signals. These results unambiguously confirmed that the combination of the *N*-Boc protecting group and the carboxylate moiety in the compounds caused the observed rotational isomerism in solution during NMR measurements.

For compound **(*R*)-5a**, an in situ *N*-Boc deprotection was performed directly in an NMR tube using a CDCl_3_/CF_3_COOD (1:1, *v*/*v*) mixture, with CF_3_COOD serving as the lock signal during NMR measurements. This strategy was intended to significantly accelerate structure elucidation by avoiding additional synthesis and purification steps. As expected, the ^1^H and ^13^C NMR spectra (referenced to TMS) showed sharp signals without broadening or splitting, unlike those observed in pure CDCl_3_. Additionally, the ^15^N NMR spectral data (referenced to MeNO_2_ in a coaxial capillary), obtained via a ^1^H-^15^N HMBC experiment, showed that the pyrrolidine nitrogen N-1′ (δ −336.6 ppm) resonated significantly upfield relative to the δ −295.0 ppm value observed in CDCl_3_. This indicated successful *N*-Boc deprotection, yielding the expected pyrrolidin-1-ium trifluoroacetate. The indole nitrogen N-1 (δ −234.2 ppm) resonated at a chemical shift similar to that observed in CDCl_3_ (δ −232.3 ppm). Rotational isomerism in the *N*-Boc-protected compound **(*R*)-5a** in CDCl_3_ was most evident in its ^13^C and ^13^C DEPT-135 NMR spectra, particularly in the signals from the 1-(tert-butoxycarbonyl)pyrrolidin-3-yl group. Therefore, comparing these data with those obtained in the CDCl_3_/CF_3_COOD mixture clearly confirmed the absence of rotamers in that mixture. This comparison provides a much faster approach to structural elucidation and verification of rotational isomerism in *N*-Boc-protected compounds containing a carboxylate moiety (see the Supporting Information, Figures S12–S15). A complementary *N*-Boc deprotection and purification approach was also used in dipeptide synthesis (Scheme 3), yielding the corresponding amine **(*R*)-8**, which, as expected, displayed a single set of sharp NMR signals in CDCl_3_, without broadening or splitting (see the Supporting Information, Figures S44–S46).

Ley et al. demonstrated that selective chemical exchange NMR experiments are highly effective for identifying equilibrating rotamers, including chiral *N*-Boc derivatives, from non-equilibrating diastereomers [85]. Chemical exchange NMR methods, such as saturation transfer, are commonly used in these cases. For example, 1D-selective gradient NOESY is often employed. When ^1^H resonances are within the saturation timescale and the irradiated proton exchanges significantly with another proton, the second proton appears as a negative peak. Recently, we successfully applied this strategy for the structural elucidation of a series of chiral methyl 5-(*N*-Boc-cycloaminyl)-1,2-oxazole-4-carboxylates, some of which exhibited rotamer behavior [40].

Chemical exchange can easily be observed in 2D gradient NOESY because exchanging protons appear in phase with the diagonal. Recently, 1D and 2D gradient NOESY experiments were used to distinguish between *E*- and *Z*-isomers of conjugated enamino-carbonyl derivatives [81], and these NOESY techniques have proven highly useful for characterizing tautomeric 10-(1*H*-benzo[*d*]imidazole-2-yl)-3,4-dihydro-1*H*-[1,4]oxazino[4,3-*a*]indol-1-ones, providing valuable insights due to chemical exchange [86]. When 1D and 2D NOESY experiments are used specifically to study chemical exchange, they are called EXSY, which stands for NOESY/exchange spectroscopy, or referred to as NOESY/EXSY [87].

For compound **(*R*)-5a**, the ^1^H NMR spectrum showed two broad, overlapping singlets for the *tert*-butyl protons of the *N*-Boc protecting group at δ 1.49 and 1.51 ppm, indicating the presence of rotamers. However, the separation was too small to allow application of 1D or 2D NOESY/EXSY techniques. As reported by Ley et al. [85], we also confirmed that 1D selective chemical-exchange NMR techniques can identify rotamer peaks in spectra, even when separated by as little as 0.06 ppm at 700 MHz. Adequate signal separation is essential because the saturating pulse does not have a perfectly square frequency profile, increasing the risk that a second negative peak will result from overlap of the irradiation tail with the peak rather than from actual chemical exchange.

**Figure 6:**
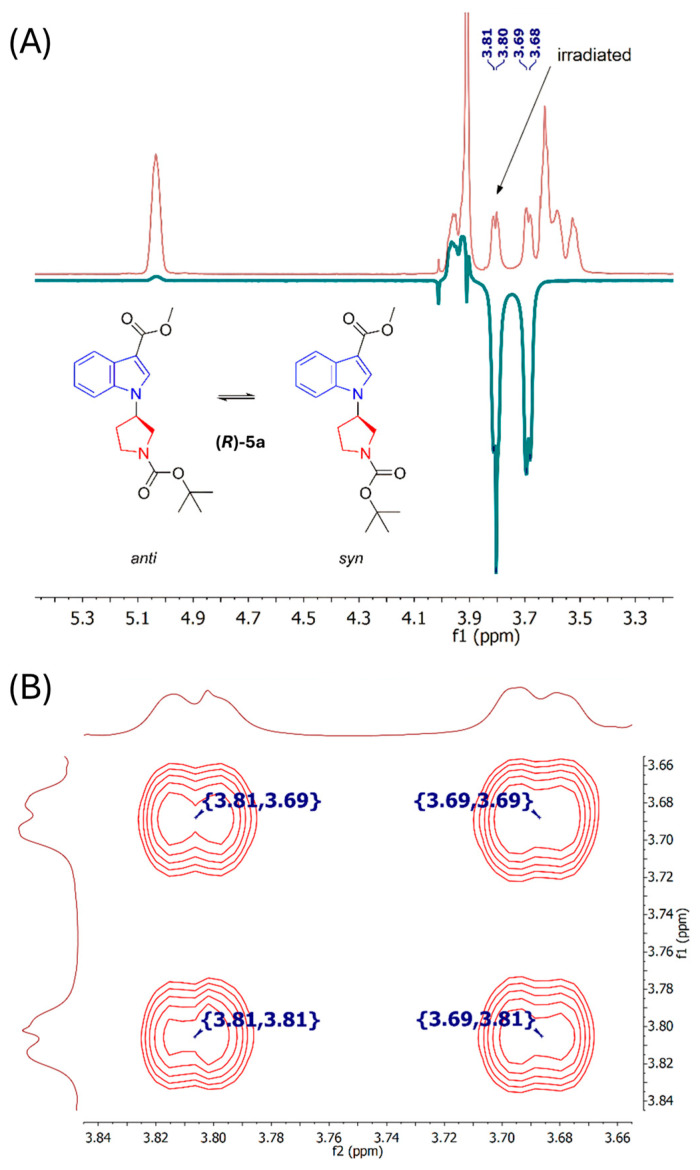
Superimposed ^1^H NMR and 1D gradient NOESY/EXSY spectra (**A**) with selective irradiation of the signal at δ 3.78–3.83 ppm, along with the relevant correlations in the 2D NOESY/EXSY spectra (**B**) (700 MHz, CDCl_3_, 25 °C) for compound **(*R*)-5a**.

Given the minimal separation requirement for ^1^H signals, only one pair of protons from different *syn*- and *anti*-conformers in the entire ^1^H NMR spectrum meets this criterion for **(*R*)-5a**. This pair includes one of the geminal protons, H_a_-2′, resonating at δ 3.78–3.83 and 3.66–3.71 ppm, from the pyrrolidine ring system. The 2D NOESY/EXSY spectrum unambiguously confirmed equilibrium between these protons by showing diagonal cross peaks of the same phase at δ 3.78–3.83 and 3.66–3.71 ppm. Additional confirmation was provided by the 1D-selective gradient NOESY/EXSY experiment. Upon irradiation at δ 3.78–3.83 ppm, two negative signals of the same phase appeared at δ 3.78–3.83 and 3.66–3.71 ppm, indicating chemical exchange and the presence of rotamers (Figure 6). For compound **(*R*)-5a**, the rotamer ratio was determined from the ^1^H NMR spectra to be 1:0.8, based on non-overlapping ^1^H signals.

**Figure 7:**
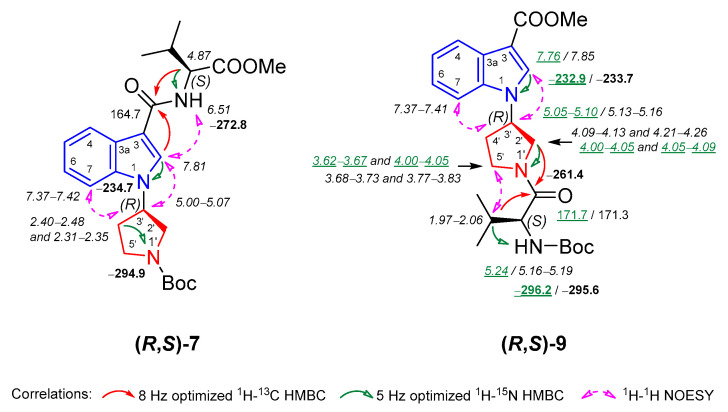
Relevant ^1^H-^13^C HMBC, ^1^H-^15^N HMBC, and ^1^H-^1^H NOESY correlations, along with ^1^H NMR (italic), ^13^C NMR, and ^15^N NMR (bold) chemical shifts for dipeptides **(*R*,*S*)-7** and **(*R*,*S*)-9**; in the case of the major rotamer, the chemical shift value is underlined.

Rotational isomerism was also observed for the dipeptides **(*R,S*)-7** and **(*R,S*)-9**, thereby excluding diastereomeric mixtures, as confirmed by the corresponding 2D NOESY/EXSY spectral data. The structure elucidation strategy followed the same methodology as for the previously described compound **(*R*)-5a**, emphasizing long-range HMBC correlations and using ^1^H-^1^H NOESY spectral data to verify the spatial proximity of distinct heterocyclic moieties. Additionally, the inclusion of ^1^H-^15^N HSQC spectra enabled straightforward identification of protons directly bonded to nitrogen atoms, providing unequivocal evidence of peptide formation. For compound **(*R,S*)-7**, the proton resonating at δ 6.51 ppm showed direct connectivity to the nitrogen resonating at δ −272.8 ppm. In compound **(*R,S*)-9**, the proton of the major rotamer resonating at δ 5.24 ppm displayed an HSQC correlation with the nitrogen resonating at δ −296.2 ppm, and the proton of the minor rotamer resonating at δ 5.16–5.19 ppm correlated with the nitrogen resonating at δ −295.6 ppm (Figure 7).

**Figure 8:**
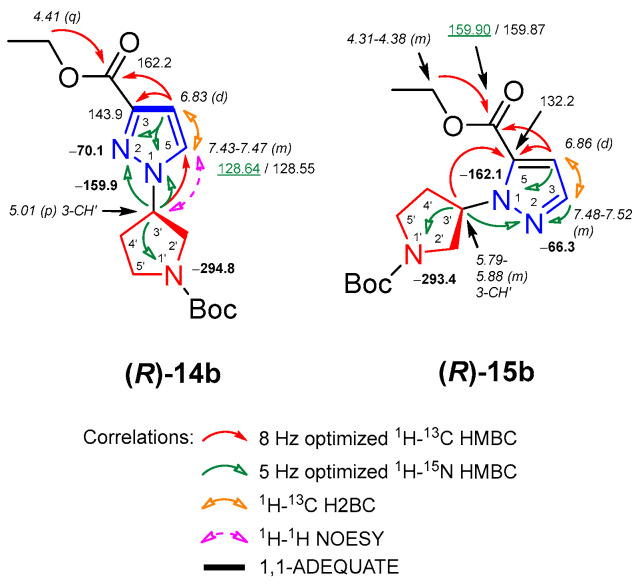
Relevant ^1^H-^13^C HMBC, ^1^H-^13^C H2BC, ^1^H-^15^N HMBC, 1,1-ADEQUATE, and ^1^H-^1^H NOESY correlations, along with ^1^H NMR (italic), ^13^C NMR, and ^15^N NMR (bold) chemical shifts for compounds **(*R*)-14b** and **(*R*)-15b**; in the case of the major rotamer, the chemical shift value is underlined.

Discrimination between the regioisomeric compounds **(*R*)-14b**, **(*R*)-15b**, **(*R*)-19a**–**c**, and **(*R*)-20a**–**c** was achieved based on data from the ^1^H-^13^C HMBC, ^1^H-^15^N HMBC, ^1^H-^1^H NOESY, ^1^H-^13^C H2BC, and 1,1-ADEQUATE experiments. For example, in the ^1^H-^1^H NOESY spectrum of regioisomer **(*R*)-14b**, strong NOEs were observed between the pyrrolidine 3′-H proton (δ 5.01 ppm) and the 5-H proton (δ 7.43–7.47 ppm) of the pyrazole ring system. In contrast, the second regioisomer, **(*R*)-15b**, showed no distinct NOEs between these heterocyclic units. Instead, a weak three-bond correlation was observed between the pyrrolidine 3′-H proton (δ 5.79–5.88 ppm) and the pyrazole C-5 carbon (δ 132.2 ppm) in the ^1^H-^13^C HMBC spectrum (Figure 8).

**Figure 9:**
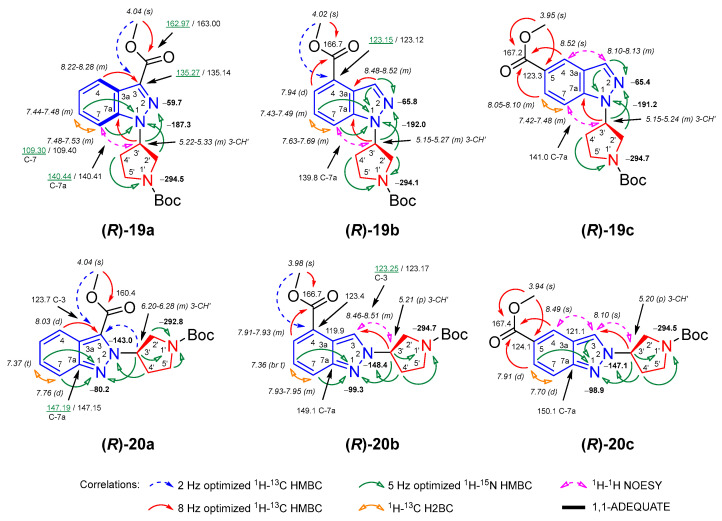
Relevant ^1^H-^13^C HMBC, ^1^H-^13^C H2BC, ^1^H-^15^N HMBC, 1,1-ADEQUATE, and ^1^H-^1^H NOESY correlations, as well as ^1^H NMR (italic), ^13^C NMR, and ^15^N NMR (bold) chemical shifts of regioisomers, **(*R*)-19a**–**c** and **(*R*)-20a**–**c**; in the case of the major rotamer, the chemical shift value is underlined.

Across the series of regioisomeric 1*H*- and 2*H*-indazole carboxylates, the primary information for structure elucidation was obtained from ^1^H-^1^H NOESY, ^1^H-^13^C HMBC, and ^1^H-^15^N HMBC spectral data (Figure 9). For example, in the 1*H*-indazole carboxylates **(*R*)-19a**–**c**, strong NOEs were observed only between the pyrrolidine 3′-H proton and the 7-H proton of the indazole moiety, confirming their proximity. Additionally, the pyrrolidine 3′-H proton showed a weak ^1^H-^13^C HMBC correlation with the C-7a quaternary carbon, which resonated from δ 139.8 to 141.0 ppm in the 1*H*-indazole carboxylates **(*R*)-19a**–**c**. In the case of **(*R*)-19a**, this finding was unambiguously confirmed by a combination of ^1^H-^13^C HSQC and 1,1-ADEQUATE experiments, which showed a strong correlation between the indazole methine C-7 carbon (δ 109.30 and 109.40 ppm for the major and minor rotamers, respectively) and the neighboring quaternary carbon C-7a (δ 140.44 and 140.41 ppm for the major and minor rotamers, respectively).

For the series of regioisomeric 2*H*-indazole carboxylates **(*R*)-20a**–**c**, the structural determination method remained consistent and relied primarily on ^1^H-^1^H NOESY, ^1^H-^13^C HMBC, and ^1^H-^15^N HMBC spectral data. For example, in the case of 2*H*-indazole carboxylates **(*R*)-20b** and **(*R*)-20c**, distinct NOEs were observed only between the pyrrolidine 3′-H proton and the indazole 3-H proton. Additionally, the pyrrolidine 3′-H proton showed a ^1^H-^13^C HMBC correlation with the methine C-3 carbon. As expected, in the case of 2*H*-indazole **(*R*)-20a**, no NOEs were observed between the pyrrolidine and indazole moieties in the ^1^H-^1^H NOESY spectrum, and structural determination mainly depended on a combination of variously optimized standard ^1^H-^13^C HMBC and 2D band-selective ^1^H-^13^C HMBC experiments, which led to the identification of an indazole quaternary C-3 carbon (δ 123.7 ppm). Lastly, the ^1^H-^15^N HMBC spectral data showed long-range correlations between nearby protons of the pyrrolidine and indazole moieties, and the ^15^N-NMR spectral data matched the values reported for *N*-substituted indazole compounds [88,89].

Generally, all the *N*-Boc-substituted biheterocycle–pyrrolidine carboxylates showed NMR spectra with either two sets of signals or noticeable signal broadening, due to a dynamic equilibrium between *syn*- and *anti*-conformers arising from Boc group rotation. Testing some compounds in different deuterated solvents did not significantly alter the conformer equilibrium, although some 2D NMR data, such as correlations, showed slight improvements, particularly in the HMBC spectra. The classical 2D HMBC NMR experiment, run under standard parameters, primarily detects three-bond couplings that depend on dihedral angles (Karplus-type relationship), making it difficult, or even impossible, to observe certain expected correlations in some compounds [87,90]. In our case, for very weak correlations, the 2D band-selective ^1^H-^13^C HMBC experiment proved particularly helpful, even when significant signal broadening was present [77].

## 3. Materials and Methods

### 3.1. General

All reagents and solvents were purchased from commercial sources and used without further purification. Flash column chromatography was performed on silica gel 60 Å (63–200 μm, Merck KGaA, Darmstadt, Germany). Thin-layer chromatography was carried out on silica gel plates (Merck Kieselgel 60 F254) and visualized under UV light (254 nm). Microwave reactions were conducted in a CEM Discover Synthesis Unit (CEM Corp., Matthews, NC, USA) using glass vessels (10 mL capacity) sealed with a septum. Pressure was controlled via a load cell connected to the vessel. The vessel contents were monitored with a calibrated infrared temperature controller mounted beneath the vessel. All experiments were performed with stirring. Melting points were determined on a Büchi M-565 melting point apparatus (Uster, Switzerland) and are uncorrected. Mass spectra were obtained on a Shimadzu LCMS 2020 single-quadrupole liquid chromatography–mass spectrometer (Nakagyo-ku, Japan). IR spectra were recorded on a Bruker Vertex 70v FT-IR spectrometer (Billerica, MA, USA) using neat samples. HRMS spectra were recorded with a Bruker micrOTOF-QIII spectrometer (ESI). Optical rotation data were recorded on a UniPol L SCHMIDT + HAENSCH polarimeter (Berlin, Germany) (the compound concentration (g/100 mL) was automatically included in the calculations). HPLC analysis was carried out on a Shimadzu LC-2030C apparatus with CHIRAL ART Amylose-SA (100 × 4.6 mm I.D.; S-3 μm), CHIRAL ART Cellulose-SJ (100 × 4.6 mm I.D.; S-3 μm), and CHIRAL ART Cellulose-SB (100 × 4.6 mm I.D.; S-3 μm) with a mobile phase of water + 0.1% formic acid/acetonitrile at a flow rate of 1 mL/min, maintaining the column temperature at 36 °C. Single crystals were investigated on a Rigaku XtaLAB Synergy Dualflex HyPix diffractometer (Akishima-shi, Japan). The crystals were kept at 160.0(2) K during data collection. The structures were solved with SHELXT (version 2014/4) [91] using Intrinsic Phasing. The absolute configuration of the asymmetric carbon atoms of the enantiomers has been determined using anomalous copper K-alpha X-ray scattering. For these single crystals, anomalous scattering causes small but measurable differences in intensity between reflections and Friedel’s pairs. Using refinement of the absolute structure parameter (Flack’s *x* parameter) [92], the absolute crystal structure was clearly established. Absolute structure parameter values range from −0.04(7) to 0.05(8) (expected value is 0 (within 3 esd’s) for correct absolute structure). For the calculations, the Olex2.refine refinement package [93] with Levenberg–Marquardt and Gauss–Newton minimization was used. ^1^H NMR, ^13^C NMR and ^15^N NMR spectra in appropriate deuterated solutions at 25 °C were recorded on a Bruker Avance III 700 instrument (700 MHz for ^1^H, 176 MHz for ^13^C) equipped with a 5 mm TCI ^1^H-^13^C/^15^N/D z-gradient cryoprobe. ^19^F NMR spectra (376 MHz, absolute referencing via Ξ ratio) were obtained on a Bruker Avance III 400 using a directly detecting BBO probe. Chemical shifts (*δ*) are expressed in ppm relative to tetramethylsilane (TMS). The ^15^N NMR spectrum was referenced to neat, external nitromethane (coaxial capillary).

### 3.2. Synthetic Procedures

**Synthesis of *tert*-butyl (3*R*)-3-iodopyrrolidine-1-carboxylate ((*R*)-3)**

To a solution of (*S*)-*N*-Boc-3-hydroxypyrrolidine **(*S*)-2** (300 mg, 1.6 mmol) in toluene (15 mL) were added imidazole (327 mg, 4.8 mmol), PPh_3_ (840 mg, 3.2 mmol), and I_2_ (610 mg, 2.4 mmol). The reaction mixture was heated at 110 °C for 4 h. The excess iodine was quenched with a 10% aqueous Na_2_S_2_O_3_ solution (20 mL). The mixture was extracted with EtOAc (3 × 20 mL). The combined organic layer was dried over sodium sulfate, filtered, and concentrated under reduced pressure. The residue was purified by flash column chromatography on silica gel using *n*-hexane/acetone (6/1, *v*/*v*). Yield 386 mg (91%), colorless oil. IR (ν_max_, cm^−1^): 2975 (-CH_2_-), 1689 (C=O), 1391 (-CH_2_-), 1154 (C-O). ^1^H NMR (700 MHz, CDCl_3_): *δ*_H_ 1.45–1.51 (m, 9H, C(CH_3_)_3_), 2.18–2.29 (m, 2H, 4-CH_2_), 3.38–3.47 (m, 1H, 5-CH_A_), 3.53–3.62 (m, 1H, 5-CH_B_), 3.64–3.89 (m, 2H, 2-CH_2_), 4.31–4.39 (m, 1H, 3-CH). ^13^C NMR (176 MHz, CDCl_3_): *δ*_C_ 20.08 and 20.11 (3-CH), 28.61 (C(CH_3_)_3_), 37.74 and 38.52 (4-CH_2_), 44.88 and 45.20 (5-CH_2_), 57.19 and 57.52 (2-CH_2_), 79.84 and 79.91 (C(CH_3_)_3_), 154.26 and 154.40 (Boc C=O). ^15^N NMR (71 MHz, CDCl_3_): *δ*_N_ −292.6. HRMS (ESI TOF): [M + Na]^+^, found 320.0121. [C_9_H_16_INO_2_ + Na]^+^ requires 320.0118.

**Synthesis of *tert*-butyl (3*S*)-3-[(methanesulfonyl)oxy]pyrrolidine-1-carboxylate ((*S*)-4)**

A solution of (*S*)-*N*-Boc-3-hydroxypyrrolidine **(*S*)-2** (2000 mg, 10.68 mmol) and triethylamine (1.49 mL, 10.68 mmol) in dichloromethane (20 mL) was cooled to 0 °C. Methanesulfonyl chloride (0.83 mL, 10.68 mmol) was added. The mixture was allowed to reach room temperature and stirred for 3 h. The mixture was then diluted with dichloromethane (50 mL), washed with water (30 mL), dried over sodium sulfate, filtered, and concentrated under reduced pressure. The crude product was obtained in sufficient purity and used in the subsequent reaction without further purification. Yield: 2780 mg (98%) as a yellow oil. IR (ν_max_, cm^−1^): 2977 (-CH_2_-), 1688 (C=O), 1404 (-CH_2_-), 1354 (-O-SO_2_-), 1163 (C-O). ^1^H NMR (700 MHz, CDCl_3_): *δ*_H_ 1.46 (s, 9H, C(CH_3_)_3_), 2.06–2.19 (m, 1H, 4-CH_A_), 2.21–2.34 (m, 1H, 4-CH_B_), 3.04 (s, 3H, CH_3_), 3.41–3.76 (m, 4H, 2-CH_2_ and 5-CH_2_), 5.24–5.28 (m, 1H, 3-CH). ^13^C NMR (176 MHz, CDCl_3_): *δ*_C_ 28.58 (C(CH_3_)_3_), 31.86 and 32.82 (4-CH_2_), 38.91 (CH_3_), 43.41 and 43.73 (5-CH_2_), 51.90 and 52.33 (2-CH_2_), 79.71 (3-CH), 80.06 (C(CH_3_)_3_), 154.33 (Boc C=O). ^15^N NMR (71 MHz, CDCl_3_): *δ*_N_ −294.0. HRMS (ESI TOF): [M + Na]^+^, found 288.0879. [C_10_H_19_NO_5_S + Na]^+^ requires 288.0876.

**Synthesis of *tert*-butyl (3*R*)-3-[(methanesulfonyl)oxy]pyrrolidine-1-carboxylate ((*R*)-4)**

A solution of (*R*)-*N*-Boc-3-hydroxypyrrolidine **(*R*)-2** (2000 mg, 10.68 mmol) and triethylamine (1.49 mL, 10.68 mmol) in dichloromethane (20 mL) was cooled to 0 °C. Methanesulfonyl chloride (0.83 mL, 10.68 mmol) was added. The mixture was allowed to reach room temperature and stirred for 3 h. The mixture was then diluted with dichloromethane (50 mL), washed with water (30 mL), dried over sodium sulfate, filtered, and concentrated under reduced pressure. The crude product was used directly in the subsequent reaction without further purification. Yield: 2745 mg (97%) as a yellow oil. IR (ν_max_, cm^−1^): 2978 (-CH_2_-), 1682 (C=O), 1404 (-CH_2_-), 1351 (-O-SO_2_-), 1162 (C-O). ^1^H NMR (700 MHz, CDCl_3_): *δ*_H_ 1.46 (s, 9H, C(CH_3_)_3_), 2.09–2.18 (m, 1H, 4-CH_A_), 2.20–2.34 (m, 1H, 4-CH_B_), 3.04 (s, 3H, CH_3_), 3.41–3.72 (m, 4H, 2-CH_2_ and 5-CH_2_), 5.23–5.28 (m, 1H, 3-CH). ^13^C NMR (176 MHz, CDCl_3_): *δ*_C_ 28.58 (C(CH_3_)_3_), 31.86 and 32.82 (4-CH_2_), 38.90 (CH_3_), 43.43 and 43.74 (5-CH_2_), 51.92 and 52.32 (2-CH_2_), 79.68 (3-CH), 80.06 (C(CH_3_)_3_), 154.30 (Boc C=O). ^15^N NMR (71 MHz, CDCl_3_): *δ*_N_ −294.0. HRMS (ESI TOF): [M + Na]^+^, found 288.0878. [C_10_H_19_NO_5_S + Na]^+^ requires 288.0876.

**General procedure for the *N*-alkylation of 1*H*-indolecarboxylates 1a–c with pyrrolidine mesylates (*S*)-4 and (*R*)-4 to afford compounds (*R*)-5a–c and (*S*)-5a–c, respectively.**

To a solution of the appropriate 1*H*-indolecarboxylate (3 mmol) in dimethylformamide (8 mL), (*S*)- or (*R*)-*N*-Boc-3-methanesulfonyloxypyrrolidine (**(*S*)-4** or **(*R*)-4**) (874 mg, 4.2 mmol) and Cs_2_CO_3_ (1467 mg, 4.5 mmol) were added, and the mixture was heated at 80 °C for 4 h. After cooling to room temperature, the mixture was diluted with water (20 mL) and extracted with ethyl acetate (3 × 20 mL). The combined organic layers were washed with brine (20 mL), dried over sodium sulfate, filtered, and concentrated under reduced pressure. The residue was purified by flash column chromatography using an appropriate eluent to afford the corresponding indole derivatives **(*R*)-5a**–**c** or **(*S*)-5a**–**c**.

**Methyl 1-[(3*R*)-1-(*tert*-butoxycarbonyl)pyrrolidin-3-yl]-1*H*-indole-3-carboxylate ((*R*)-5a)**

The reaction was carried out using a general alkylation procedure using 1*H*-indole-3-carboxylate **1a** and (*S*)-1-Boc-3-methanesulfonyloxypyrrolidine **(*S*)-4**, and the product was purified by column chromatography on silica gel using *n*-hexane/ethyl acetate (2/1, *v*/*v*). Yield 950 mg (92%), white solid, mp 116–117 °C. ${\left[\alpha \right]}_{D}^{20}$ = −47.8° (c 1.82, MeOH). IR (ν_max_, cm^−1^): 2971 (-CH_2_-), 1682 (C=O), 1401 (-CH_3_), 1167 (C-O). ^1^H NMR (700 MHz, CDCl_3_): (two rotamers are present in the spectrum, ratio ~1:0.8) *δ*_H_ 1.48–1.52 (m, 9H, C(CH_3_)_3_), 2.28–2.35 (m, 1H, Pyrr 4-H_A_), 2.40–2.48 (m, 1H, Pyrr 4-H_B_), 3.48–3.66 (m, 2H, Pyrr 5-CH_2_), 3.66–3.71 (m, 0.55H, Pyrr 2-H_A_ of major rotamer), 3.78–3.83 (m, 0.45H, Pyrr 2-H_A_ of minor rotamer), 3.88–3.99 (m, 4H, CH_3_ and Pyrr 2-H_B_), 5.00–5.07 (m, 1H, Pyrr 3-H), 7.28–7.34 (m, 2H, 5-H and 6-H), 7.37–7.42 (m, 1H, 7-H), 7.86 (s, 1H, 2-H), 8.18–8.22 (m, 1H, 4-H). ^13^C NMR (176 MHz, CDCl_3_): *δ*_C_ 28.46 (C(CH_3_)_3_), 30.88 and 31.75 (Pyrr 4-CH_2_), 43.96 and 44.22 (Pyrr 5-CH_2_), 50.59 and 51.01 (Pyrr 2-CH_2_), 51.05 (CH_3_), 54.43 and 55.17 (Pyrr C-3), 80.19 and 80.24 (C(CH_3_)_3_), 107.93 and 107.97 (C-3), 109.79 and 109.86 (C-7), 122.00 (C-4), 122.30 (C-5), 123.04 (C-6), 126.78 (C-3a), 130.64 and 130.71 (C-2), 136.44 (C-7a), 154.26 and 154.40 (Boc C=O), 165.27 and 165.34 (C=O). ^15^N NMR (71 MHz, CDCl_3_): *δ*_N_ −295.0 (Pyrr N-1), −232.3 (N-1). HRMS (ESI TOF): [M + Na]^+^, found 367.1624. [C_19_H_24_N_2_O_4_ + Na]^+^ requires 367.1628.

**Methyl 1-[(3*S*)-1-(*tert*-butoxycarbonyl)pyrrolidin-3-yl]-1*H*-indole-3-carboxylate ((*S*)-5a)**

The reaction was carried out using a general alkylation procedure using 1*H*-indole-3-carboxylate **1a** and (*R*)-1-Boc-3-methanesulfonyloxypyrrolidine **(*R*)-4**, and the product was purified by column chromatography on silica gel using *n*-hexane/ethyl acetate (2/1, *v*/*v*). Yield 971 mg (94%), white solid, mp 116–117 °C. ${\left[\alpha \right]}_{D}^{20}$ = 47.7° (c 1.96, MeOH). IR (ν_max_, cm^−1^): 2971 (-CH2-), 1686 (C=O), 1404 (-CH_3_), 1168 (C-O). ^1^H NMR (700 MHz, CDCl_3_): (two rotamers are present in the spectrum, ratio ~1:0.8) *δ*_H_ 1.48–1.52 (m, 9H, C(CH_3_)_3_), 2.28–2.35 (m, 1H, Pyrr 4-H_A_), 2.40–2.48 (m, 1H, Pyrr 4-H_B_), 3.49–3.66 (m, 2H, Pyrr 5-CH_2_), 3.66–3.72 (m, 0.55H, Pyrr 2-H_A_ of major rotamer), 3.78–3.83 (m, 0.45H, Pyrr 2-H_A_ of minor rotamer), 3.88–3.99 (m, 4H, CH_3_ and Pyrr 2-H_B_), 5.00–5.07 (m, 1H, Pyrr 3-H), 7.28–7.32 (m, 2H, 5-H and 6-H), 7.38–7.42 (m, 1H, 7-H), 7.86 (s, 1H, 2-H), 8.18–8.22 (m, 1H, 4-H). ^13^C NMR (176 MHz, CDCl_3_): *δ*_C_ 28.46 (C(CH_3_)_3_), 30.87 and 31.74 (Pyrr 4-CH_2_), 43.96 and 44.21 (Pyrr 5-CH_2_), 50.58 and 51.01 (Pyrr 2-CH_2_), 51.06 (CH_3_), 54.43 and 55.17 (Pyrr C-3), 80.21 and 80.26 (C(CH_3_)_3_), 107.92 and 107.97 (C-3), 109.79 and 109.87 (C-7), 121.99 (C-4), 122.30 (C-5), 123.04 (C-6), 126.78 (C-3a), 130.65 and 130.72 (C-2), 136.44 (C-7a), 154.27 and 154.40 (Boc C=O), 165.28 and 165.36 (C=O). ^15^N NMR (71 MHz, CDCl_3_): *δ*_N_ −295.1 (Pyrr N-1), −232.3 (N-1). HRMS (ESI TOF): [M + Na]^+^, found 367.1630. [C_19_H_24_N_2_O_4_ + Na]^+^ requires 367.1628.

**Methyl 1-[(3*R*)-1-(*tert*-butoxycarbonyl)pyrrolidin-3-yl]-1*H*-indole-5-carboxylate ((*R*)-5b)**

The reaction was carried out using a general alkylation procedure using 1*H*-indole-5-carboxylate **1b** and (*S*)-*N*-Boc-3-methanesulfonyloxypyrrolidine **(*S*)-4**, and the product was purified by column chromatography on silica gel using n-hexane/ethyl acetate (4/1, *v*/*v*). Yield 827 mg (80%), white solid, mp 117–118 °C. ${\left[\alpha \right]}_{D}^{20}$ = −19.8° (c 1.76, CHCl_3_). IR (ν_max_, cm^−1^): 2974 (-CH_2_-), 1693 (C=O), 1395 (-CH_3_), 1124 (C-O). ^1^H NMR (700 MHz, CDCl_3_): (two rotamers are present in the spectrum, ratio ~1:0.8) *δ*_H_ 1.46–1.52 (m, 9H, C(CH_3_)_3_), 2.23–2.31 (m, 1H, Pyrr 4-H_A_), 2.37–2.45 (m, 1H, Pyrr 4-H_B_), 3.47–3.68 (m, 2.55H, Pyrr 5-CH_2_ and Pyrr 2-H_A_, of major rotamer), 3.77–3.82 (m, 0.45H, Pyrr 2-H_A_, of minor rotamer), 3.86–3.98 (m, 4H, CH_3_ and Pyrr 2-H_B_), 5.01–5.09 (m, 1H, Pyrr 3-H), 6.62–6.65 (m, 1H, 3-H), 7.19–7.22 (m, 1H, 2-H), 7.38 (d, *J* = 8.7 Hz, 1H, 7-H), 7.93 (d, *J* = 8.7 Hz, 1H, 6-H), 8.40 (s, 1H, 4-H). ^13^C NMR (176 MHz, CDCl_3_): *δ*_C_ 28.48 (C(CH_3_)_3_), 31.09 and 32.09 (Pyrr 4-CH_2_), 44.10 and 44.36 (Pyrr 5-CH_2_), 50.45 and 51.03 (Pyrr 2-CH_2_), 51.88 (CH_3_), 54.17 and 54.89 (Pyrr C-3), 80.06 (C(CH_3_)_3_), 103.74 and 103.87 (C-3), 108.91 and 108.94 (C-7), 121.93 (C-5), 123.16 (C-6), 124.13 (C-4), 125.47 (C-2), 128.19 and 128.23 (C-3a), 138.37 (C-7a), 154.31 and 154.40 (Boc C=O), 168.06 (C=O). ^15^N NMR (71 MHz, CDCl_3_): *δ*_N_ −294.5 (Pyrr N-1), −237.8 (N-1). HRMS (ESI TOF): [M + Na]^+^, found 367.1628. [C_19_H_24_N_2_O_4_ + Na]^+^ requires 367.1628.

**Methyl 1-[(3*S*)-1-(*tert*-butoxycarbonyl)pyrrolidin-3-yl]-1*H*-indole-5-carboxylate ((*S*)-5b)**

The reaction was carried out using a general alkylation procedure using 1*H*-indole-5-carboxylate **1b** and (*R*)-1-Boc-3-methanesulfonyloxypyrrolidine **(*R*)-4**, and the product was purified by column chromatography on silica gel using *n*-hexane/ethyl acetate (4/1, *v*/*v*). Yield 856 mg (83%), white solid, mp 117–118 °C. ${\left[\alpha \right]}_{D}^{20}$ = 19.6° (c 1.76, CHCl_3_). IR (ν_max_, cm^−1^): 2969 (-CH_2_-), 1669 (C=O), 1409 (-CH_3_), 1132 (C-O). ^1^H NMR (700 MHz, CDCl_3_): (two rotamers are present in the spectrum, ratio ~1:0.8) *δ*_H_ 1.46–1.52 (m, 9H, C(CH_3_)_3_), 2.22–2.31 (m, 1H, Pyrr 4-H_A_), 2.37–2.44 (m, 1H, Pyrr 4-H_B_), 3.48–3.67 (m, 2.55H, Pyrr 5-CH_2_ and Pyrr 2-H_A_, of major rotamer), 3.75–3.82 (m, 0.45H, Pyrr 2-H_A_, of minor rotamer), 3.87–3.96 (m, 4H, CH_3_ and Pyrr 2-H_B_), 5.01–5.08 (m, 1H, Pyrr 3-H), 6.61–6.65 (m, 1H, 3-H), 7.18–7.22 (m, 1H, 2-H), 7.37 (d, *J* = 8.7 Hz, 1H, 7-H), 7.93 (d, *J* = 8.7 Hz, 1H, 6-H), 8.39 (s, 1H, 4-H). ^13^C NMR (176 MHz, CDCl_3_): *δ*_C_ 28.48 (C(CH_3_)_3_), 31.08 and 32.07 (Pyrr 4-CH_2_), 44.09 and 44.37 (Pyrr 5-CH_2_), 50.44 and 51.03 (Pyrr 2-CH_2_), 51.88 (CH_3_), 54.17 and 54.88 (Pyrr C-3), 80.07 (C(CH_3_)_3_), 103.73 and 103.86 (C-3), 108.91 and 108.94 (C-7), 121.92 (C-5), 123.15 (C-6), 124.12 (C-4), 125.48 (C-2), 128.20 and 128.23 (C-3a), 138.37 (C-7a), 154.32 and 154.41 (Boc C=O), 168.06 (C=O). ^15^N NMR (71 MHz, CDCl_3_): *δ*_N_ −294.5 (Pyrr N-1), −237.8 (N-1). HRMS (ESI TOF): [M + Na]^+^, found 367.1627. [C_19_H_24_N_2_O_4_ + Na]^+^ requires 367.1628.

**Methyl 1-[(3*R*)-1-(*tert*-butoxycarbonyl)pyrrolidin-3-yl]-1*H*-indole-6-carboxylate ((*R*)-5c)**

The reaction was carried out using a general alkylation procedure using 1*H*-indole-6-carboxylate **1c** and (*S*)-1-Boc-3-methanesulfonyloxypyrrolidine **(*S*)-4**, and the product was purified by column chromatography on silica gel using *n*-hexane/ethyl acetate (2/1, *v*/*v*). Yield 682 mg (66%), white solid, mp 96–97 °C. ${\left[\alpha \right]}_{D}^{20}$ = 31.4° (c 1.85, MeOH). IR (ν_max_, cm^−1^): 2981 (-CH_2_-), 1697 (C=O), 1394 (-CH_3_), 1136 (C-O). ^1^H NMR (700 MHz, CDCl_3_): (two rotamers are present in the spectrum, ratio ~1:0.8) *δ*_H_ 1.47–1.51 (m, 9H, C(CH_3_)_3_), 2.21–2.31 (m, 1H, Pyrr 4-H_A_), 2.39–2.46 (m, 1H, Pyrr 4-H_B_), 3.47–3.67 (m, 2.55H, Pyrr 5-CH_2_ and Pyrr 2-H_A_, of major rotamer), 3.78–3.83 (m, 0.45H, Pyrr 2-H_A_, of minor rotamer), 3.88–3.97 (m, 4H, CH_3_ and Pyrr 2-H_B_), 5.08–5.16 (m, 1H, Pyrr 3-H), 6.55–6.59 (m, 1H, 3-H), 7.28–7.32 (m, 1H, 2-H), 7.64 (d, *J* = 8.3 Hz, 1H, 4-H), 7.81–7.83 (m, *J* = 8.3 Hz, 1H, 5-H), 8.15 (s, 1H, 7-H). ^13^C NMR (176 MHz, CDCl_3_): *δ*_C_ 28.49 (C(CH_3_)_3_), 31.22 and 32.28 (Pyrr 4-CH_2_), 44.10 and 44.37 (Pyrr 5-CH_2_), 50.56 and 51.23 (Pyrr 2-CH_2_), 52.02 (CH_3_), 54.04 and 54.73 (Pyrr C-3), 80.00 and 80.04 (C(CH_3_)_3_), 102.54 and 102.68 (C-3), 111.59 and 111.64 (C-7), 120.68 (C-4), 120.87 (C-5), 123.43 (C-6), 127.29 and 127.35 (C-2), 132.27 and 132.32 (C-3a), 135.40 (C-7a), 154.32 and 154.41 (Boc C=O), 168.09 (C=O). ^15^N NMR (71 MHz, CDCl_3_): *δ*_N_ −294.5 (Pyrr N-1), −237.4 (N-1). HRMS (ESI TOF): [M + Na]^+^, found 367.1628. [C_19_H_24_N_2_O_4_ + Na]^+^ requires 367.1628.

**Methyl 1-[(3*S*)-1-(*tert*-butoxycarbonyl)pyrrolidin-3-yl]-1*H*-indole-6-carboxylate ((*S*)-5c)**

The reaction was carried out using a general alkylation procedure using 1*H*-indole-6-carboxylate **1c** and (*R*)-1-Boc-3-methanesulfonyloxypyrrolidine **(*R*)-4**, and the product was purified by column chromatography on silica gel using *n*-hexane/ethyl acetate (2/1, *v*/*v*). Yield 713 mg (69%), white solid, mp 96–97 °C. ${\left[\alpha \right]}_{D}^{20}$ = −31.4° (c 1.83, MeOH). IR (ν_max_, cm^−1^): 2981 (-CH_2_-), 1697 (C=O), 1394 (-CH_3_), 1136 (C-O). ^1^H NMR (700 MHz, CDCl_3_): (two rotamers are present in the spectrum, ratio ~1:0.8) *δ*_H_ 1.47–1.52 (m, 9H, C(CH_3_)_3_), 2.23–2.31 (m, 1H, Pyrr 4-H_A_), 2.39–2.47 (m, 1H, Pyrr 4-H_B_), 3.49–3.66 (m, 2.55H, Pyrr 5-CH_2_ and Pyrr 2-H_A_, of major rotamer), 3.79–3.84 (m, 0.45H, Pyrr 2-H_A_, of minor rotamer), 3.89–3.96 (m, 4H, CH_3_ and Pyrr 2-H_B_), 5.10–5.17 (m, 1H, Pyrr 3-H), 6.57–6.60 (m, 1H, 3-H), 7.28–7.32 (m, 1H, 2-H), 7.64 (d, *J* = 8.3 Hz, 1H, 4-H), 7.80–7.84 (m, 1H, 5-H), 8.15 (s, 1H, 7-H). ^13^C NMR (176 MHz, CDCl_3_): *δ*_C_ 28.49 (C(CH_3_)_3_), 31.23 and 32.30 (Pyrr 4-CH_2_), 44.11 and 44.37 (Pyrr 5-CH_2_), 50.57 and 51.24 (Pyrr 2-CH_2_), 52.03 (CH_3_), 54.05 and 54.73 (Pyrr C-3), 80.01 and 80.05 (C(CH_3_)_3_), 102.55 and 102.69 (C-3), 111.59 and 111.64 (C-7), 120.69 (C-4), 120.88 (C-5), 123.44 (C-6), 127.29 and 127.34 (C-2), 132.28 and 132.32 (C-3a), 135.40 (C-7a), 154.32 and 154.41 (Boc C=O), 168.11 (C=O). ^15^N NMR (71 MHz, CDCl_3_): *δ*_N_ −294.5 (Pyrr N-1), −237.4 (N-1). HRMS (ESI TOF): [M + Na]^+^, found 367.1623. [C_19_H_24_N_2_O_4_ + Na]^+^ requires 367.1628.

**Synthesis of 1-[(3*R*)-1-(*tert*-butoxycarbonyl)pyrrolidin-3-yl]-1*H*-indole-3-carboxylic acid ((*R*)-6)**

The ester **(*R*)-5a** (150 mg) was dissolved in a MeOH and THF mixture (1/1, *v*/*v*, 2 mL) and treated with 1 N LiOH (1 mL). The solution was stirred at 60 °C for 6 h. After removal of the solvent in vacuo, the residue was purified by flash chromatography on silica gel using DCM/MeOH (20/1, *v*/*v*). Yield 141 mg (98%), white solid, mp 96–97 °C. IR (ν_max_, cm^−1^): 1682 (C=O), 1530 (C=C), 1399 (CH_3_), 1163 (C-O). ^1^H NMR (700 MHz, CDCl_3_): (two rotamers are present in the spectrum, ratio ~1:1) *δ*_H_ 1.47–1.54 (m, 9H, C(CH_3_)_3_), 2.31–2.38 (m, 1H, Pyrr 4-H_A_), 2.43–2.45 (m, 1H, Pyrr 4-H_B_), 3.52–3.68 (m, 2H, Pyrr 5-CH_2_), 3.68–3.74 (m, 0.5H, Pyrr 2-H_A_, of rotamer A), 3.79–3.86 (m, 0.5H, Pyrr 2-H_A_, of rotamer B), 3.92–3.97 (m, 0.5H, Pyrr 2-H_A_, of rotamer A), 3.97–4.03 (m, 0.5H, Pyrr 2-H_A_, of rotamer B), 5.02–5.09 (m, 1H, Pyrr 3-H), 7.30–7.37 (m, 2H, 5-H and 6-H), 7.40–7.44 (m, 1H, 7-H), 7.98 (s, 1H, 2-H), 8.24–8.29 (m, 1H, 4-H). ^13^C NMR (176 MHz, CDCl_3_): *δ*_C_ 28.47 (C(CH_3_)_3_), 30.83 and 31.68 (Pyrr 4-CH_2_), 43.96 and 44.22 (Pyrr 5-CH_2_), 50.60 and 50.95 (Pyrr 2-CH_2_), 54.58 and 55.32 (Pyrr C-3), 80.33 (C(CH_3_)_3_), 107.33 and 107.40 (C-3), 109.92 and 109.96 (C-7), 122.2 (C-4), 122.6 (C-5), 123.2 (C-6), 127.0 (C-3a), 132.0 (C-2), 136.6 (C-7a), 154.28 and 154.43 (Boc C=O), 170.1 (C=O). ^15^N NMR (71 MHz, CDCl_3_): *δ*_N_ −294.1 (Pyrr N-1), −230.2 (N-1). HRMS (ESI TOF): [M + Na]^+^, found 353.1473. [C_18_H_22_N_2_O_4_ + Na]^+^ requires 353.1472.

**Synthesis of methyl *N*-{1-[(3*R*)-1-(*tert*-butoxycarbonyl)pyrrolidin-3-yl]-1*H*-indole-3-carbonyl}-*L*-valinate ((*R*,*S*)-7)**

DIPEA (55 µL, 0.3 mmol) was added to a mixture of carboxylic acid **(*R*)-6** (100 mg, 0.3 mmol) and HATU (115 mg, 0.3 mmol) in anhydrous DMF (1 mL), and the solution was stirred at r.t. for 5 min. In a separate flask, *L*-valine methyl ester hydrochloride (51 mg, 0.3 mmol) and DIPEA (110 µL, 0.6 mmol) were dissolved in anhydrous DMF (1 mL). This solution was transferred dropwise to the activated acid mixture, and the resulting reaction mixture was stirred at room temperature for 1 h. The reaction mixture was diluted with EtOAc (10 mL), washed with 1 M KHSO_4_ (10 mL), 1 M NaHCO_3_ (10 mL), and brine (10 mL). The organic layer was dried with anhydrous sodium sulfate, filtered, and concentrated under reduced pressure. The crude product was purified by flash chromatography on silica gel using acetone/*n*-hexane (1/3, *v*/*v*). Yield 109 mg (79%), white solid, mp 61–62 °C. IR (ν_max_, cm^−1^): 3350 (CO-NH), 2966 (-CH_2_-), 1693 (C=O), 1402 (-CH_3_), 1164 (C-O). ^1^H NMR (700 MHz, CDCl_3_): (two rotamers are present in the spectrum, ratio ~1:1) *δ*_H_ 1.02 (d, *J* = 6.9 Hz, 3H, CH(CH_3_)_2_), 1.05 (d, *J* = 6.9 Hz, 3H, CH(CH_3_)_2_), 1.46–1.52 (m, 9H, C(CH_3_)_3_), 2.28–2.31 (m, 1H, NHCHC*H*), 2.31–2.35 (m, 1H, Pyrr 4-H_A_), 2.40–2.48 (m, 1H, Pyrr 4-H_B_), 3.53–3.65 (m, 2H, Pyrr 5-CH_2_), 3.65–3.69 (m, 0.5H, Pyrr 2-H_A_, of rotamer A), 3.74–3.78 (m, 0.5H, Pyrr 2-H_A_, of rotamer B), 3.79 (s, 3H, CH_3_), 3.89–3.94 (m, 0.5H, Pyrr 2-H_A_, of rotamer A), 3.94–4.00 (m, 0.5H, Pyrr 2-H_A_, of rotamer B), 4.87 (dd, *J* = 8.7, 4.9 Hz, 1H, NHC*H*CH), 4.99–5.08 (m, 1H, Pyrr 3-H), 6.51 (d, *J* = 7.4 Hz, 1H, NH), 7.29–7.35 (m, 2H, 5-H and 6-H), 7.40–7.45 (m, 1H, 7-H), 7.81 (s, 1H, 2-H), 7.98–8.03 (m, 1H, 4-H). ^13^C NMR (176 MHz, CDCl_3_): *δ*_C_ 18.1 and 19.1 (CH(CH_3_)_2_), 28.5(C(CH_3_)_3_), 30.9 and 31.6 (Pyrr 4-CH_2_), 31.7 (NHCH*C*H), 44.0 and 44.3 (Pyrr 5-CH_2_), 50.58 and 50.85 (Pyrr 2-CH_2_), 52.2 (CH_3_), 54.3 and 55.1 (Pyrr C-3), 56.9 (NH*C*HCH), 80.14 and 80.18 (C(CH_3_)_3_), 110.22 and 110.30 (C-7), 111.57 and 111.65 (C-3), 120.18 and 120.29 (C-4), 122.1 (C-5), 122.9 (C-6), 125.4 (C-3a), 128.24 and 128.35 (C-2), 136.6 (C-7a), 154.26 and 154.35 (Boc C=O), 164.7 (NH C=O), 173.1 (COOMe C=O). ^15^N NMR (71 MHz, CDCl_3_): *δ*_N_ −294.9 (Pyrr N-1), −272.8 (NH), −234.7 (N-1). HRMS (ESI TOF): [M + Na]^+^, found 466.2318. [C_24_H_33_N_3_O_5_ + Na]^+^ requires 466.2312.

**Synthesis of methyl 1-[(3*R*)-pyrrolidin-3-yl]-1*H*-indole-3-carboxylate ((*R*)-8)**

To a solution of compound (R)-5a (150 mg, 0.45 mmol) in dichloromethane (2 mL) was added trifluoroacetic acid (1 mL), and the mixture was stirred at room temperature for 15 min. After removal of the solvent in vacuo, the residue was dissolved in EtOAc (20 mL), washed with 1 M Na_2_CO_3_ (10 mL) and brine (10 mL), dried over sodium sulfate, filtered, and concentrated under reduced pressure to afford the title compound (R)-8 as white crystals (90 mg, 84%), mp 160–161 °C. IR (ν_max_, cm^−1^): 3325 (N–H), 2942 (-CH_2_-), 1685 (C=O), 1450 (-CH_2_-), 1240 (C–O), 1160 (C–O). ^1^H NMR (700 MHz, CDCl_3_): δ_H_ 2.41–2.48 (m, 1H, Pyrr 4-CH_A_), 2.56–2.64 (m, 1H, Pyrr 4-CH_B_), 3.49–3.53 (m, 1H, Pyrr 5-CH_A_), 3.56–3.61 (m, 2H, Pyrr 5-CH_B_ and Pyrr 2-CH_A_), 3.81 (s, 3H, CH_3_), 3.86–3.90 (m, 1H, Pyrr 2-CH_B_), 5.23–5.29 (m, 1H, Pyrr 3-H), 7.20–7.27 (m, 2H, 5-H and 6-H), 7.33 (d, J = 7.7 Hz, 1H, 7-H), 8.04 (s, 1H, 2-H), 8.09 (d, J = 7.7 Hz, 1H, 4-H), 10.33 (s, 1H, NH). ^13^C NMR (176 MHz, CDCl_3_): δ_C_ 29.9 (Pyrr 4-CH_2_), 43.3 (Pyrr 5-CH_2_), 47.7 (Pyrr 2-CH_2_), 50.2 (CH_3_), 53.1 (Pyrr 3-CH), 108.1 (C-3), 108.6 (C-7), 121.2 (C-4), 121.7 (C-5), 122.61 (C-6), 125.59 (C-3a), 129.3 (C-2), 135.5 (C-7a), 164.3 (C=O). ^15^N NMR (71 MHz, CDCl_3_): δ_N_ −335.7 (Pyrr N-1), −237.4 (N-1). HRMS (ESI TOF): [M + H]^+^, found 245.1285. [C_14_H_16_N_2_O_2_ + H]^+^ requires 245.1287.

**Synthesis of methyl 1-{(3*R*)-1-[*N*-(*tert*-butoxycarbonyl)-*L*-valyl]pyrrolidin-3-yl}-1*H*-indole-3-carboxylate ((*R*,*S*)-9)**

DIPEA (55 µL, 0.3 mmol) was added to a mixture of Boc-*L*-valine (71 mg, 0.3 mmol) and HATU (124 mg, 0.3 mmol) in anhydrous DMF (1 mL), and the solution was stirred at r.t. for 5 min. In a separate flask, amine **(*R*)-8** (51 mg, 0.3 mmol) and DIPEA (110 µL, 0.6 mmol) were dissolved in anhydrous DMF (1 mL). This solution was transferred dropwise to the activated acid mixture, and the resulting reaction mixture was stirred at room temperature for 1 h. The reaction mixture was diluted with EtOAc (10 mL), washed with 1 M KHSO_4_ (10 mL), 1 M NaHCO_3_ (10 mL), and brine (10 mL). The organic layer was dried with anhydrous sodium sulfate, filtered, and concentrated under reduced pressure. The crude product was purified by flash chromatography on silica gel using acetone/*n*-hexane (1/2, *v*/*v*). Yield 109 mg (73%), white solid, mp 65–66 °C. IR (ν_max_, cm^−1^): 3313 (CO-NH), 2967 (-CH_2_-), 1700 (C=O), 1435 (-CH_3_), 1168 (C-O). ^1^H NMR (700 MHz, CDCl_3_): (two rotamers are present in the spectrum, ratio ~1:0.4) *δ*_H_ 0.95–1.00 (m, 1.8H, CH(CH_3_)_2_, of minor rotamer), 1.00 (d, *J* = 6.9 Hz, 2.1H, CH(CH_3_)_A_, of major rotamer), 1.03 (d, *J* = 6.7 Hz, 2.1H, CH(CH_3_)_B_, of major rotamer), 1.40 (s, 2.6H, C(CH_3_)_3_, of minor rotamer), 1.45 (s, 6.4H, C(CH_3_)_3_, of major rotamer), 1.97–2.06 (m, 1H, NHCHC*H*, of both rotamers), 2.30–2.36 (m, 0.3H, Pyrr 4-H_A_, of minor rotamer), 2.37–2.43 (m, 0.7H, Pyrr 4-H_A_, of major rotamer), 2.43–2.49 (m, 0.3H, Pyrr 4-H_B_, of minor rotamer), 2.51–2.59 (m, 0.7H, Pyrr 4-H_B_, of major rotamer), 3.62–3.67 (m, 0.7H, Pyrr 5-H_A_, of major rotamer), 3.68–3.73 (m, 0.3H, Pyrr 5-H_A_, of minor rotamer), 3.77–3.83 (m, 0.3H, Pyrr 5-H_B_, of minor rotamer), 3.88 (s, 2.1H, CH_3_, of major rotamer), 3.88 (s, 0.9H, CH_3_, of minor rotamer), 4.00–4.05 (m, 1.4H, Pyrr 5-H_B_, of major rotamer and Pyrr 2-H_A_, of major rotamer), 4.05–4.09 (m, 0.7H, Pyrr 2-H_B_, of major rotamer), 4.11 (dd, *J* = 11.3, 6.8 Hz, 0.3H, Pyrr 2-H_A_, of minor rotamer), 4.13–4.17 (m, 0.3H, NHC*H*CH, of minor rotamer), 4.24 (dd, *J* = 11.3, 4.5 Hz, 0.3H, Pyrr 2-H_B_, of minor rotamer), 4.28 (dd, *J* = 9.2, 6.9 Hz, 0.7H, NHC*H*CH, of major rotamer), 5.05–5.10 (m, 0.7H, Pyrr 3-H, of major rotamer), 5.13–5.16 (m, 0.3H, Pyrr 3-H, of minor rotamer), 5.16–5.19 (m, 0.3H, NH, of minor rotamer), 5.24 (d, *J* = 9.4 Hz, 0.7H, NH, of major rotamer), 7.29–7.34 (m, 2H, 5-H and 6-H, of both rotamers), 7.37–7.41 (m, 1H, 7-H, of both rotamers), 7.76 (s, 0.7H, 2-H, of major rotamer), 7.85 (s, 0.3H, 2-H, of minor rotamer), 8.18–8.21 (m, 0.7H, 4-H, of major rotamer), 8.21–8.24 (m, 0.3H, 2-H, of minor rotamer). ^13^C NMR (176 MHz, CDCl_3_): *δ*_C_ 17.71, 17.95, 19.50 and 19.54 (CH(CH_3_)_2_), 28.27 and 28.37 (C(CH_3_)_3_), 30.3 and 32.06 (Pyrr 4-CH_2_), 31.0 and 31.3 (NHCH*C*H), 44.1 and 44.6 (Pyrr 5-CH_2_), 50.5 and 51.1 (Pyrr 2-CH_2_), 51.0 (CH_3_), 53.6 and 55.3 (Pyrr C-3), 57.1 and 57.5 (NH*C*HCH), 79.7 and 79.8 (C(CH_3_)_3_), 108.0 and 108.2 (C-3), 109.6 and 109.8 (C-7), 120.0 and 120.1 (C-4), 122.39 and 122.45 (C-5), 123.17 and 123.22 (C-6), 126.87 and 126.98 (C-3a), 130.2 and 130.4 (C-2), 136.30 and 136.35 (C-7a), 155.86 and 155.91 (Boc C=O), 165.06 and 165.09 (COOMe C=O), 171.3 and 171.7 (Pyr-N C=O). ^15^N NMR (71 MHz, CDCl_3_): *δ*_N_ −296.2 and −295.6 (NH), −261.4 (Pyrr N-1), −233.7 and −232.9 (N-1). HRMS (ESI TOF): [M + Na]^+^, found 466.2313. [C_24_H_33_N_3_O_5_ + Na]^+^ requires 466.2312.

**Synthesis of methyl 1-[(3*R*)-1-(*tert*-butoxycarbonyl)pyrrolidin-3-yl]-3-iodo-1*H*-indole-5-carboxylate ((*R*)-10)**

To a solution of **(*R*)-5b** (689 mg, 2 mmol) in DMF (7 mL), KOH (224 mg, 4 mmol) was added, and the resulting suspension was stirred at room temperature for 20 min. A solution of I_2_ (508 mg, 2 mmol) in DMF (2 mL) was added dropwise, and after 1 h, the reaction mixture was poured into ice water (50 mL) and extracted with ethyl acetate (2 × 30 mL). The organic layer was washed with a 10% Na_2_S_2_O_3_ aqueous solution (20 mL) and brine (20 mL), dried over sodium sulfate, filtered, and concentrated under reduced pressure. The residue was purified by flash column chromatography on silica gel using *n*-hexane/acetone (6/1, *v*/*v*). Yield 783 mg (83%), white solid, mp 60–61 °C. IR (ν_max_, cm^−1^): 2975 (-CH_2_-), 1690 (C=O), 1395 (-CH_3_), 1128 (C-O). ^1^H NMR (700 MHz, CDCl_3_): (two rotamers are seen in the spectrum at a ratio of ~1:0.8) *δ*_H_ 1.45–1.52 (m, 9H, C(CH_3_)_3_), 2.18–2.32 (m, 1H, Pyrr 4-CH_A_), 2.35–2.49 (m, 1H, Pyrr 4-CH_B_), 3.45–3.68 (m, 2.55H, Pyrr 5-CH_2_ and Pyrr 2-CH_A_ of major rotamer), 3.75–3.80 (m, 0.45H, Pyrr 2-CH_A_ of minor rotamer), 3.84–4.04 (m, 4H, CH_3_ and Pyrr 2-CH_B_), 4.93–5.03 (m, 1H, Pyrr 3-H), 7.20–7.23 (m, 1H, 2-H), 7.29 (d, *J* = 8.7 Hz, 1H, 7-H), 7.91 (d, *J* = 8.7 Hz, 1H, 6-H), 8.12 (s, 1H, 4-H). ^13^C NMR (176 MHz, CDCl_3_): *δ*_C_ 28.49 (C(CH_3_)_3_), 31.20 and 32.12 (Pyrr 4-CH_2_), 44.04 and 44.28 (Pyrr 5-CH_2_), 50.59 and 51.11 (Pyrr 2-CH_2_), 52.06 (CH_3_), 54.72 and 55.39 (Pyrr C-3), 58.28 and 58.44 (C-3), 80.28 (C(CH_3_)_3_), 109.29 and 109.34 (C-7), 123.03 (C-5), 124.27 (C-6), 124.47 (C-4), 129.70 (C-2), 130.32 and 130.37 (C-3a), 138.53 (C-7a), 154.27 and 154.40 (Boc C=O), 167.63 (C=O). ^15^N NMR (71 MHz, CDCl_3_): *δ*_N_ −294.9 (Pyrr N-1), −231.2 (N-1). HRMS (ESI TOF): [M + Na]^+^, found 493.0600. [C_19_H_23_IN_2_O_4_ + Na]^+^ requires 493.0595.

**General procedure for the Suzuki–Miyaura coupling of 3-iodoindole ((*R*)-10) with arylboronic acids 11a–c to afford compounds (*R*)-12a–c.**

To a solution of 3-iodoindole **(*R*)-10** (94 mg, 0.2 mmol) in a dioxane/water mixture (4:1, *v*/*v*, 1 mL), the given amount of boronic acid (0.26 mmol), Pd(dppf)Cl_2_·DCM (8 mg, 5 mol%), and K_3_PO_4_ (127 mg, 0.6 mmol) were added. The reaction mixture was placed under an Ar atmosphere and stirred at 80 °C under microwave irradiation (100 W) for 15 min. After cooling to room temperature, the mixture was filtered over Celite^®^ and the filter cake was washed with ethyl acetate (2 mL). The filtrate was diluted with ethyl acetate (20 mL), washed with brine (20 mL), dried over sodium sulfate, filtered, and concentrated under reduced pressure. The residue was purified by flash column chromatography on silica gel using an appropriate eluent to afford the corresponding indole derivatives **(*R*)-12a**–**c**.

**Methyl 1-[(3*R*)-1-(*tert*-butoxycarbonyl)pyrrolidin-3-yl]-3-phenyl-1*H*-indole-5-carboxylate ((*R*)-12a)**

The reaction was carried out using a general Suzuki–Miyaura coupling procedure using **(*R*)-10** and phenylboronic acid (**11a**) (32 mg, 0.26 mmol), and the product was purified by column chromatography on silica gel using *n*-hexane/acetone (6/1, *v*/*v*). Yield 51 mg (61%), white solid, mp 54–55 °C. IR (ν_max_, cm^−1^): 2976 (-CH_2_-), 1692 (C=O), 1397 (-CH_3_), 1125 (C-O). ^1^H NMR (700 MHz, CDCl_3_): (two rotamers are present in a ~1:0.8 ratio) *δ*_H_ 1.47–1.52 (m, 9H), 2.29–2.38 (m, 1H), 2.42–2.50 (m, 1H), 3.53–3.66 (m, 2.55H), 3.85–3.90 (m, 0.45H), 3.91–4.01 (m, 4H), 5.06–5.13 (m, 1H), 7.31–7.38 (m, 2H), 7.41–7.50 (m, 3H), 7.62–7.66 (m, 2H), 7.99 (d, *J* = 8.7 Hz, 1H), 8.67 (s, 1H). ^13^C NMR (176 MHz, CDCl_3_): *δ*_C_ 28.61, 31.28 and 32.27, 44.29 and 44.53, 50.64 and 51.22, 52.08, 54.37 and 55.05, 80.27, 109.34 and 109.39, 119.54 and 119.73, 122.62, 122.96 and 123.04, 123.34, 123.78, 126.22 and 126.29, 127.80, 128.88, 129.08, 134.52 and 134.58, 139.26, 154.45 and 154.58, 168.12. ^15^N NMR (71 MHz, CDCl_3_): *δ*_N_ −294.6, −237.7. HRMS (ESI TOF): [M + Na]^+^, found 443.1941. [C_25_H_28_N_2_O_4_ + Na]^+^ requires 443.1941.

**Methyl 1-[(3*R*)-1-(*tert*-butoxycarbonyl)pyrrolidin-3-yl]-3-(4-methylphenyl)-1*H*-indole-5-carboxylate ((*R*)-12b)**

The reaction was carried out using a general Suzuki–Miyaura coupling procedure using **(*R*)-10** and *p*-tolylboronic acid **11b** (35 mg, 0.26 mmol), and the product was purified by column chromatography on silica gel using *n*-hexane/acetone (6/1, *v*/*v*). Yield 60 mg (69%), white solid, mp 64–65 °C. IR (ν_max_, cm^−1^): 2976 (-CH_2_-), 1693 (C=O), 1398 (-CH_3_), 1125 (C-O). ^1^H NMR (700 MHz, CDCl_3_): (two rotamers are present in a ~1:0.8 ratio) *δ*_H_ 1.47–1.52 (m, 9H), 2.28–2.36 (m, 1H), 2.40–2.50 (m, 4H), 3.52–3.66 (m, 1.55H), 3.66–3.72 (m, 1H), 3.84–3.89 (m, 0.45H), 3.90–4.00 (m, 4H), 5.06–5.12 (m, 1H), 7.25–7.33 (m, 3H), 7.41 (d, *J* = 8.7 Hz, 1H), 7.53 (d, *J* = 8.0 Hz, 2H), 7.98 (d, *J* = 8.7 Hz, 1H), 8.65 (s, 1H). ^13^C NMR (176 MHz, CDCl_3_): *δ*_C_ 21.33, 28.62, 31.28 and 32.28, 44.29 and 44.54, 50.63 and 51.22, 52.05, 54.34 and 55.02, 80.25, 109.27 and 109.33, 119.51 and 119.70, 122.48, 122.65 and 122.74, 123.39, 123.70, 126.29 and 126.37, 127.71, 129.77, 131.55 and 131.60, 136.40, 139.22, 154.45 and 154.57, 168.15. ^15^N NMR (71 MHz, CDCl_3_): *δ*_N_ −294.6, −238.2. HRMS (ESI TOF): [M + Na]^+^, found 457.2096. [C_26_H_30_N_2_O_4_ + Na]^+^ requires 457.2098.

**Methyl 1-[(3*R*)-1-(*tert*-butoxycarbonyl)pyrrolidin-3-yl]-3-(4-fluorophenyl)-1*H*-indole-5-carboxylate ((*R*)-12c)**

The reaction was carried out using a general Suzuki–Miyaura coupling procedure using **(*R*)-10** and 4-fluorophenylboronic acid (**11c**) (36 mg, 0.26 mmol), and the product was purified by column chromatography on silica gel using *n*-hexane/acetone (6/1, *v*/*v*). Yield 67 mg (76%), white solid, mp 58–59 °C. IR (ν_max_, cm^−1^): 2977 (-CH_2_-), 1692 (C=O), 1398 (-CH_3_), 1125 (C-O). ^1^H NMR (700 MHz, CDCl_3_): (two rotamers are present in a ~1:0.8 ratio) *δ*_H_ 1.47–1.52 (m, 9H), 2.29–2.37 (m, 1H), 2.42–2.50 (m, 1H), 3.53–3.66 (m, 1.55H), 3.66–3.72 (m, 1H), 3.84–3.90 (m, 0.45H), 3.94 (s, 4H), 5.06–5.13 (m, 1H), 7.13–7.19 (m, 2H), 7.27–7.31 (m, 1H), 7.42 (d, *J* = 8.6 Hz, 1H), 7.56–7.59 (m, 2H), 7.99 (d, *J* = 8.6 Hz, 1H), 8.58 (s, 1H). ^13^C NMR (176 MHz, CDCl_3_): *δ*_C_ 28.61, 31.27 and 32.26, 44.28 and 44.52, 50.63 and 51.22, 52.10, 54.38 and 55.06, 80.30, 109.39 and 109.43, 115.97 (d, *J* = 21.5 Hz), 118.60 and 118.78, 122.69, 122.82 and 122.88, 123.04, 123.85, 126.18 and 126.24, 129.29 (d, *J* = 7.8 Hz), 130.54 and 130.58, 139.16, 154.47 and 154.59, 161.92 (d, *J* = 245.8 Hz), 168.05. ^15^N NMR (71 MHz, CDCl_3_): *δ*_N_ −294.7, −237.8. ^19^F NMR (376 MHz, CDCl_3_): *δ*_F_ −116.1 and −116.0. HRMS (ESI TOF): [M + Na]^+^, found 461.1847. [C_25_H_27_FN_2_O_4_ + Na]^+^ requires 461.1847.

**Methyl 1-[(3*R*)-1-(*tert*-butoxycarbonyl)pyrrolidin-3-yl]-3-(pyridin-4-yl)-1*H*-indole-5-carboxylate ((*R*)-12d)**

A solution of **(*R*)-10** (94 mg, 0.2 mmol) in dioxane/H_2_O (1/1, *v*/*v*, 1 mL) was placed under an Ar atmosphere. Pd(dppf)Cl_2_·DCM (8 mg, 5 mol%) and K_3_PO_4_ (127 mg, 0.6 mmol) were added, and the reaction mixture was heated at 80 °C. After 10 min, 4-pyridinylboronic acid (**11d**) (32 mg, 0.26 mmol) was added and the reaction mixture was stirred at 80 °C for 1 h. After cooling to room temperature, the reaction mixture was filtered over Celite^®^, and the filter cake was washed with ethyl acetate (2 mL). The filtrate was diluted with ethyl acetate (20 mL), washed with brine (20 mL), dried over sodium sulfate, filtered, and concentrated under reduced pressure. The residue was purified by column chromatography on silica gel using *n*-hexane/acetone (6/1, *v*/*v*). Yield 46 mg (55%), yellow solid, mp 52–53 °C. IR (ν_max_, cm^−1^): 2974 (-CH_2_-), 1692 (C=O), 1399 (-CH_3_), 1126 (C-O). ^1^H NMR (700 MHz, CDCl_3_): (two rotamers are present in a ~1:0.8 ratio) *δ*_H_ 1.46–1.53 (m, 9H), 2.30–2.37 (m, 1H), 2.45–2.52 (m, 1H), 3.51–3.74 (m, 2.55H), 3.86–3.92 (m, 0.45H), 3.93–4.01 (m, 4H), 5.06–5.14 (m, 1H), 7.44–7.48 (m, 1H), 7.50 (s, 1H), 7.55–7.58 (m, 2H), 8.00–8.04 (m, 1H), 8.63–8.66 (m, 2H), 8.67–8.70 (m, 1H). ^13^C NMR (176 MHz, CDCl_3_): *δ*_C_ 28.60, 31.23 and 32.21, 44.22 and 44.43, 50.62 and 51.23, 52.23, 54.60 and 55.26, 80.45, 109.78 and 109.84, 116.50 and 116.69, 121.83, 122.96, 123.43, 124.29, 124.53 and 124.62, 125.63 and 125.70, 139.52, 142.38 and 142.44, 150.34, 154.40 and 154.58, 167.83. ^15^N NMR (71 MHz, CDCl_3_): *δ*_N_ −294.7, −234.7, −79.1. HRMS (ESI TOF): [M + H]^+^, found 422.2074. [C_24_H_28_N_3_O_4_ + H]^+^ requires 422.2074.

**Synthesis of methyl 1-[(3*R*)-1-(*tert*-butoxycarbonyl)pyrrolidin-3-yl]-3-(phenylethynyl)-1*H*-indole-5-carboxylate ((*R*)-12e)**

To a solution of **(*R*)-10** (94 mg, 0.2 mmol) in dry DMF (1 mL) under an argon atmosphere, TEA (139 µL, 1 mmol), phenylacetylene (83 µL, 0.75 mmol), Pd(PPh_3_)_2_Cl_2_ (7 mg, 5 mol%), and CuI (7 mg, 10 mol%) were added. The mixture was stirred for 1 h at 65 °C, cooled to room temperature, and poured into water (20 mL). The mixture was extracted with ethyl acetate (2 × 20 mL). The combined organic layers were washed with brine, dried over Na_2_SO_4_, and concentrated. The residue was purified by flash chromatography on silica gel using *n*-hexane/acetone (6/1, *v*/*v*). Yield 82 mg (92%), yellow solid, mp 71–72 °C. IR (ν_max_, cm^−1^): 2976 (-CH_2_-), 2210 (C≡C), 1693 (C=O), 1393 (-CH_3_), 1165 (C-O). ^1^H NMR (700 MHz, CDCl_3_): (two rotamers are present in a ~1:0.8 ratio) *δ*_H_ 1.48–1.53 (m, 9H), 2.24–2.31 (m, 1H), 2.40–2.48 (m, 1H), 3.50–3.71 (m, 2.55H), 3.78–3.85 (m, 0.45H), 3.88–3.99 (m, 4H), 5.03–5.09 (m, 1H), 7.31–7.42 (m, 4H), 7.47–7.49 (m, 1H), 7.56–7.60 (m, 2H), 8.00 (d, *J* = 8.6 Hz, 1H), 8.55 (s, 1H). ^13^C NMR (176 MHz, CDCl_3_): *δ*_C_ 28.61, 31.27 and 32.16, 44.18 and 44.39, 50.70 and 51.22, 52.17, 54.66 and 55.35, 80.39, 81.80 and 81.89, 92.16 and 92.25, 100.07 and 100.24, 109.48 and 109.55, 123.13, 123.45, 123.83, 124.47, 128.04, 128.49, 128.89 and 128.96, 129.06, 131.57, 138.00, 154.38 and 154.51, 167.92. ^15^N NMR (71 MHz, CDCl_3_): *δ*_N_ −294.8, −235.6. HRMS (ESI TOF): [M + Na]^+^, found 467.1941. [C_27_H_28_N_2_O_4_ + Na]^+^ requires 467.1941.

**General procedure for the *N*-alkylation of 1*H*-pyrazolecarboxylates 13a,b with pyrrolidine mesylates (*S*)-4 or (*R*)-4 to afford compounds (*R*)-14a,b and (*R*)-15b or compounds (*S*)-14a,b and (*S*)-15b, respectively.**

To a solution of the appropriate ethyl 1*H*-pyrazolecarboxylate (420 mg, 3 mmol) in dimethylformamide (8 mL), (*S*)- or (*R*)-1-Boc-3-methanesulfonyloxypyrrolidine (**(*S*)-4** or **(*R*)-4**) (874 mg, 4.2 mmol) and Cs_2_CO_3_ (1467 mg, 4.5 mmol) were added. The reaction mixture was heated at 80 °C for 4 h. After cooling to room temperature, the mixture was diluted with water (20 mL) and extracted with ethyl acetate (3 × 20 mL). The combined organic layers were washed with brine (20 mL), dried over sodium sulfate, filtered, and concentrated under reduced pressure. The residue was purified by flash column chromatography using an appropriate eluent.

**Ethyl 1-[(3*R*)-1-(*tert*-butoxycarbonyl)pyrrolidin-3-yl]-1*H*-pyrazole-4-carboxylate ((*R*)-14a)**

The reaction was carried out using a general *N*-alkylation procedure using 1*H*-pyrazole-4-carboxylate **13a** and pyrrolidine mesylate **(*S*)-4**, and the product was purified by column chromatography on silica gel using *n*-hexane/acetone (4/1, *v*/*v*). Yield 872 mg (93%), colorless oil. ${\left[\alpha \right]}_{D}^{20}$ = −23.7° (c 2.41, MeOH). IR (ν_max_, cm^−1^): 2977 (-CH_2_-), 1690 (C=O), 1401 (-CH_3_), 1165 (C-O). ^1^H NMR (700 MHz, CDCl_3_): (two rotamers are present in the spectrum, ratio ~1:0.8*) δ*_H_ 1.34 (t, *J* = 7.2 Hz, 3H, CH_2_CH_3_), 1.48 (s, 9H, C(CH_3_)_3_), 2.36–2.41 (m, 2H, Pyrr 4-CH_2_), 3.51–3.59 (m, 1.45H, Pyrr 5-CH_B_ of minor rotamer and Pyrr 5-CH_A_), 3.60–3.65 (m, 0.55H, Pyrr 5-CH_B_ of major rotamer), 3.68–3.73 (m, 0.55H, Pyrr 2-CH_A_ of major rotamer), 3.76–3.81 (m, 0.45H, Pyrr 2-CH_A_ of minor rotamer), 3.83–3.88 (m, 1H, Pyrr 2-CH_B_), 4.29 (q, *J* = 7.1 Hz, 2H, CH_2_CH_3_), 4.89–4.91 (m, 1H, Pyrr 3-H), 7.93 (s, 2H, 3-H and 5-H). ^13^C NMR (176 MHz, CDCl_3_): *δ*_C_ 14.39 (CH_2_CH_3_), 28.46 (C(CH_3_)_3_), 31.00 and 31.94 (Pyrr 4-CH_2_), 43.94 and 44.29 (Pyrr 5-CH_2_), 50.80 and 51.12 (Pyrr 2-CH_2_), 60.26 (CH_2_CH_3_), 60.35 and 61.02 (Pyrr C-3), 79.96 and 80.01 (C(CH_3_)_3_), 115.36 (C-4), 130.99 and 141.24 (C-3 and C-5), 154.24 and 154.32 (Boc C=O), 162.88 (C=O). ^15^N NMR (71 MHz, CDCl_3_): *δ*_N_ −294.6 (Pyrr N-1), −161.4 (N-1), −76.3 (N-2). HRMS (ESI TOF): [M + Na]^+^, found 332.1579. [C_15_H_23_N_3_O_4_ + Na]^+^ requires 332.1581.

**Ethyl 1-[(3*S*)-1-(*tert*-butoxycarbonyl)pyrrolidin-3-yl]-1*H*-pyrazole-4-carboxylate ((*S*)-14a)**

The reaction was carried out using a general alkylation procedure with 1*H*-pyrazole-4-carboxylate **13a** and (*R*)-1-Boc-3-methanesulfonyloxypyrrolidine **(*R*)-4**. The reaction was run for 4 h, and the product was purified by column chromatography on silica gel using *n*-hexane/acetone (4/1, *v*/*v*). Yield: 826 mg (89%), colorless oil. ${\left[\alpha \right]}_{D}^{20}$ = 23.9° (c 1.09, MeOH). IR (ν_max_, cm^−1^): 2978 (-CH_2_-), 1690 (C=O), 1401 (-CH_3_), 1165 (C-O). ^1^H NMR (700 MHz, CDCl_3_): (two rotamers are present in the spectrum, ratio ~1:0.8) *δ*_H_ 1.33 (t, *J* = 7.1 Hz, 3H, CH_2_CH_3_), 1.46 (s, 9H, C(CH_3_)_3_), 2.33–2.41 (m, 2H, Pyrr 4-CH_2_), 3.48–3.57 (m, 1.45H, Pyrr 5-CH_B_ of minor rotamer and Pyrr 5-CH_A_), 3.58–3.66 (m, 0.55H, Pyrr 5-CH_B_ of major rotamer), 3.66–3.73 (m, 0.55H, Pyrr 2-CH_A_ of major rotamer), 3.75–3.80 (m, 0.45H, Pyrr 2-CH_A_ of minor rotamer), 3.80–3.88 (m, 1H, Pyrr 2-CH_B_), 4.28 (q, *J* = 7.1 Hz, 2H, CH_2_CH_3_), 4.81–4.94 (m, 1H, Pyrr 3-H), 7.87–7.94 (m, 2H, 3-H and 5-H). ^13^C NMR (176 MHz, CDCl_3_): *δ*_C_ 14.39 (CH_2_CH_3_), 28.46 (C(CH_3_)_3_), 31.00 and 31.94 (Pyrr 4-CH_2_), 43.93 and 44.28 (Pyrr 5-CH_2_), 50.80 and 51.11 (Pyrr 2-CH_2_), 60.28 (CH_2_CH_3_), 60.35 and 61.02 (Pyrr C-3), 80.00 and 80.04 (C(CH_3_)_3_), 115.37 (C-4), 130.97 and 141.25 (C-3 and C-5), 154.25 and 154.33 (Boc C=O), 162.89 (C=O). ^15^N NMR (71 MHz, CDCl_3_): *δ*_N_ −294.6 (Pyrr N-1), −161.4 (N-1), −76.3 (N-2). HRMS (ESI TOF): [M + Na]^+^, found 332.1581. [C_15_H_23_N_3_O_4_ + Na]^+^ requires 332.1581.

**Ethyl 1-[(3*R*)-1-(*tert*-butoxycarbonyl)pyrrolidin-3-yl]-1*H*-pyrazole-3-carboxylate ((*R*)-14b)**

The reaction was carried out using a general alkylation procedure using 1*H*-pyrazole-3-carboxylate **13b** and pyrrolidine mesylate **(*S*)-4**, and the product was purified by column chromatography on silica gel using *n*-hexane/acetone (4/1, *v*/*v*). Yield 343 mg (37%), white solid, mp 95–96 °C. ${\left[\alpha \right]}_{D}^{20}$ = −26.6° (c 1.60, MeOH). IR (ν_max_, cm^−1^): 2982 (-CH_2_-), 1683 (C=O), 1409 (-CH_2_CH_3_), 1170 (C-O). ^1^H NMR (700 MHz, CDCl_3_): (two rotamers are seen in the spectrum, ratio ~1:0.8) *δ*_H_ 1.40 (t, *J* = 7.1 Hz, 3H, CH_2_CH_3_), 1.45–1.48 (m, 9H, C(CH_3_)_3_), 2.36–2.48 (m, 2H, Pyrr 4-CH_2_), 3.49–3.58 (m, 1.45H, Pyrr 5-CH_A_ and Pyrr 5-CH_B_ of minor rotamer), 3.59–3.65 (m, 0.55H, Pyrr 5-CH_B_ of major rotamer), 3.70–3.76 (m, 0.55H, Pyrr 2-CH_B_ of major rotamer), 3.79–3.84 (m, 0.45H, Pyrr 2-CH_B_ of minor rotamer), 3.84–3.89 (m, 1H, Pyrr 2-CH_A_), 4.41 (q, *J* = 7.1 Hz, 2H, CH_2_CH_3_), 5.01 (p, *J* = 6.0 Hz, 1H, Pyrr 3-H), 6.83 (d, *J* = 2.7 Hz, 1H, 4-H), 7.43–7.47 (m, 1H, 5-H). ^13^C NMR (176 MHz, CDCl_3_): *δ*_C_ 14.40 (CH_2_CH_3_), 28.45 (C(CH_3_)_3_), 31.14 and 32.21 (Pyrr 4-CH_2_), 43.91 and 44.23 (Pyrr 5-CH_2_), 50.74 and 51.20 (Pyrr 2-CH_2_), 60.87 and 61.53 (Pyrr C-3), 61.02 (CH_2_CH_3_), 80.01 (C(CH_3_)_3_), 109.22 (C-4), 128.55 and 128.64 (C-5), 143.90 (C-3), 154.26 and 154.31 (Boc C=O), 162.23 (C=O). ^15^N NMR (71 MHz, CDCl_3_): *δ*_N_ −294.8 (Pyrr N-1), −159.9 (N-1), −70.1 (N-2). HRMS (ESI TOF): [M + Na]^+^, found 332.1580. [C_15_H_23_N_3_O_4_ + Na]^+^ requires 332.1581.

**Ethyl 1-[(3*S*)-1-(*tert*-butoxycarbonyl)pyrrolidin-3-yl]-1*H*-pyrazole-3-carboxylate ((*S*)-14b)**

The reaction was carried out using a general alkylation procedure using 1*H*-pyrazole-3-carboxylate **13b** and pyrrolidine mesylate **(*R*)-4**, and the product was purified by column chromatography on silica gel using *n*-hexane/acetone (4/1, *v*/*v*). Yield 334 mg (37%), white solid, mp 95–96 °C. ${\left[\alpha \right]}_{D}^{20}$ = 26.8° (c 0.82, MeOH). IR (ν_max_, cm^−1^): 2982 (-CH_2_-), 1683 (C=O), 1409 (-CH_2_CH_3_), 1171 (C-O). ^1^H NMR (700 MHz, CDCl_3_): (two rotamers are seen in the spectrum, ratio ~1:0.8) *δ*_H_ 1.40 (t, *J* = 7.1 Hz, 3H, CH_2_CH_3_), 1.41–1.51 (m, 9H, C(CH_3_)_3_), 2.37–2.47 (m, 2H, Pyrr 4-CH_2_), 3.49–3.58 (m, 1.45H, Pyrr 5-CH_A_ and Pyrr 5-CH_B_ of minor rotamer), 3.59–3.65 (m, 0.55H, Pyrr 5-CH_B_ of major rotamer), 3.70–3.75 (m, 0.55H, Pyrr 2-CH_B_ of major rotamer), 3.79–3.83 (m, 0.45H, Pyrr 2-CH_B_ of minor rotamer), 3.84–3.89 (m, 1H, Pyrr 2-CH_A_), 4.41 (q, *J* = 7.1 Hz, 2H, CH_2_CH_3_), 5.01 (p, *J* = 6.0 Hz, 1H, Pyrr 3-H), 6.83 (d, *J* = 2.4 Hz, 1H, 4-H), 7.43–7.55 (m, 1H, 5-H). ^13^C NMR (176 MHz, CDCl_3_): *δ*_C_ 14.40 (CH_2_CH_3_), 28.46 (C(CH_3_)_3_), 31.14 and 32.22 (Pyrr 4-CH_2_), 43.91 and 44.23 (Pyrr 5-CH_2_), 50.75 and 51.21 (Pyrr 2-CH_2_), 60.87 and 61.54 (Pyrr C-3), 61.03 (CH_2_CH_3_), 80.03 (C(CH_3_)_3_), 109.23 (C-4), 128.54 and 128.62 (C-5), 143.91 (C-3), 154.25 and 154.30 (Boc C=O), 162.23 (C=O). ^15^N NMR (71 MHz, CDCl_3_): *δ*_N_ −294.9 (Pyrr N-1), −160.0 (N-1), −70.1 (N-2). HRMS (ESI TOF): [M + Na]^+^, found 332.1578. [C_15_H_23_N_3_O_4_ + Na]^+^ requires 332.1581.

**Ethyl 1-[(3*R*)-1-(*tert*-butoxycarbonyl)pyrrolidin-3-yl]-1*H*-pyrazole-5-carboxylate ((*R*)-15b)**

The reaction was carried out using a general alkylation procedure using 1*H*-pyrazole-5-carboxylate **13b** and (*S*)-1-Boc-3-methanesulfonyloxypyrrolidine **(*S*)-4,** and the product was purified by column chromatography on silica gel using *n*-hexane/acetone (6/1, *v*/*v*). Yield: 446 mg (48%), colorless oil. ${\left[\alpha \right]}_{D}^{20}$ = 14.0° (c 1.09, MeOH). IR (ν_max_, cm^−1^): 2979 (-CH_2_-), 1690 (C=O), 1400 (-CH_2_CH_3_), 1165 (C-O). ^1^H NMR (700 MHz, CDCl_3_): *δ*_H_ 1.36–1.41 (m, 3H, CH_2_CH_3_), 1.44–1.48 (m, 9H, C(CH_3_)_3_), 2.28–2.36 (m, 1H, Pyrr 4-CH_A_), 2.43–2.54 (m, 1H, Pyrr 4-CH_B_), 3.47–3.55 (m, 1H, Pyrr 5-CH_A_), 3.63–3.74 (m, 2H, Pyrr 5-CH_B_ and Pyrr 2-CH_A_), 3.79–3.87 (m, 1H, Pyrr 2-CH_B_), 4.31–4.38 (m, 2H, CH_2_CH_3_), 5.79–5.88 (m, 1H, Pyrr 3-H), 6.86 (d, *J* = 2.2 Hz, 1H, 4-H), 7.48–7.52 (m, 1H, 3-H). ^13^C NMR (176 MHz, CDCl_3_): *δ*_C_ 14.23 (CH_2_CH_3_), 28.50 (C(CH_3_)_3_), 31.00 and 31.55 (Pyrr 4-CH_2_), 44.55 and 45.12 (Pyrr 5-CH_2_), 51.27 and 51.56 (Pyrr 2-CH_2_), 58.35 and 58.84 (Pyrr C-3), 61.10 and 61.12 (CH_2_CH_3_), 79.38 and 79.45 (C(CH_3_)_3_), 111.75 and 111.82 (C-4), 132.19 (C-5), 138.03 and 138.19 (C-3), 154.42 (Boc C=O), 159.87 and 159.90 (C=O). ^15^N NMR (71 MHz, CDCl_3_): *δ*_N_ −293.4 (Pyrr N-1), −162.1 (N-1), −66.3 (N-2). HRMS (ESI TOF): [M + Na]^+^, found 332.1581. [C_15_H_23_N_3_O_4_ + Na]^+^ requires 332.1581.

**Ethyl 1-[(3*S*)-1-(*tert*-butoxycarbonyl)pyrrolidin-3-yl]-1*H*-pyrazole-5-carboxylate ((*S*)-15b)**

The reaction was carried out using a general alkylation procedure using 1*H*-pyrazole-3-carboxylate **13b** and pyrrolidine mesylate **(*R*)-4**, and the product was purified by column chromatography on silica gel using n-hexane/acetone (6/1, *v*/*v*). Yield 445 mg (48%), colorless oil. ${\left[\alpha \right]}_{D}^{20}$ = −14.1° (c 1.68, MeOH). IR (ν_max_, cm^−1^): 2978 (-CH_2_-), 1694 (C=O), 1394 (-CH_2_CH_3_), 1162 (C-O). ^1^H NMR (700 MHz, CDCl_3_): *δ*_H_ 1.37–1.41 (m, 3H, CH_2_CH_3_), 1.44–1.48 (m, 9H, C(CH_3_)_3_), 2.29–2.35 (m, 1H, Pyrr 4-CH_A_), 2.43–2.54 (m, 1H, Pyrr 4-CH_B_), 3.47–3.55 (m, 1H, Pyrr 5-CH_A_), 3.63–3.74 (m, 2H, Pyrr 5-CH_B_ and Pyrr 2-CH_A_), 3.79–3.87 (m, 1H, Pyrr 2-CH_B_), 4.32–4.38 (m, 2H, CH_2_CH_3_), 5.80–5.87 (m, 1H, Pyrr 3-H), 6.86 (d, *J* = 2.2 Hz, 1H, 4-H), 7.48–7.52 (m, 1H, 3-H). ^13^C NMR (176 MHz, CDCl_3_): *δ*_C_ 14.23 (CH_2_CH_3_), 28.51 (C(CH_3_)_3_), 31.00 and 31.55 (Pyrr 4-CH_2_), 44.55 and 45.12 (Pyrr 5-CH_2_), 51.27 and 51.56 (Pyrr 2-CH_2_), 58.35 and 58.84 (Pyrr C-3), 61.10 and 61.12 (CH_2_CH_3_), 79.38 and 79.46 (C(CH_3_)_3_), 111.75 and 111.82 (C-4), 132.19 (C-5), 138.03 and 138.20 (C-3), 154.43 (Boc C=O), 159.87 and 159.90 (C=O). ^15^N NMR (71 MHz, CDCl_3_): *δ*_N_ −293.0 (Pyrr N-1), −162.2 (N-1), −66.2 (N-2). HRMS (ESI TOF): [M + Na]^+^, found 332.1577. [C_15_H_23_N_3_O_4_ + Na]^+^ requires 332.1581.

**Synthesis of ethyl 4-bromo-1-[(3*R*)-1-(tert-butoxycarbonyl)pyrrolidin-3-yl]-1*H*-pyrazole-5-carboxylate ((*R*)-16)**

To a solution of compound **(*R*)-15b** (885 mg, 3 mmol) in acetonitrile (12 mL), *N*-bromosuccinimide (1068 mg, 6 mmol) was added, and the mixture was stirred at rt for 4 h. The mixture was poured into water (20 mL), and the solution was extracted with ethyl acetate (2 × 20 mL). The combined organic layers were washed with brine (20 mL), dried over sodium sulfate, filtered, and concentrated under reduced pressure. The residue was purified by flash column chromatography on silica gel using *n*-hexane/acetone (8/1, *v*/*v*). Yield 559 mg (48%), colorless oil. IR (ν_max_, cm^−1^): 2978 (-CH_2_-), 1690 (C=O), 1400 (-CH_2_CH_3_), 1165 (C-O). ^1^H NMR (700 MHz, CDCl_3_): *δ*_H_ 1.40–1.46 (m, 12H, C(CH_3_)_3_ and CH_2_CH_3_), 2.25–2.33 (m, 1H, Pyrr 4-CH_A_), 2.42–2.49 (m, 1H, Pyrr 4-CH_B_), 3.45–3.52 (m, 1H, Pyrr 5-CH_A_), 3.60–3.71 (m, 2H, Pyrr 5-CH_B_ and Pyrr 2-CH_A_), 3.77–3.82 (m, 1H, Pyrr 2-CH_B_), 4.41 (q, *J* = 7.0 Hz, 2H, CH_2_CH_3_), 5.68–5.77 (m, 1H, Pyrr 3-H), 7.52 (s, 1H, 3-H). ^13^C NMR (176 MHz, CDCl_3_): *δ*_C_ 14.21 (CH_2_CH_3_), 28.47 (C(CH_3_)_3_), 30.85 and 31.42 (Pyrr 4-CH_2_), 44.53 and 45.02 (Pyrr 5-CH_2_), 51.37 and 51.52 (Pyrr 2-CH_2_), 59.70 and 60.19 (Pyrr C-3), 61.74 (CH_2_CH_3_), 79.54 (C(CH_3_)_3_), 99.78 (C-4), 130.18 (C-5), 140.35 (C-3), 154.37 (Boc C=O), 159.17 (C=O). ^15^N NMR (71 MHz, CDCl_3_): *δ*_N_ −293.3 (Pyrr N-1), −160.7 (N-1), −68.2 (N-2). HRMS (ESI TOF): [M + Na]^+^, found 410.0686. [C_15_H_22_^79^BrN_3_O_4_ + Na]^+^ requires 410.0686; [M + Na]^+^, found 412.0668. [C_15_H_22_^81^BrN_3_O_4_ + Na]^+^ requires 412.0668.

**General procedure for the Suzuki–Miyaura coupling of bromopyrazole (*R*)-16 with boronic acids to afford compounds (*R*)-17a–f.**

To a solution of bromopyrazole **(*R*)-16** (78 mg, 0.2 mmol) in a dioxane/water mixture (4:1, *v*/*v*, 1 mL), the appropriate boronic acid (0.26 mmol), Pd(dppf)Cl_2_·DCM (8 mg, 5 mol%), and K_3_PO_4_ (127 mg, 0.6 mmol) were added. The reaction mixture was placed under an Ar atmosphere and stirred at 80 °C under microwave irradiation (100 W) for 15 min. After cooling to room temperature, the mixture was filtered over Celite^®^, and the filter cake was washed with ethyl acetate (2 mL). The filtrate was diluted with ethyl acetate (20 mL), washed with brine (20 mL), dried over sodium sulfate, filtered, and concentrated under reduced pressure. The residue was purified by flash column chromatography on silica gel using an appropriate eluent.

**Ethyl 1-[(3*R*)-1-(*tert*-butoxycarbonyl)pyrrolidin-3-yl]-4-phenyl-1*H*-pyrazole-5-carboxylate ((*R*)-17a)**

The reaction was carried out using a general Suzuki–Miyaura coupling procedure using bromopyrazole (**(*R*)-16**) and phenylboronic acid **11a** (32 mg, 0.26 mmol), and the product was purified by column chromatography on silica gel using *n*-hexane/acetone (8/1, *v*/*v*). Yield: 56 mg (72%), yellow oil. IR (ν_max_, cm^−1^): 2977 (-CH_2_-), 1694 (C=O), 1400 (-CH_2_CH_3_), 1166 (C-O). ^1^H NMR (700 MHz, CDCl_3_): *δ*_H_ 1.12 (t, *J* = 7.2 Hz, 3H), 1.47 (s, 9H), 2.31–2.38 (m, 1H), 2.49–2.60 (m, 1H), 3.49–3.55 (m, 1H), 3.69–3.78 (m, 2H), 3.83–3.88 (m, 1H), 4.18–4.24 (m, 2H), 5.68–5.73 (m, 1H), 7.30–7.38 (m, 5H), 7.50–7.55 (m, 1H). ^13^C NMR (176 MHz, CDCl_3_): *δ*_C_ 13.76, 28.62, 31.18 and 31.70, 44.69 and 45.29, 51.50 and 51.85, 58.93 and 59.39, 61.31, 79.57, 127.47, 127.75, 127.94, 129.08 and 129.14, 129.56, 132.62 and 132.68, 138.84 and 139.00, 154.54, 160.66. ^15^N NMR (71 MHz, CDCl_3_): *δ*_N_ −292.9, −162.5, −71.1. HRMS (ESI TOF): [M + Na]^+^, found 408.1888. [C_21_H_27_N_3_O_4_ + Na]^+^ requires 408.1894.

**Ethyl 1-[(3*R*)-1-(*tert*-butoxycarbonyl)pyrrolidin-3-yl]-4-(4-methylphenyl)-1*H*-pyrazole-5-carboxylate ((*R*)-17b)**

The reaction was carried out using a general Suzuki–Miyaura coupling procedure using **(*R*)-16** and *p*-tolylboronic acid **11b** (35 mg, 0.26 mmol), and the product was purified by column chromatography on silica gel using *n*-hexane/ethyl acetate (8/1, *v*/*v*). Yield: 54 mg (67%), yellow oil. IR (ν_max_, cm^−1^): 2978 (-CH_2_-), 1695 (C=O), 1401 (-CH_2_CH_3_), 1166 (C-O). ^1^H NMR (700 MHz, CDCl_3_): *δ*_H_ 1.16 (t, *J* = 7.1 Hz, 3H), 1.46 (s, 9H), 2.30–2.41 (m, 4H), 2.49–2.59 (m, 1H), 3.48–3.55 (m, 1H), 3.69–3.77 (m, 2H), 3.82–3.88 (m, 1H), 4.19–4.26 (m, 2H), 5.66–5.71 (m, 1H), 7.17 (d, *J* = 8.2 Hz, 2H), 7.25 (d, *J* = 8.2 Hz, 2H), 7.51 (s, 1H). ^13^C NMR (176 MHz, CDCl_3_): *δ*_C_ 13.88, 21.34, 28.64, 31.19 and 31.71, 44.70 and 45.30, 51.52 and 51.85, 58.95 and 59.39, 61.31, 79.56, 127.77 and 127.86, 128.69, 129.01, 129.42, 129.60, 137.23, 138.93 and 139.04, 154.57, 160.76. ^15^N NMR (71 MHz, CDCl_3_): *δ*_N_ −293.1, −163.5, −71.1. HRMS (ESI TOF): [M + Na]^+^, found 422.2052. [C_22_H_29_N_3_O_4_ + Na]^+^ requires 422.2050.

**Ethyl 1-[(3*R*)-1-(*tert*-butoxycarbonyl)pyrrolidin-3-yl]-4-(4-fluorophenyl)-1*H*-pyrazole-5-carboxylate ((*R*)-17c)**

The reaction was carried out using a general Suzuki–Miyaura coupling procedure using **(*R*)-16** and 4-fluorophenylboronic acid **11c** (36 mg, 0.26 mmol), and the product was purified by column chromatography on silica gel using *n*-hexane/ethyl acetate (8/1, *v*/*v*). Yield: 61 mg (76%), yellow oil. IR (ν_max_, cm^−1^): 2978 (-CH_2_-), 1692 (C=O), 1400 (-CH_2_CH_3_), 1161 (C-O). ^1^H NMR (700 MHz, CDCl_3_): *δ*_H_ 1.14 (t, *J* = 7.2 Hz, 3H), 1.46 (s, 9H), 2.29–2.39 (m, 1H), 2.48–2.61 (m, 1H), 3.48–3.57 (m, 1H), 3.67–3.80 (m, 2H), 3.82–3.91 (m, 1H), 4.17–4.25 (m, 2H), 5.67–5.76 (m, 1H), 7.02–7.08 (m, 2H), 7.30–7.34 (m, 2H), 7.46–7.53 (m, 1H). ^13^C NMR (176 MHz, CDCl_3_): *δ*_C_ 13.85, 28.64, 31.20 and 31.67, 44.70 and 45.31, 51.53 and 51.92, 59.08 and 59.52, 61.34 and 61.38, 79.58 and 79.63, 114.90 (d, *J* = 21.5 Hz), 126.83 and 126.98, 128.66 and 128.72, 129.08 and 129.14, 131.27 (d, *J* = 8.0 Hz), 138.82 and 138.98, 154.57, 160.43 and 160.48, 162.42 (d, *J* = 246.6 Hz). ^15^N NMR (71 MHz, CDCl_3_): *δ*_N_ −293.8, −162.6, −70.7. ^19^F NMR (376 MHz, CDCl_3_): *δ*_F_ −115.1 and −114.9. HRMS (ESI TOF): [M + Na]^+^, found 426.1800. [C_21_H_26_FN_3_O_4_ + Na]^+^ requires 426.1800.

**Ethyl 1-[(3*R*)-1-(*tert*-butoxycarbonyl)pyrrolidin-3-yl]-4-(pyridin-4-yl)-1*H*-pyrazole-5-carboxylate ((*R*)-17d)**

To a solution of bromopyrazole (**(*R*)-16**) (78 mg, 0.2 mmol) in dioxane/H_2_O (1/1, *v*/*v*, 1 mL) under an Ar atmosphere, Pd(dppf)Cl_2_·DCM (8 mg, 5 mol%) and K_3_PO_4_ (127 mg, 0.6 mmol) were added. The reaction mixture was heated at 80 °C. After 10 min, 4-pyridinylboronic acid **11d** (32 mg, 0.26 mmol) was added, and the mixture was stirred at 80 °C for 1 h. After cooling to room temperature, the mixture was filtered over Celite^®^ and the filter cake was washed with ethyl acetate (2 mL). The filtrate was diluted with ethyl acetate (20 mL), washed with brine (20 mL), dried over sodium sulfate, filtered, and concentrated under reduced pressure. The residue was purified by column chromatography on silica gel using *n*-hexane/acetone (4/1, *v*/*v*). Yield: 43 mg (56%), yellow oil. IR (ν_max_, cm^−1^): 2977 (-CH_2_-), 1690 (C=O), 1400 (-CH_2_CH_3_), 1165 (C-O). ^1^H NMR (700 MHz, CDCl_3_): *δ*_H_ 1.13–1.19 (m, 3H), 1.43–1.49 (m, 9H), 2.31–2.40 (m, 1H), 2.47–2.60 (m, 1H), 3.49–3.58 (m, 1H), 3.66–3.79 (m, 2H), 3.82–3.90 (m, 1H), 4.17–4.32 (m, 2H), 5.67–5.75 (m, 1H), 7.28–7.31 (m, 2H), 7.52–7.60 (m, 1H), 8.57–8.62 (m, 2H). ^13^C NMR (176 MHz, CDCl_3_): *δ*_C_ 13.80, 28.62, 31.24 and 31.70, 44.68 and 45.28, 51.55 and 51.95, 59.28 and 59.73, 61.72 and 61.78, 79.65 and 79.70, 124.34 and 124.40, 124.86 and 125.00, 129.46 and 129.55, 138.53 and 138.71, 140.91 and 141.04, 149.35 and 149.41, 154.49 and 154.58, 160.03 and 160.10. ^15^N NMR (71 MHz, CDCl_3_): *δ*_N_ −293.2, −160.4, −75.6, −69.5. HRMS (ESI TOF): [M + H]^+^, found 387.2027. [C_20_H_27_N_4_O_4_ + H]^+^ requires 387.2027.

**Ethyl 1-[(3*R*)-1-(*tert*-butoxycarbonyl)pyrrolidin-3-yl]-4-[4-(trifluoromethyl)phenyl]-1*H*-pyrazole-5-carboxylate ((*R*)-17e)**

The reaction was carried out using a general Suzuki–Miyaura coupling procedure using **(*R*)-16** and 4-(trifluoromethyl)phenylboronic acid **11e** (49 mg, 0.26 mmol), and the product was purified by column chromatography on silica gel using *n*-hexane/acetone (10/1, *v*/*v*). Yield: 73 mg (80%), yellow oil. IR (ν_max_, cm^−1^): 2980 (-CH_2_-), 1693 (C=O), 1402 (-CH_2_CH_3_), 1323 (-CF_3_), 1163 (C-O). ^1^H NMR (700 MHz, CDCl_3_): *δ*_H_ 1.12 (t, *J* = 7.1 Hz, 3H), 1.47 (s, 9H), 2.32–2.43 (m, 1H), 2.49–2.61 (m, 1H), 3.50–3.57 (m, 1H), 3.67–3.82 (m, 2H), 3.84–3.91 (m, 1H), 4.17–4.27 (m, 2H), 5.67–5.77 (m, 1H), 7.48 (d, *J* = 7.9 Hz, 2H), 7.51–7.57 (m, 1H), 7.62 (d, *J* = 8.1 Hz, 2H). ^13^C NMR (176 MHz, CDCl_3_): *δ*_C_ 13.79, 28.64, 31.26 and 31.70, 44.73 and 45.32, 51.59 and 51.95, 59.21 and 59.63, 61.56, 79.79, 115.63, 124.33 (q, *J* = 272.1 Hz), 124.90 (q, *J* = 3.7 Hz), 127.10 (q, *J* = 3.8 Hz), 129.37, 129.94, 136.52, 138.77 and 138.88, 154.64, 160.24. ^15^N NMR (71 MHz, CDCl_3_): *δ*_N_ −293.4, −161.4, −62.5. ^19^F NMR (376 MHz, CDCl_3_): *δ*_F_ −62.5. HRMS (ESI TOF): [M + Na]^+^, found 476.1766. [C_22_H_26_F_3_N_3_O_4_ + Na]^+^ requires 476.1768.

**Ethyl 1-[(3*R*)-1-(*tert*-butoxycarbonyl)pyrrolidin-3-yl]-4-(thiophen-3-yl)-1*H*-pyrazole-5-carboxylate ((*R*)-17f)**

The reaction was carried out using a general Suzuki–Miyaura coupling procedure using **(*R*)-16** and 3-thienylboronic acid **11f** (33 mg, 0.26 mmol), and the product was purified by column chromatography on silica gel using *n*-hexane/acetone (10/1, *v*/*v*). Yield: 49 mg (63%), yellow oil. IR (ν_max_, cm^−1^): 2977 (-CH_2_-), 1691 (C=O), 1400 (-CH_2_CH_3_), 1165 (C-O). ^1^H NMR (700 MHz, CDCl_3_): *δ*_H_ 1.24 (t, *J* = 7.2 Hz, 3H), 1.46 (s, 9H), 2.29–2.38 (m, 1H), 2.47–2.60 (m, 1H), 3.47–3.55 (m, 1H), 3.67–3.77 (m, 2H), 3.81–3.88 (m, 1H), 4.24–4.33 (m, 2H), 5.65–5.71 (m, 1H), 7.15–7.19 (m, 1H), 7.29–7.32 (m, 1H), 7.33–7.36 (m, 1H), 7.56 (s, 1H). ^13^C NMR (176 MHz, CDCl_3_): *δ*_C_ 14.00, 28.62, 31.20 and 31.68, 44.71 and 45.27, 51.54 and 51.86, 59.12 and 59.55, 61.45, 79.57, 122.50, 123.46, 124.72, 128.96, 129.21, 132.37, 139.02, 154.54, 160.52. ^15^N NMR (71 MHz, CDCl_3_): *δ*_N_ −293.2, −163.3, −71.0. HRMS (ESI TOF): [M + Na]^+^, found 414.1458. [C_19_H_25_N_3_O_4_S + Na]^+^ requires 414.1458.

**General procedure for the alkylation of 1*H*-indazolecarboxylates 18a–c with pyrrolidine mesylates (*S*)-4 or (*R*)-4 to afford compounds (*R*)-19a–c and (*R*)-20a–c or compounds (*S*)-19a–c and (*S*)-20a–c, respectively.**

To a solution of the appropriate 1*H*-indazolecarboxylate **18a**–**c** (529 mg, 3 mmol) in dimethylformamide (8 mL), pyrrolidine mesylate **(*S*)-4** or **(*R*)-4** (1114 mg, 4.2 mmol) and Cs_2_CO_3_ (1467 mg, 4.5 mmol) were added. The reaction mixture was heated at 80 °C for 4 h. After cooling to room temperature, the mixture was diluted with water (20 mL) and extracted with ethyl acetate (3 × 20 mL). The combined organic layers were washed with brine (20 mL), dried over sodium sulfate, filtered, and concentrated under reduced pressure. The residue was purified by flash column chromatography using an appropriate eluent.

**Methyl 1-[(3*R*)-1-(*tert*-butoxycarbonyl)pyrrolidin-3-yl]-1*H*-indazole-3-carboxylate ((*R*)-19a)**

The reaction was carried out using a general alkylation procedure with 1*H*-indazole-3-carboxylate **18a** and pyrrolidine mesylate **(*S*)-4**, and the product was purified by column chromatography on silica gel using *n*-hexane/ethyl acetate (2/1, *v*/*v*). Yield 446 mg (43%), white solid, mp 118–119 °C. ${\left[\alpha \right]}_{D}^{20}$ = −34.8° (c 0.99, MeOH). IR (ν_max_, cm^−1^): 2955 (-CH_2_), 1693 (C=O), 1397 (-CH_3_), 1158 (C-O). ^1^H NMR (700 MHz, CDCl_3_): (two rotamers are present in the spectrum, ratio ~1:0.8) *δ*_H_ 1.45–1.51 (m, 9H, C(CH_3_)_3_), 2.42–2.48 (m, 1H, Pyrr 4-CH_A_), 2.68–2.74 (m, 0.55H, Pyrr 4-CH_B_ of major rotamer), 2.78–2.84 (m, 0.45H, Pyrr 4-CH_B_ of minor rotamer), 3.53–3.58 (m, 1H, Pyrr 5-CH_A_), 3.76–3.88 (m, 2H, Pyrr 5-CH_B_ and Pyrr 2-CH_A_), 3.93–4.01 (m, 1H, Pyrr 2-CH_B_), 4.04 (s, 3H, CH_3_), 5.22–5.33 (m, 1H, Pyrr 3-H), 7.32–7.37 (m, 1H, 5-H), 7.44–7.48 (m, 1H, 6-H), 7.48–7.53 (m, 1H, 7-H), 8.22–8.28 (m, 1H, 4-H). ^13^C NMR (176 MHz, CDCl_3_): *δ*_C_ 28.48 (C(CH_3_)_3_), 30.38 and 30.88 (Pyrr 4-CH_2_), 44.34 and 44.82 (Pyrr 5-CH_2_), 50.03 and 50.14 (Pyrr 2-CH_2_), 52.06 and 52.11 (CH_3_), 57.42 and 57.99 (Pyrr C-3), 79.77 and 79.84 (C(CH_3_)_3_), 109.30 and 109.40 (C-7), 122.54 (C-4), 123.45 (C-5), 124.02 and 124.06 (C-3a), 127.08 and 127.13 (C-6), 135.14 and 135.27 (C-3), 140.41 and 140.44 (C-7a), 154.33 (Boc C=O), 162.97 and 163.00 (C=O). ^15^N NMR (71 MHz, CDCl_3_): *δ*_N_ −294.5 (Pyrr N-1), −187.3 (N-1), −59.7 (N-2). HRMS (ESI TOF): [M + Na]^+^, found 368.1581. [C_18_H_23_N_3_O_4_ + Na]+ requires 368.1581.

**Methyl 1-[(3*S*)-1-(*tert*-butoxycarbonyl)pyrrolidin-3-yl]-1*H*-indazole-3-carboxylate ((*S*)-19a)**

The reaction was carried out using a general alkylation procedure using 1*H*-indazole-3-carboxylate **18a** and (*R*)-1-Boc-3-methanesulfonyloxypyrrolidine **(*R*)-4**, and the product was purified by column chromatography on silica gel using *n*-hexane/ethyl acetate (2/1, *v*/*v*). Yield 383 mg (37%), white solid, mp 118–119 °C. ${\left[\alpha \right]}_{D}^{20}$ = 34.6° (c 1.47, MeOH). IR (ν_max_, cm^−1^): 2955 (-CH_2_), 1693 (C=O), 1397 (-CH_3_), 1158 (C-O). ^1^H NMR (700 MHz, CDCl_3_): (two rotamers are present in the spectrum at a ratio of ~1:0.8) *δ*_H_ 1.45–1.50 (m, 9H, C(CH_3_)_3_), 2.42–2.47 (m, 1H, Pyrr 4-CH_A_), 2.68–2.73 (m, 0.55H, Pyrr 4-CH_B_ of major rotamer), 2.79–2.84 (m, 0.45H, Pyrr 4-CH_B_ of minor rotamer), 3.54–3.58 (m, 1H, Pyrr 5-CH_A_), 3.77–3.88 (m, 2H, Pyrr 5-CH_B_ and Pyrr 2-CH_A_), 3.93–4.01 (m, 1H, Pyrr 2-CH_B_), 4.04 (s, 3H, CH_3_), 5.25–5.31 (m, 1H, Pyrr 3-H), 7.33–7.36 (m, 1H, 5-H), 7.45–7.48 (m, 1H, 6-H), 7.48–7.53 (m, 1H, 7-H), 8.22–8.28 (m, 1H, 4-H). ^13^C NMR (176 MHz, CDCl_3_): *δ*_C_ 28.47 (C(CH_3_)_3_), 30.38 and 30.87 (Pyrr 4-CH_2_), 44.34 and 44.82 (Pyrr 5-CH_2_), 50.03 and 50.14 (Pyrr 2-CH_2_), 52.06 and 52.11 (CH_3_), 57.42 and 57.99 (Pyrr C-3), 79.77 and 79.84 (C(CH_3_)_3_), 109.30 and 109.39 (C-7), 122.54 (C-4), 123.45 (C-5), 124.02 and 124.05 (C-3a), 127.08 and 127.13 (C-6), 135.14 and 135.27 (C-3), 140.41 and 140.44 (C-7a), 154.33 (Boc C=O), 162.97 and 163.01 (C=O). ^15^N NMR (71 MHz, CDCl_3_): *δ*_N_ −294.4 (Pyrr N-1), −187.3 (N-1), −59.8 (N-2). HRMS (ESI TOF): [M + Na]^+^, found 368.1580. [C_18_H_23_N_3_O_4_ + Na]^+^ requires 368.1581.

**Methyl 2-[(3*R*)-1-(*tert*-butoxycarbonyl)pyrrolidin-3-yl]-2*H*-indazole-3-carboxylate ((*R*)-20a)**

The reaction was carried out using a general alkylation procedure using 1*H*-indazole-3-carboxylate **18a** and (*S*)-1-Boc-3-methanesulfonyloxypyrrolidine **(*S*)-4**, and the product was purified by column chromatography on silica gel using *n*-hexane/ethyl acetate (4/1, *v*/*v*). Yield 404 mg (39%), white solid, mp 92–93 °C. ${\left[\alpha \right]}_{D}^{20}$ = −24.8° (c 1.06, MeOH). IR (ν_max_, cm^−1^): 2974 (-CH_2_-), 1681 (C=O), 1408 (-CH_3_), 1167 (C-O). ^1^H NMR (700 MHz, Acetone-*d*_6_): (two rotamers are present in the spectrum, ratio ~1:1) *δ*_H_ 1.39–1.48 (m, 9H, C(CH_3_)_3_), 2.45–2.64 (m, 2H, Pyrr 4-CH_A_ and Pyrr 4-CH_B_), 3.53–3.60 (m, 1H, Pyrr 5-CH_A_), 3.70–3.78 (m, 1.6H, Pyrr 5-CH_B_ and Pyrr 2-CH_A_ of major rotamer), 3.82–3.86 (m, 0.4H, Pyrr 2-CH_A_ of minor rotamer), 3.93–3.99 (m, 1H, Pyrr 2-CH_B_), 4.02–4.06 (m, 3H, CH_3_), 6.21–6.29 (m, 1H, Pyrr 3-H), 7.27–7.31 (m, 1H, 5-H), 7.33–7.38 (m, 1H, 6-H), 7.76–7.80 (m, 1H, 7-H), 8.01 (d, *J* = 8.5 Hz, 1H, 4-H). ^13^C NMR (176 MHz, Acetone-*d*_6_): *δ*_C_ 28.51 (C(CH_3_)_3_), 31.39 and 32.03 (Pyrr 4-CH_2_), 44.79 and 45.22 (Pyrr 5-CH_2_), 51.87 and 51.93 (Pyrr 2-CH_2_), 52.03 and 52.05 (CH_3_), 60.03 and 60.54 (Pyrr C-3), 79.48 and 79.56 (C(CH_3_)_3_), 118.59 (C-7), 121.29 and 121.33 (C-4), 123.58 and 123.60 (C-3a), 123.69 (C-3), 125.28 and 125.37 (C-5), 126.32 and 126.43 (C-6), 147.27 and 147.36 (C-7a), 154.41 (Boc C=O), 160.87 and 160.92 (C=O). ^15^N NMR (71 MHz, Acetone-*d*_6_): *δ*_N_ −292.8 (Pyrr N-1), −143.0 (N-2), −80.2 (N-1). HRMS (ESI TOF): [M + Na]^+^, found 368.1581. [C_18_H_23_N_3_O_4_ + Na]^+^ requires 368.1581.

**Methyl 2-[(3*S*)-1-(*tert*-butoxycarbonyl)pyrrolidin-3-yl]-2*H*-indazole-3-carboxylate ((*S*)-20a)**

The reaction was carried out using a general alkylation procedure using 1*H*-indazole-3-carboxylate **18a** and (*R*)-1-Boc-3-methanesulfonyloxypyrrolidine **(*R*)-4**, and the product was purified by column chromatography on silica gel using *n*-hexane/ethyl acetate (4/1, *v*/*v*). Yield 373 mg (36%), white solid, mp 92–93 °C. ${\left[\alpha \right]}_{D}^{20}$ = 24.7° (c 1.49, MeOH). IR (ν_max_, cm^−1^): 2974 (-CH_2_-), 1682 (C=O), 1409 (-CH_3_), 1167 (C-O). ^1^H NMR (700 MHz, CDCl_3_): (two rotamers are present in the spectrum, ratio ~1:0.7) *δ*_H_ 1.44–1.50 (m, 9H, C(CH_3_)_3_), 2.42–2.48 (m, 1H, Pyrr 4-CH_A_), 2.61–2.69 (m, 1H, Pyrr 4-CH_B_), 3.55–3.62 (m, 1H, Pyrr 5-CH_A_), 3.76–3.86 (m, 1.6H, Pyrr 5-CH_B_ and Pyrr 2-CH_A_ of major rotamer), 3.88–3.91 (m, 0.4H, Pyrr 2-CH_A_ of minor rotamer), 3.93–3.99 (m, 1H, Pyrr 2-CH_B_), 4.02–4.06 (m, 3H, CH_3_), 6.22–6.28 (m, 1H, Pyrr 3-H), 7.28–7.31 (m, 1H, 5-H), 7.34–7.37 (m, 1H, 6-H), 7.77–7.80 (m, 1H, 7-H), 8.01 (d, *J* = 8.4 Hz, 1H, 4-H). ^13^C NMR (176 MHz, CDCl_3_): *δ*_C_ 28.50 (C(CH_3_)_3_), 31.40 and 32.03 (Pyrr 4-CH_2_), 44.78 and 45.22 (Pyrr 5-CH_2_), 51.86 and 51.93 (Pyrr 2-CH_2_), 52.03 and 52.05 (CH_3_), 60.02 and 60.53 (Pyrr C-3), 79.48 and 79.56 (C(CH_3_)_3_), 118.60 (C-7), 121.29 and 121.33 (C-4), 123.58 and 123.61 (C-3a), 123.69 (C-3), 125.28 and 125.38 (C-5), 126.33 and 126.44 (C-6), 147.28 and 147.37 (C-7a), 154.41 (Boc C=O), 160.88 and 160.93 (C=O). ^15^N NMR (71 MHz, CDCl_3_): *δ*_N_ −292.8 (Pyrr N-1), −143.0 (N-2), −80.2 (N-1). HRMS (ESI TOF): [M + Na]^+^, found 368.1583. [C_18_H_23_N_3_O_4_ + Na]^+^ requires 368.1581.

**Methyl 1-[(3*R*)-1-(*tert*-butoxycarbonyl)pyrrolidin-3-yl]-1*H*-indazole-4-carboxylate ((*R*)-19b)**

The reaction was carried out using a general alkylation procedure using 1*H*-indazole-4-carboxylate **18b** and pyrrolidine mesylate **(*S*)-4**, and the product was purified by column chromatography on silica gel using *n*-hexane/ethyl acetate (2/1, *v*/*v*). Yield: 394 mg (38%), yellow oil. ${\left[\alpha \right]}_{D}^{20}$ = −18.9° (c 0.73, MeOH). IR (ν_max_, cm^−1^): 2976 (-CH_2_-), 1688 (C=O), 1401 (-CH_3_), 1164 (C-O). ^1^H NMR (700 MHz, CDCl_3_): *δ*_H_ 1.45–1.50 (m, 9H, C(CH_3_)_3_), 2.39–2.44 (m, 1H, Pyrr 4-CH_A_), 2.57–2.69 (m, 1H, Pyrr 4-CH_B_), 3.55–3.60 (m, 1H, Pyrr 5-CH_A_), 3.73–3.82 (m, 2H, Pyrr 5-CH_B_ and Pyrr 2-CH_A_), 3.89–3.97 (m, 1H, Pyrr 2-CH_B_), 4.02 (s, 3H, CH_3_), 5.15–5.27 (m, 1H, Pyrr 3-H), 7.43–7.49 (m, 1H, 6-H), 7.63–7.69 (m, 1H, 7-H), 7.94 (d, *J* = 7.2 Hz, 1H, 5-H), 8.48–8.52 (m, 1H, 3-H). ^13^C NMR (176 MHz, CDCl_3_): *δ*_C_ 28.50 (C(CH_3_)_3_), 30.59 and 31.19 (Pyrr 4-CH_2_), 44.43 and 44.91 (Pyrr 5-CH_2_), 50.35 and 50.44 (Pyrr 2-CH_2_), 52.19 (CH_3_), 56.56 and 57.25 (Pyrr C-3), 79.64 and 79.69 (C(CH_3_)_3_), 113.60 and 113.68 (C-7), 122.80 and 122.85 (C-3a), 123.12 and 123.15 (C-4), 124.43 (C-5), 125.66 (C-6), 134.19 and 134.34 (C-3), 139.76 (C-7a), 154.41 (Boc C=O), 166.67 (C=O). ^15^N NMR (71 MHz, CDCl_3_): *δ*_N_ −294.0 (Pyrr N-1), −192.8 (N-2), −65.6 (N-1). HRMS (ESI TOF): [M + Na]^+^, found 368.1581. [C_18_H_23_N_3_O_4_ + Na]^+^ requires 368.1581.

**Methyl 1-[(3*S*)-1-(*tert*-butoxycarbonyl)pyrrolidin-3-yl]-1*H*-indazole-4-carboxylate ((*S*)-19b)**

The reaction was carried out using a general alkylation procedure using 1*H*-indazole-4-carboxylate **18b** and (*R*)-1-Boc-3-methanesulfonyloxypyrrolidine **(*R*)-4**, and the product was purified by column chromatography on silica gel using *n*-hexane/ethyl acetate (2/1, *v*/*v*). Yield: 383 mg (37%), yellow oil. ${\left[\alpha \right]}_{D}^{20}$ = 18.8° (c 0.81, MeOH). IR (ν_max_, cm^−1^): 2976 (-CH_2_-), 1688 (C=O), 1400 (-CH_3_), 1164 (C-O). ^1^H NMR (700 MHz, CDCl_3_): *δ*_H_ 1.45–1.50 (m, 9H, C(CH_3_)_3_), 2.39–2.45 (m, 1H, Pyrr 4-CH_A_), 2.58–2.68 (m, 1H, Pyrr 4-CH_B_), 3.55–3.60 (m, 1H, Pyrr 5-CH_A_), 3.73–3.82 (m, 2H, Pyrr 5-CH_B_ and Pyrr 2-CH_A_), 3.89–3.97 (m, 1H, Pyrr 2-CH_B_), 4.02 (s, 3H, CH_3_), 5.18–5.24 (m, 1H, Pyrr 3-H), 7.44–7.48 (m, 1H, 6-H), 7.64–7.68 (m, 1H, 7-H), 7.94 (d, *J* = 7.2 Hz, 1H, 5-H), 8.50–8.53 (m, 1H, 3-H). ^13^C NMR (176 MHz, CDCl_3_): *δ*_C_ 28.50 (C(CH_3_)_3_), 30.59 and 31.19 (Pyrr 4-CH_2_), 44.43 and 44.91 (Pyrr 5-CH_2_), 50.35 and 50.44 (Pyrr 2-CH_2_), 52.19 (CH_3_), 56.56 and 57.25 (Pyrr C-3), 79.64 and 79.69 (C(CH_3_)_3_), 113.60 and 113.68 (C-7), 122.80 and 122.85 (C-3a), 123.12 and 123.15 (C-4), 124.43 (C-5), 125.66 (C-6), 134.19 and 134.34 (C-3), 139.76 (C-7a), 154.42 (Boc C=O), 166.68 (C=O). ^15^N NMR (71 MHz, CDCl_3_): *δ*_N_ −294.0 (Pyrr N-1), −192.8 (N-2), −65.6 (N-1). HRMS (ESI TOF): [M + Na]^+^, found 368.1583. [C_18_H_23_N_3_O_4_ + Na]^+^ requires 368.1581.

**Methyl 2-[(3*R*)-1-(*tert*-butoxycarbonyl)pyrrolidin-3-yl]-2*H*-indazole-4-carboxylate ((*R*)-20b)**

The reaction was carried out using a general alkylation procedure using 1*H*-indazole-4-carboxylate **18b** and (*S*)-1-Boc-3-methanesulfonyloxypyrrolidine **(*S*)-4**, and the product was purified by column chromatography on silica gel using *n*-hexane/ethyl acetate (2/1, *v*/*v*). Yield: 372 mg (36%), yellow oil. ${\left[\alpha \right]}_{D}^{20}$ = −31.7° (c 0.89, MeOH). IR (ν_max_, cm^−1^): 2976 (-CH_2_-), 1690 (C=O), 1401 (-CH_3_), 1164 (C-O). ^1^H NMR (700 MHz, CDCl_3_): (two rotamers are present in the spectrum, ratio ~1:0.8) *δ*_H_ 1.46–1.52 (m, 9H, C(CH_3_)_3_), 2.50–2.55 (m, 1H, Pyrr 4-CH_A_), 2.57–2.62 (m, 1H, Pyrr 4-CH_B_), 3.57–3.64 (m, 1H, Pyrr 5-CH_A_), 3.66–3.71 (m, 0.45H, Pyrr 5-CH_B_ of minor rotamer), 3.75–3.79 (m, 0.55H, Pyrr 5-CH_B_ of major rotamer), 3.85–3.90 (m, 0.55H, Pyrr 2-CH_A_ of major rotamer), 3.95–4.04 (m, 4.45H, Pyrr 2-CH_A_ of minor rotamer, Pyrr 2-CH_B_ and CH_3_), 5.21 (p, *J* = 6.3 Hz, 1H, Pyrr 3-H), 7.36 (t, *J* = 7.9 Hz, 1H, 6-H), 7.91–7.95 (m, 2H, 5-H and 7-H), 8.46–8.51 (m, 1H, 3-H). ^13^C NMR (176 MHz, CDCl_3_): *δ*_C_ 28.48 (C(CH_3_)_3_), 31.54 and 32.50 (Pyrr 4-CH_2_), 44.27 and 44.62 (Pyrr 5-CH_2_), 51.27 and 51.57 (Pyrr 2-CH_2_), 52.05 (CH_3_), 61.54 and 62.17 (Pyrr C-3), 79.90 and 79.96 (C(CH_3_)_3_), 119.87 (C-3a), 122.35 (C-4), 123.18 and 123.25 (C-3), 123.35 (C-7), 125.20 and 125.28 (C-6), 126.46 (C-5), 149.06 (C-7a), 154.28 and 154.32 (Boc C=O), 166.72 (C=O). ^15^N NMR (71 MHz, CDCl_3_): *δ*_N_ −294.7 (Pyrr N-1), −148.4 (N-1), −99.3 (N-2). HRMS (ESI TOF): [M + Na]^+^, found 368.1581. [C_18_H_23_N_3_O_4_ + Na]^+^ requires 368.1581.

**Methyl 2-[(3*S*)-1-(*tert*-butoxycarbonyl)pyrrolidin-3-yl]-2*H*-indazole-4-carboxylate ((*S*)-20b)**

The reaction was carried out using a general alkylation procedure using 1*H*-indazole-4-carboxylate **18b** and (*R*)-1-Boc-3-methanesulfonyloxypyrrolidine **(*R*)-4**, and the product was purified by column chromatography on silica gel using *n*-hexane/ethyl acetate (2/1, *v*/*v*). Yield 415 mg (40%), yellow oil. ${\left[\alpha \right]}_{D}^{20}$ = 31.7° (c 1.72, MeOH). IR (ν_max_, cm^−1^): 2976 (-CH_2_-), 1689 (C=O), 1400 (-CH_3_), 1164 (C-O). ^1^H NMR (700 MHz, CDCl_3_): (two rotamers are present in the spectrum, ratio ~1:0.8) *δ*_H_ 1.46–1.51 (m, 9H, C(CH_3_)_3_), 2.50–2.56 (m, 1H, Pyrr 4-CH_A_), 2.57–2.62 (m, 1H, Pyrr 4-CH_B_), 3.56–3.64 (m, 1H, Pyrr 5-CH_A_), 3.66–3.71 (m, 0.45H, Pyrr 5-CH_B_ of minor rotamer), 3.75–3.79 (m, 0.55H, Pyrr 5-CH_B_ of major rotamer), 3.86–3.90 (m, 0.55H, Pyrr 2-CH_A_ of major rotamer), 3.95–4.04 (m, 4.45H, Pyrr 2-CH_A_ of minor rotamer, Pyrr 2-CH_B_ and CH_3_), 5.21 (p, *J* = 6.1 Hz, 1H, Pyrr 3-H), 7.36 (t, *J* = 7.9 Hz, 1H, 6-H), 7.90–7.95 (m, 2H, 5-H and 7-H), 8.46–8.50 (m, 1H, 3-H). ^13^C NMR (176 MHz, CDCl_3_): *δ*_C_ 28.48 (C(CH_3_)_3_), 31.54 and 32.50 (Pyrr 4-CH_2_), 44.27 and 44.63 (Pyrr 5-CH_2_), 51.27 and 51.57 (Pyrr 2-CH_2_), 52.05 (CH_3_), 61.54 and 62.16 (Pyrr C-3), 79.90 and 79.95 (C(CH_3_)_3_), 119.88 (C-3a), 122.35 (C-4), 123.17 and 123.25 (C-3), 123.34 (C-7), 125.20 and 125.28 (C-6), 126.45 (C-5), 149.06 (C-7a), 154.28 (Boc C=O), 166.72 (C=O). ^15^N NMR (71 MHz, CDCl_3_): *δ*_N_ −294.7 (Pyrr N-1), −148.6 (N-1), −99.3 (N-2). HRMS (ESI TOF): [M + Na]^+^, found 368.1582. [C_18_H_23_N_3_O_4_ + Na]^+^ requires 368.1581.

**Methyl 1-[(3*R*)-1-(*tert*-butoxycarbonyl)pyrrolidin-3-yl]-1*H*-indazole-5-carboxylate ((*R*)-19c)**

The reaction was carried out using a general alkylation procedure using 1*H*-indazole-5-carboxylate **18c** and (*S*)-1-Boc-3-methanesulfonyloxypyrrolidine **(*S*)-4**, and the product was purified by column chromatography on silica gel using *n*-hexane/ethyl acetate (1/2, *v*/*v*). Yield 455 mg (44%), white solid, mp 86–87 °C. ${\left[\alpha \right]}_{D}^{20}$ = 22.0° (c 1.90, MeOH). IR (ν_max_, cm^−1^): 2977 (-CH_2_-), 1693 (C=O), 1400 (-CH_3_), 1160 (C-O). ^1^H NMR (700 MHz, CDCl_3_): *δ*_H_ 1.44–1.52 (m, 9H, C(CH_3_)_3_), 2.39–2.45 (m, 1H, Pyrr 4-CH_A_), 2.55–2.65 (m, 1H, Pyrr 4-CH_B_), 3.55–3.60 (m, 1H, Pyrr 5-CH_A_), 3.72–3.83 (m, 2H, Pyrr 5-CH_B_ and Pyrr 2-CH_A_), 3.89–3.97 (m, 4H, Pyrr 2-CH_B_ and CH_3_), 5.15–5.24 (m, 1H, Pyrr 3-H), 7.42–7.48 (m, 1H, 7-H), 8.05–8.10 (m, 1H, 6-H), 8.10–8.13 (m, 1H, 3-H), 8.52 (s, 1H, 4-H). ^13^C NMR (176 MHz, CDCl_3_): *δ*_C_ 28.50 (C(CH_3_)_3_), 30.59 and 31.24 (Pyrr 4-CH_2_), 44.43 and 44.91 (Pyrr 5-CH_2_), 50.34 (Pyrr 2-CH_2_), 52.15 (CH_3_), 56.64 and 57.32 (Pyrr C-3), 79.68 and 79.72 (C(CH_3_)_3_), 108.48 and 108.52 (C-7), 123.26 (C-5), 124.01 (C-3a), 124.74 (C-4), 127.26 (C-6), 135.02 and 135.13 (C-3), 141.04 (C-7a), 154.40 (Boc C=O), 167.19 (C=O). ^15^N NMR (71 MHz, CDCl_3_): *δ*_N_ −294.7 (Pyrr N-1), −191.2 (N-2), −65.4 (N-1). HRMS (ESI TOF): [M + Na]^+^, found 368.1581. [C_18_H_23_N_3_O_4_ + Na]^+^ requires 368.1581.

**Methyl 1-[(3*S*)-1-(*tert*-butoxycarbonyl)pyrrolidin-3-yl]-1*H*-indazole-5-carboxylate ((*S*)-19c)**

The reaction was carried out using a general alkylation procedure using 1*H*-indazole-5-carboxylate **18c** and (*R*)-1-Boc-3-methanesulfonyloxypyrrolidine **(*R*)-4**, and the product was purified by column chromatography on silica gel using *n*-hexane/ethyl acetate (1/2, *v*/*v*). Yield 466 mg (45%), white solid, mp 86–87 °C. ${\left[\alpha \right]}_{D}^{20}$ = −22.2° (c 1.39, MeOH). IR (ν_max_, cm^−1^): 2976 (-CH_2_-), 1693 (C=O), 1399 (-CH_3_), 1160 (C-O). ^1^H NMR (700 MHz, CDCl_3_): *δ*_H_ 1.44–1.52 (m, 9H, C(CH_3_)_3_), 2.38–2.45 (m, 1H, Pyrr 4-CH_A_), 2.55–2.65 (m, 1H, Pyrr 4-CH_B_), 3.55–3.60 (m, 1H, Pyrr 5-CH_A_), 3.72–3.83 (m, 2H, Pyrr 5-CH_B_ and Pyrr 2-CH_A_), 3.89–3.97 (m, 4H, Pyrr 2-CH_B_ and CH_3_), 5.16–5.23 (m, 1H, Pyrr 3-H), 7.43–7.46 (m, 1H, 7-H), 8.06–8.10 (m, 1H, 6-H), 8.10–8.13 (m, 1H, 3-H), 8.52 (s, 1H, 4-H). ^13^C NMR (176 MHz, CDCl_3_): *δ*_C_ 28.49 (C(CH_3_)_3_), 30.59 and 31.24 (Pyrr 4-CH_2_), 44.43 and 44.91 (Pyrr 5-CH_2_), 50.34 (Pyrr 2-CH_2_), 52.14 (CH_3_), 56.64 and 57.32 (Pyrr C-3), 79.67 and 79.71 (C(CH_3_)_3_), 108.48 and 108.52 (C-7), 123.24 (C-5), 124.01 (C-3a), 124.73 (C-4), 127.25 (C-6), 135.02 and 135.12 (C-3), 141.03 (C-7a), 154.39 (Boc C=O), 167.18 (C=O). ^15^N NMR (71 MHz, CDCl_3_): *δ*_N_ −294.5 (Pyrr N-1), −191.3 (N-2), −65.4 (N-1). HRMS (ESI TOF): [M + Na]^+^, found 368.1582. [C_18_H_23_N_3_O_4_ + Na]^+^ requires 368.1581.

**Methyl 2-[(3*R*)-1-(*tert*-butoxycarbonyl)pyrrolidin-3-yl]-2*H*-indazole-5-carboxylate ((*R*)-20c)**

The reaction was carried out using a general alkylation procedure using 1*H*-indazole-5-carboxylate **18c** and (*S*)-1-Boc-3-methanesulfonyloxypyrrolidine **(*S*)-4**, and the product was purified by column chromatography on silica gel using *n*-hexane/ethyl acetate (1/1, *v*/*v*). Yield 425 mg (41%), white solid, mp 96–97 °C. ${\left[\alpha \right]}_{D}^{20}$ = −19.3° (c 0.88, MeOH). IR (ν_max_, cm^−1^): 2972 (-CH_2_-), 1690 (C=O), 1386 (-CH_3_), 1168 (C-O). ^1^H NMR (700 MHz, CDCl_3_): (two rotamers are present in a ~1:0.4 ratio) *δ*_H_ 1.46–1.52 (m, 9H, C(CH_3_)_3_), 2.49–2.59 (m, 2H, Pyrr 4-CH_2_), 3.58–3.64 (m, 1.4H, Pyrr 5-CH_2_ of major rotamer), 3.71–3.76 (m, 0.6H, Pyrr 5-CH_2_ of minor rotamer), 3.86–3.89 (m, 0.6H, Pyrr 2-CH_2_ of minor rotamer), 3.94 (s, 3H, CH_3_), 3.97–4.00 (m, 1.4H, Pyrr 2-CH_2_ of major rotamer), 5.20 (p, *J* = 6.0 Hz, 1H, Pyrr 3-H), 7.70 (d, *J* = 9.1 Hz, 1H, 7-H), 7.91 (d, *J* = 9.1 Hz, 1H, 6-H), 8.10 (s, 1H, 3-H), 8.49 (s, 1H, 4-H). ^13^C NMR (176 MHz, CDCl_3_): *δ*_C_ 28.47 (C(CH_3_)_3_), 31.50 and 32.53 (Pyrr 4-CH_2_), 44.17 and 44.45 (Pyrr 5-CH_2_), 51.13 and 51.58 (Pyrr 2-CH_2_), 52.09 (CH_3_), 61.67 and 62.34 (Pyrr C-3), 80.07 (C(CH_3_)_3_), 117.37 (C-7), 121.06 (C-3a), 123.61 and 123.65 (C-3), 124.00 and 124.06 (C-5), 124.87 (C-4), 126.01 and 126.08 (C-6), 150.06 (C-7a), 154.25 and 154.35 (Boc C=O), 167.41 (C=O). ^15^N NMR (71 MHz, CDCl_3_): *δ*_N_ −294.5 (Pyrr N-1), −147.1 (N-1), −98.9 (N-2). HRMS (ESI TOF): [M + Na]^+^, found 368.1581. [C_18_H_23_N_3_O_4_ + Na]^+^ requires 368.1581.

**Methyl 2-[(3*S*)-1-(*tert*-butoxycarbonyl)pyrrolidin-3-yl]-2*H*-indazole-5-carboxylate ((*S*)-20c)**

The reaction was carried out using a general alkylation procedure using 1*H*-indazole-5-carboxylate **18c** and (*R*)-1-Boc-3-methanesulfonyloxypyrrolidine **(*R*)-4**, and the product was purified by column chromatography on silica gel using *n*-hexane/ethyl acetate (1/1, *v*/*v*). Yield 456 mg (44%), white solid, mp 96–97 °C. ${\left[\alpha \right]}_{D}^{20}$ = 19.5° (c 1.25, MeOH). IR (ν_max_, cm^−1^): 2975 (-CH_2_-), 1690 (C=O), 1393 (-CH_3_), 1161 (C-O). ^1^H NMR (700 MHz, CDCl_3_): (two rotamers are present in a ~1:0.4 ratio) *δ*_H_ 1.46–1.51 (m, 9H, C(CH_3_)_3_), 2.49–2.59 (m, 2H, Pyrr 4-CH_2_), 3.57–3.65 (m, 1.4H, Pyrr 5-CH_2_ of major rotamer), 3.72–3.76 (m, 0.6H, Pyrr 5-CH_2_ of minor rotamer), 3.86–3.90 (m, 0.6H, Pyrr 2-CH_2_ of minor rotamer), 3.94 (s, 3H, CH_3_), 3.96–4.00 (m, 1.4H, Pyrr 2-CH_2_ of major rotamer), 5.20 (p, *J* = 6.0 Hz, 1H, Pyrr 3-H), 7.70 (d, *J* = 9.1 Hz, 1H, 7-H), 7.91 (d, *J* = 9.1 Hz, 1H, 6-H), 8.11 (s, 1H, 3-H), 8.49 (s, 1H, 4-H). ^13^C NMR (176 MHz, CDCl_3_): *δ*_C_ 28.47 (C(CH_3_)_3_), 31.50 and 32.52 (Pyrr 4-CH_2_), 44.17 and 44.45 (Pyrr 5-CH_2_), 51.13 and 51.57 (Pyrr 2-CH_2_), 52.08 (CH_3_), 61.66 and 62.34 (Pyrr C-3), 80.07 (C(CH_3_)_3_), 117.36 (C-7), 121.06 (C-3a), 123.63 and 123.68 (C-3), 124.00 and 124.06 (C-5), 124.87 (C-4), 126.01 and 126.07 (C-6), 150.06 (C-7a), 154.25 and 154.36 (Boc C=O), 167.42 (C=O). ^15^N NMR (71 MHz, CDCl_3_): *δ*_N_ −294.5 (Pyrr N-1), −147.1 (N-1), −98.9 (N-2). HRMS (ESI TOF): [M + Na]^+^, found 368.1586. [C_18_H_23_N_3_O_4_ + Na]^+^ requires 368.1581.

## 4. Conclusions

To summarize, alkylation of indole-, pyrazole-, and indazolecarboxylates using chiral mesylated pyrrolidines produced a diverse library of biheterocyclic Boc-amino esters. For non-symmetrical pyrazole- and indazolecarboxylates, the reaction yielded mixtures of two regioisomers, which were separated via column chromatography. Stereochemical inversion during alkylation was confirmed by single-crystal X-ray diffraction. The enantiomeric purity of the products was assessed using chiral HPLC. Complementary dipeptide-coupling reactions confirmed that both the carboxyl and amino functionalities of the synthesized pyrrolidine hybrids can be selectively modified under standard HATU-mediated conditions. Selected representatives, namely, methyl 1-[(3*R*)-1-(*tert*-butoxycarbonyl)pyrrolidin-3-yl]-1*H*-indole-5-carboxylate and ethyl 1-[(3*R*)-1-(*tert*-butoxycarbonyl)pyrrolidin-3-yl]-1*H*-pyrazole-5-carboxylate, underwent further functionalization. This process included halogenation followed by palladium-catalyzed cross-coupling reactions to introduce aryl, heteroaryl, or alkynyl groups. Detailed NMR studies using standard and advanced methods enabled unambiguous assignment of all structures, while in some cases, additional band-selective HMBC and NOESY/EXSY experiments were required to resolve challenging assignments of certain *N*-Boc-substituted biheterocycle–pyrrolidine carboxylates. Overall, the developed methodology offers access to novel chiral heterocyclic building blocks for peptide engineering, medicinal chemistry, and DNA-encoded library applications.

## Figures and Tables

**Table 1:** Optimization of the coupling reaction of **1a** with 3-functionalized *N*-Boc-pyrrolidines.

Entry	Pyrrolidines	X	Conditions	Product	Yield % ^a^
1	**(*S*)-2**	OH	DIAD, PPh_3_, DCM, 48 h, rt	**(*R*)-5a**	11
2	**(*R*)-3**	I	Cs_2_CO_3_, DMF, 100 °C, 18 h	**(*S*)-5a**	19
3	**(*R*)-3**	I	K_2_CO_3_, DMF, 100 °C, 18 h	**(*S*)-5a**	8
4	**(*S*)-4**	OMs	Cs_2_CO_3_, DMF, 80 °C, 4 h	**(*R*)-5a**	92

^a^ Isolated yield.

**Table 2:** Screening of selected reaction conditions for the Suzuki–Miyaura coupling of 3-iodoindole **(*R*)-10** with phenylboronic acid **11a**.

Entry	[Pd] (5 mol%)	Base (3 Equiv)	Solvent	Temp. (°C)	Time	(*R*)-12a (Yield %) ^a^
1	Pd(PPh_3_)_4_	K_3_PO_4_	1,4-dioxane	100	2 h	32
2	Pd-PEPPSI-IPr	K_3_PO_4_	1,4-dioxane	100	4 h	6
3	Pd(dppf)Cl_2_·DCM	K_3_PO_4_	1,4-dioxane/H_2_O	80	1 h	57
4	Pd(dppf)Cl_2_·DCM	K_3_PO_4_	1,4-dioxane/H_2_O	80 ^b^	15 min	61

^a^ Isolated yield. ^b^ Microwave irradiation (MW), 100 W.

## Data Availability

The original contributions presented in this study are included in the article/Supplementary Materials. Further inquiries can be directed to the corresponding authors.

## Supplementary Materials

The following supporting information can be downloaded at: https://www.mdpi.com/article/10.3390/molecules31152736/s1, Figures S1–S199: NMR spectra and HRMS spectral data; Figures S200–S203 and Tables S1–S26: X-ray analysis; Figures S204–S227: Chiral HPLC chromatograms.
