# Supplementary material for: Facile Synthesis of Indole–, Pyrazole–, and Indazole–Pyrrolidine Hybrids as Novel Chiral Heterocyclic Building Blocks
Source: Molecules. 2026 Aug 6;31(15):2736. doi: 10.3390/molecules31152736 (PMC13468305; doi:10.3390/molecules31152736)
Supplement: Supplementary file 1 [file molecules-31-02736-s001.zip › molecules-4397315-supplementary.pdf]

Supplementary materials

Facile Synthesis of Indole–, Pyrazole–, and Indazole–Pyrrolidine Hybrids as Novel Chiral Heterocyclic Building Blocks

Rokas Jankauskas [1,2]*, Neringa Kleizienė [1], Greta Račkauskienė [1], Aurimas Bieliauskas [1], Miglė Dagilienė [1], Sonata Krikštolė [2], Sergey Belyakov [3], Frank A. Sløk [4], Algirdas Šačkus [1,2]*

[1] Institute of Synthetic Chemistry, Kaunas University of Technology, K. Baršausko g. 59, Kaunas LT-51423, Lithuania

[2] Department of Organic Chemistry, Kaunas University of Technology, Radvilėnų pl. 19, Kaunas LT-50254, Lithuania

[3] Latvian Institute of Organic Synthesis, Aizkraukles 21, LV-1006 Riga, Latvia

[4] Vipergen ApS, Gammel Kongevej 23A, Copenhagen V DK-1610, Denmark

* Correspondence: rokas.jankauskas@ktu.lt, algirdas.sackus@ktu.lt

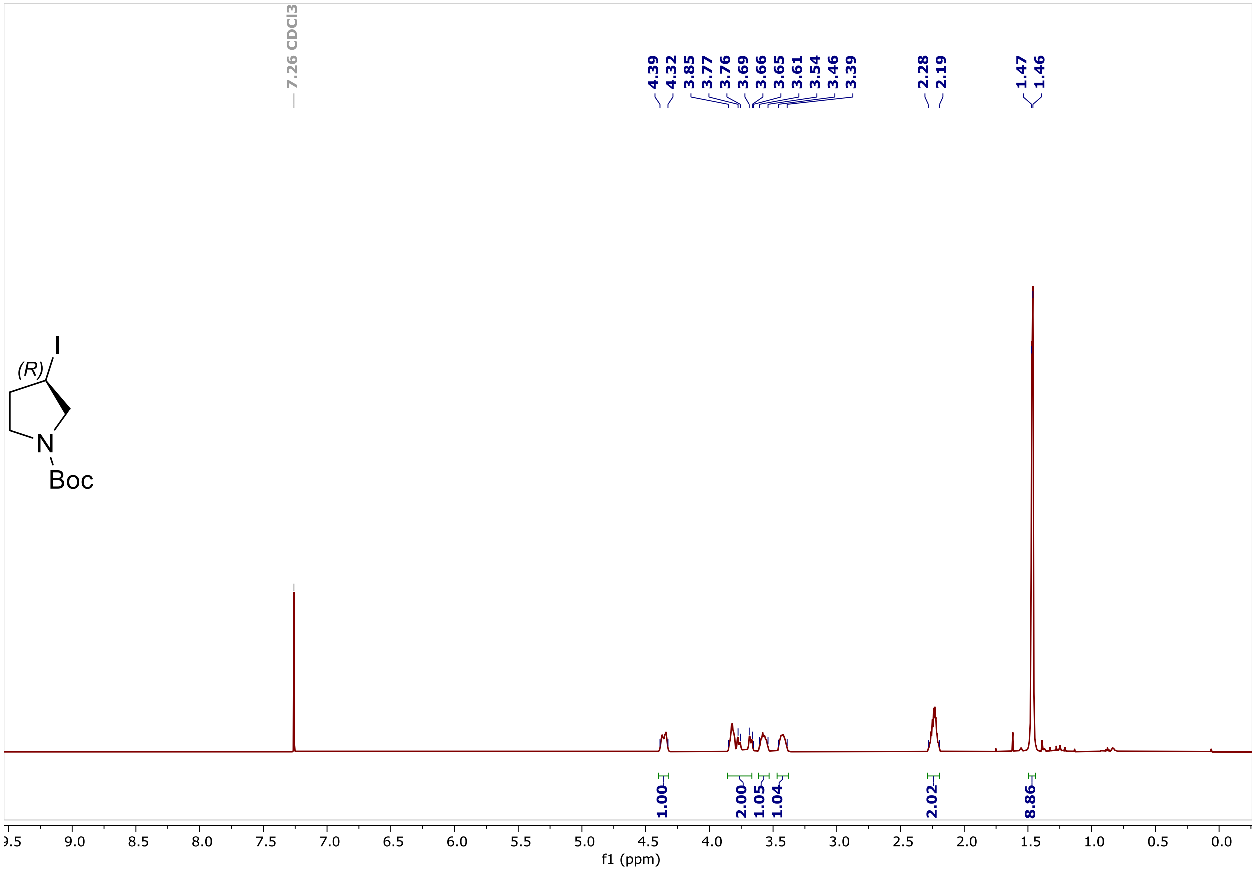

Figure S1. ^{1}H NMR (700 MHz, $CDCl_3$) spectrum of ***(R)*-3**.

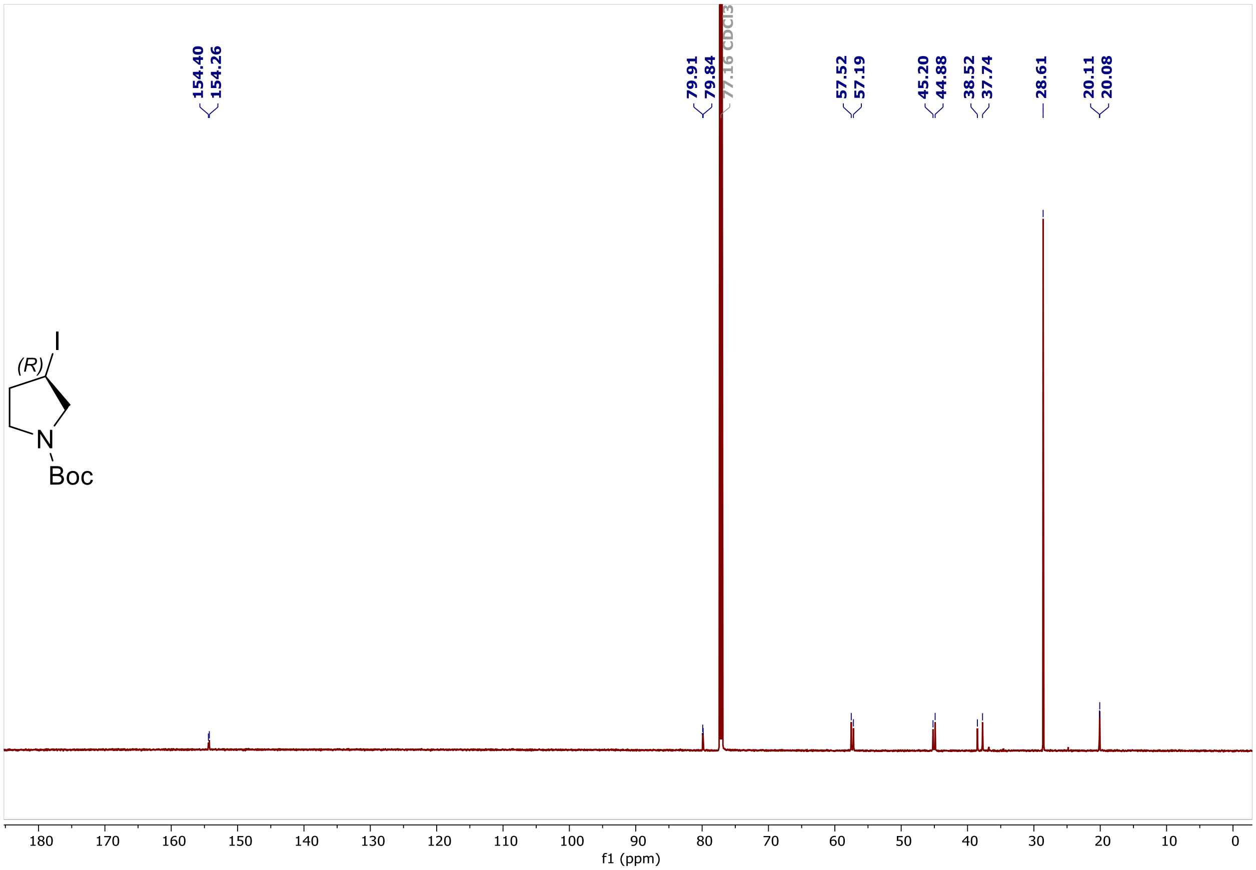

Figure S2. ^{13}C NMR (176 MHz, $CDCl_3$) spectrum of ***(R)*-3**.

Acquisition Parameter

Source Type	ESI	Ion Polarity	Positive	Set Nebulizer	0.4 Bar
Focus	Not active	Set Capillary	4500 V	Set Dry Heater	180 °C
Scan Begin	50 m/z	Set End Plate Offset	-500 V	Set Dry Gas	4.0 l/min
Scan End	1000 m/z	Set Collision Cell RF	140.0 Vpp	Set Divert Valve	Waste

#	RT [min]	Area	Int. Type	I	S/N	Chromatogram	Max. m/z	FWHM [min]
n.a.	5.3	n.a.	Single spectrum	n.a.	n.a.	n.a.	320.0121	n.a.

+MS, 5.3min #316

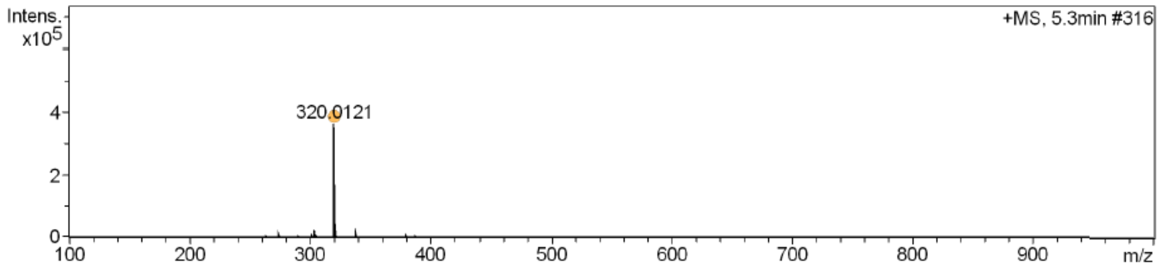

Meas. m/z	#	Ion Formula	m/z	err [ppm]	mSigma	# Sigma	Score	rdb	e⁻ Conf	N-Rule
320.0121	1	C9H16INNaO2	320.0118	-0.8	9.0	2	100.00	1.5	even	ok

Figure S3. HRMS (ESI$^+$) report of ***(R)*-3**.

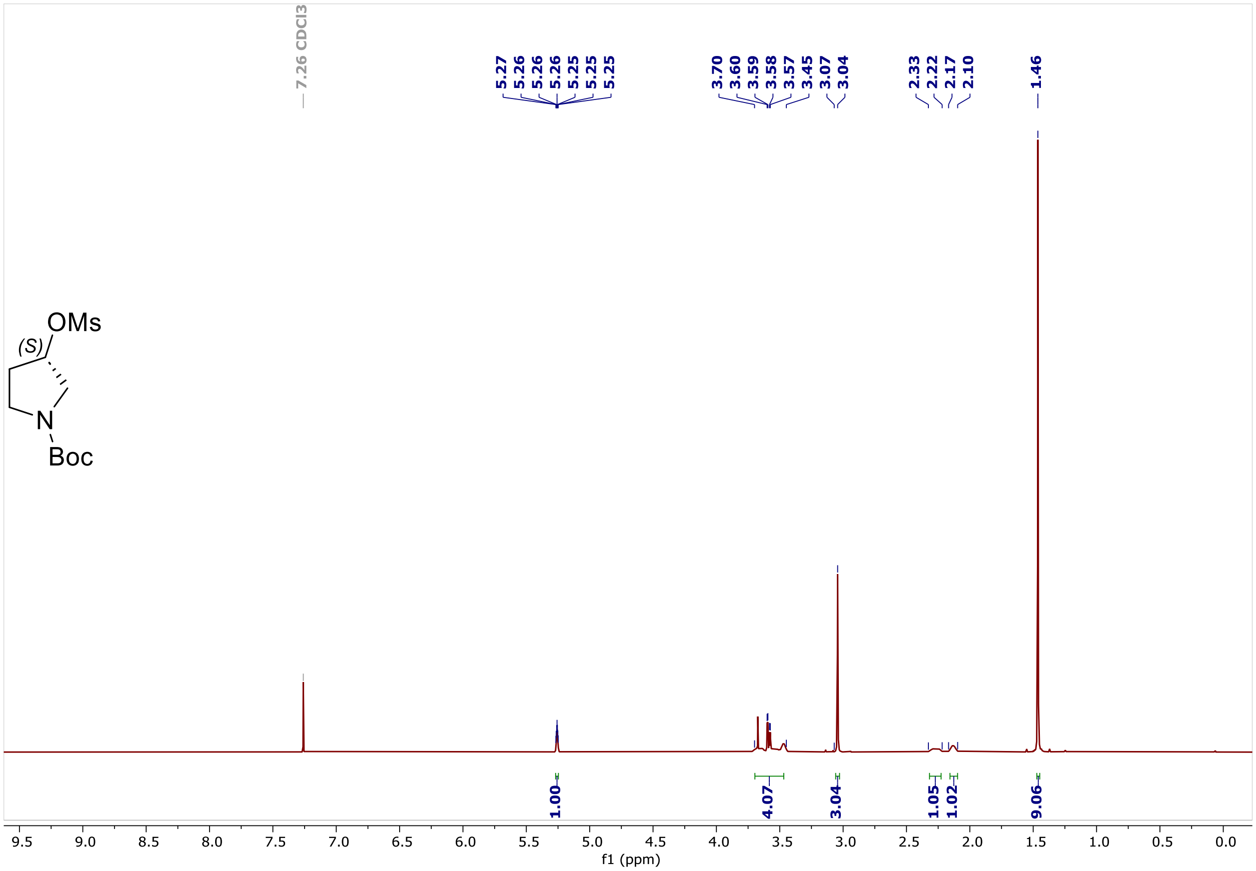

Figure S4. ^{1}H NMR (700 MHz, $CDCl_3$) spectrum of ***(S)*-4**.

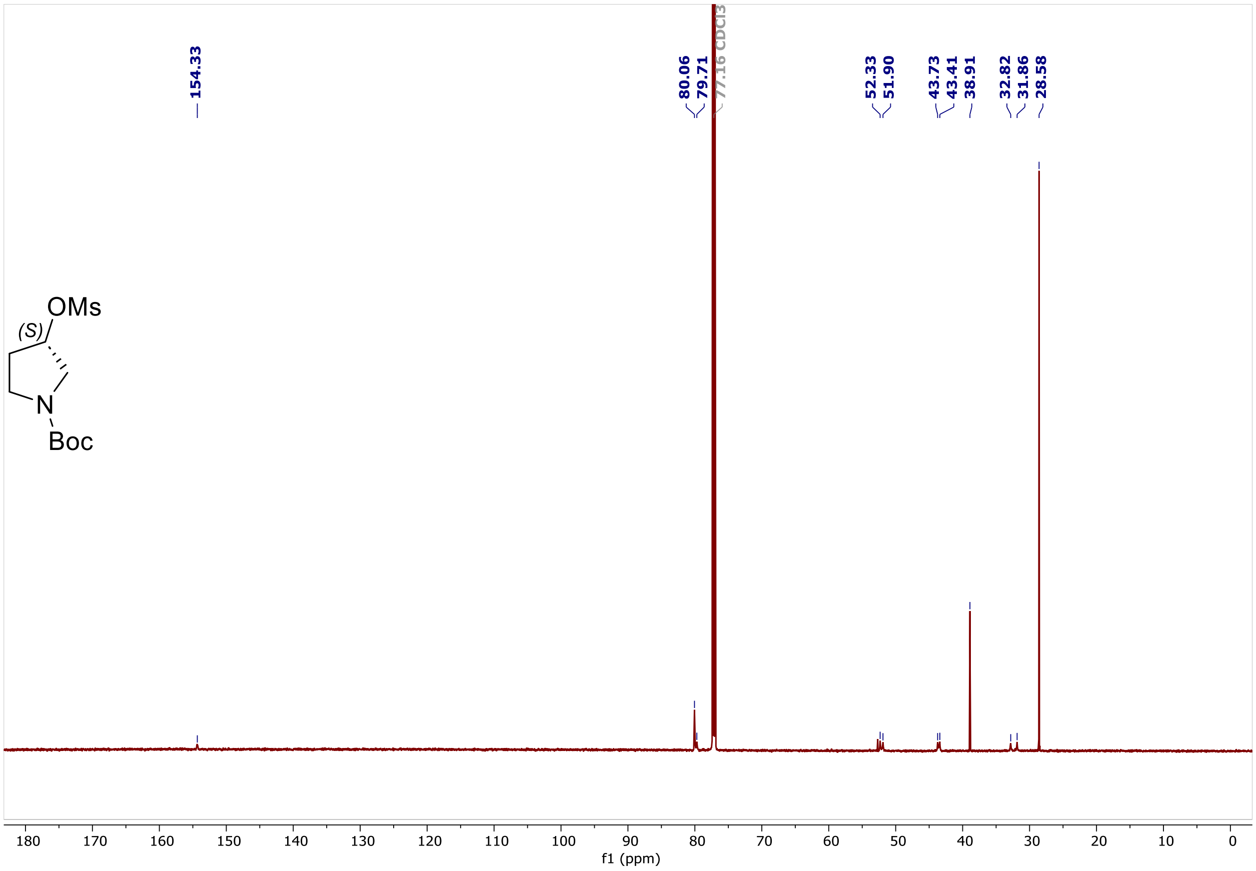

Figure S5. ^{13}C NMR (176 MHz, $CDCl_3$) spectrum of **(*S*)-4**.

Acquisition Parameter

Source Type	ESI	Ion Polarity	Positive	Set Nebulizer	0.4 Bar
Focus	Not active	Set Capillary	4500 V	Set Dry Heater	180 °C
Scan Begin	50 m/z	Set End Plate Offset	-500 V	Set Dry Gas	4.0 l/min
Scan End	1000 m/z	Set Collision Cell RF	140.0 Vpp	Set Divert Valve	Waste

#	RT [min]	Area	Int. Type	I	S/N	Chromatogram	Max. m/z	FWHM [min]
n.a.	2.9	n.a.	Single spectrum	n.a.	n.a.	n.a.	288.0879	n.a.

+MS, 2.9min #174

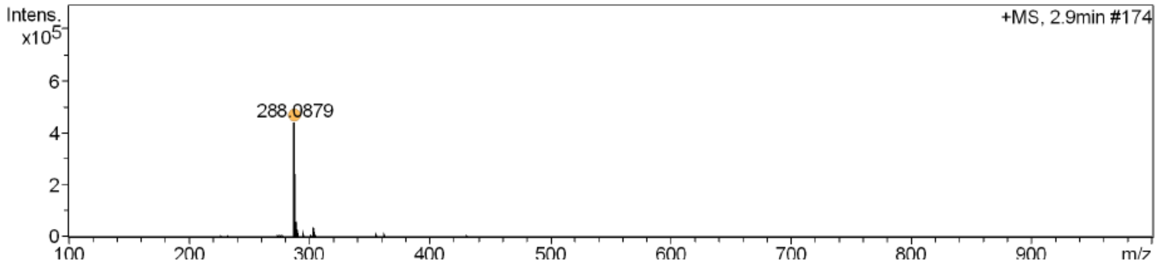

Meas. m/z	#	Ion Formula	m/z	err [ppm]	mSigma	# Sigma	Score	rdb	e⁻ Conf	N-Rule
288.0879	1	C10H19NNaO5S	288.0876	1.1	4.2	1	100.00	1.5	even	ok

Figure S6. HRMS (ESI$^+$) report of **(*S*)-4**.

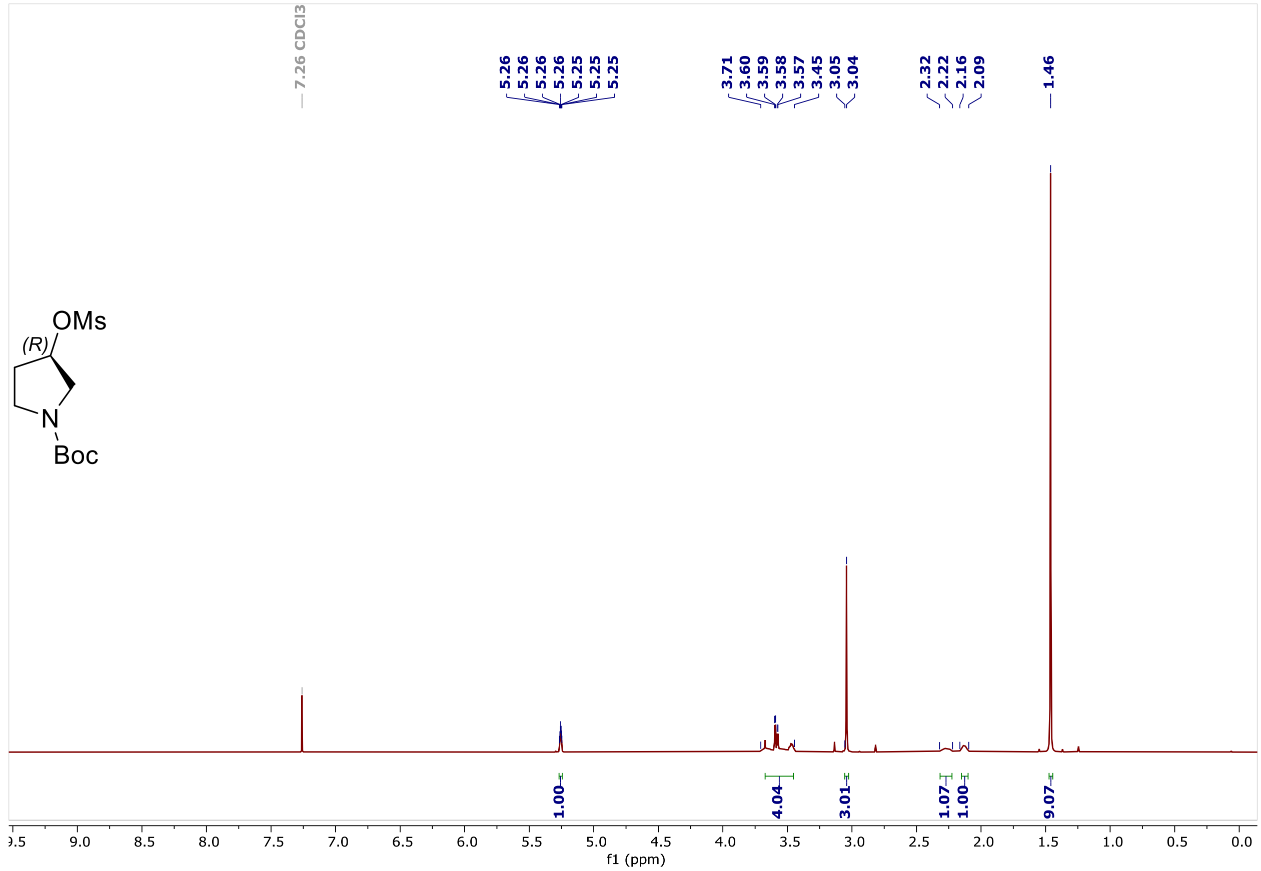

Figure S7. ^{1}H NMR (700 MHz, $CDCl_3$) spectrum of ***(R)*-4**.

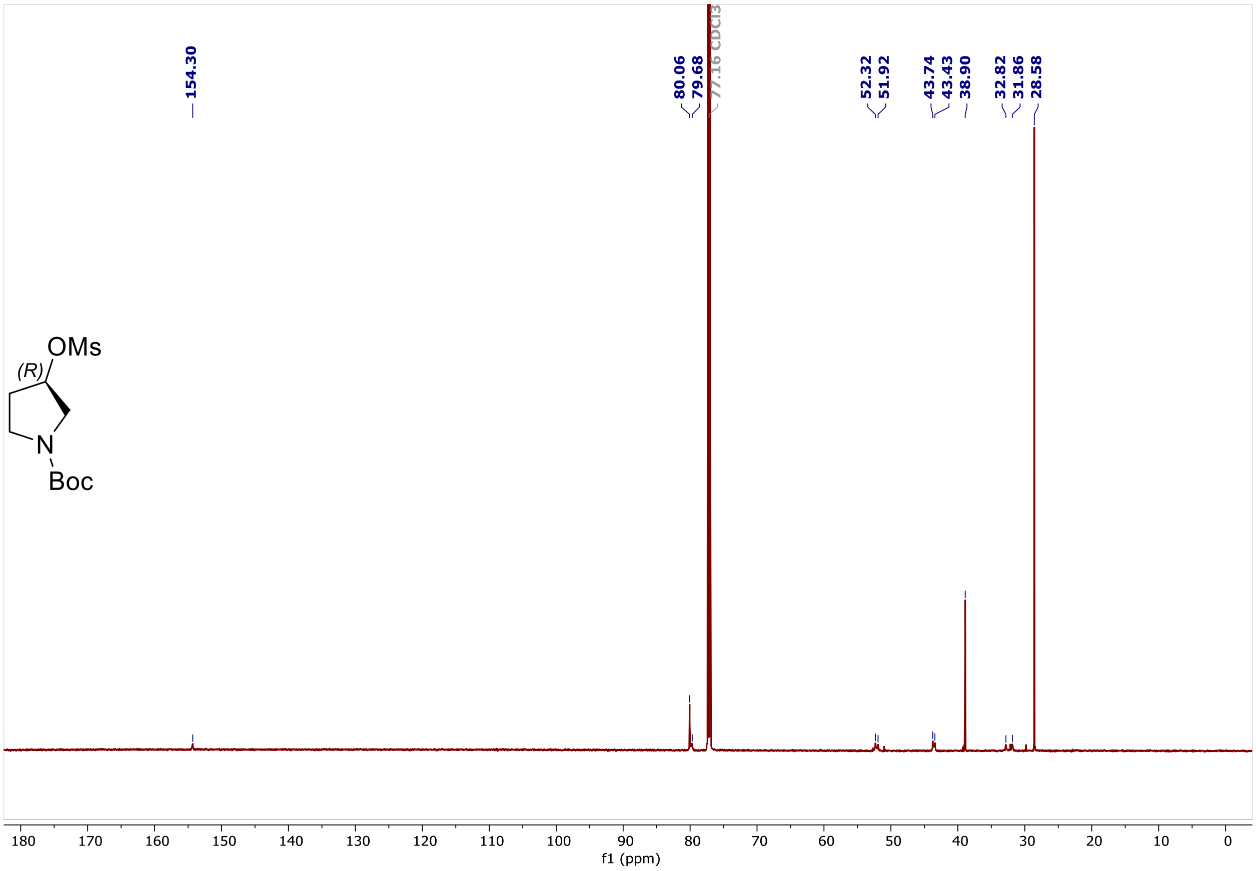

Figure S8. ^{13}C NMR (176 MHz, $CDCl_3$) spectrum of **(*R*)-4**.

Acquisition Parameter

Source Type	ESI	Ion Polarity	Positive	Set Nebulizer	0.4 Bar
Focus	Not active	Set Capillary	4500 V	Set Dry Heater	180 °C
Scan Begin	50 m/z	Set End Plate Offset	-500 V	Set Dry Gas	4.0 l/min
Scan End	1000 m/z	Set Collision Cell RF	140.0 Vpp	Set Divert Valve	Waste

#	RT [min]	Area	Int. Type	I	S/N	Chromatogram	Max. m/z	FWHM [min]
n.a.	3.5	n.a.	Single spectrum	n.a.	n.a.	n.a.	288.0878	n.a.

+MS, 3.5min #211

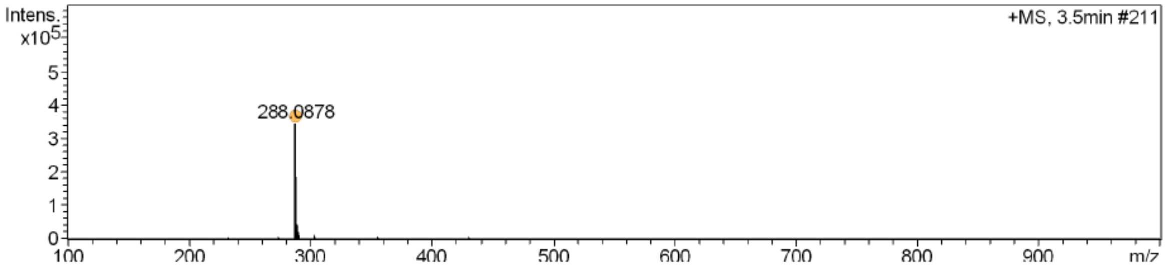

Meas. m/z	#	Ion Formula	m/z	err [ppm]	mSigma	# Sigma	Score	rdb	e⁻ Conf	N-Rule
288.0878	1	C10H19NNaO5S	288.0876	0.8	2.1	1	100.00	1.5	even	ok

Figure S9. HRMS (ESI^+) report of ***(R)*-4**.

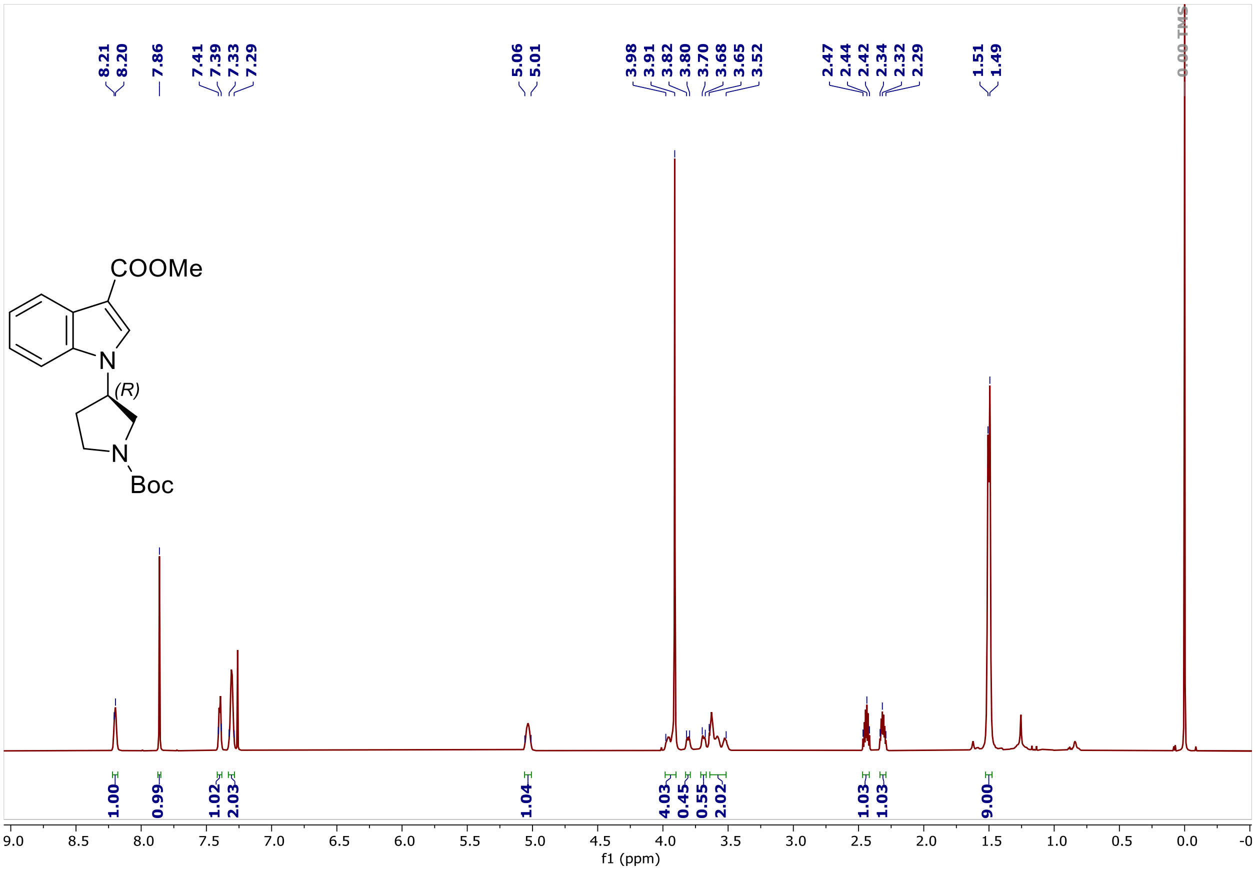

Figure S10. ^{1}H NMR (700 MHz, $CDCl_3$) spectrum of ***(R)*-5a**.

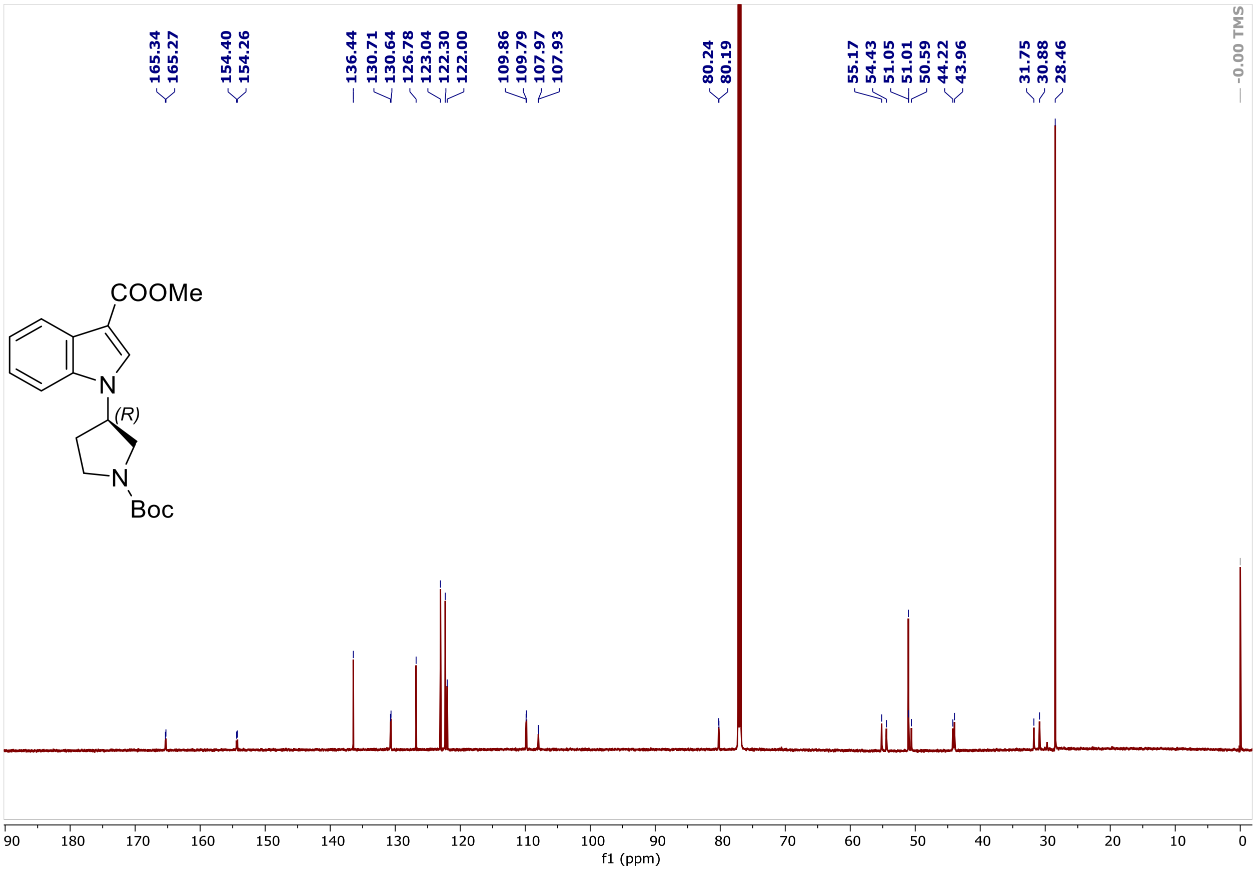

Figure S11. ^{13}C NMR (176 MHz, $CDCl_3$) spectrum of **(*R*)-5a**.

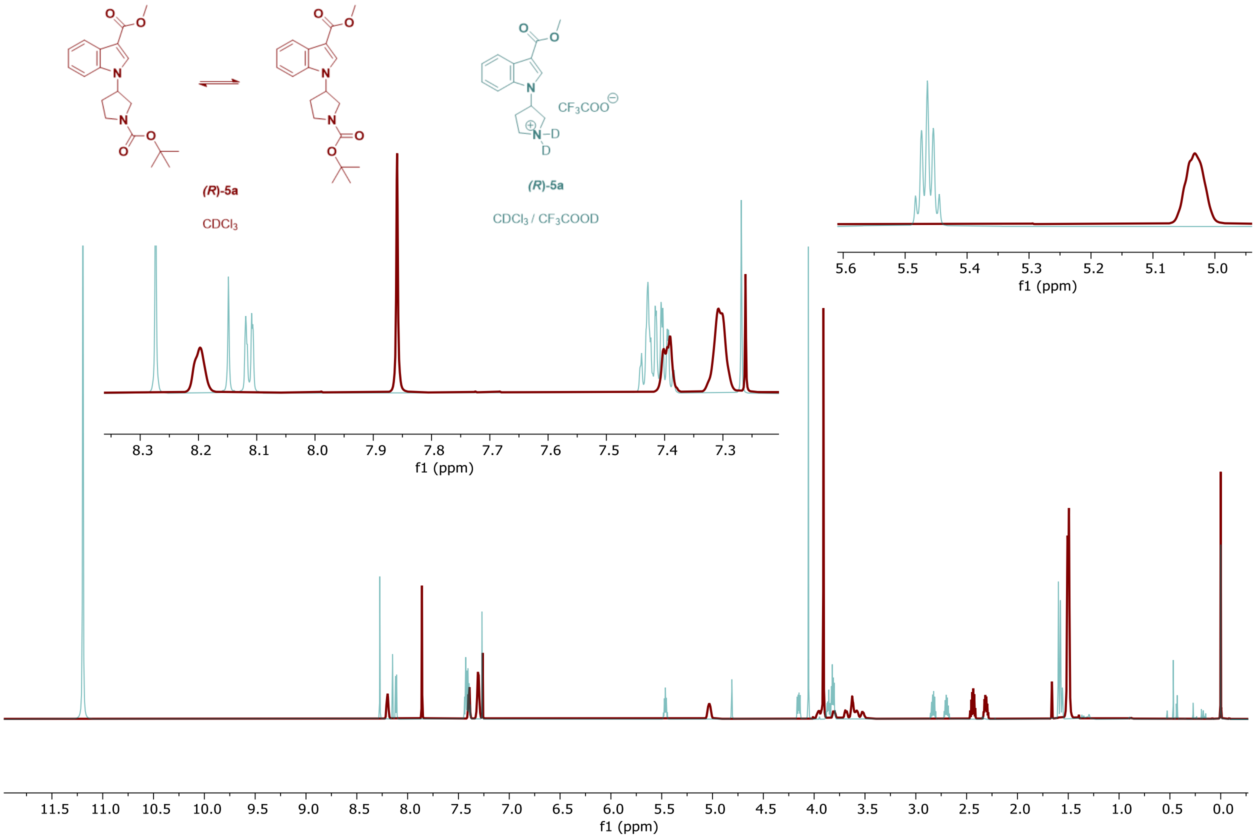

Figure S12. The overlaid ^{1}H NMR spectra of ***(R)*-5a** at 700 MHz in $CDCl_3$ (maroon) and in $CDCl_3/CF_3COOD$ (cyan).

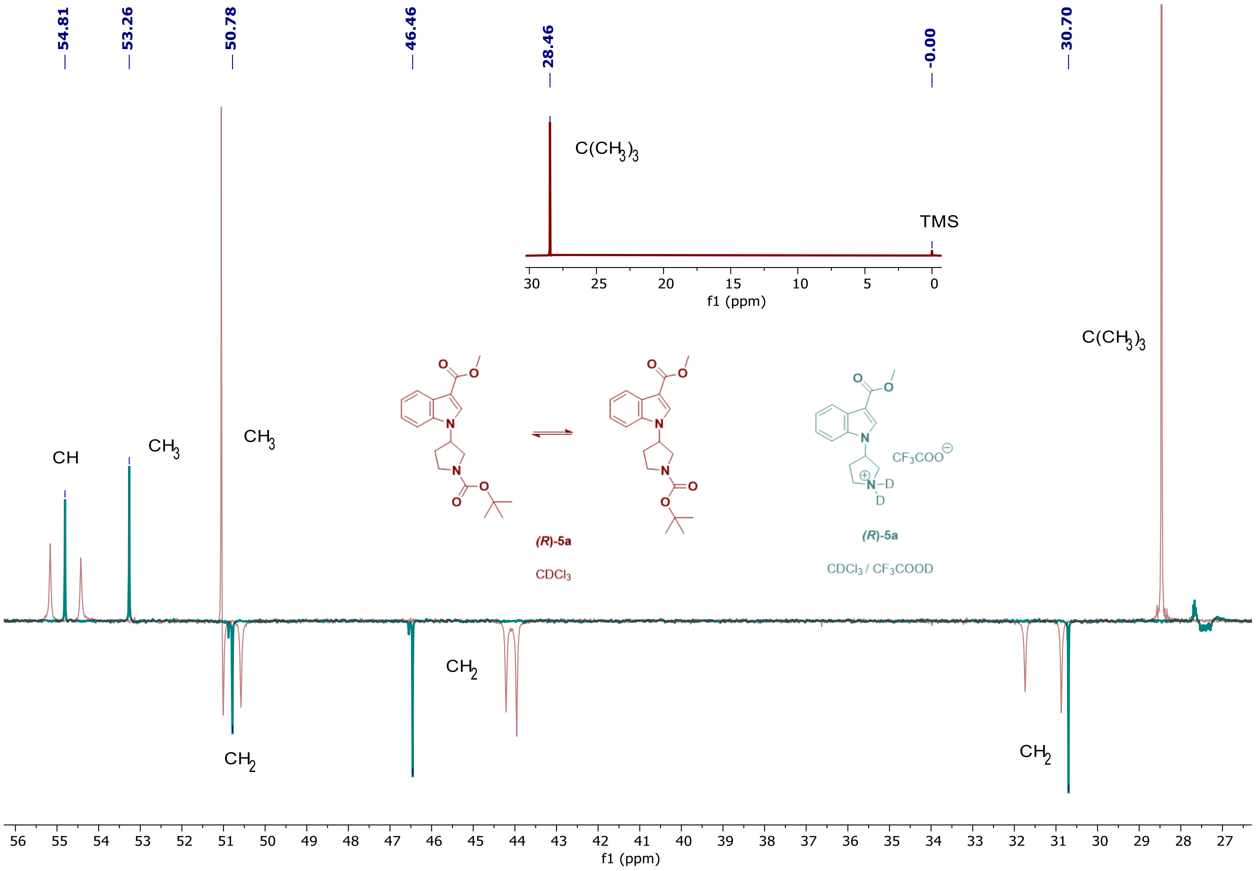

Figure S13. The overlaid ^{13}C NMR DEPT-135 spectra of ***(R)*-5a** at 176MHz in $CDCl_3$ (maroon) and in $CDCl_3/CF_3COOD$ (cyan).

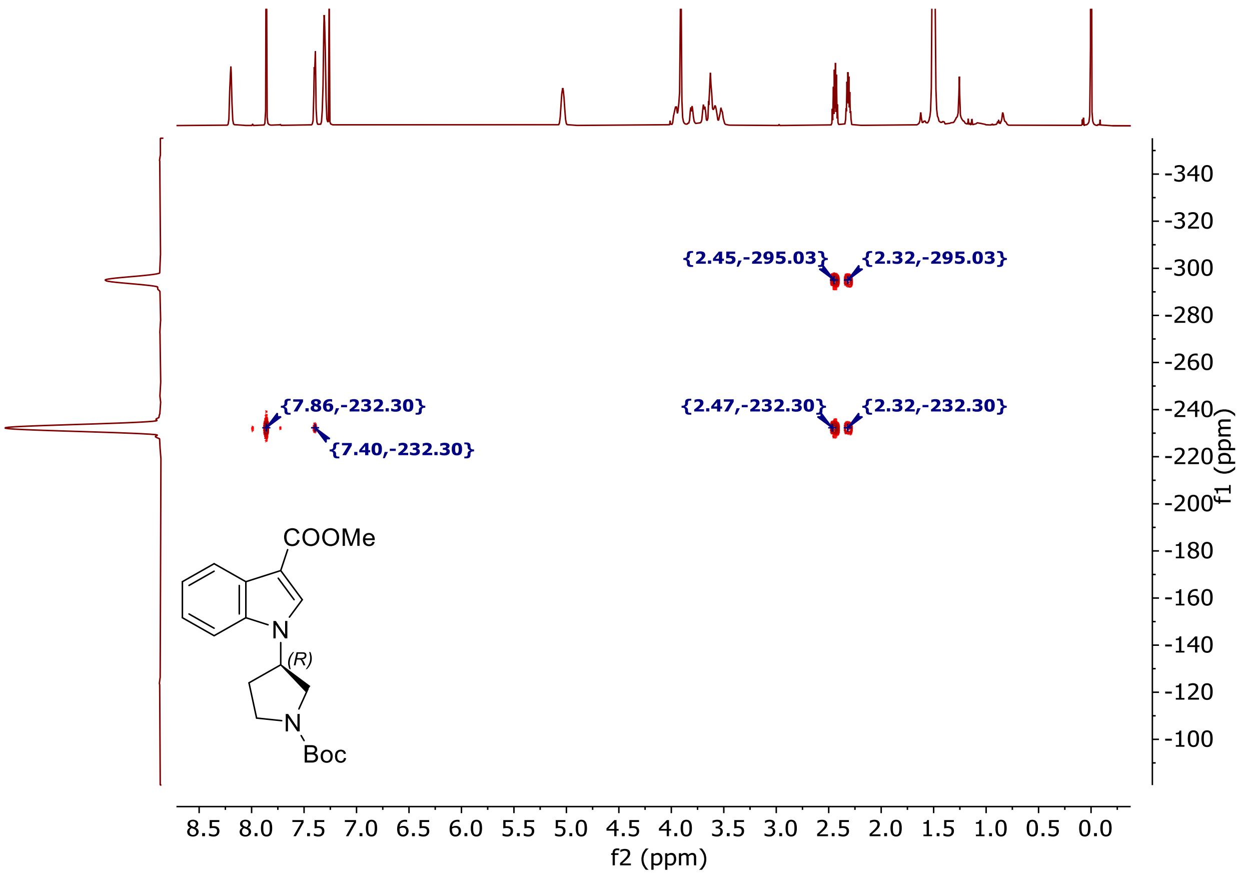

Figure S14. ^{1}H-^{15}N HMBC NMR spectrum of ***(R)*-5a** (71 MHz, $CDCl_3$).

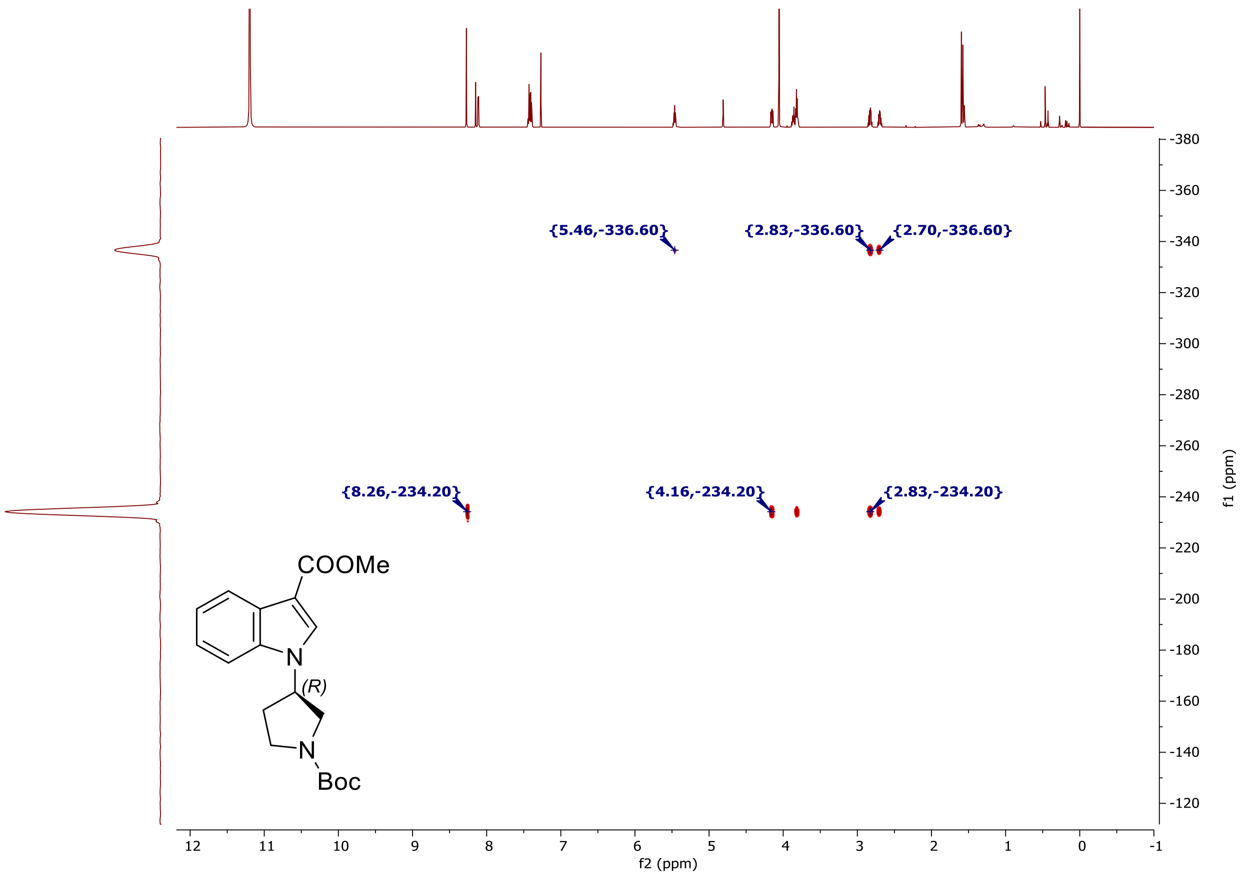

Figure S15. ^{1}H-^{15}N HMBC NMR spectrum of ***(R)*-5a** (71 MHz, $CDCl_3/CF_3COOD$).

Acquisition Parameter

Source Type	ESI	Ion Polarity	Positive	Set Nebulizer	0.4 Bar
Focus	Not active	Set Capillary	4500 V	Set Dry Heater	180 °C
Scan Begin	50 m/z	Set End Plate Offset	-500 V	Set Dry Gas	4.0 l/min
Scan End	1000 m/z	Set Collision Cell RF	140.0 Vpp	Set Divert Valve	Waste

#	RT [min]	Area	Int. Type	I	S/N	Chromatogram	Max. m/z	FWHM [min]
n.a.	0.1	n.a.	Single spectrum	n.a.	n.a.	n.a.	304.2619	n.a.
n.a.	4.3	n.a.	Single spectrum	n.a.	n.a.	n.a.	367.1624	n.a.

+MS, 4.3min #259

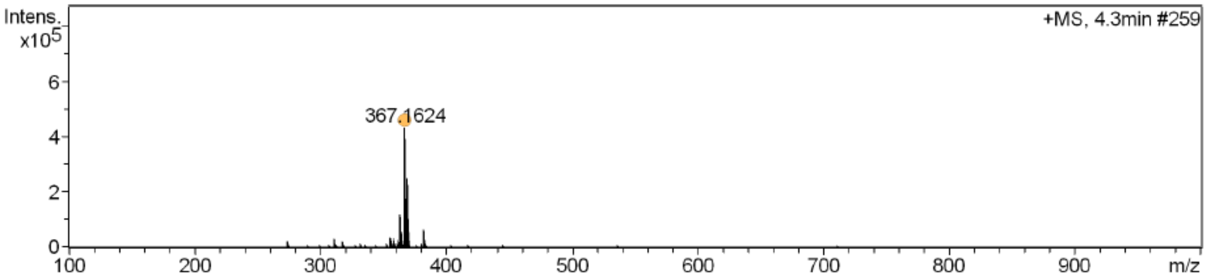

Meas. m/z	#	Ion Formula	m/z	err [ppm]	mSigma	# Sigma	Score	rdb	e⁻ Conf	N-Rule
367.1624	1	C19H24N2NaO4	367.1628	1.2	11.6	1	100.00	8.5	even	ok

Figure S16. HRMS (ESI^+) report of **(*R*)-5a**.

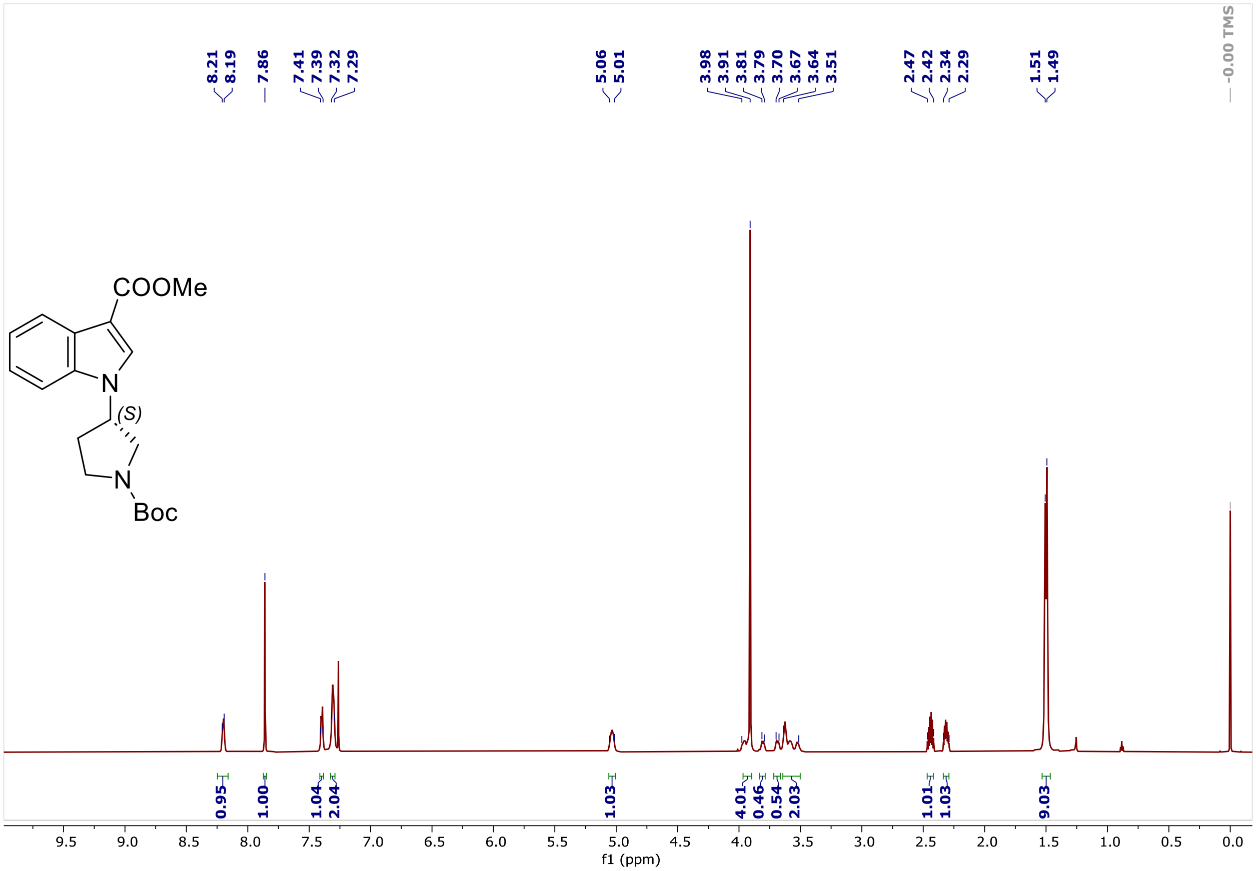

Figure S17. ^{1}H NMR (700 MHz, $CDCl_3$) spectrum of ***(S)*-5a**.

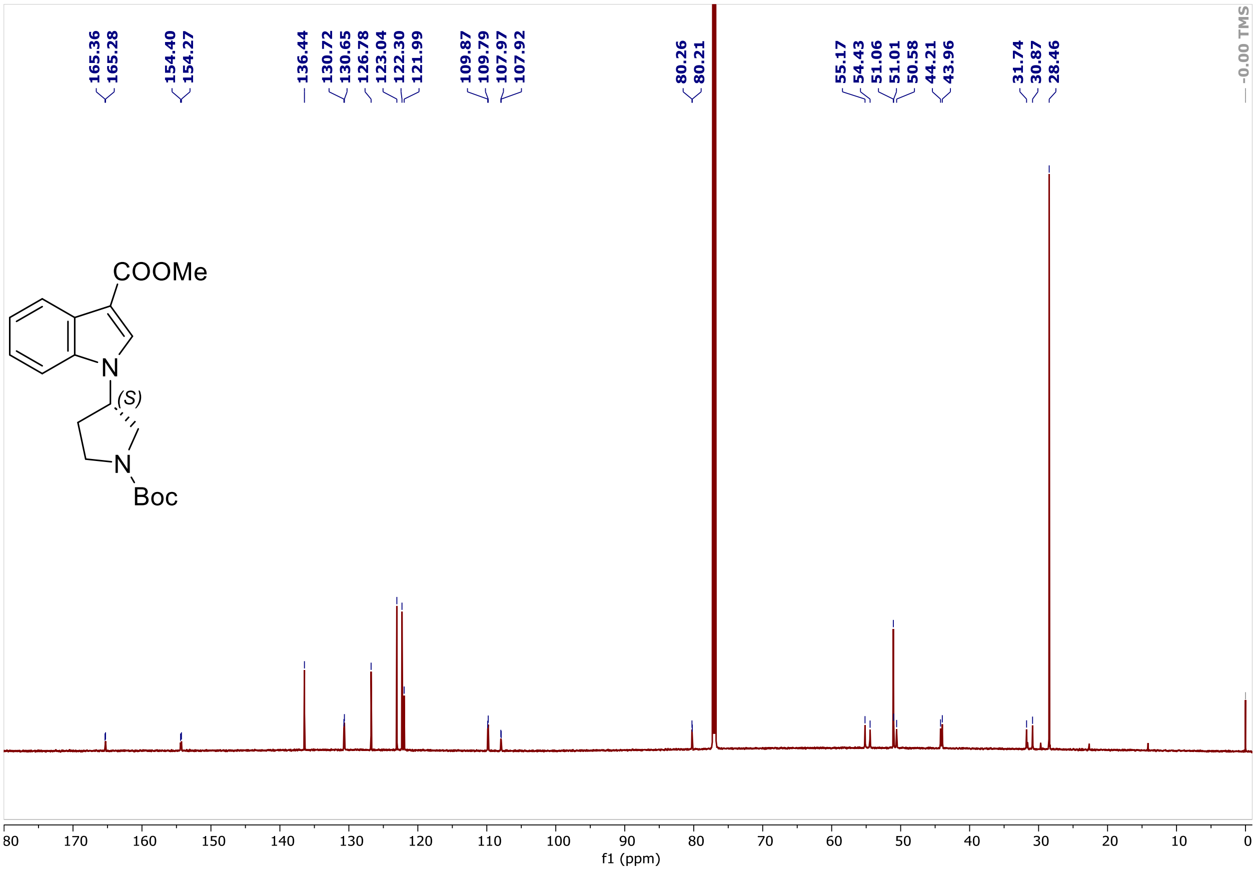

Figure S18. ^{13}C NMR (176 MHz, $CDCl_3$) spectrum of ***(S)*-5a**.

Acquisition Parameter

Source Type	ESI	Ion Polarity	Positive	Set Nebulizer	0.4 Bar
Focus	Not active	Set Capillary	4500 V	Set Dry Heater	180 °C
Scan Begin	50 m/z	Set End Plate Offset	-500 V	Set Dry Gas	4.0 l/min
Scan End	1000 m/z	Set Collision Cell RF	140.0 Vpp	Set Divert Valve	Waste

#	RT [min]	Area	Int. Type	I	S/N	Chromatogram	Max. m/z	FWHM [min]
n.a.	0.1	n.a.	Single spectrum	n.a.	n.a.	n.a.	304.2634	n.a.
n.a.	4.3	n.a.	Single spectrum	n.a.	n.a.	n.a.	367.1630	n.a.

+MS, 4.3min #258

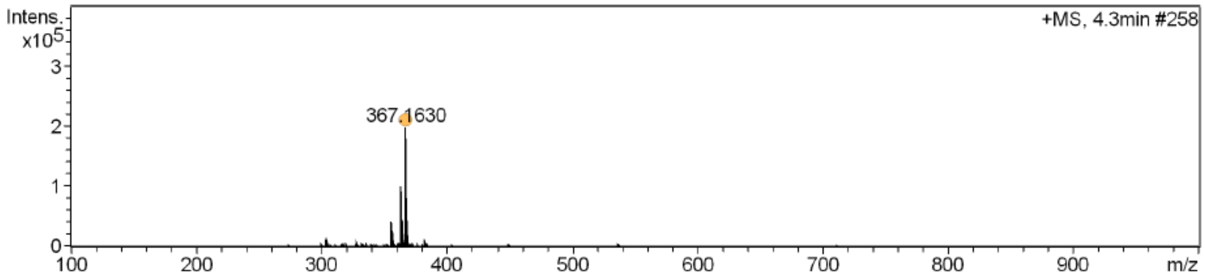

Meas. m/z	#	Ion Formula	m/z	err [ppm]	mSigma	# Sigma	Score	rdb	e⁻ Conf	N-Rule
367.1630	1	C19H24N2NaO4	367.1628	-0.5	13.6	1	100.00	8.5	even	ok

Figure S19. HRMS (ESI^+) report of **(*S*)-5a**.

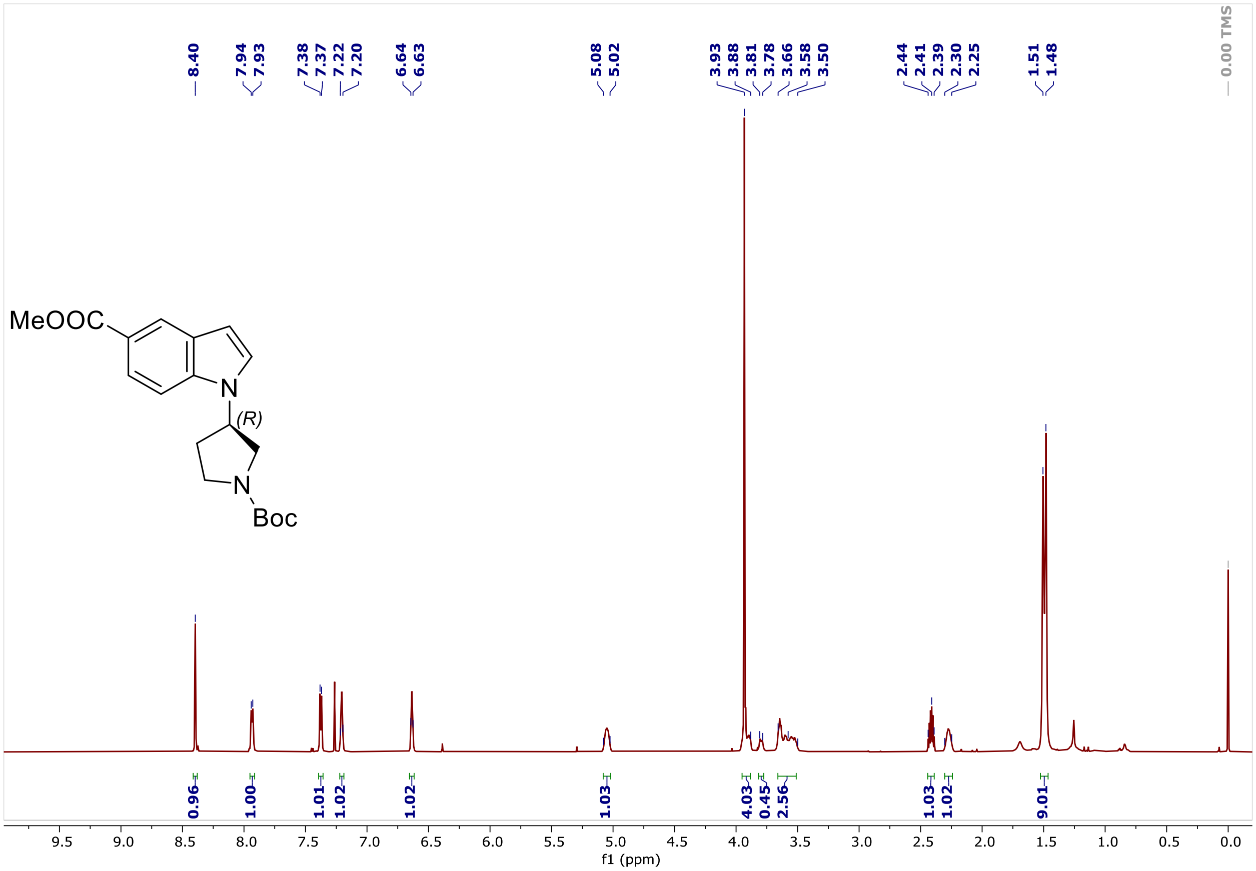

Figure S20. ^{1}H NMR (700 MHz, $CDCl_3$) spectrum of ***(R)*-5b**.

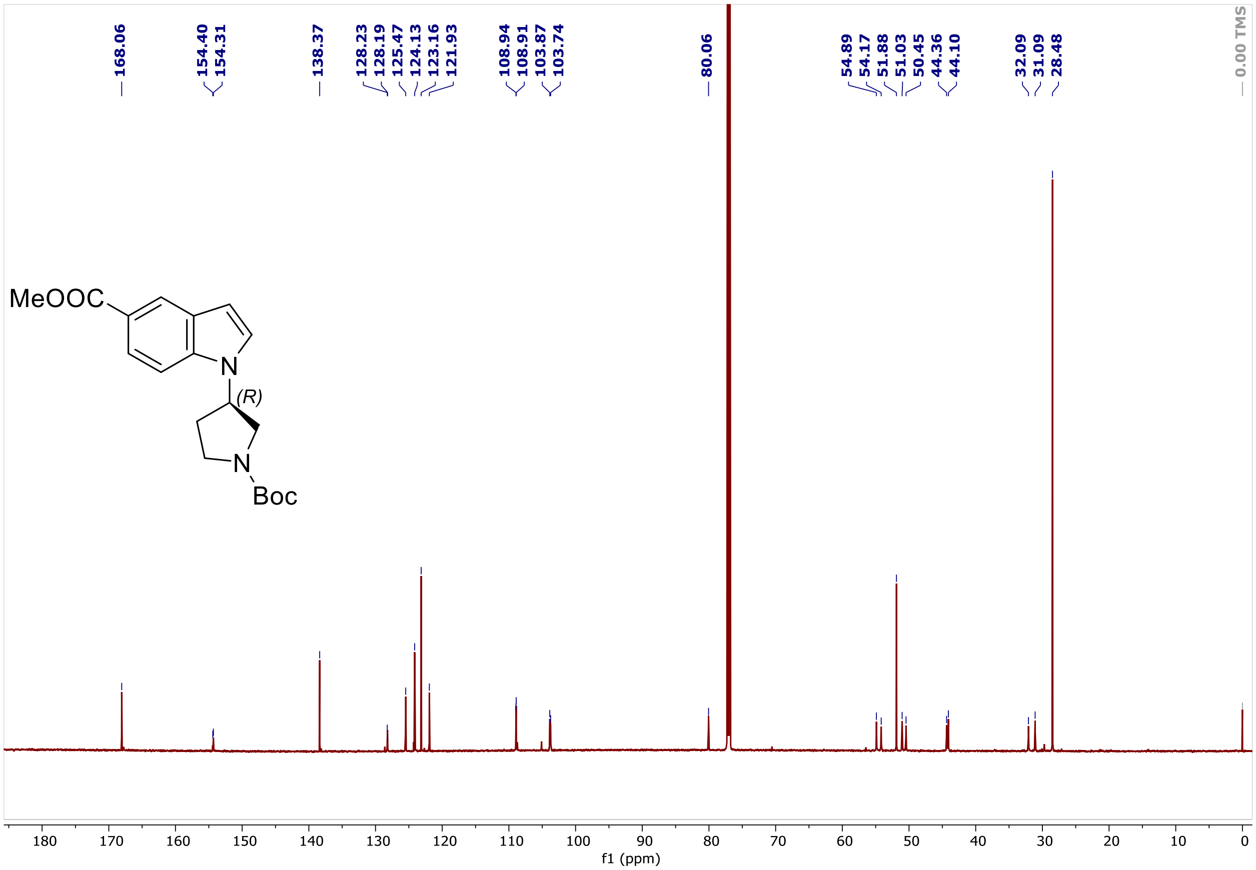

Figure S21. ^{13}C NMR (176 MHz, $CDCl_3$) spectrum of (***R***)-**5b**.

Acquisition Parameter

Source Type	ESI	Ion Polarity	Positive	Set Nebulizer	0.4 Bar
Focus	Not active	Set Capillary	4500 V	Set Dry Heater	180 °C
Scan Begin	50 m/z	Set End Plate Offset	-500 V	Set Dry Gas	4.0 l/min
Scan End	1000 m/z	Set Collision Cell RF	140.0 Vpp	Set Divert Valve	Waste

#	RT [min]	Area	Int. Type	I	S/N	Chromatogram	Max. m/z	FWHM [min]
n.a.	0.5	n.a.	Single spectrum	n.a.	n.a.	n.a.	304.2624	n.a.
n.a.	3.9	n.a.	Single spectrum	n.a.	n.a.	n.a.	367.1628	n.a.

+MS, 3.9min #234

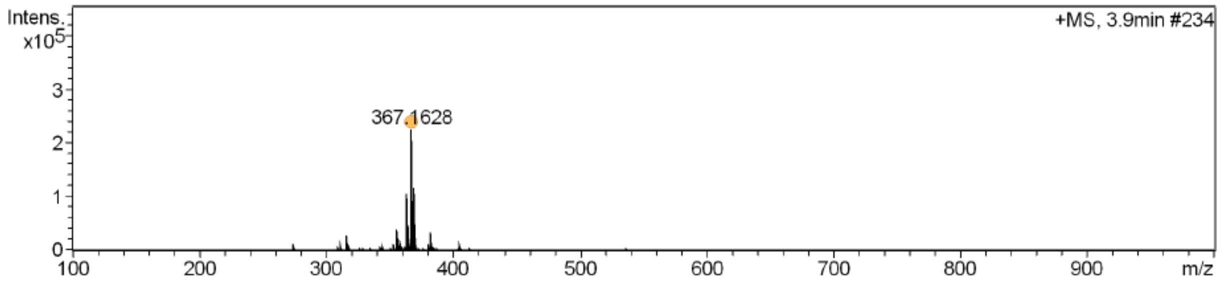

Meas. m/z	#	Ion Formula	m/z	err [ppm]	mSigma	# Sigma	Score	rdb	e¯ Conf	N-Rule
367.1628	1	C19H24N2NaO4	367.1628	0.1	17.7	1	100.00	8.5	even	ok

Figure S22. HRMS (ESI$^+$) report of ***(R)*-5a**.

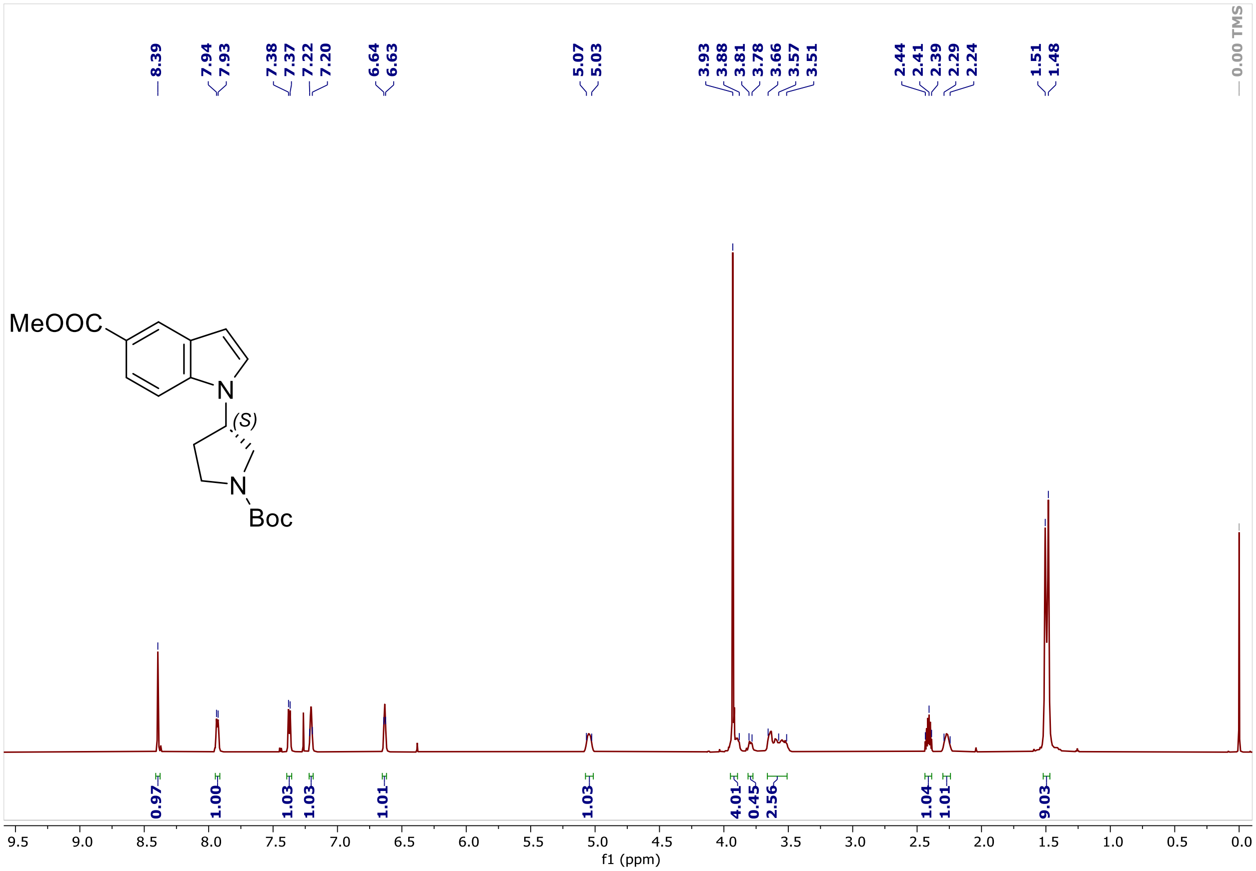

Figure S23. ^{1}H NMR (700 MHz, $CDCl_3$) spectrum of ***(S)*-5b**.

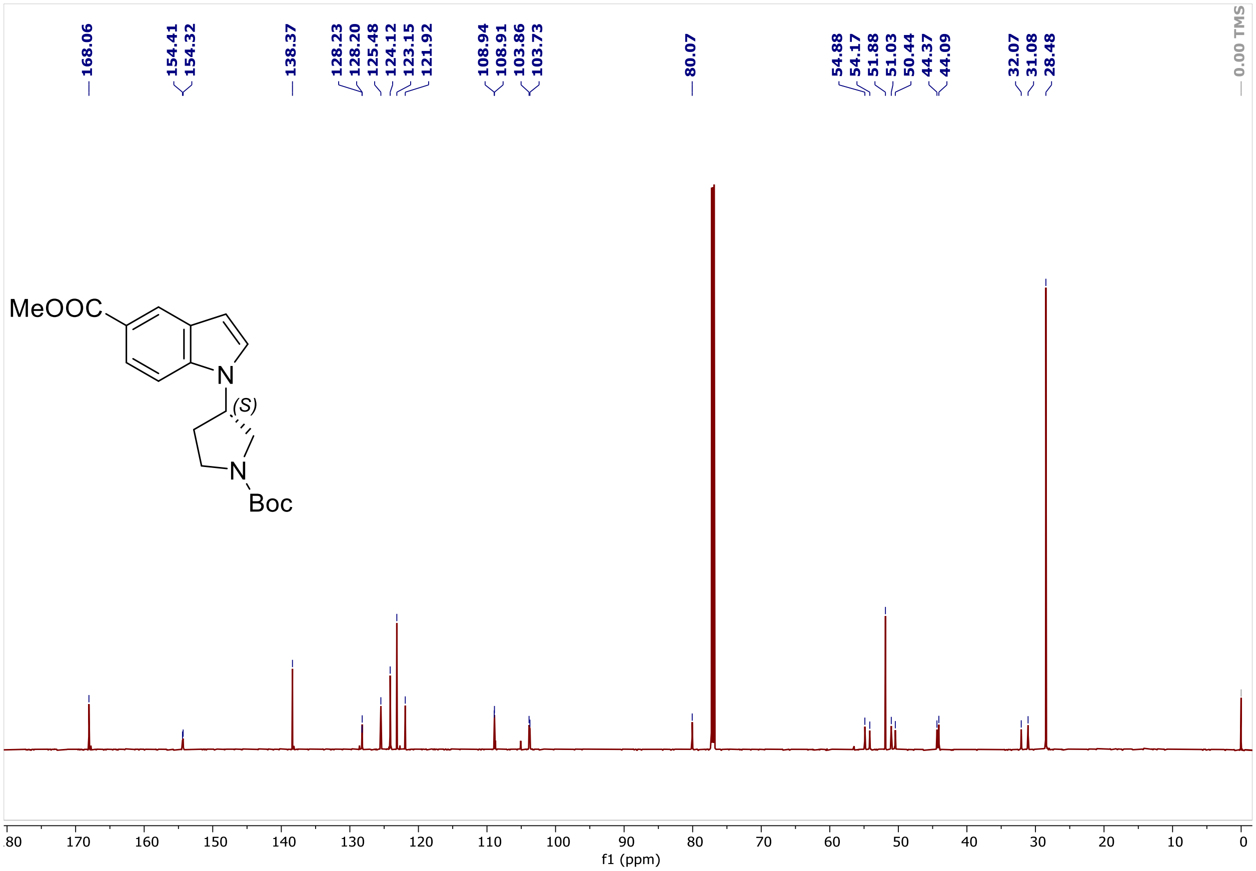

Figure S24. ^{13}C NMR (176 MHz, $CDCl_3$) spectrum of **(*S*)-5b**.

Acquisition Parameter

Source Type	ESI	Ion Polarity	Positive	Set Nebulizer	0.4 Bar
Focus	Not active	Set Capillary	4500 V	Set Dry Heater	180 °C
Scan Begin	50 m/z	Set End Plate Offset	-500 V	Set Dry Gas	4.0 l/min
Scan End	1000 m/z	Set Collision Cell RF	140.0 Vpp	Set Divert Valve	Waste

#	RT [min]	Area	Int. Type	I	S/N	Chromatogram	Max. m/z	FWHM [min]
n.a.	0.2	n.a.	Single spectrum	n.a.	n.a.	n.a.	304.2633	n.a.
n.a.	3.3	n.a.	Single spectrum	n.a.	n.a.	n.a.	367.1627	n.a.

+MS, 3.3min #200

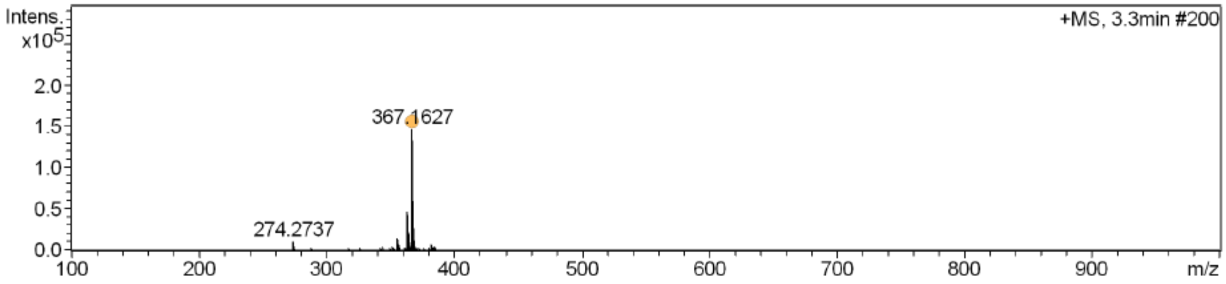

Meas. m/z	#	Ion Formula	m/z	err [ppm]	mSigma	# Sigma	Score	rdb	e¯ Conf	N-Rule
367.1627	1	C19H24N2NaO4	367.1628	0.2	10.4	1	100.00	8.5	even	ok

Figure S25. HRMS (ESI^+) report of ***(S)*-5b**.

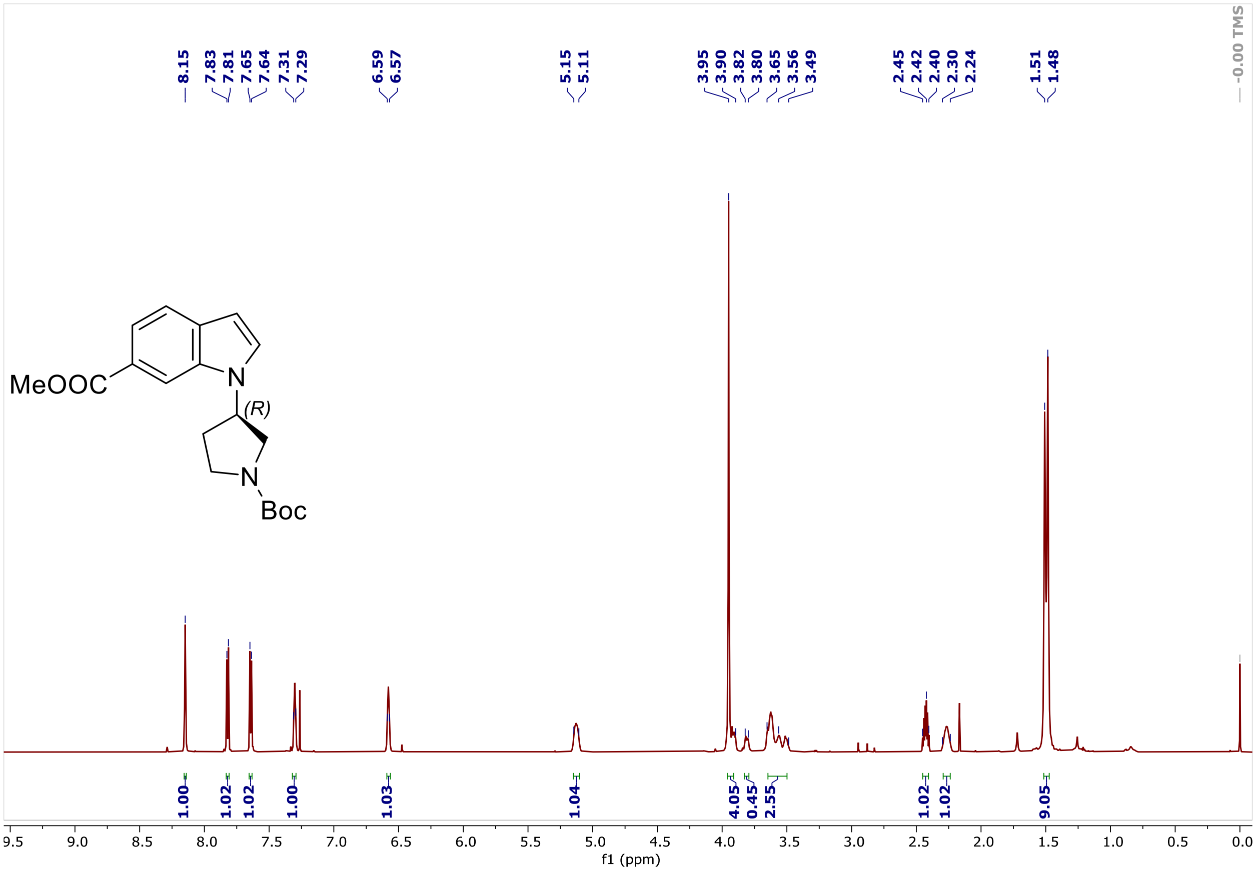

Figure S26. ^{1}H NMR (700 MHz, $CDCl_3$) spectrum of ***(R)*-5c**.

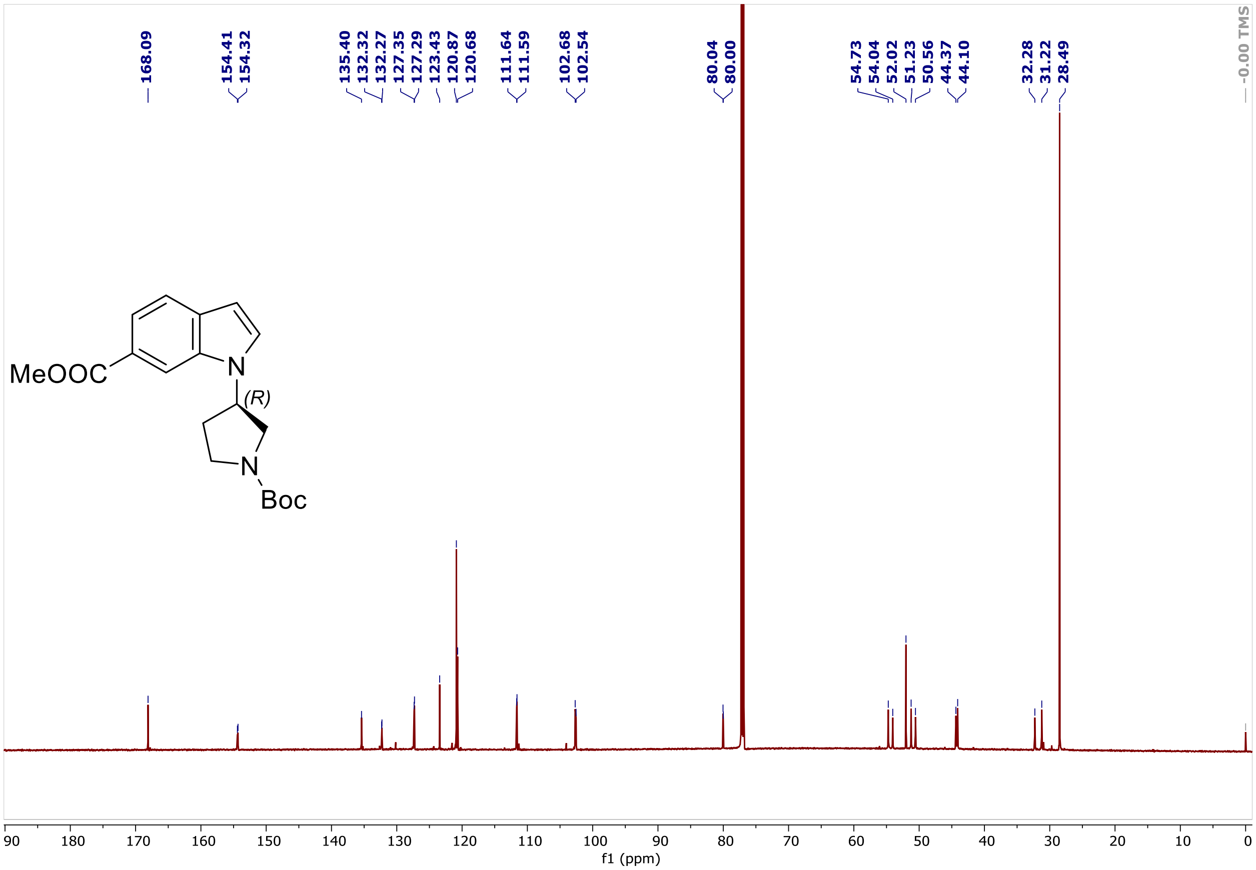

Figure S27. ^{13}C NMR (176 MHz, $CDCl_3$) spectrum of **(*R*)-5c**.

Acquisition Parameter

Source Type	ESI	Ion Polarity	Positive	Set Nebulizer	0.4 Bar
Focus	Not active	Set Capillary	4500 V	Set Dry Heater	180 °C
Scan Begin	50 m/z	Set End Plate Offset	-500 V	Set Dry Gas	4.0 l/min
Scan End	1000 m/z	Set Collision Cell RF	140.0 Vpp	Set Divert Valve	Waste

#	RT [min]	Area	Int. Type	I	S/N	Chromatogram	Max. m/z	FWHM [min]
n.a.	1.0	n.a.	Single spectrum	n.a.	n.a.	n.a.	304.2626	n.a.
n.a.	6.0	n.a.	Single spectrum	n.a.	n.a.	n.a.	367.1628	n.a.

+MS, 6.0min #357

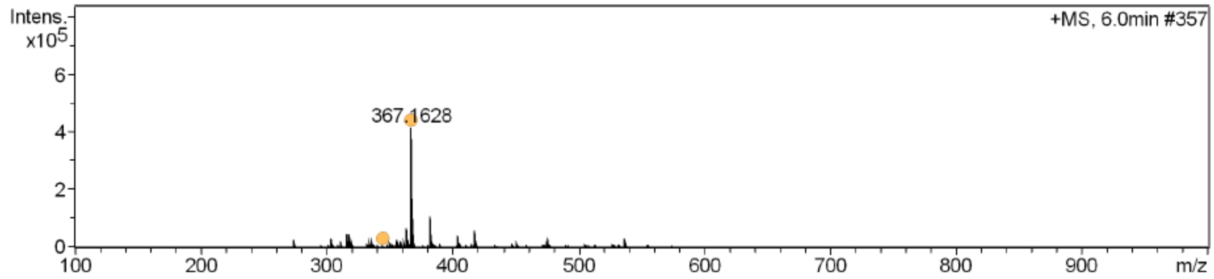

Meas. m/z	#	Ion Formula	m/z	err [ppm]	mSigma	# Sigma	Score	rdb	e¯ Conf	N-Rule
345.1799	1	C19H25N2O4	345.1809	2.8	117.5	1	100.00	8.5	even	ok
367.1628	1	C19H24N2NaO4	367.1628	0.1	23.5	1	100.00	8.5	even	ok

Figure S28. HRMS (ESI^+) report of ***(R)*-5c**.

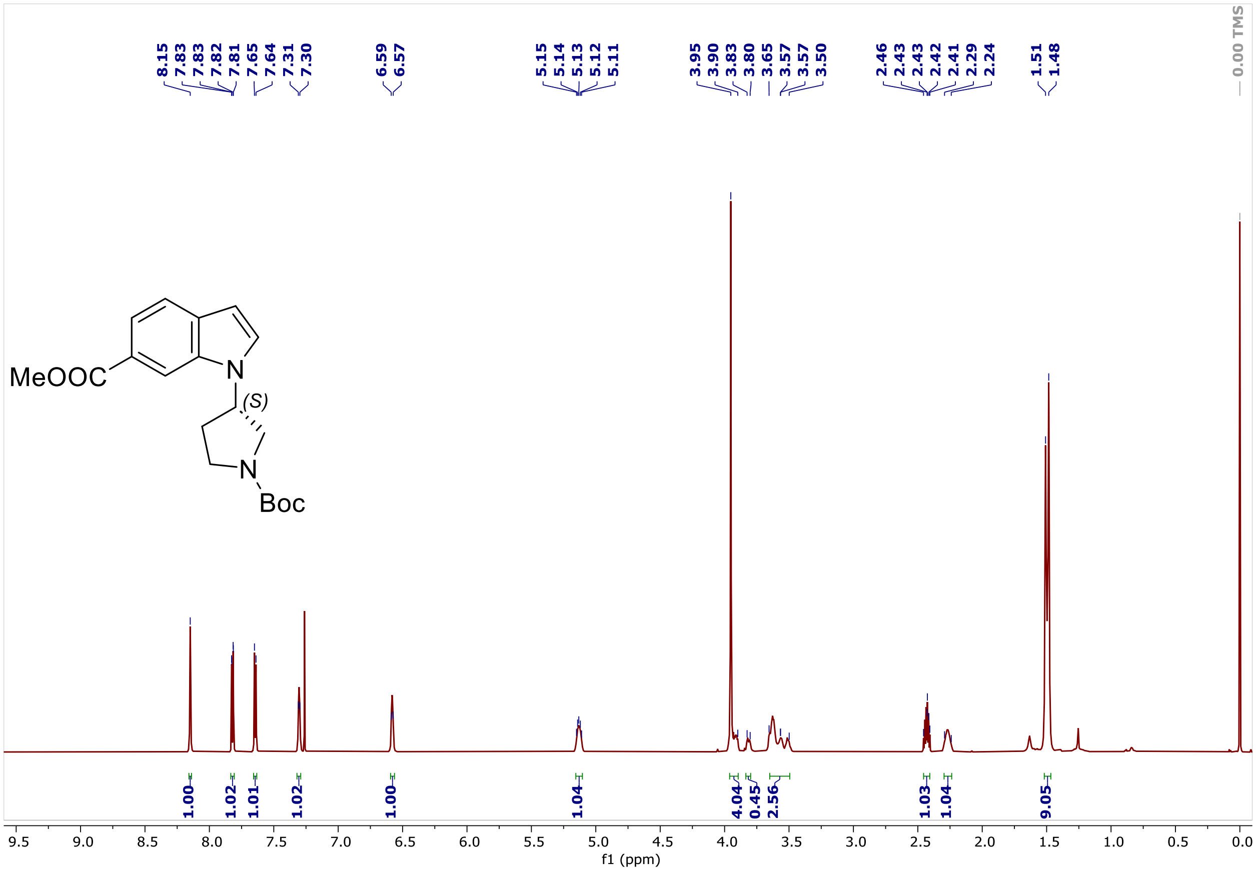

Figure S29. ^{1}H NMR (700 MHz, $CDCl_3$) spectrum of ***(S)*-5c**.

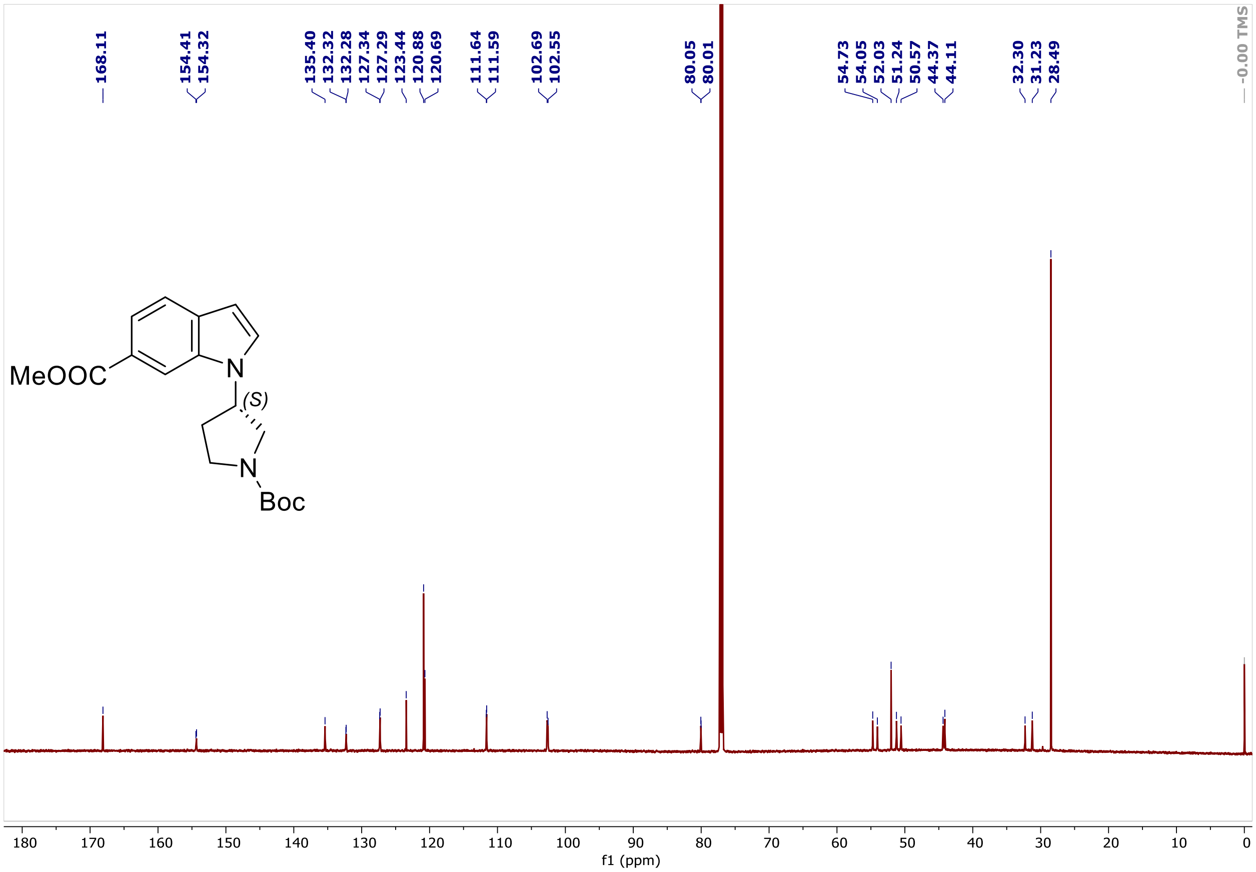

Figure S30. ^{13}C NMR (176 MHz, $CDCl_3$) spectrum of **(*S*)-5c**.

Acquisition Parameter

Source Type	ESI	Ion Polarity	Positive	Set Nebulizer	0.4 Bar
Focus	Not active	Set Capillary	4500 V	Set Dry Heater	180 °C
Scan Begin	50 m/z	Set End Plate Offset	-500 V	Set Dry Gas	4.0 l/min
Scan End	1000 m/z	Set Collision Cell RF	140.0 Vpp	Set Divert Valve	Waste

#	RT [min]	Area	Int. Type	I	S/N	Chromatogram	Max. m/z	FWHM [min]
n.a.	0.1	n.a.	Single spectrum	n.a.	n.a.	n.a.	304.2634	n.a.
n.a.	4.4	n.a.	Single spectrum	n.a.	n.a.	n.a.	367.1623	n.a.

+MS, 4.4min #264

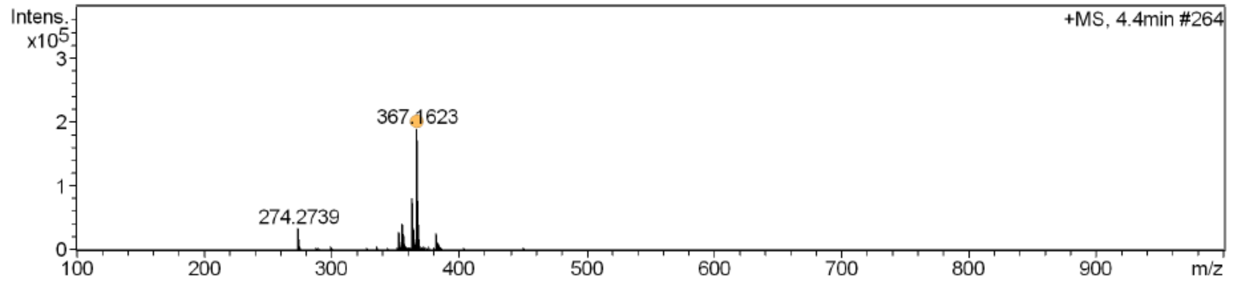

Meas. m/z	#	Ion Formula	m/z	err [ppm]	mSigma	# Sigma	Score	rdb	e¯ Conf	N-Rule
367.1623	1	C19H24N2NaO4	367.1628	1.3	11.6	1	100.00	8.5	even	ok

Figure S31. HRMS (ESI^+) report of ***(S)*-5c**.

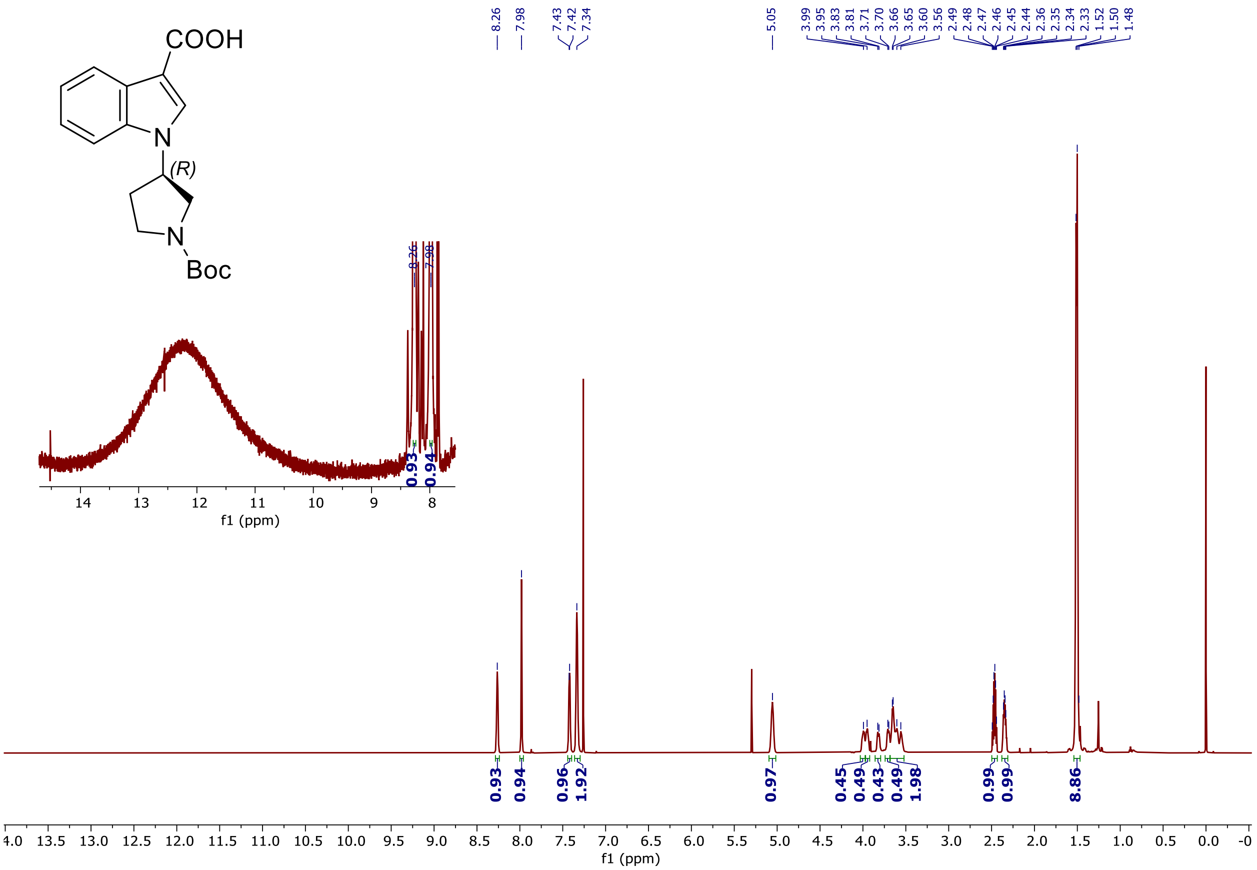

Figure S32. ^{1}H NMR (700 MHz, $CDCl_3$) spectrum of **(*R*)-6**.

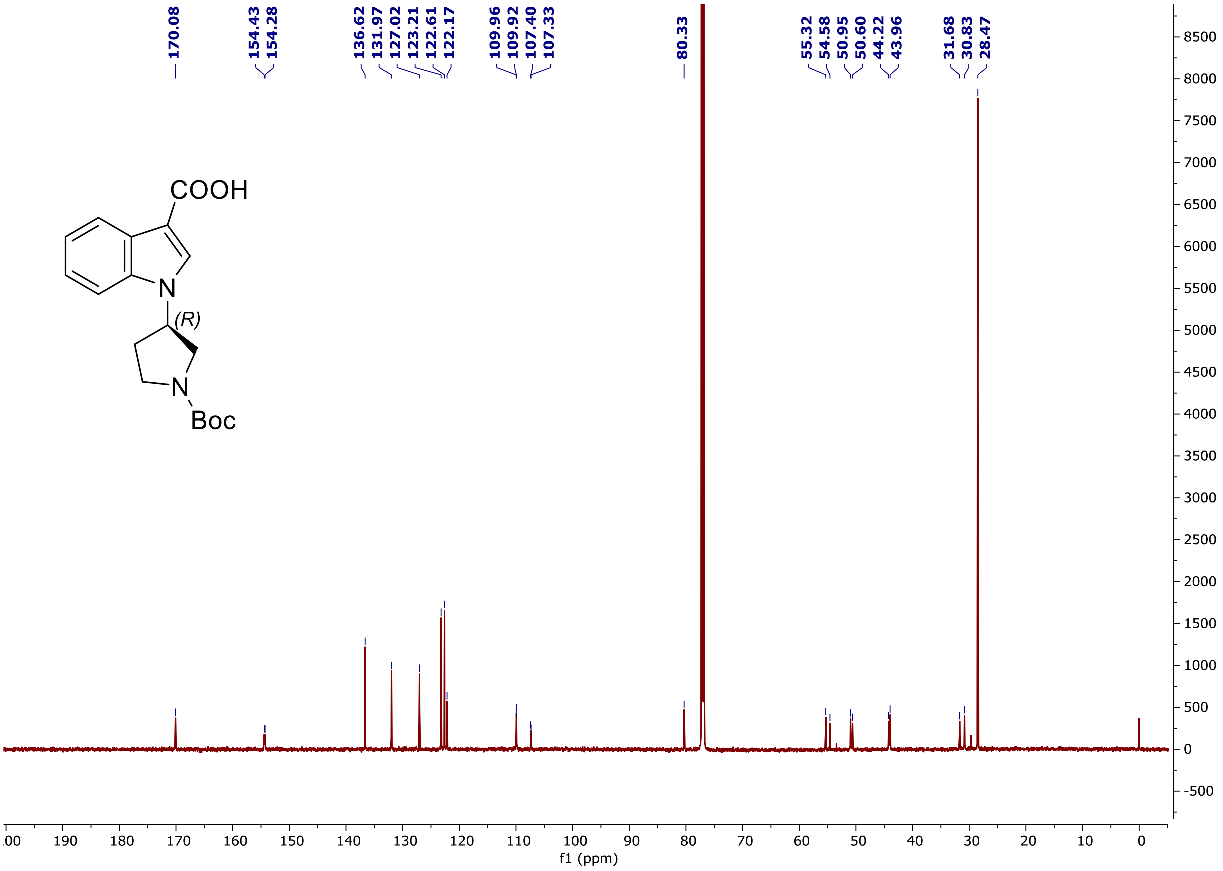

Figure S33. ^{13}C NMR (176 MHz, $CDCl_3$) spectrum of ***(R)*-6**.

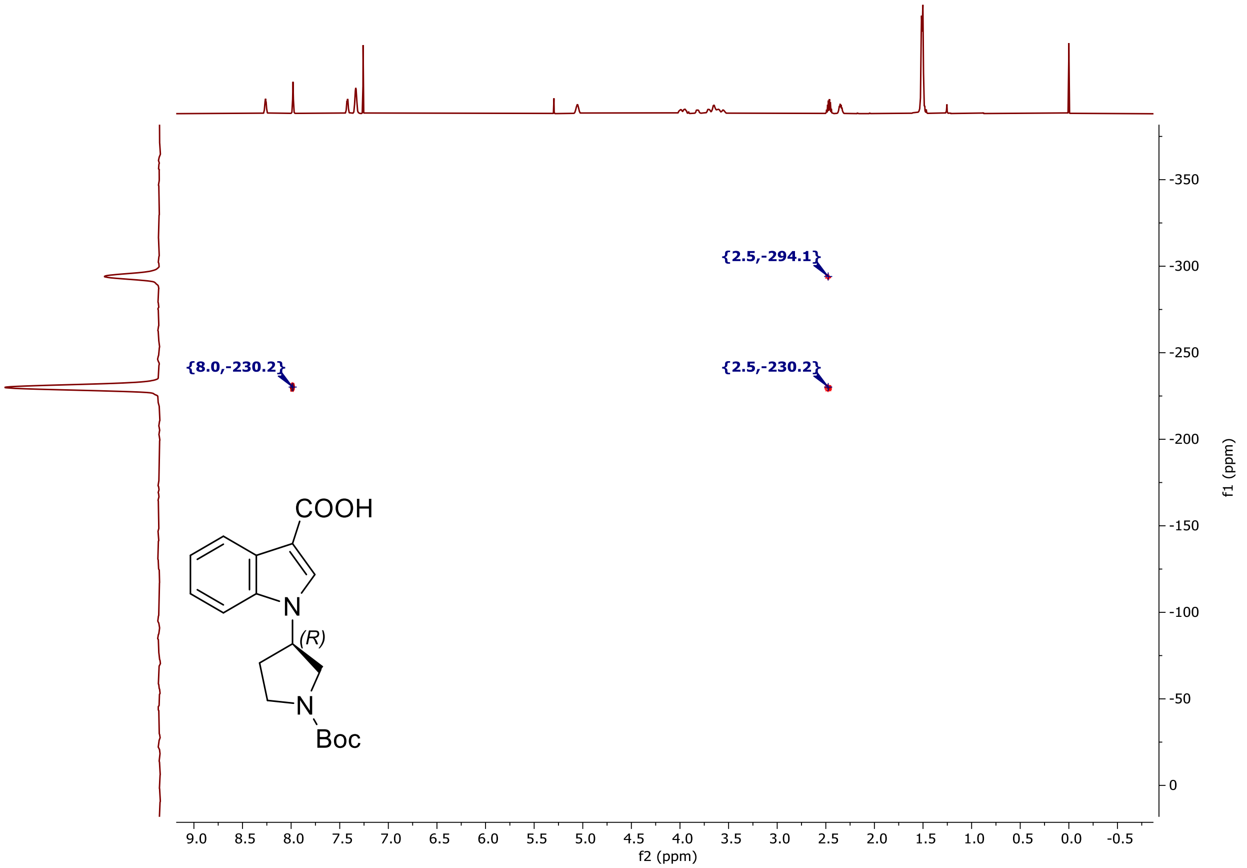

Figure S34. ^{1}H-^{15}N HMBC NMR spectrum of **(*R*)-6** (71 MHz, $CDCl_3$).

Acquisition Parameter

Source Type	ESI	Ion Polarity	Positive	Set Nebulizer	0.4 Bar
Focus	Not active	Set Capillary	4500 V	Set Dry Heater	180 °C
Scan Begin	50 m/z	Set End Plate Offset	-500 V	Set Dry Gas	4.0 l/min
Scan End	1000 m/z	Set Collision Cell RF	140.0 Vpp	Set Divert Valve	Waste

#	RT [min]	Area	Int. Type	I	S/N	Chromatogram	Max. m/z	FWHM [min]
n.a.	1.3	n.a.	Single spectrum	n.a.	n.a.	n.a.	226.9516	n.a.
n.a.	2.2	n.a.	Single spectrum	n.a.	n.a.	n.a.	353.1477	n.a.
n.a.	2.6	n.a.	Single spectrum	n.a.	n.a.	n.a.	353.1475	n.a.
n.a.	6.0	n.a.	Single spectrum	n.a.	n.a.	n.a.	353.1473	n.a.
n.a.	2.0	n.a.	Single spectrum	n.a.	n.a.	n.a.	353.1472	n.a.

+MS, 6.0min #359

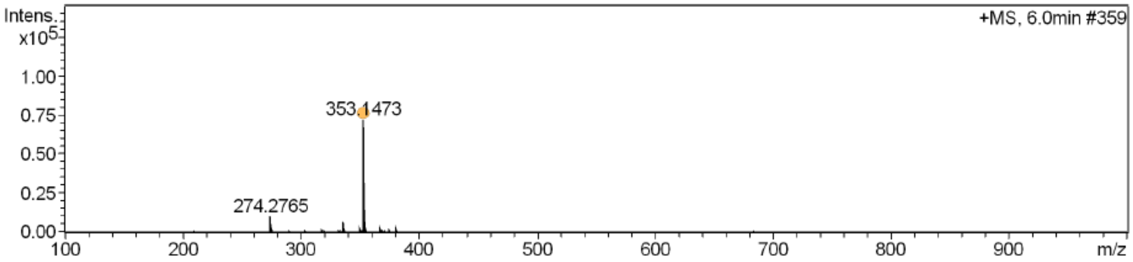

Meas. m/z	#	Ion Formula	m/z	err [ppm]	mSigma	# Sigma	Score	rdb	e⁻ Conf	N-Rule
353.1473	1	C18H22N2NaO4	353.1472	0.4	5.3	1	100.00	8.5	even	ok

Figure S35. HRMS (ESI$^+$) report of ***(R)*-6**.

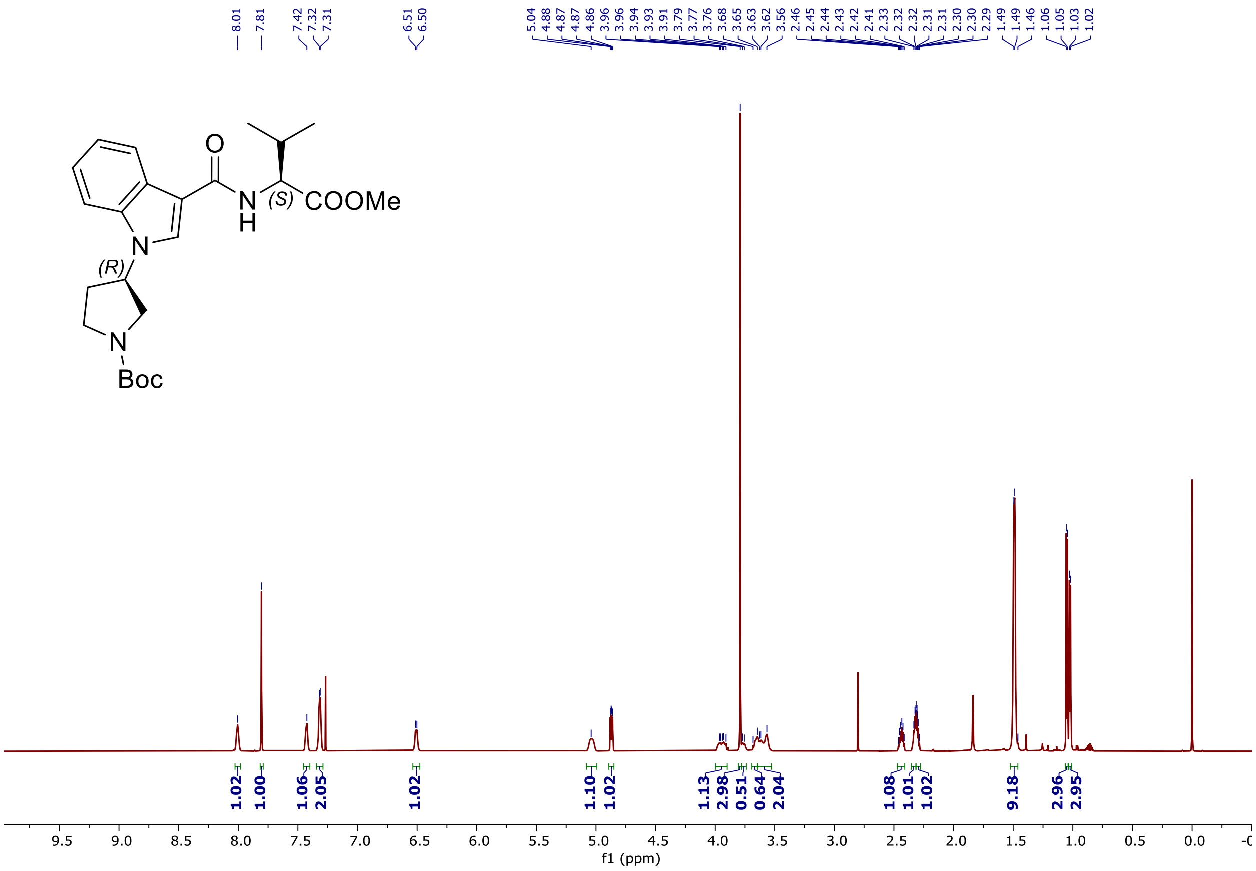

Figure S36. ^{1}H NMR (700 MHz, $CDCl_3$) spectrum of ***(R,S)*-7**.

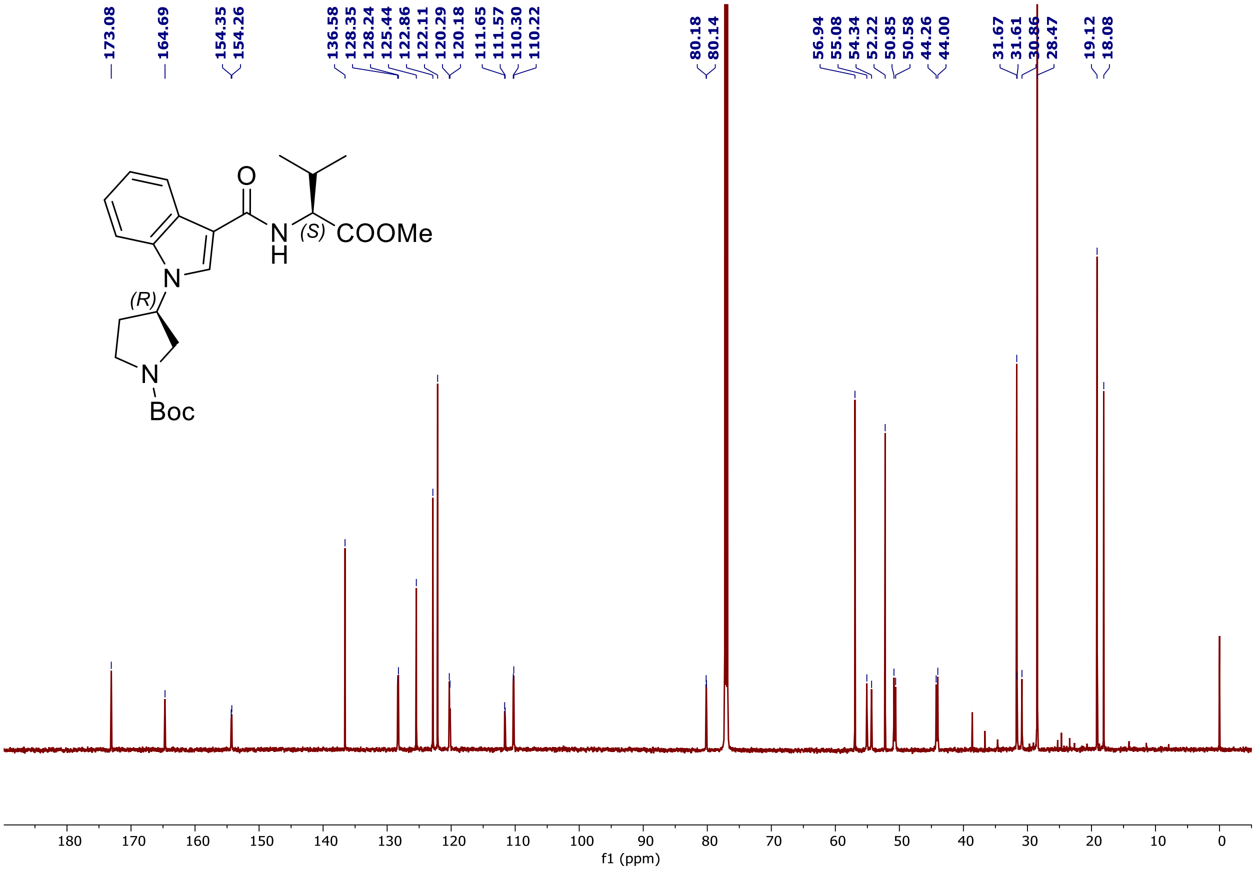

Figure S37. ^{13}C NMR (176 MHz, $CDCl_3$) spectrum of ***(R,S)*-7**.

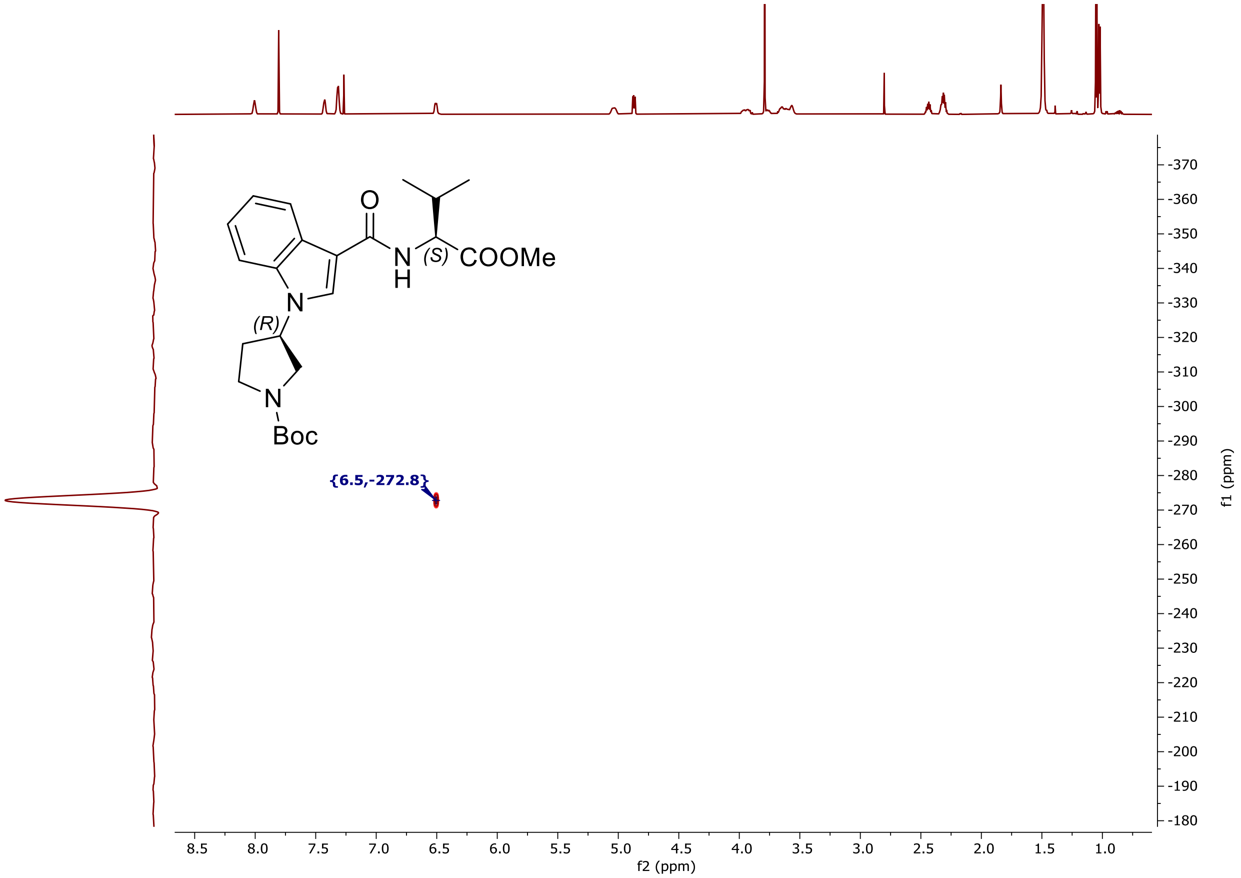

Figure S38. ^{1}H-^{15}N HSQC NMR spectrum of ***(R,S)*-7** (71 MHz, $CDCl_3$).

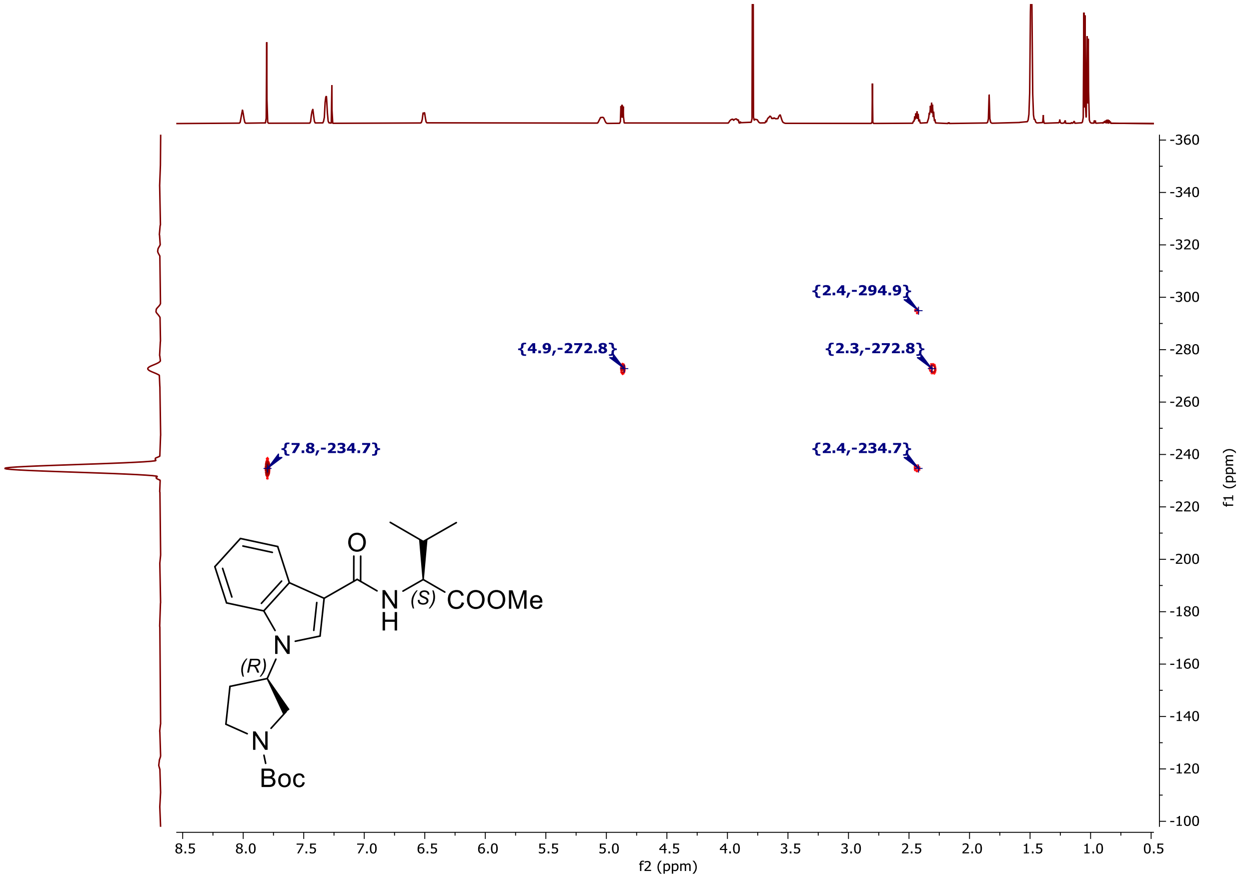

Figure S39. ^{1}H-^{15}N HMBC NMR spectrum of ***(R,S)*-7** (71 MHz, $CDCl_3$).

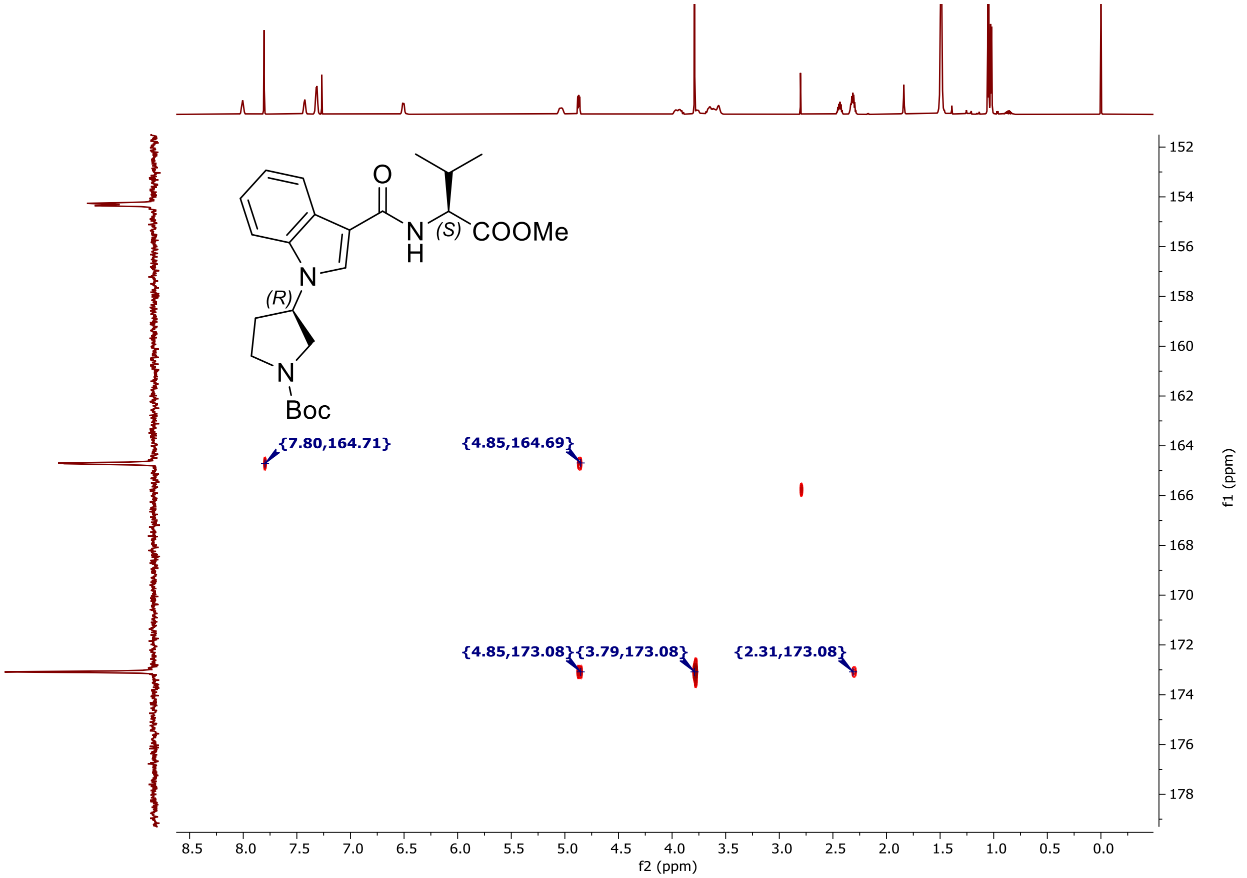

Figure S40. The 8Hz optimized ^{1}H-^{13}C HMBC NMR spectrum of ***(R,S)*-7** ($CDCl_3$).

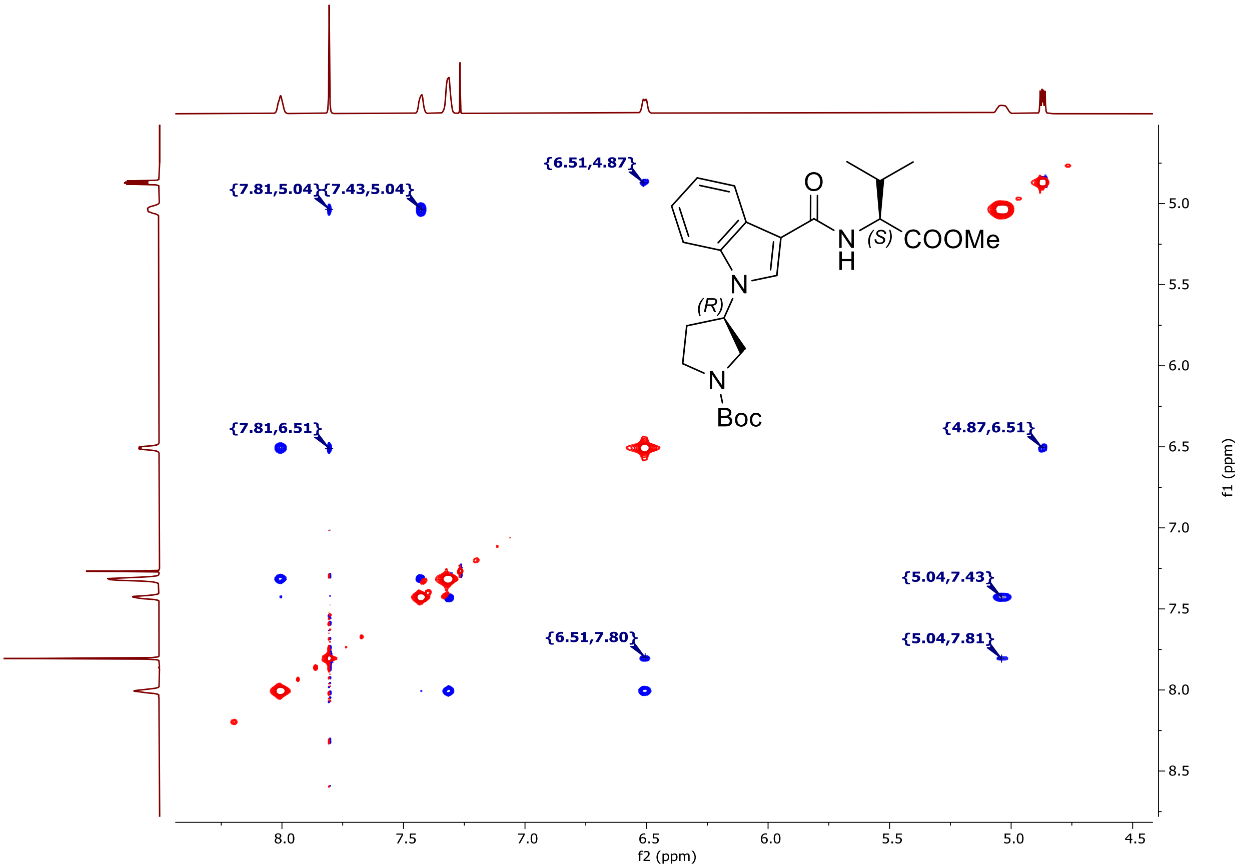

Figure S41. ^{1}H-^{1}H NOESY NMR spectrum of ***(R,S)*-7** ($CDCl_3$).

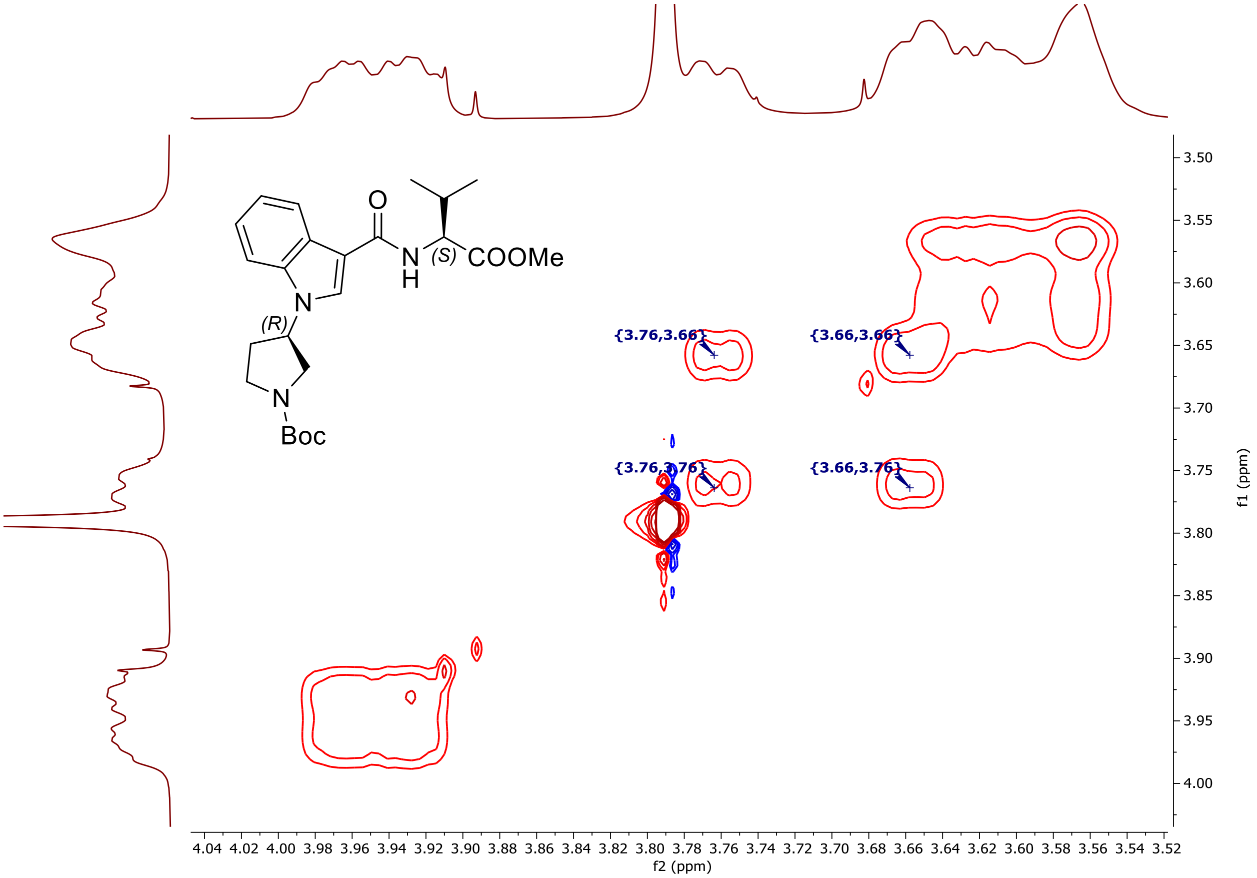

Figure S42. ^{1}H-^{1}H NOESY/EXSY NMR spectrum of ***(R,S)*-7** (CDCl$_3$).

Acquisition Parameter

Source Type	ESI	Ion Polarity	Positive	Set Nebulizer	0.4 Bar
Focus	Not active	Set Capillary	4500 V	Set Dry Heater	180 °C
Scan Begin	50 m/z	Set End Plate Offset	-500 V	Set Dry Gas	4.0 l/min
Scan End	1000 m/z	Set Collision Cell RF	140.0 Vpp	Set Divert Valve	Waste

#	RT [min]	Area	Int. Type	I	S/N	Chromatogram	Max. m/z	FWHM [min]
n.a.	1.6	n.a.	Single spectrum	n.a.	n.a.	n.a.	226.9517	n.a.
n.a.	3.2	n.a.	Single spectrum	n.a.	n.a.	n.a.	466.2326	n.a.
n.a.	3.4	n.a.	Single spectrum	n.a.	n.a.	n.a.	466.2325	n.a.
n.a.	3.1	n.a.	Single spectrum	n.a.	n.a.	n.a.	466.2318	n.a.

+MS, 3.1min #186

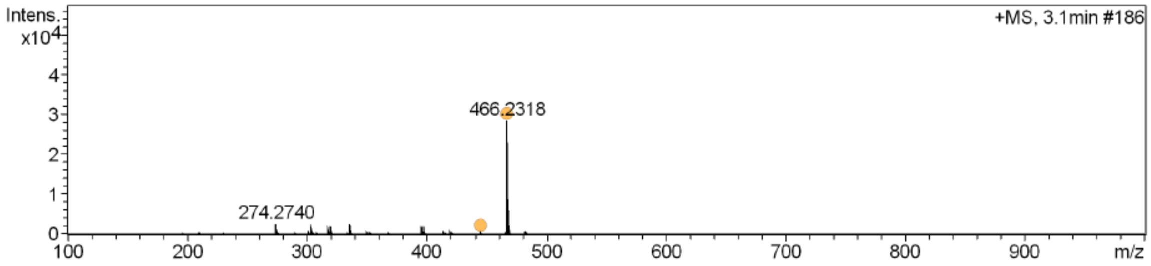

Meas. m/z	#	Ion Formula	m/z	err [ppm]	mSigma	# Sigma	Score	rdb	e⁻ Conf	N-Rule
444.2496	1	C24H34N3O5	444.2493	-0.8	27.1	1	100.00	9.5	even	ok
466.2318	1	C24H33N3NaO5	466.2312	1.2	8.8	1	100.00	9.5	even	ok

Figure S43. HRMS (ESI^+) report of ***(R,S)*-7**.

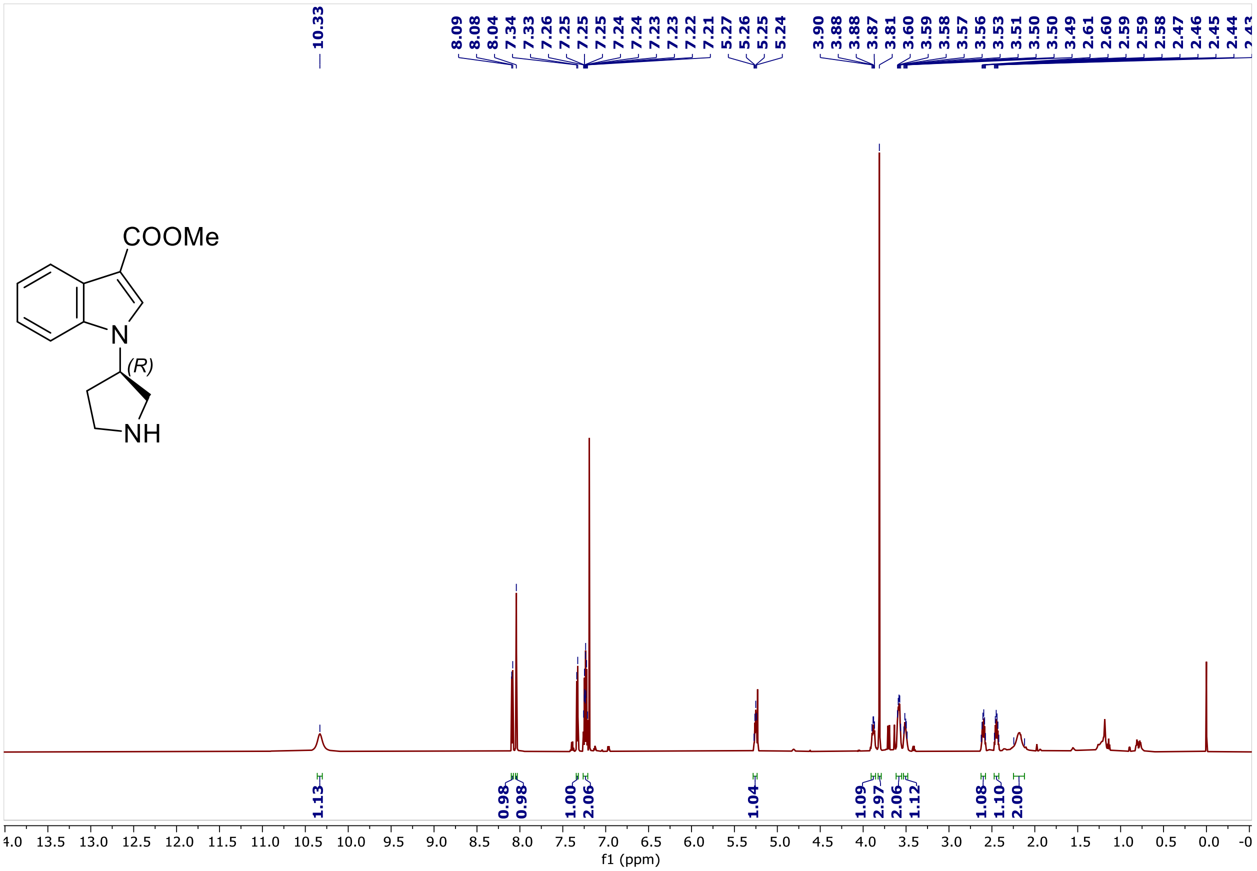

Figure S44. ^{1}H NMR (700 MHz, $CDCl_3$) spectrum of ***(R)*-8**.

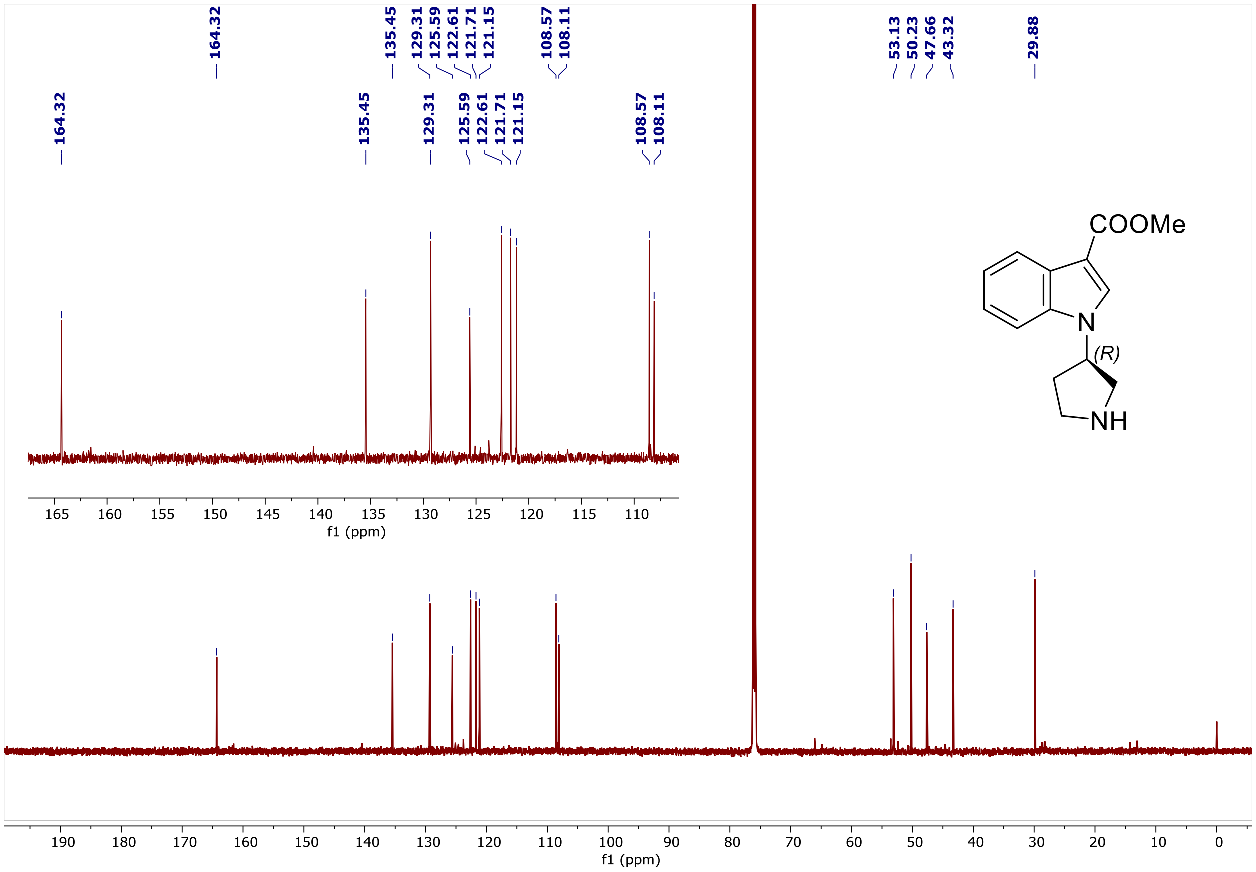

Figure S45. ^{13}C NMR (176 MHz, $CDCl_3$) spectrum of ***(R)*-8**.

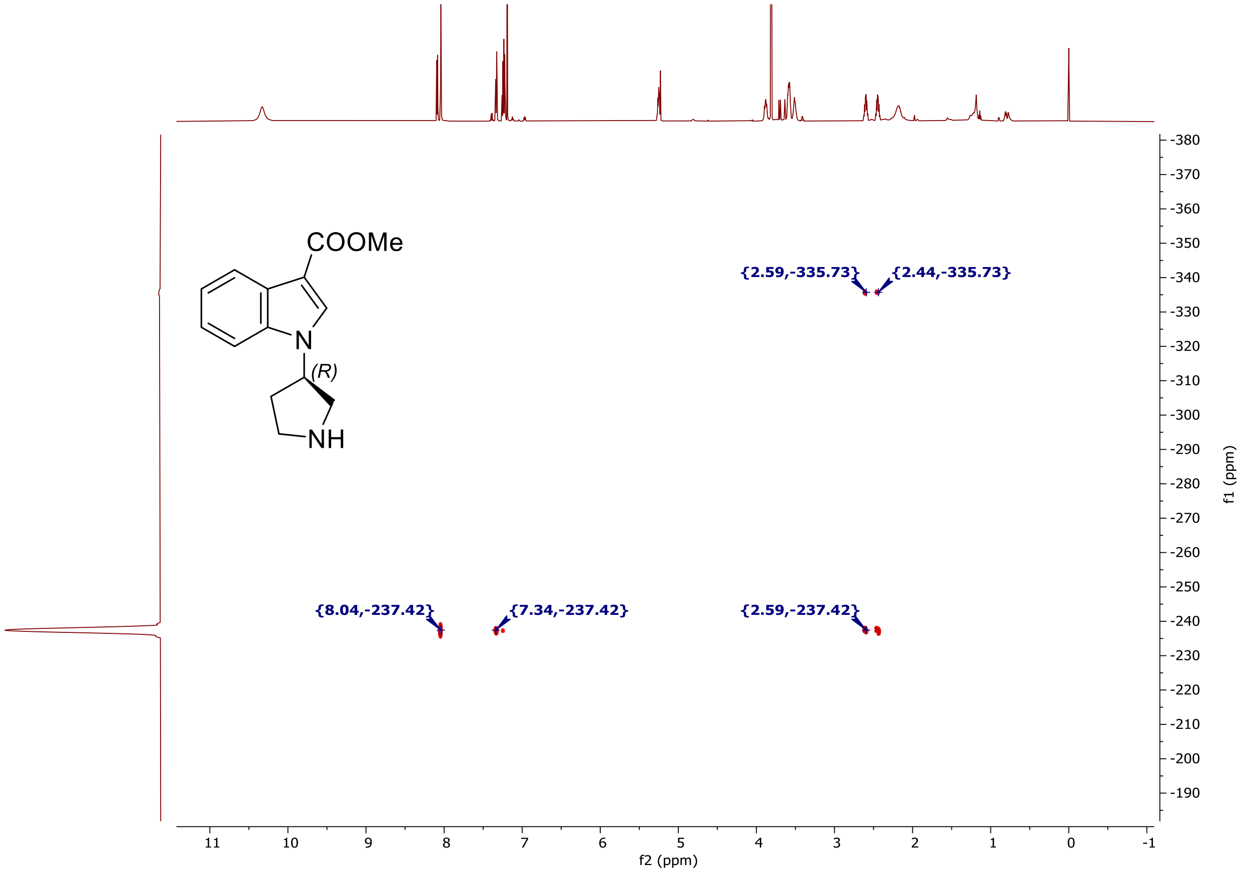

Figure S46. ^1H-^{15}N HMBC NMR spectrum of ***(R)*-8** (71 MHz, $CDCl_3$).

Acquisition Parameter

Source Type	ESI	Ion Polarity	Positive	Set Nebulizer	0.4 Bar
Focus	Not active	Set Capillary	4500 V	Set Dry Heater	180 °C
Scan Begin	50 m/z	Set End Plate Offset	-500 V	Set Dry Gas	4.0 l/min
Scan End	1000 m/z	Set Collision Cell RF	140.0 Vpp	Set Divert Valve	Waste

#	RT [min]	Area	Int. Type	I	S/N	Chromatogram	Max. m/z	FWHM [min]
n.a.	5.9	n.a.	Single spectrum	n.a.	n.a.	n.a.	245.1287	n.a.

+MS, 5.9min #356

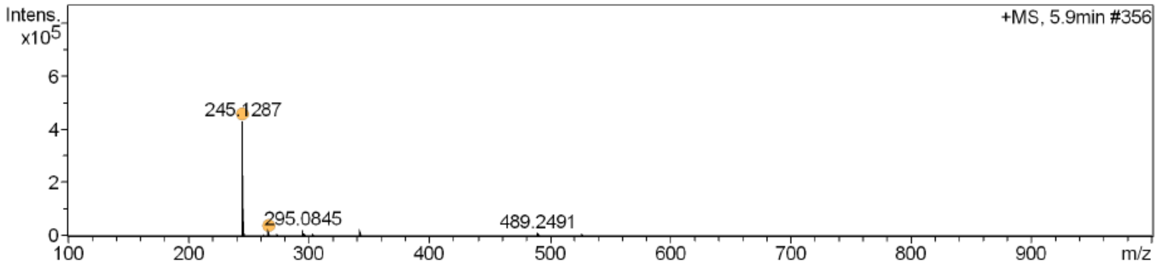

Meas. m/z	#	Ion Formula	m/z	err [ppm]	mSigma	# Sigma	Score	rdb	e⁻ Conf	N-Rule
245.1287	1	C14H17N2O2	245.1285	1.2	44.2	1	100.00	7.5	even	ok
267.1104	1	C14H16N2NaO2	267.1104	-0.0	14.3	1	100.00	7.5	even	ok

Figure S47. HRMS (ESI^+) report of ***(R)*-8**.

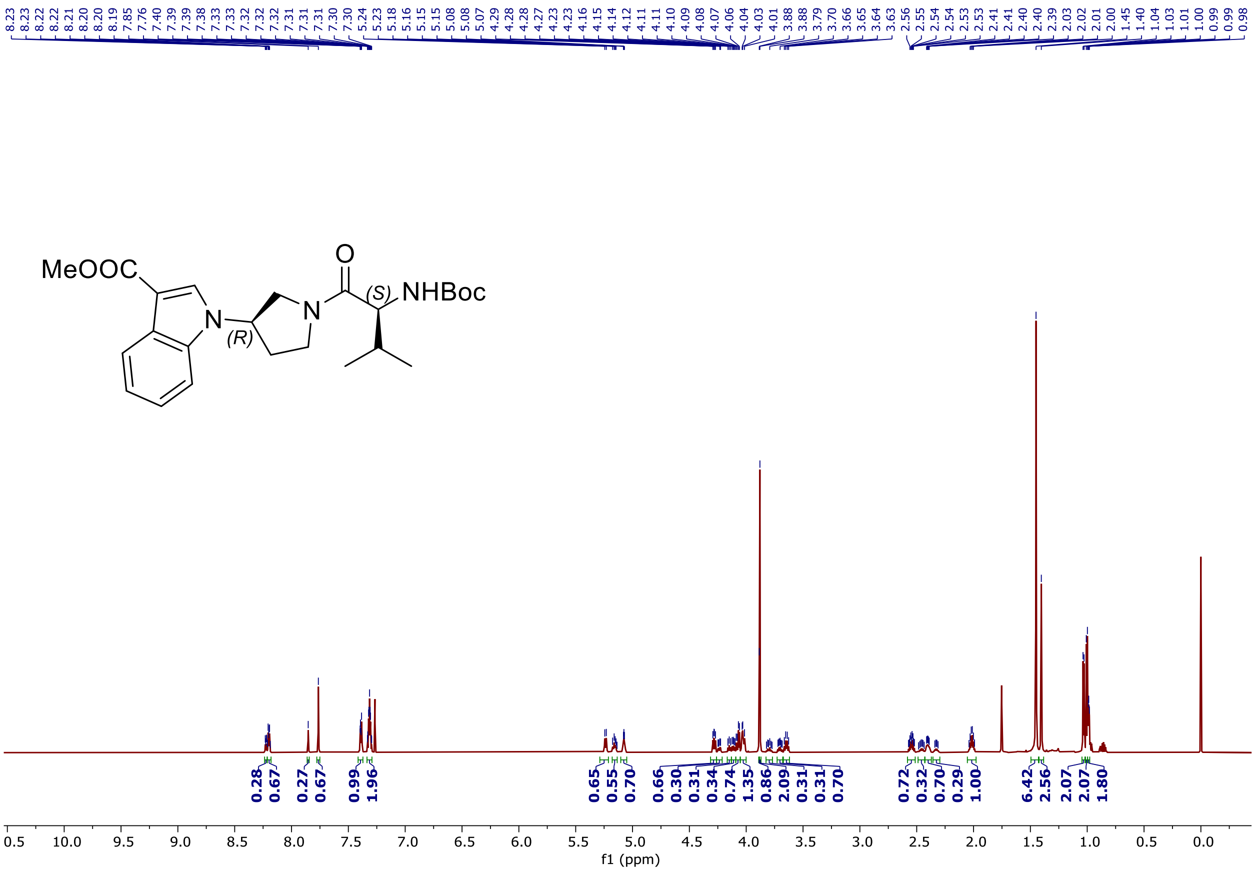

Figure S48. ^{1}H NMR (700 MHz, $CDCl_3$) spectrum of ***(R,S)*-9**.

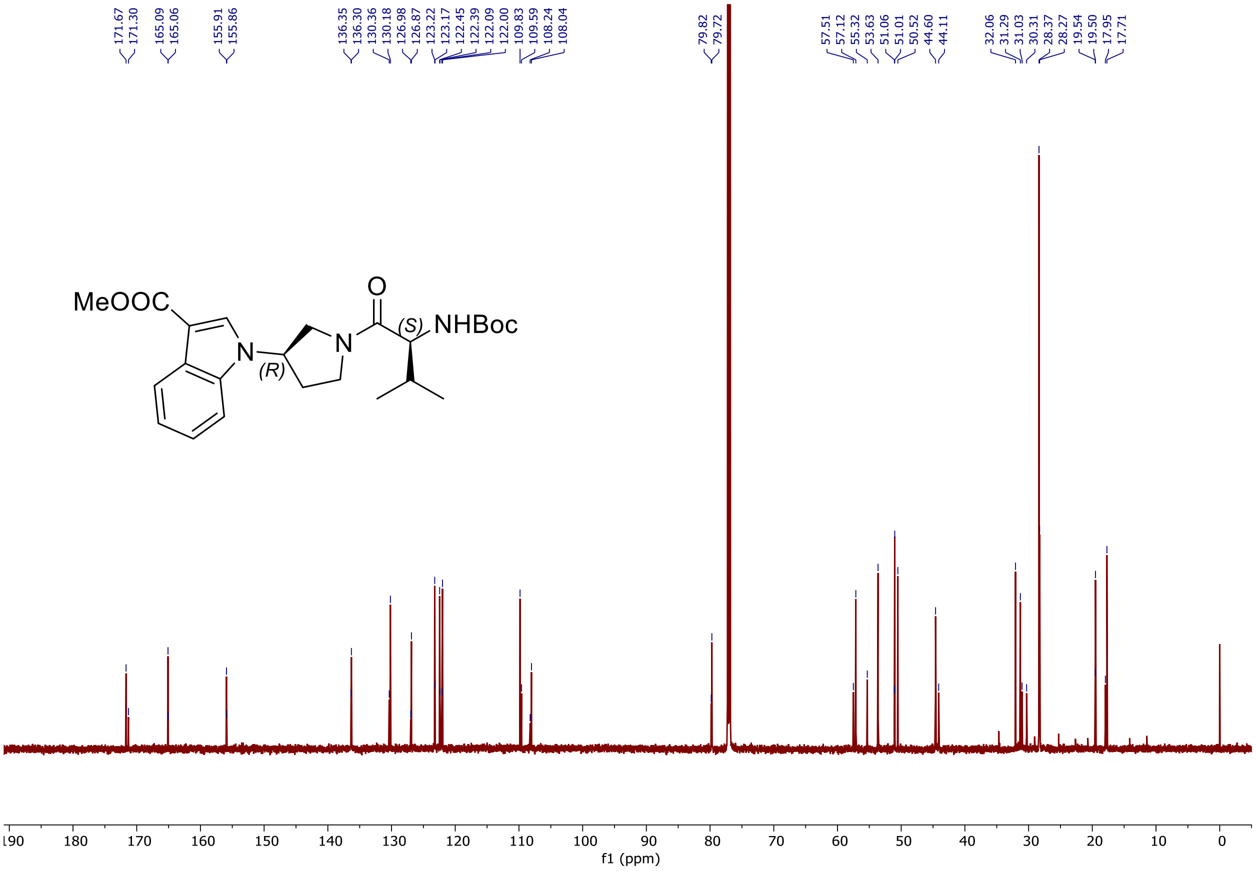

Figure S49. ^{13}C NMR (176 MHz, $CDCl_3$) spectrum of ***(R,S)*-9**.

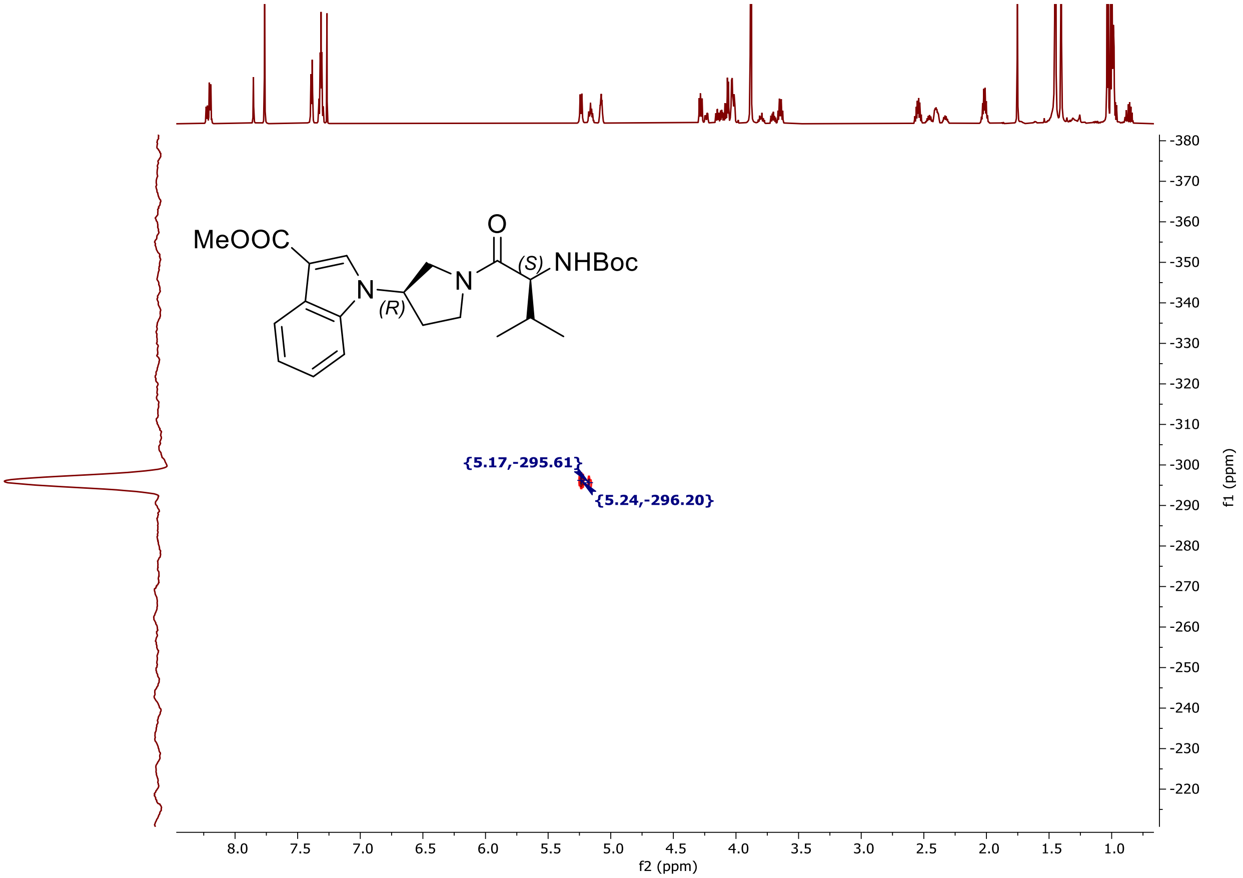

Figure S50. ¹H-¹⁵N HSQC NMR spectrum of ***(R,S)*-9** (71 MHz, $CDCl_3$).

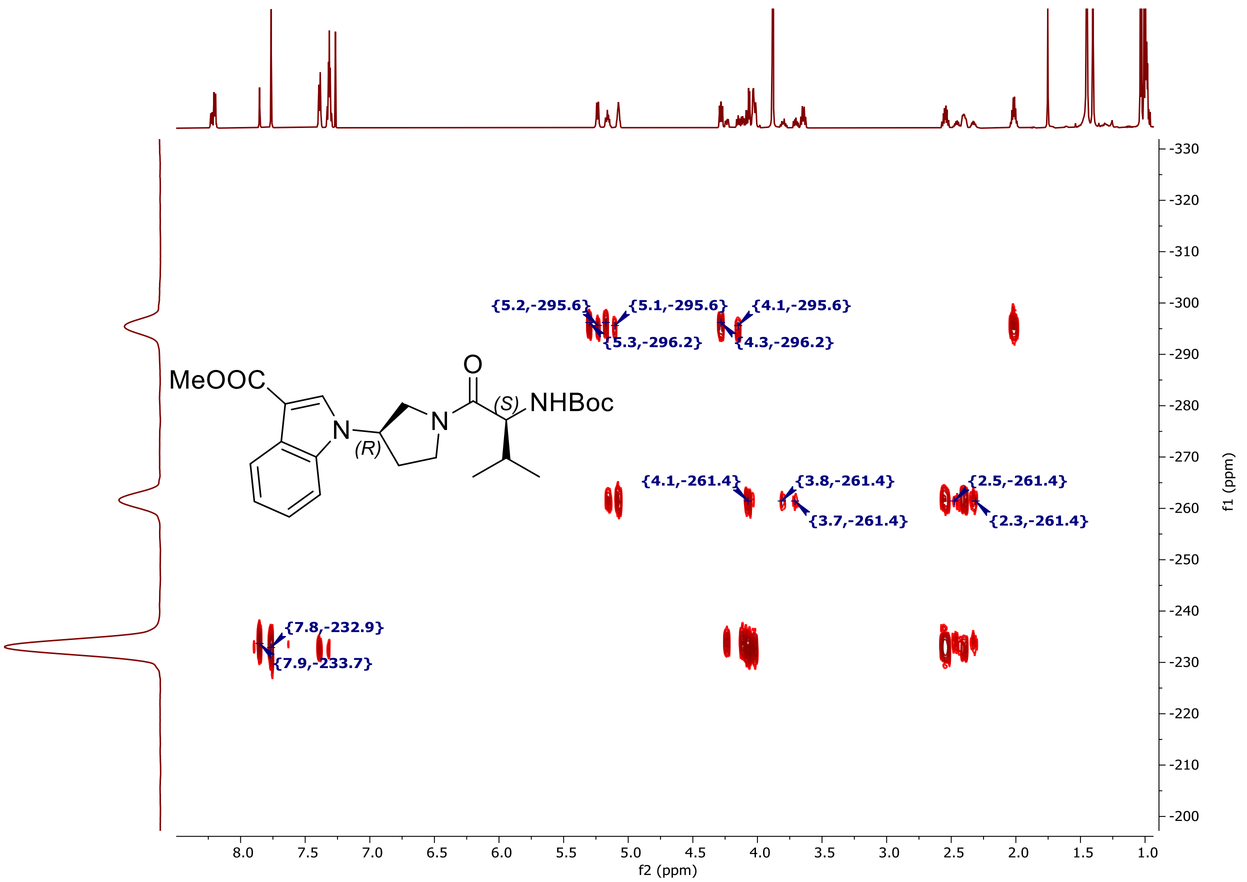

Figure S51. ^{1}H-^{15}N HMBC NMR spectrum of ***(R,S)*-9** (71 MHz, $CDCl_3$).

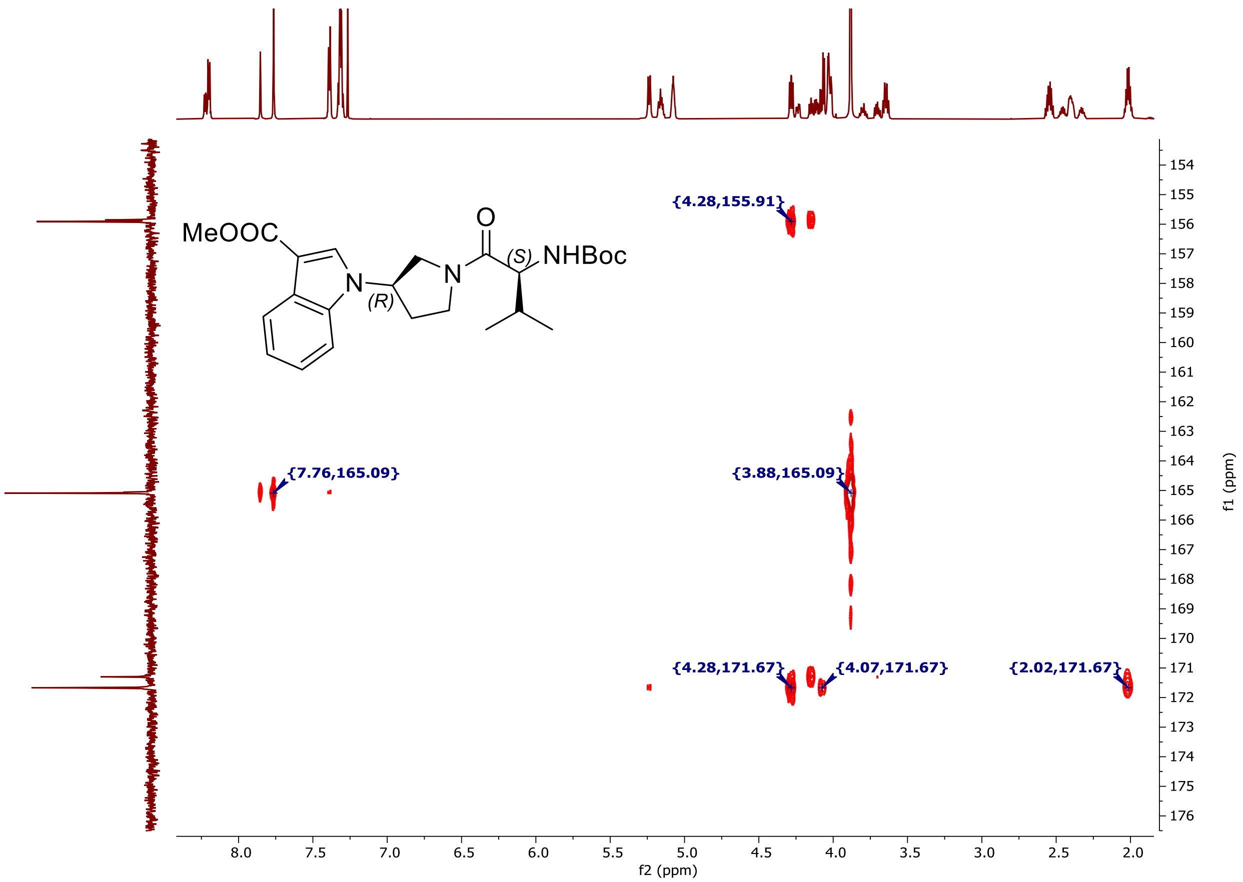

Figure S52. The 8Hz optimized ^{1}H-^{13}C HMBC NMR spectrum of ***(R,S)*-9** ($CDCl_3$).

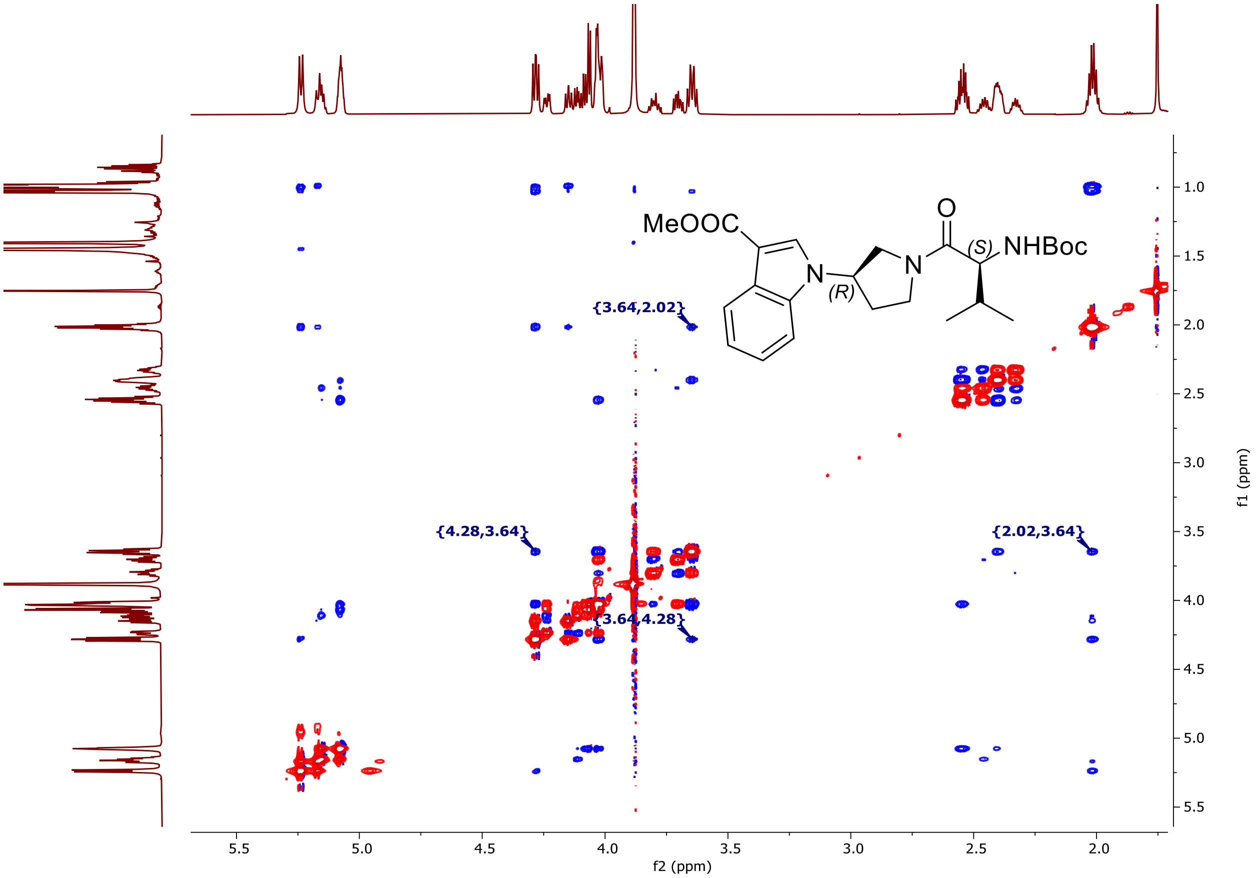

Figure S53. ^{1}H-^{1}H NOESY NMR spectrum of ***(R,S)*-9** ($CDCl_3$).

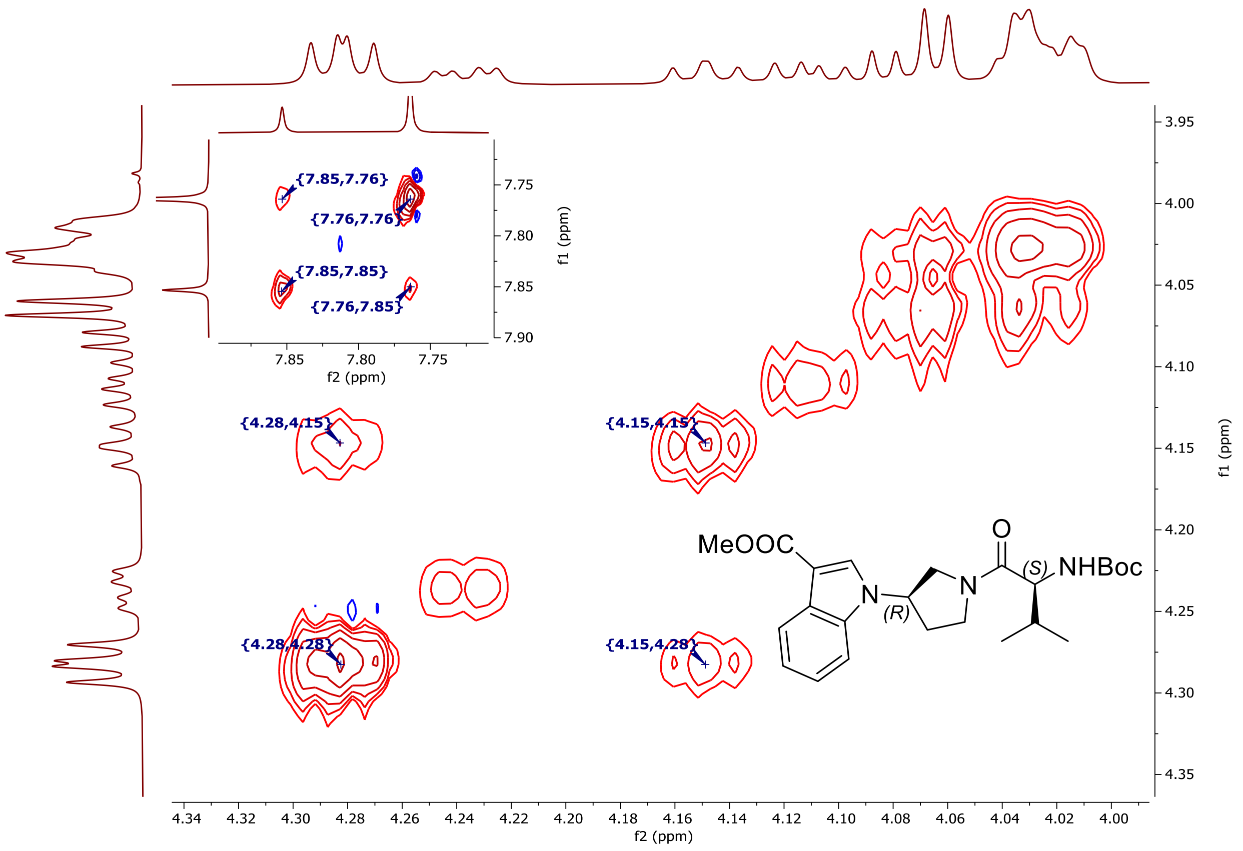

Figure S54. ^{1}H-^{1}H NOESY/EXSY NMR spectrum of ***(R,S)*-9** ($CDCl_3$).

Acquisition Parameter

Source Type	ESI	Ion Polarity	Positive	Set Nebulizer	0.4 Bar
Focus	Not active	Set Capillary	4500 V	Set Dry Heater	180 °C
Scan Begin	50 m/z	Set End Plate Offset	-500 V	Set Dry Gas	4.0 l/min
Scan End	1000 m/z	Set Collision Cell RF	140.0 Vpp	Set Divert Valve	Waste

#	RT [min]	Area	Int. Type	I	S/N	Chromatogram	Max. m/z	FWHM [min]
n.a.	1.8	n.a.	Single spectrum	n.a.	n.a.	n.a.	226.9516	n.a.
n.a.	2.2	n.a.	Single spectrum	n.a.	n.a.	n.a.	466.2313	n.a.
n.a.	2.8	n.a.	Single spectrum	n.a.	n.a.	n.a.	466.2319	n.a.
n.a.	5.8	n.a.	Single spectrum	n.a.	n.a.	n.a.	466.2319	n.a.

+MS, 2.2min #129

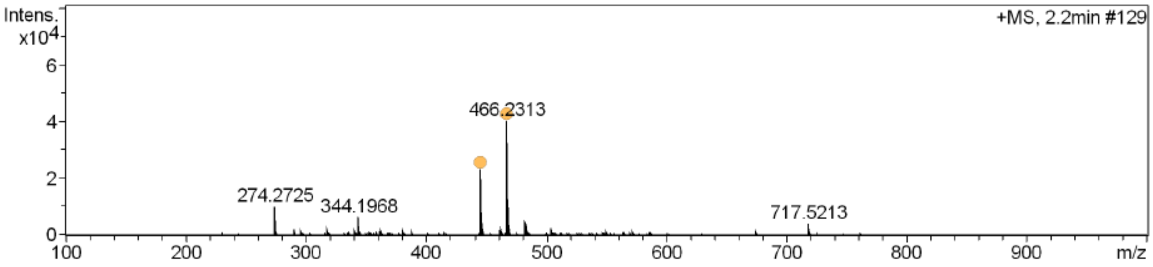

Meas. m/z	#	Ion Formula	m/z	err [ppm]	mSigma	# Sigma	Score	rdb	e⁻ Conf	N-Rule
444.2510	1	C24H34N3O5	444.2493	3.9	30.7	1	100.00	9.5	even	ok
466.2313	1	C24H33N3NaO5	466.2312	0.0	15.2	1	100.00	9.5	even	ok

Figure S55. HRMS (ESI$^+$) report of ***(R,S)*-9**.

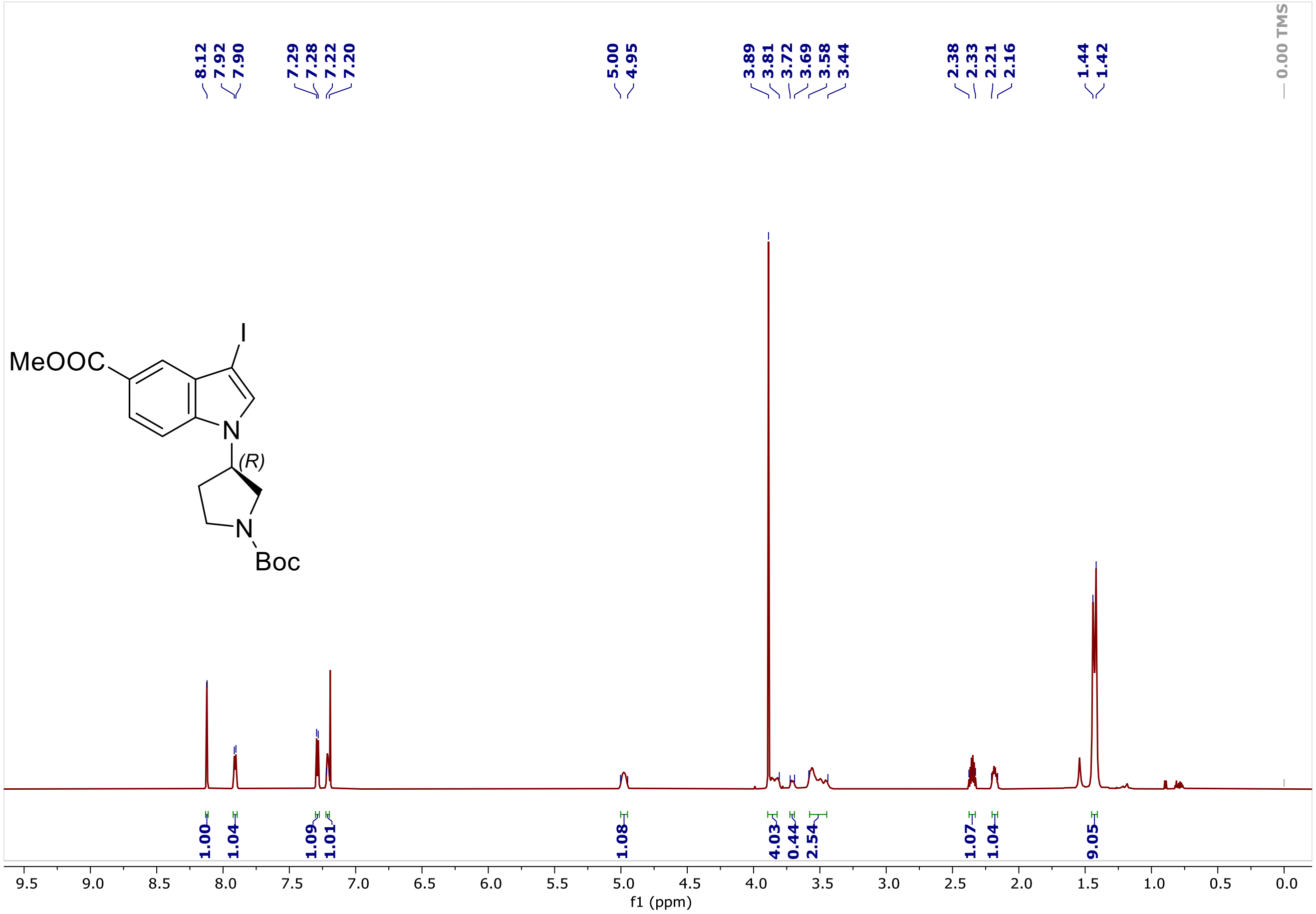

Figure S56. ^{1}H NMR (700 MHz, $CDCl_3$) spectrum of ***(R)*-10**.

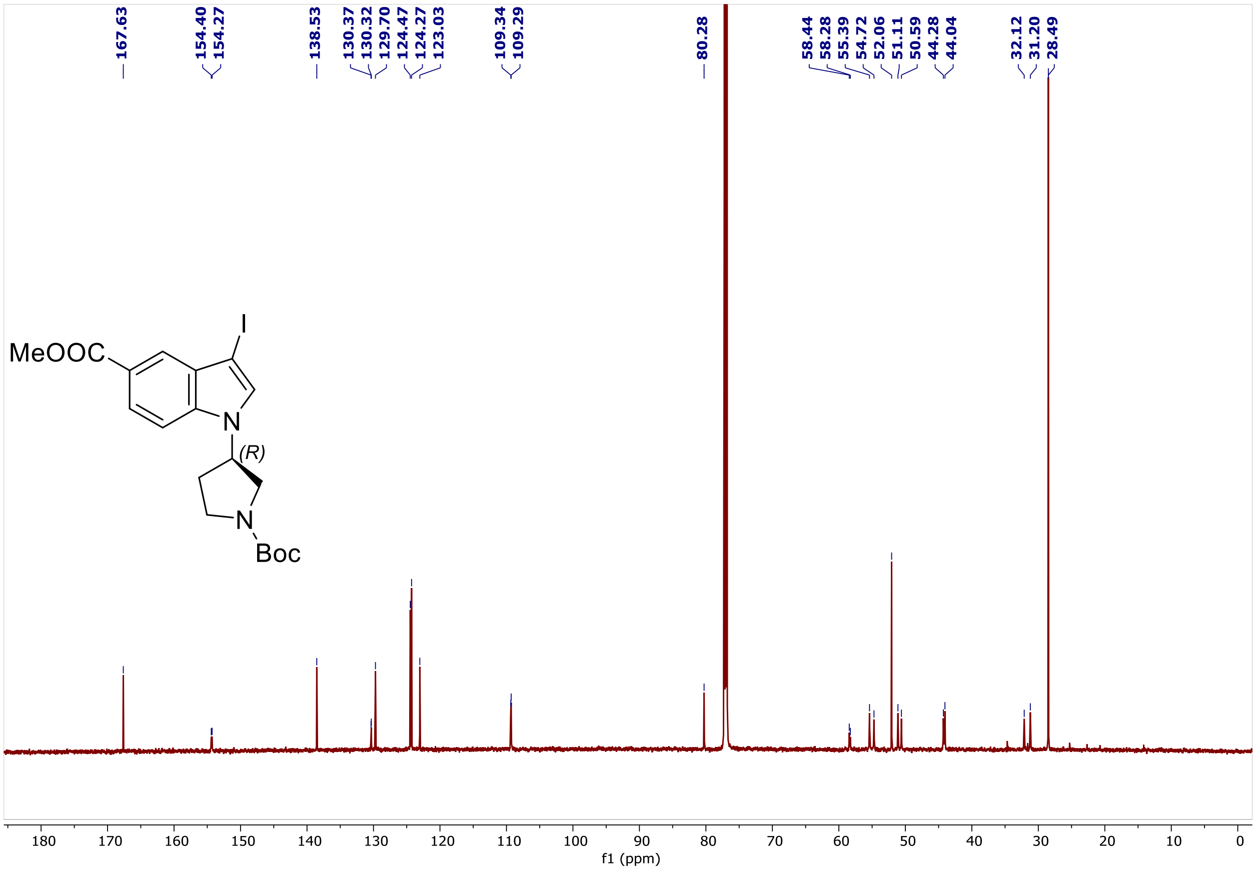

Figure S57. ^{13}C NMR (176 MHz, $CDCl_3$) spectrum of ***(R)*-10**.

Acquisition Parameter

Source Type	ESI	Ion Polarity	Positive	Set Nebulizer	0.4 Bar
Focus	Not active	Set Capillary	4500 V	Set Dry Heater	180 °C
Scan Begin	50 m/z	Set End Plate Offset	-500 V	Set Dry Gas	4.0 l/min
Scan End	1000 m/z	Set Collision Cell RF	140.0 Vpp	Set Divert Valve	Waste

#	RT [min]	Area	Int. Type	I	S/N	Chromatogram	Max. m/z	FWHM [min]
n.a.	1.6	n.a.	Single spectrum	n.a.	n.a.	n.a.	304.2624	n.a.
n.a.	3.2	n.a.	Single spectrum	n.a.	n.a.	n.a.	493.0600	n.a.

+MS, 3.2min #194

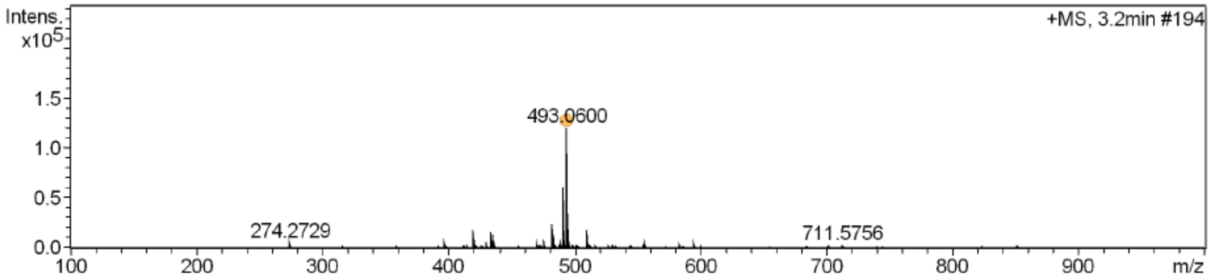

Meas. m/z	#	Ion Formula	m/z	err [ppm]	mSigma	# Sigma	Score	rdb	e⁻ Conf	N-Rule
493.0600	1	C19H23IN2NaO4	493.0595	-1.1	6.8	1	100.00	8.5	even	ok

Figure S58. HRMS (ESI$^+$) report of ***(R)*-10**.

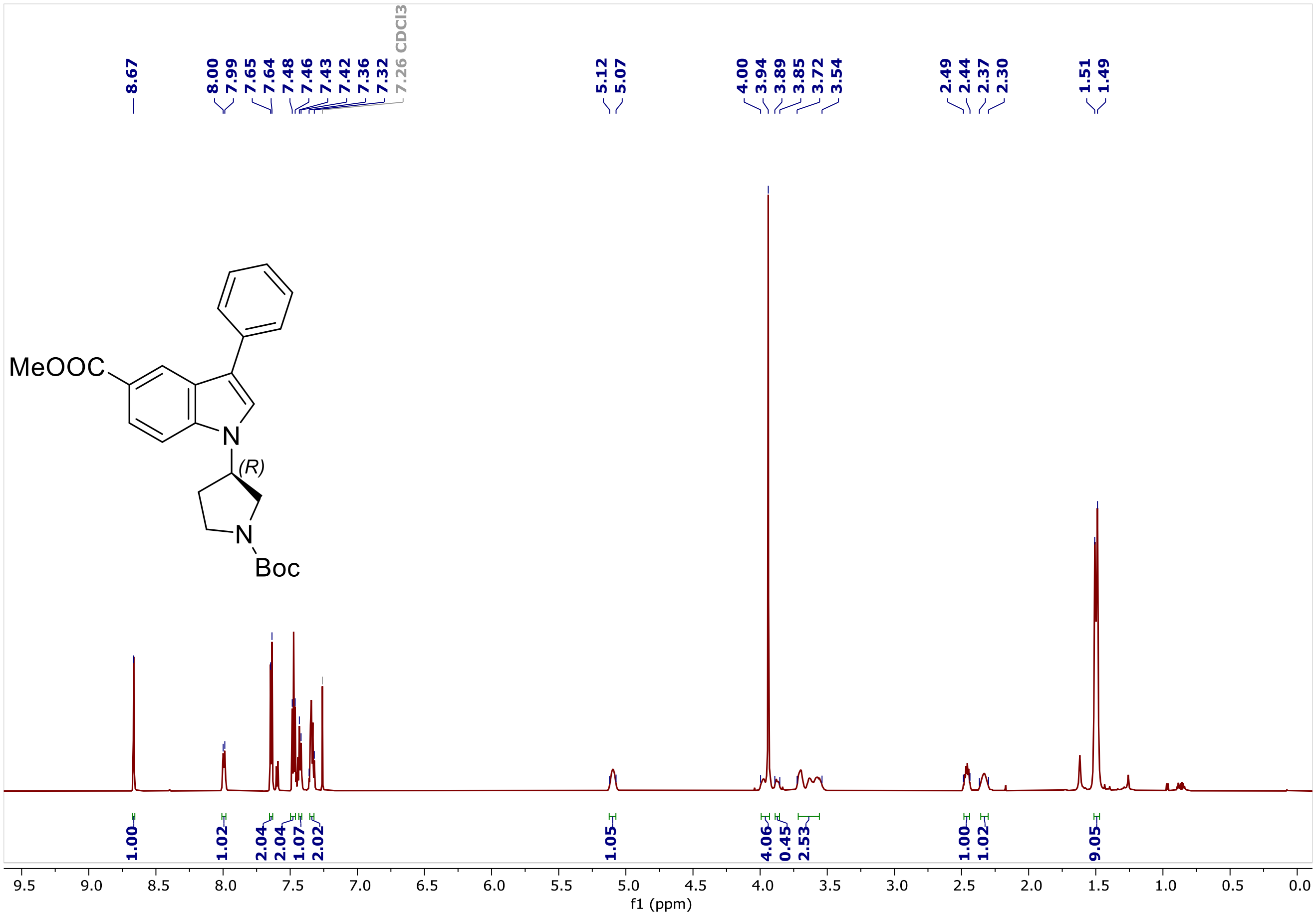

Figure S59. ^{1}H NMR (700 MHz, $CDCl_3$) spectrum of **(*R*)-12a**.

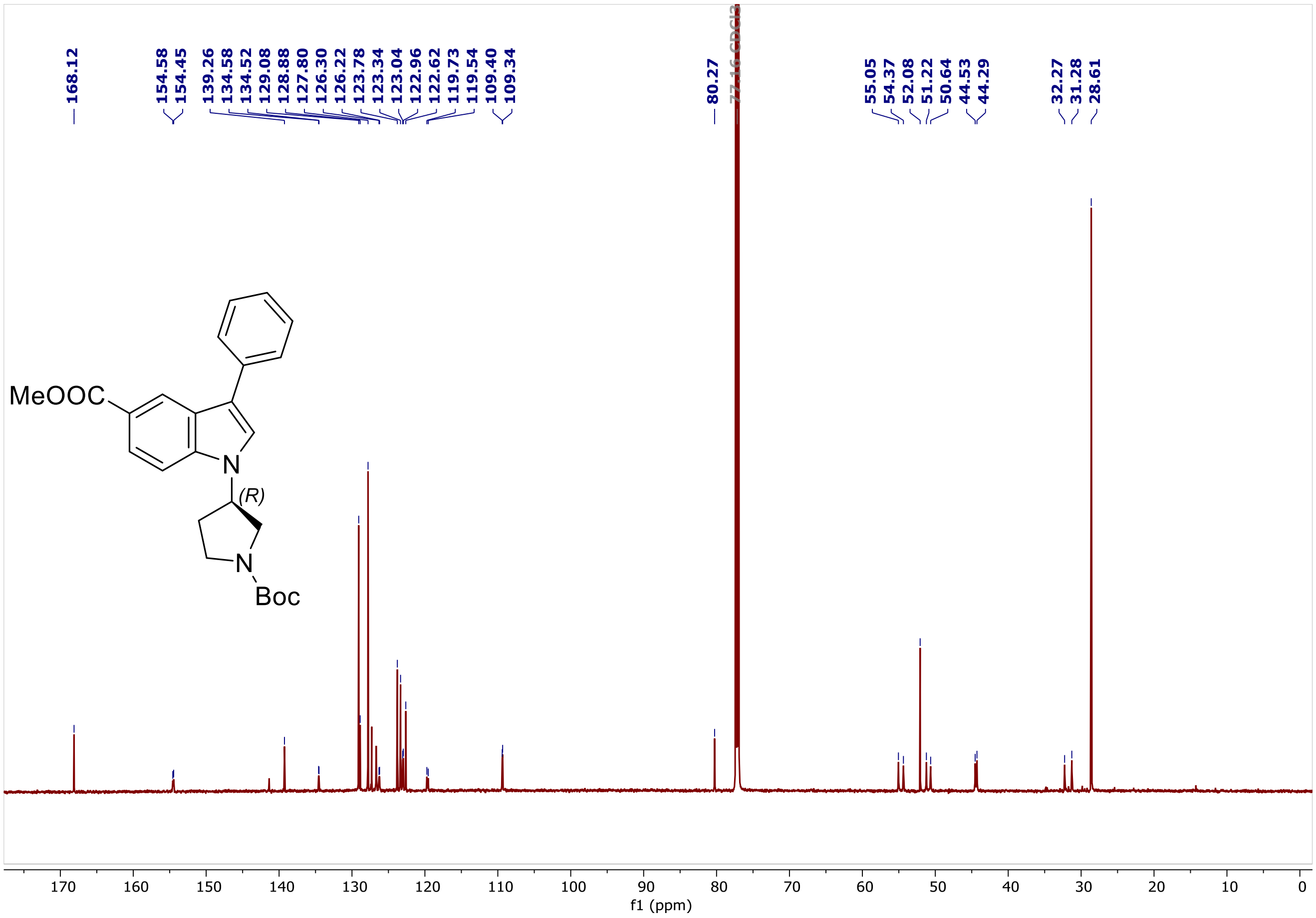

Figure S60. ^{13}C NMR (176 MHz, $CDCl_3$) spectrum of ***(R)*-12a**.

Acquisition Parameter

Source Type	ESI	Ion Polarity	Positive	Set Nebulizer	0.4 Bar
Focus	Not active	Set Capillary	4500 V	Set Dry Heater	180 °C
Scan Begin	50 m/z	Set End Plate Offset	-500 V	Set Dry Gas	4.0 l/min
Scan End	1000 m/z	Set Collision Cell RF	140.0 Vpp	Set Divert Valve	Waste

#	RT [min]	Area	Int. Type	I	S/N	Chromatogram	Max. m/z	FWHM [min]
n.a.	0.2	n.a.	Single spectrum	n.a.	n.a.	n.a.	304.2623	n.a.
n.a.	4.1	n.a.	Single spectrum	n.a.	n.a.	n.a.	443.1941	n.a.

+MS, 4.1min #246

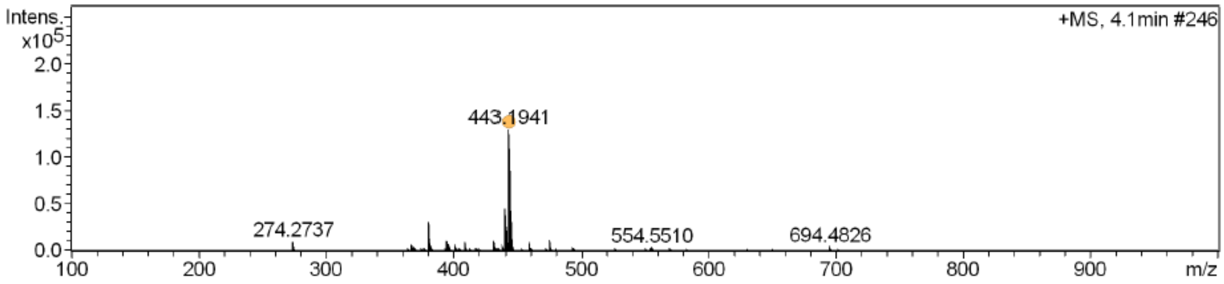

Meas. m/z	#	Ion Formula	m/z	err [ppm]	mSigma	# Sigma	Score	rdb	e⁻ Conf	N-Rule
443.1941	1	C25H28N2NaO4	443.1941	-0.1	3.3	1	100.00	12.5	even	ok

Figure S61. HRMS (ESI^+) report of ***(R)*-12a**.

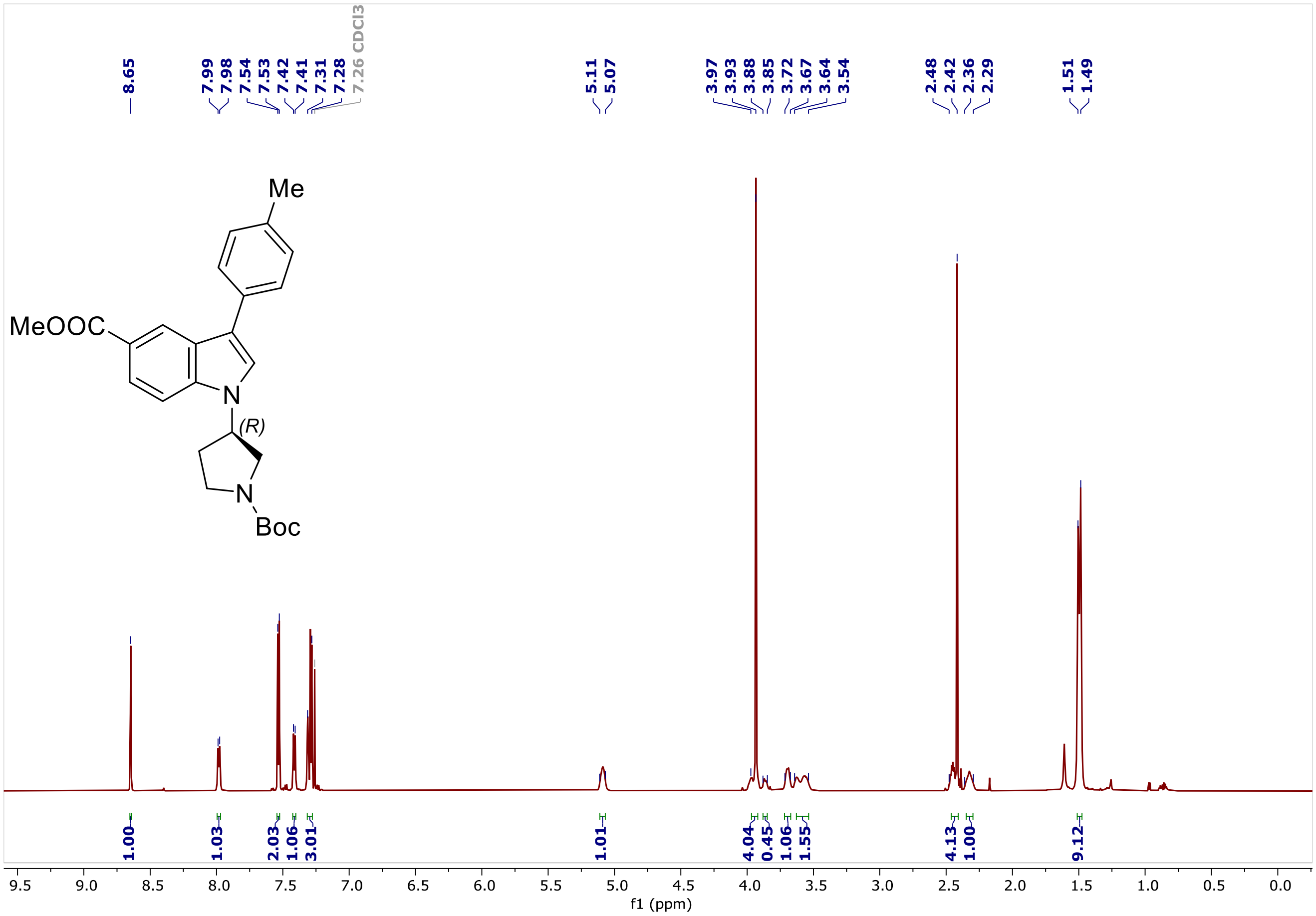

Figure S62. ^{1}H NMR (700 MHz, $CDCl_3$) spectrum of ***(R)*-12b**.

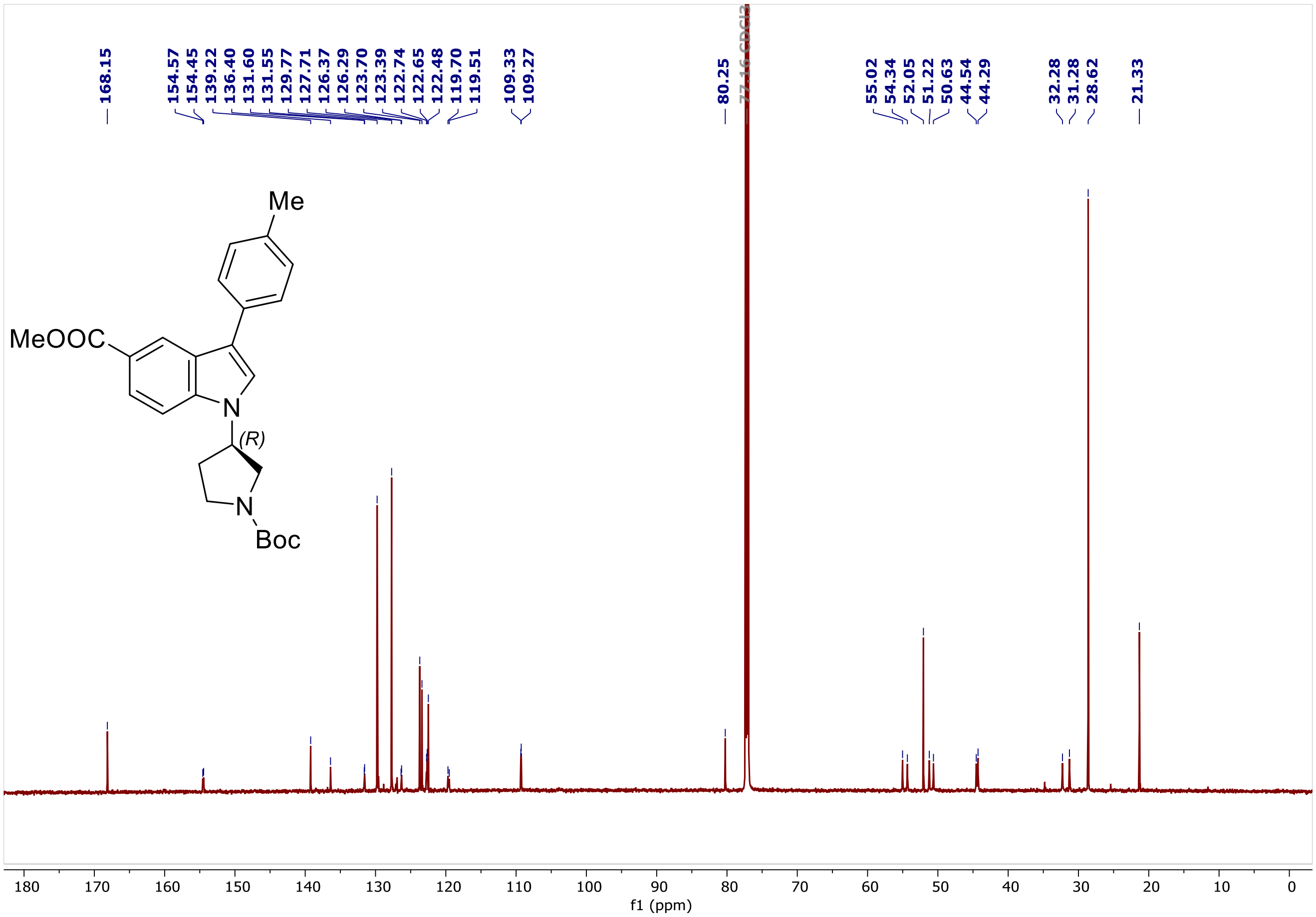

Figure S63. ^{13}C NMR (176 MHz, $CDCl_3$) spectrum of ***(R)*-12b**.

Acquisition Parameter

Source Type	ESI	Ion Polarity	Positive	Set Nebulizer	0.4 Bar
Focus	Not active	Set Capillary	4500 V	Set Dry Heater	180 °C
Scan Begin	50 m/z	Set End Plate Offset	-500 V	Set Dry Gas	4.0 l/min
Scan End	1000 m/z	Set Collision Cell RF	140.0 Vpp	Set Divert Valve	Waste

#	RT [min]	Area	Int. Type	I	S/N	Chromatogram	Max. m/z	FWHM [min]
n.a.	0.2	n.a.	Single spectrum	n.a.	n.a.	n.a.	304.2619	n.a.
n.a.	6.4	n.a.	Single spectrum	n.a.	n.a.	n.a.	457.2096	n.a.

+MS, 6.4min #384

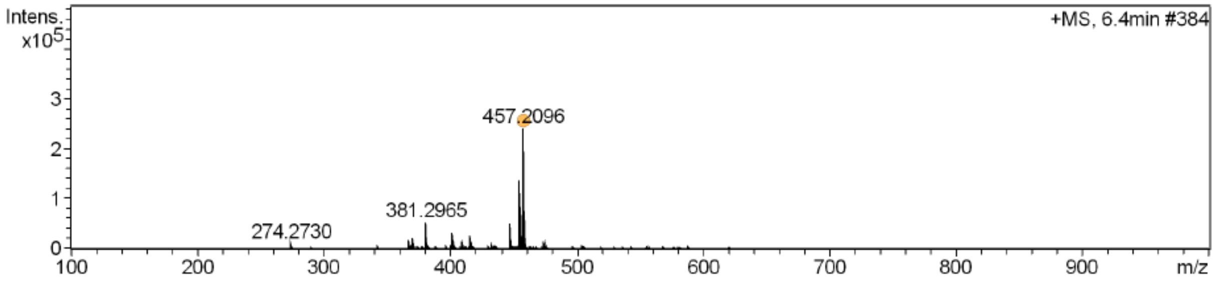

Meas. m/z	#	Ion Formula	m/z	err [ppm]	mSigma	# Sigma	Score	rdb	e⁻ Conf	N-Rule
457.2096	1	C26H30N2NaO4	457.2098	0.5	2.6	1	100.00	12.5	even	ok

Figure S64. HRMS (ESI$^+$) report of ***(R)*-12b**.

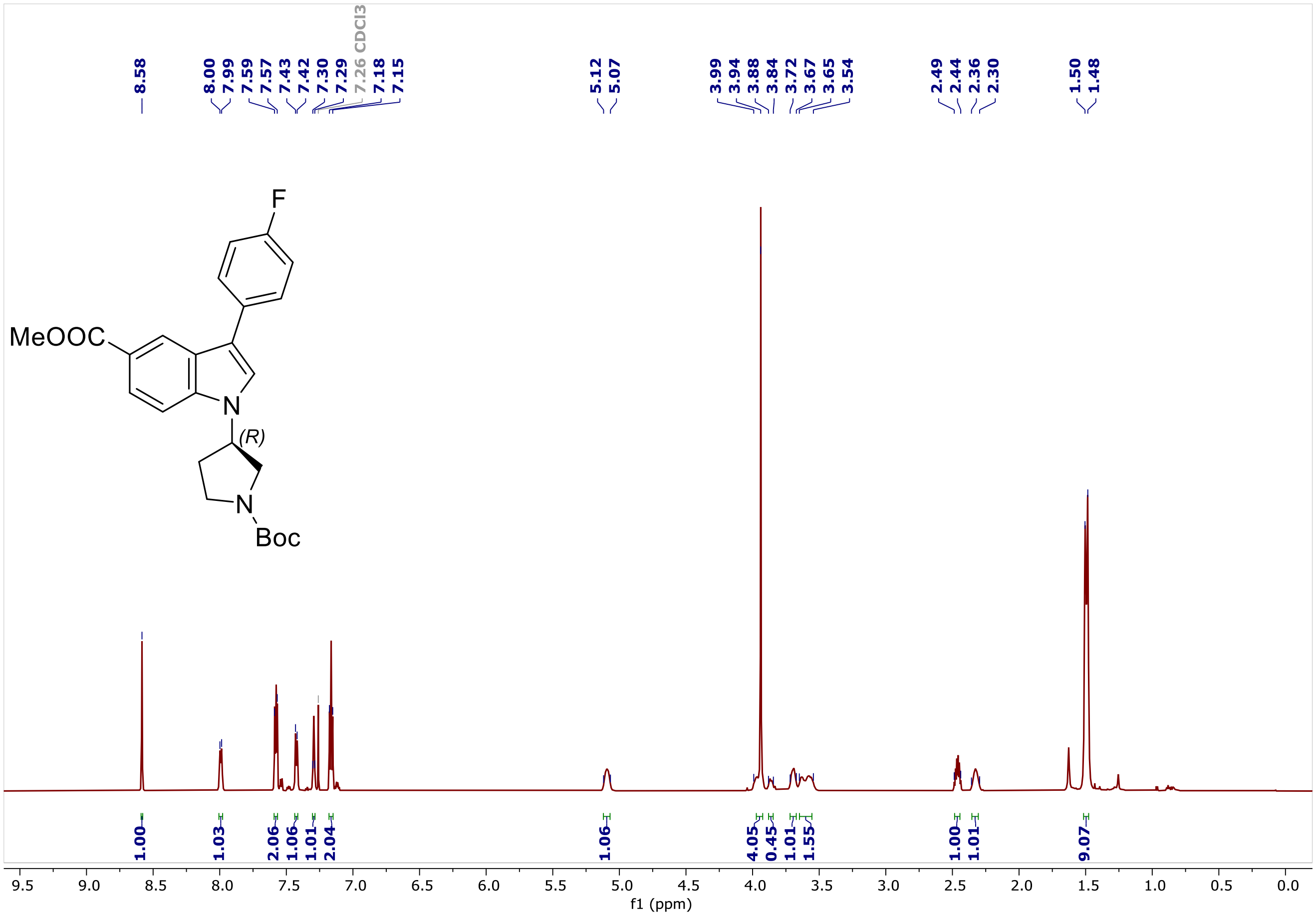

Figure S65. ^{1}H NMR (700 MHz, $CDCl_3$) spectrum of ***(R)*-12c**.

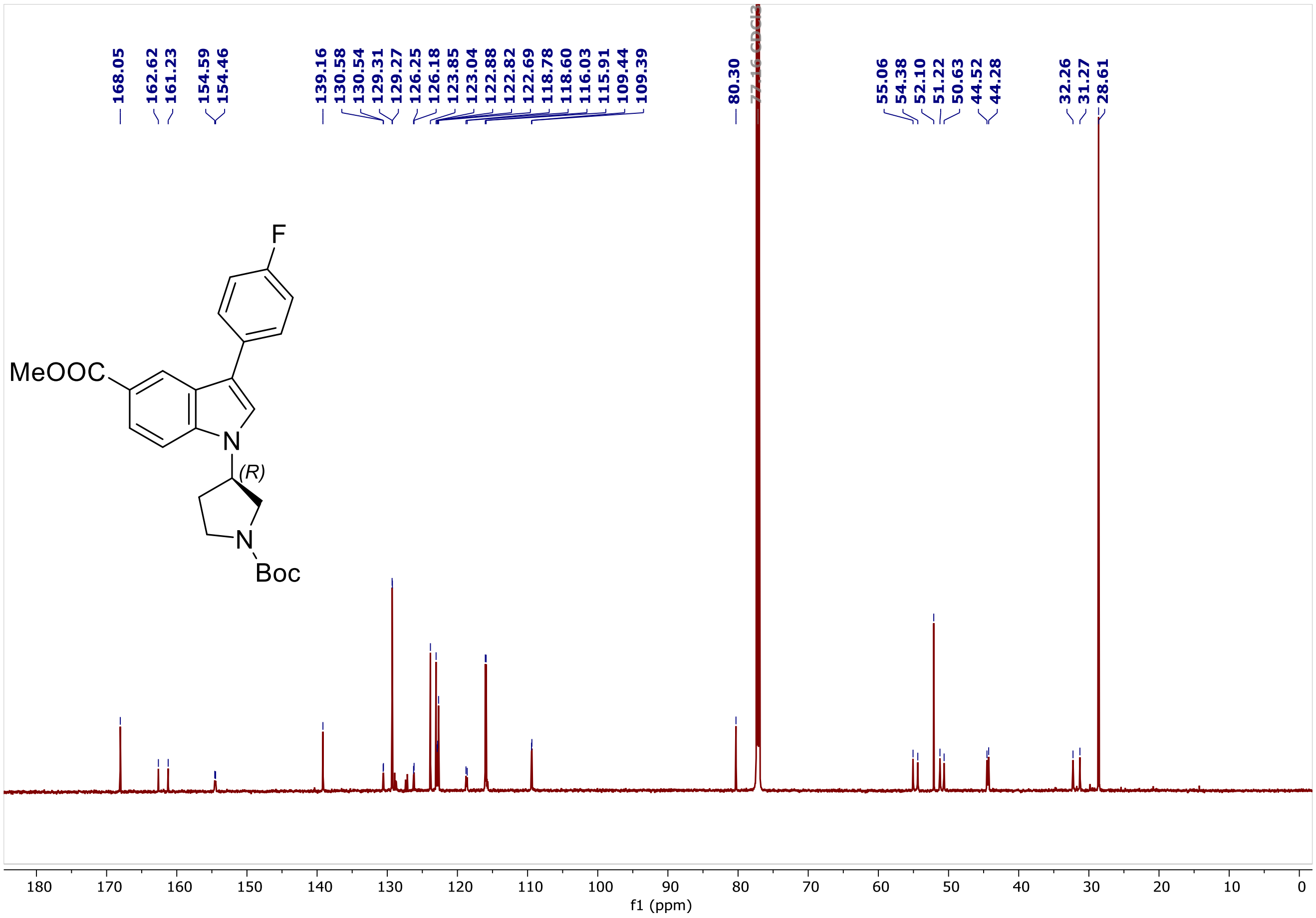

Figure S66. ^{13}C NMR (176 MHz, $CDCl_3$) spectrum of ***(R)*-12c**.

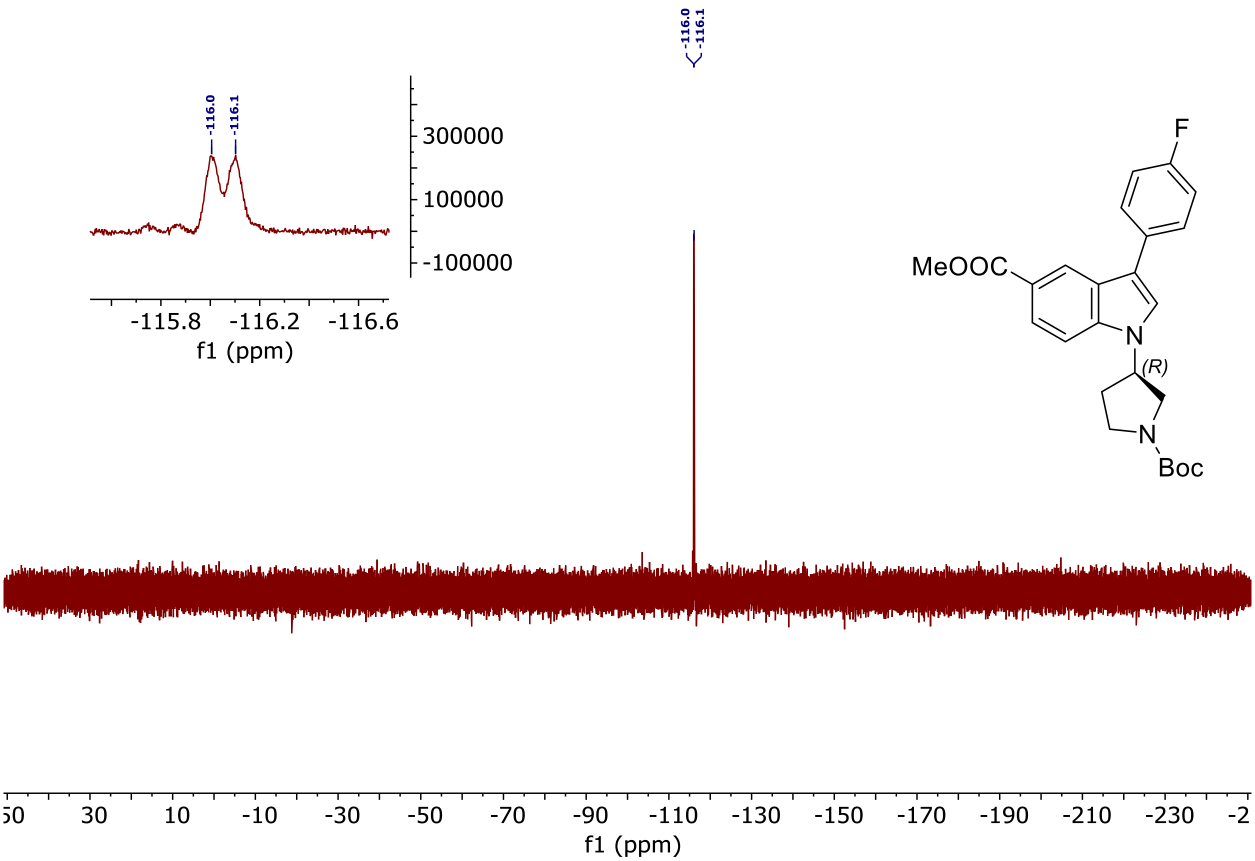

Figure S67. ^{19}F NMR (376 MHz, CDCl$_3$) spectrum of ***(R)*-12c**.

Acquisition Parameter

Source Type	ESI	Ion Polarity	Positive	Set Nebulizer	0.4 Bar		
Focus	Not active	Set Capillary	4500 V	Set Dry Heater	180 °C		
Scan Begin	50 m/z	Set End Plate Offset	-500 V	Set Dry Gas	4.0 l/min		
Scan End	1000 m/z	Set Collision Cell RF	140.0 Vpp	Set Divert Valve	Waste		

#	RT [min]	Area	Int. Type	I	S/N	Chromatogram	Max. m/z	FWHM [min]
n.a.	0.2	n.a.	Single spectrum	n.a.	n.a.	n.a.	304.2623	n.a.
n.a.	5.6	n.a.	Single spectrum	n.a.	n.a.	n.a.	461.1847	n.a.

+MS, 5.6min #335

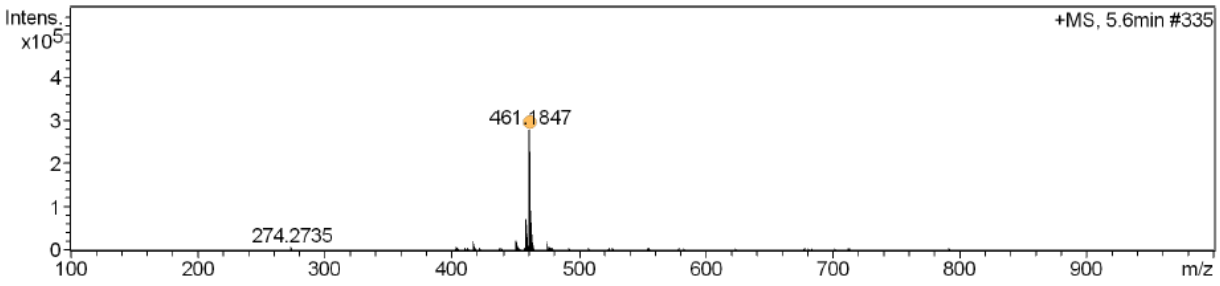

Meas. m/z	#	Ion Formula	m/z	err [ppm]	mSigma	# Sigma	Score	rdb	e⁻ Conf	N-Rule
461.1847	1	C25H27FN2NaO4	461.1847	-0.1	4.7	1	100.00	12.5	even	ok

Figure S68. HRMS (ESI^+) report of **(*R*)-12c**.

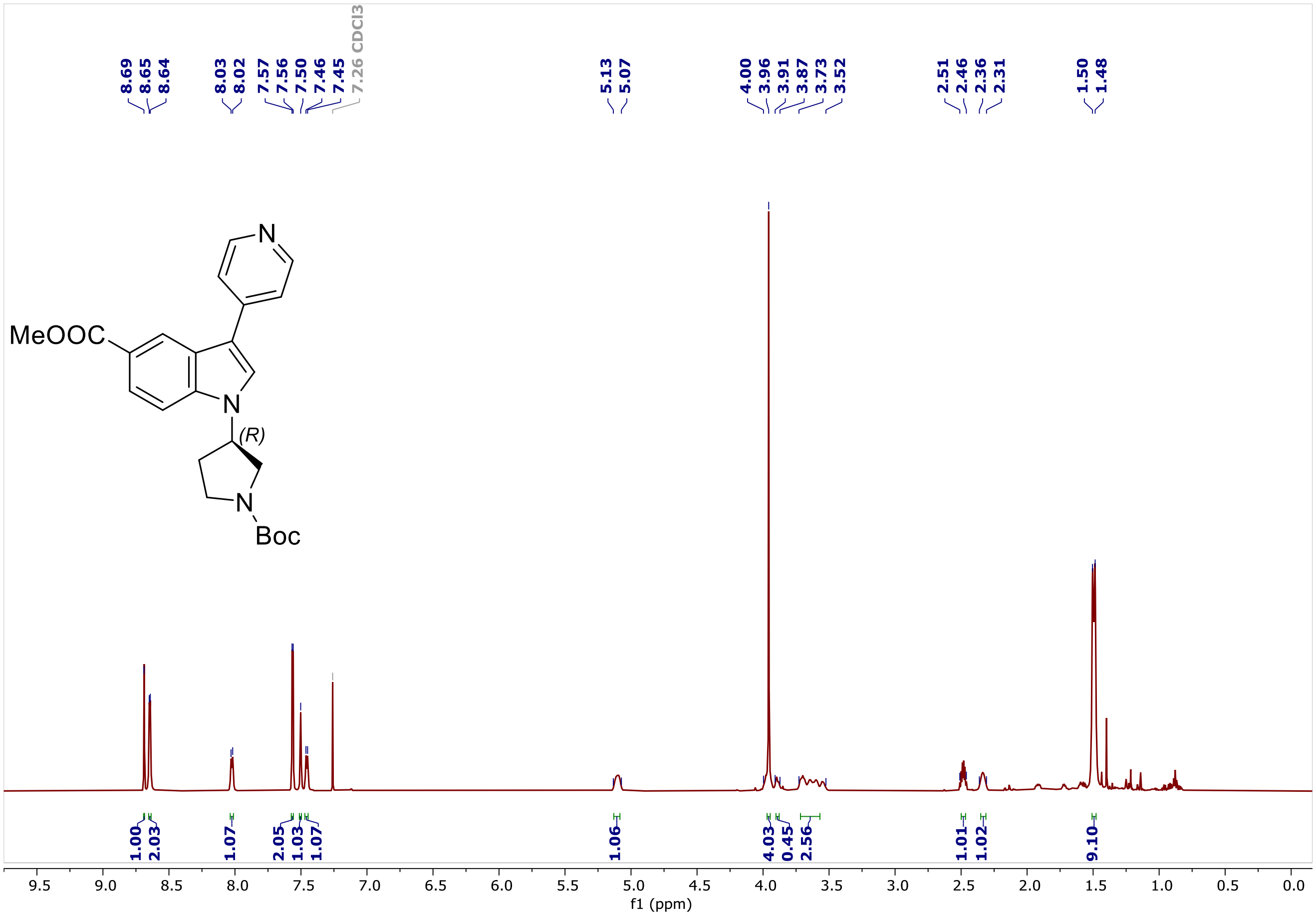

Figure S69. ^{1}H NMR (700 MHz, $CDCl_3$) spectrum of ***(R)*-12d**.

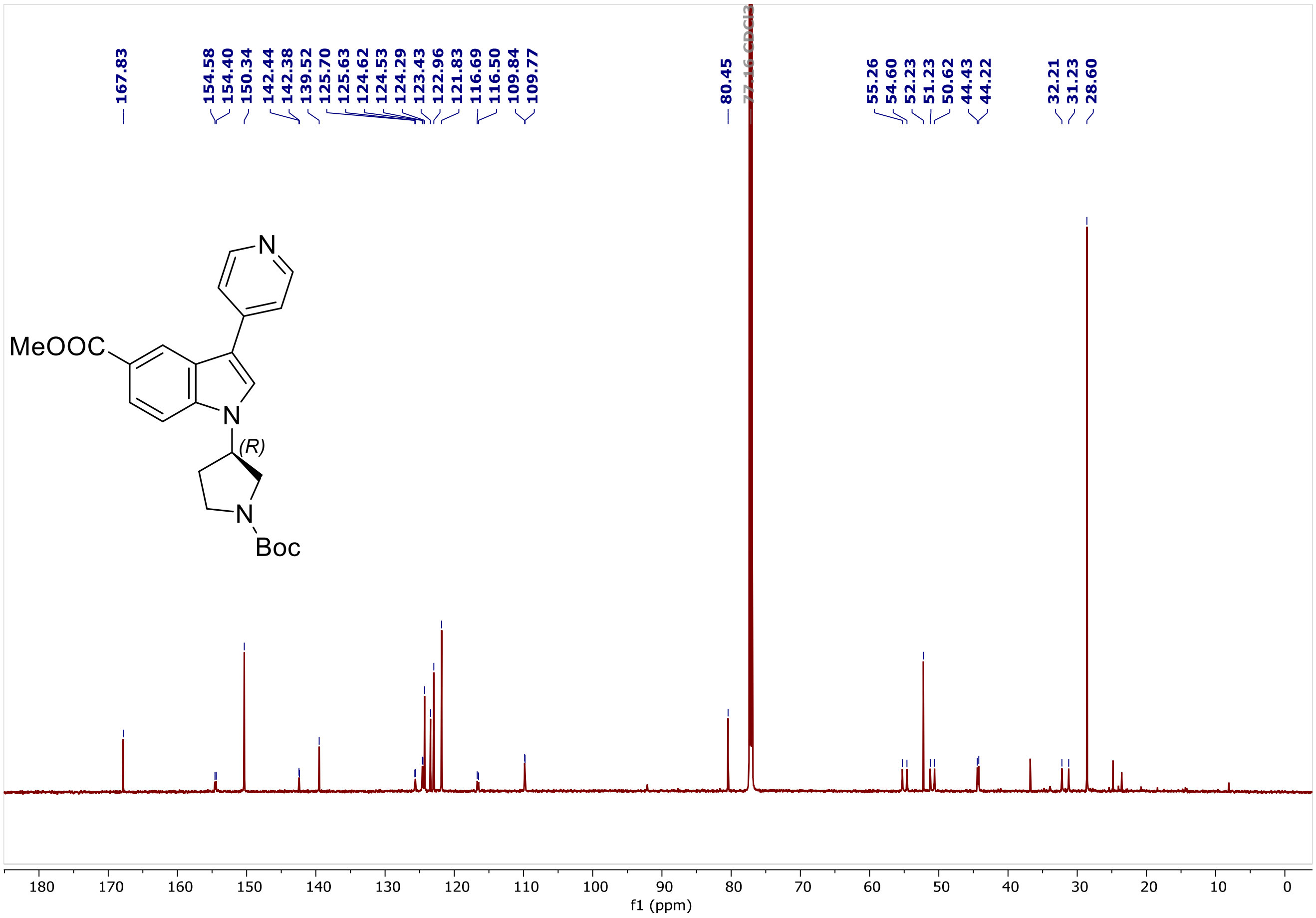

Figure S70. ^{13}C NMR (176 MHz, $CDCl_3$) spectrum of ***(R)*-12d**.

Acquisition Parameter

Source Type	ESI	Ion Polarity	Positive	Set Nebulizer	0.4 Bar
Focus	Not active	Set Capillary	4500 V	Set Dry Heater	180 °C
Scan Begin	50 m/z	Set End Plate Offset	-500 V	Set Dry Gas	4.0 l/min
Scan End	1000 m/z	Set Collision Cell RF	140.0 Vpp	Set Divert Valve	Waste

#	RT [min]	Area	Int. Type	I	S/N	Chromatogram	Max. m/z	FWHM [min]
n.a.	0.2	n.a.	Single spectrum	n.a.	n.a.	n.a.	304.2625	n.a.
n.a.	3.1	n.a.	Single spectrum	n.a.	n.a.	n.a.	422.2074	n.a.

+MS, 3.1min #187

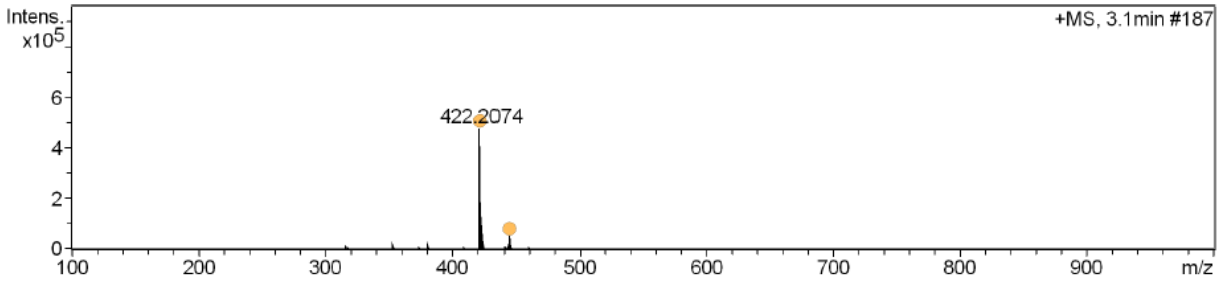

Meas. m/z	#	Ion Formula	m/z	err [ppm]	mSigma	# Sigma	Score	rdb	e⁻ Conf	N-Rule
422.2074	1	C24H28N3O4	422.2074	-0.2	8.8	1	100.00	12.5	even	ok
444.1898	1	C24H27N3NaO4	444.1894	1.0	17.2	1	100.00	12.5	even	ok

Figure S71. HRMS (ESI+) report of **(*R*)-12d**.

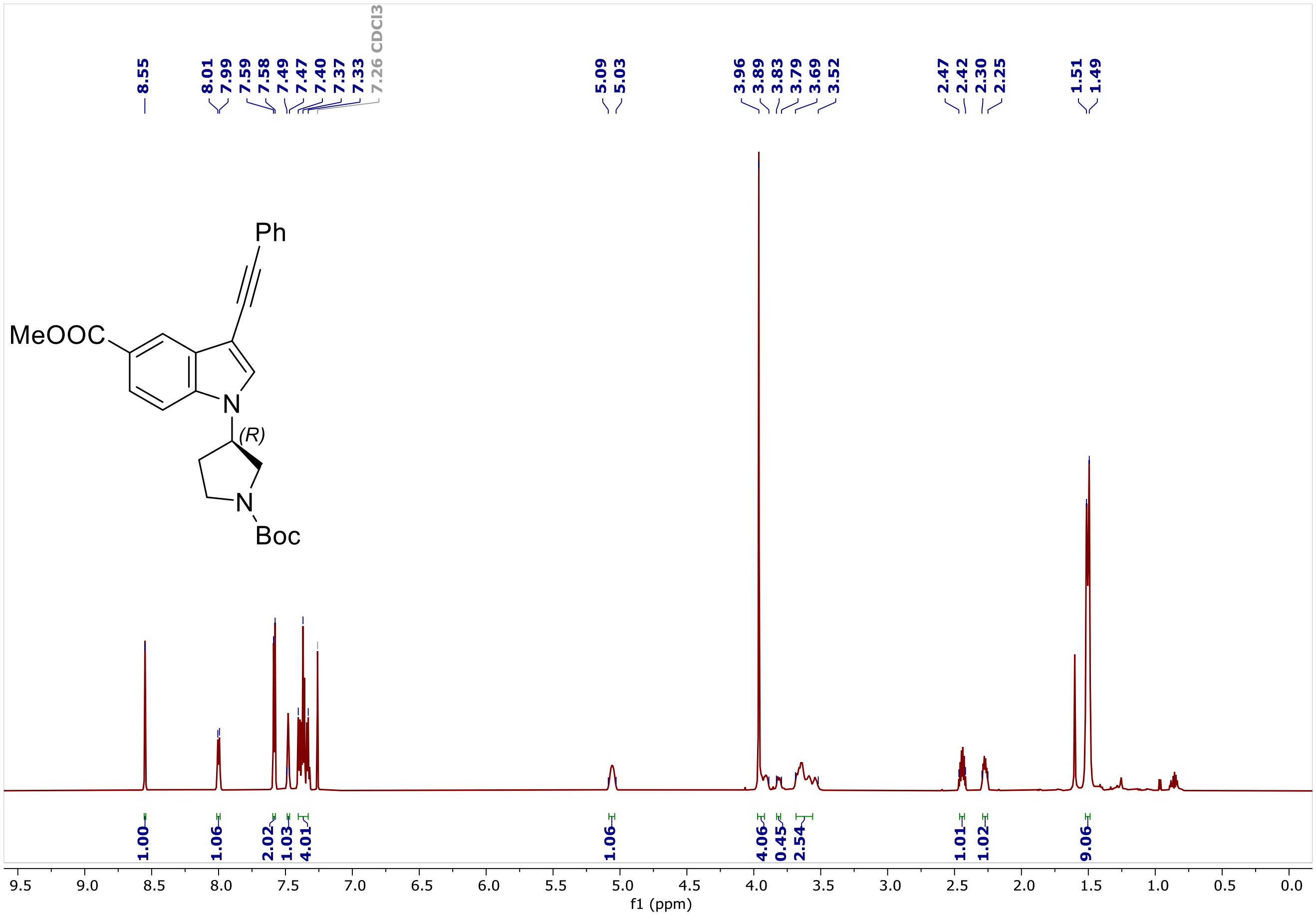

Figure S72. ^{1}H NMR (700 MHz, $CDCl_3$) spectrum of ***(R)*-12e**.

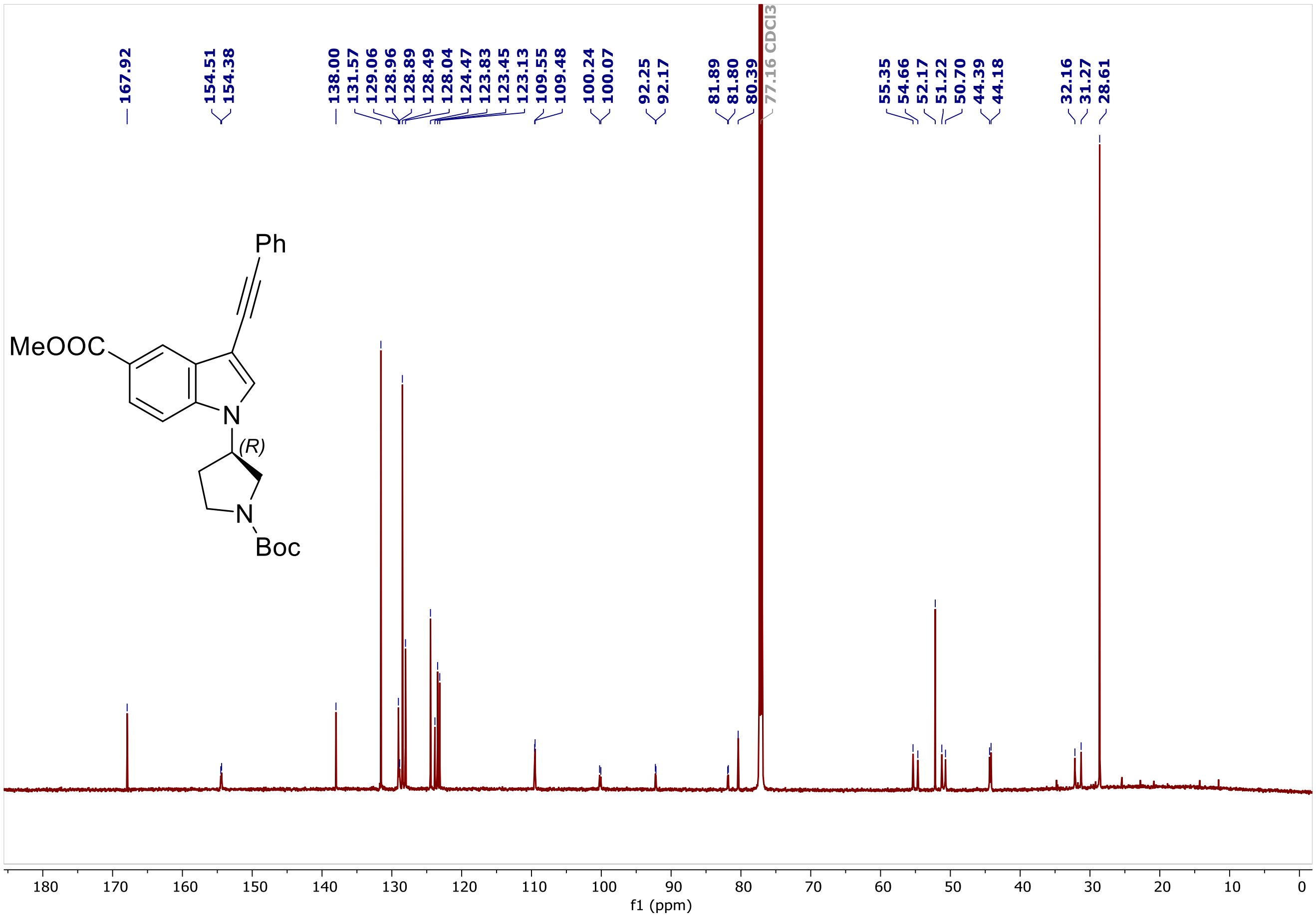

Figure S73. ^{13}C NMR (176 MHz, $CDCl_3$) spectrum of ***(R)*-12e**.

Acquisition Parameter

Source Type	ESI	Ion Polarity	Positive	Set Nebulizer	0.4 Bar
Focus	Not active	Set Capillary	4500 V	Set Dry Heater	180 °C
Scan Begin	50 m/z	Set End Plate Offset	-500 V	Set Dry Gas	4.0 l/min
Scan End	1000 m/z	Set Collision Cell RF	140.0 Vpp	Set Divert Valve	Waste

#	RT [min]	Area	Int. Type	I	S/N	Chromatogram	Max. m/z	FWHM [min]
n.a.	0.6	n.a.	Single spectrum	n.a.	n.a.	n.a.	304.2623	n.a.
n.a.	4.5	n.a.	Single spectrum	n.a.	n.a.	n.a.	467.1941	n.a.

+MS, 4.5min #271

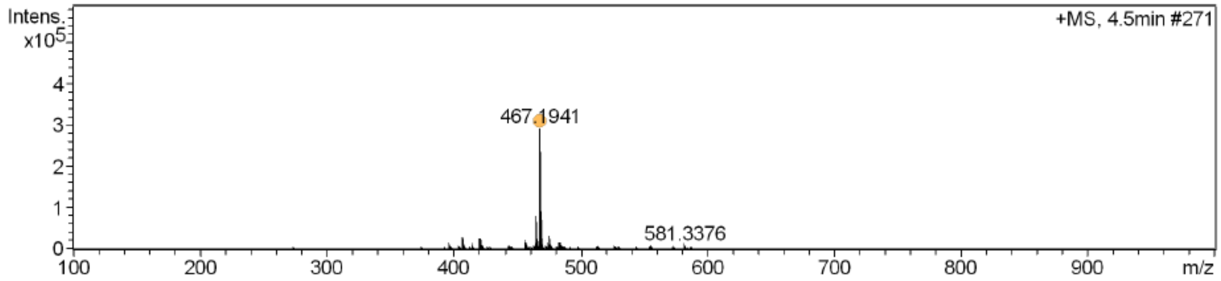

Meas. m/z	#	Ion Formula	m/z	err [ppm]	mSigma	# Sigma	Score	rdb	e⁻ Conf	N-Rule
467.1941	1	C27H28N2NaO4	467.1941	0.0	2.3	1	100.00	14.5	even	ok

Figure S74. HRMS (ESI^+) report of ***(R)*-12e**.

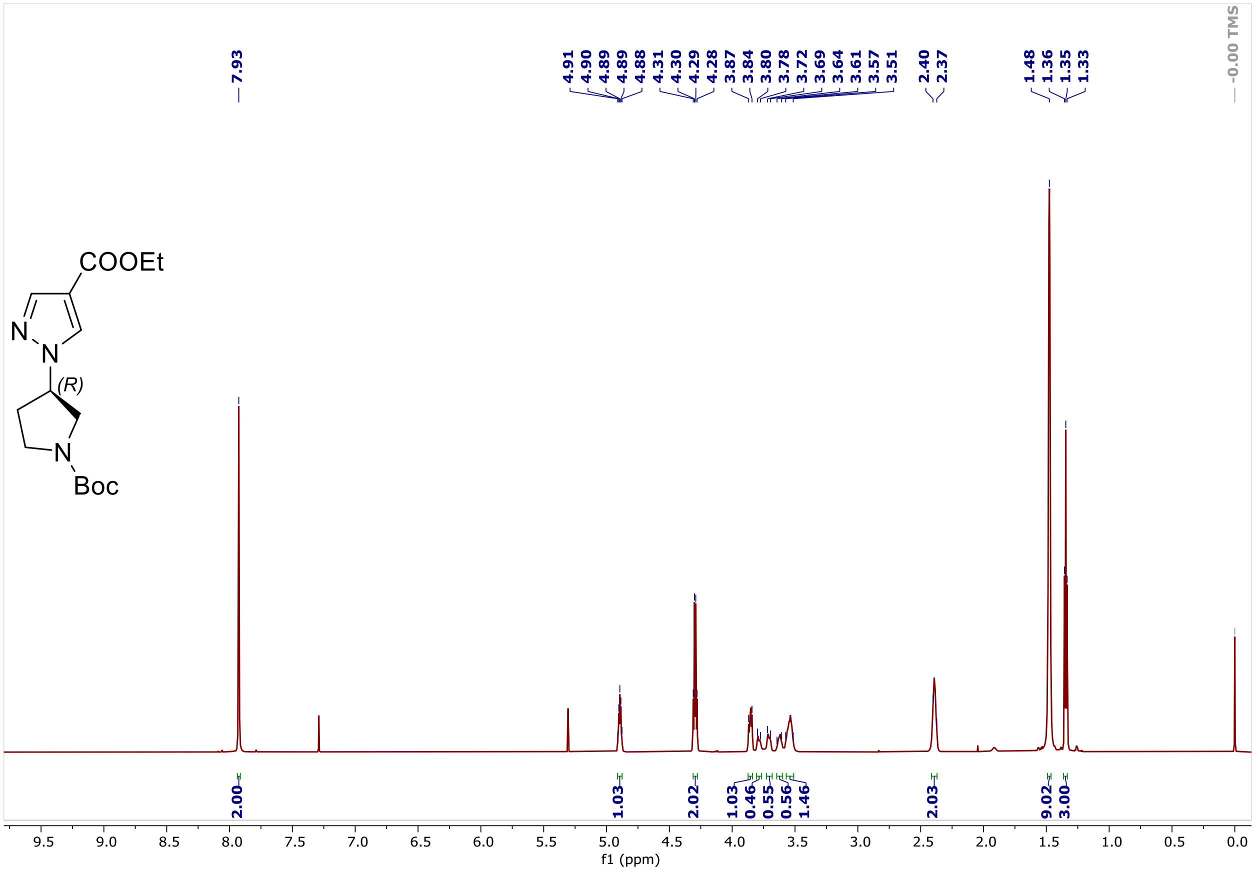

Figure S75. ^{1}H NMR (700 MHz, $CDCl_3$) spectrum of ***(R)*-14a**.

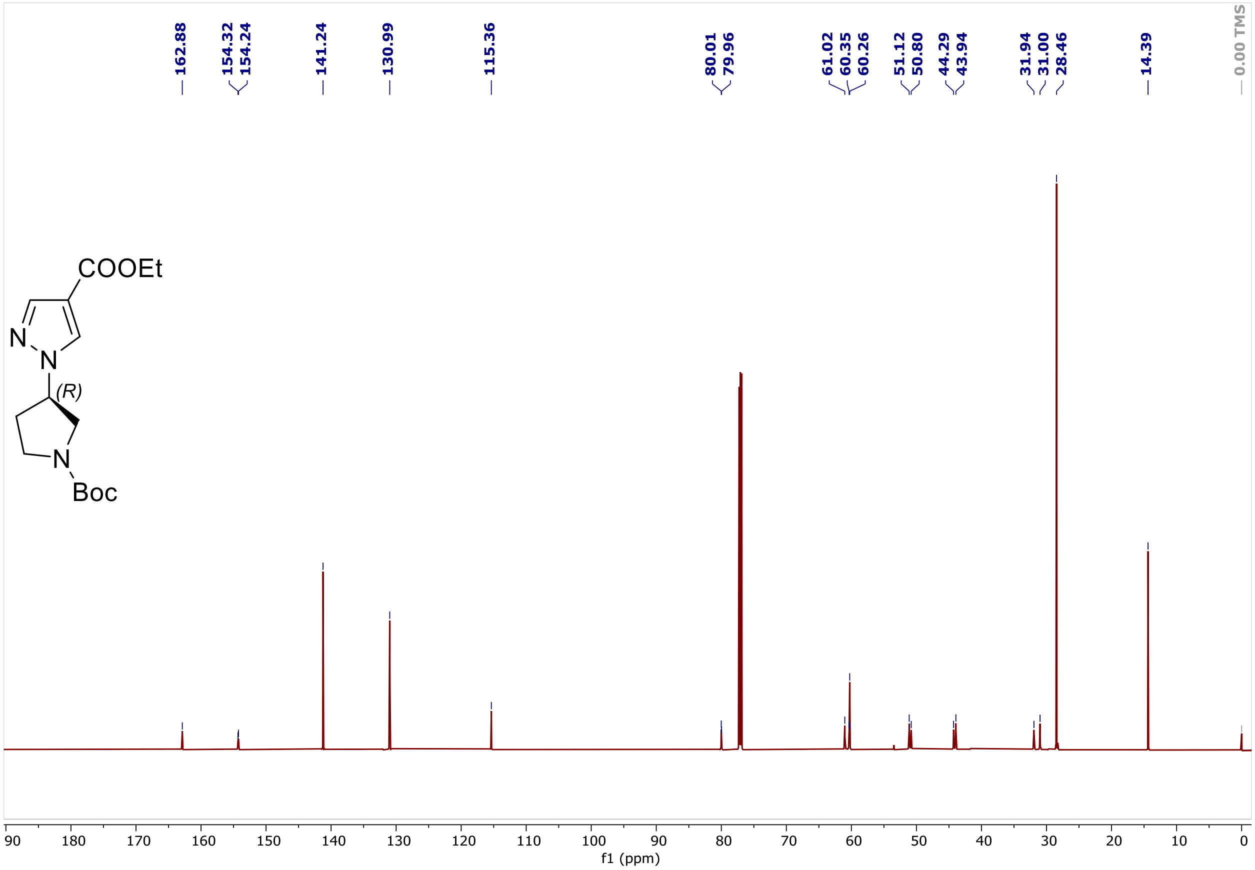

Figure S76. ^{13}C NMR (176 MHz, $CDCl_3$) spectrum of ***(R)*-14a**.

Acquisition Parameter

Source Type	ESI	Ion Polarity	Positive	Set Nebulizer	0.4 Bar
Focus	Not active	Set Capillary	4500 V	Set Dry Heater	180 °C
Scan Begin	50 m/z	Set End Plate Offset	-500 V	Set Dry Gas	4.0 l/min
Scan End	1000 m/z	Set Collision Cell RF	140.0 Vpp	Set Divert Valve	Waste

#	RT [min]	Area	Int. Type	I	S/N	Chromatogram	Max. m/z	FWHM [min]
n.a.	0.2	n.a.	Single spectrum	n.a.	n.a.	n.a.	304.2617	n.a.
n.a.	5.1	n.a.	Single spectrum	n.a.	n.a.	n.a.	332.1579	n.a.

+MS, 5.1min #306

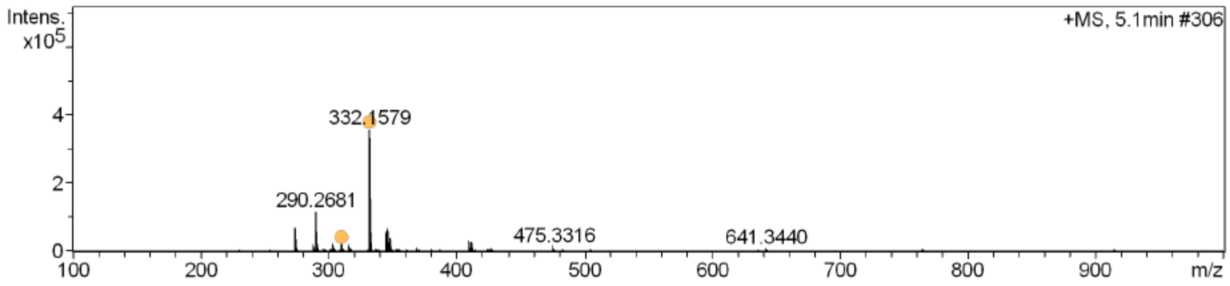

Meas. m/z	#	Ion Formula	m/z	err [ppm]	mSigma	# Sigma	Score	rdb	e⁻ Conf	N-Rule
310.1757	1	C15H24N3O4	310.1761	1.4	51.4	1	100.00	5.5	even	ok
332.1579	1	C15H23N3NaO4	332.1581	0.6	4.7	1	100.00	5.5	even	ok

Figure S77. HRMS (ESI$^+$) report of ***(R)*-14a**.

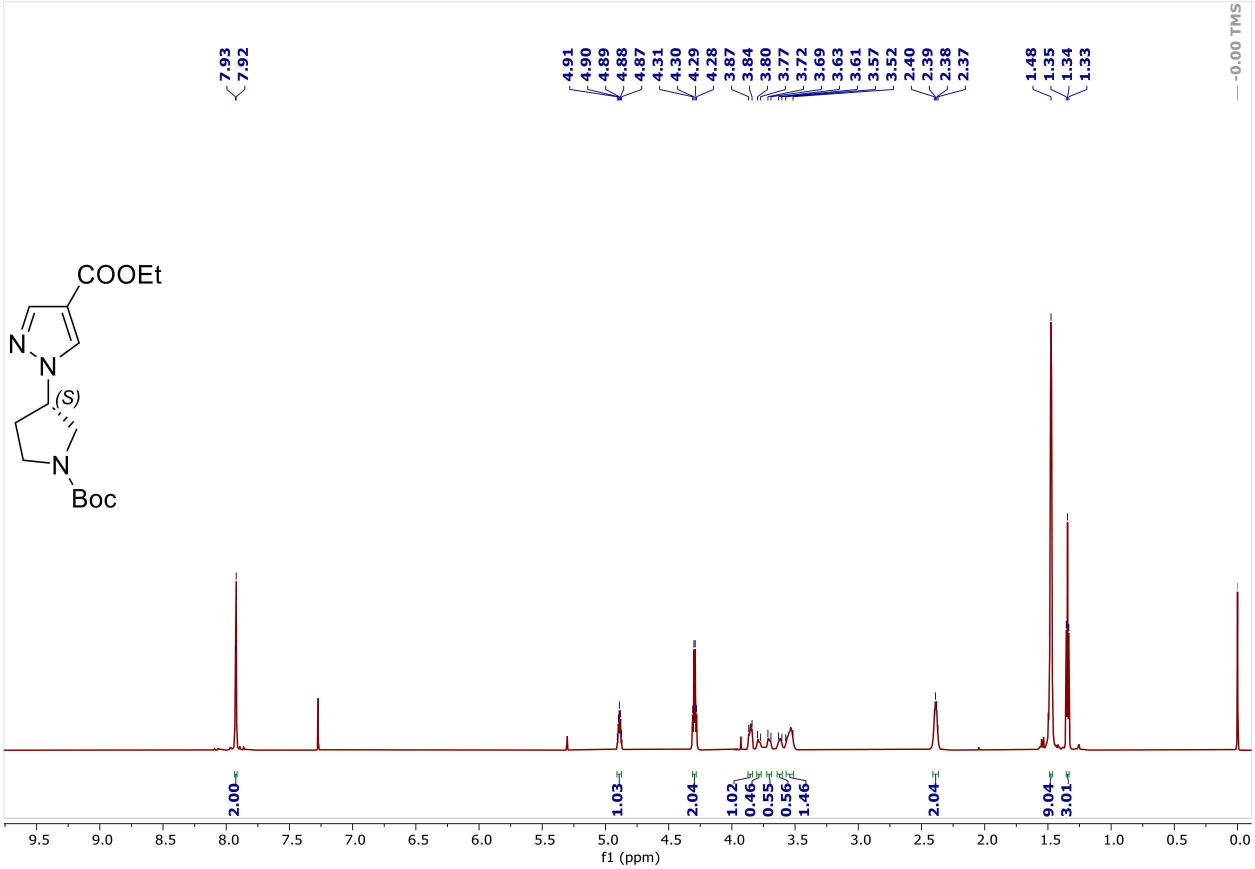

Figure S78. ^{1}H NMR (700 MHz, $CDCl_3$) spectrum of ***(S)*-14a**.

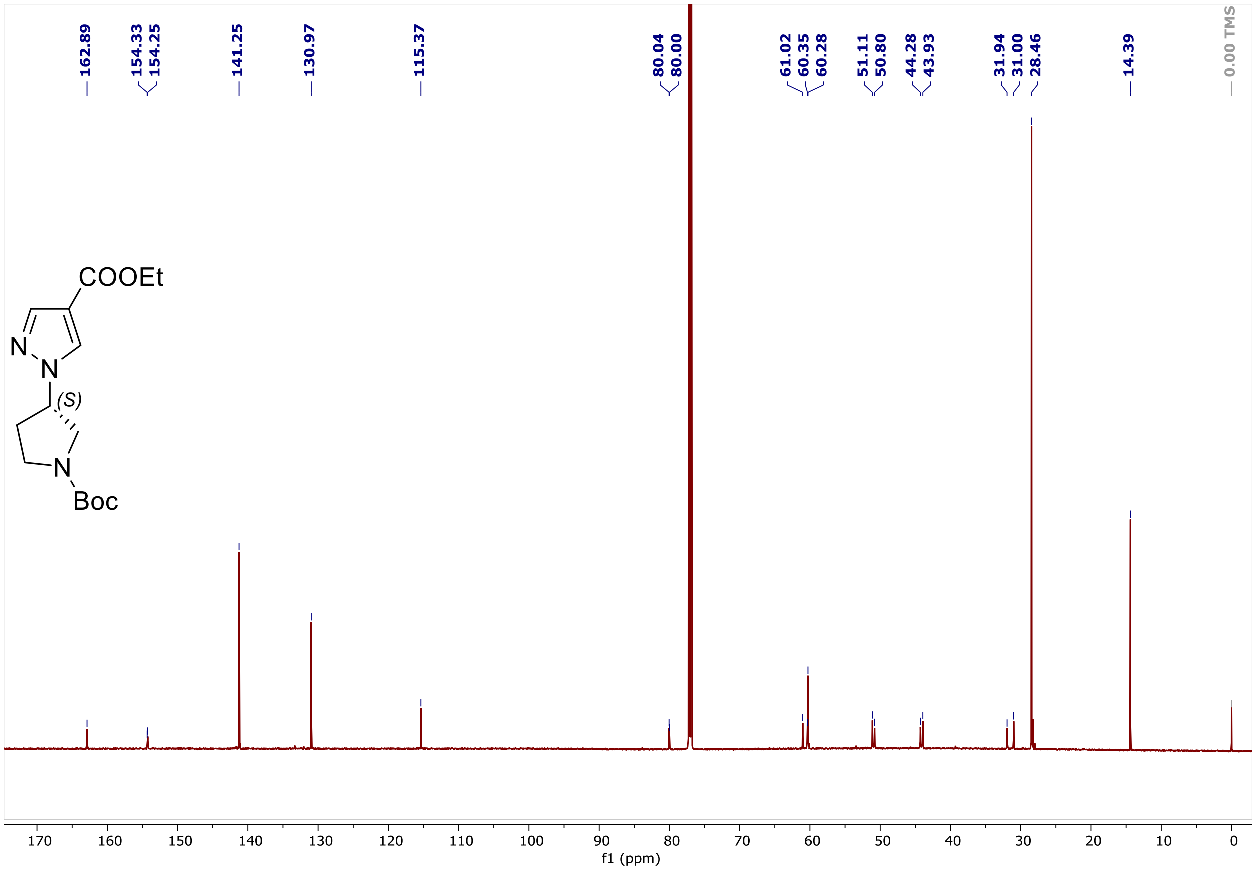

Figure S79. ^{13}C NMR (176 MHz, $CDCl_3$) spectrum of ***(S)*-14a**.

Acquisition Parameter

Source Type	ESI	Ion Polarity	Positive	Set Nebulizer	0.4 Bar
Focus	Not active	Set Capillary	4500 V	Set Dry Heater	180 °C
Scan Begin	50 m/z	Set End Plate Offset	-500 V	Set Dry Gas	4.0 l/min
Scan End	1000 m/z	Set Collision Cell RF	140.0 Vpp	Set Divert Valve	Waste

#	RT [min]	Area	Int. Type	I	S/N	Chromatogram	Max. m/z	FWHM [min]
n.a.	0.2	n.a.	Single spectrum	n.a.	n.a.	n.a.	304.2622	n.a.
n.a.	5.0	n.a.	Single spectrum	n.a.	n.a.	n.a.	332.1581	n.a.

+MS, 5.0min #297

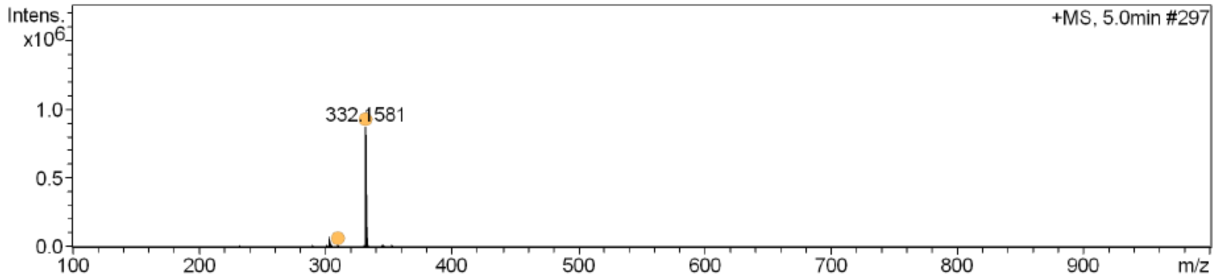

Meas. m/z	#	Ion Formula	m/z	err [ppm]	mSigma	# Sigma	Score	rdb	e⁻ Conf	N-Rule
310.1768	1	C15H24N3O4	310.1761	-2.1	126.6	1	100.00	5.5	even	ok
332.1581	1	C15H23N3NaO4	332.1581	0.1	1.0	1	100.00	5.5	even	ok

Figure S80. HRMS (ESI^{+}) report of ***(S)*-14a**.

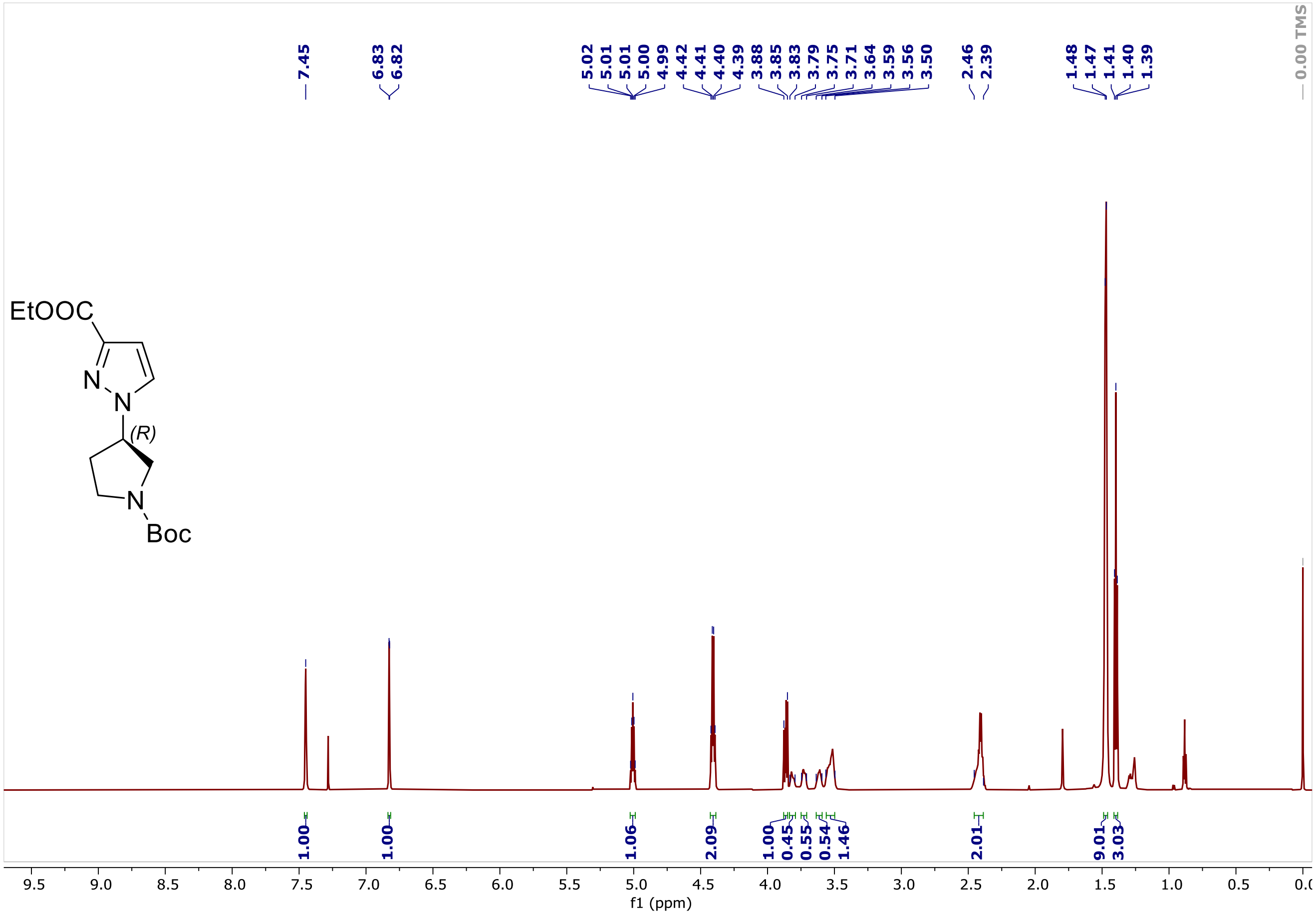

Figure S81. ^{1}H NMR (700 MHz, $CDCl_3$) spectrum of ***(R)*-14b**.

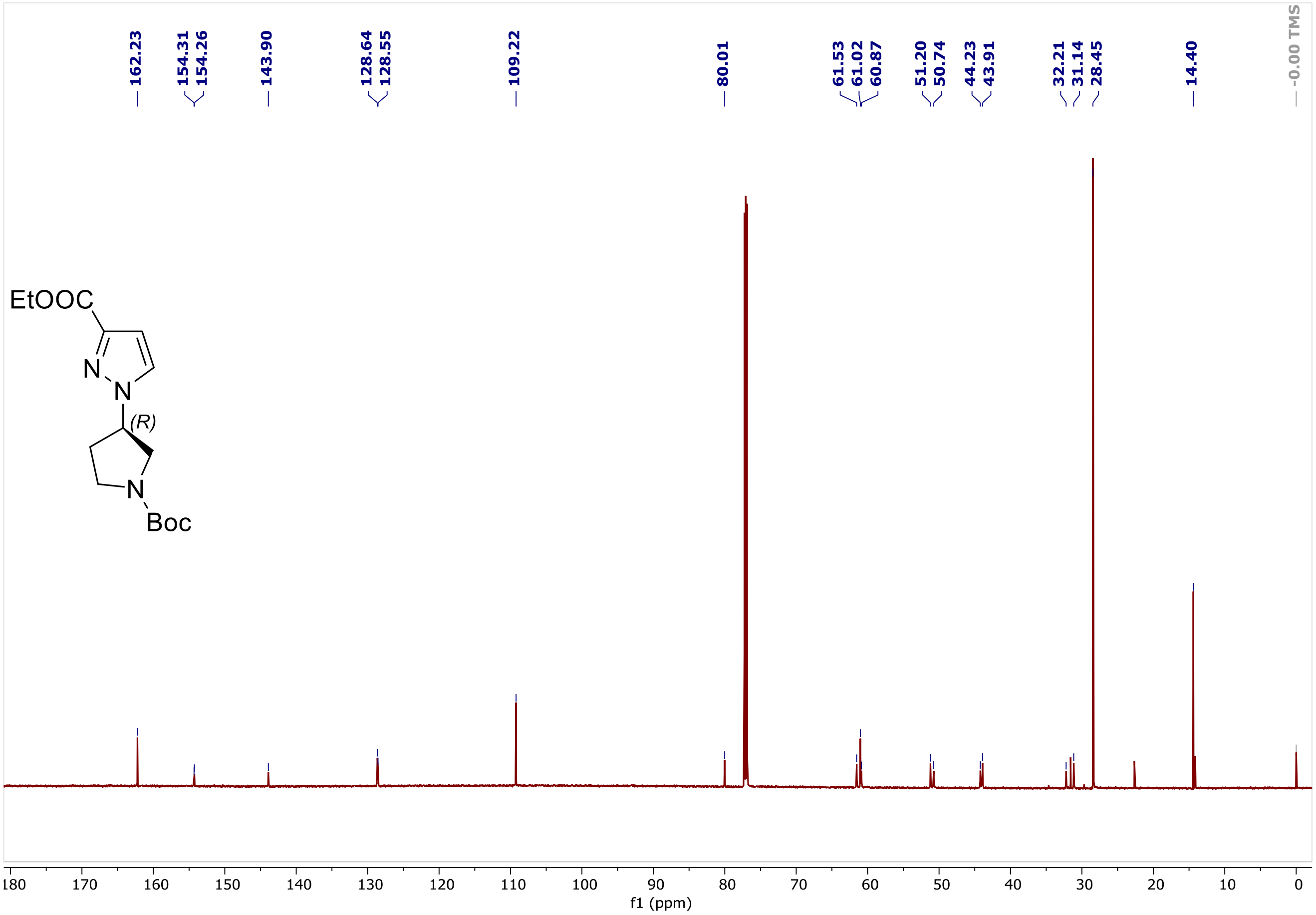

Figure S82. ^{13}C NMR (176 MHz, $CDCl_3$) spectrum of ***(R)*-14b**.

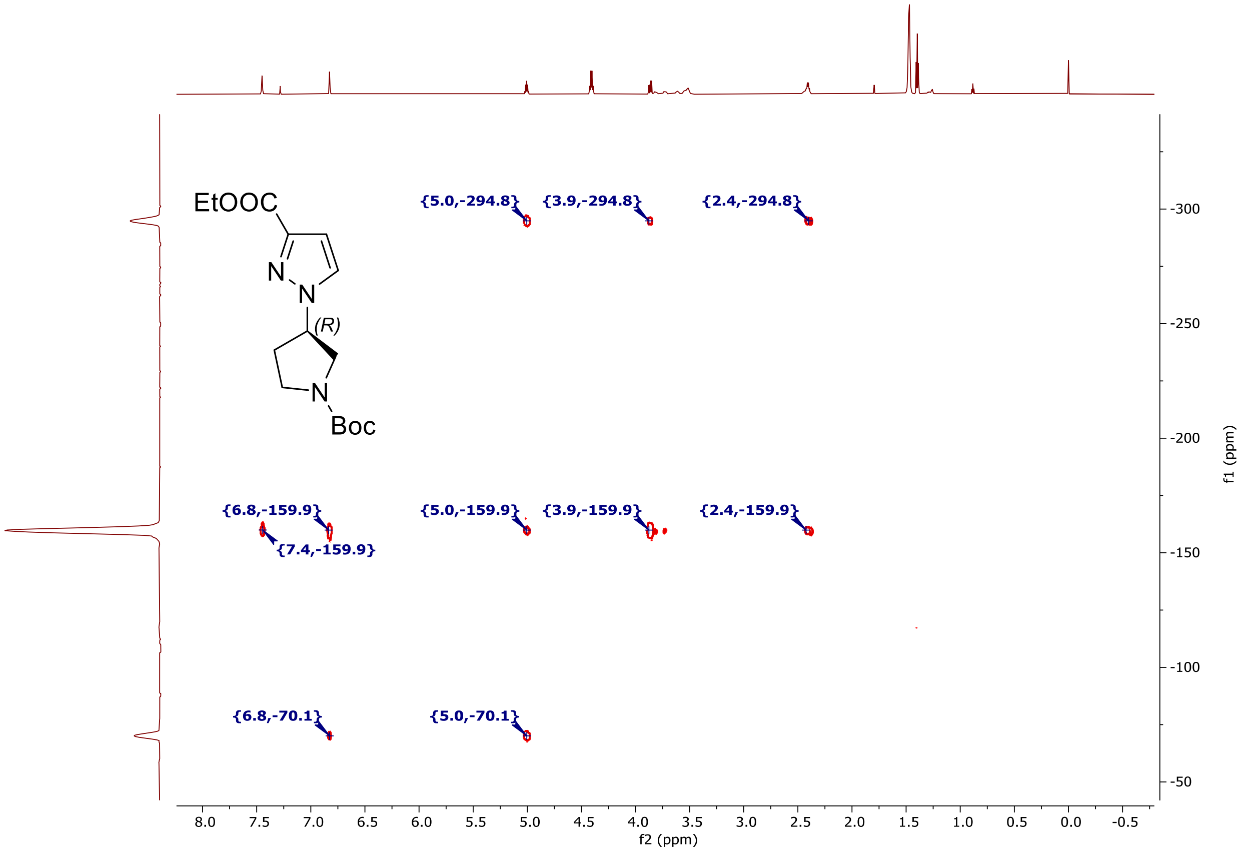

Figure S83. ^{1}H-^{15}N HMBC NMR spectrum of ***(R)*-14b** (71 MHz, $CDCl_3$).

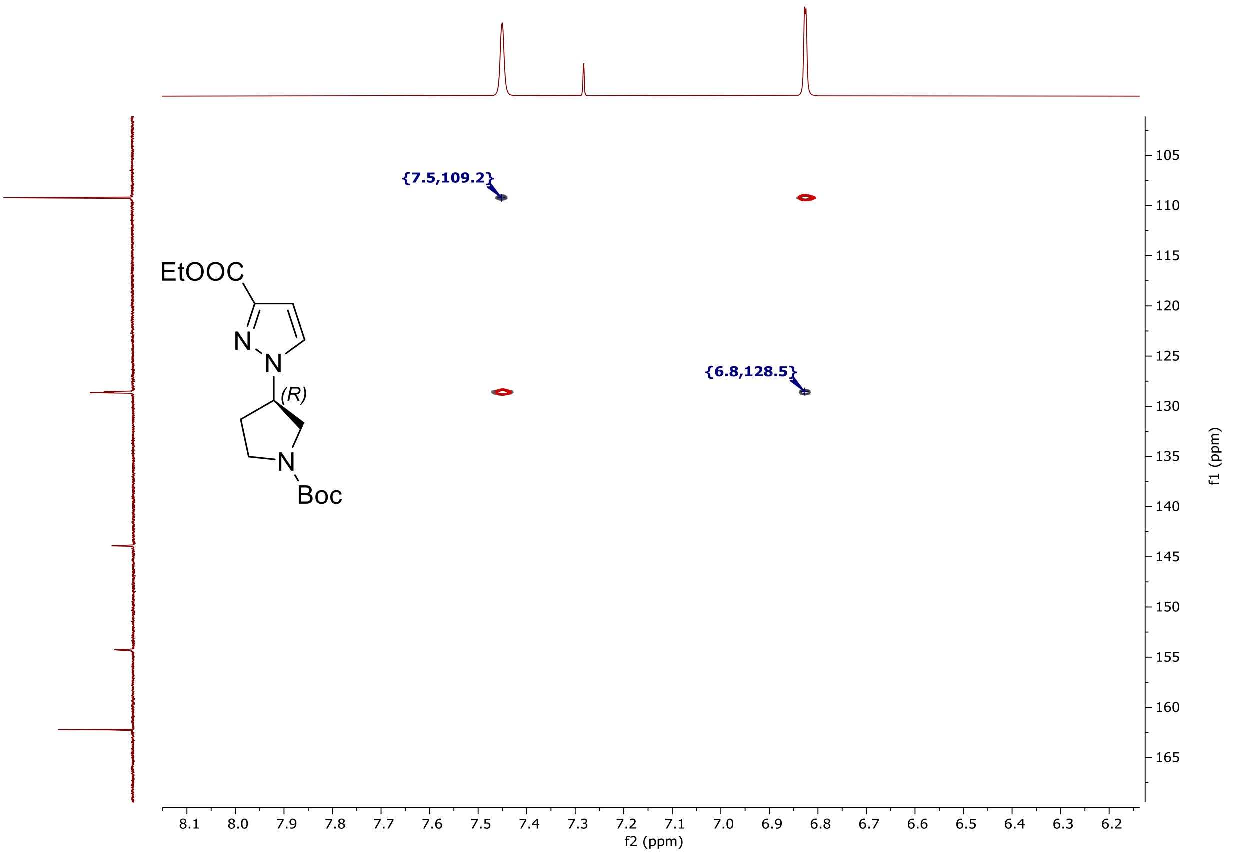

Figure S84. Overlaid multiplicity-edited ^{1}H-^{13}C HSQC (red correlations) and ^{1}H-^{13}C H2BC (black correlations) NMR spectra of ***(R)*-14b** ($CDCl_3$).

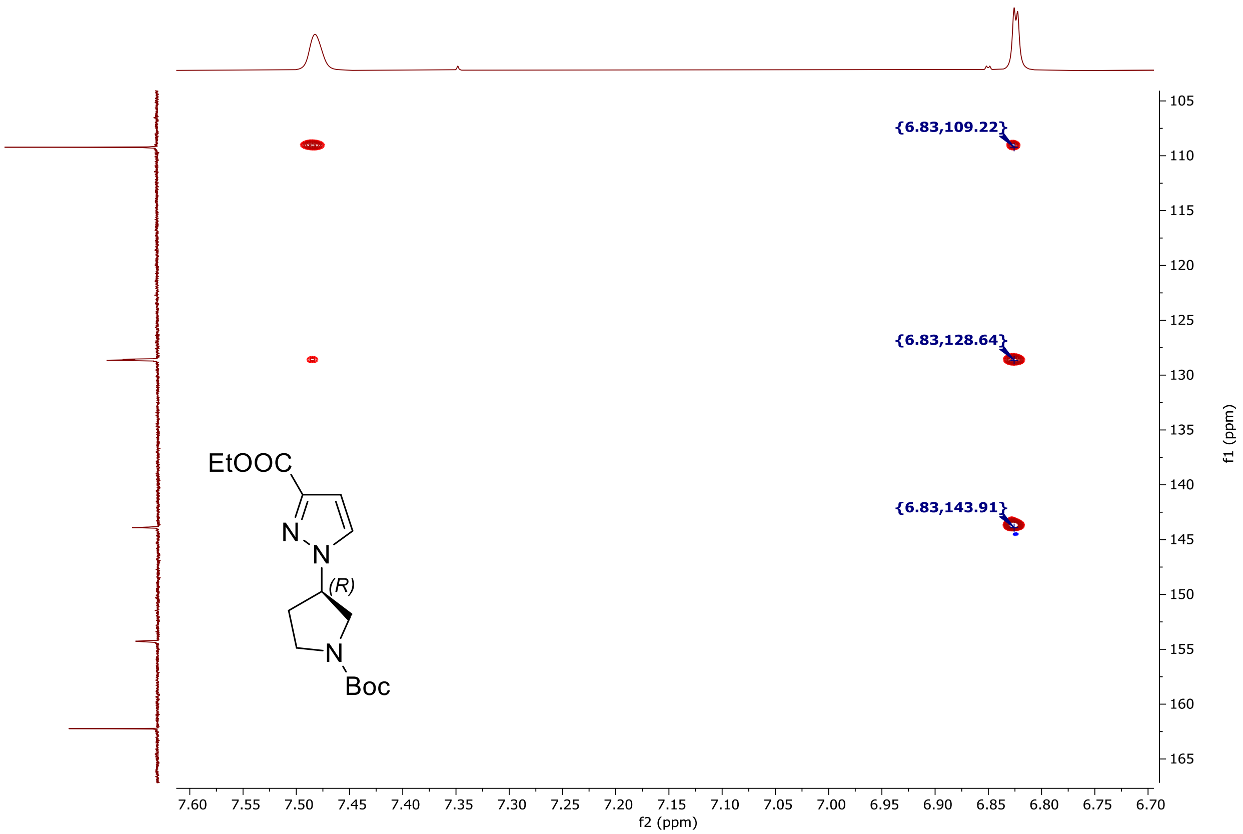

Figure S85. 1,1-ADEQUATE 60Hz NMR spectrum of **(*R*)-14b** ($CDCl_3$).

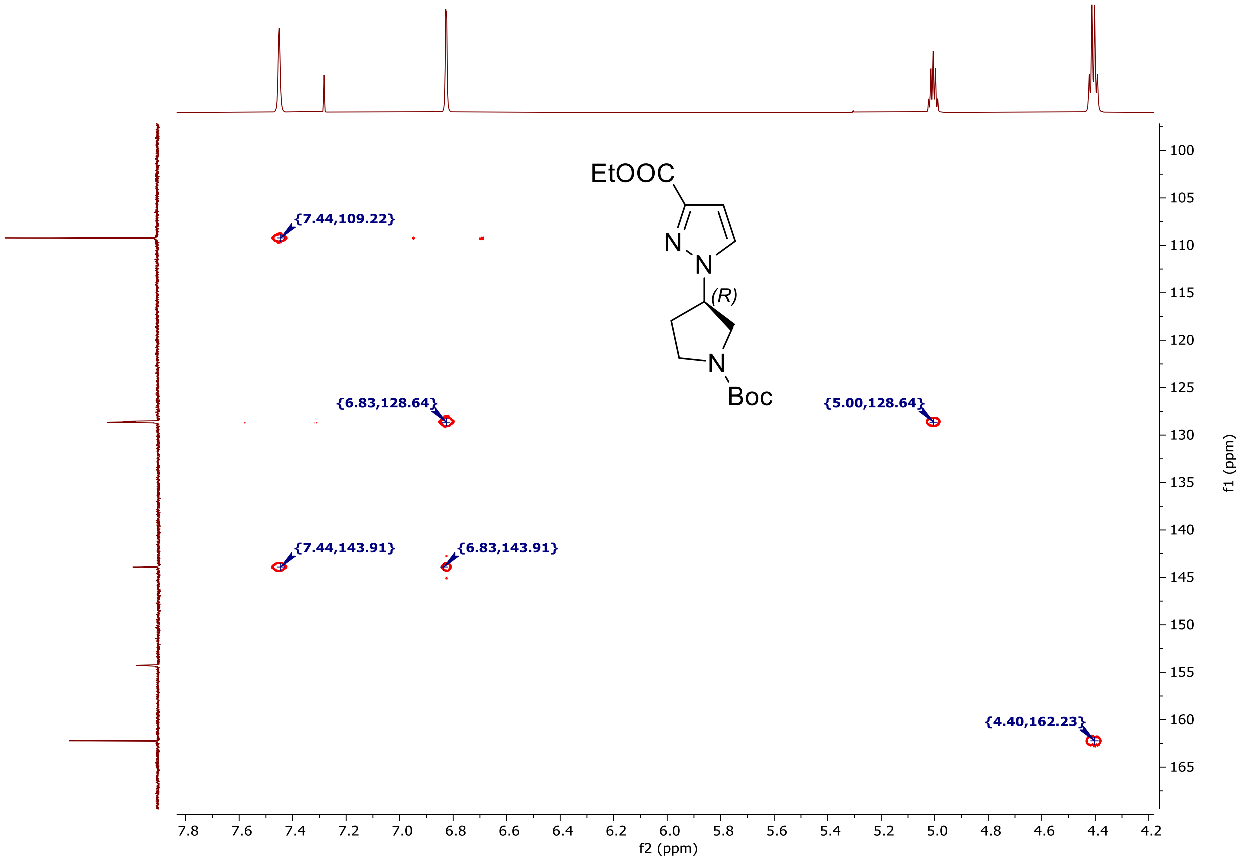

Figure S86. The 8Hz optimized ^{1}H-^{13}C HMBC NMR spectrum of ***(R)*-14b** ($CDCl_3$).

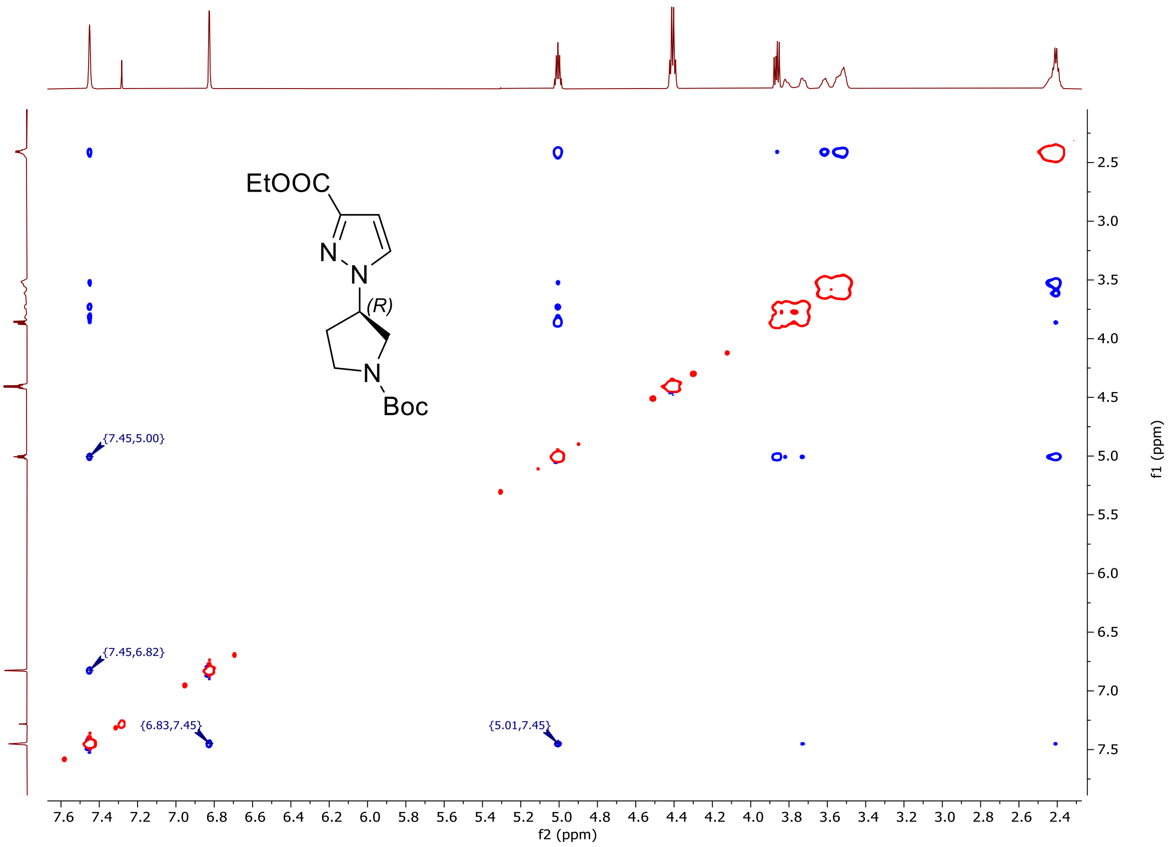

Figure S87. ^{1}H-^{1}H NOESY NMR spectrum of ***(R)*-14b** ($CDCl_3$).

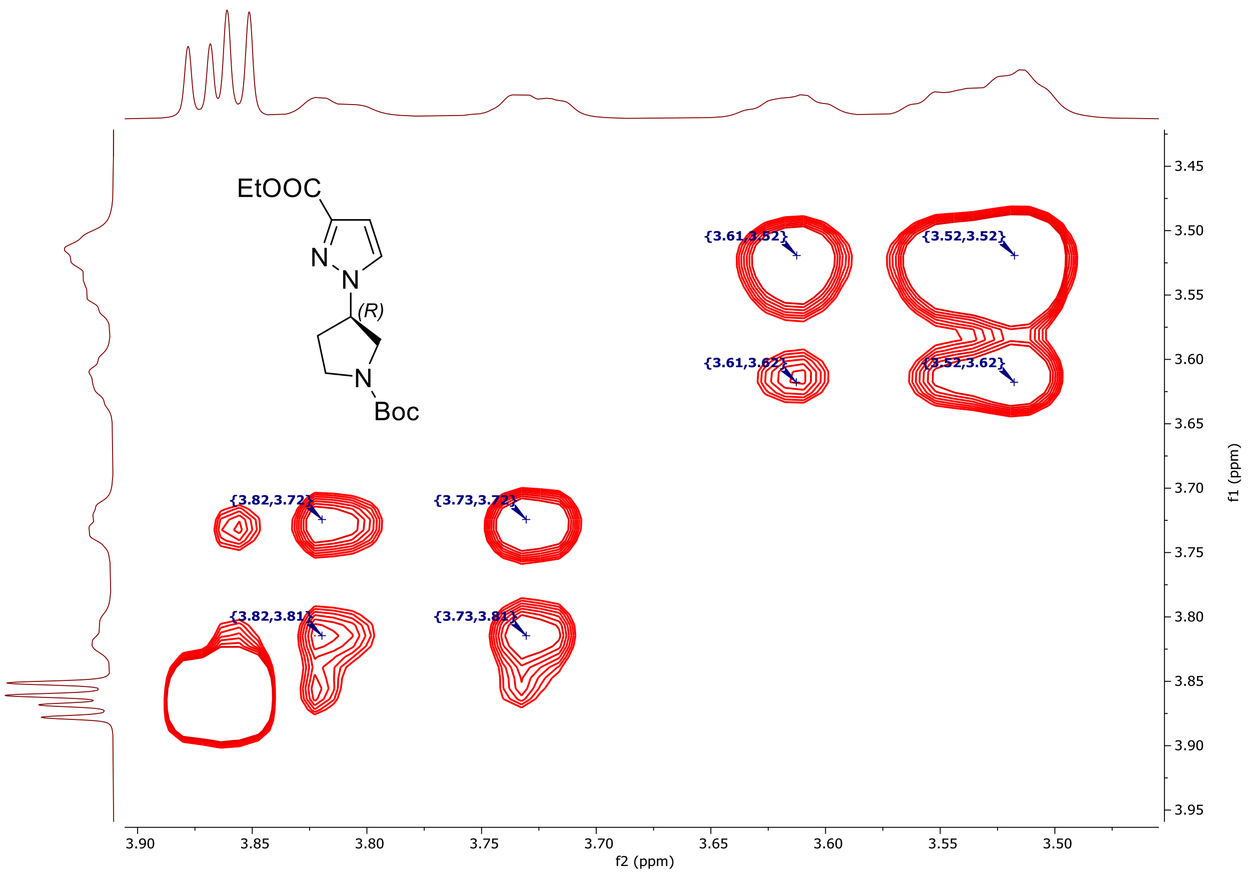

Figure S88. ^{1}H-^{1}H NOESY/EXSY NMR spectrum of ***(R)*-14b** ($CDCl_3$).

Acquisition Parameter

Source Type	ESI	Ion Polarity	Positive	Set Nebulizer	0.4 Bar
Focus	Not active	Set Capillary	4500 V	Set Dry Heater	180 °C
Scan Begin	50 m/z	Set End Plate Offset	-500 V	Set Dry Gas	4.0 l/min
Scan End	1000 m/z	Set Collision Cell RF	140.0 Vpp	Set Divert Valve	Waste

#	RT [min]	Area	Int. Type	I	S/N	Chromatogram	Max. m/z	FWHM [min]
n.a.	0.1	n.a.	Single spectrum	n.a.	n.a.	n.a.	304.2614	n.a.
n.a.	2.3	n.a.	Single spectrum	n.a.	n.a.	n.a.	332.1580	n.a.

+MS, 2.3min #140

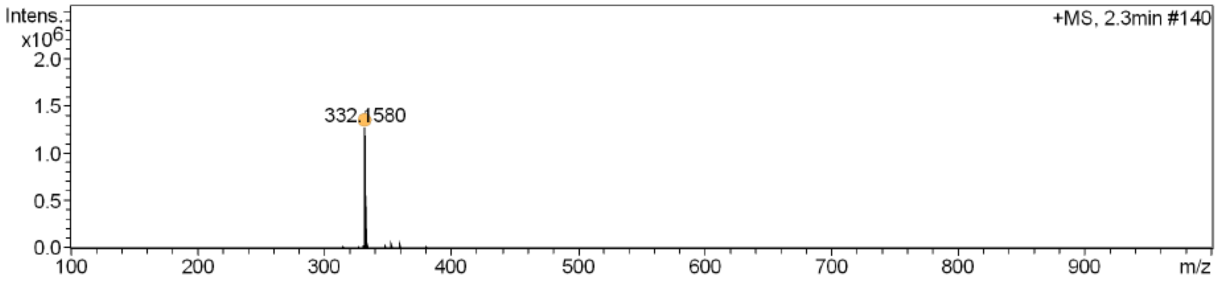

Meas. m/z	#	Ion Formula	m/z	err [ppm]	mSigma	# Sigma	Score	rdb	e¯ Conf	N-Rule
332.1580	1	C15H23N3NaO4	332.1581	-0.2	110.9	1	100.00	5.5	even	ok

Figure S89. HRMS (ESI$^+$) report of ***(R)*-10b**.

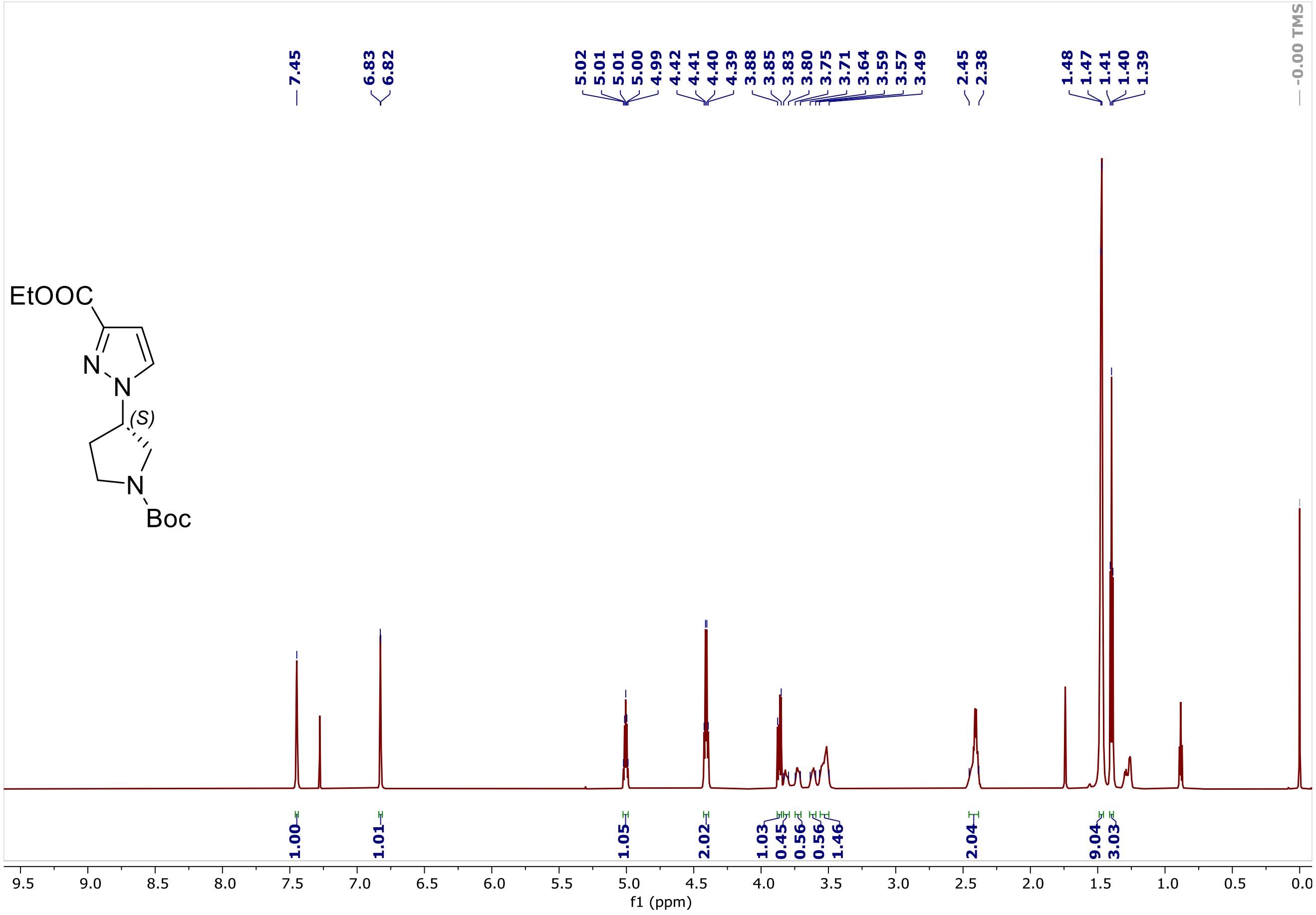

Figure S90. ^{1}H NMR (700 MHz, $CDCl_3$) spectrum of ***(S)*-14b**.

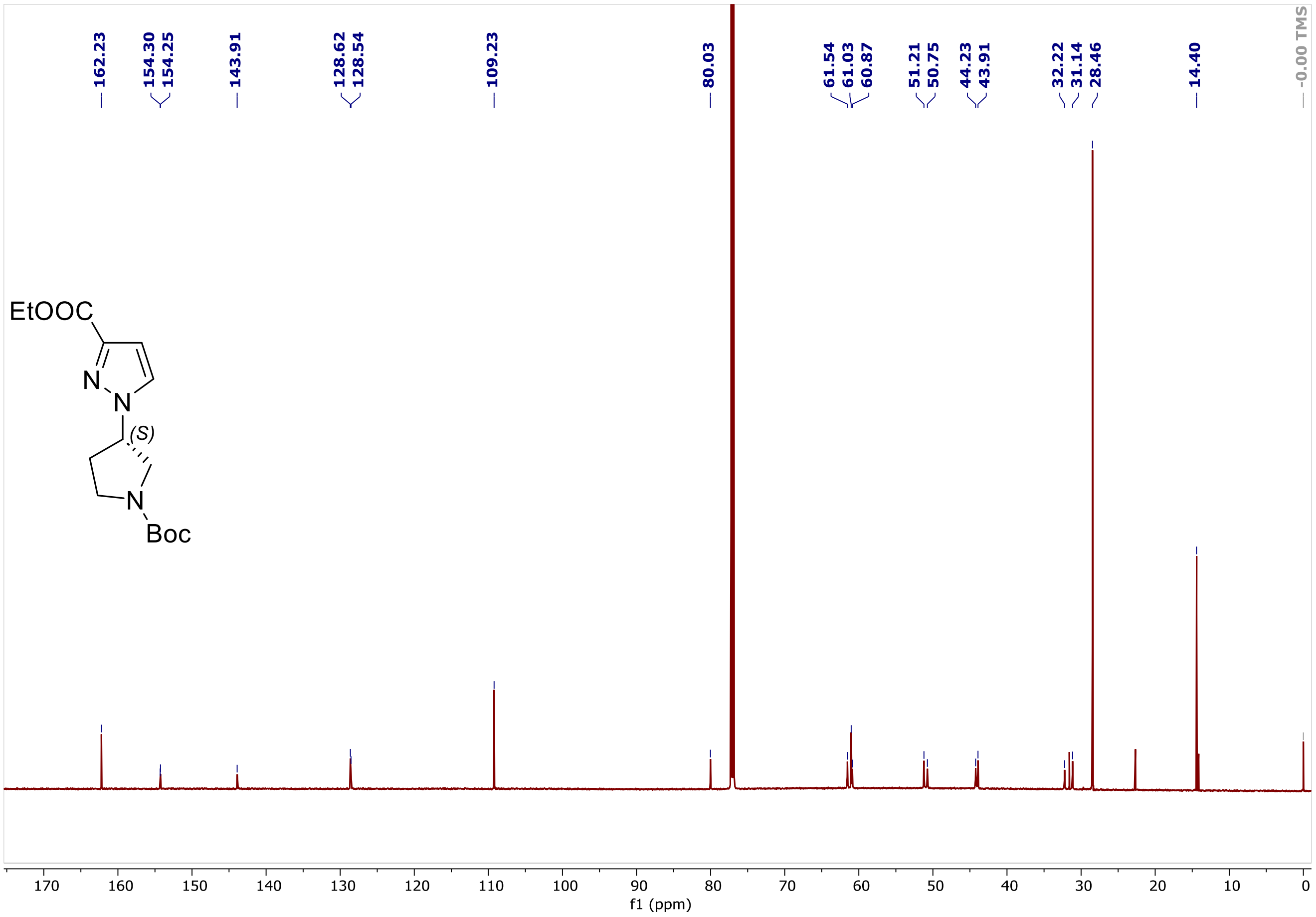

Figure S91. ^{13}C NMR (176 MHz, $CDCl_3$) spectrum of ***(S)*-14b**.

Acquisition Parameter

Source Type	ESI	Ion Polarity	Positive	Set Nebulizer	0.4 Bar
Focus	Not active	Set Capillary	4500 V	Set Dry Heater	180 °C
Scan Begin	50 m/z	Set End Plate Offset	-500 V	Set Dry Gas	4.0 l/min
Scan End	1000 m/z	Set Collision Cell RF	140.0 Vpp	Set Divert Valve	Waste

#	RT [min]	Area	Int. Type	I	S/N	Chromatogram	Max. m/z	FWHM [min]
n.a.	0.2	n.a.	Single spectrum	n.a.	n.a.	n.a.	304.2614	n.a.
n.a.	3.2	n.a.	Single spectrum	n.a.	n.a.	n.a.	332.1578	n.a.

+MS, 3.2min #194

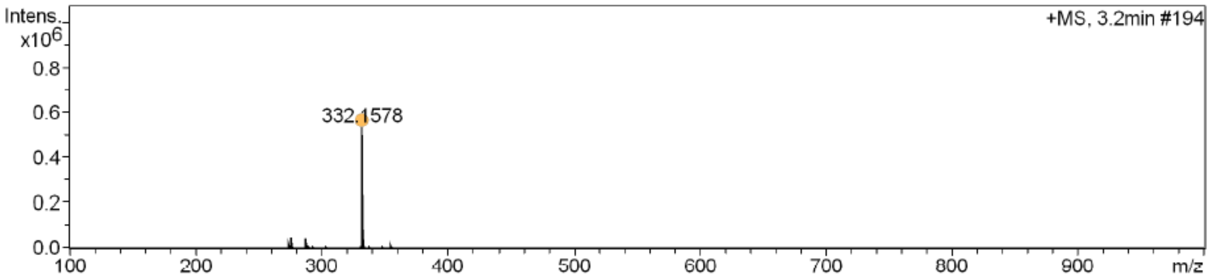

Meas. m/z	#	Ion Formula	m/z	err [ppm]	mSigma	# Sigma	Score	rdb	e⁻ Conf	N-Rule
332.1578	1	C15H23N3NaO4	332.1581	0.8	1.1	1	100.00	5.5	even	ok

Figure S92. HRMS (ESI$^+$) report of ***(S)*-14b**.

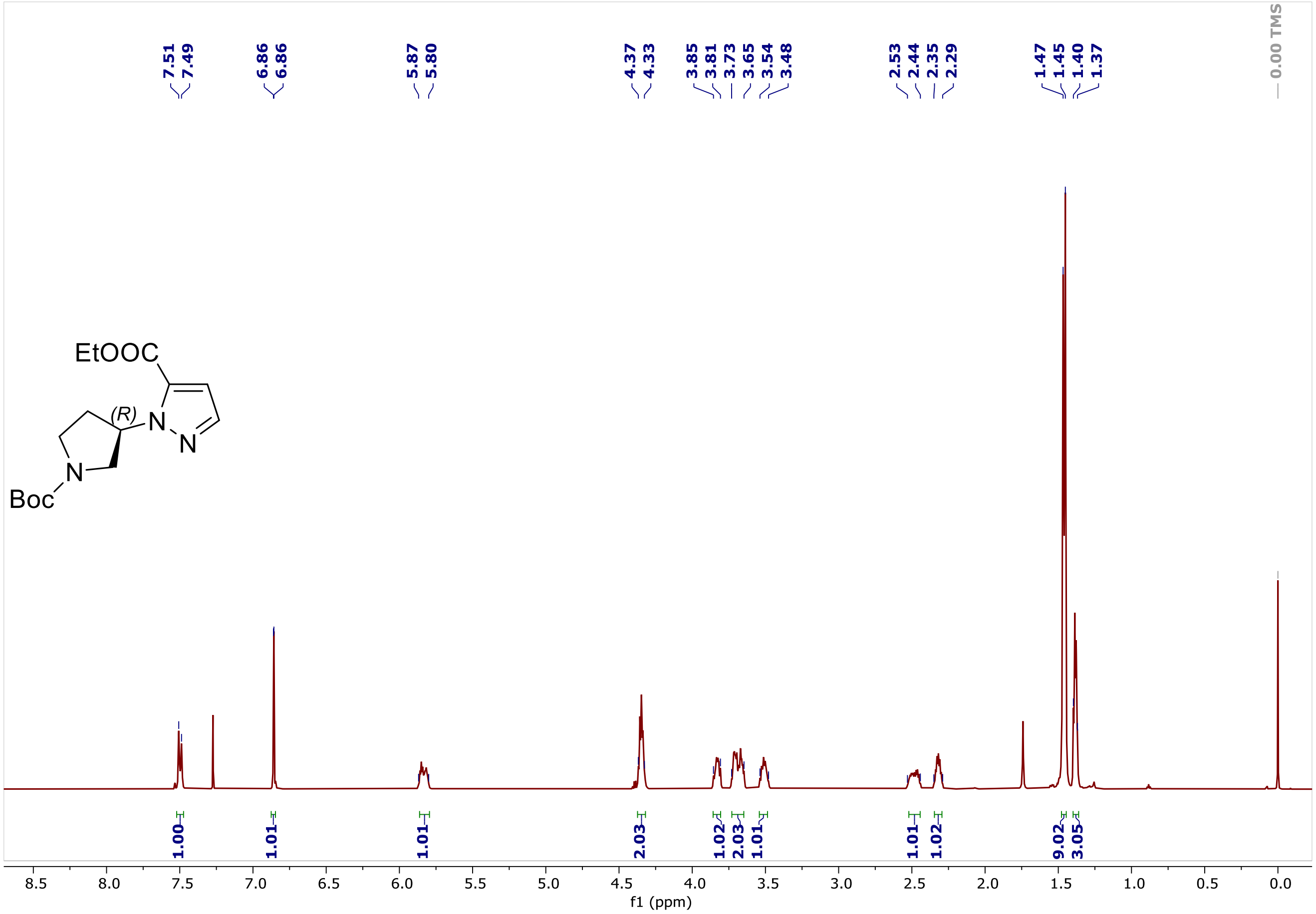

Figure S93. ^{1}H NMR (700 MHz, $CDCl_3$) spectrum of ***(R)*-15b**.

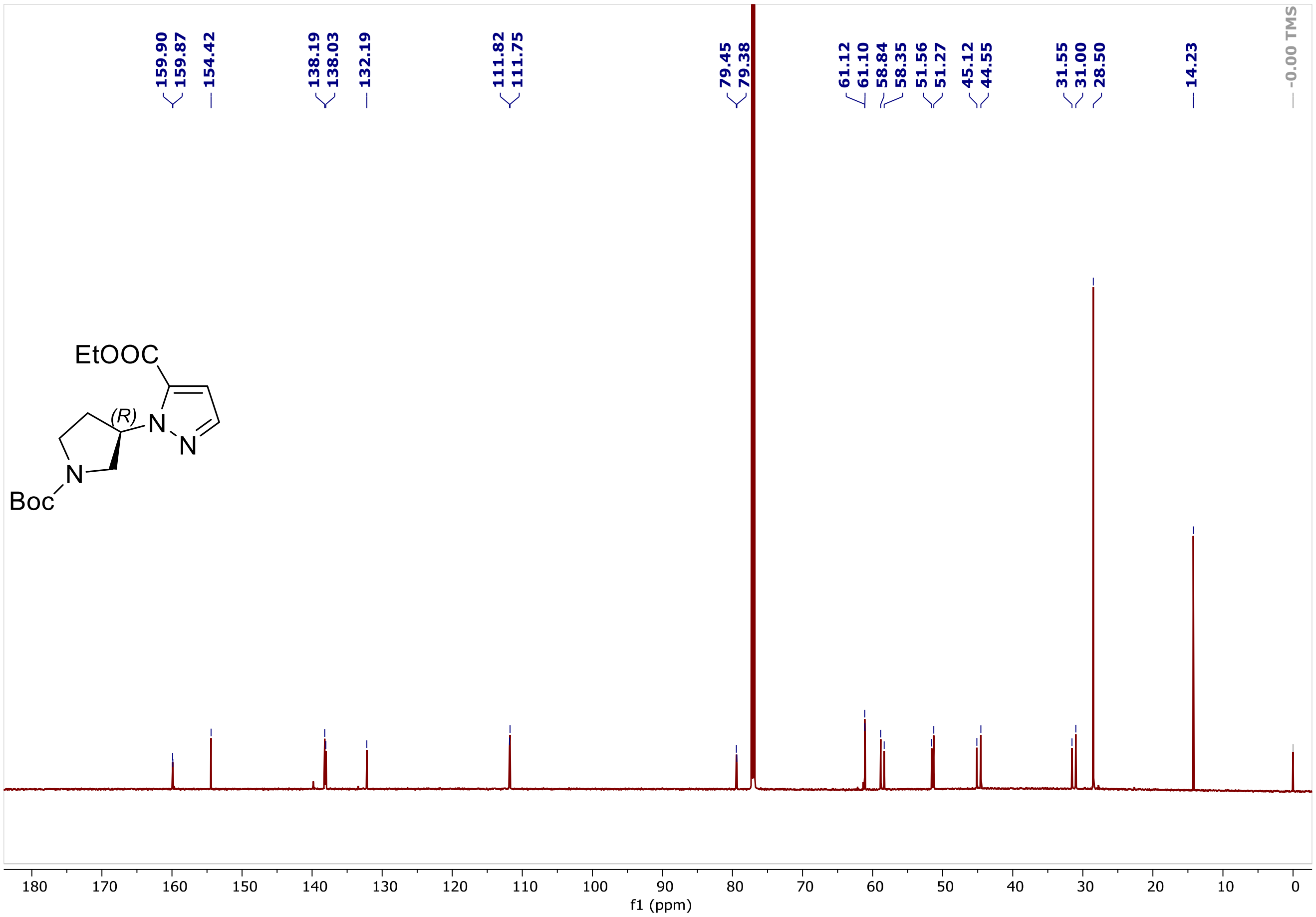

Figure S94. ^{13}C NMR (176 MHz, $CDCl_3$) spectrum of ***(R)*-15b**.

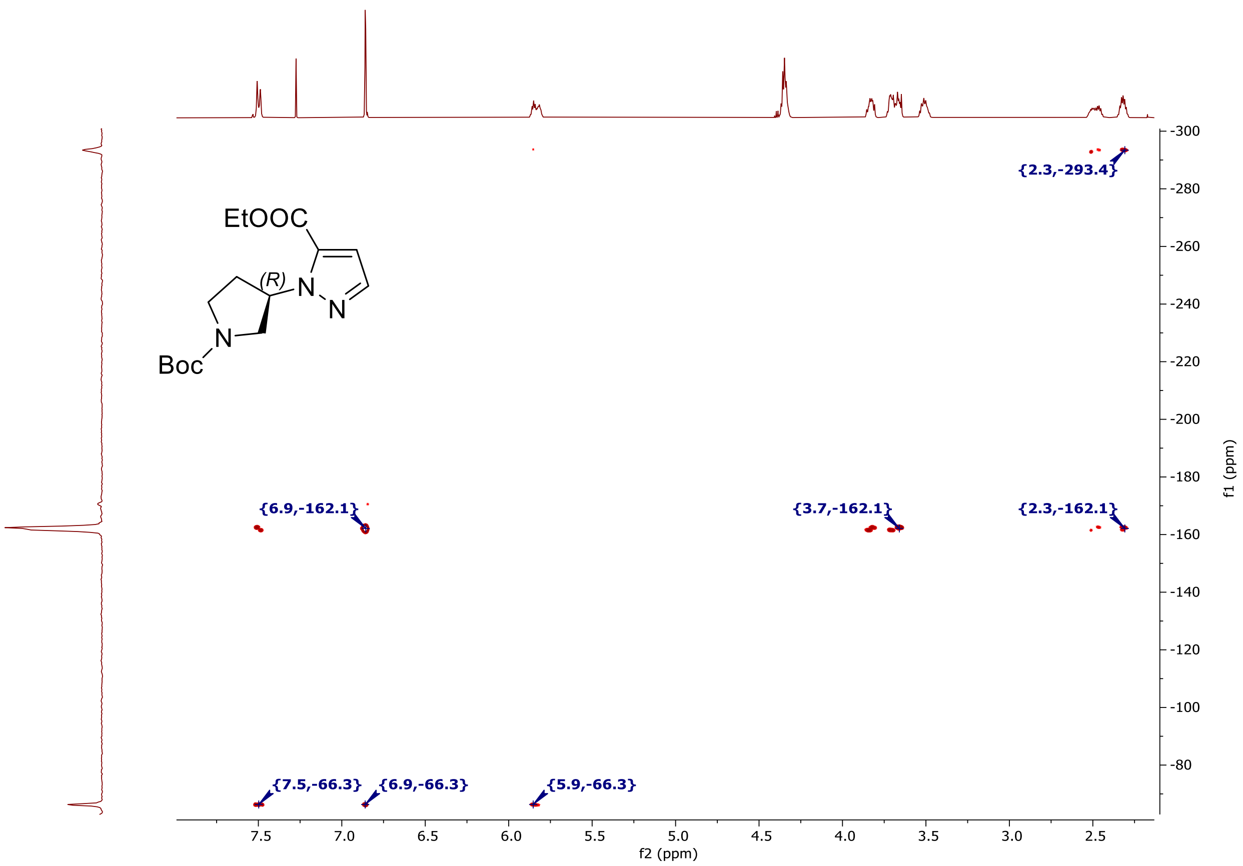

Figure S95. ^{1}H-^{15}N HMBC NMR spectrum of ***(R)*-15b** (71 MHz, $CDCl_3$).

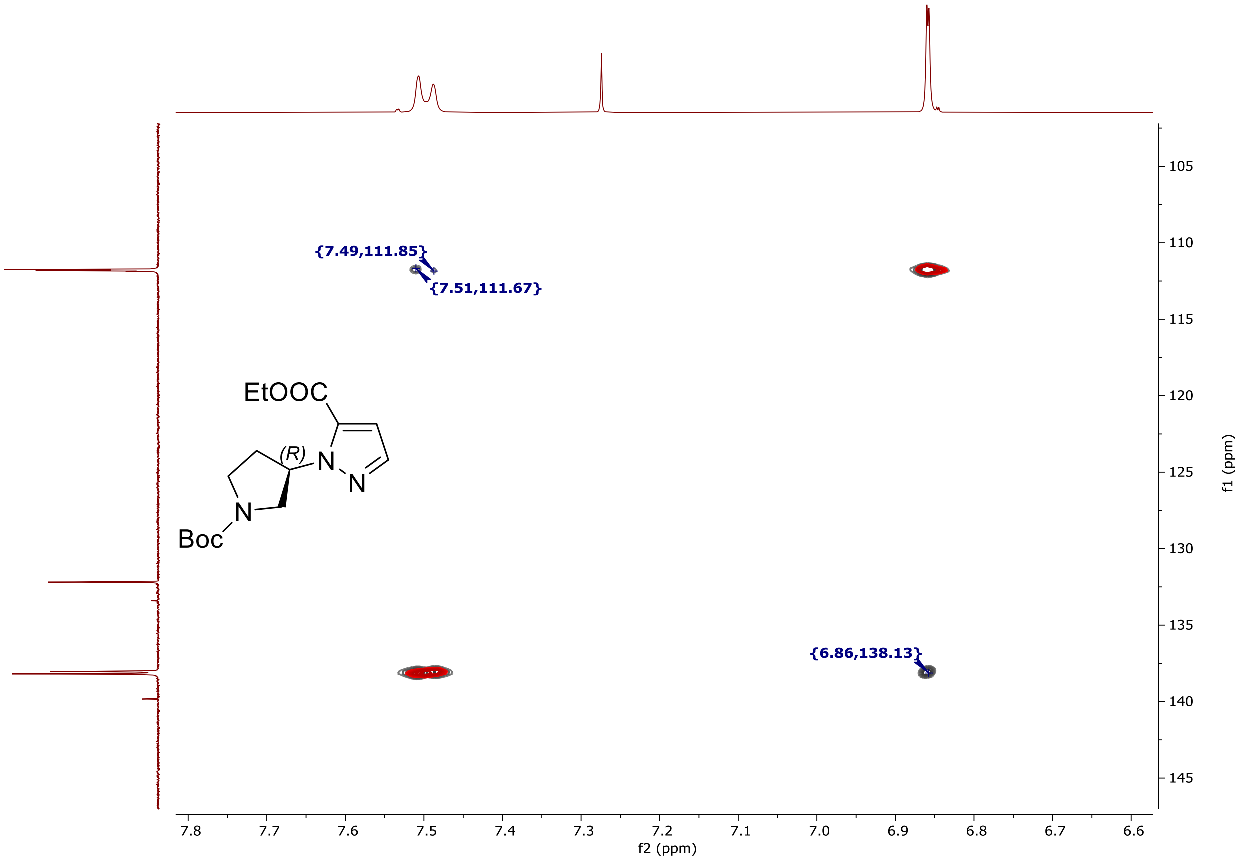

Figure S96. Overlaid multiplicity-edited ^{1}H-^{13}C HSQC (red correlations) and ^{1}H-^{13}C H2BC (black correlations) NMR spectra of ***(R)*-15b** ($CDCl_3$).

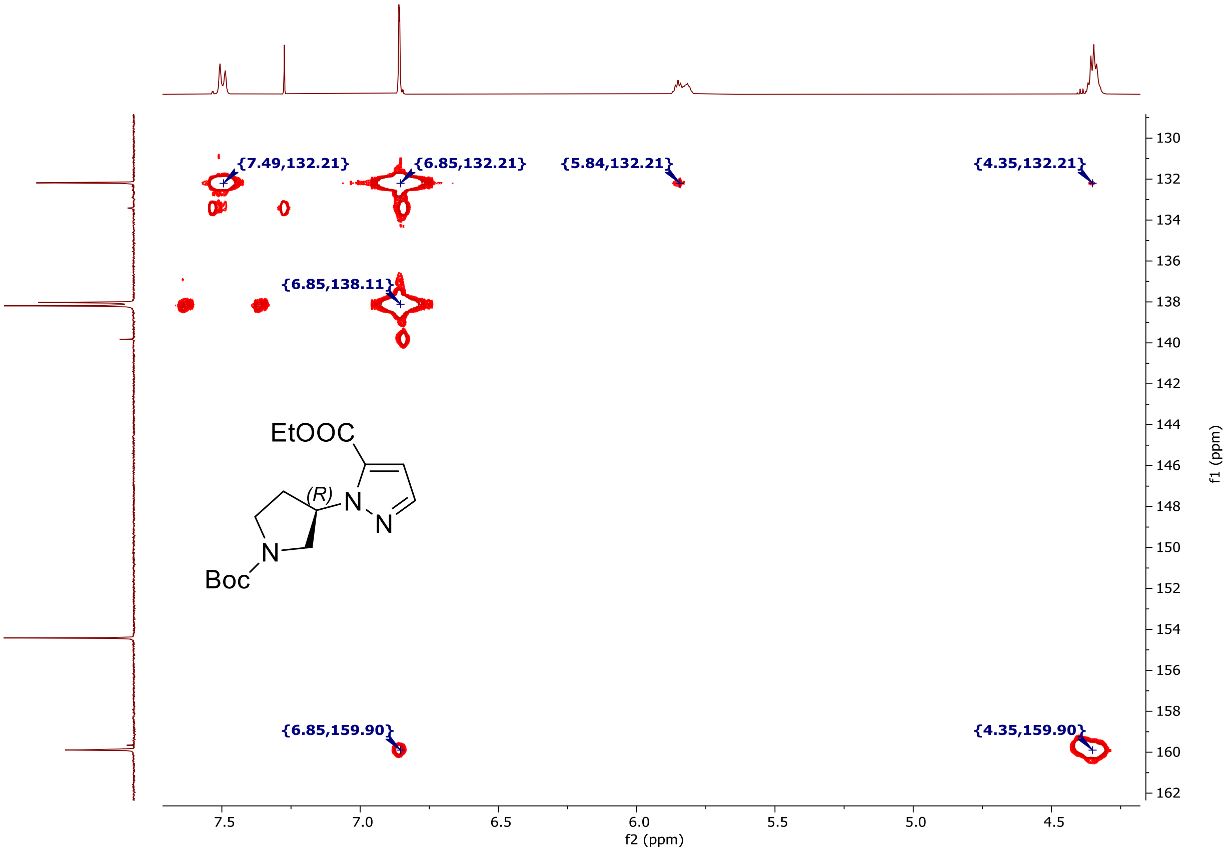

Figure S97. The 8Hz optimized ^{1}H-^{13}C HMBC NMR spectrum of ***(R)*-15b** ($CDCl_3$).

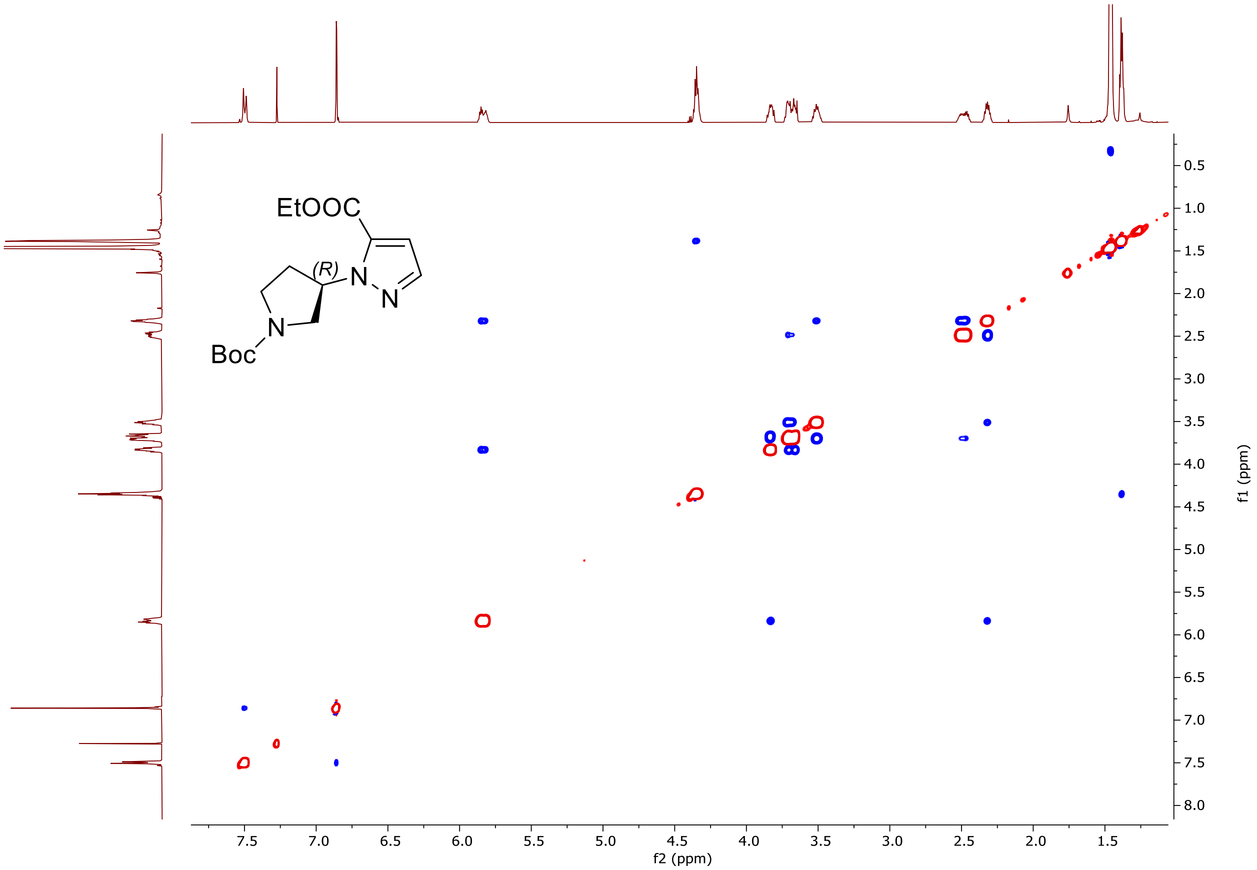

Figure S98. ^{1}H-^{1}H NOESY NMR spectrum of ***(R)*-15b** (CDCl$_3$).

Acquisition Parameter

Source Type	ESI	Ion Polarity	Positive	Set Nebulizer	0.4 Bar
Focus	Not active	Set Capillary	4500 V	Set Dry Heater	180 °C
Scan Begin	50 m/z	Set End Plate Offset	-500 V	Set Dry Gas	4.0 l/min
Scan End	1000 m/z	Set Collision Cell RF	140.0 Vpp	Set Divert Valve	Waste

#	RT [min]	Area	Int. Type	I	S/N	Chromatogram	Max. m/z	FWHM [min]
n.a.	0.2	n.a.	Single spectrum	n.a.	n.a.	n.a.	304.2618	n.a.
n.a.	2.9	n.a.	Single spectrum	n.a.	n.a.	n.a.	332.1581	n.a.

+MS, 2.9min #172

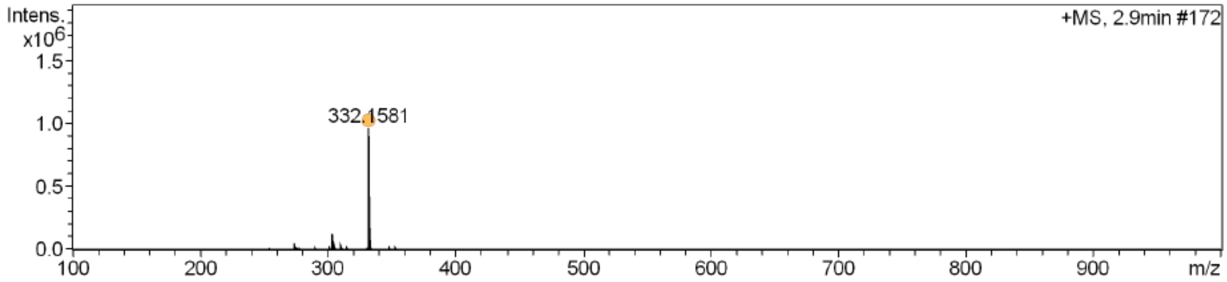

Meas. m/z	#	Ion Formula	m/z	err [ppm]	mSigma	# Sigma	Score	rdb	e⁻ Conf	N-Rule
332.1581	1	C15H23N3NaO4	332.1581	-0.2	9.5	1	100.00	5.5	even	ok

Figure S99. HRMS (ESI$^+$) report of ***(R)*-15b**.

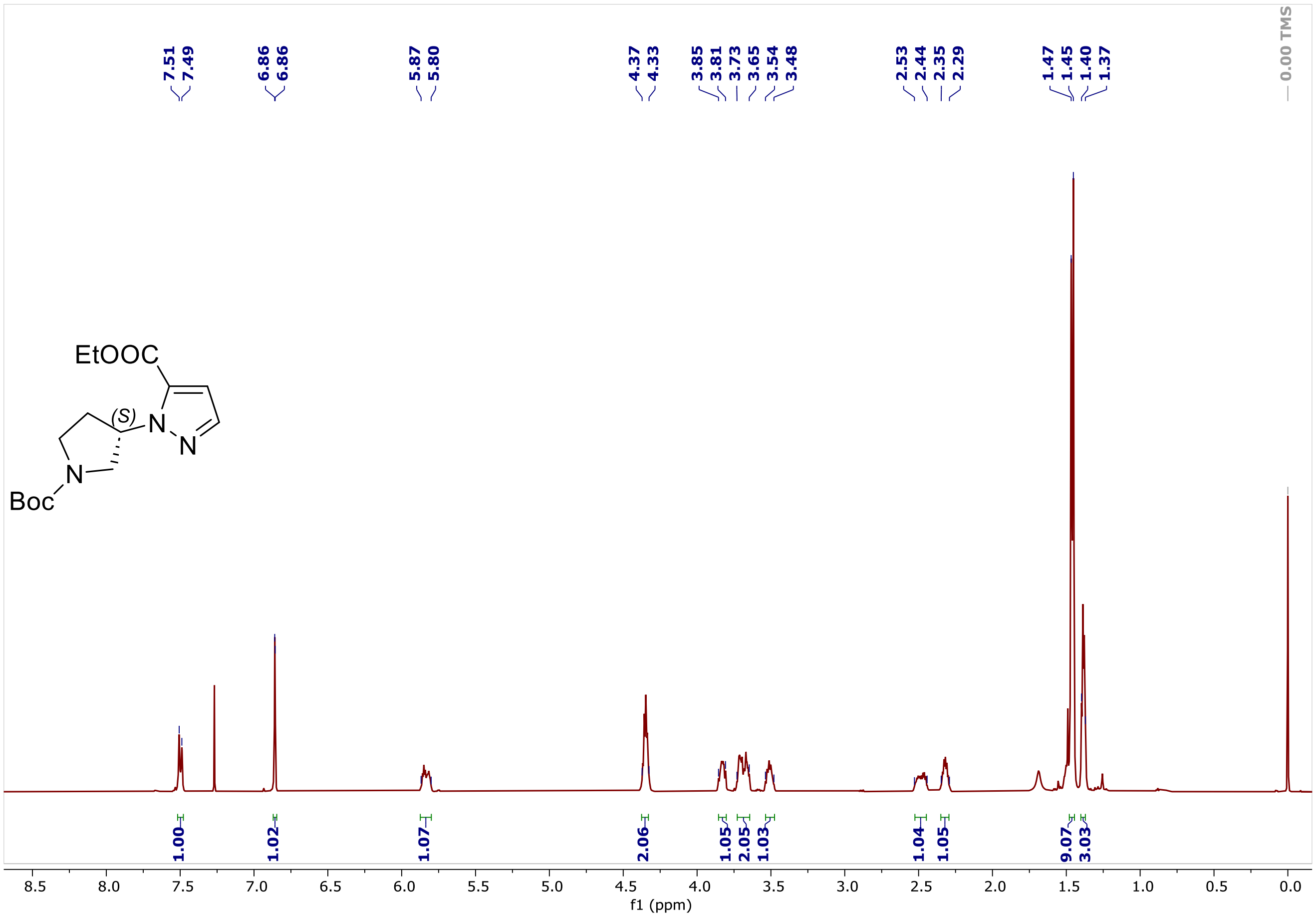

Figure S100. ^{1}H NMR (700 MHz, $CDCl_3$) spectrum of ***(S)*-15b**.

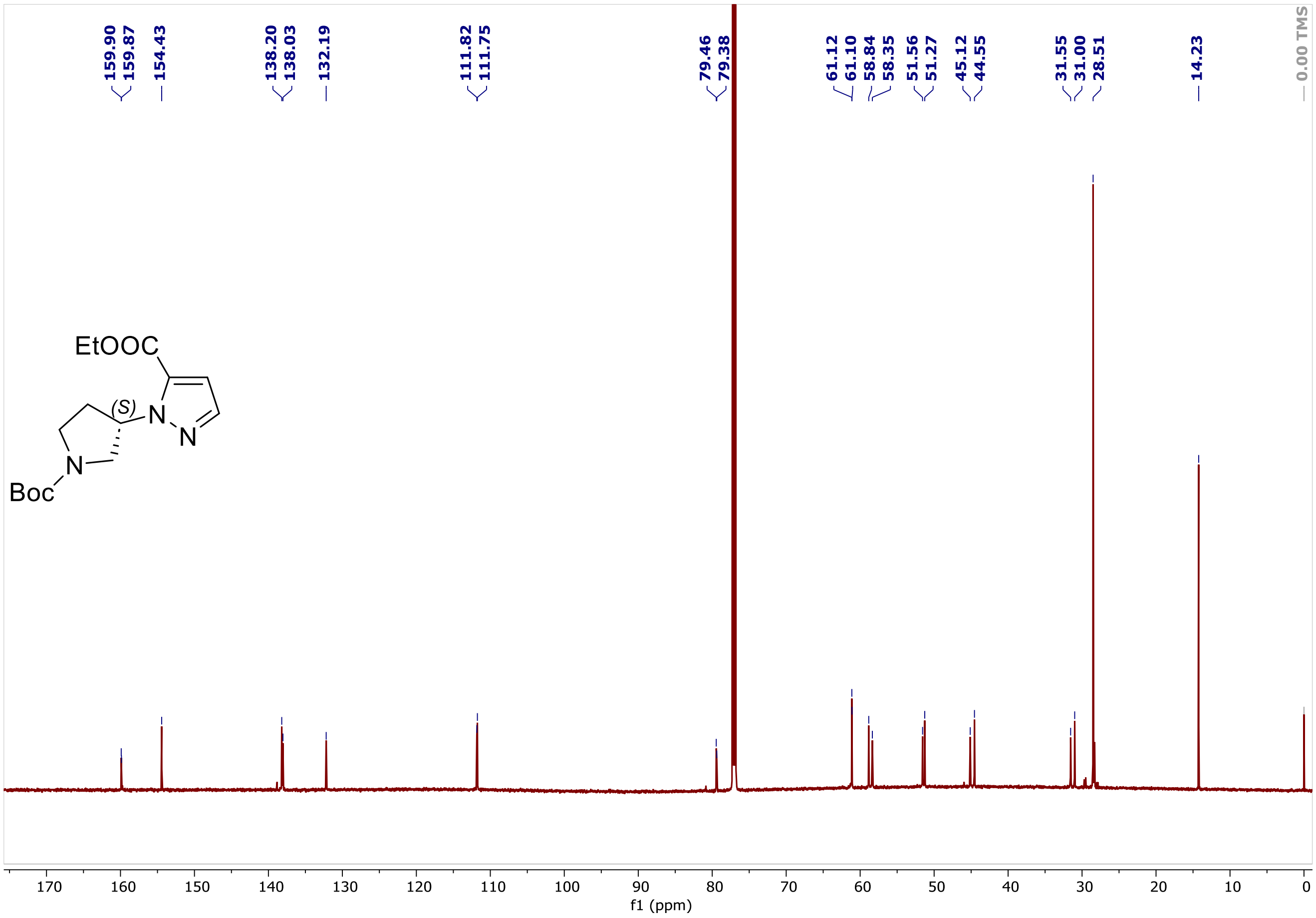

Figure S101. ^{13}C NMR (176 MHz, $CDCl_3$) spectrum of ***(S)*-15b**.

Acquisition Parameter

Source Type	ESI	Ion Polarity	Positive	Set Nebulizer	0.4 Bar
Focus	Not active	Set Capillary	4500 V	Set Dry Heater	180 °C
Scan Begin	50 m/z	Set End Plate Offset	-500 V	Set Dry Gas	4.0 l/min
Scan End	1000 m/z	Set Collision Cell RF	140.0 Vpp	Set Divert Valve	Waste

#	RT [min]	Area	Int. Type	I	S/N	Chromatogram	Max. m/z	FWHM [min]
n.a.	0.6	n.a.	Single spectrum	n.a.	n.a.	n.a.	304.2614	n.a.
n.a.	5.4	n.a.	Single spectrum	n.a.	n.a.	n.a.	332.1577	n.a.

+MS, 5.4min #324

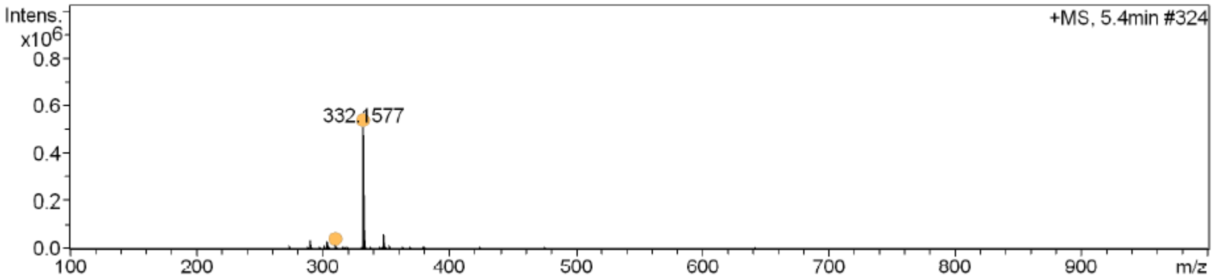

Meas. m/z	#	Ion Formula	m/z	err [ppm]	mSigma	# Sigma	Score	rdb	e⁻ Conf	N-Rule
310.1762	1	C15H24N3O4	310.1761	-0.4	127.3	1	100.00	5.5	even	ok
332.1577	1	C15H23N3NaO4	332.1581	1.1	2.9	1	100.00	5.5	even	ok

Figure S102. HRMS (ESI^+) report of **(*S*)-15b**.

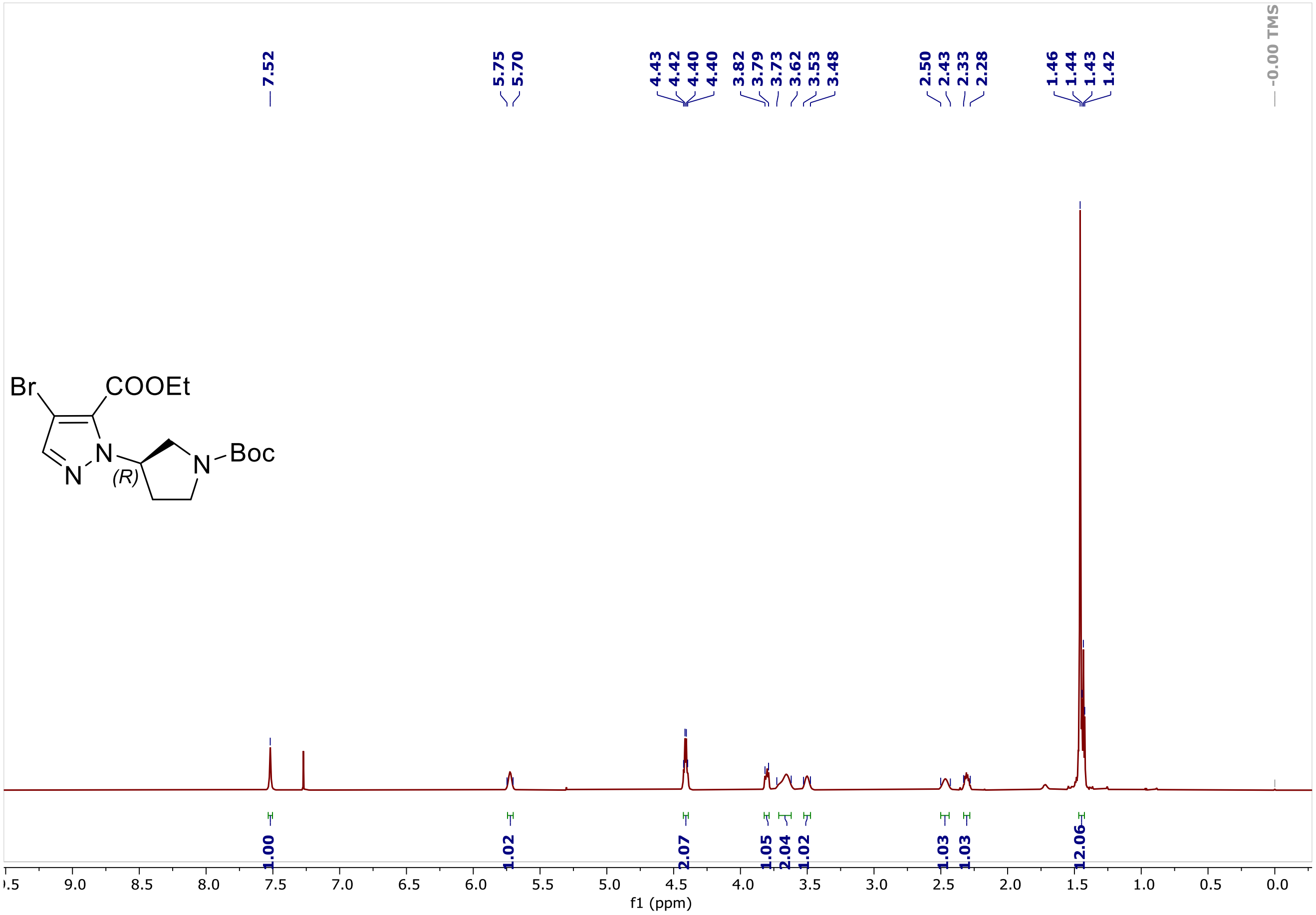

Figure S103. ^{1}H NMR (700 MHz, $CDCl_3$) spectrum of ***(R)*-16**.

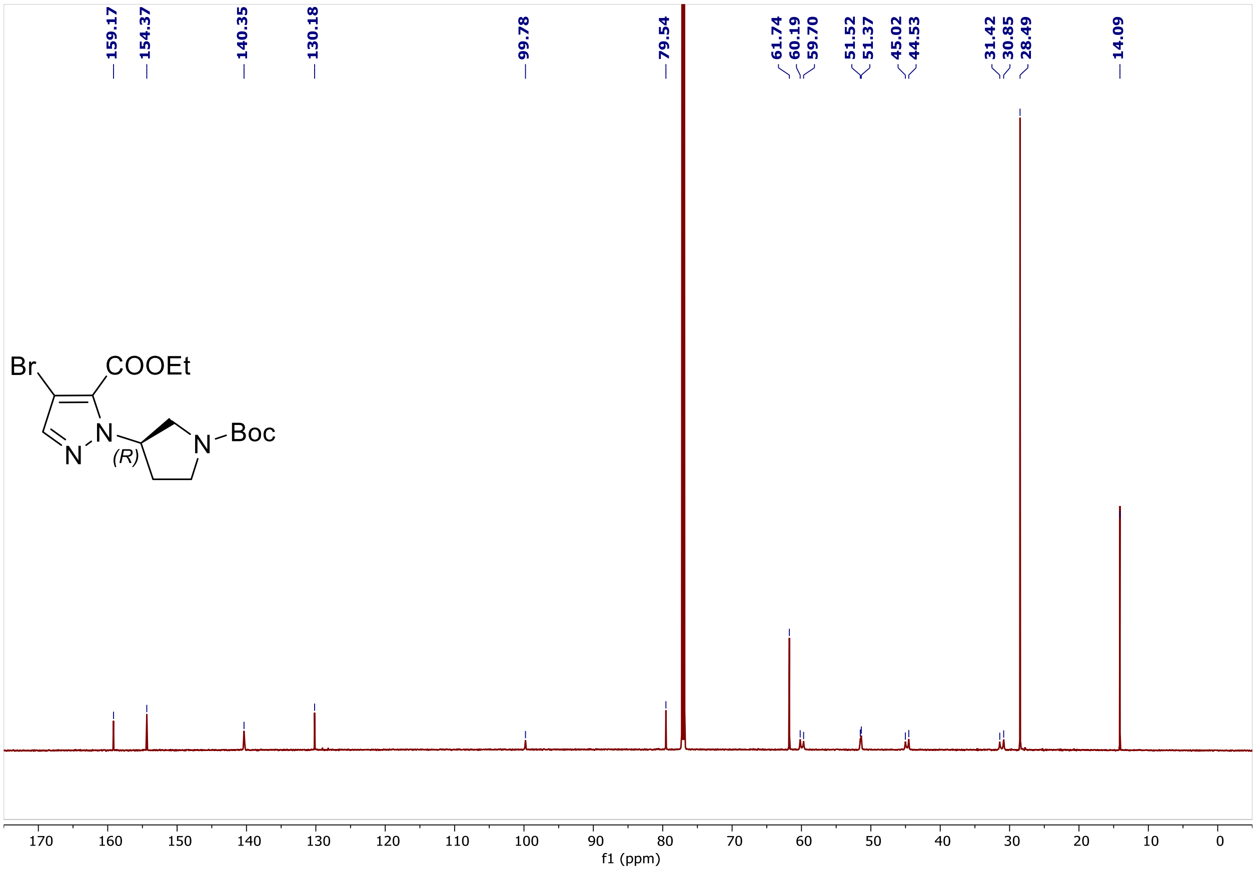

Figure S104. ^{13}C NMR (176 MHz, $CDCl_3$) spectrum of ***(R)*-16**.

Acquisition Parameter

Source Type	ESI	Ion Polarity	Positive	Set Nebulizer	0.4 Bar
Focus	Not active	Set Capillary	4500 V	Set Dry Heater	180 °C
Scan Begin	50 m/z	Set End Plate Offset	-500 V	Set Dry Gas	4.0 l/min
Scan End	1000 m/z	Set Collision Cell RF	140.0 Vpp	Set Divert Valve	Waste

#	RT [min]	Area	Int. Type	I	S/N	Chromatogram	Max. m/z	FWHM [min]
n.a.	0.2	n.a.	Single spectrum	n.a.	n.a.	n.a.	304.2622	n.a.
n.a.	7.8	n.a.	Single spectrum	n.a.	n.a.	n.a.	410.0686	n.a.

+MS, 7.8min #466

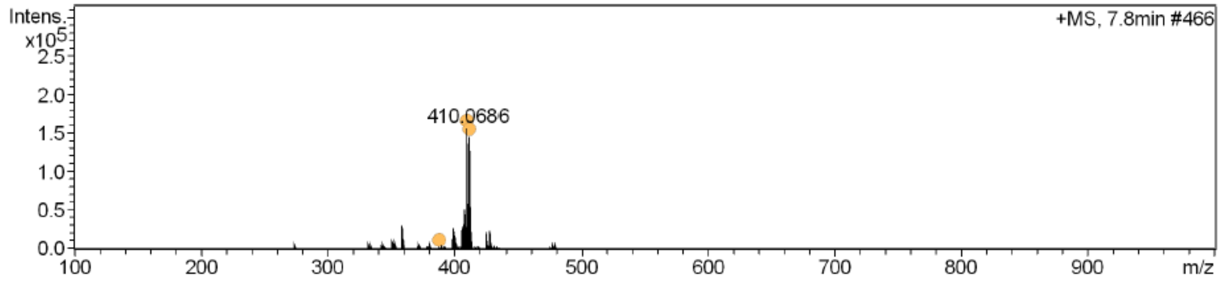

Meas. m/z	#	Ion Formula	m/z	err [ppm]	mSigma	# Sigma	Score	rdb	e⁻ Conf	N-Rule
388.0856	1	C15H23BrN3O4	388.0866	-2.6	105.3	1	100.00	5.5	even	ok
	1	C15H23BrN3O4	388.0866	-2.6	105.3	1	100.00	5.5	even	ok
410.0686	1	C15H22BrN3NaO4	410.0686	-0.0	29.6	1	100.00	5.5	even	ok
412.0668	1	C15H22BrN3NaO4	410.0686	0.2	29.6	1	100.00	5.5	even	ok

Figure S105. HRMS (ESI^+) report of ***(R)*-16**.

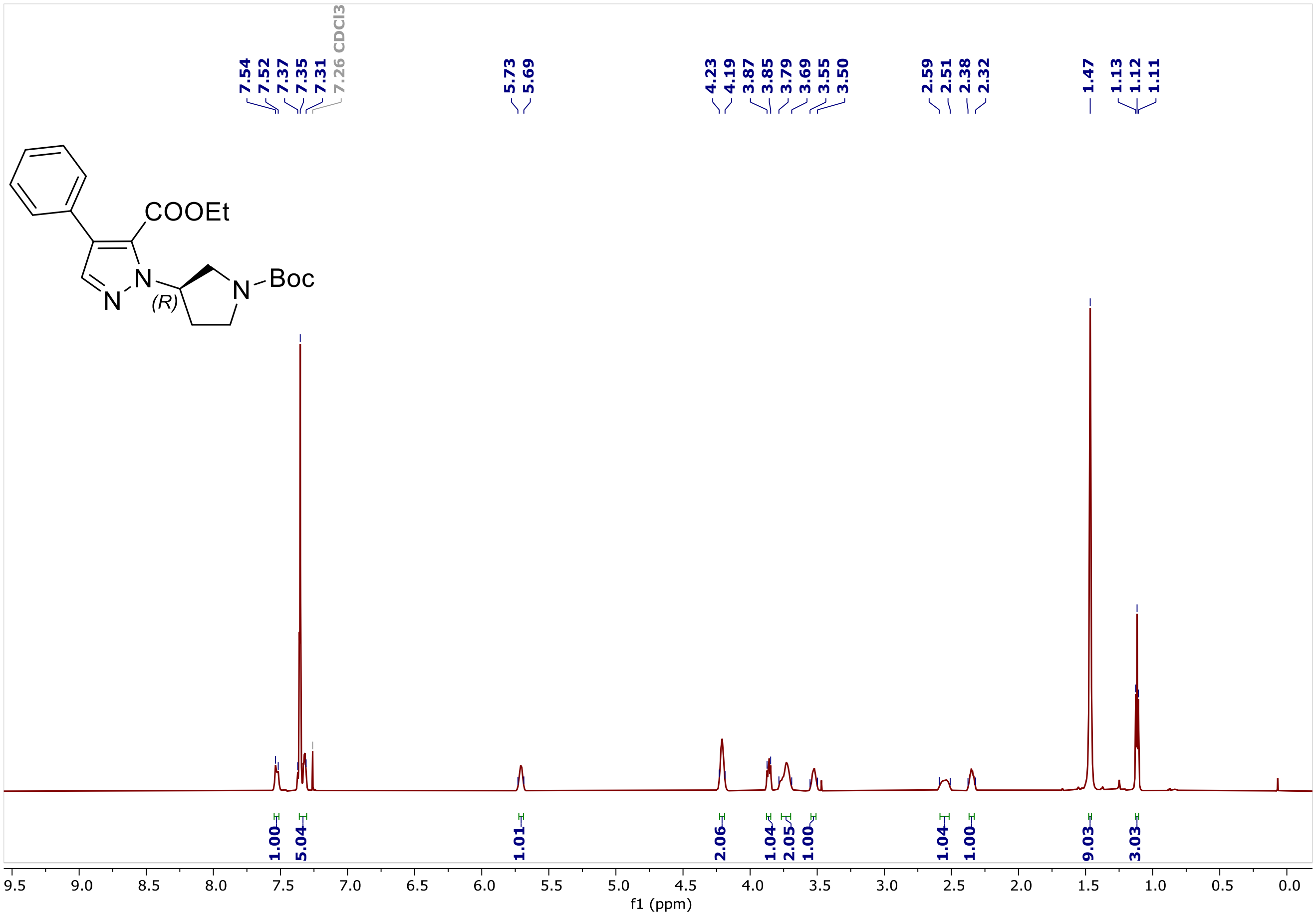

Figure S106. ^{1}H NMR (700 MHz, $CDCl_3$) spectrum of ***(R)*-17a**.

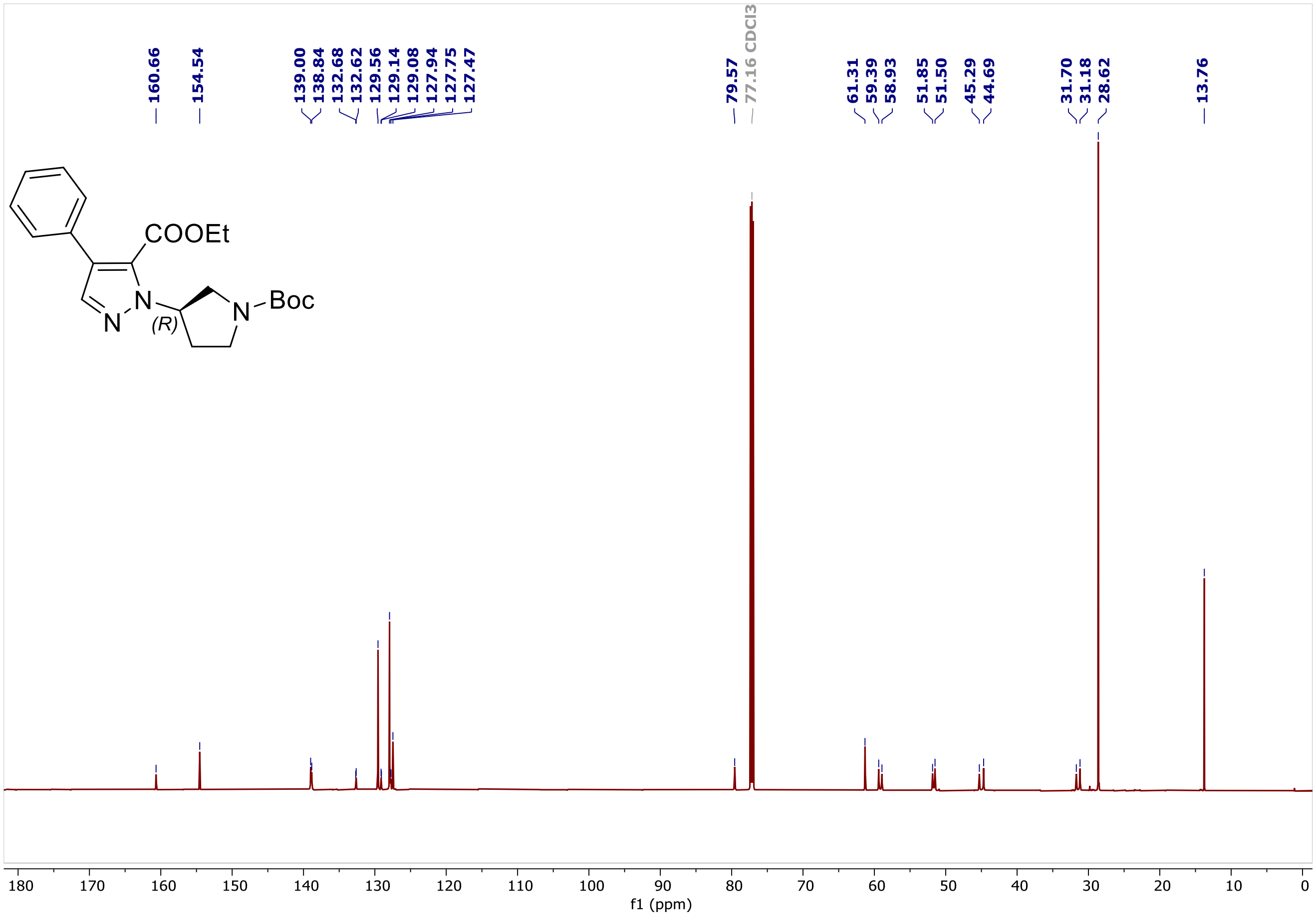

Figure S107. ^{13}C NMR (176 MHz, $CDCl_3$) spectrum of ***(R)*-17a**.

Acquisition Parameter

Source Type	ESI	Ion Polarity	Positive	Set Nebulizer	0.4 Bar		
Focus	Not active	Set Capillary	4500 V	Set Dry Heater	180 °C		
Scan Begin	50 m/z	Set End Plate Offset	-500 V	Set Dry Gas	4.0 l/min		
Scan End	1000 m/z	Set Collision Cell RF	140.0 Vpp	Set Divert Valve	Waste		

#	RT [min]	Area	Int. Type	I	S/N	Chromatogram	Max. m/z	FWHM [min]
n.a.	0.6	n.a.	Single spectrum	n.a.	n.a.	n.a.	304.2612	n.a.
n.a.	3.8	n.a.	Single spectrum	n.a.	n.a.	n.a.	408.1888	n.a.

+MS, 3.8min #226

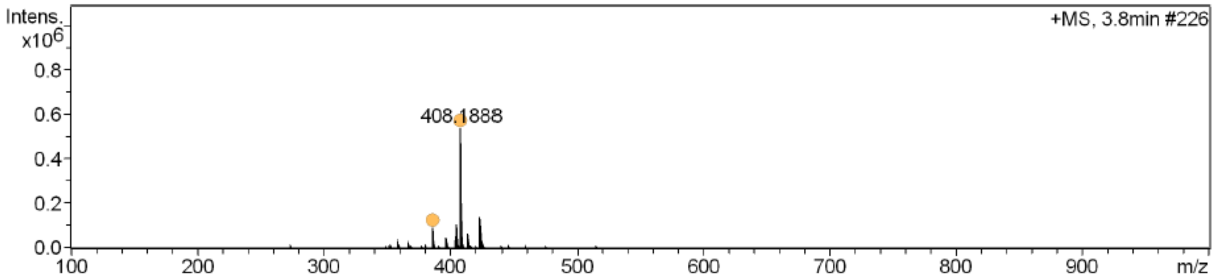

Meas. m/z	#	Ion Formula	m/z	err [ppm]	mSigma	# Sigma	Score	rdb	e⁻ Conf	N-Rule
386.2059	1	C21H28N3O4	386.2074	4.1	6.9	1	100.00	9.5	even	ok
408.1888	1	C21H27N3NaO4	408.1894	1.3	9.8	1	100.00	9.5	even	ok

Figure S108. HRMS (ESI^+) report of **(*R*)-17a**.

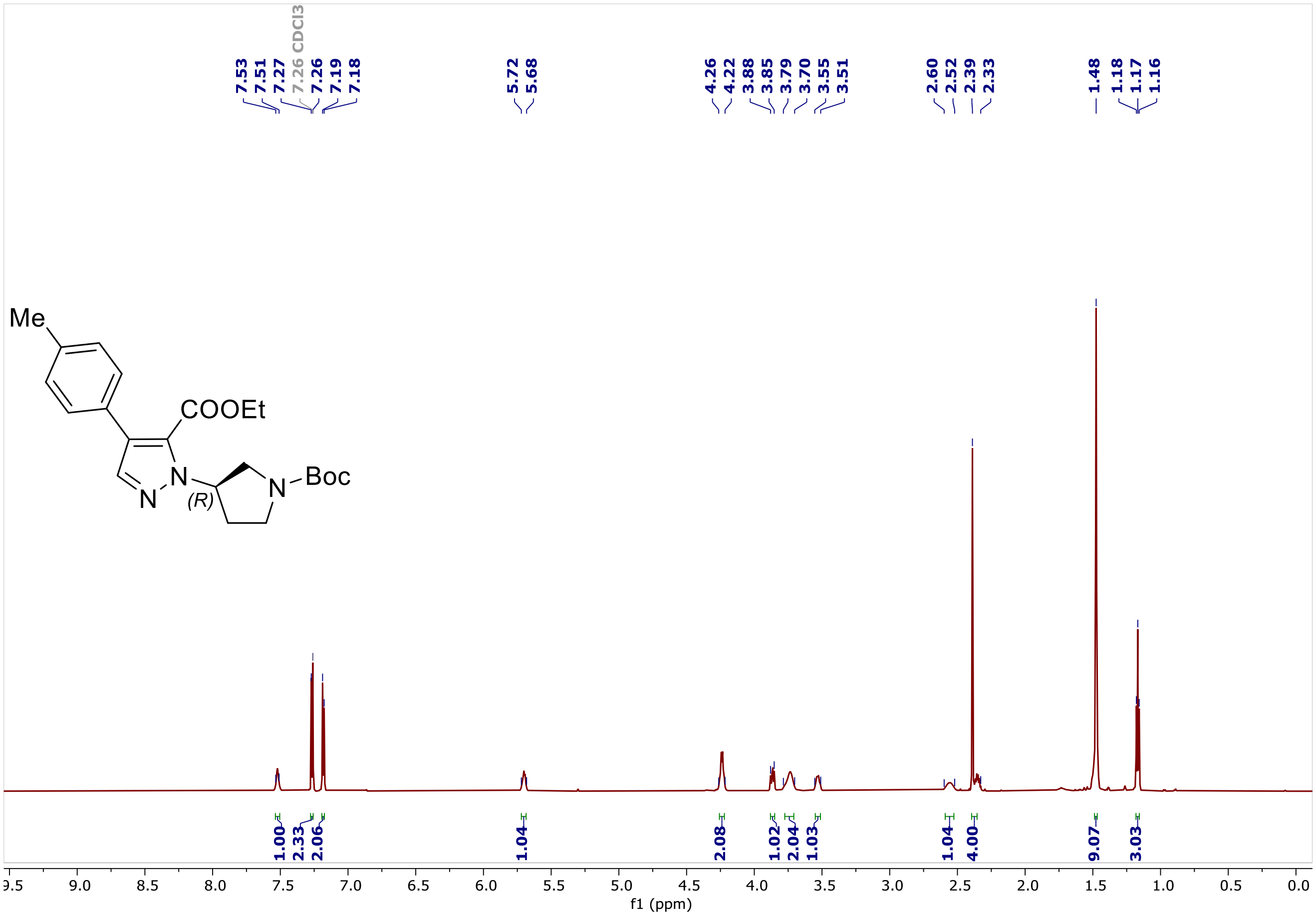

Figure S109. ^{1}H NMR (700 MHz, $CDCl_3$) spectrum of ***(R)*-17b**.

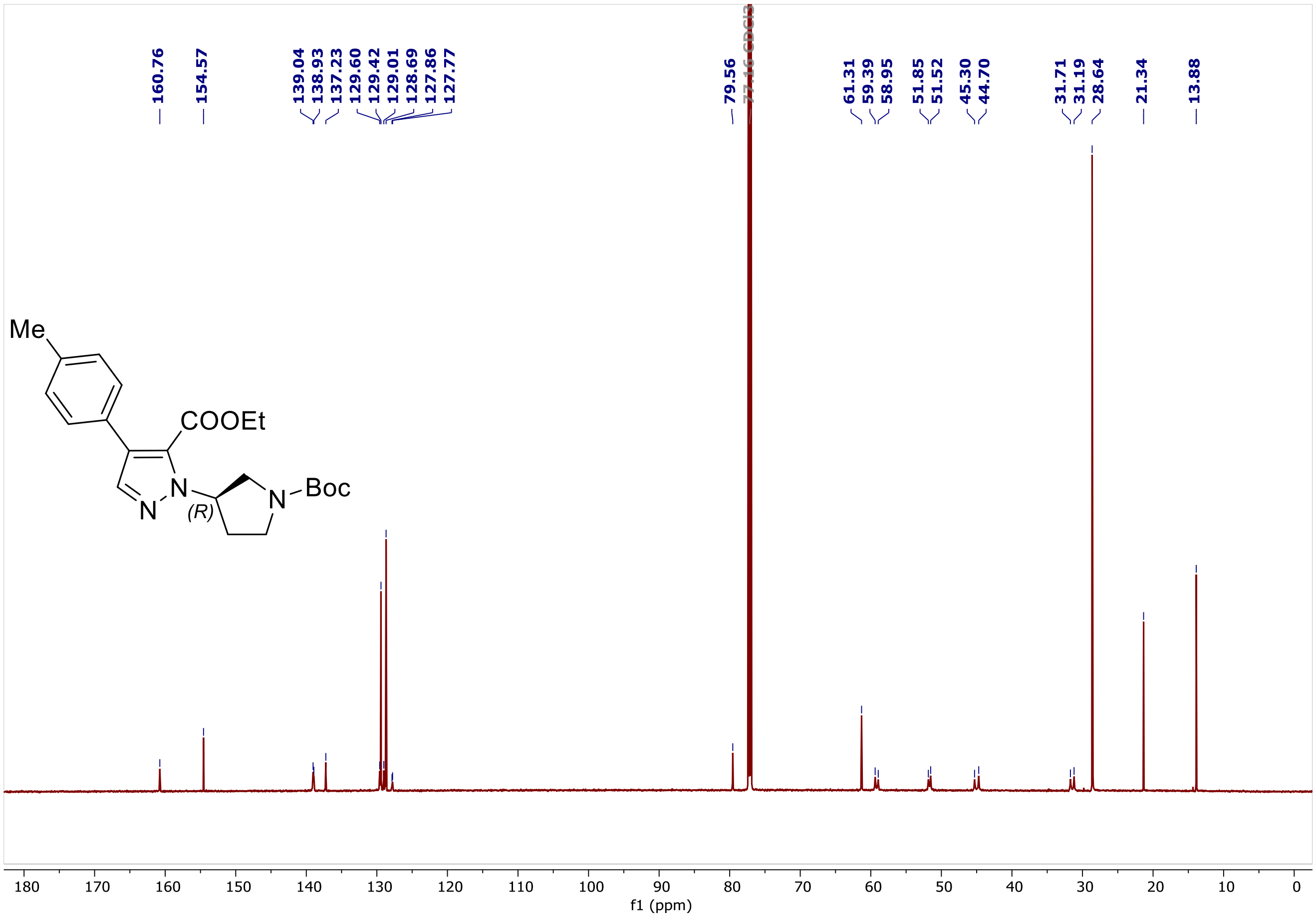

Figure S110. ^{13}C NMR (176 MHz, $CDCl_3$) spectrum of ***(R)*-17b**.

Acquisition Parameter

Source Type	ESI	Ion Polarity	Positive	Set Nebulizer	0.4 Bar
Focus	Not active	Set Capillary	4500 V	Set Dry Heater	180 °C
Scan Begin	50 m/z	Set End Plate Offset	-500 V	Set Dry Gas	4.0 l/min
Scan End	1000 m/z	Set Collision Cell RF	140.0 Vpp	Set Divert Valve	Waste

#	RT [min]	Area	Int. Type	I	S/N	Chromatogram	Max. m/z	FWHM [min]
n.a.	0.1	n.a.	Single spectrum	n.a.	n.a.	n.a.	304.2621	n.a.
n.a.	7.4	n.a.	Single spectrum	n.a.	n.a.	n.a.	422.2052	n.a.

+MS, 7.4min #446

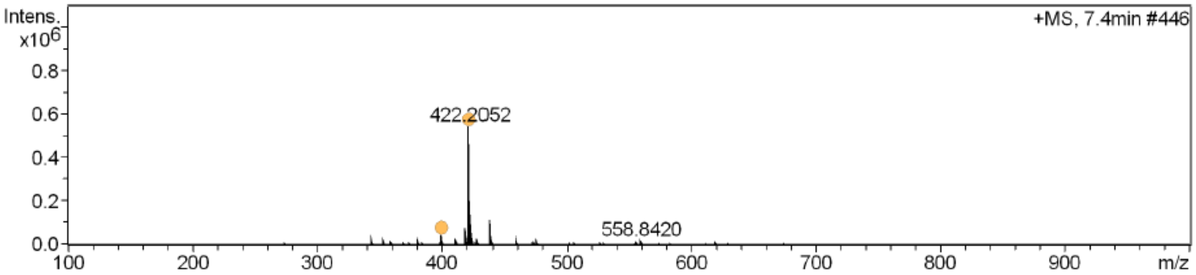

Meas. m/z	#	Ion Formula	m/z	err [ppm]	mSigma	# Sigma	Score	rdb	e⁻ Conf	N-Rule
400.2222	1	C22H30N3O4	400.2231	2.3	10.6	1	100.00	9.5	even	ok
422.2052	1	C22H29N3NaO4	422.2050	-0.3	16.3	1	100.00	9.5	even	ok

Figure S111. HRMS (ESI^+) report of ***(R)*-17b**.

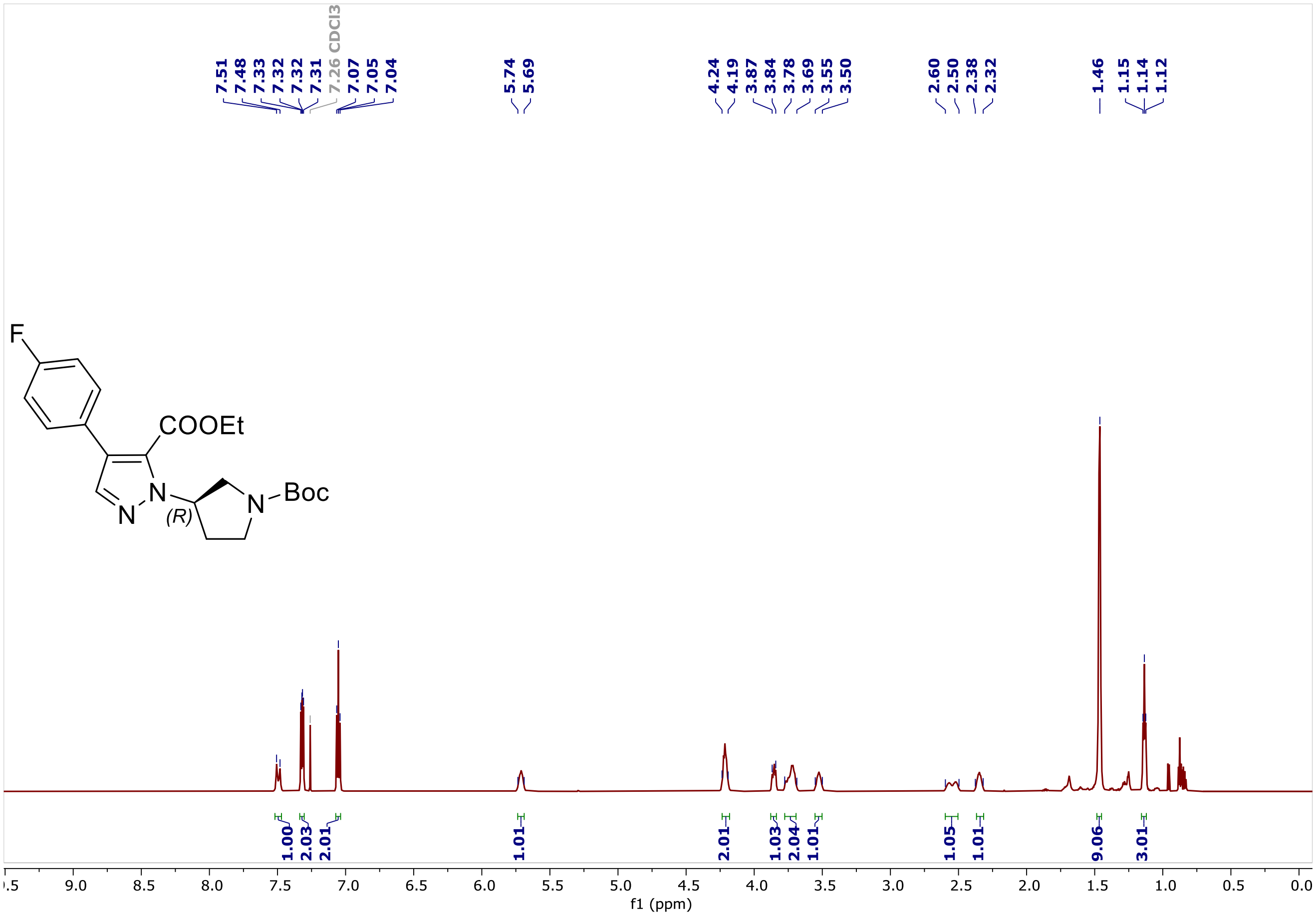

Figure S112. ^{1}H NMR (700 MHz, $CDCl_3$) spectrum of ***(R)*-17c**.

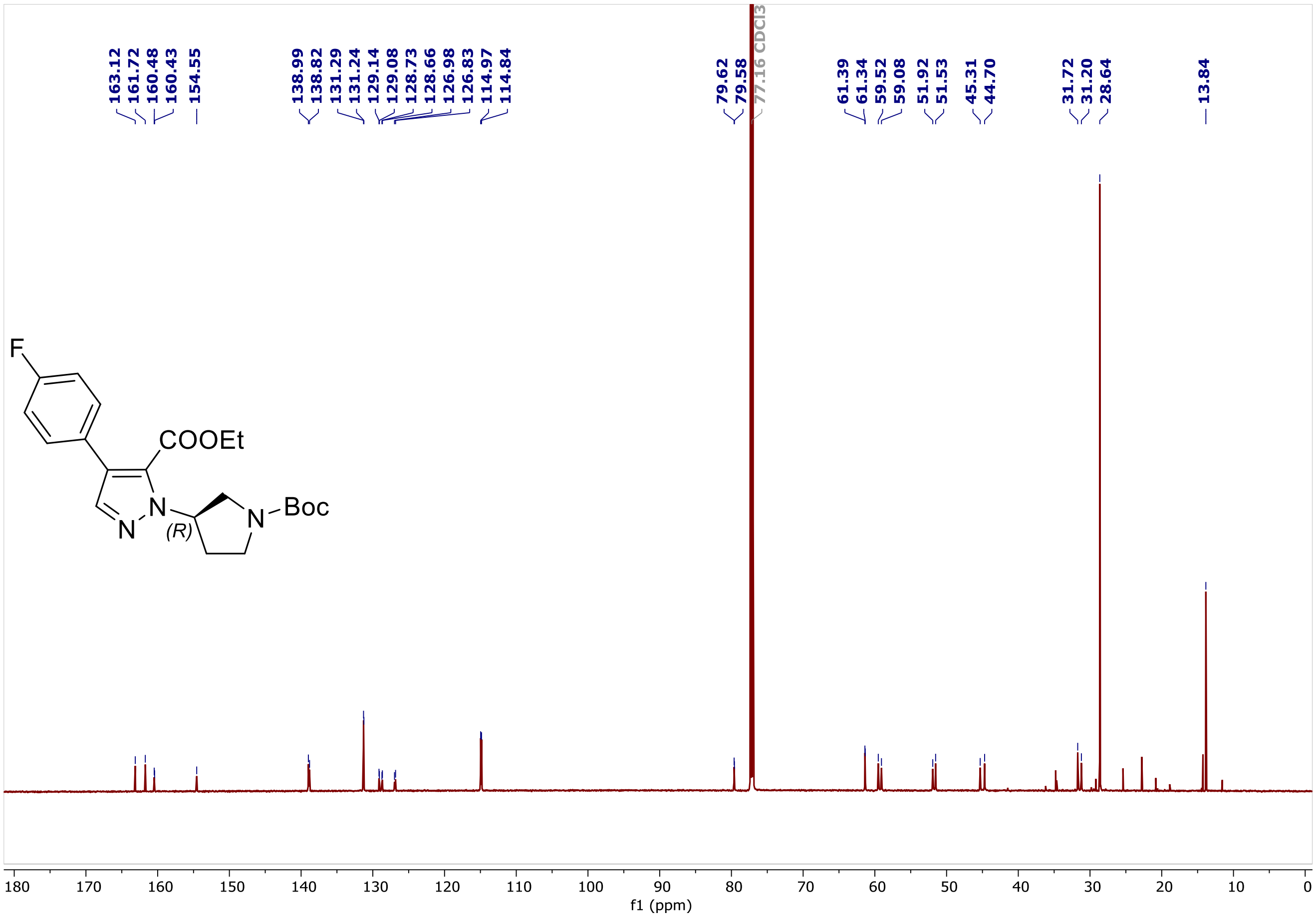

Figure S113. ^{13}C NMR (176 MHz, $CDCl_3$) spectrum of ***(R)*-17c**.

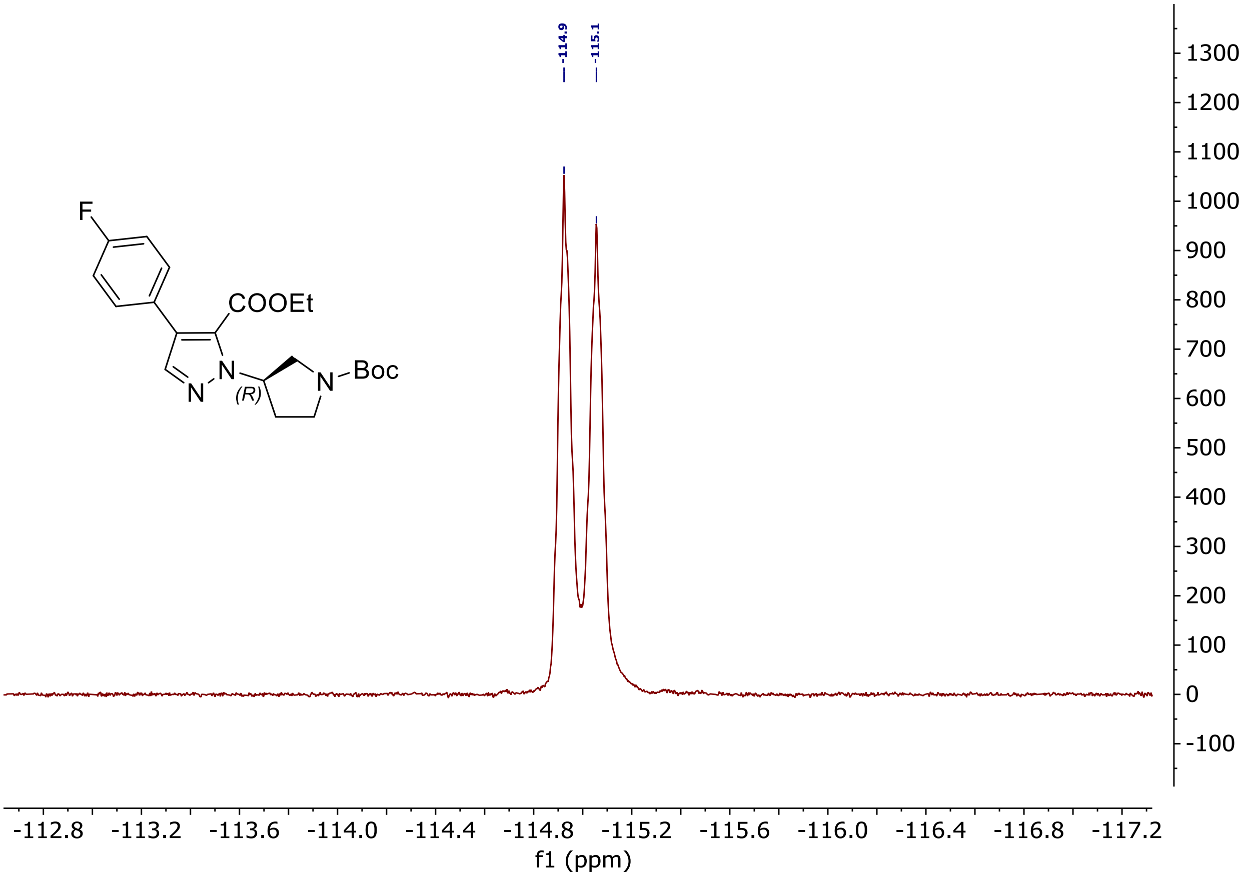

Figure S114. ^{19}F NMR (376 MHz, $CDCl_3$) spectrum of ***(R)*-17c**.

Acquisition Parameter

Source Type	ESI	Ion Polarity	Positive	Set Nebulizer	0.4 Bar
Focus	Not active	Set Capillary	4500 V	Set Dry Heater	180 °C
Scan Begin	50 m/z	Set End Plate Offset	-500 V	Set Dry Gas	4.0 l/min
Scan End	1000 m/z	Set Collision Cell RF	140.0 Vpp	Set Divert Valve	Waste

#	RT [min]	Area	Int. Type	I	S/N	Chromatogram	Max. m/z	FWHM [min]
n.a.	0.1	n.a.	Single spectrum	n.a.	n.a.	n.a.	304.2626	n.a.
n.a.	4.1	n.a.	Single spectrum	n.a.	n.a.	n.a.	426.1800	n.a.

+MS, 4.1min #246

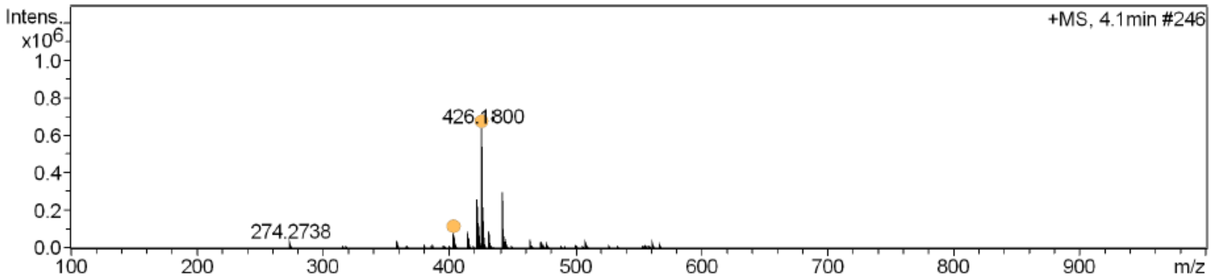

Meas. m/z	#	Ion Formula	m/z	err [ppm]	mSigma	# Sigma	Score	rdb	e⁻ Conf	N-Rule
404.1974	1	C21H27FN3O4	404.1980	1.5	3.0	1	100.00	9.5	even	ok
426.1800	1	C21H26FN3NaO4	426.1800	-0.1	14.3	1	100.00	9.5	even	ok

Figure S115. HRMS (ESI^+) report of ***(R)*-17c**.

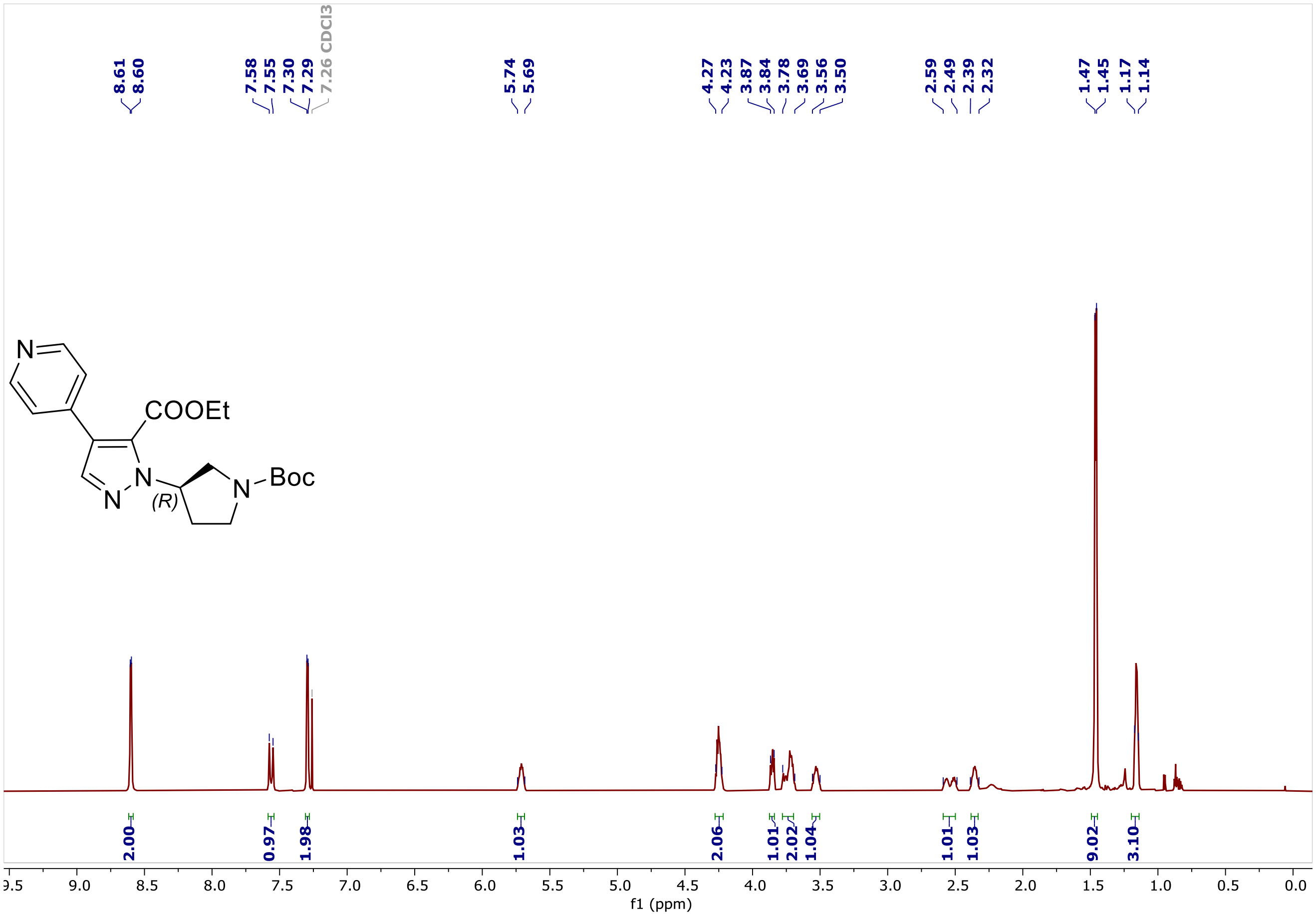

Figure S116. ^{1}H NMR (700 MHz, $CDCl_3$) spectrum of ***(R)*-17d**.

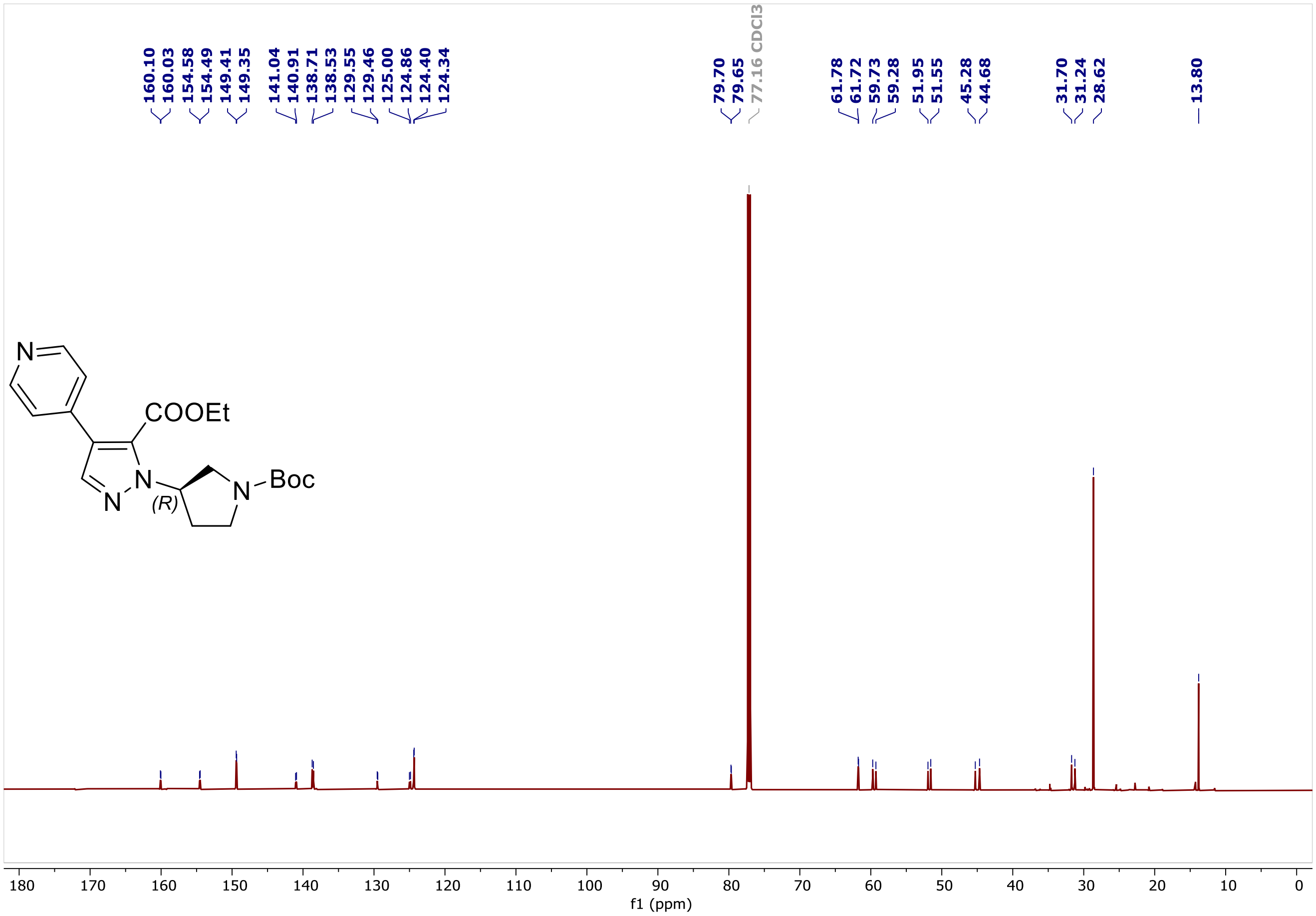

Figure S117. ^{13}C NMR (176 MHz, $CDCl_3$) spectrum of ***(R)*-17d**.

Acquisition Parameter

Source Type	ESI	Ion Polarity	Positive	Set Nebulizer	0.4 Bar
Focus	Not active	Set Capillary	4500 V	Set Dry Heater	180 °C
Scan Begin	50 m/z	Set End Plate Offset	-500 V	Set Dry Gas	4.0 l/min
Scan End	1000 m/z	Set Collision Cell RF	140.0 Vpp	Set Divert Valve	Waste

#	RT [min]	Area	Int. Type	I	S/N	Chromatogram	Max. m/z	FWHM [min]
n.a.	0.1	n.a.	Single spectrum	n.a.	n.a.	n.a.	304.2622	n.a.
n.a.	3.9	n.a.	Single spectrum	n.a.	n.a.	n.a.	387.2027	n.a.

+MS, 3.9min #231

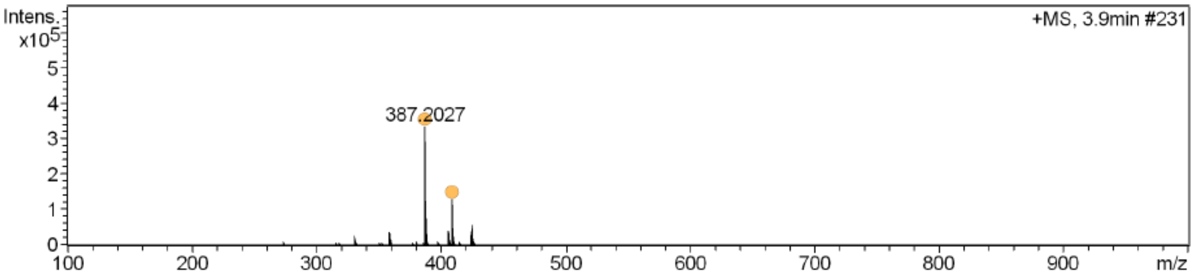

Meas. m/z	#	Ion Formula	m/z	err [ppm]	mSigma	# Sigma	Score	rdb	e⁻ Conf	N-Rule
387.2027	1	C20H27N4O4	387.2027	0.1	13.1	1	100.00	9.5	even	ok
409.1853	1	C20H26N4NaO4	409.1846	1.7	9.1	1	100.00	9.5	even	ok

Figure S118. HRMS (ESI^+) report of ***(R)*-17d**.

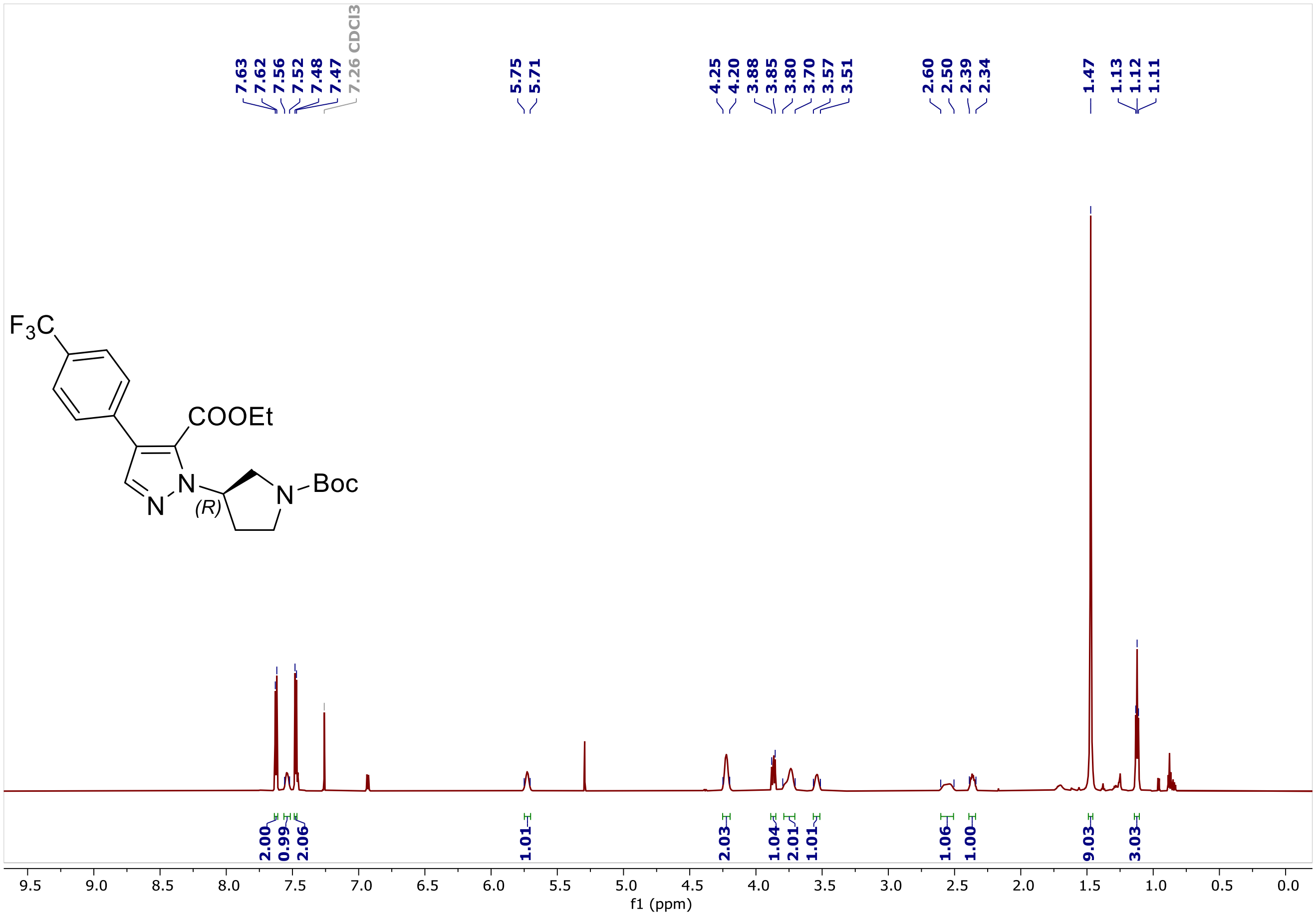

Figure S119. ^{1}H NMR (700 MHz, $CDCl_3$) spectrum of ***(R)*-17e**.

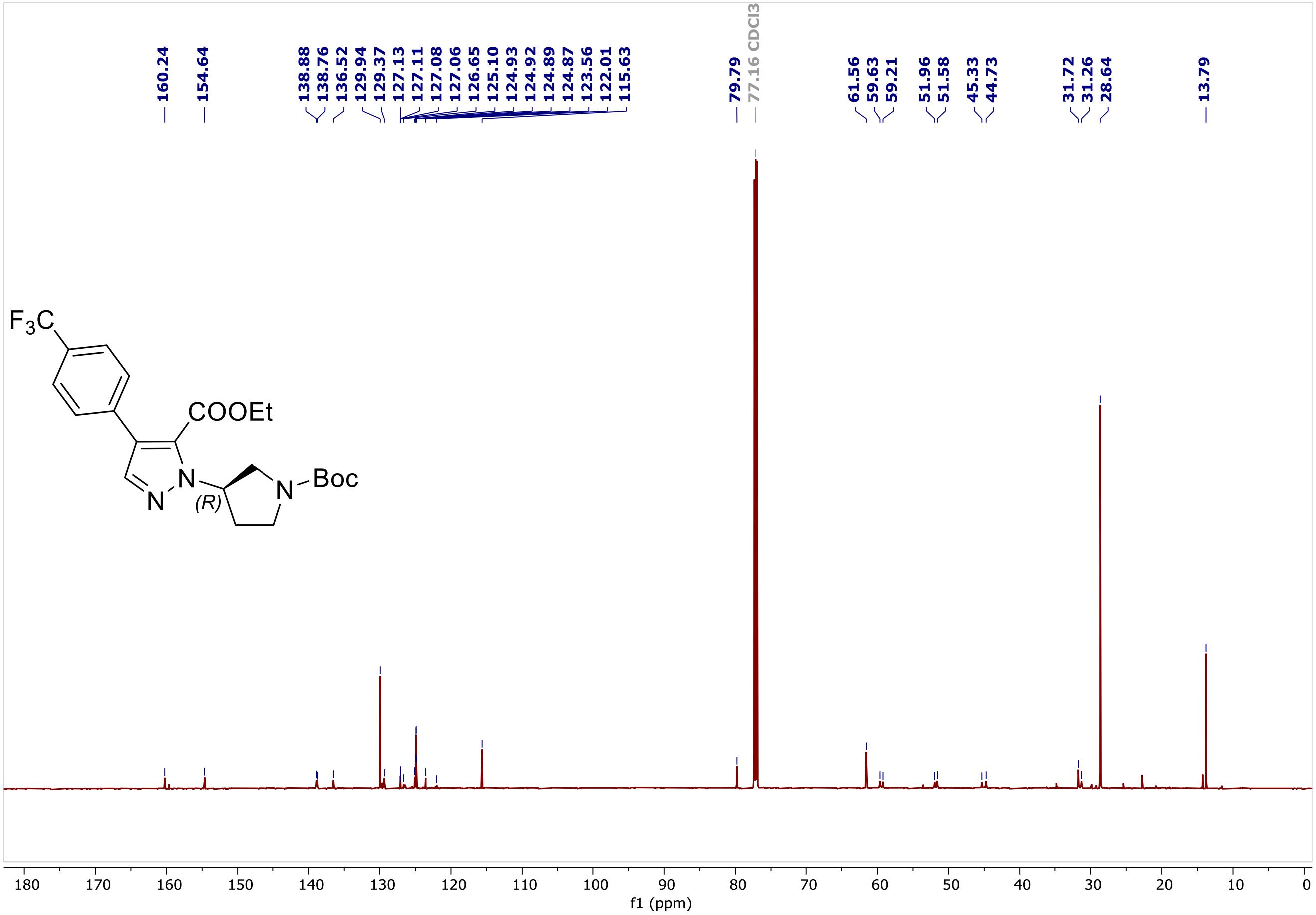

Figure S120. ^{13}C NMR (176 MHz, $CDCl_3$) spectrum of ***(R)*-17e**.

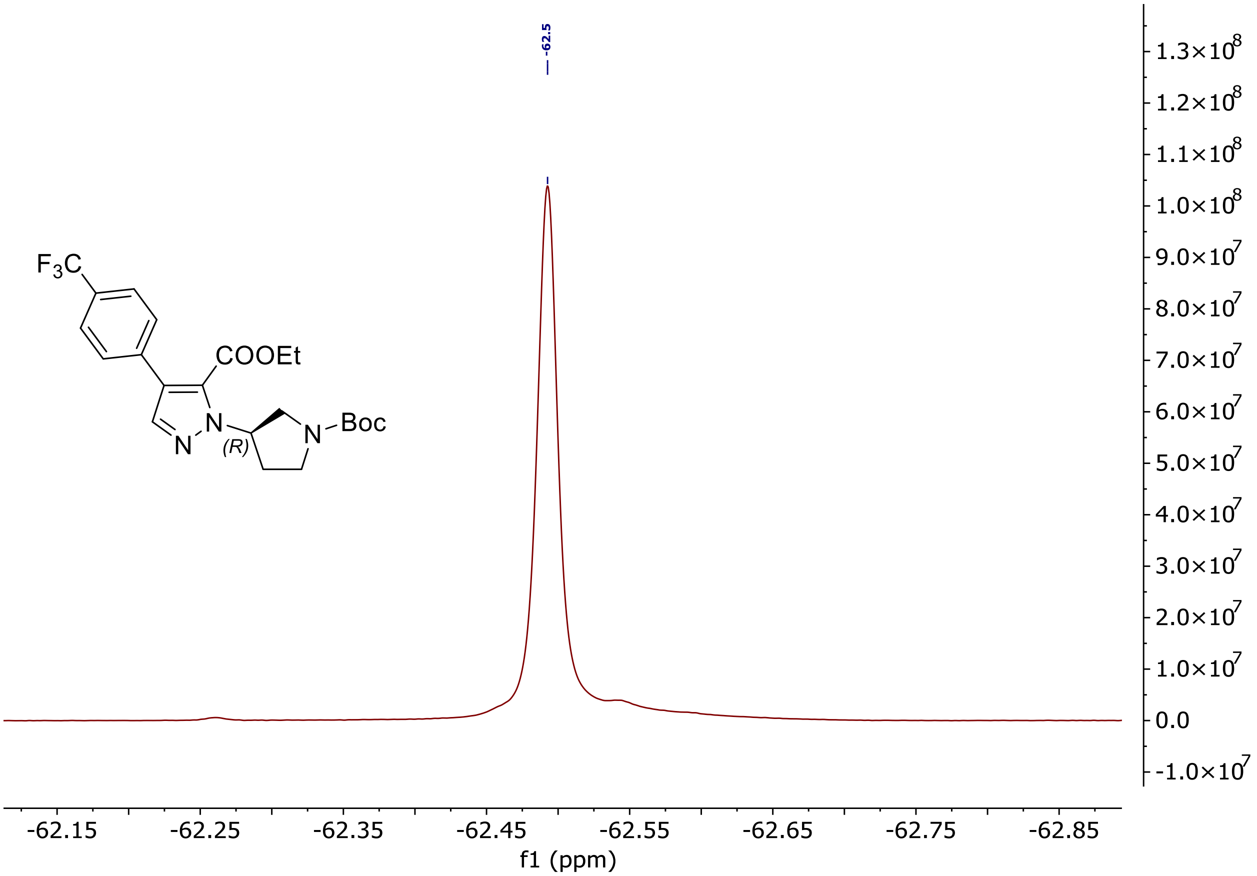

Figure S121. ^{19}F NMR (376 MHz, $CDCl_3$) spectrum of ***(R)*-17e**.

Acquisition Parameter

Source Type	ESI	Ion Polarity	Positive	Set Nebulizer	0.4 Bar
Focus	Not active	Set Capillary	4500 V	Set Dry Heater	180 °C
Scan Begin	50 m/z	Set End Plate Offset	-500 V	Set Dry Gas	4.0 l/min
Scan End	1000 m/z	Set Collision Cell RF	140.0 Vpp	Set Divert Valve	Waste

#	RT [min]	Area	Int. Type	I	S/N	Chromatogram	Max. m/z	FWHM [min]
n.a.	0.4	n.a.	Single spectrum	n.a.	n.a.	n.a.	304.2626	n.a.
n.a.	3.9	n.a.	Single spectrum	n.a.	n.a.	n.a.	476.1766	n.a.

+MS, 3.9min #236

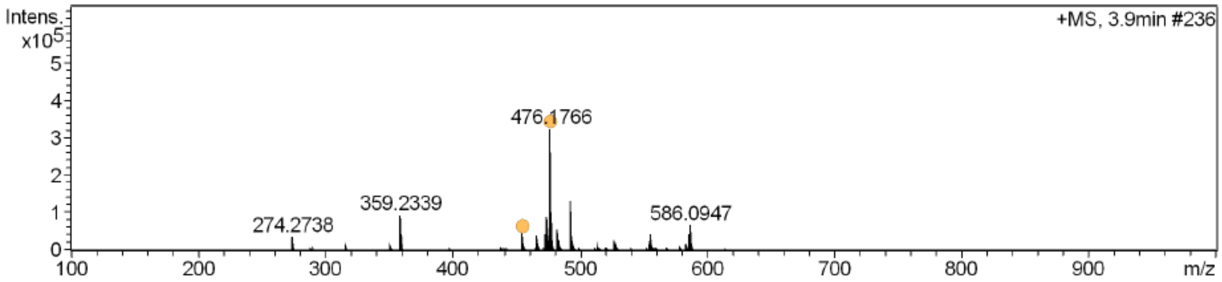

Meas. m/z	#	Ion Formula	m/z	err [ppm]	mSigma	# Sigma	Score	rdb	e⁻ Conf	N-Rule
454.1942	1	C22H27F3N3O4	454.1948	1.3	44.6	1	100.00	9.5	even	ok
476.1766	1	C22H26F3N3NaO4	476.1768	0.3	11.5	1	100.00	9.5	even	ok

Figure S122. HRMS (ESI^+) report of ***(R)*-17e**.

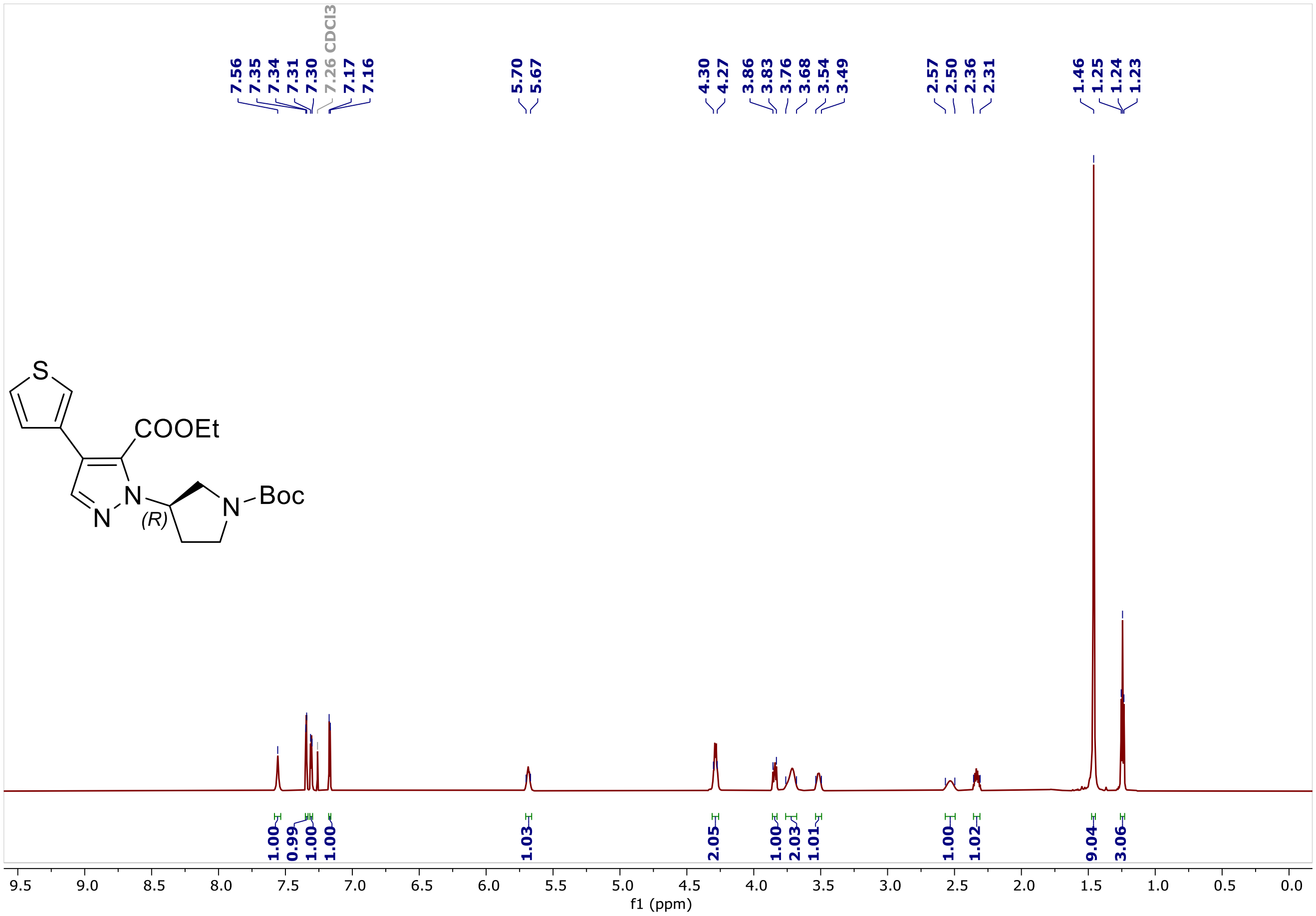

Figure S123. ^{1}H NMR (700 MHz, $CDCl_3$) spectrum of ***(R)*-17f**.

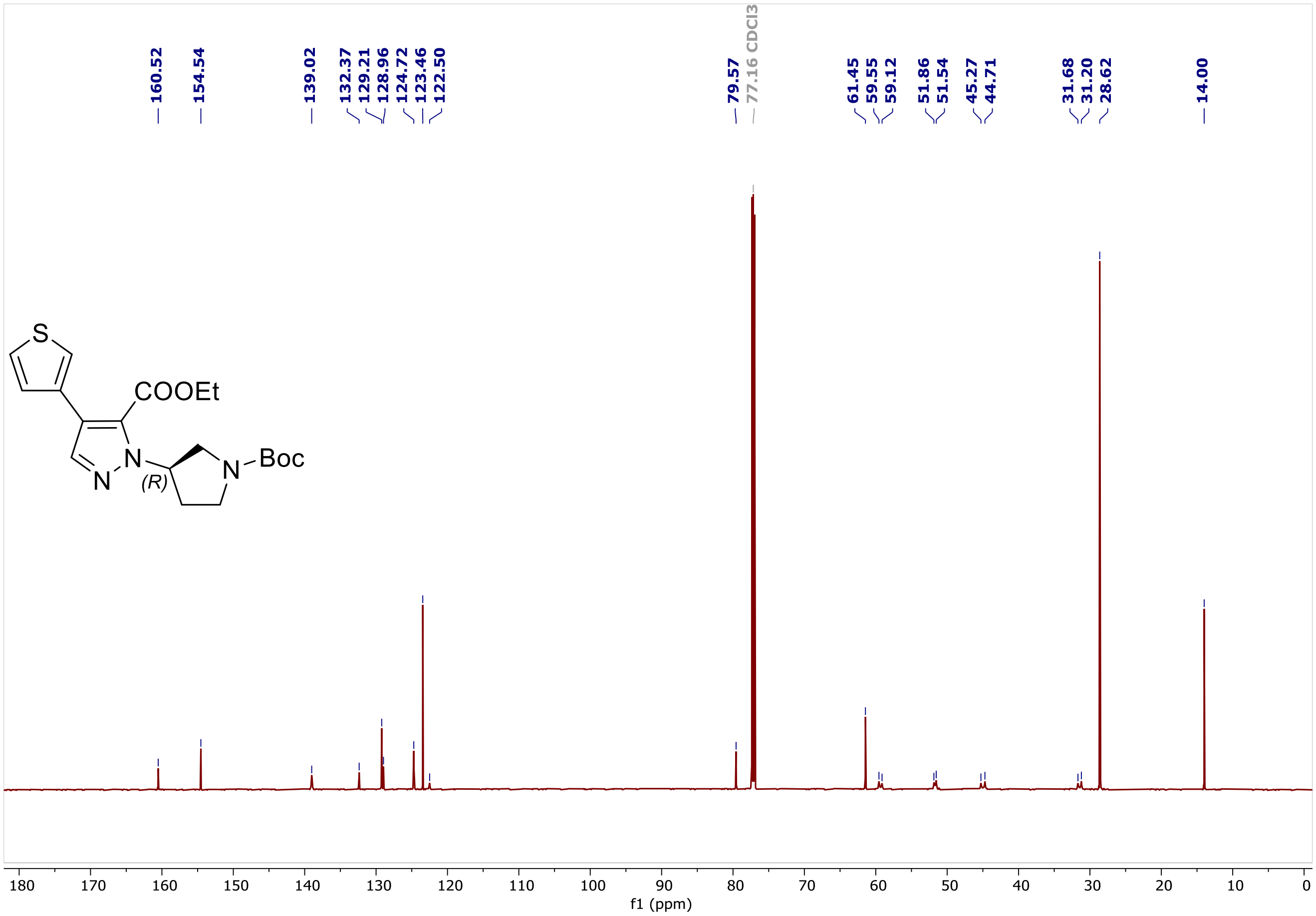

Figure S124. ^{13}C NMR (176 MHz, $CDCl_3$) spectrum of **(*R*)-17f**.

Acquisition Parameter

Source Type	ESI	Ion Polarity	Positive	Set Nebulizer	0.4 Bar
Focus	Not active	Set Capillary	4500 V	Set Dry Heater	180 °C
Scan Begin	50 m/z	Set End Plate Offset	-500 V	Set Dry Gas	4.0 l/min
Scan End	1000 m/z	Set Collision Cell RF	140.0 Vpp	Set Divert Valve	Waste

#	RT [min]	Area	Int. Type	I	S/N	Chromatogram	Max. m/z	FWHM [min]
n.a.	0.2	n.a.	Single spectrum	n.a.	n.a.	n.a.	304.2624	n.a.
n.a.	3.6	n.a.	Single spectrum	n.a.	n.a.	n.a.	414.1458	n.a.

+MS, 3.6min #217

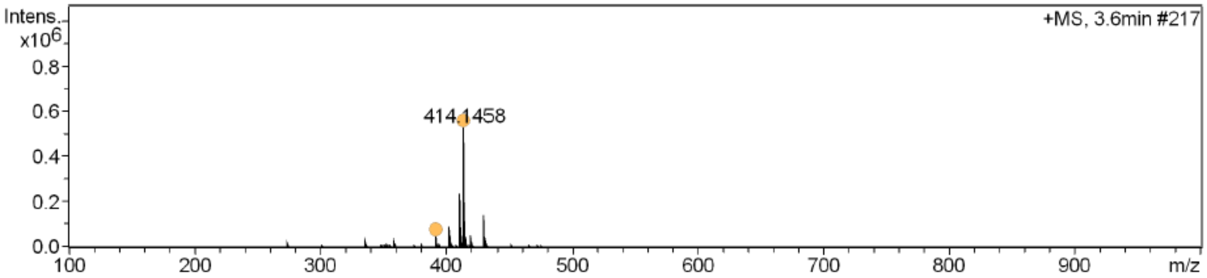

Meas. m/z	#	Ion Formula	m/z	err [ppm]	mSigma	# Sigma	Score	rdb	e⁻ Conf	N-Rule
392.1633	1	C19H26N3O4S	392.1639	1.4	11.7	1	100.00	8.5	even	ok
414.1458	1	C19H25N3NaO4S	414.1458	-0.1	10.0	1	100.00	8.5	even	ok

Figure S125. HRMS (ESI$^+$) report of **(*R*)-17f**.

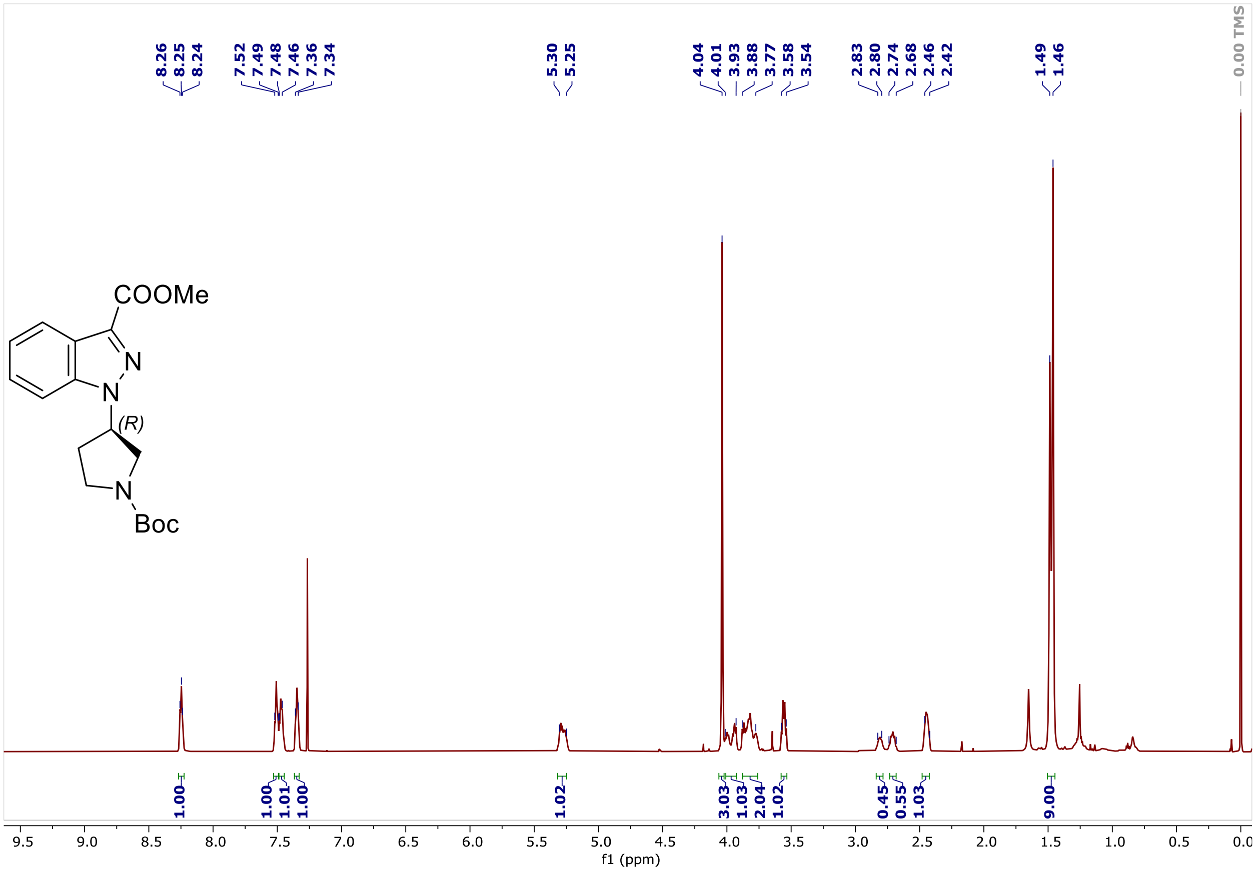

Figure S126. ^{1}H NMR (700 MHz, $CDCl_3$) spectrum of ***(R)*-19a**.

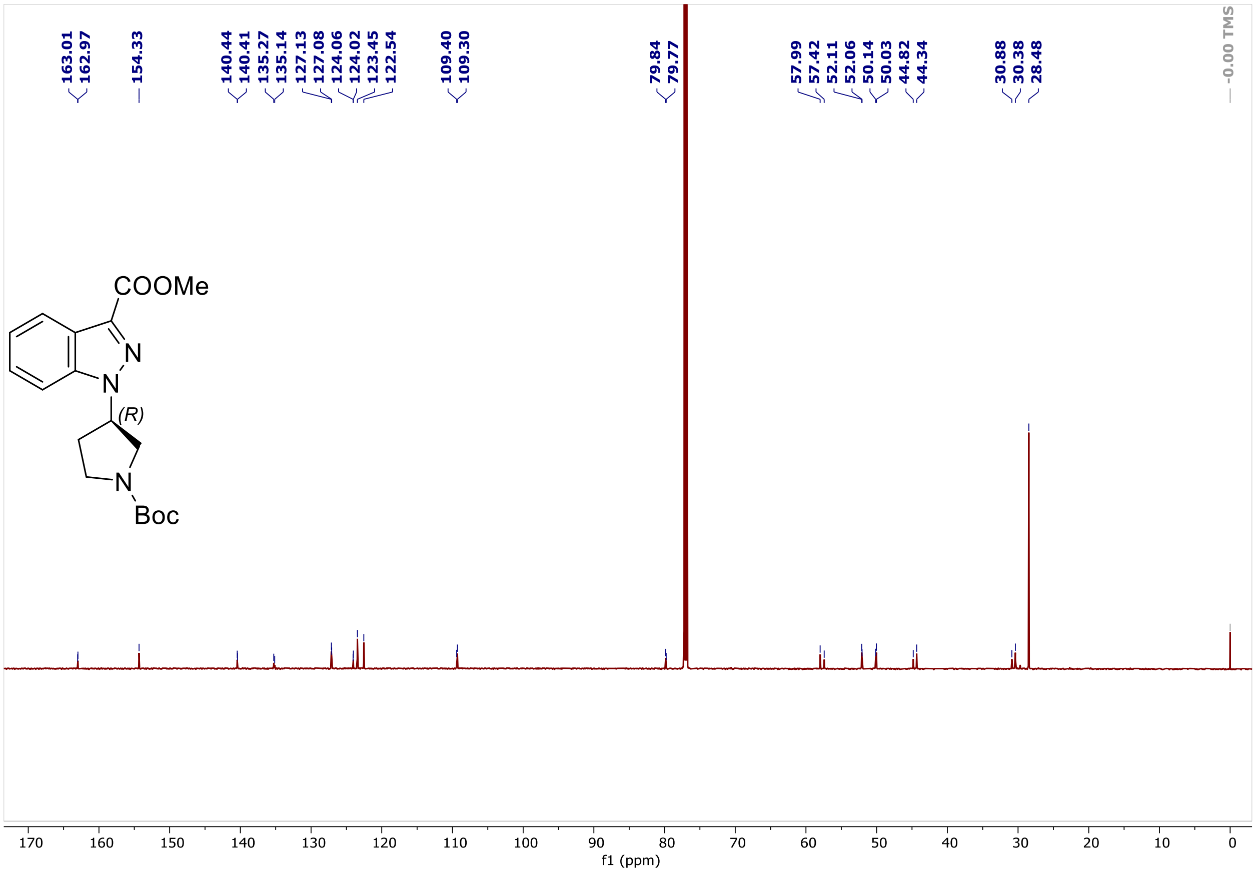

Figure S127. ^{13}C NMR (176 MHz, $CDCl_3$) spectrum of ***(R)*-19a**.

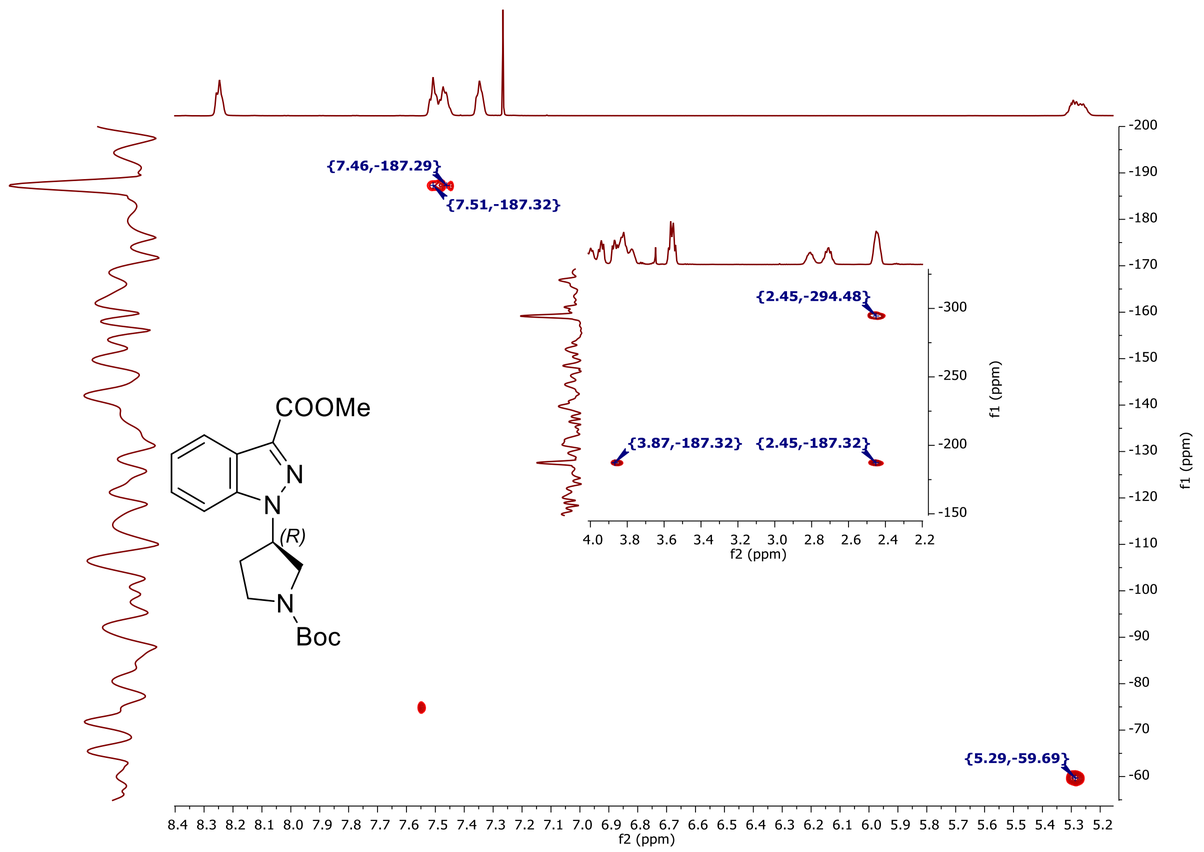

Figure S128. ^{1}H-^{15}N HMBC NMR spectrum of **(*R*)-19a** (71 MHz, $CDCl_3$).

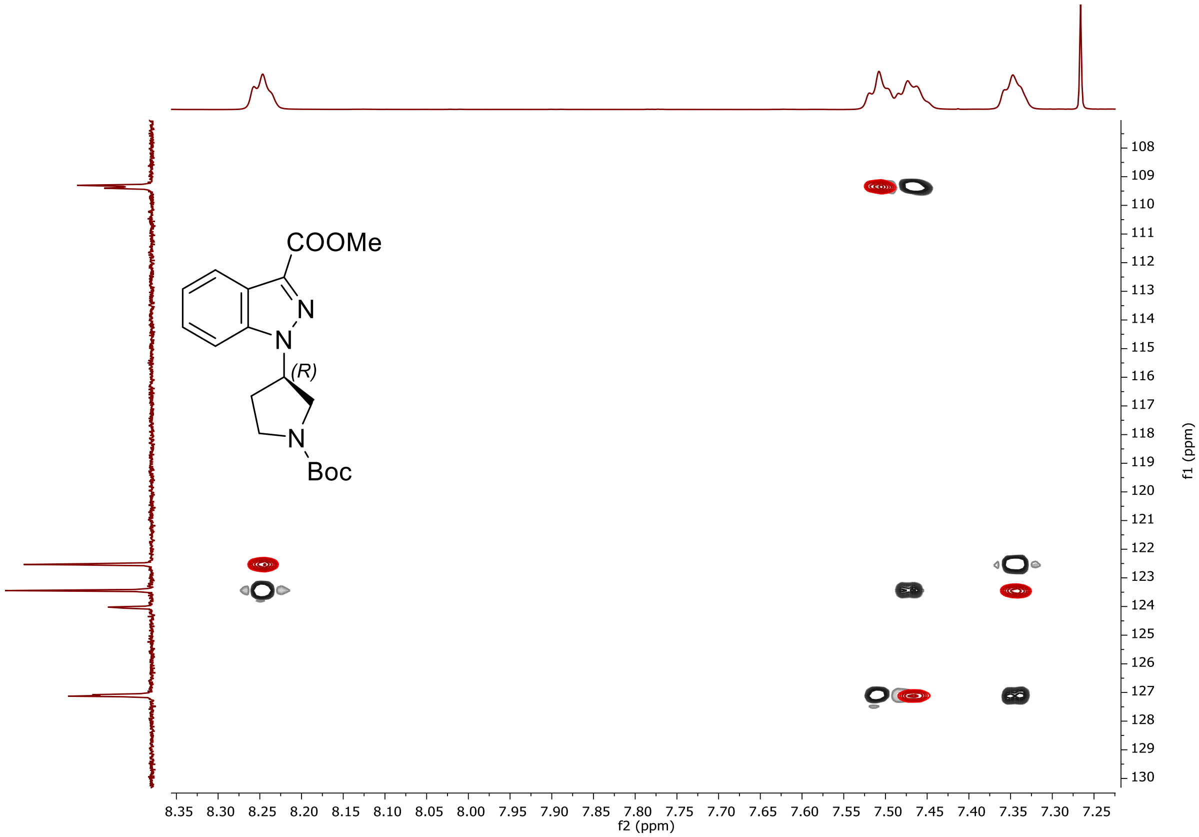

Figure S129. Overlaid multiplicity-edited ^{1}H-^{13}C HSQC (red correlations) and ^{1}H-^{13}C H2BC (black correlations) NMR spectra of ***(R)*-19a** ($CDCl_3$).

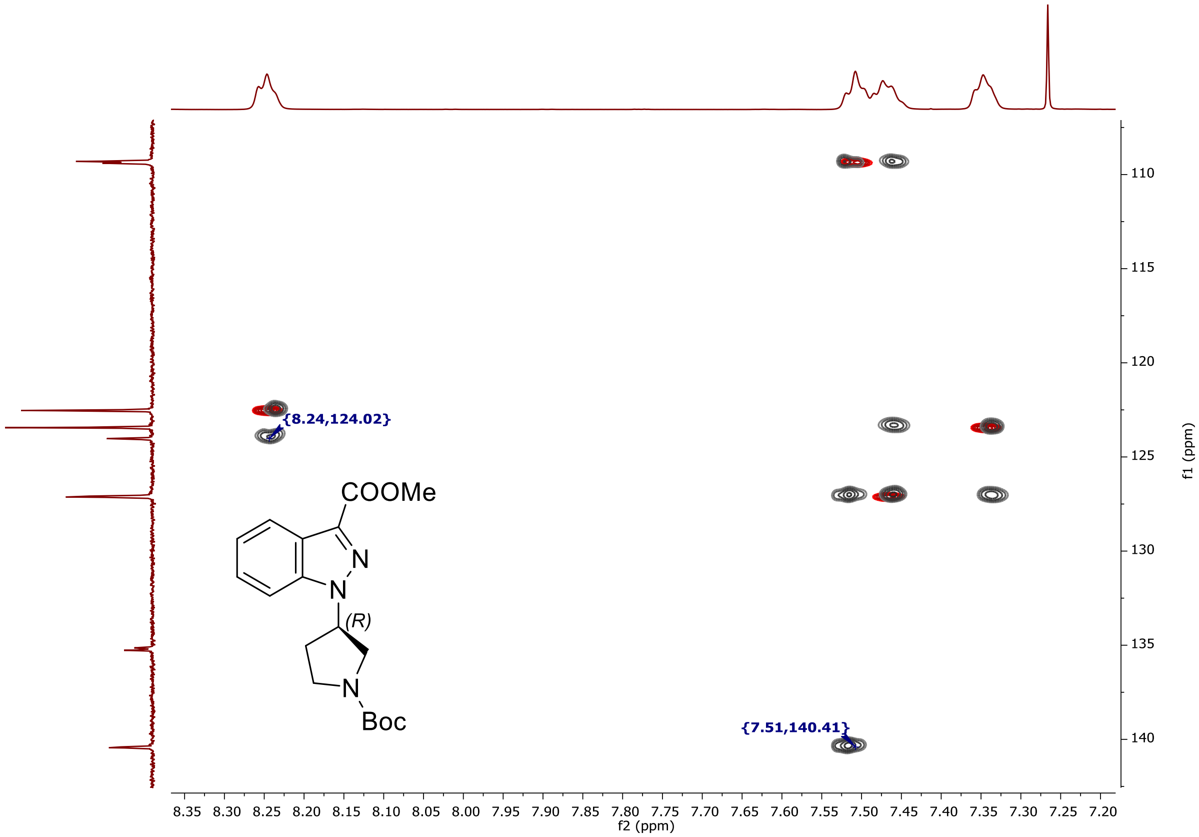

Figure S130. Overlaid multiplicity-edited ^{1}H-^{13}C HSQC (red correlations) and 1,1-ADEQUATE 60Hz (black correlations) NMR spectra of ***(R)*-19a** ($CDCl_3$).

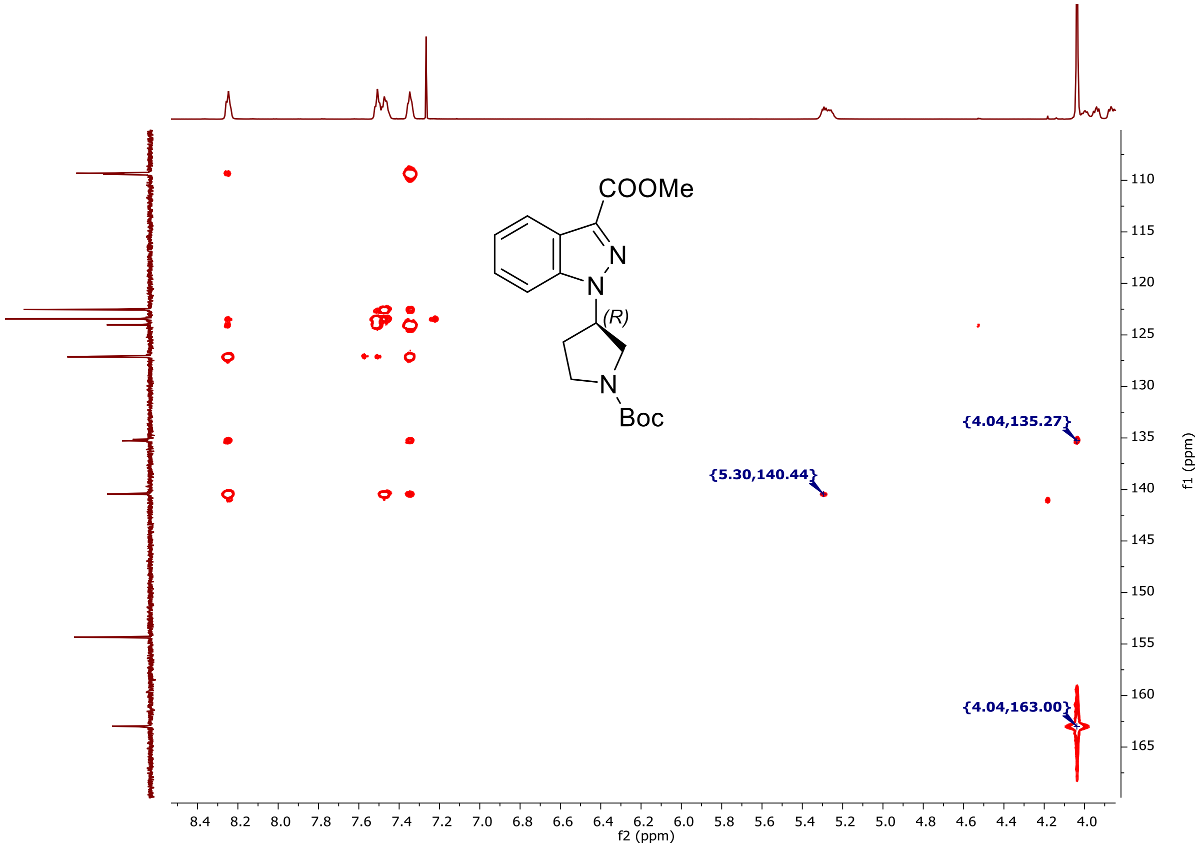

Figure S131. The 8Hz optimized ^{1}H-^{13}C HMBC NMR spectrum of ***(R)*-19a** ($CDCl_3$).

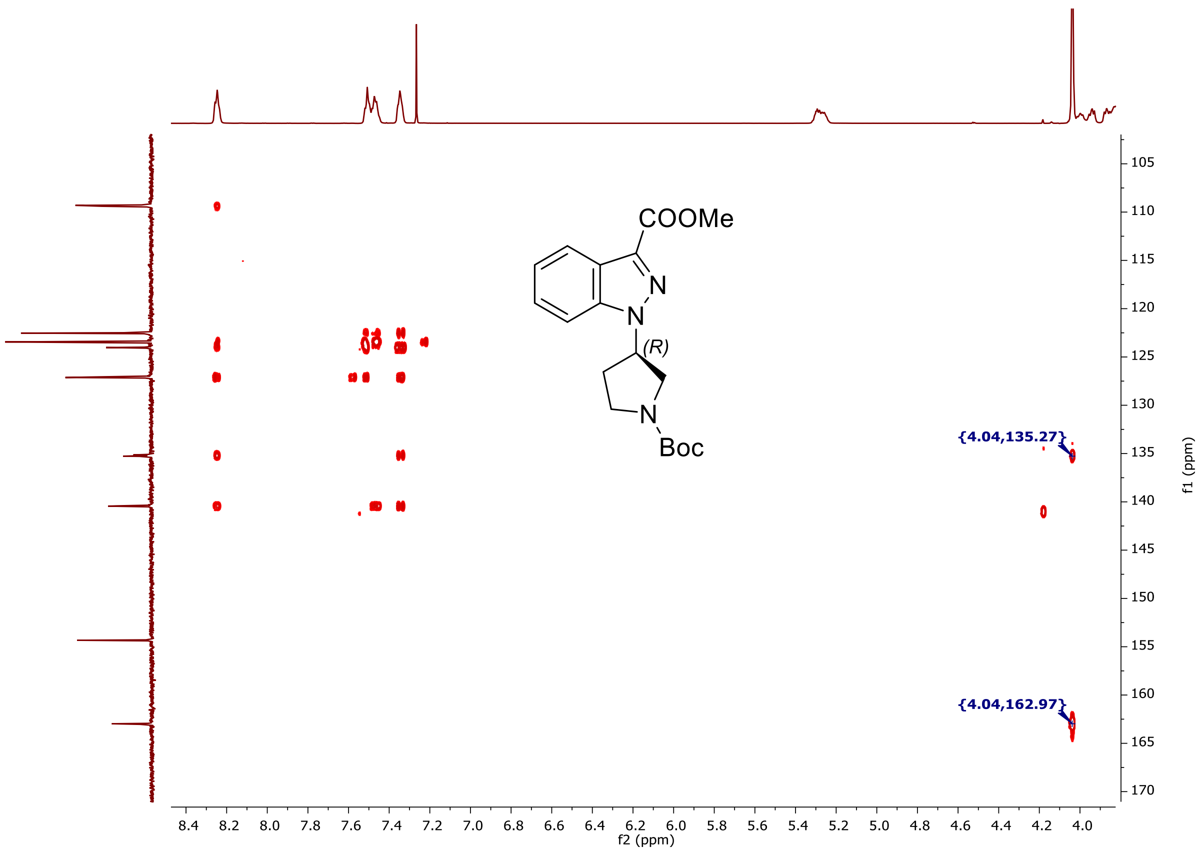

Figure S132. The 2Hz optimized ^{1}H-^{13}C HMBC NMR spectrum of ***(R)*-19a** ($CDCl_3$).

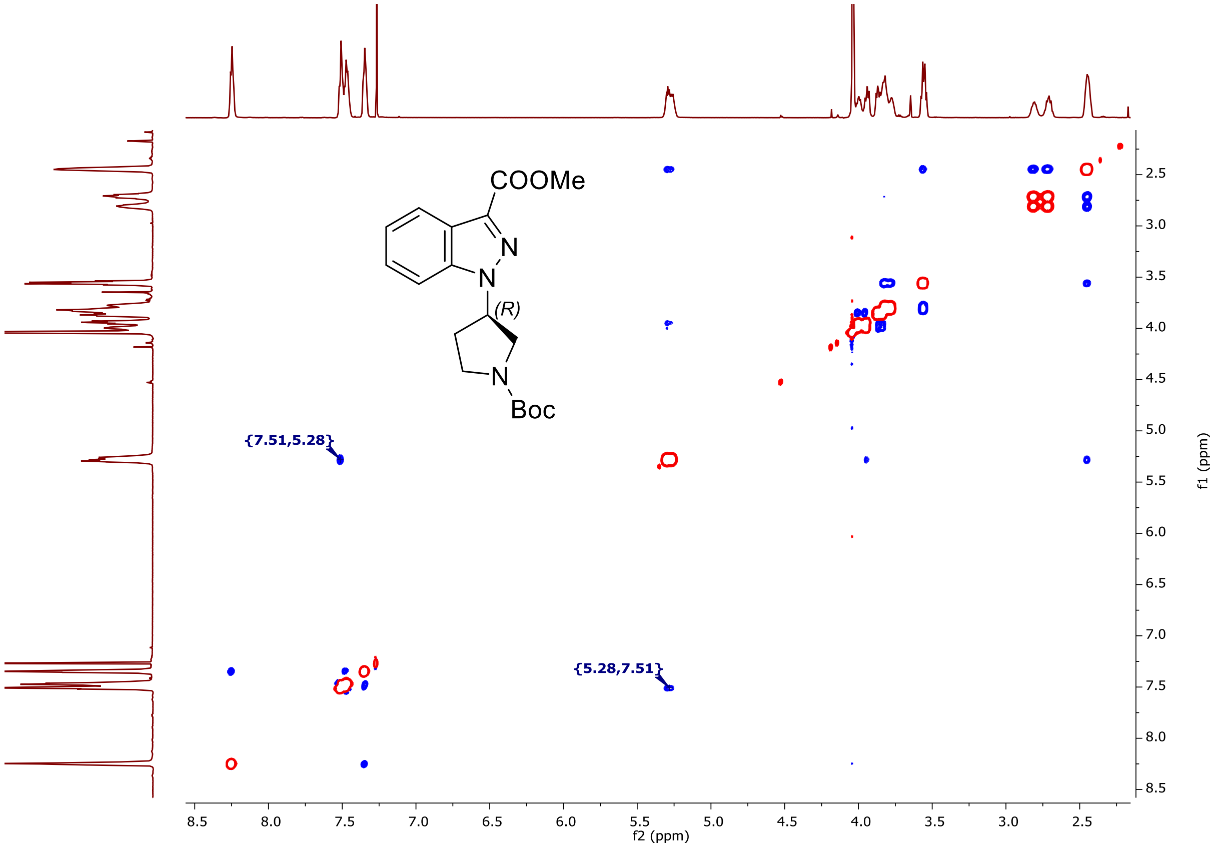

Figure S133. ^{1}H-^{1}H NOESY NMR spectrum of ***(R)*-19a** ($CDCl_3$).

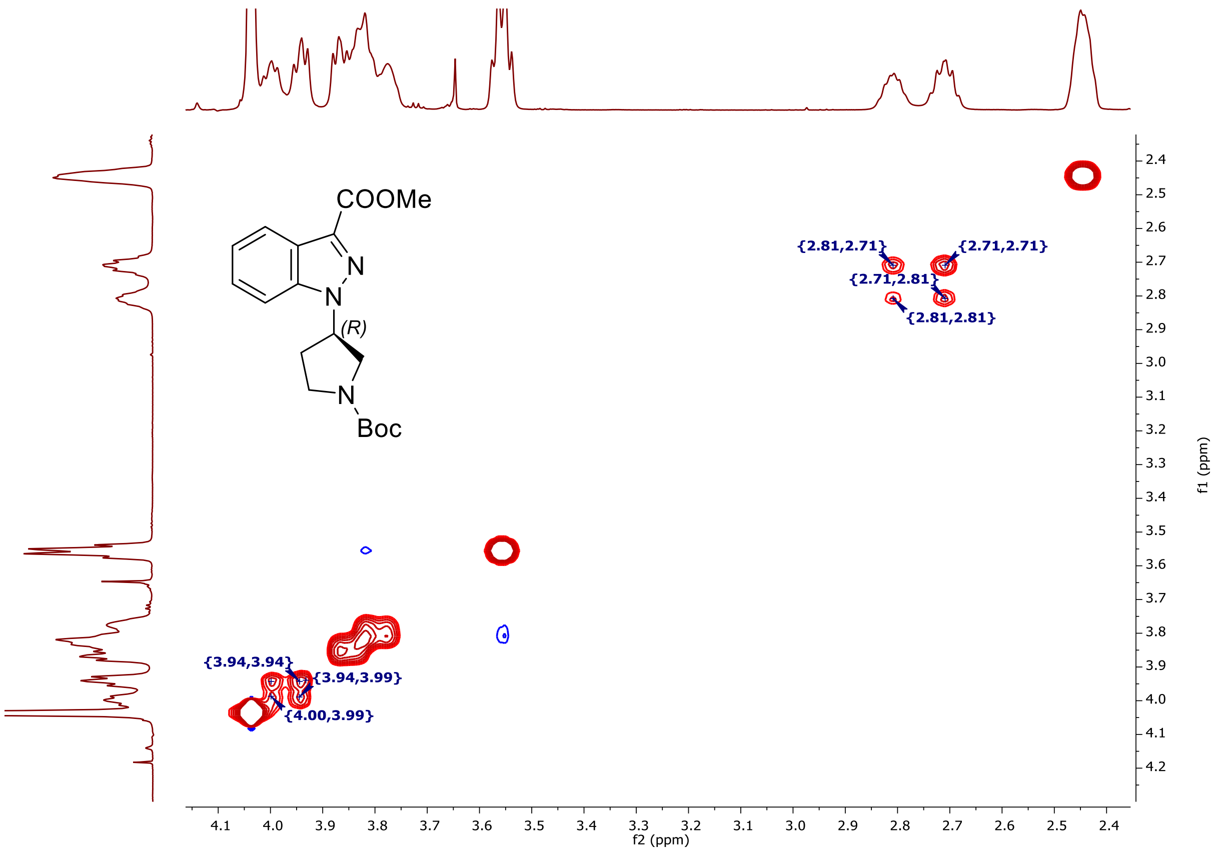

Figure S134. ^{1}H-^{1}H NOESY/EXSY NMR spectrum of ***(R)*-19a** ($CDCl_3$).

Acquisition Parameter

Source Type	ESI	Ion Polarity	Positive	Set Nebulizer	0.4 Bar
Focus	Not active	Set Capillary	4500 V	Set Dry Heater	180 °C
Scan Begin	50 m/z	Set End Plate Offset	-500 V	Set Dry Gas	4.0 l/min
Scan End	1000 m/z	Set Collision Cell RF	140.0 Vpp	Set Divert Valve	Waste

#	RT [min]	Area	Int. Type	I	S/N	Chromatogram	Max. m/z	FWHM [min]
n.a.	0.2	n.a.	Single spectrum	n.a.	n.a.	n.a.	304.2623	n.a.
n.a.	6.0	n.a.	Single spectrum	n.a.	n.a.	n.a.	368.1581	n.a.

+MS, 6.0min #361

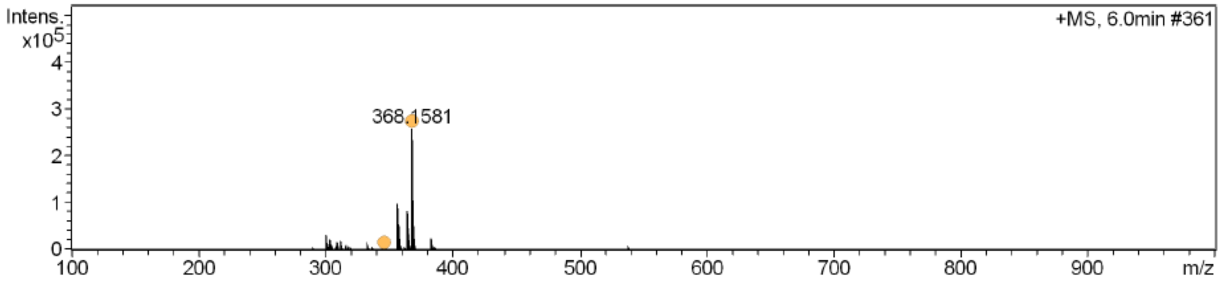

Meas. m/z	#	Ion Formula	m/z	err [ppm]	mSigma	# Sigma	Score	rdb	e⁻ Conf	N-Rule
346.1777	1	C18H24N3O4	346.1761	-4.5	205.0	1	100.00	8.5	even	ok
368.1581	1	C18H23N3NaO4	368.1581	0.0	3.6	1	100.00	8.5	even	ok

Figure S135. HRMS (ESI$^+$) report of ***(R)*-19a**.

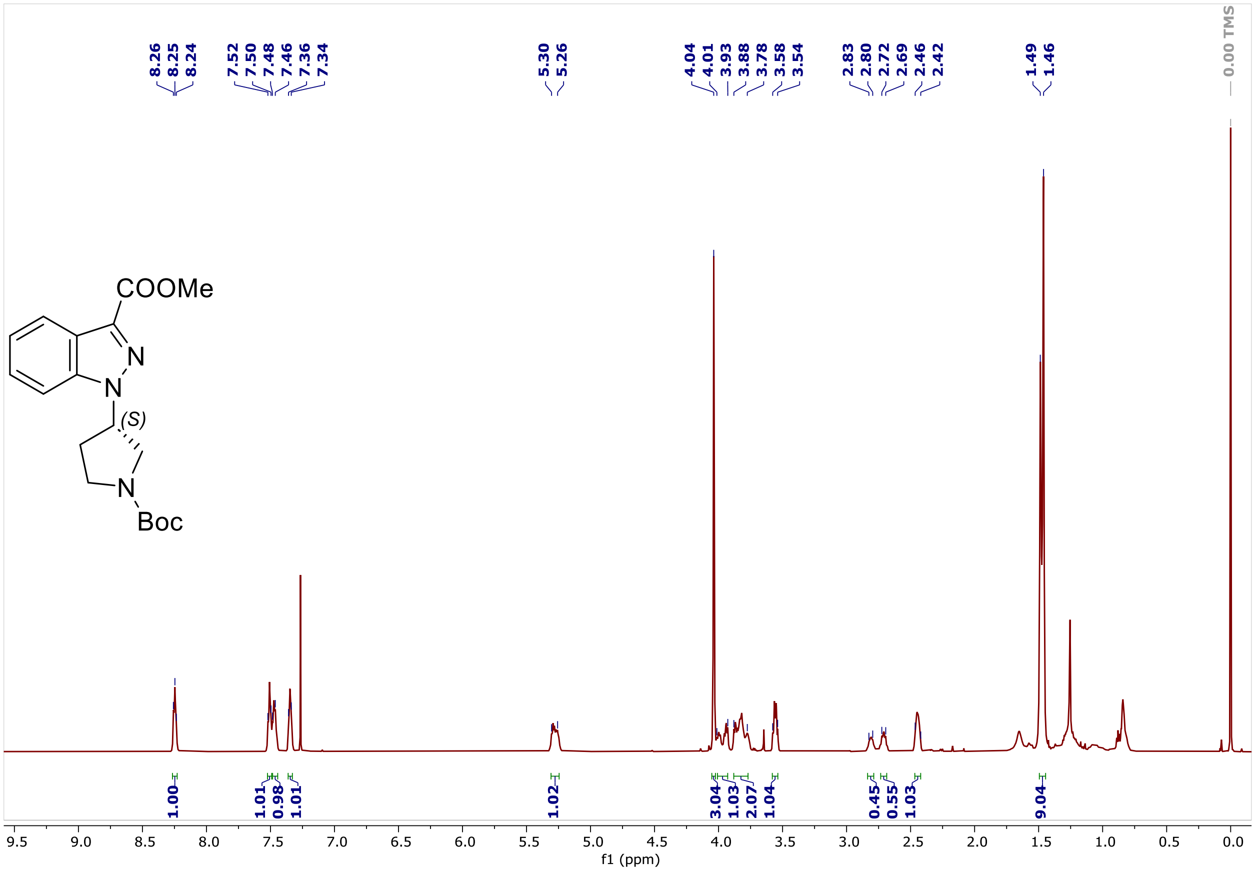

Figure S136. ^{1}H NMR (700 MHz, $CDCl_3$) spectrum of **(*S*)-19a**.

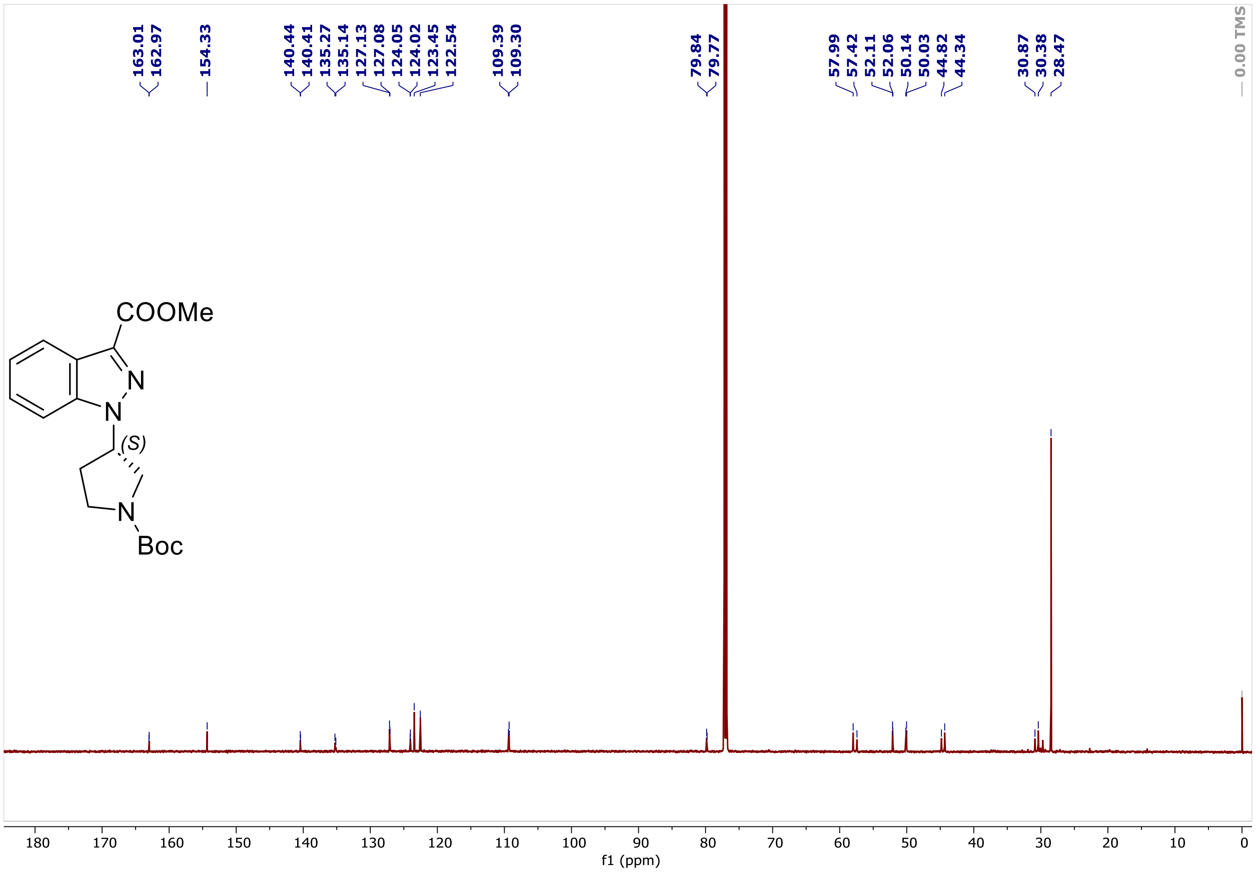

Figure S137. ^{13}C NMR (176 MHz, $CDCl_3$) spectrum of ***(S)*-19a**.

Acquisition Parameter

Source Type	ESI	Ion Polarity	Positive	Set Nebulizer	0.4 Bar		
Focus	Not active	Set Capillary	4500 V	Set Dry Heater	180 °C		
Scan Begin	50 m/z	Set End Plate Offset	-500 V	Set Dry Gas	4.0 l/min		
Scan End	1000 m/z	Set Collision Cell RF	140.0 Vpp	Set Divert Valve	Waste		

#	RT [min]	Area	Int. Type	I	S/N	Chromatogram	Max. m/z	FWHM [min]
n.a.	0.1	n.a.	Single spectrum	n.a.	n.a.	n.a.	304.2636	n.a.
n.a.	4.3	n.a.	Single spectrum	n.a.	n.a.	n.a.	368.1580	n.a.

+MS, 4.3min #260

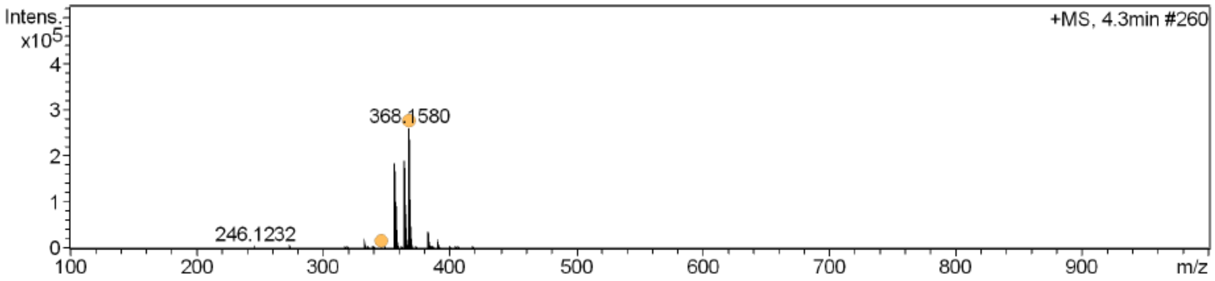

Meas. m/z	#	Ion Formula	m/z	err [ppm]	mSigma	# Sigma	Score	rdb	e⁻ Conf	N-Rule
346.1737	1	C18H24N3O4	346.1761	7.1	396.5	1	100.00	8.5	even	ok
368.1580	1	C18H23N3NaO4	368.1581	0.3	3.7	1	100.00	8.5	even	ok

Figure S138. HRMS (ESI^+) report of ***(S)*-19a**.

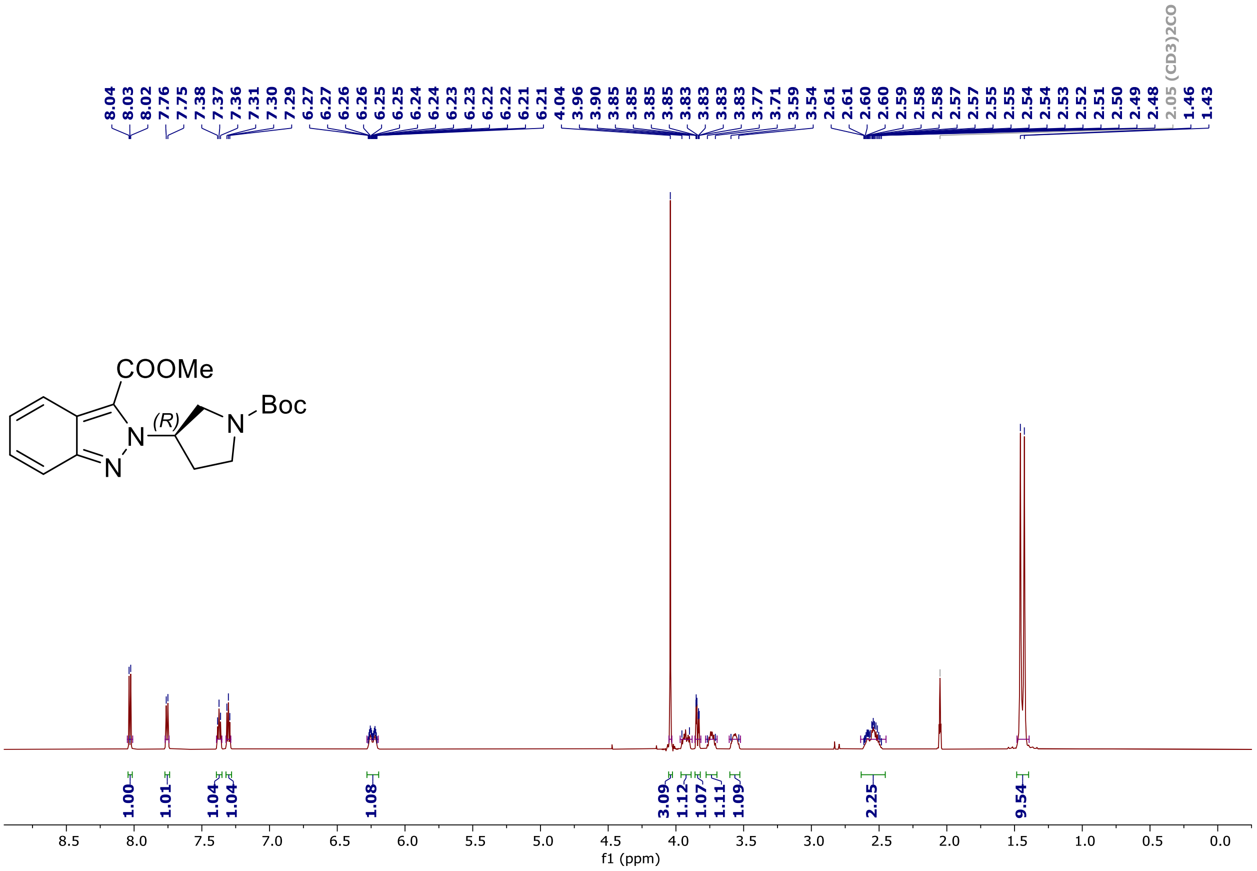

Figure S139. ^{1}H NMR (700 MHz, Acetone-d_6) spectrum of ***(R)*-20a**.

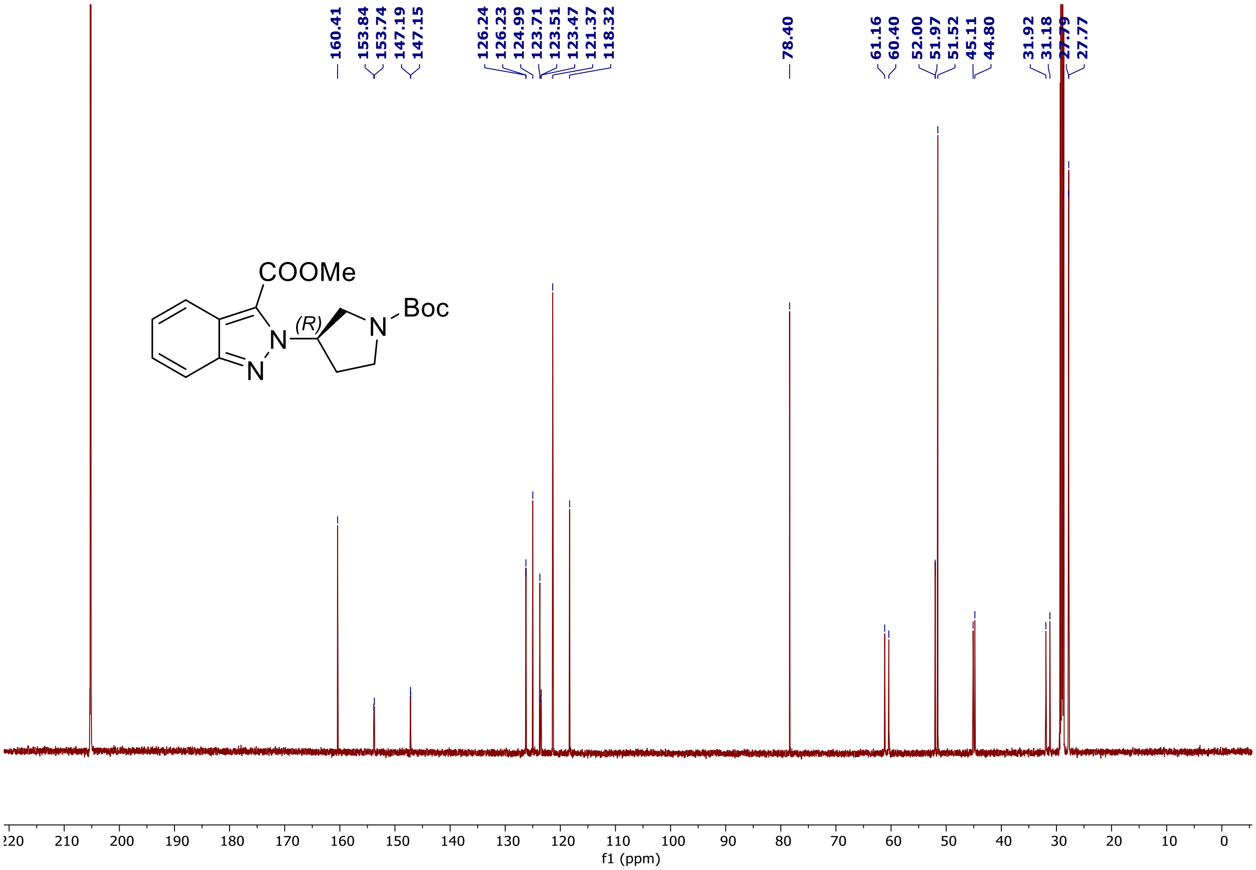

Figure S140. ^{13}C NMR (176 MHz, Acetone-d_6) spectrum of ***(R)*-20a**.

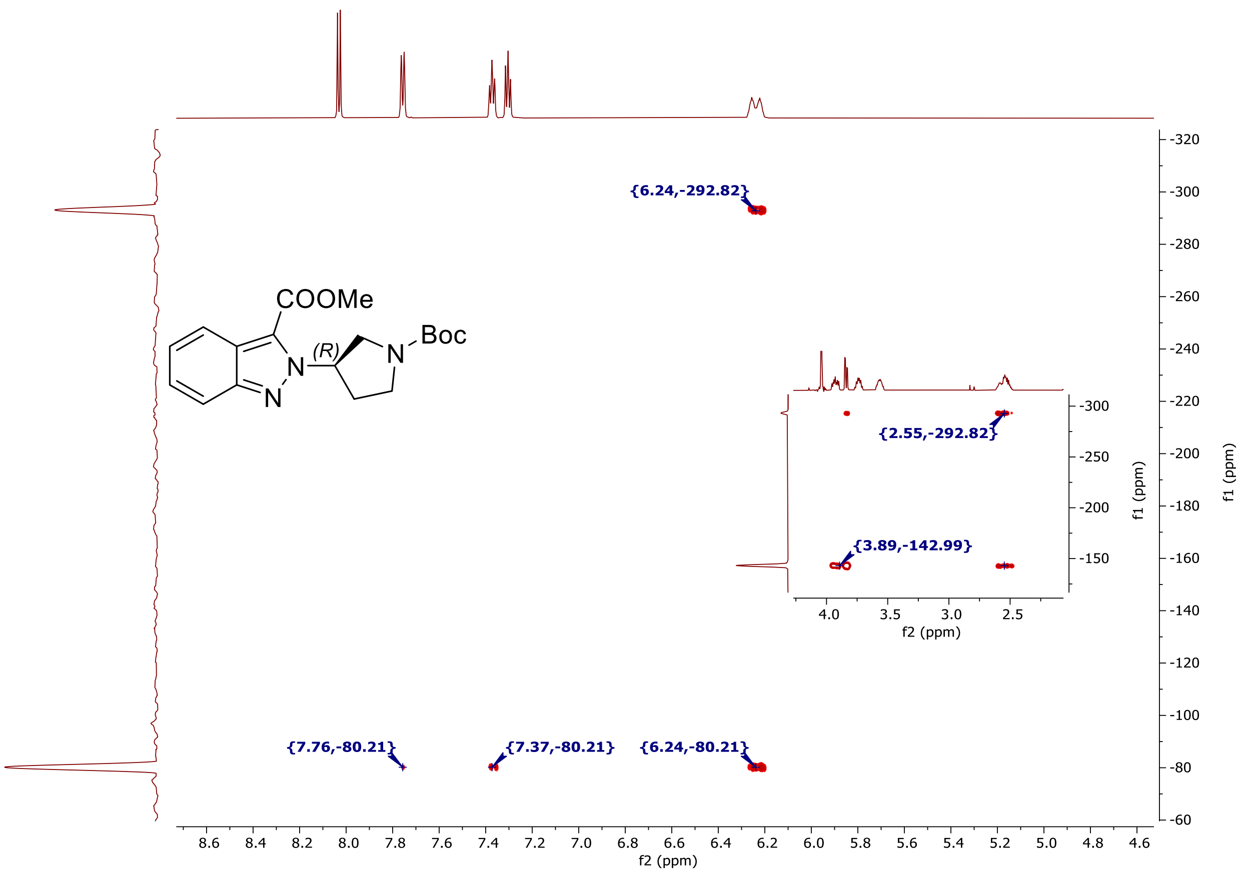

Figure S141. ^{1}H-^{15}N HMBC NMR spectrum of ***(R)*-20a** (71 MHz, Acetone-d_6).

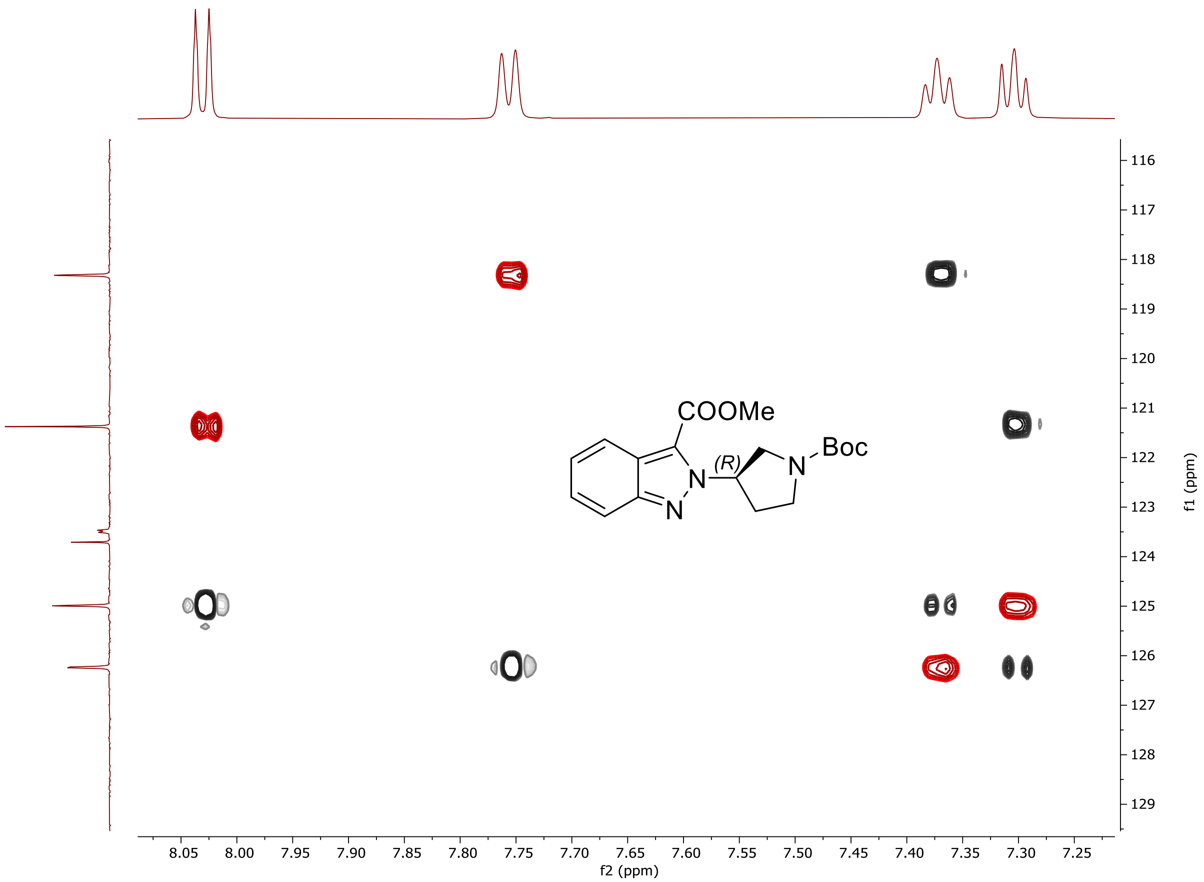

Figure S142. Overlaid multiplicity-edited ^{1}H-^{13}C HSQC (red correlations) and ^{1}H-^{13}C H2BC (black correlations) NMR spectra of ***(R)*-20a** (Acetone-d_6).

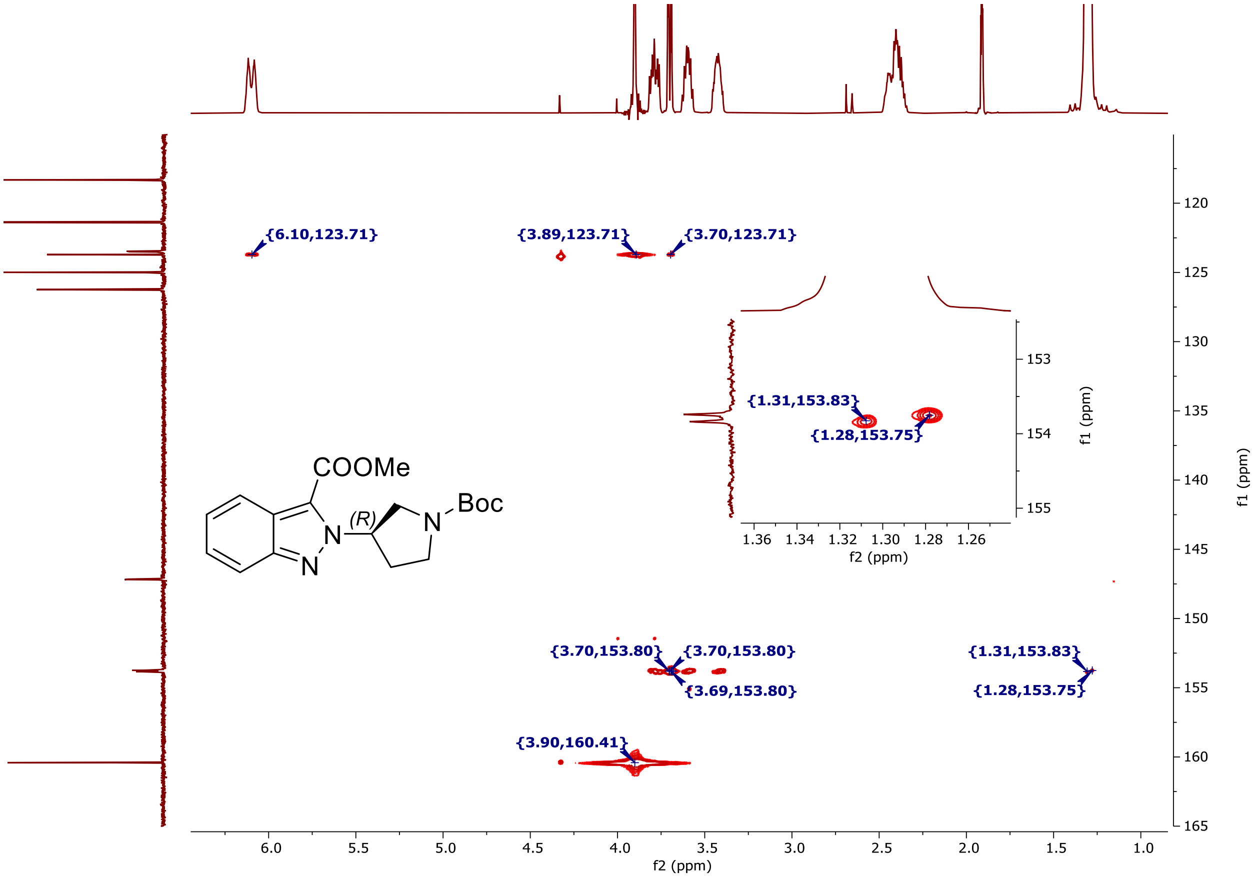

Figure S143. The 2Hz optimized 2D band-selective ^{1}H-^{13}C HMBC NMR spectrum of ***(R)*-20a** (Acetone-d_6).

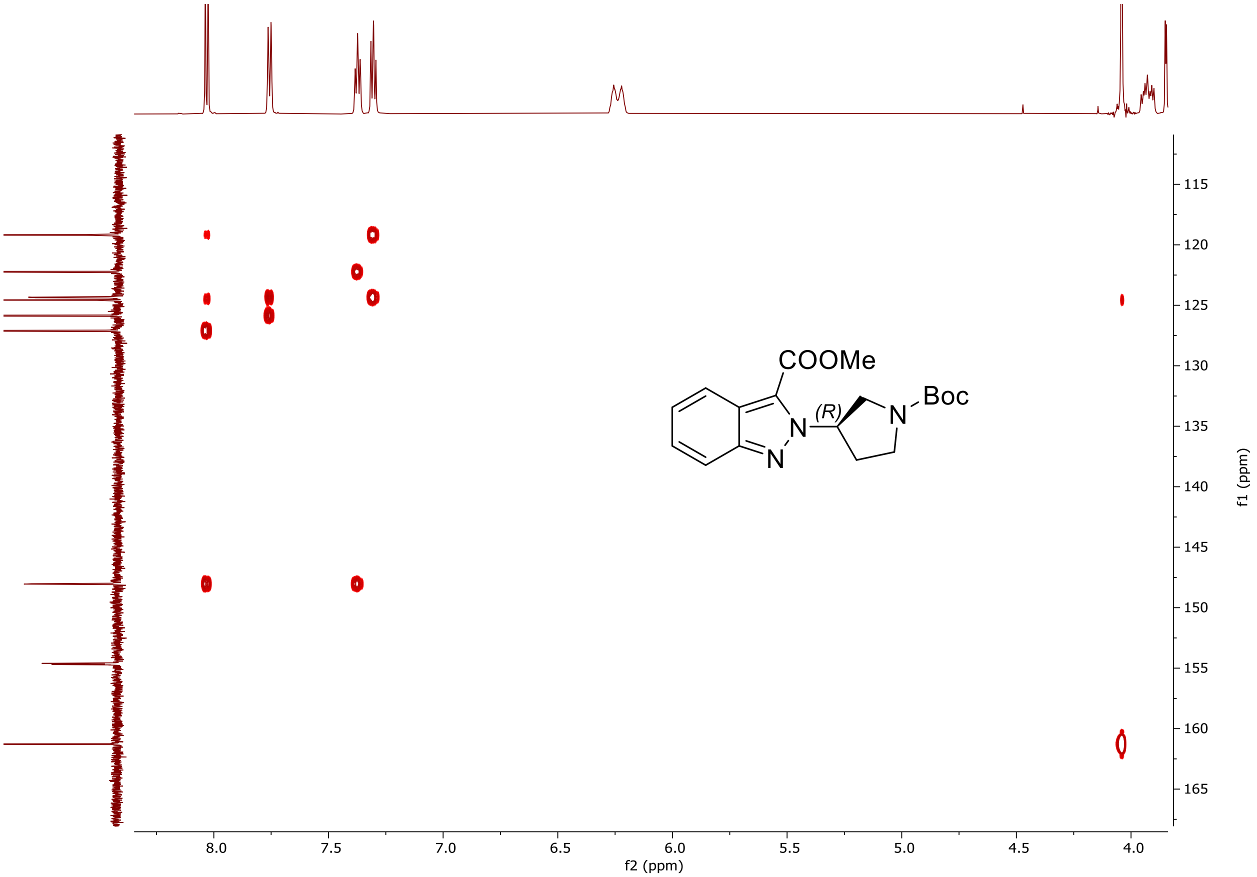

Figure S144. The 8Hz optimized ^{1}H-^{13}C HMBC NMR spectrum of ***(R)*-20a** (Acetone-d_6).

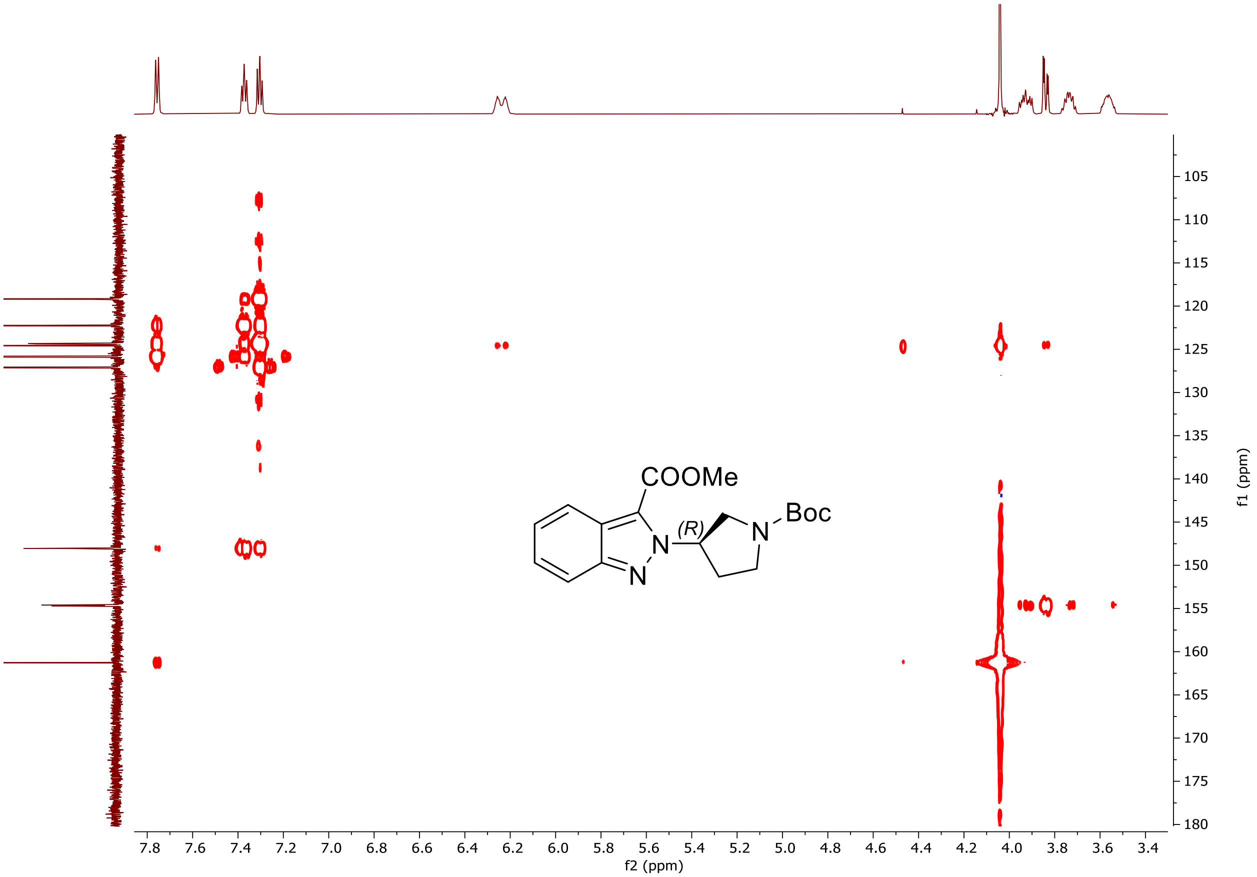

Figure S145. The 2Hz optimized ^{1}H-^{13}C HMBC NMR spectrum of ***(R)*-20a** (Acetone-d_6).

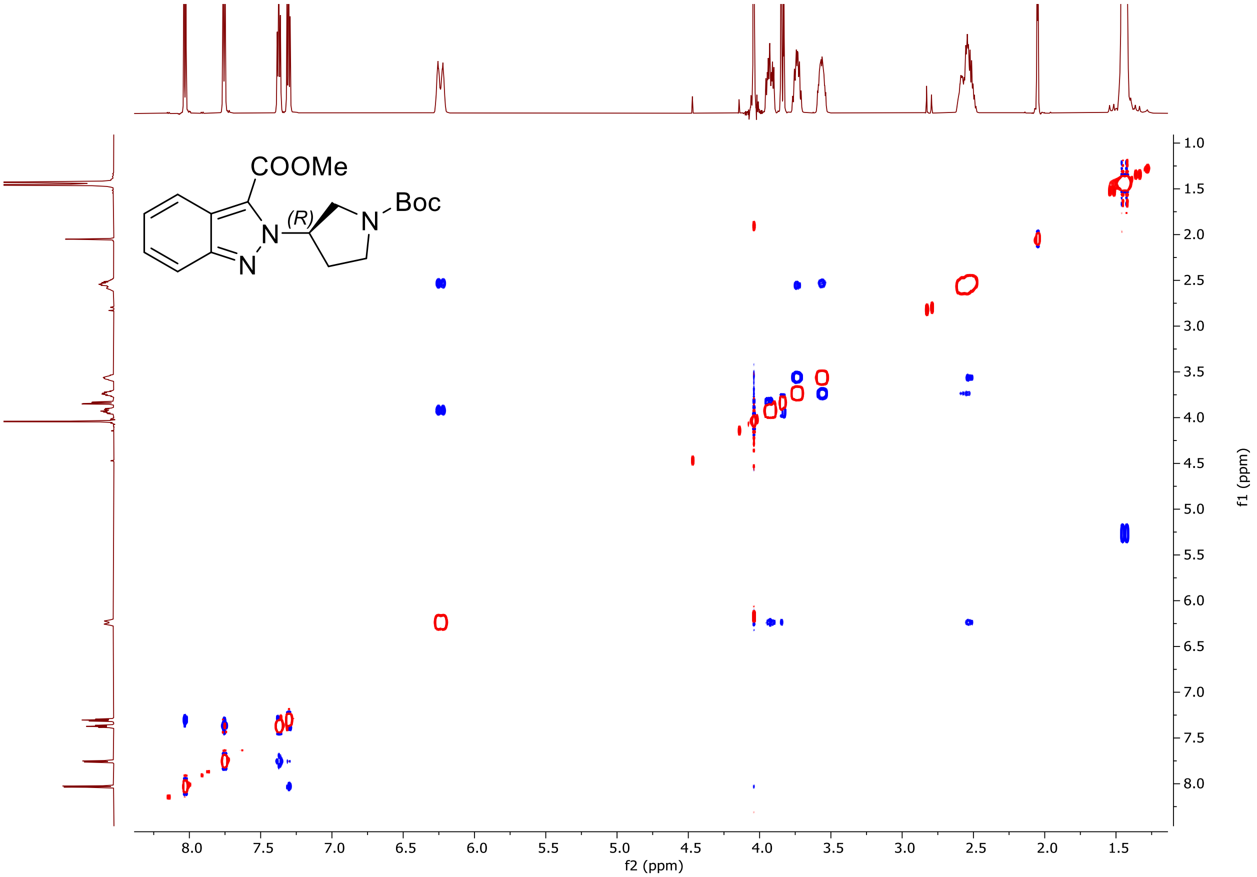

Figure S146. ^{1}H-^{1}H NOESY NMR spectrum of ***(R)*-20a** (Acetone-d_6).

Acquisition Parameter

Source Type	ESI	Ion Polarity	Positive	Set Nebulizer	0.4 Bar
Focus	Not active	Set Capillary	4500 V	Set Dry Heater	180 °C
Scan Begin	50 m/z	Set End Plate Offset	-500 V	Set Dry Gas	4.0 l/min
Scan End	1000 m/z	Set Collision Cell RF	140.0 Vpp	Set Divert Valve	Waste

#	RT [min]	Area	Int. Type	I	S/N	Chromatogram	Max. m/z	FWHM [min]
n.a.	0.0	n.a.	Single spectrum	n.a.	n.a.	n.a.	304.2626	n.a.
n.a.	5.9	n.a.	Single spectrum	n.a.	n.a.	n.a.	368.1581	n.a.

+MS, 5.9min #353

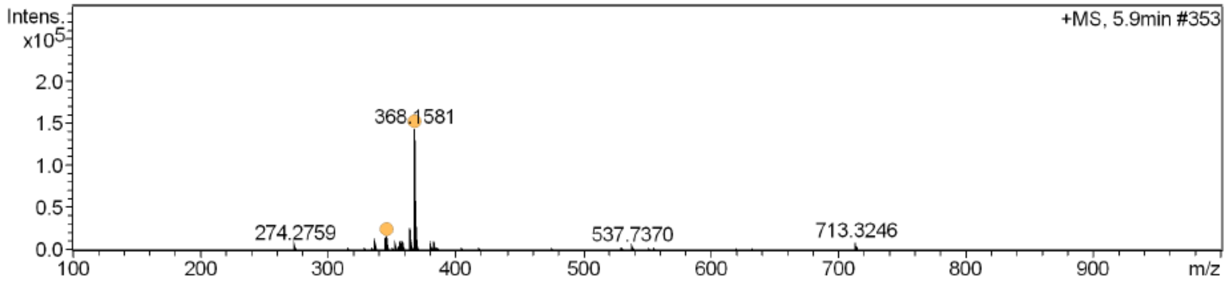

Meas. m/z	#	Ion Formula	m/z	err [ppm]	mSigma	# Sigma	Score	rdb	e⁻ Conf	N-Rule
346.1744	1	C18H24N3O4	346.1761	5.1	28.1	1	100.00	8.5	even	ok
368.1581	1	C18H23N3NaO4	368.1581	-0.2	2.6	1	100.00	8.5	even	ok

Figure S147. HRMS (ESI$^+$) report of ***(R)*-20a**.

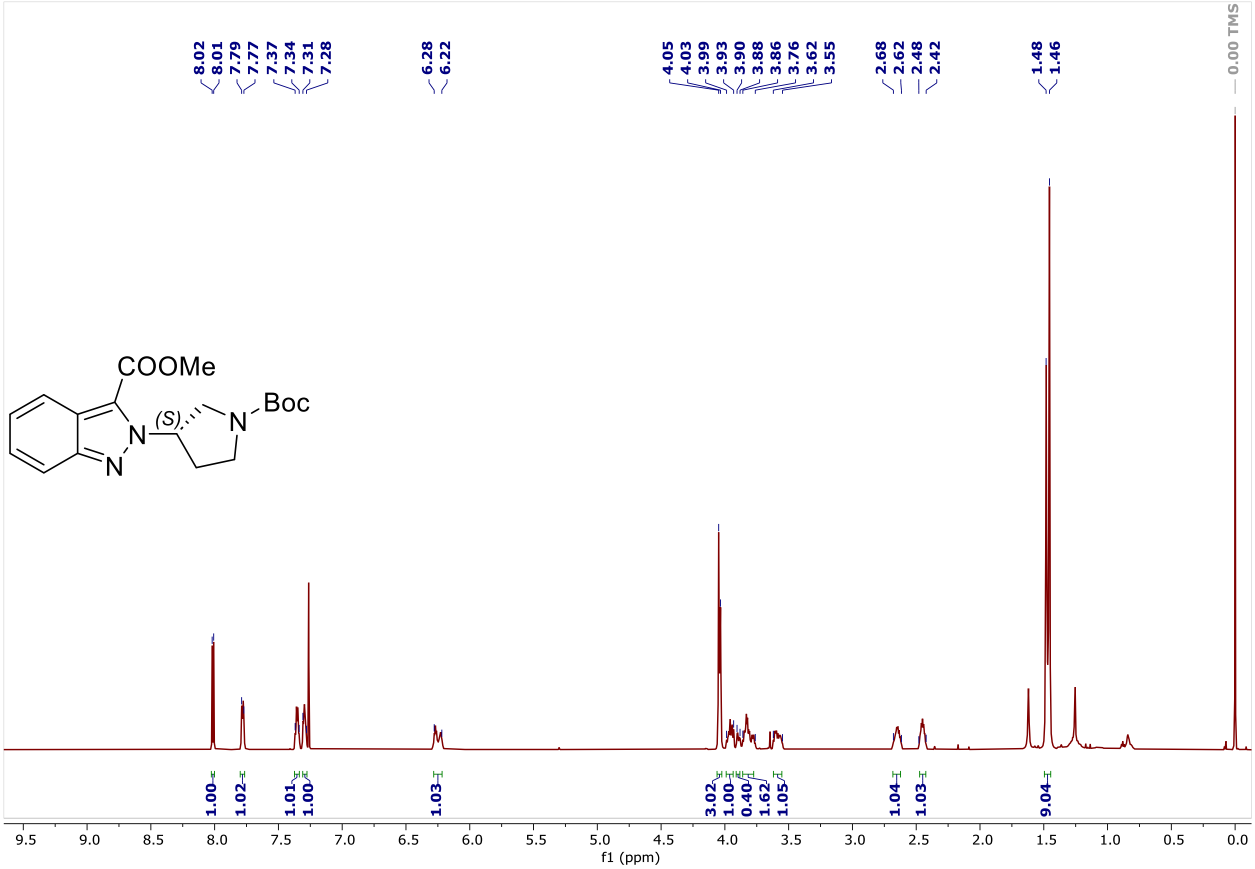

Figure S148. ^{1}H NMR (700 MHz, $CDCl_3$) spectrum of **(*S*)-20a**.

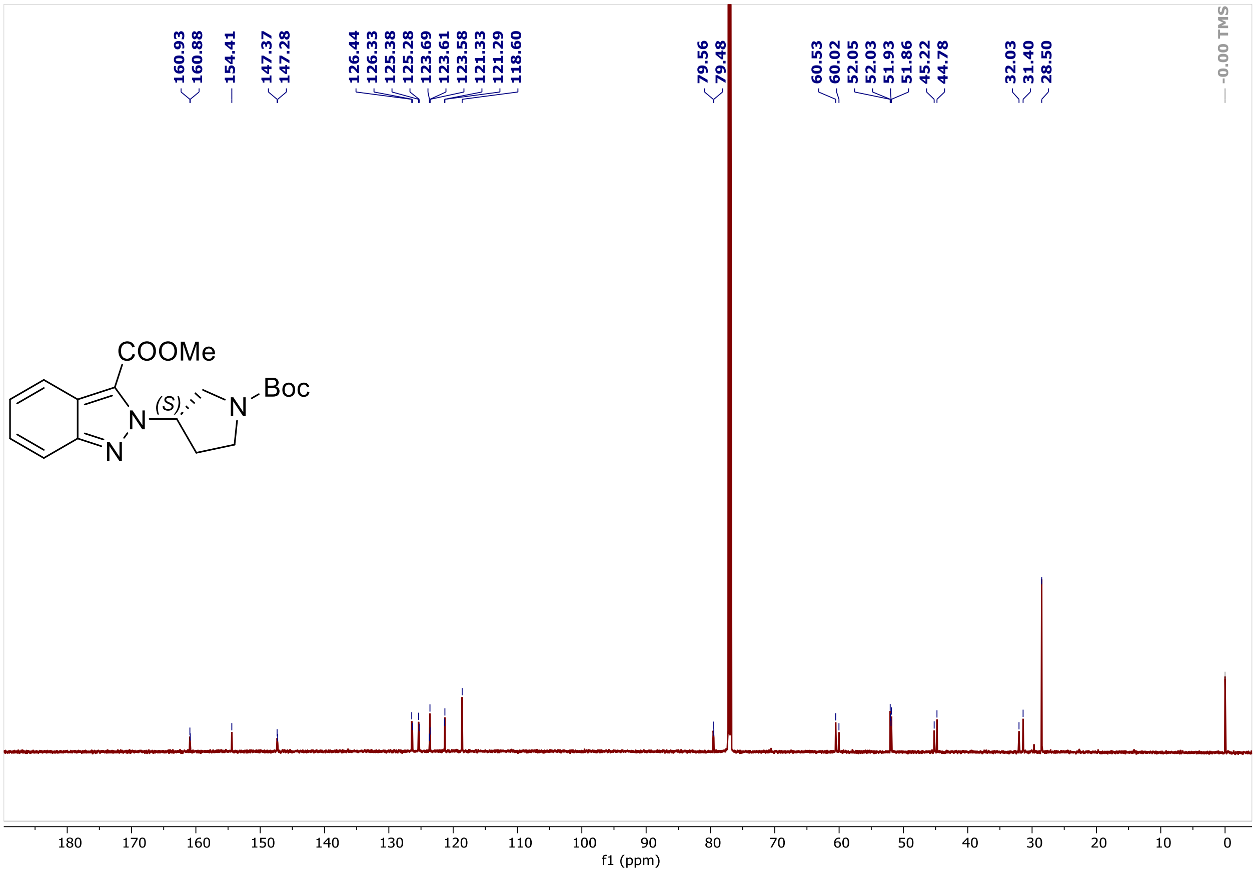

Figure S149. ^{13}C NMR (176 MHz, $CDCl_3$) spectrum of **(*S*)-20a**.

Acquisition Parameter

Source Type	ESI	Ion Polarity	Positive	Set Nebulizer	0.4 Bar
Focus	Not active	Set Capillary	4500 V	Set Dry Heater	180 °C
Scan Begin	50 m/z	Set End Plate Offset	-500 V	Set Dry Gas	4.0 l/min
Scan End	1000 m/z	Set Collision Cell RF	140.0 Vpp	Set Divert Valve	Waste

#	RT [min]	Area	Int. Type	I	S/N	Chromatogram	Max. m/z	FWHM [min]
n.a.	0.1	n.a.	Single spectrum	n.a.	n.a.	n.a.	304.2631	n.a.
n.a.	7.0	n.a.	Single spectrum	n.a.	n.a.	n.a.	368.1583	n.a.

+MS, 7.0min #417

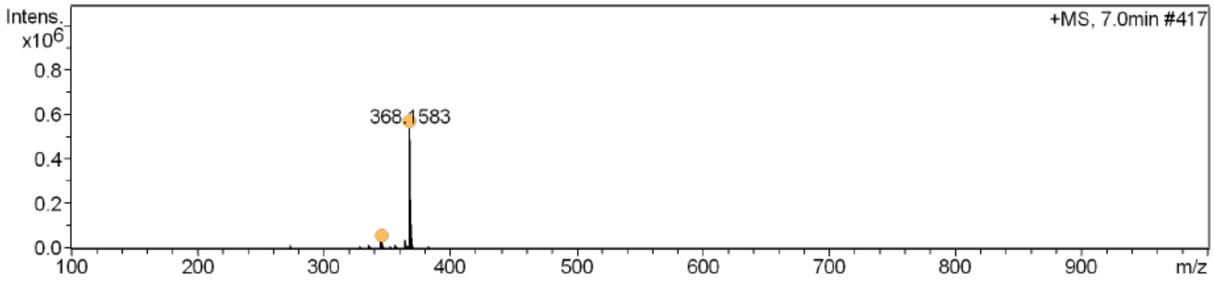

Meas. m/z	#	Ion Formula	m/z	err [ppm]	mSigma	# Sigma	Score	rdb	e¯ Conf	N-Rule
346.1740	1	C18H24N3O4	346.1761	6.3	35.7	1	100.00	8.5	even	ok
368.1583	1	C18H23N3NaO4	368.1581	-0.6	6.2	1	100.00	8.5	even	ok

Figure S150. HRMS (ESI$^+$) report of ***(S)*-20a**.

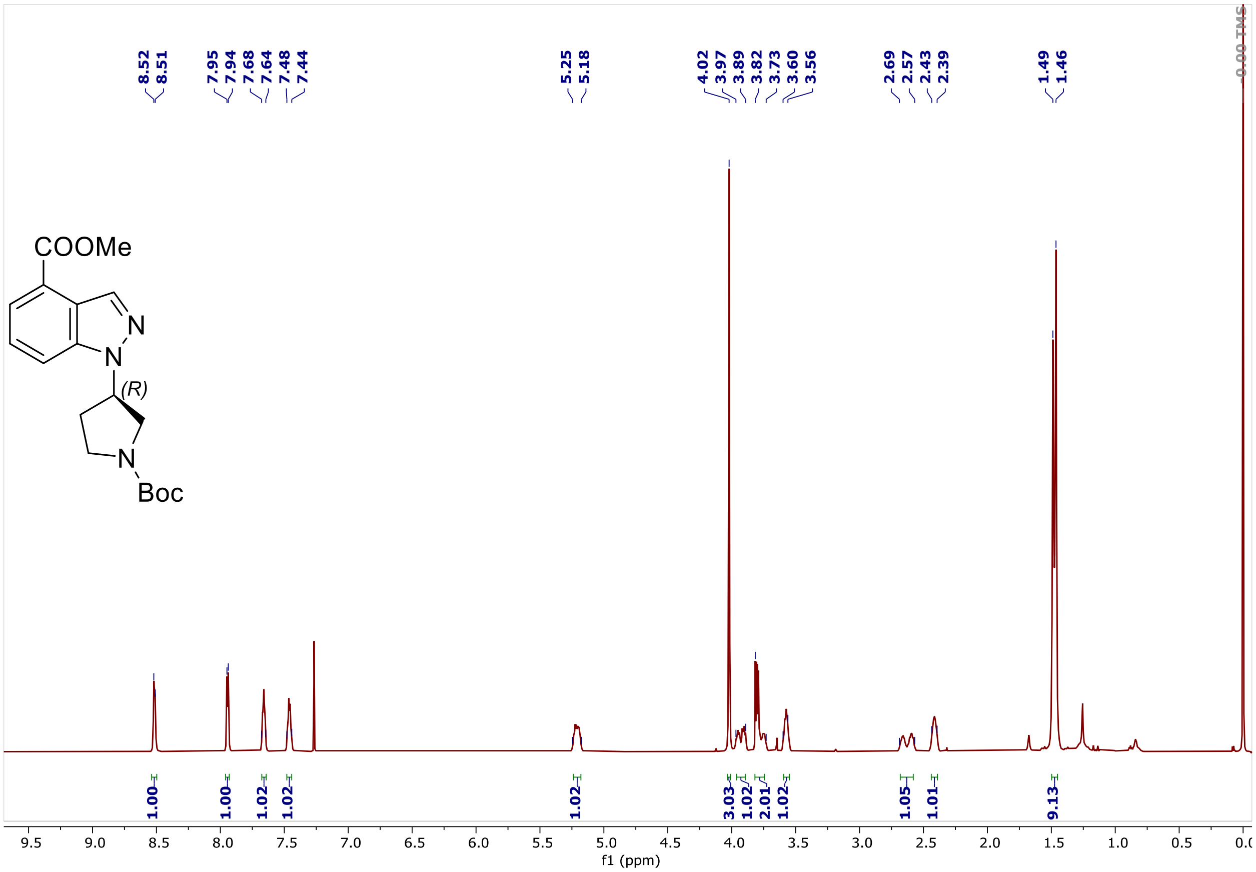

Figure S151. ^{1}H NMR (700 MHz, $CDCl_3$) spectrum of ***(R)*-19b**.

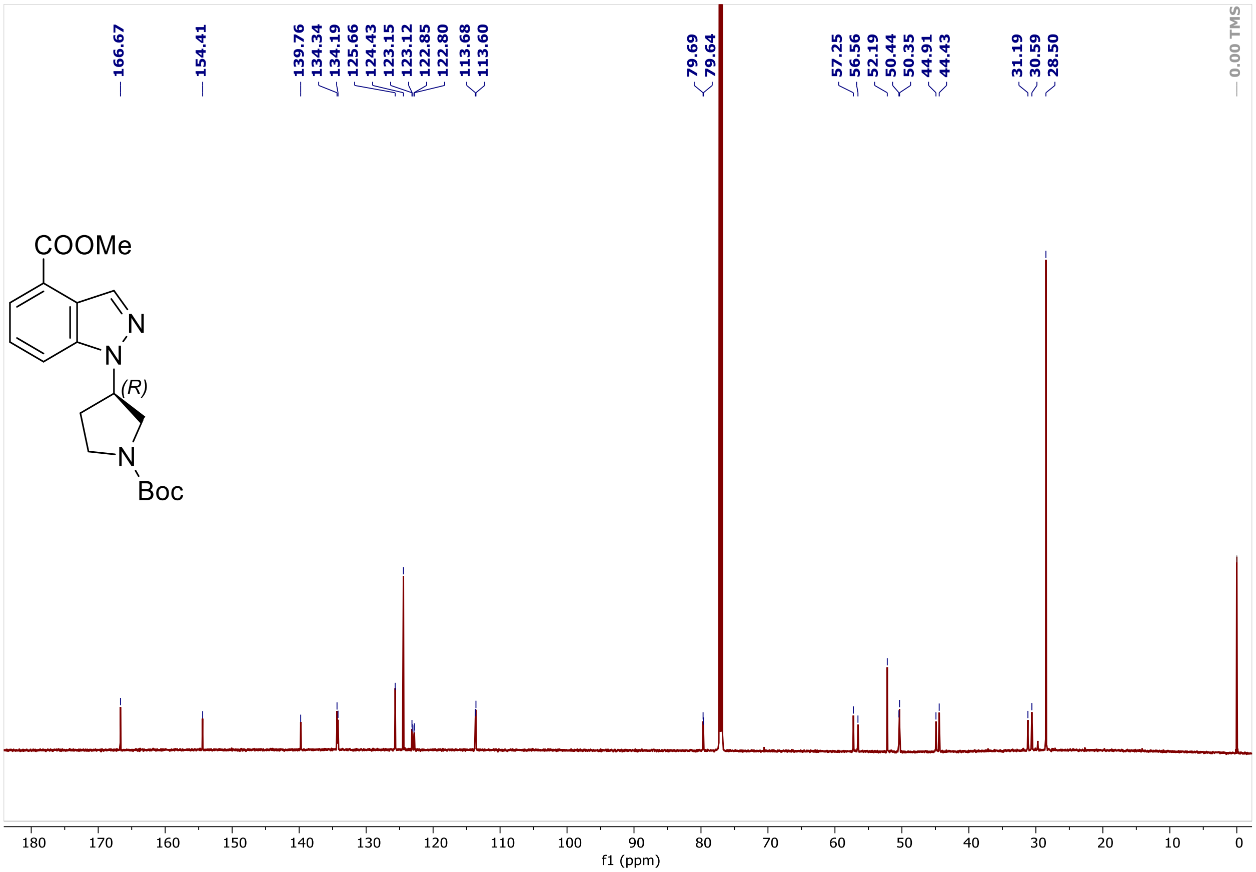

Figure S152. ^{13}C NMR (176 MHz, $CDCl_3$) spectrum of ***(R)*-19b**.

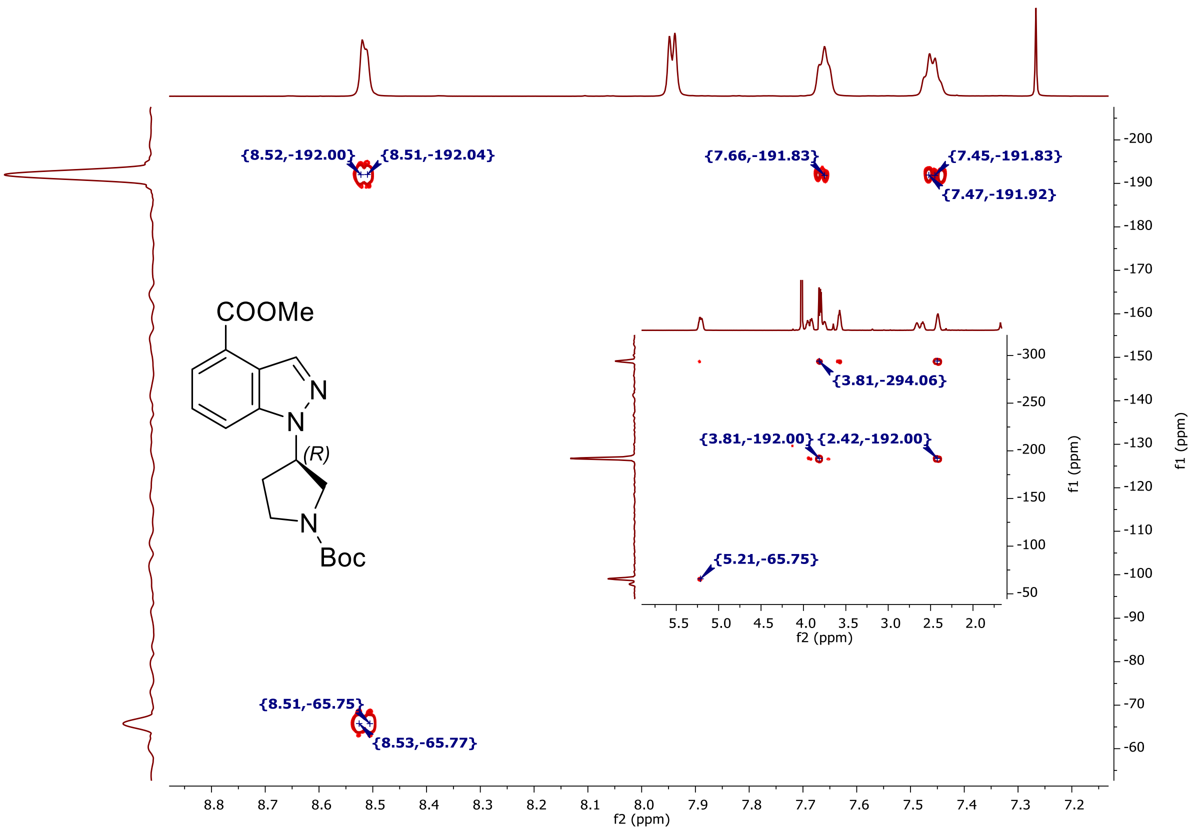

Figure S153. ^{1}H-^{15}N HMBC NMR spectrum of ***(R)*-19b** (71 MHz, $CDCl_3$).

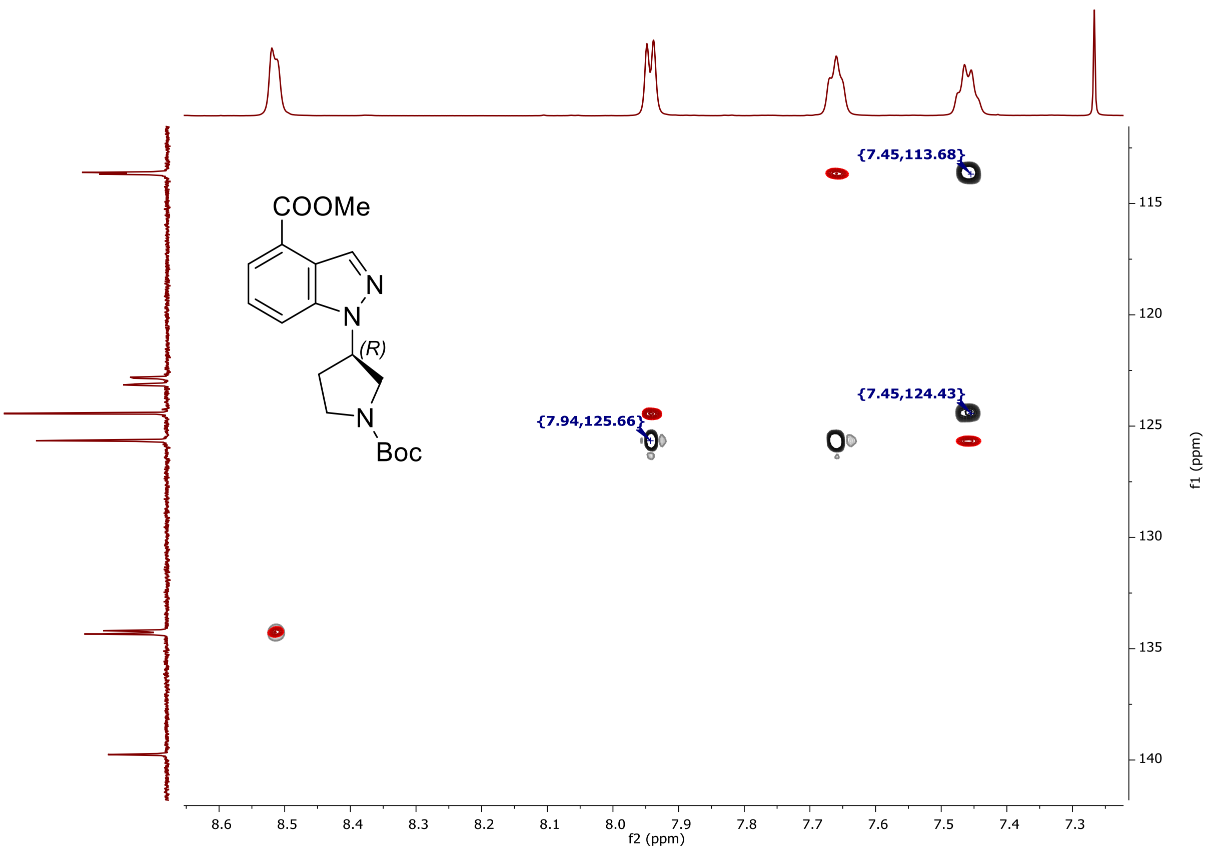

Figure S154. Overlaid multiplicity-edited ^{1}H-^{13}C HSQC (red correlations) and ^{1}H-^{13}C H2BC (black correlations) NMR spectra of ***(R)*-19b** ($CDCl_3$).

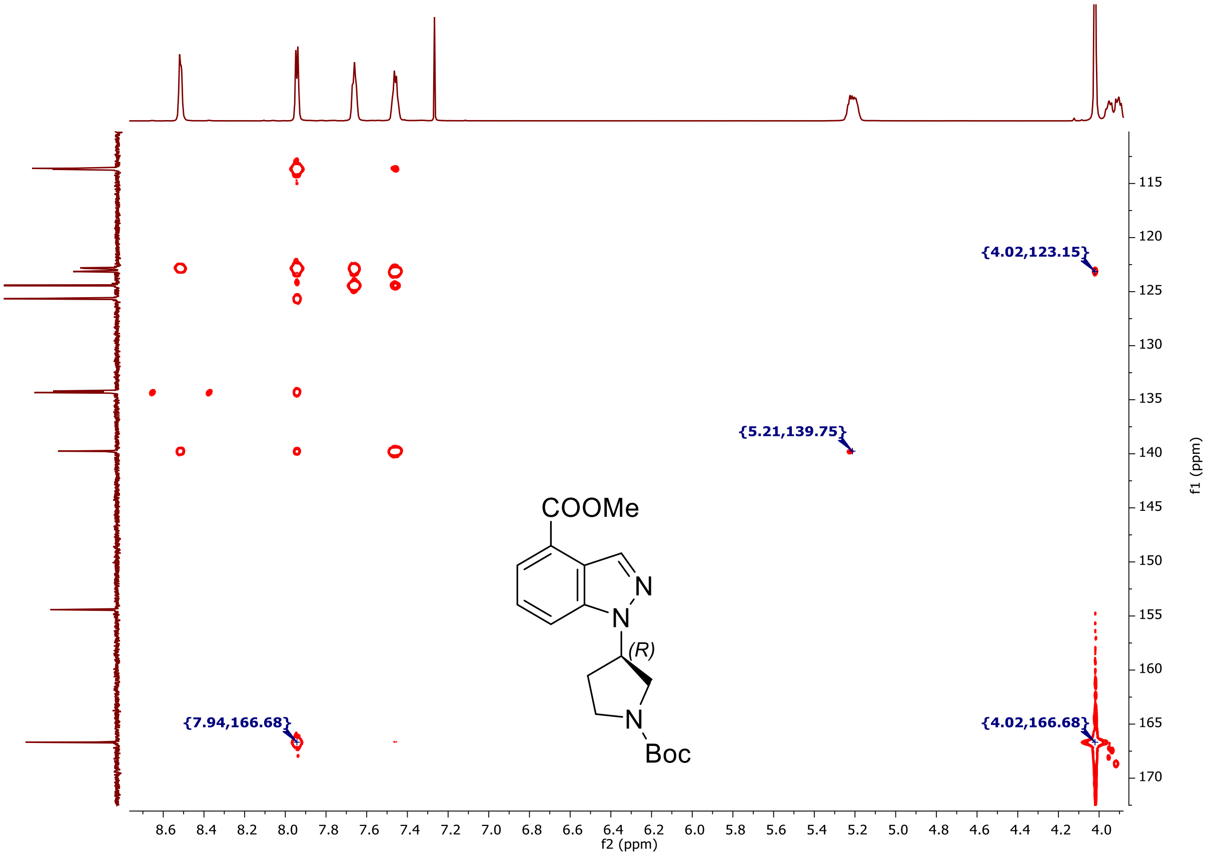

Figure S155. The 8Hz optimized ^{1}H-^{13}C HMBC NMR spectrum of ***(R)*-19b** ($CDCl_3$).

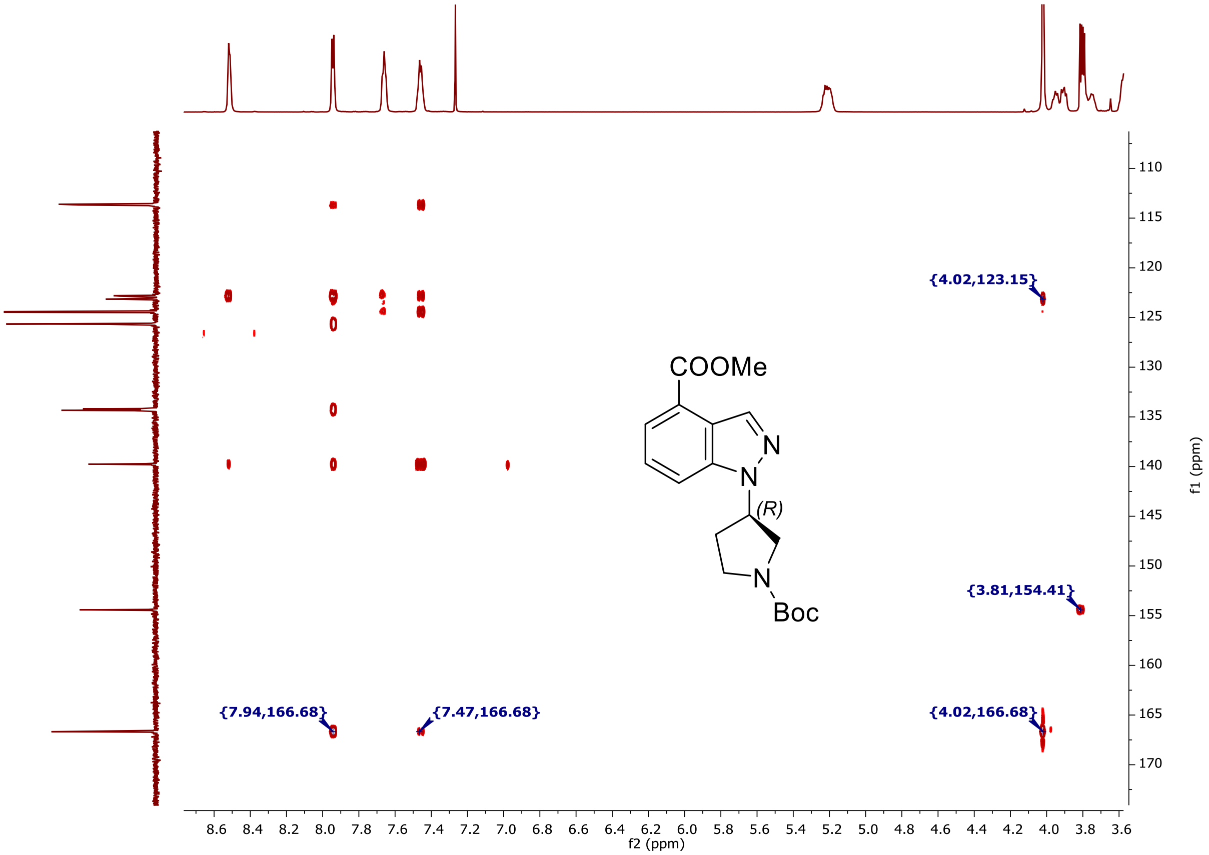

Figure S156. The 2Hz optimized ^{1}H-^{13}C HMBC NMR spectrum of ***(R)*-19b** ($CDCl_3$).

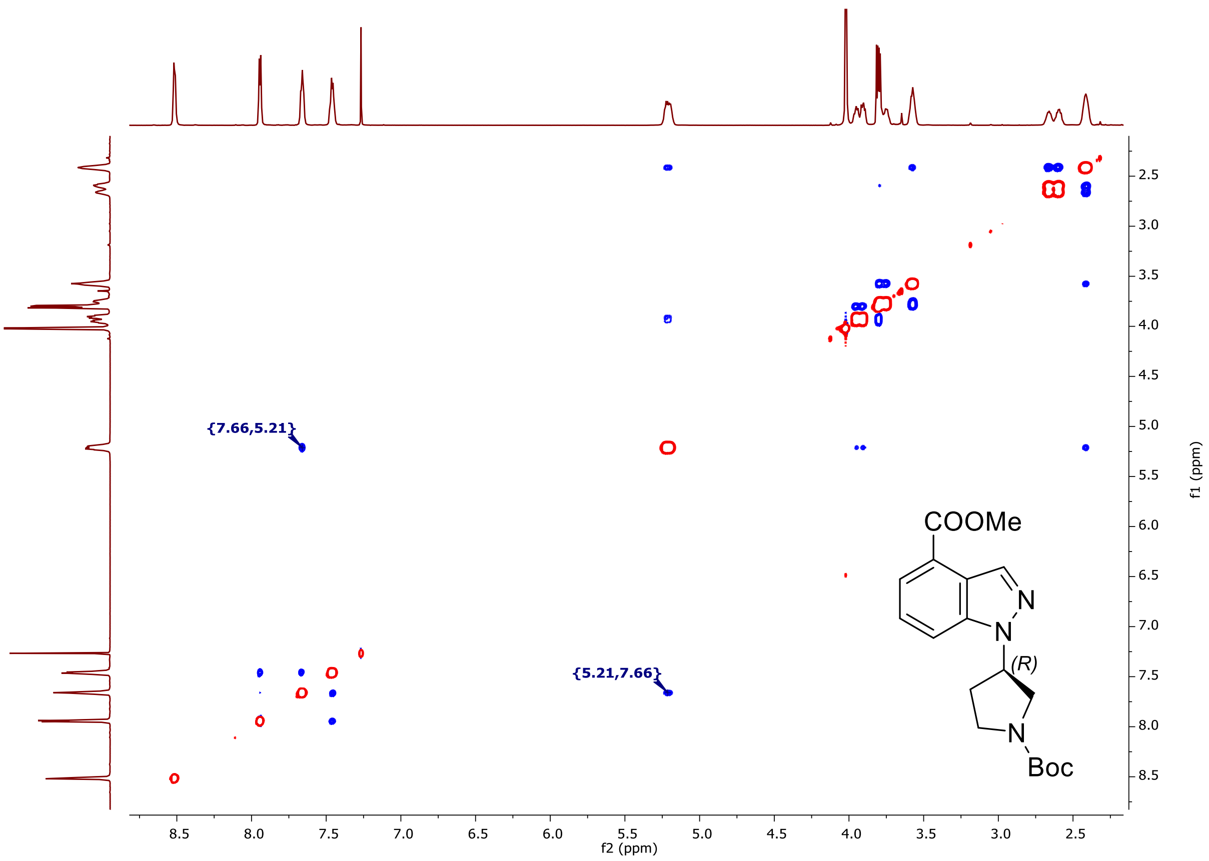

Figure S157. ^{1}H-^{1}H NOESY NMR spectrum of ***(R)*-19b** ($CDCl_3$).

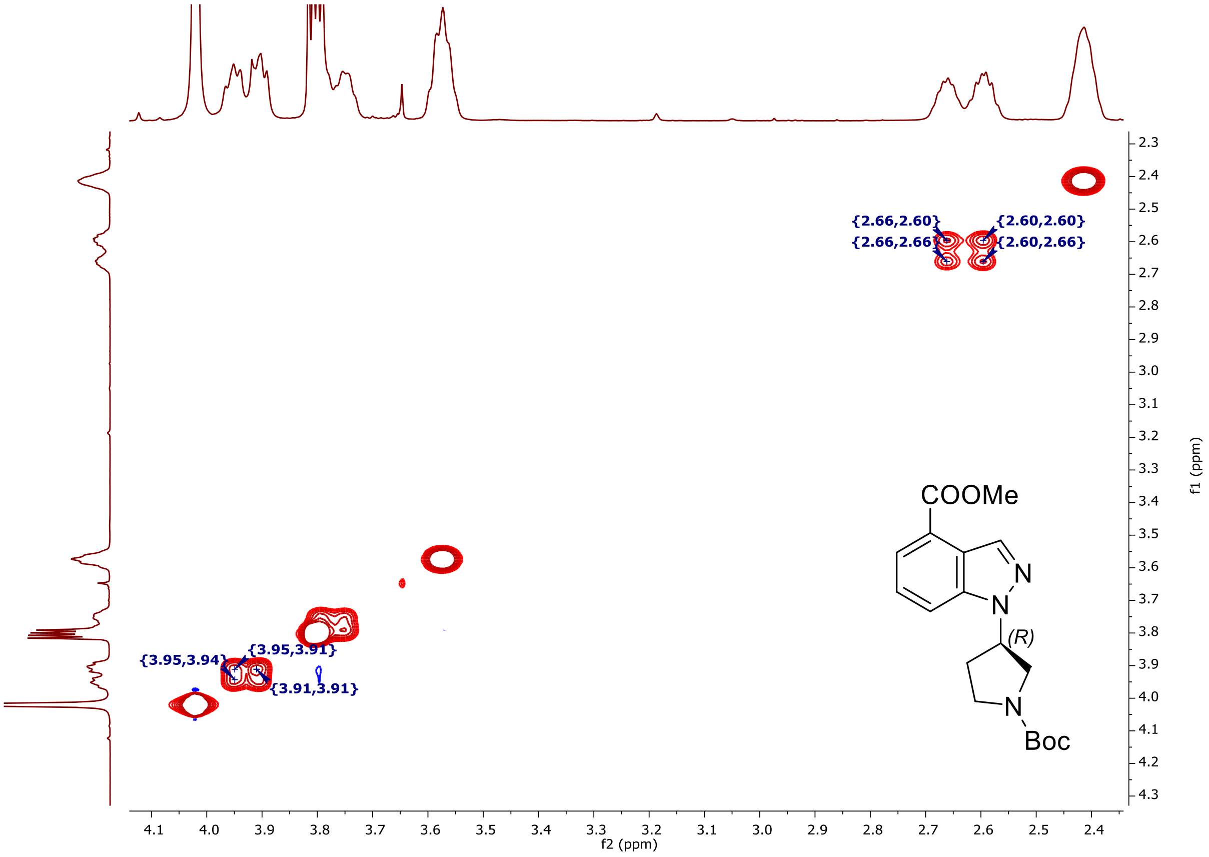

Figure S158. ^{1}H-^{1}H NOESY/EXSY NMR spectrum of (***R***)-**19b** ($CDCl_3$).

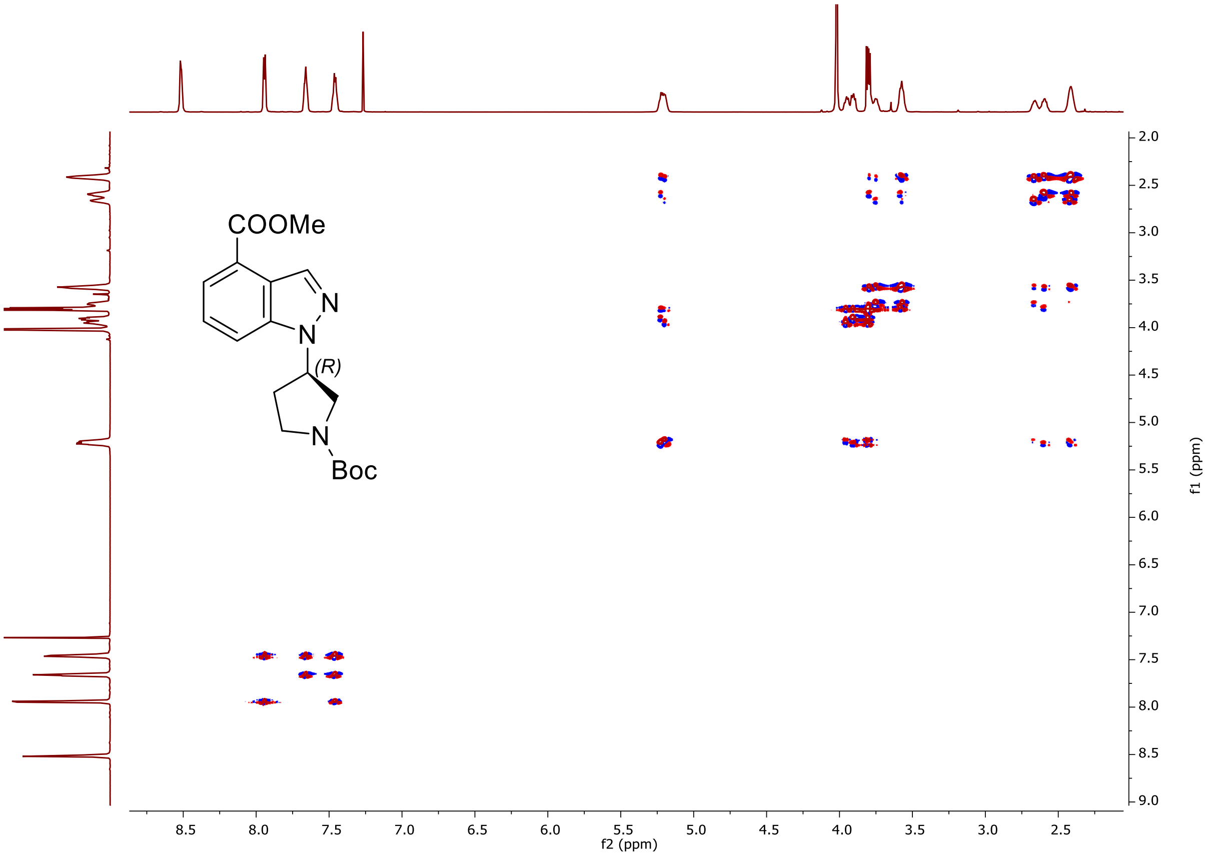

Figure S159. ^1H-^1H COSY NMR spectrum of ***(R)*-19b** ($CDCl_3$).

Acquisition Parameter

Source Type	ESI	Ion Polarity	Positive	Set Nebulizer	0.4 Bar
Focus	Not active	Set Capillary	4500 V	Set Dry Heater	180 °C
Scan Begin	50 m/z	Set End Plate Offset	-500 V	Set Dry Gas	4.0 l/min
Scan End	1000 m/z	Set Collision Cell RF	140.0 Vpp	Set Divert Valve	Waste

#	RT [min]	Area	Int. Type	I	S/N	Chromatogram	Max. m/z	FWHM [min]
n.a.	0.4	n.a.	Single spectrum	n.a.	n.a.	n.a.	304.2616	n.a.
n.a.	4.1	n.a.	Single spectrum	n.a.	n.a.	n.a.	368.1581	n.a.

+MS, 4.1min #243

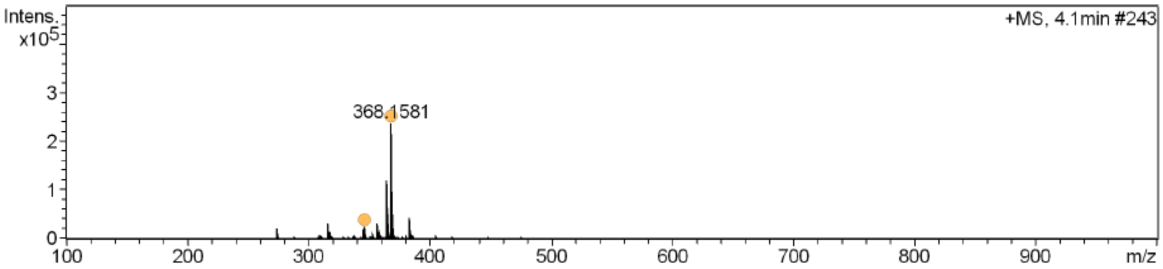

Meas. m/z	#	Ion Formula	m/z	err [ppm]	mSigma	# Sigma	Score	rdb	e⁻ Conf	N-Rule
346.1756	1	C18H24N3O4	346.1761	1.5	18.9	1	100.00	8.5	even	ok
368.1581	1	C18H23N3NaO4	368.1581	0.2	10.3	1	100.00	8.5	even	ok

Figure S160. HRMS (ESI^+) report of **(*R*)-19b**.

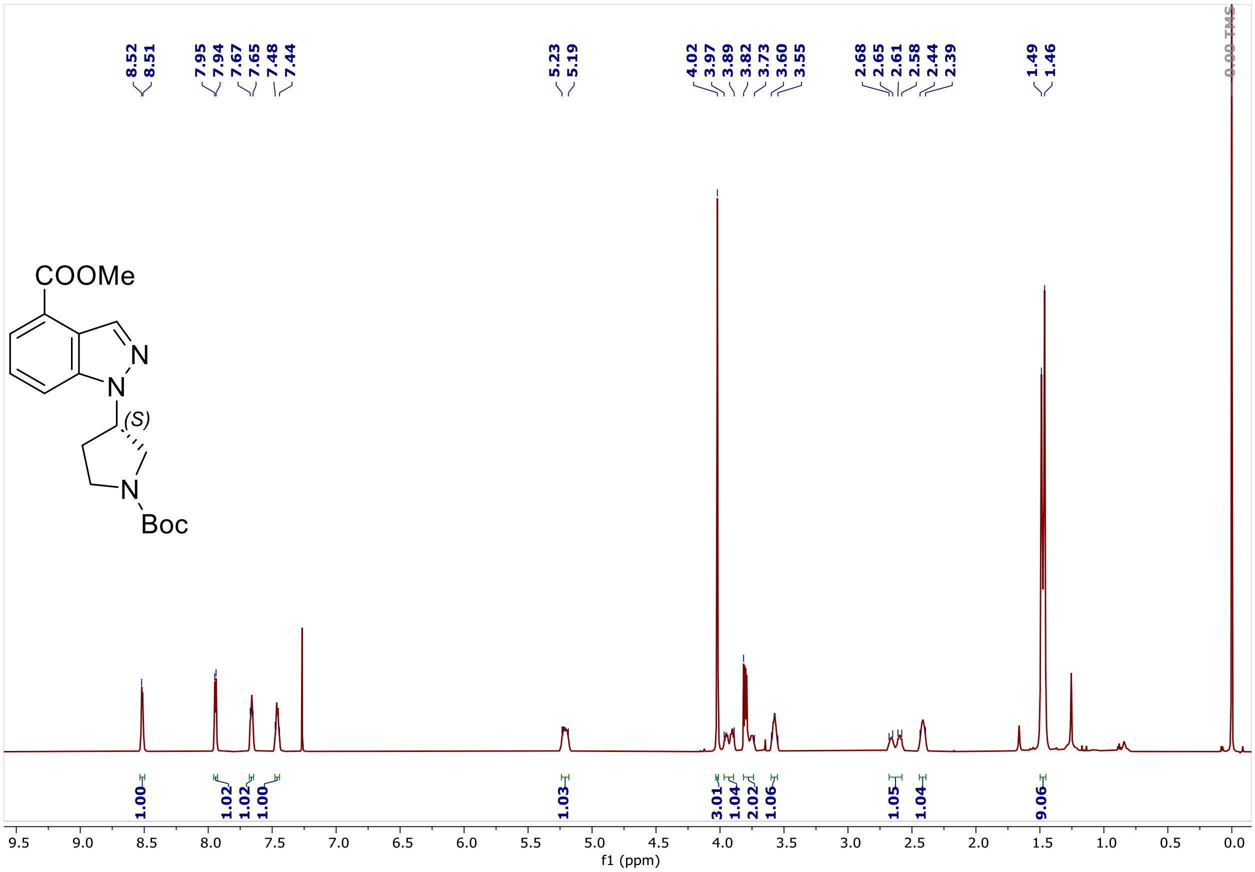

Figure S161. ^{1}H NMR (700 MHz, $CDCl_3$) spectrum of ***(S)*-19b**.

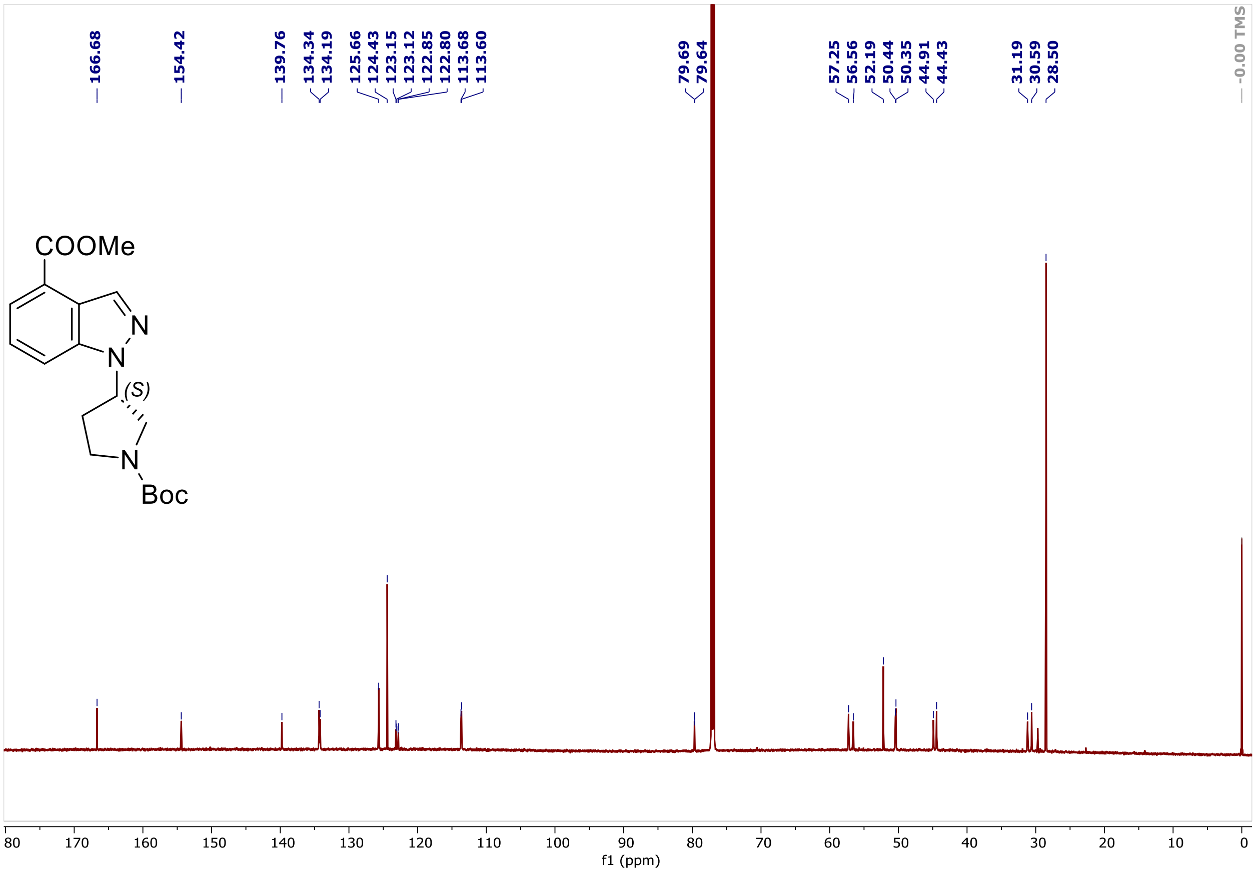

Figure S162. ^{13}C NMR (176 MHz, $CDCl_3$) spectrum of ***(S)*-19b**.

Acquisition Parameter

Source Type	ESI	Ion Polarity	Positive	Set Nebulizer	0.4 Bar
Focus	Not active	Set Capillary	4500 V	Set Dry Heater	180 °C
Scan Begin	50 m/z	Set End Plate Offset	-500 V	Set Dry Gas	4.0 l/min
Scan End	1000 m/z	Set Collision Cell RF	140.0 Vpp	Set Divert Valve	Waste

#	RT [min]	Area	Int. Type	I	S/N	Chromatogram	Max. m/z	FWHM [min]
n.a.	0.2	n.a.	Single spectrum	n.a.	n.a.	n.a.	304.2637	n.a.
n.a.	5.8	n.a.	Single spectrum	n.a.	n.a.	n.a.	368.1583	n.a.

+MS, 5.8min #348

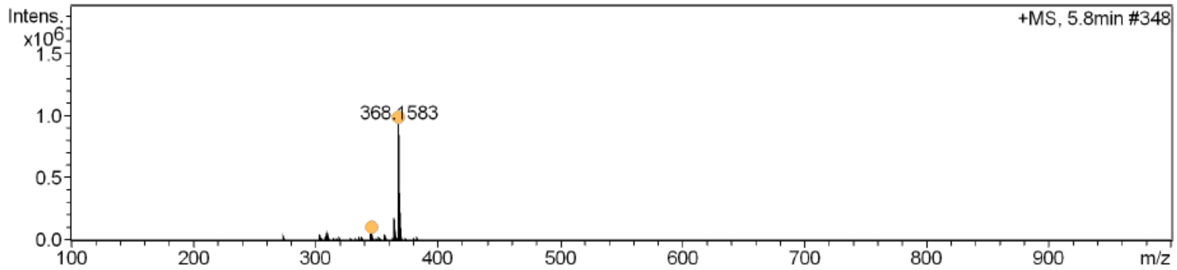

Meas. m/z	#	Ion Formula	m/z	err [ppm]	mSigma	# Sigma	Score	rdb	e⁻ Conf	N-Rule
346.1746	1	C18H24N3O4	346.1761	4.4	28.2	1	100.00	8.5	even	ok
368.1583	1	C18H23N3NaO4	368.1581	-0.5	32.1	1	100.00	8.5	even	ok

Figure S163. HRMS (ESI$^+$) report of **(*S*)-19b**.

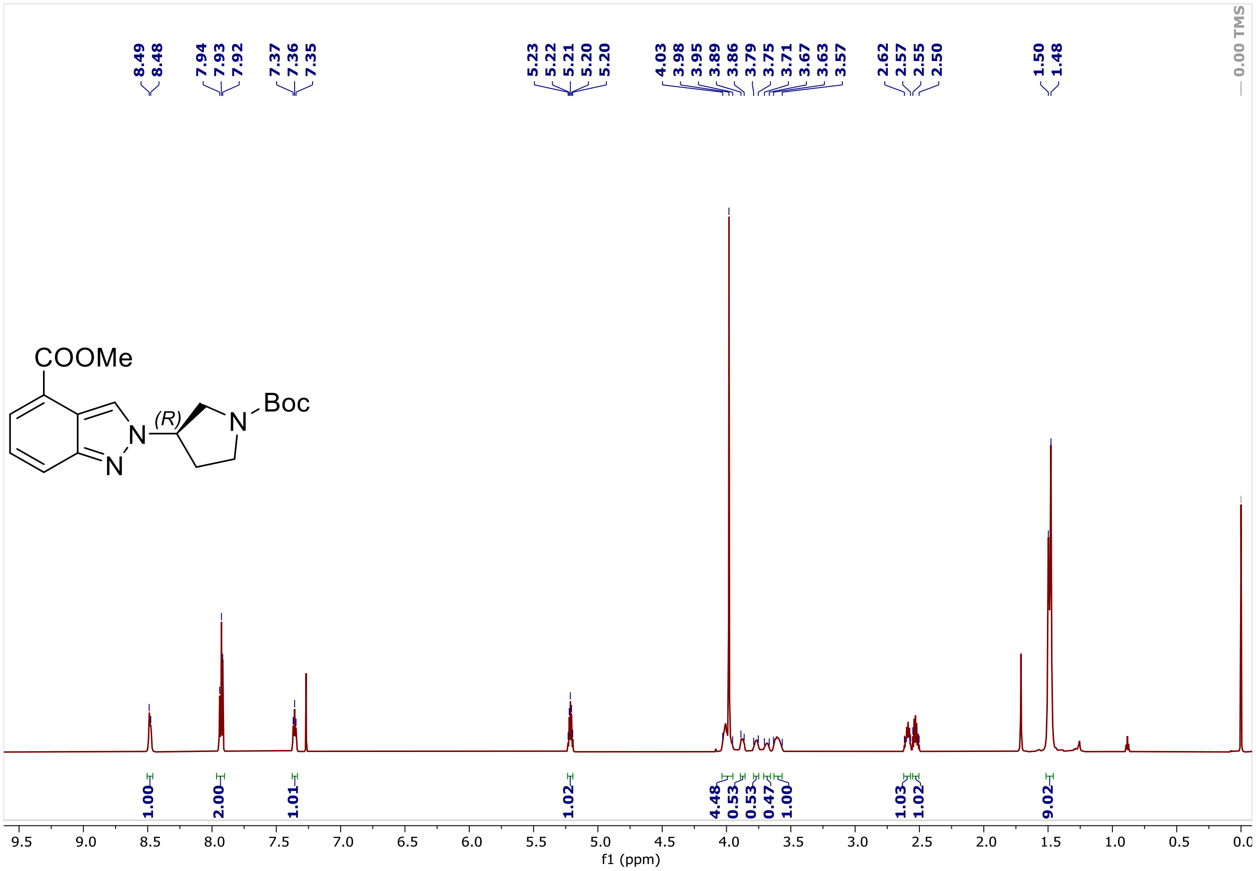

Figure S164. ^{1}H NMR (700 MHz, $CDCl_3$) spectrum of ***(R)*-20b**.

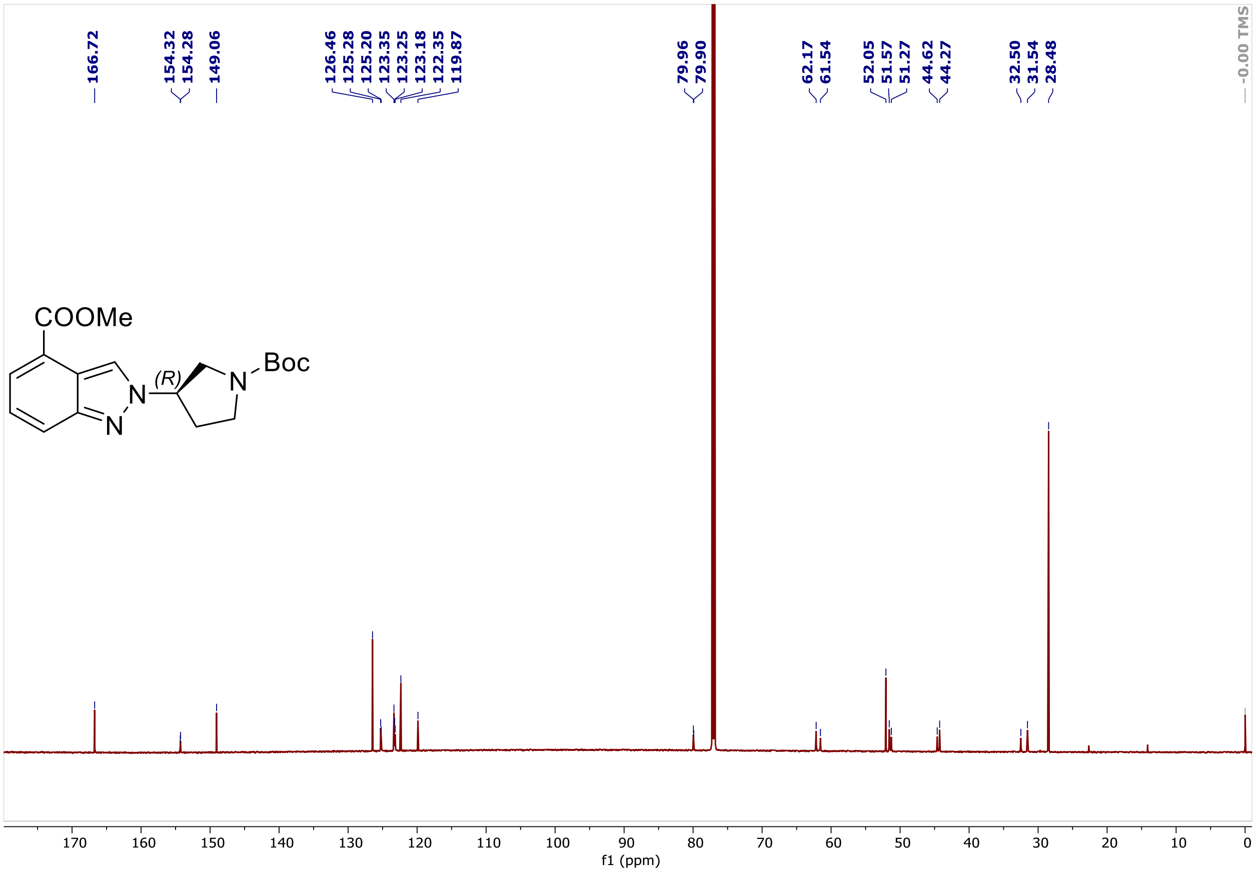

Figure S165. ^{13}C NMR (176 MHz, $CDCl_3$) spectrum of ***(R)*-20b**.

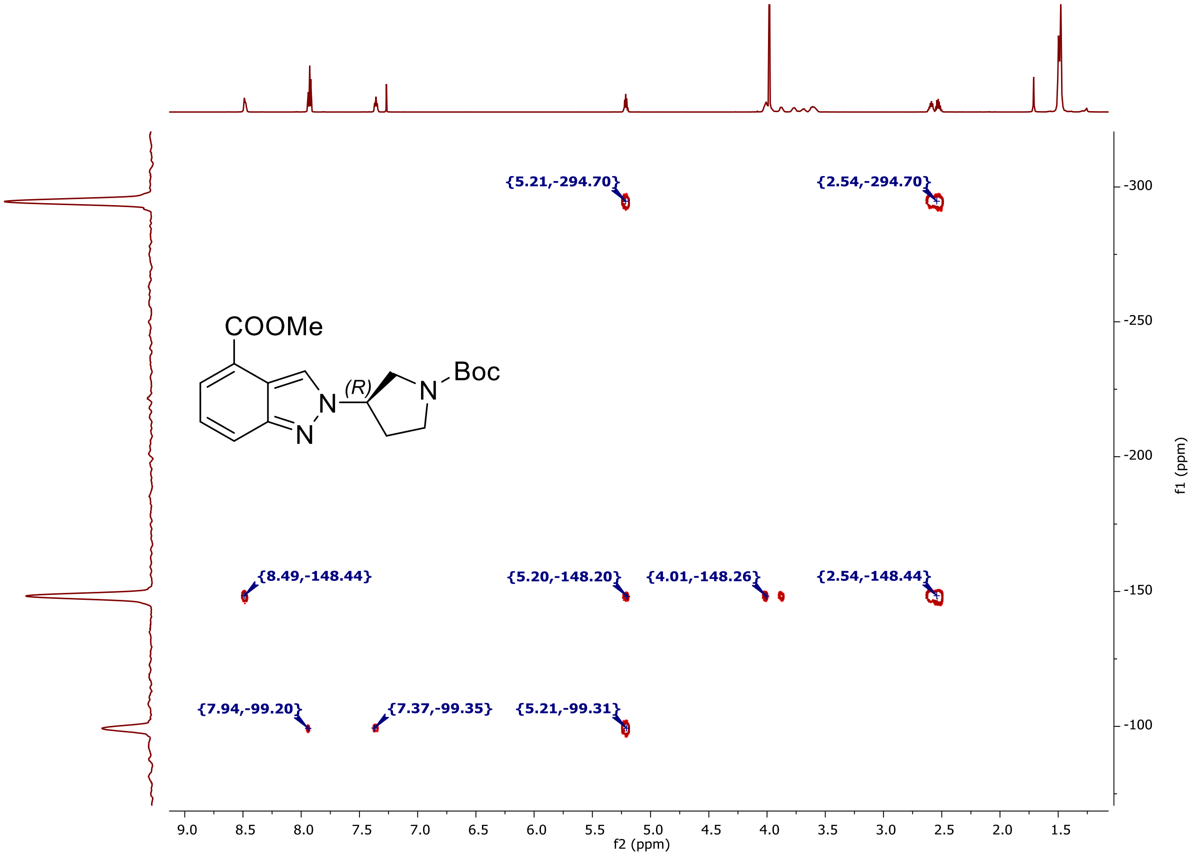

Figure S166. ^{1}H-^{15}N HMBC NMR spectrum of ***(R)*-20b** (71 MHz, $CDCl_3$).

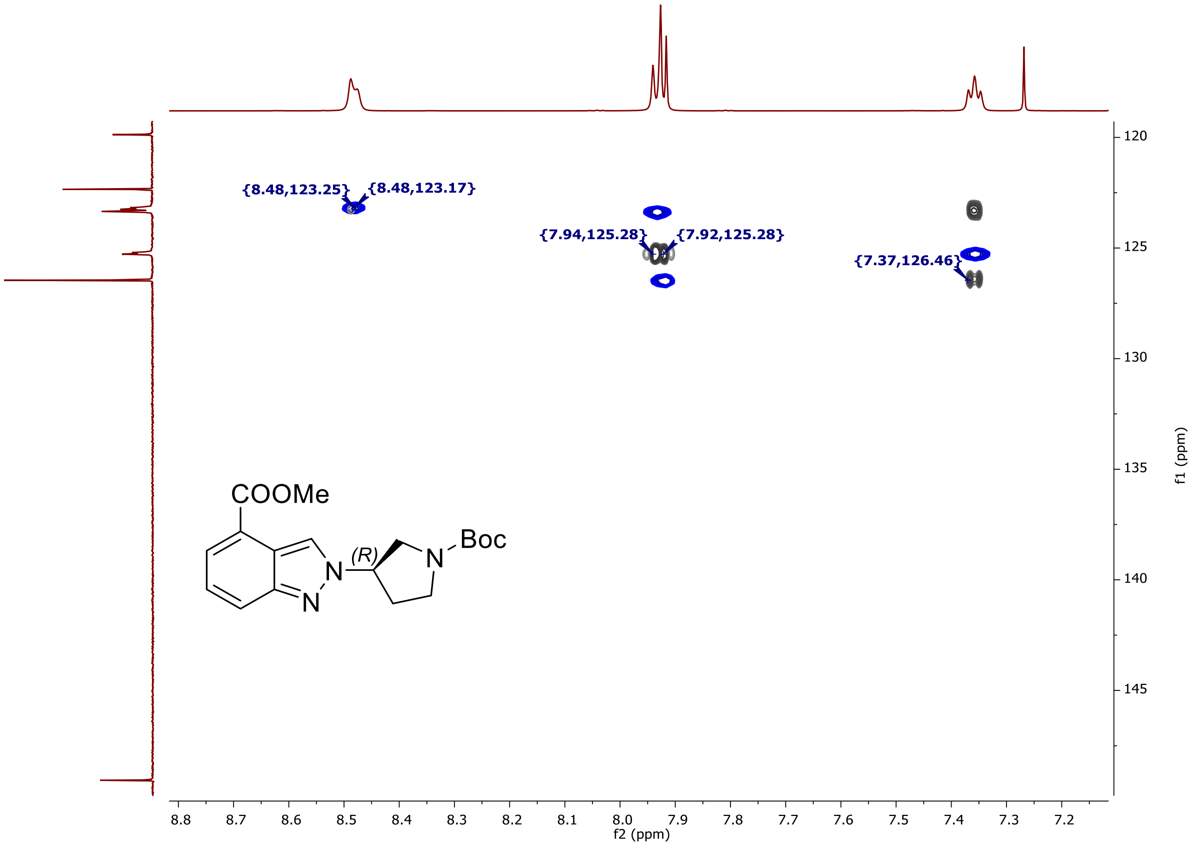

Figure S167. Overlaid multiplicity-edited ^{1}H-^{13}C HSQC (blue correlations) and ^{1}H-^{13}C H2BC (black correlations) NMR spectra of ***(R)*-20b** ($CDCl_3$).

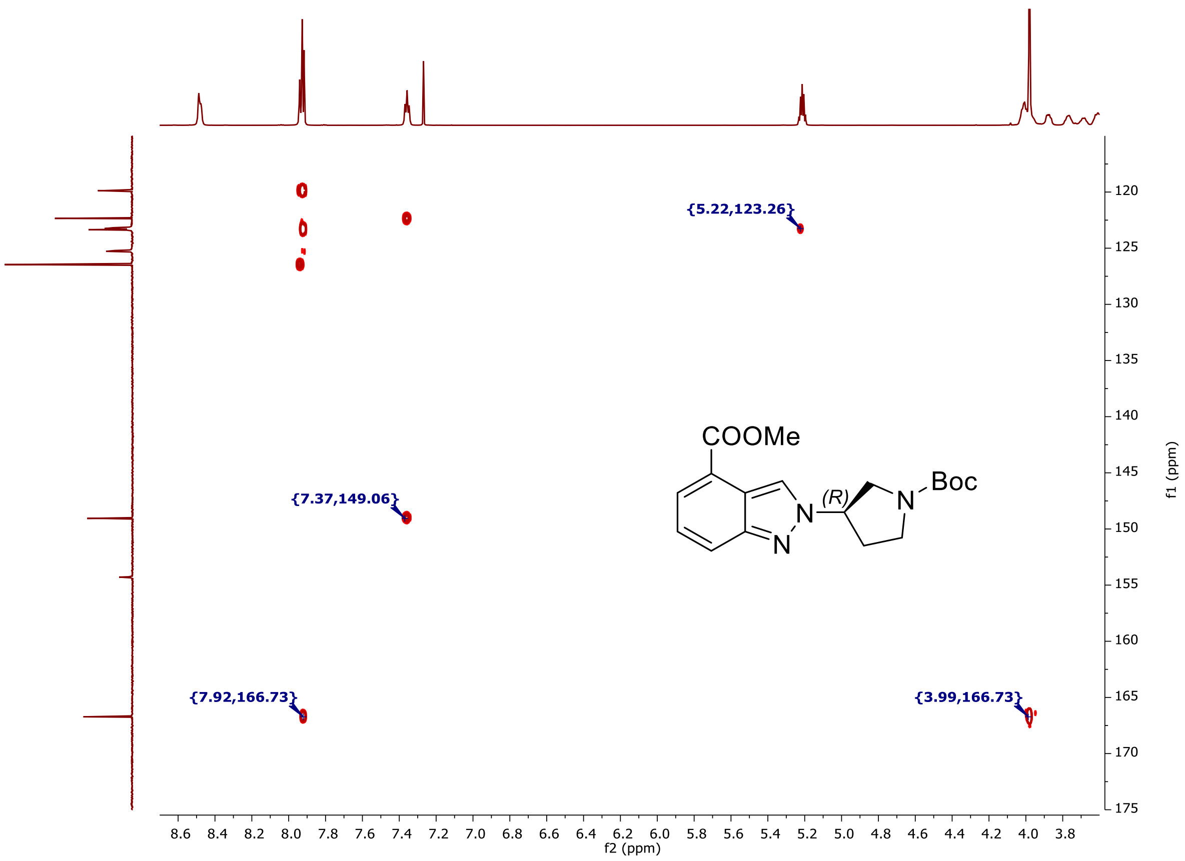

Figure S168. The 8Hz optimized ^{1}H-^{13}C HMBC NMR spectrum of ***(R)*-20b** ($CDCl_3$).

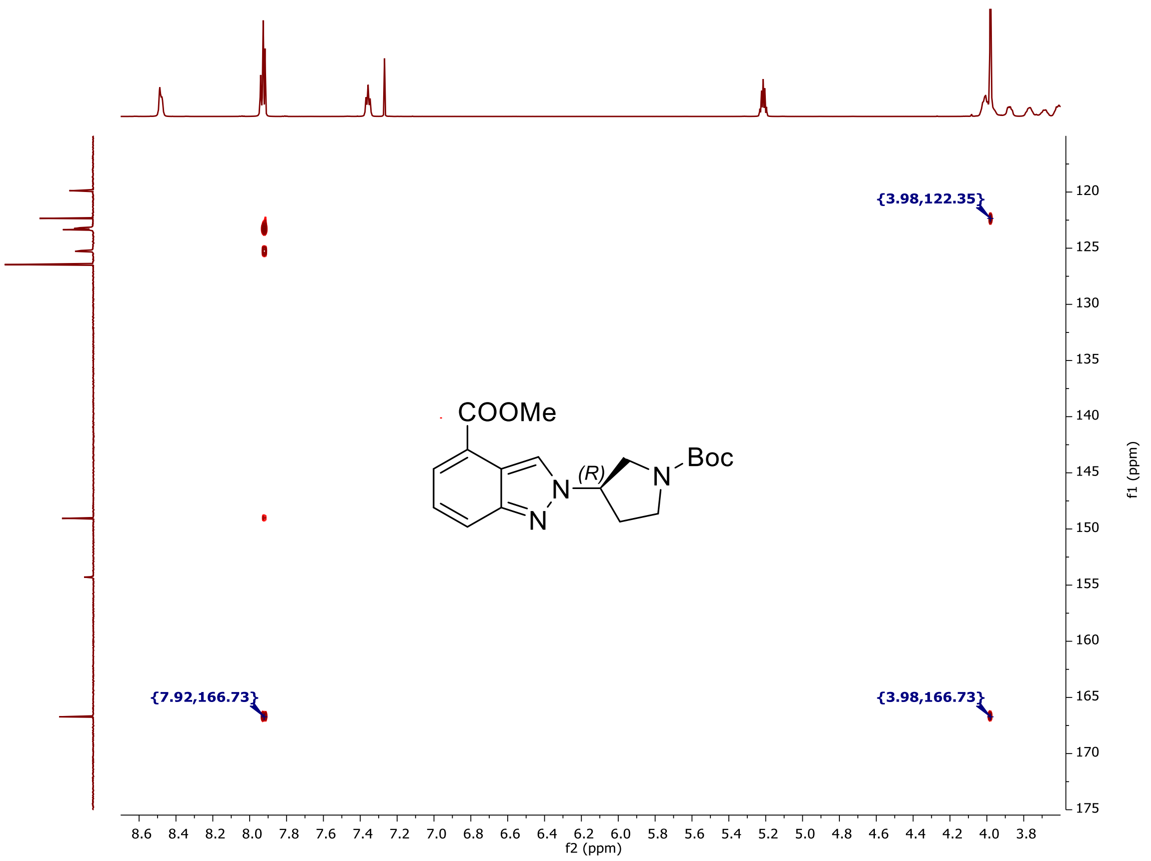

Figure S169. The 2Hz optimized ^{1}H-^{13}C HMBC NMR spectrum of ***(R)*-20b** ($CDCl_3$).

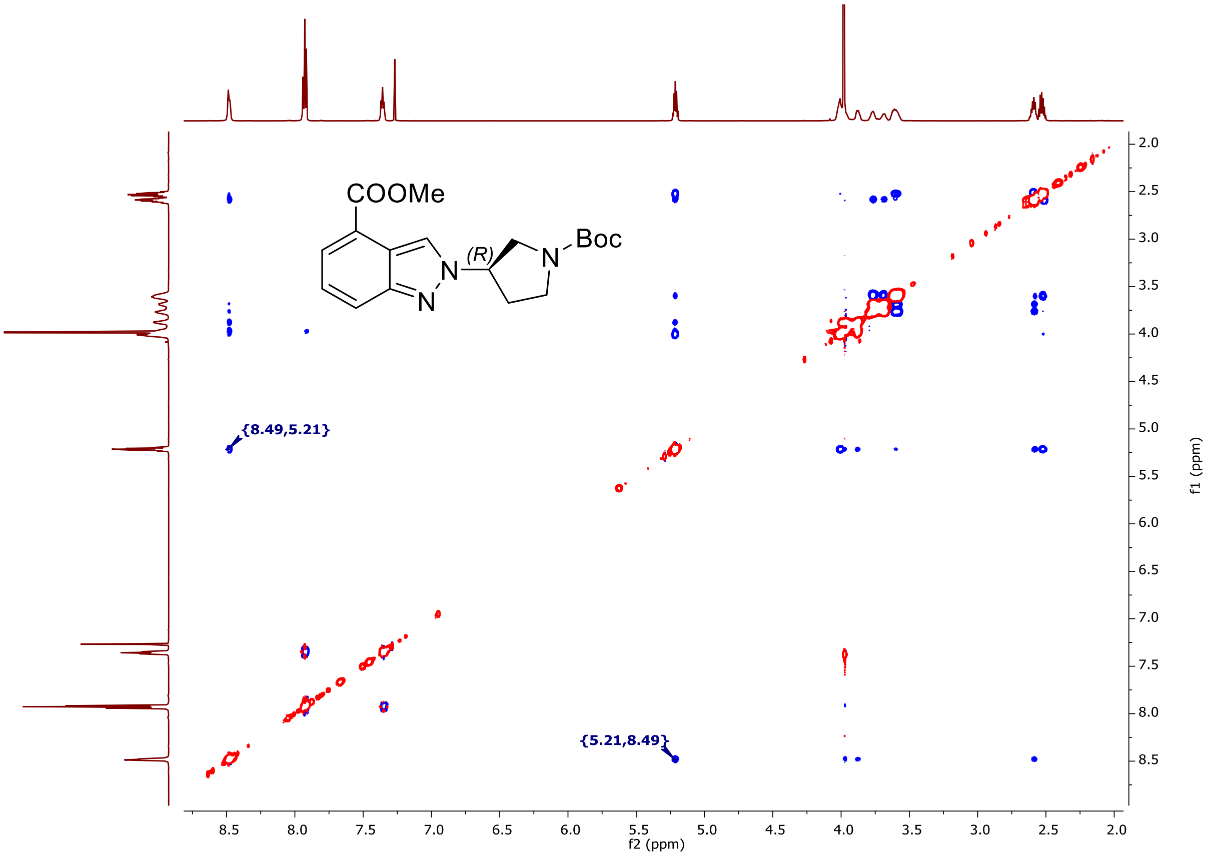

Figure S170. ^{1}H-^{1}H NOESY NMR spectrum of ***(R)*-20b** ($CDCl_3$).

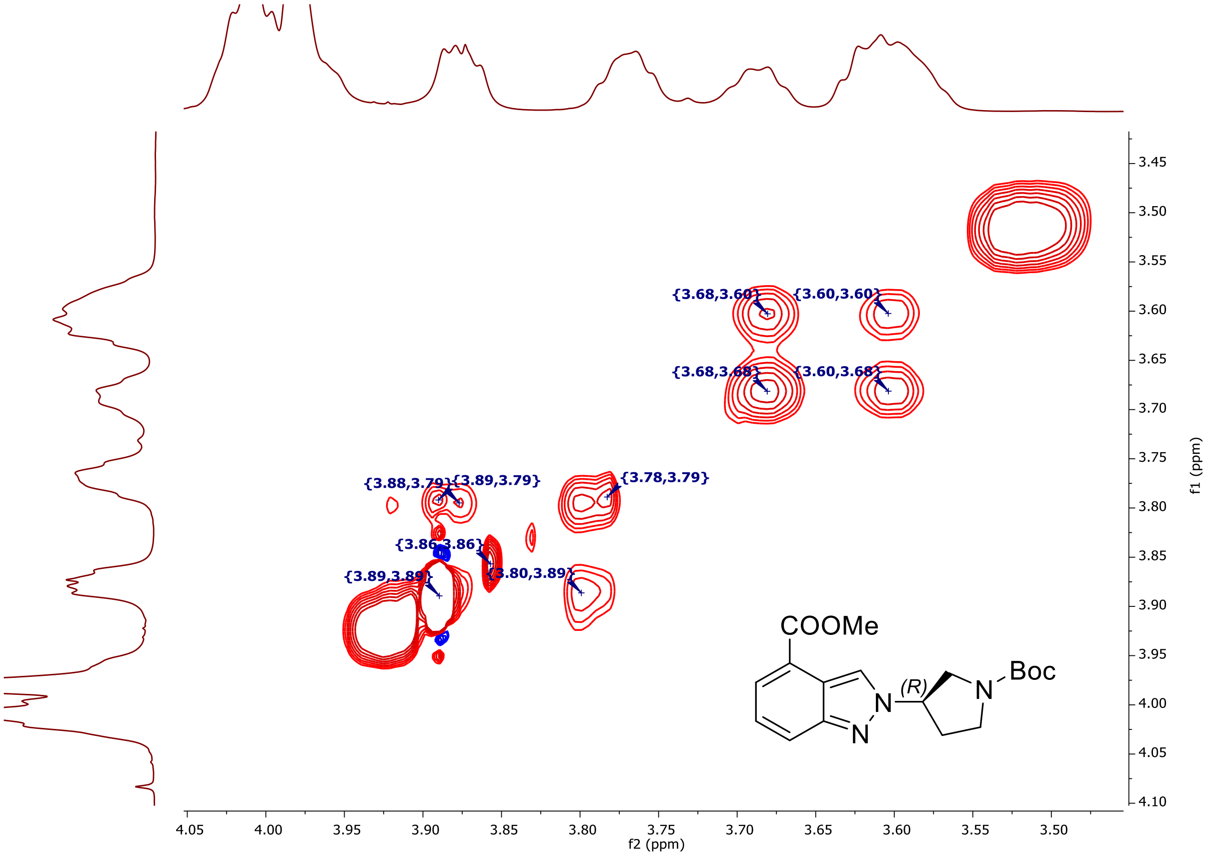

Figure S171. ^{1}H-^{1}H NOESY/EXSY NMR spectrum of ***(R)*-20b** ($CDCl_3$).

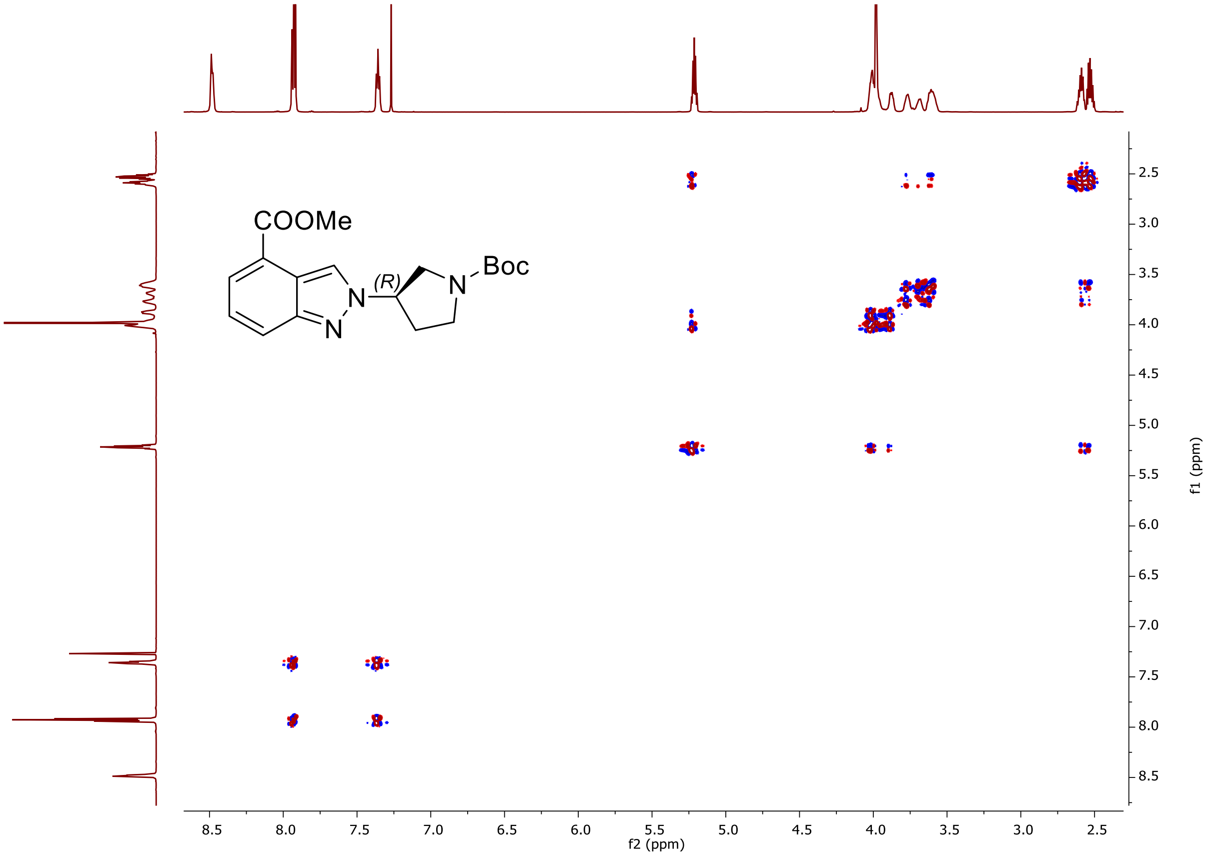

Figure S172. ^{1}H-^{1}H COSY NMR spectrum of ***(R)*-20b** ($CDCl_3$).

Acquisition Parameter

Source Type	ESI	Ion Polarity	Positive	Set Nebulizer	0.4 Bar
Focus	Not active	Set Capillary	4500 V	Set Dry Heater	180 °C
Scan Begin	50 m/z	Set End Plate Offset	-500 V	Set Dry Gas	4.0 l/min
Scan End	1000 m/z	Set Collision Cell RF	140.0 Vpp	Set Divert Valve	Waste

#	RT [min]	Area	Int. Type	I	S/N	Chromatogram	Max. m/z	FWHM [min]
n.a.	0.1	n.a.	Single spectrum	n.a.	n.a.	n.a.	304.2624	n.a.
n.a.	3.6	n.a.	Single spectrum	n.a.	n.a.	n.a.	368.1581	n.a.

+MS, 3.6min #216

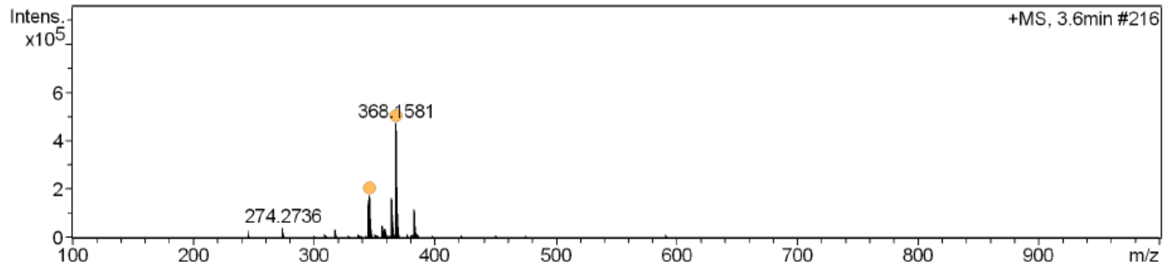

Meas. m/z	#	Ion Formula	m/z	err [ppm]	mSigma	# Sigma	Score	rdb	e⁻ Conf	N-Rule
346.1759	1	C18H24N3O4	346.1761	0.6	7.0	1	100.00	8.5	even	ok
368.1581	1	C18H23N3NaO4	368.1581	0.1	5.5	1	100.00	8.5	even	ok

Figure S173. HRMS (ESI^+) report of ***(R)*-20b**.

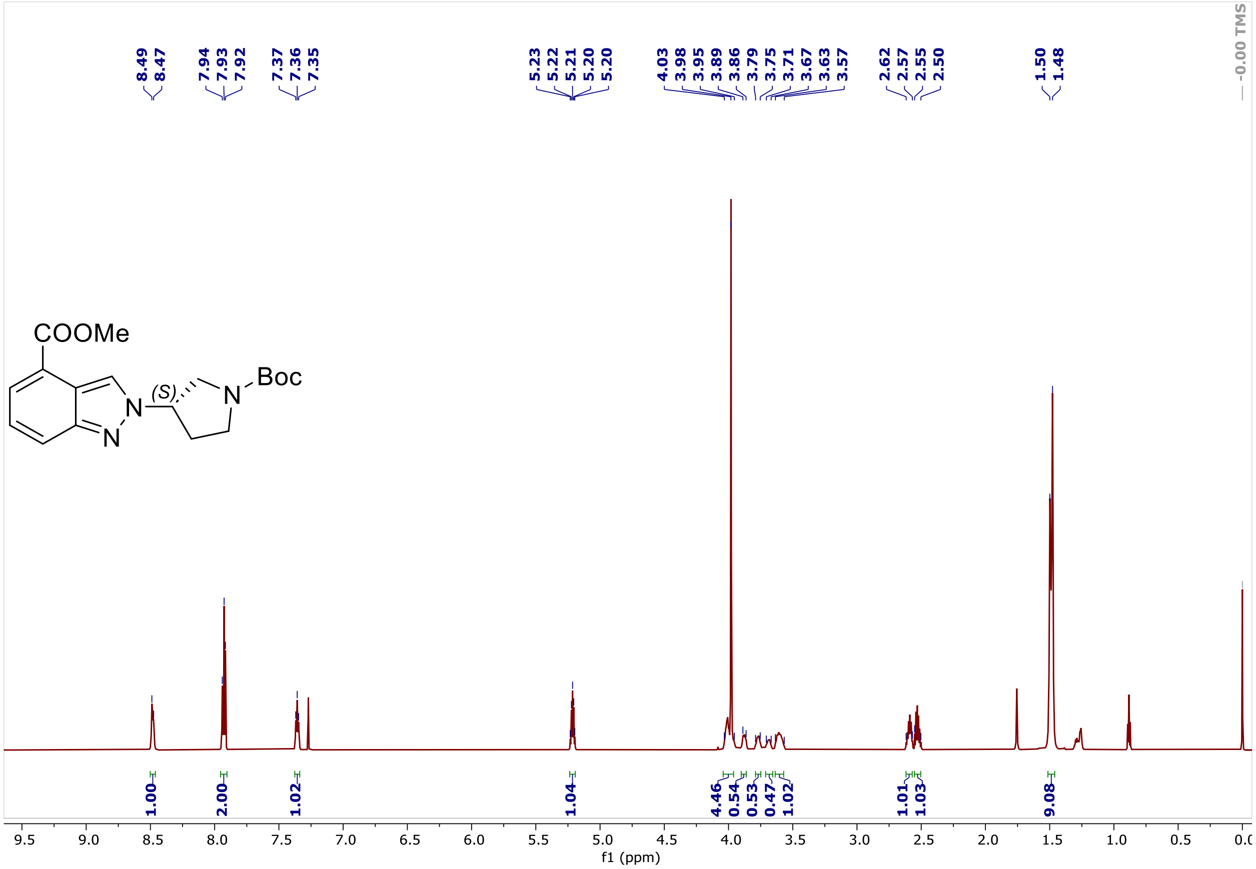

Figure S174. ^{1}H NMR (700 MHz, $CDCl_3$) spectrum of **(*S*)-20b**.

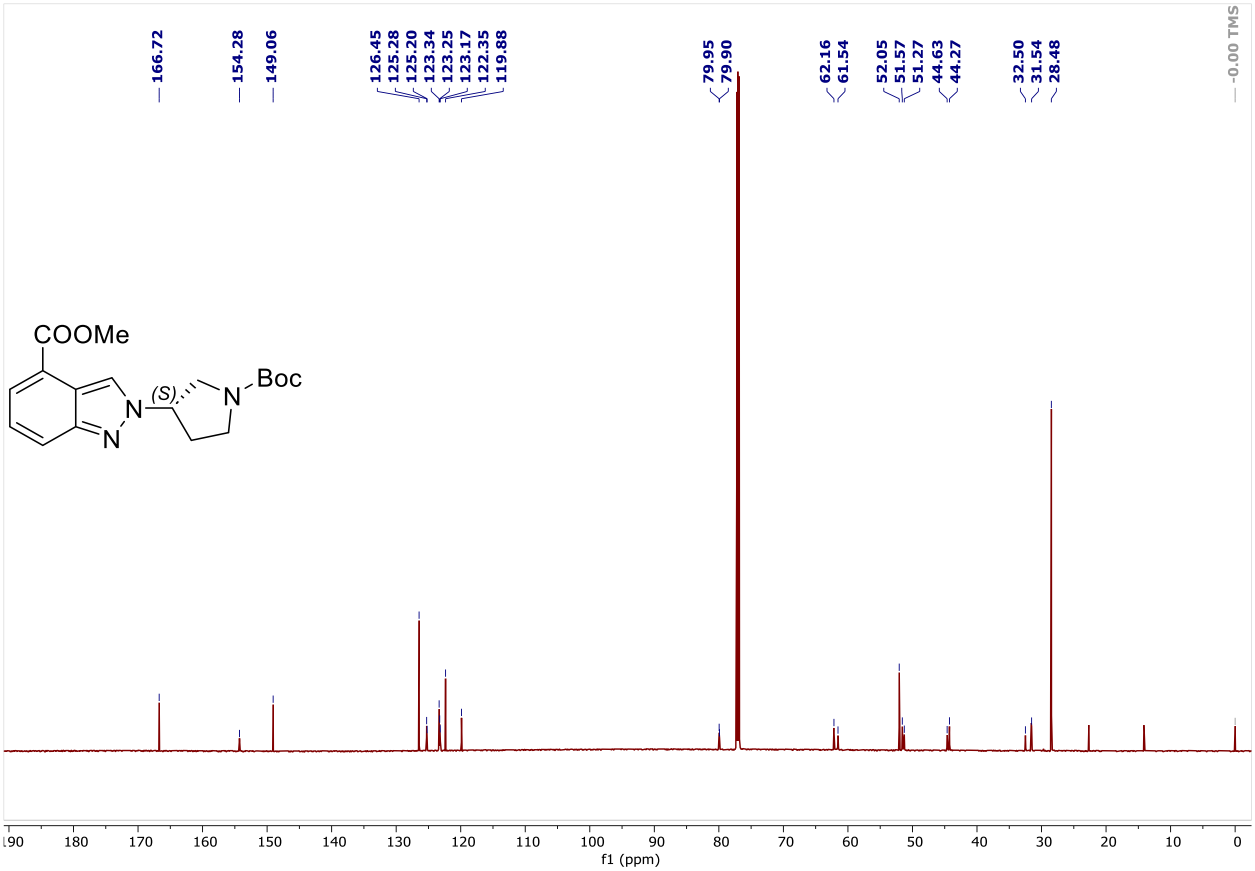

Figure S175. ^{13}C NMR (176 MHz, $CDCl_3$) spectrum of ***(S)*-20b**.

Acquisition Parameter

Source Type	ESI	Ion Polarity	Positive	Set Nebulizer	0.4 Bar
Focus	Not active	Set Capillary	4500 V	Set Dry Heater	180 °C
Scan Begin	50 m/z	Set End Plate Offset	-500 V	Set Dry Gas	4.0 l/min
Scan End	1000 m/z	Set Collision Cell RF	140.0 Vpp	Set Divert Valve	Waste

#	RT [min]	Area	Int. Type	I	S/N	Chromatogram	Max. m/z	FWHM [min]
n.a.	0.1	n.a.	Single spectrum	n.a.	n.a.	n.a.	304.2636	n.a.
n.a.	3.2	n.a.	Single spectrum	n.a.	n.a.	n.a.	368.1582	n.a.

+MS, 3.2min #189

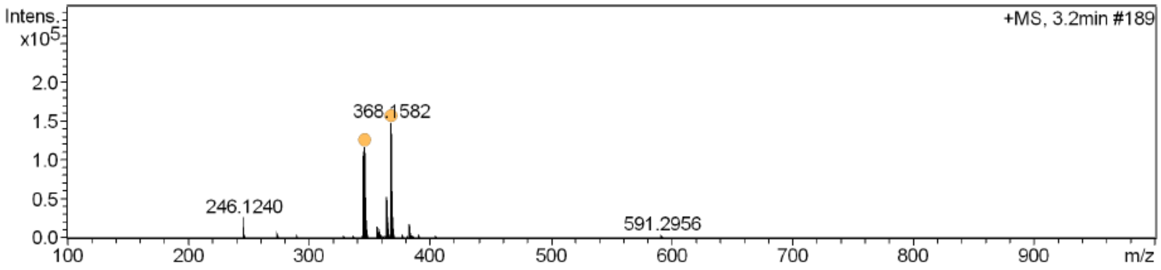

Meas. m/z	#	Ion Formula	m/z	err [ppm]	mSigma	# Sigma	Score	rdb	e¯ Conf	N-Rule
346.1764	1	C18H24N3O4	346.1761	-0.9	7.4	1	100.00	8.5	even	ok
368.1582	1	C18H23N3NaO4	368.1581	-0.4	2.2	1	100.00	8.5	even	ok

Figure S176. HRMS (ESI$^+$) report of ***(S)*-20b**.

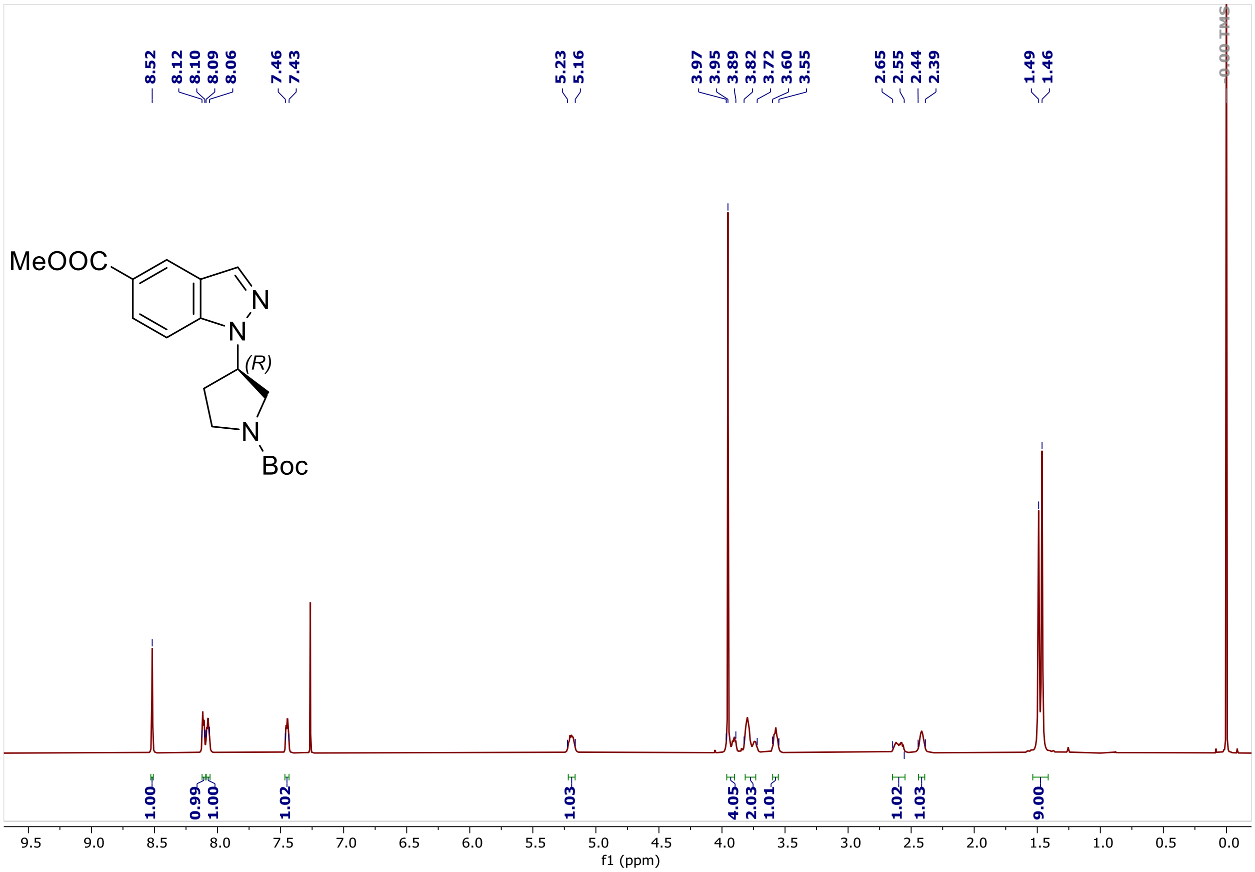

Figure S177. ^{1}H NMR (700 MHz, $CDCl_3$) spectrum of ***(R)*-19c**.

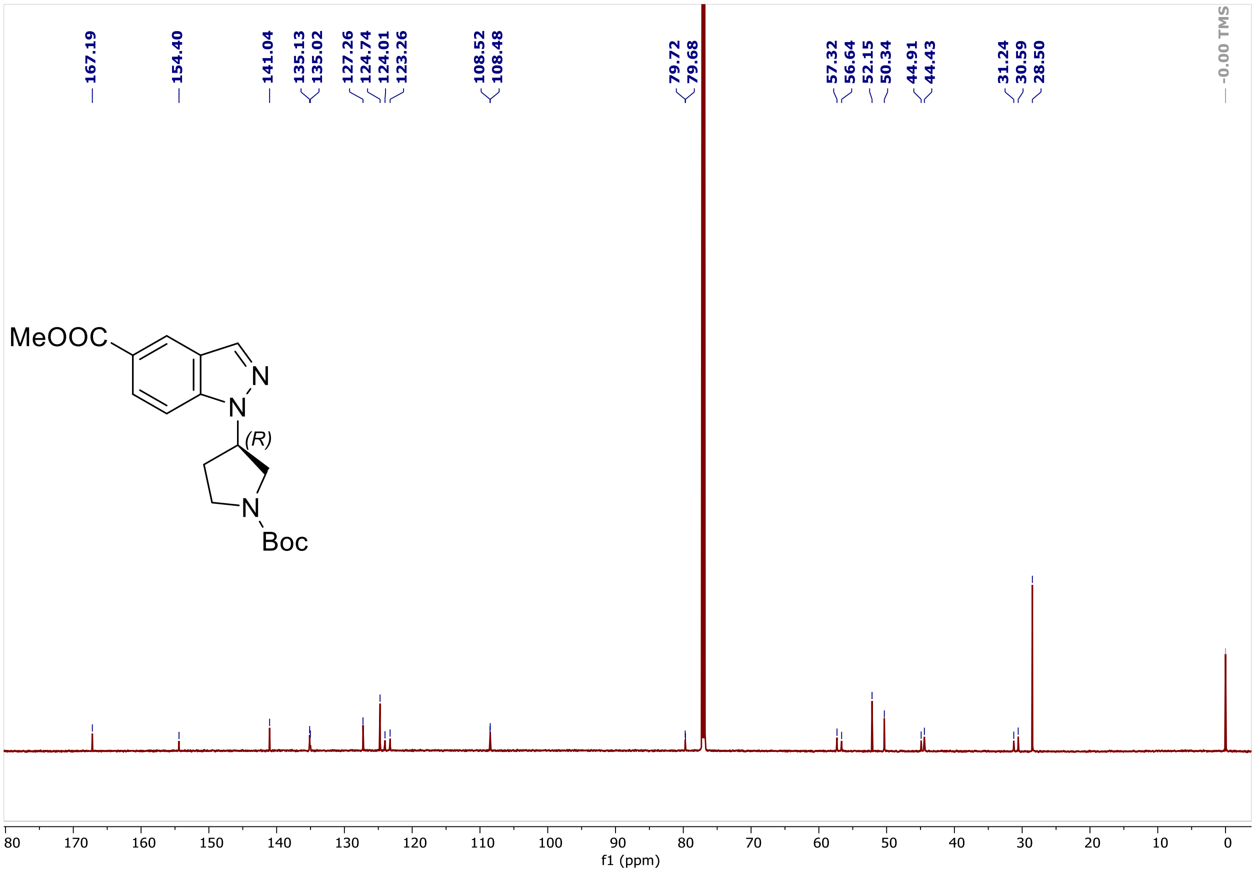

Figure S178. ^{13}C NMR (176 MHz, $CDCl_3$) spectrum of **(*R*)-19c**.

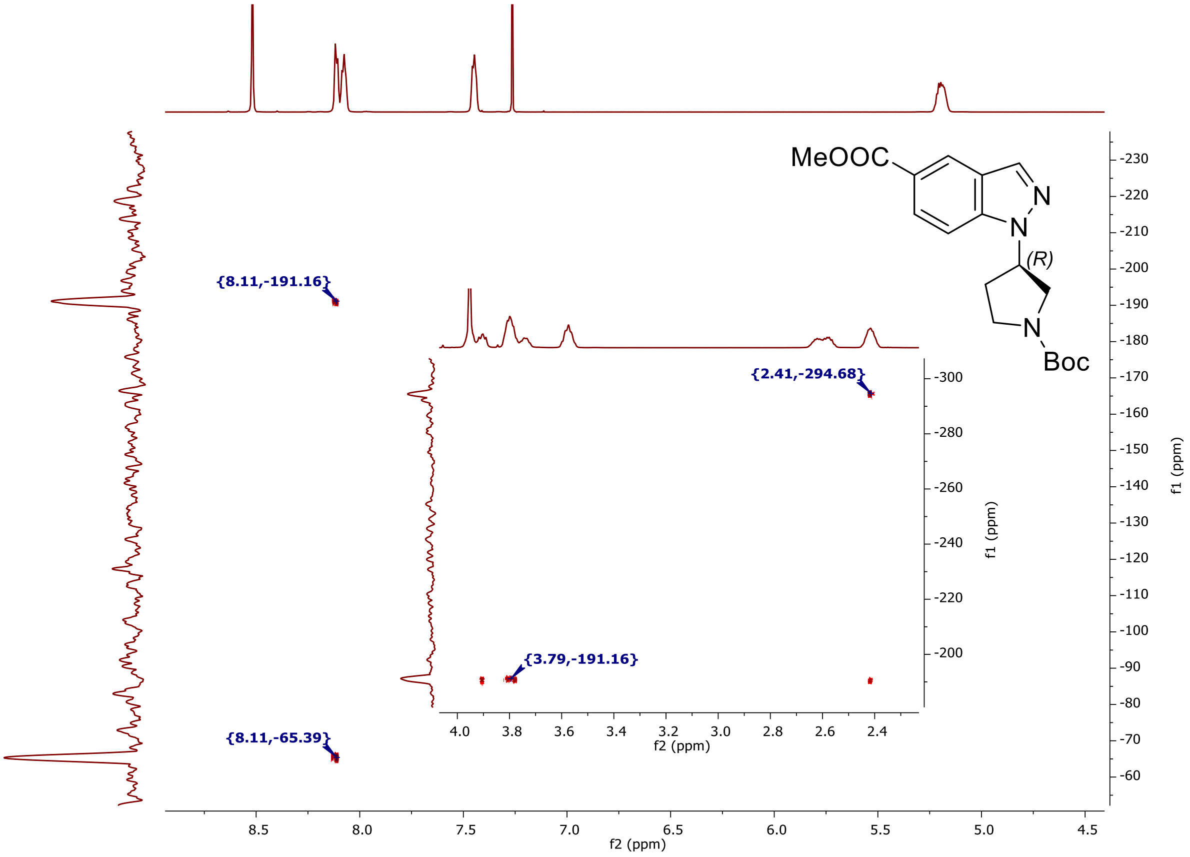

Figure S179. ^{1}H-^{15}N HMBC NMR spectrum of ***(R)*-19c** (71 MHz, $CDCl_3$).

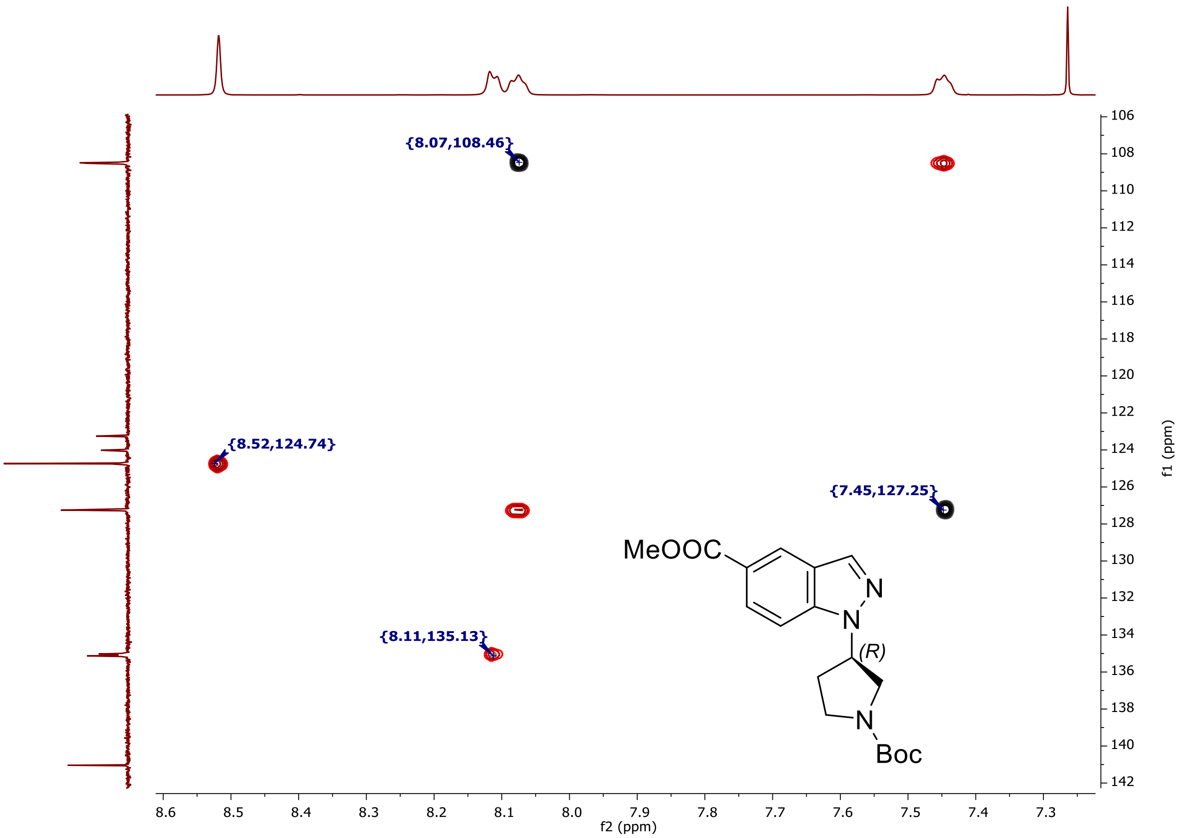

Figure S180. Overlaid multiplicity-edited ^{1}H-^{13}C HSQC (red correlations) and ^{1}H-^{13}C H2BC (black correlations) NMR spectra of ***(R)*-19c** ($CDCl_3$).

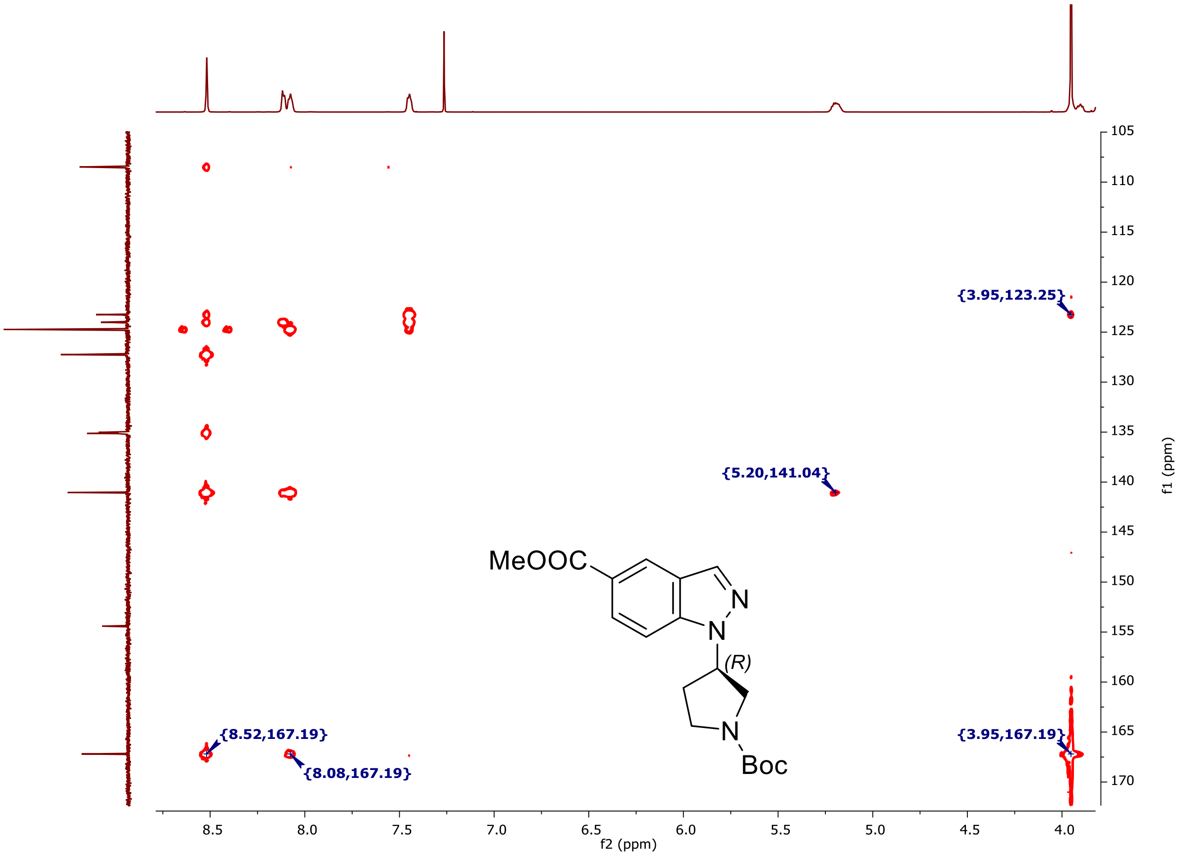

Figure S181. The 8Hz optimized ^{1}H-^{13}C HMBC NMR spectrum of ***(R)*-19c** ($CDCl_3$).

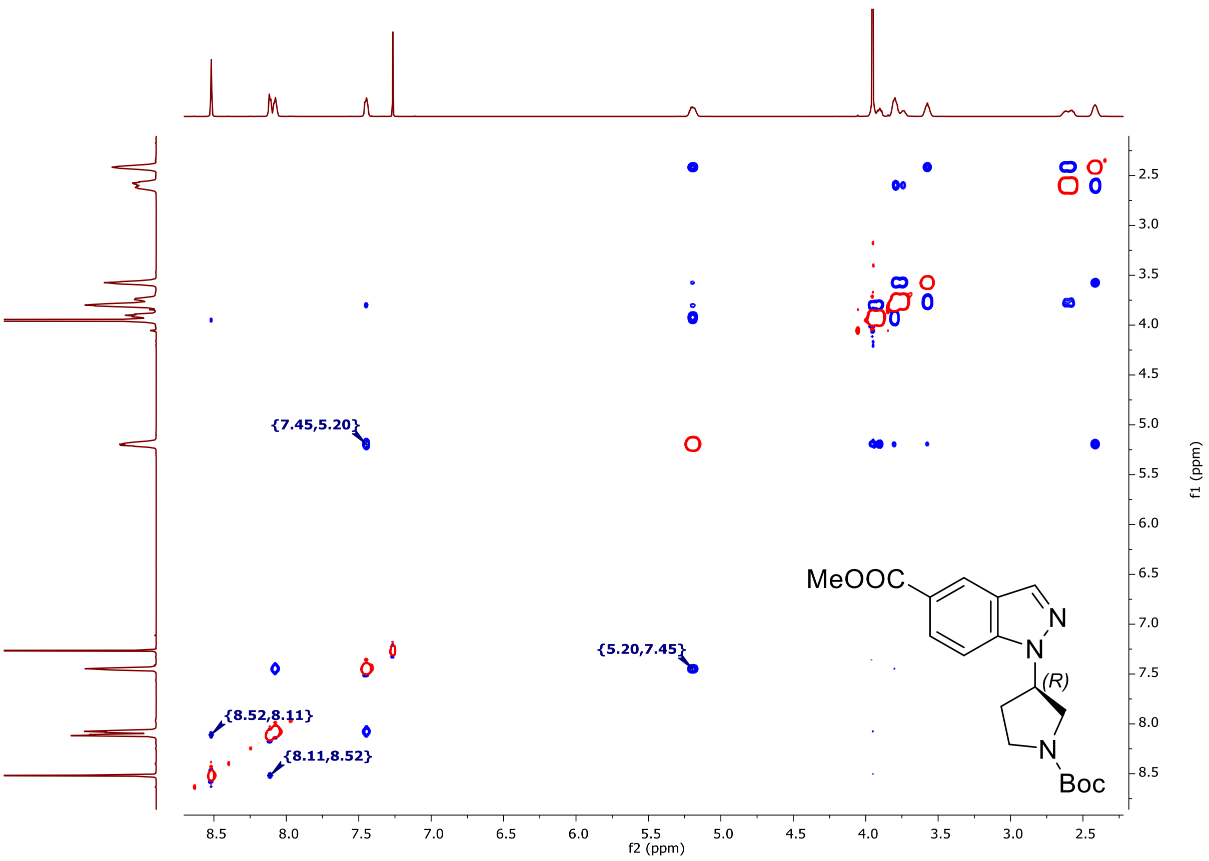

Figure S182. ^{1}H-^{1}H NOESY NMR spectrum of ***(R)*-19c** ($CDCl_3$).

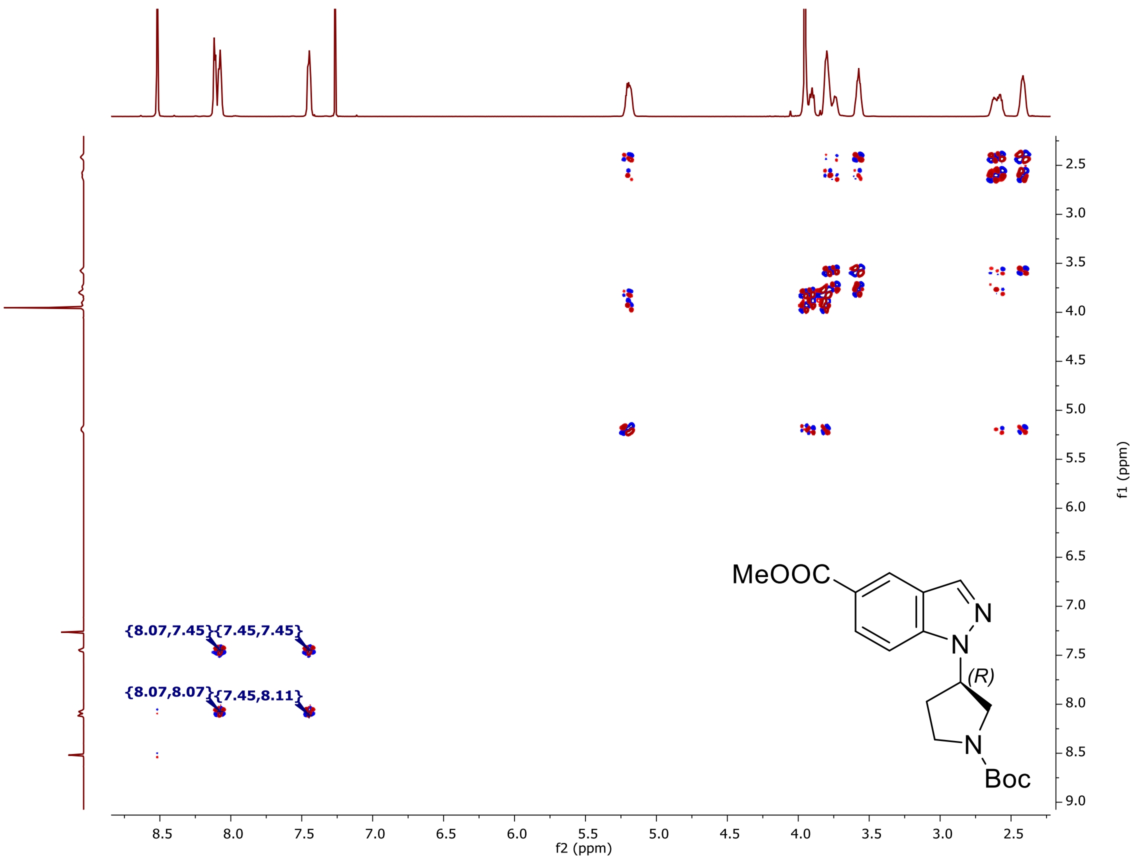

Figure S183. ^{1}H-^{1}H COSY NMR spectrum of ***(R)*-19c** ($CDCl_3$).

Acquisition Parameter

Source Type	ESI	Ion Polarity	Positive	Set Nebulizer	0.4 Bar
Focus	Not active	Set Capillary	4500 V	Set Dry Heater	180 °C
Scan Begin	50 m/z	Set End Plate Offset	-500 V	Set Dry Gas	4.0 l/min
Scan End	1000 m/z	Set Collision Cell RF	140.0 Vpp	Set Divert Valve	Waste

#	RT [min]	Area	Int. Type	I	S/N	Chromatogram	Max. m/z	FWHM [min]
n.a.	0.3	n.a.	Single spectrum	n.a.	n.a.	n.a.	304.2624	n.a.
n.a.	4.3	n.a.	Single spectrum	n.a.	n.a.	n.a.	368.1581	n.a.

+MS, 4.3min #259

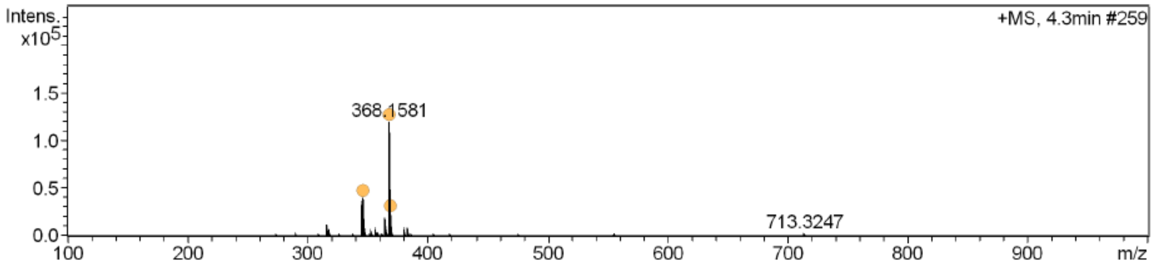

Meas. m/z	#	Ion Formula	m/z	err [ppm]	mSigma	# Sigma	Score	rdb	e⁻ Conf	N-Rule
346.1752	1	C18H24N3O4	346.1761	2.7	8.7	1	100.00	8.5	even	ok
	1	C18H24N3O4	346.1761	2.7	8.7	1	100.00	8.5	even	ok
368.1581	1	C18H23N3NaO4	368.1581	0.2	3.6	1	100.00	8.5	even	ok
369.1600	1	C18H23N3NaO4	368.1581	-3.0	3.6	1	100.00	8.5	even	ok

Figure S184. HRMS (ESI^+) report of ***(R)*-19c**.

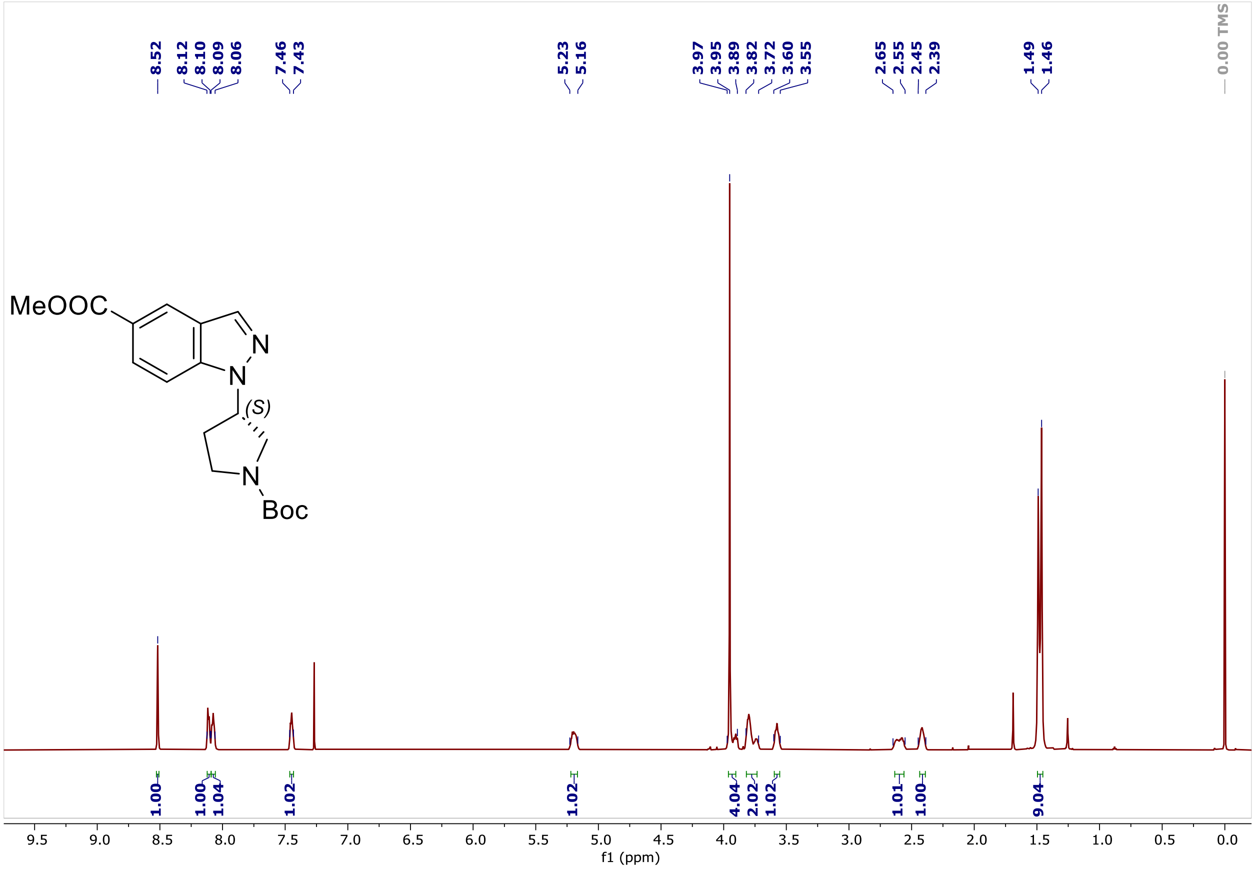

Figure S185. ^{1}H NMR (700 MHz, $CDCl_3$) spectrum of ***(S)*-19c**.

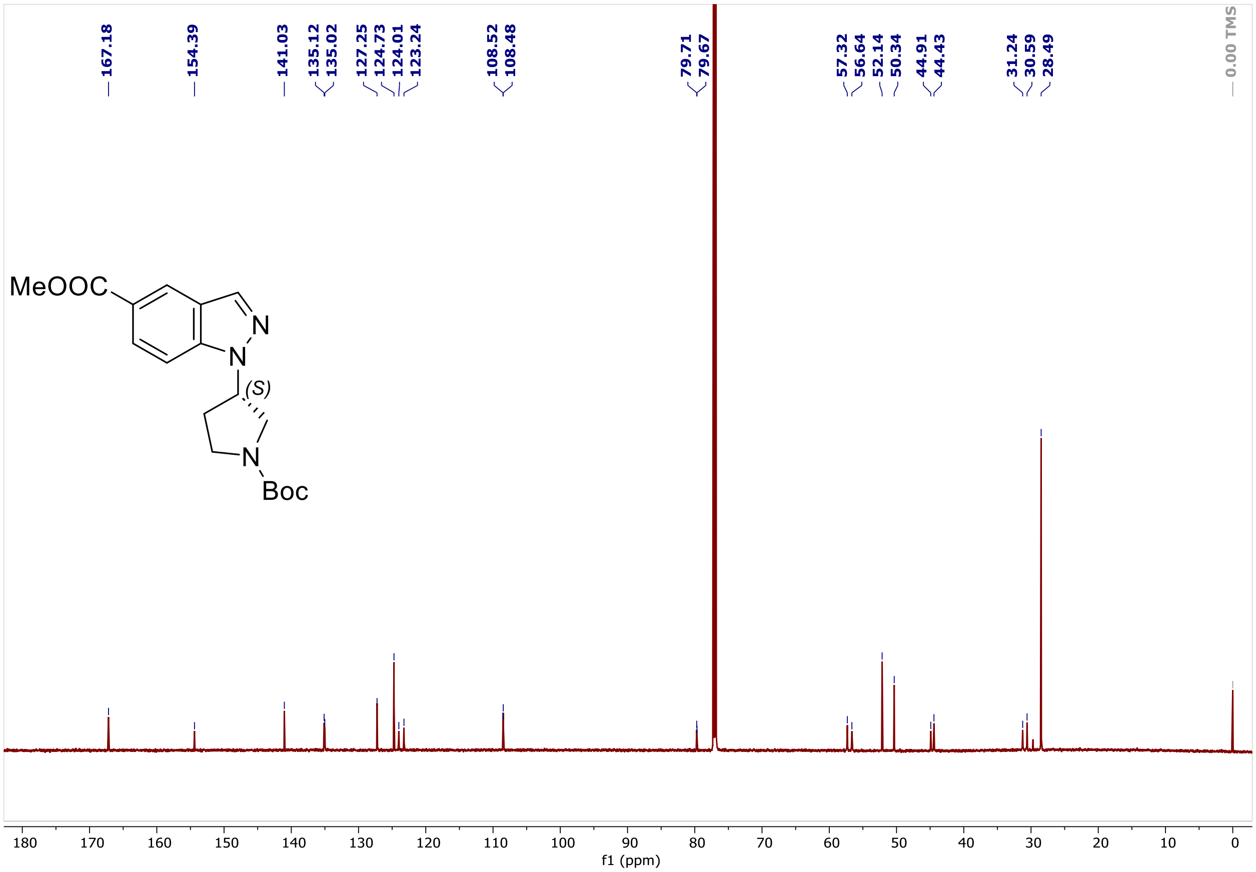

Figure S186. ^{13}C NMR (176 MHz, $CDCl_3$) spectrum of **(*S*)-19c**.

Acquisition Parameter

Source Type	ESI	Ion Polarity	Positive	Set Nebulizer	0.4 Bar
Focus	Not active	Set Capillary	4500 V	Set Dry Heater	180 °C
Scan Begin	50 m/z	Set End Plate Offset	-500 V	Set Dry Gas	4.0 l/min
Scan End	1000 m/z	Set Collision Cell RF	140.0 Vpp	Set Divert Valve	Waste

#	RT [min]	Area	Int. Type	I	S/N	Chromatogram	Max. m/z	FWHM [min]
n.a.	0.0	n.a.	Single spectrum	n.a.	n.a.	n.a.	304.2634	n.a.
n.a.	6.6	n.a.	Single spectrum	n.a.	n.a.	n.a.	368.1582	n.a.

+MS, 6.6min #397

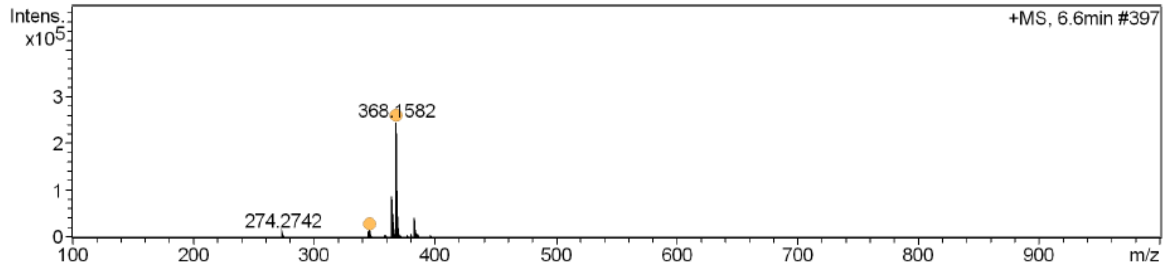

Meas. m/z	#	Ion Formula	m/z	err [ppm]	mSigma	# Sigma	Score	rdb	e⁻ Conf	N-Rule
346.1736	1	C18H24N3O4	346.1761	7.4	22.1	1	100.00	8.5	even	ok
368.1582	1	C18H23N3NaO4	368.1581	-0.4	4.4	1	100.00	8.5	even	ok

Figure S187. HRMS (ESI^+) report of ***(S)*-19c**.

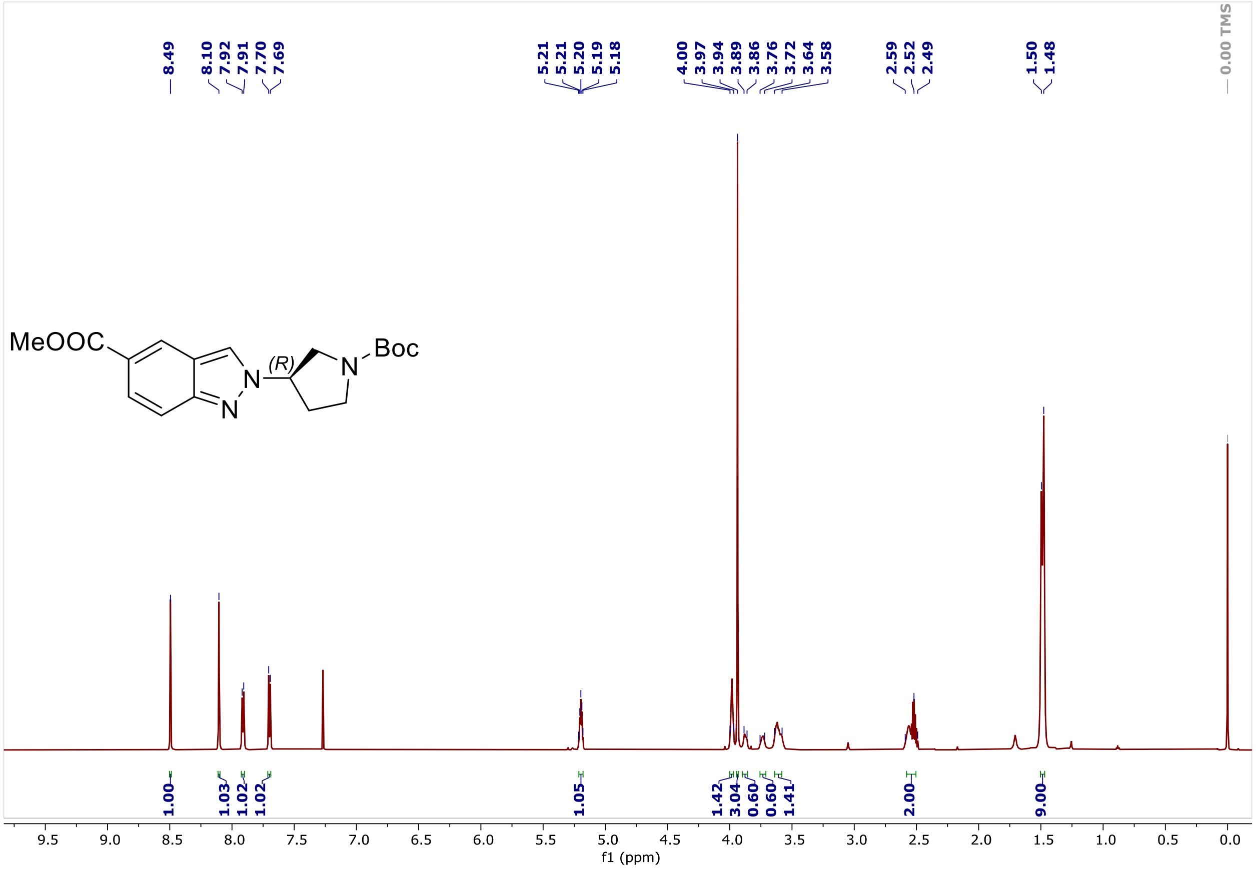

Figure S188. ^{1}H NMR (700 MHz, $CDCl_3$) spectrum of ***(R)*-20c**.

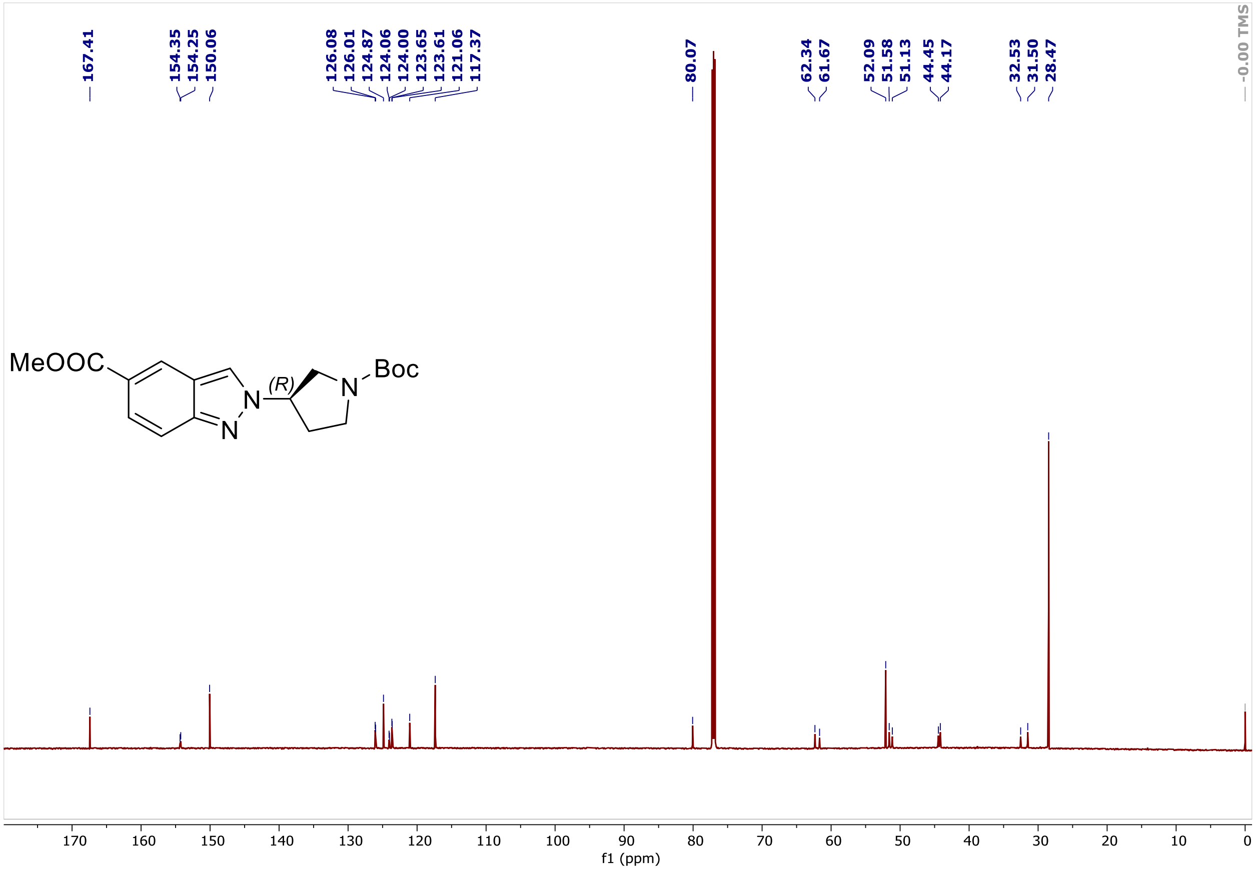

Figure S189. ^{13}C NMR (176 MHz, $CDCl_3$) spectrum of ***(R)*-20c**.

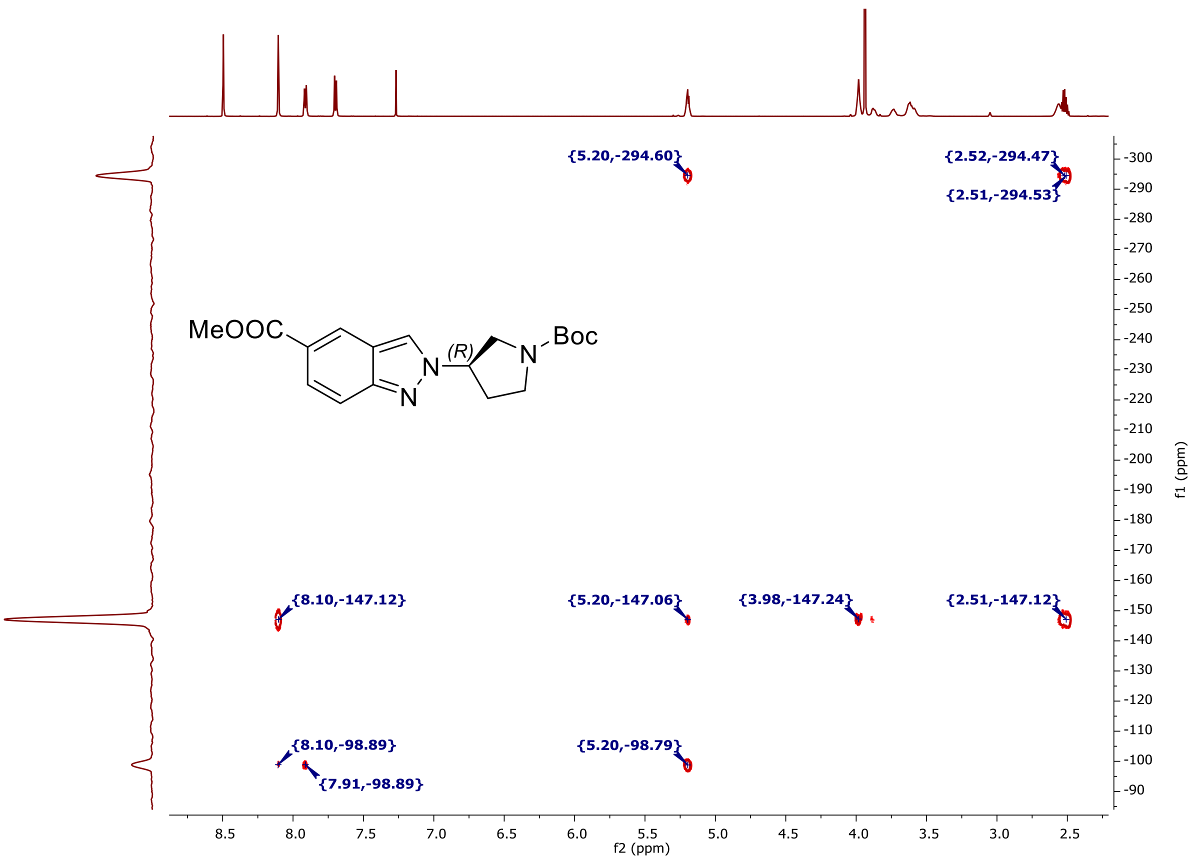

Figure S190. ^{1}H-^{15}N HMBC NMR spectrum of ***(R)*-20c** (71 MHz, $CDCl_3$).

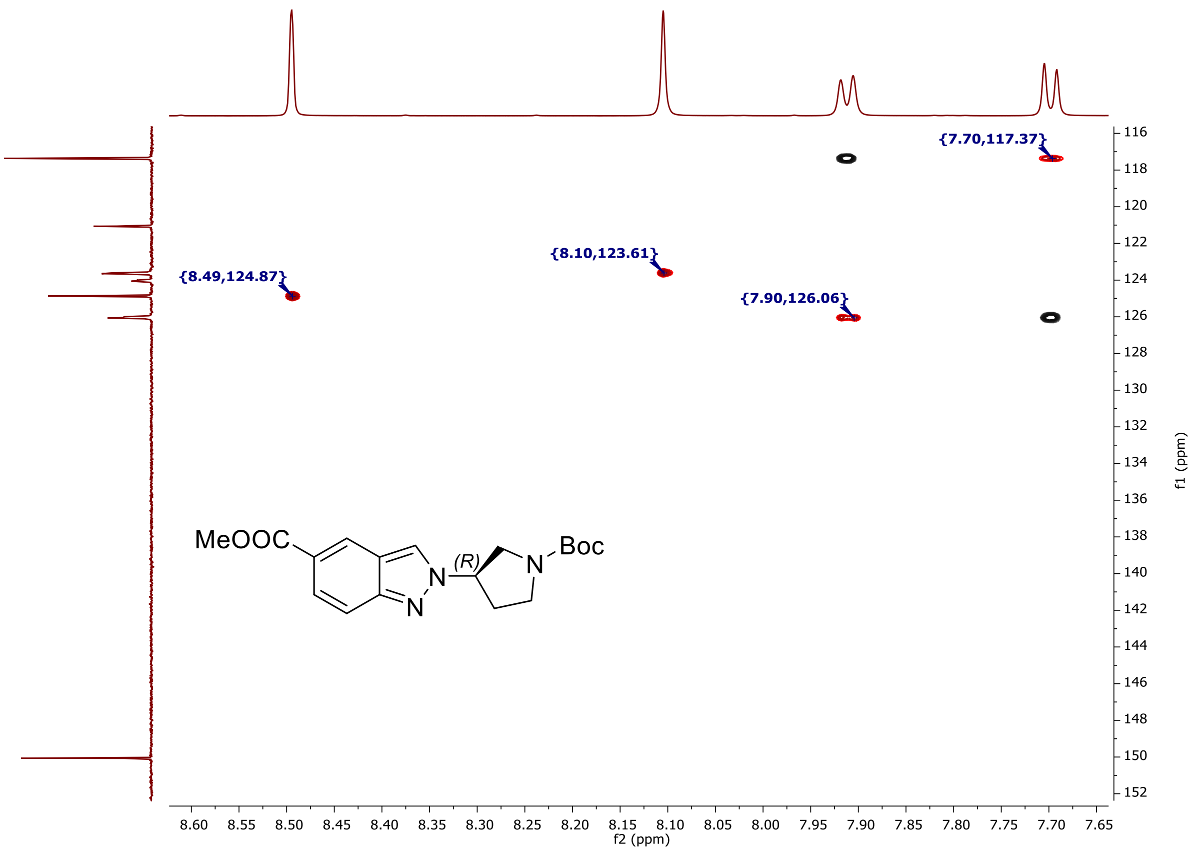

Figure S191. Overlaid multiplicity-edited ^{1}H-^{13}C HSQC (red correlations) and ^{1}H-^{13}C H2BC (black correlations) NMR spectra of ***(R)*-20c** ($CDCl_3$).

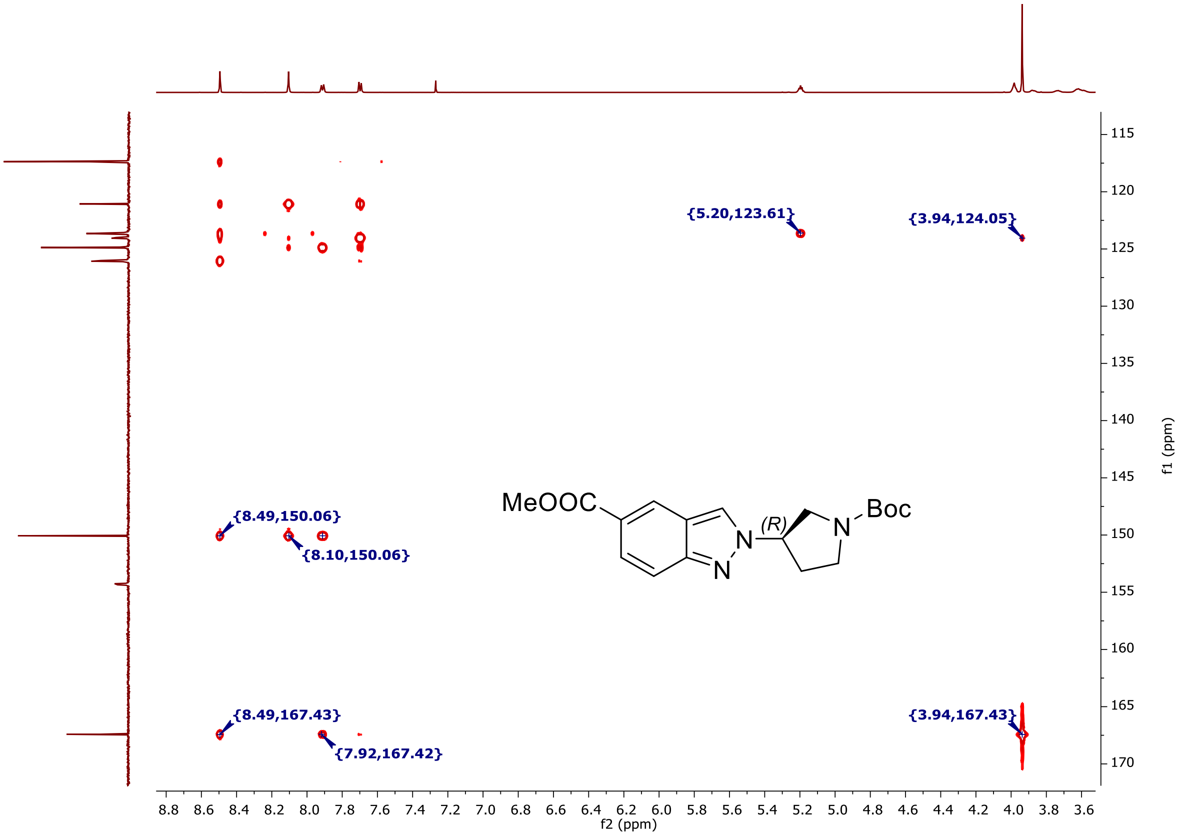

Figure S192. The 8Hz optimized ^{1}H-^{13}C HMBC NMR spectrum of ***(R)*-20c** ($CDCl_3$).

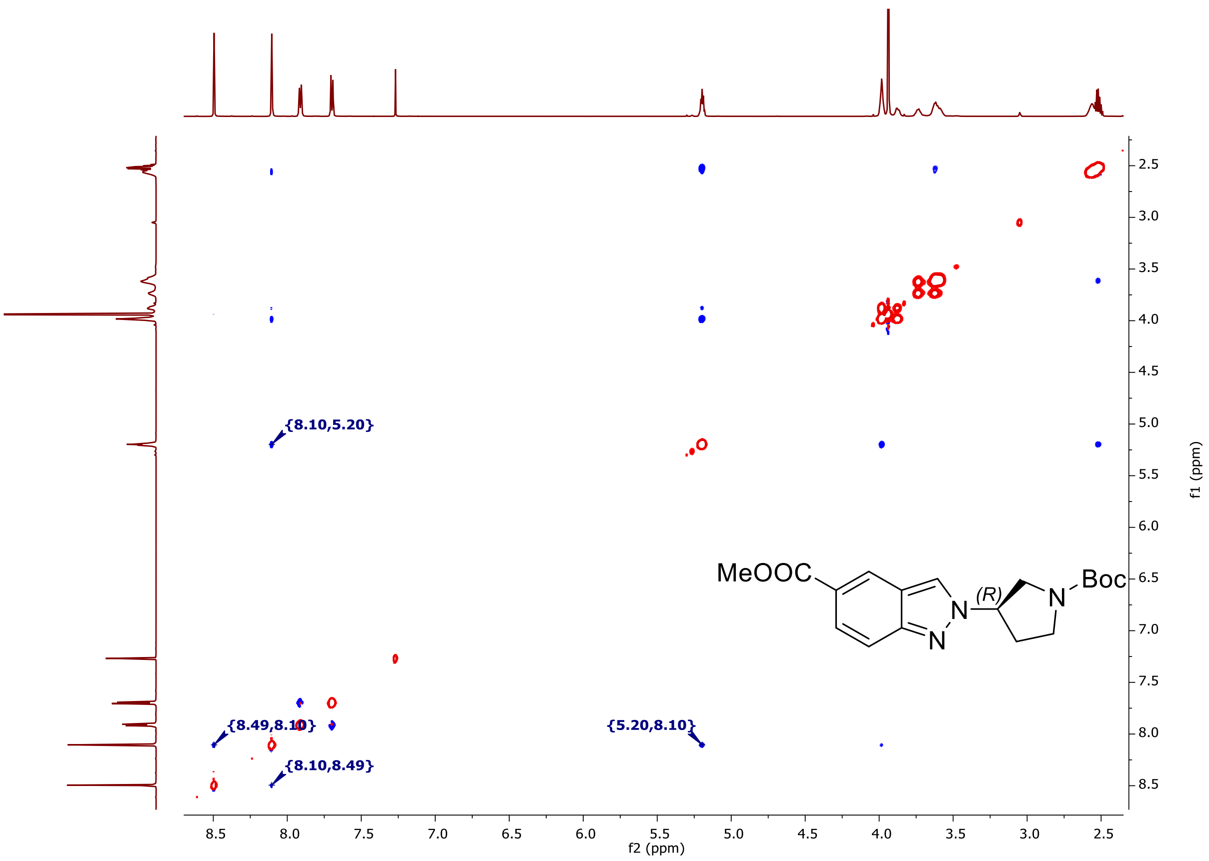

Figure S193. ^{1}H-^{1}H NOESY NMR spectrum of ***(R)*-20c** ($CDCl_3$).

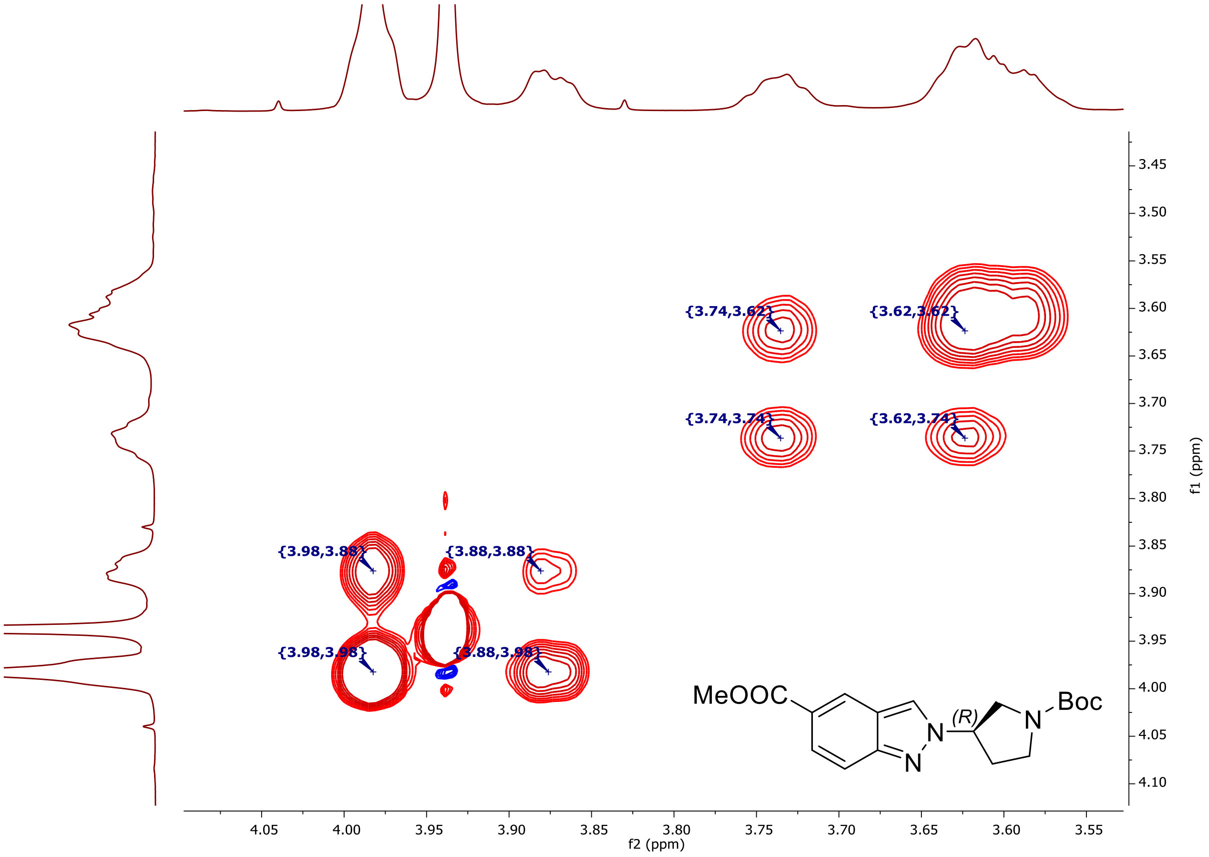

Figure S194. ^{1}H-^{1}H NOESY/EXSY NMR spectrum of **(*R*)-20c** ($CDCl_3$).

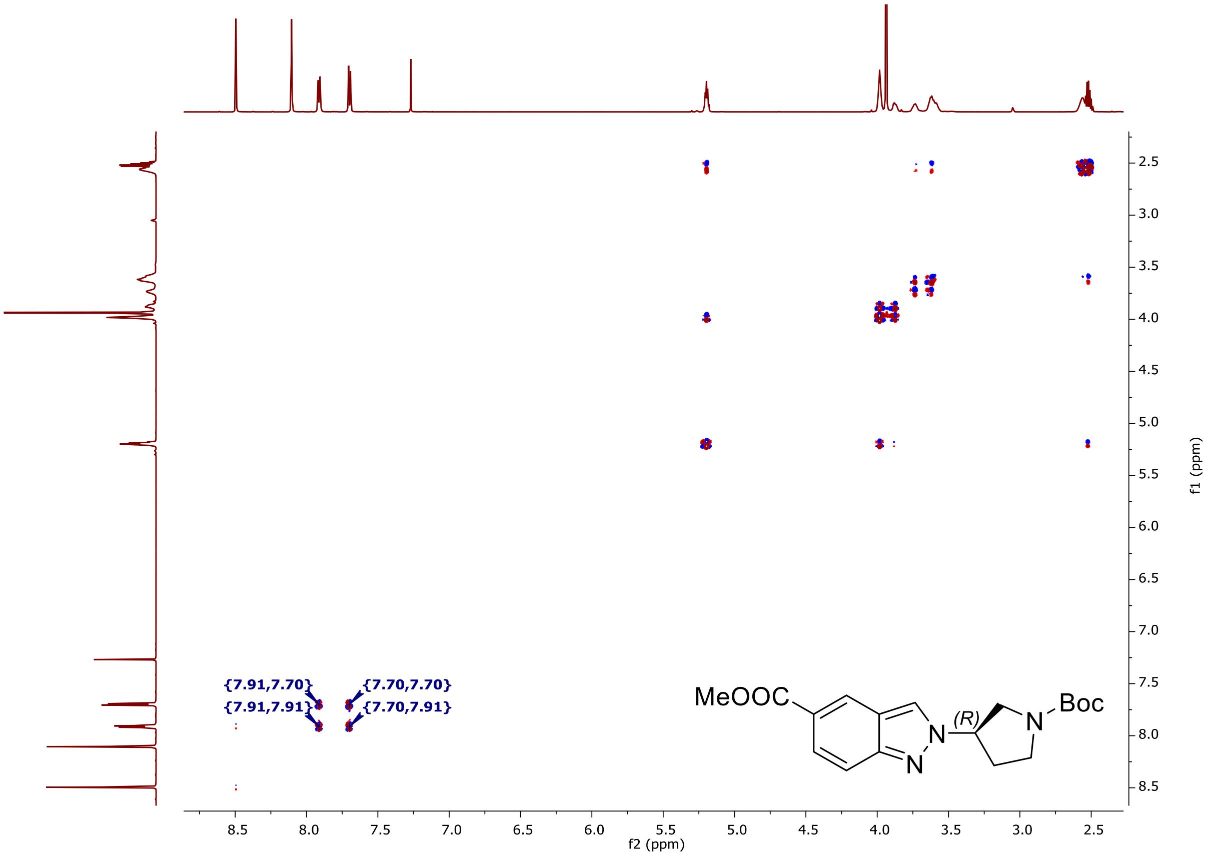

Figure S195. ^{1}H-^{1}H COSY NMR spectrum of **(*R*)-20c** ($CDCl_3$).

Acquisition Parameter

Source Type	ESI	Ion Polarity	Positive	Set Nebulizer	0.4 Bar		
Focus	Not active	Set Capillary	4500 V	Set Dry Heater	180 °C		
Scan Begin	50 m/z	Set End Plate Offset	-500 V	Set Dry Gas	4.0 l/min		
Scan End	1000 m/z	Set Collision Cell RF	140.0 Vpp	Set Divert Valve	Waste		

#	RT [min]	Area	Int. Type	I	S/N	Chromatogram	Max. m/z	FWHM [min]
n.a.	2.8	n.a.	Single spectrum	n.a.	n.a.	n.a.	304.2625	n.a.
n.a.	6.3	n.a.	Single spectrum	n.a.	n.a.	n.a.	368.1581	n.a.

+MS, 6.3min #379

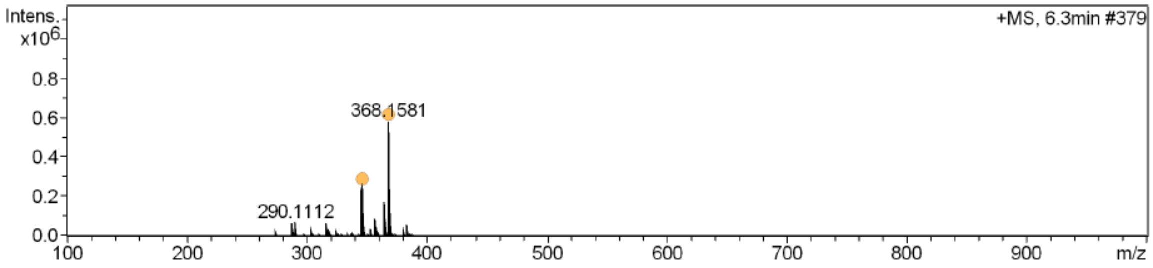

Meas. m/z	#	Ion Formula	m/z	err [ppm]	mSigma	# Sigma	Score	rdb	e⁻ Conf	N-Rule
346.1729	1	C18H24N3O4	346.1761	9.2	2.6	1	100.00	8.5	even	ok
368.1581	1	C18H23N3NaO4	368.1581	0.2	8.5	1	100.00	8.5	even	ok

Figure S196. HRMS (ESI$^+$) report of **(*R*)-20c**.

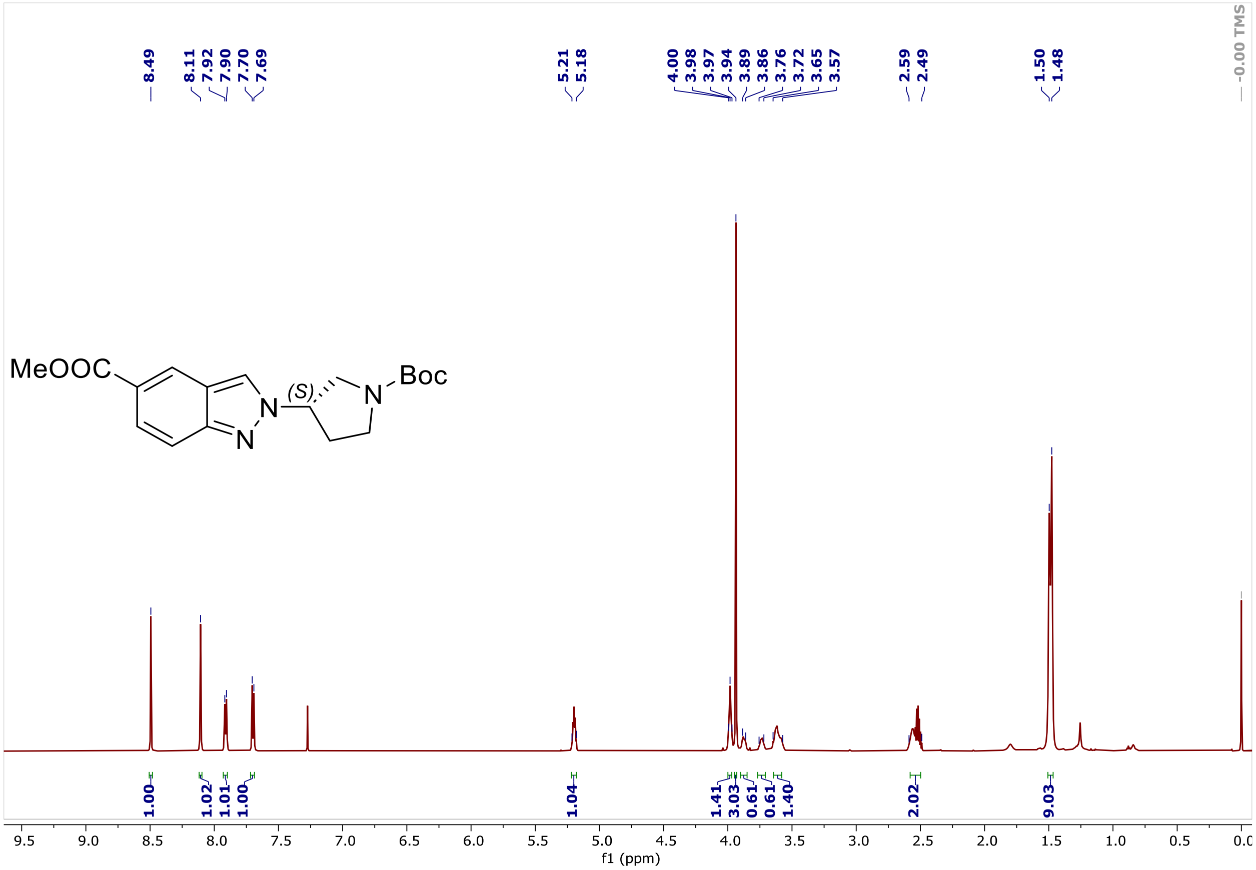

Figure S197. ^{1}H NMR (700 MHz, $CDCl_3$) spectrum of **(*S*)-20c**.

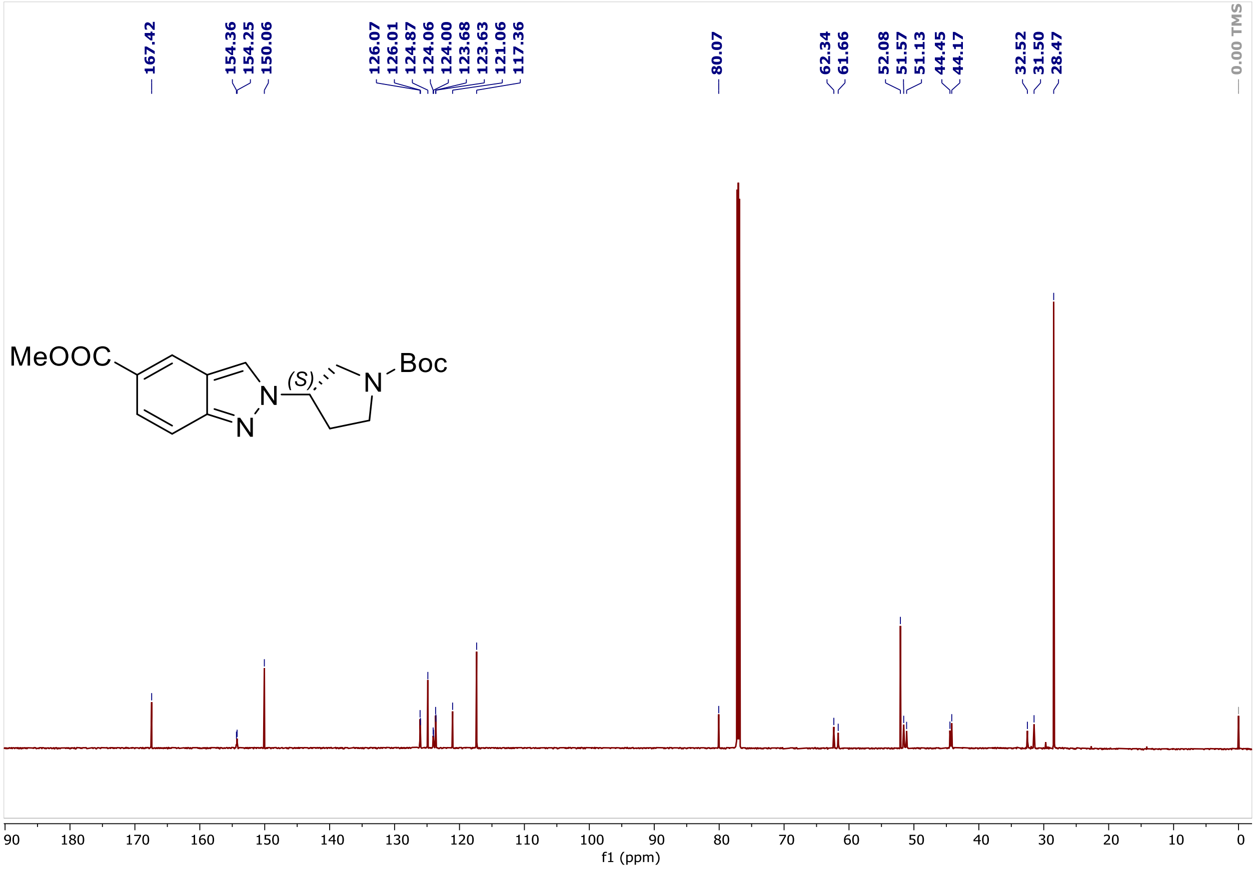

Figure S198. ^{13}C NMR (176 MHz, $CDCl_3$) spectrum of ***(S)*-20c**.

Acquisition Parameter

Source Type	ESI	Ion Polarity	Positive	Set Nebulizer	0.4 Bar
Focus	Not active	Set Capillary	4500 V	Set Dry Heater	180 °C
Scan Begin	50 m/z	Set End Plate Offset	-500 V	Set Dry Gas	4.0 l/min
Scan End	1000 m/z	Set Collision Cell RF	140.0 Vpp	Set Divert Valve	Waste

#	RT [min]	Area	Int. Type	I	S/N	Chromatogram	Max. m/z	FWHM [min]
n.a.	0.1	n.a.	Single spectrum	n.a.	n.a.	n.a.	304.2644	n.a.
n.a.	3.9	n.a.	Single spectrum	n.a.	n.a.	n.a.	368.1586	n.a.

+MS, 3.9min #232

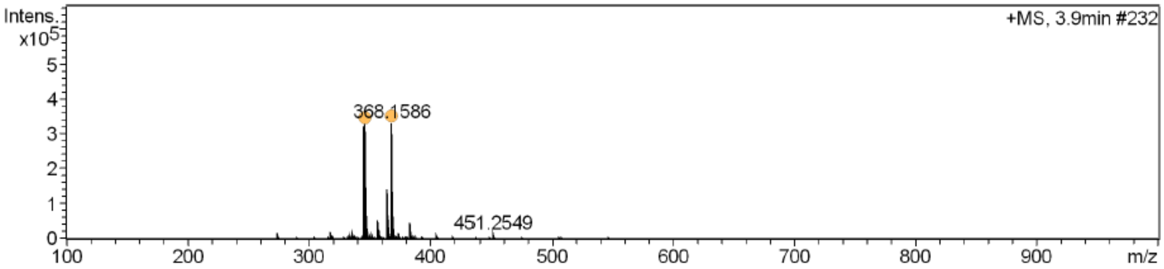

Meas. m/z	#	Ion Formula	m/z	err [ppm]	mSigma	# Sigma	Score	rdb	e⁻ Conf	N-Rule
346.1755	1	C18H24N3O4	346.1761	1.7	5.4	1	100.00	8.5	even	ok
368.1586	1	C18H23N3NaO4	368.1581	-1.3	3.3	1	100.00	8.5	even	ok

Figure S199. HRMS (ESI$^+$) report of ***(S)*-20c**.

X-ray analysis of compound (*R*)-5b:

Single crystals of $C_{19}H_{24}N_2O_4$ **(*R*)-5b** were investigated on a Rigaku XtaLAB Synergy Dualflex HyPix diffractometer. The crystal was maintained at 140.0(3) K during data collection. Using Olex2 [1], the structure was solved with the Superflip [2] structure-solution program using Charge Flipping and refined with the ShelXL [3] using Least Squares minimization.

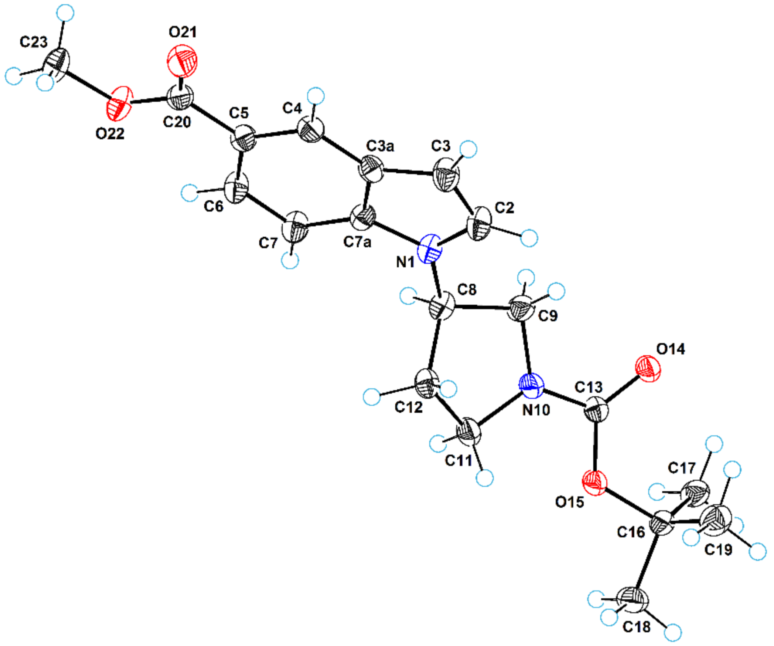

Figure S200. ORTEP view of the asymmetric unit of the crystal **(*R*)-5b**.

Crystal Data for $C_{19}H_{24}N_2O_4$ (M = 344.40 g/mol): monoclinic, space group $P2_1$ (no. 4), a = 9.9112(2) Å, b = 7.32502(6) Å, c = 12.4029(1) Å, β = 99.8921(7)°, V = 887.06(1) Å^3, Z = 2, T = 140.0(3) K, μ(CuKα) = 0.741 mm^{-1}, $Dcalc$ = 1.289 g/cm^3, 19973 reflections measured ($2\Theta \leq 160°$), 3659 unique (R_{int} = 0.0210, R_{sigma} = 0.0162) which were used in all calculations. The final R_1 was 0.0249 ($I > 2\sigma(I)$) and wR_2 was 0.0670 (all data).

Table S1. Crystal data and structure refinement for **(*R*)-5b**.

**Empirical formula**	$C_{19}H_{24}N_2O_4$	**μ/mm^{-1}**	0.741
**Formula weight**	344.40	***F*(000)**	368.0
**Temperature/K**	140.0(3)	**Crystal size/mm^3**	0.22 × 0.13 × 0.07
**Crystal system**	monoclinic	**Radiation**	CuKα (λ = 1.54184 Å)
**Space group**	$P2_1$	**2Θ max. for data collection**	160
***a*/Å**	9.91118(7)	**Index ranges**	$-12 \leq h \leq 12$, $-9 \leq k \leq 7$, $-15 \leq l \leq 15$
***b*/Å**	7.32502(6)	**Reflections collected**	19973
***c*/Å**	12.40294(9)	**Independent reflections**	3659 [R_{int} = 0.0210, R_{sigma} = 0.0162]
**α/°**	90	**Data/restraints/parameters**	3659/1/243
**β/°**	99.8921(7)	**Goodness-of-fit on F^2**	1.045
**γ/°**	90	**Final *R* indexes [*I* > 2σ(*I*)]**	R_1 = 0.0249, wR_2 = 0.0668
**Volume/Å^3**	887.062(12)	**Final *R* indexes [all data]**	R_1 = 0.0252, wR_2 = 0.0670
Z	2	**Largest diff. peak/hole / e Å^{-3}**	0.17/-0.13
**ρ_{calc}mg/mm^3**	1.289	**Flack′s *x* parameter**	-0.04(7)

Table S2. Fractional Atomic Coordinates (×10^4) and Equivalent Isotropic Displacement Parameters (Å^2×10^3) for **(*R*)-5b**. U_{eq} is defined as 1/3 of of the trace of the orthogonalised U_{IJ} tensor.

**Atom**	***x***	***y***	***z***	***U*(eq)**
N1	8209.9(12)	3894.4(18)	4248.0(10)	24.4(3)
C2	9351.5(15)	2828(2)	4198.2(13)	30.5(3)
C3	10133.2(15)	3613(3)	3527.0(13)	31.6(3)
C3A	9473.0(13)	5279(2)	3135.4(11)	23.8(3)

C4	9774.8(14)	6667(2)	2441.9(11)	24.7(3)
C5	8917.8(14)	8168(2)	2253.5(11)	24.0(3)
C6	7739.8(15)	8294(2)	2755.4(13)	27.9(3)
C7	7414.8(15)	6939(2)	3440.1(13)	28.1(3)
C7A	8278.9(14)	5428(2)	3617.3(11)	22.6(3)
C8	7173.9(14)	3609(2)	4941.7(12)	24.8(3)
C9	6888.9(16)	1590(2)	5145.5(12)	28.2(3)
N10	6529.2(13)	1609.6(19)	6236.8(10)	25.9(3)
C11	6642.9(16)	3425(2)	6748.0(13)	28.0(3)
C12	7550.6(16)	4452(2)	6079.6(13)	26.7(3)
C13	6241.3(13)	44(2)	6709.0(11)	21.2(3)
O14	6238.9(11)	-1443.0(16)	6262.6(8)	25.7(2)
O15	5940.1(11)	355.5(15)	7713.2(8)	26.0(2)
C16	5742.4(15)	-1171(2)	8446.8(11)	24.2(3)
C17	4588.2(16)	-2418(2)	7929.3(12)	28.5(3)
C18	5338.3(18)	-164(3)	9414.8(13)	33.5(4)
C19	7088.7(17)	-2179(3)	8768.7(13)	35.4(4)
C20	9309.1(15)	9646(2)	1547.1(12)	25.3(3)
O21	10381.2(12)	9685.7(18)	1207.6(10)	35.8(3)
O22	8349.9(11)	10940.7(17)	1324.6(10)	32.2(3)
C23	8688.9(18)	12431(3)	658.0(15)	36.5(4)

Table S3. Anisotropic Displacement Parameters ($Å^2×10^3$) for ***(R)*-5b**. The Anisotropic displacement factor exponent takes the form: $-2\pi^2[h^2a^{*2}U_{11}+2hka^*b^*U_{12}+…]$.

Atom	U_{11}	U_{22}	U_{33}	U_{23}	U_{13}	U_{12}
N1	22.0(5)	22.9(7)	27.8(6)	5.2(5)	3.2(4)	0.5(5)
C2	27.3(7)	25.9(8)	37.7(8)	8.4(7)	4.0(6)	4.8(6)
C3	26.7(7)	27.7(9)	41.4(8)	4.9(7)	8.7(6)	6.1(7)
C3A	22.1(6)	22.4(8)	26.5(6)	-2.2(6)	3.0(5)	-0.2(5)
C4	24.3(6)	24.1(8)	26.7(7)	-1.4(6)	7.7(5)	-2.2(6)
C5	24.4(6)	23.0(8)	24.4(6)	-0.2(5)	3.6(5)	-3.5(5)
C6	25.0(7)	23.1(9)	36.2(7)	6.5(6)	6.9(5)	2.7(6)
C7	23.2(6)	27.4(9)	35.4(7)	5.8(6)	9.3(6)	1.1(6)
C7A	21.8(6)	22.2(8)	23.0(6)	1.6(6)	2.0(5)	-3.2(5)
C8	21.9(6)	24.7(8)	27.4(6)	5.0(6)	3.4(5)	-0.4(6)
C9	36.4(7)	25.4(8)	23.2(6)	0.4(6)	6.1(6)	-7.0(7)
N10	35.0(6)	19.3(7)	24.7(6)	-1.5(5)	8.9(5)	-3.4(5)
C11	35.4(7)	18.6(8)	31.5(7)	-1.9(6)	10.1(6)	-1.1(6)
C12	29.3(7)	19.4(8)	31.4(7)	0.4(6)	5.6(6)	-2.5(5)
C13	19.4(6)	20.3(7)	24.3(6)	-0.2(5)	4.9(5)	0.1(5)
O14	31.8(5)	19.5(5)	27.7(5)	-1.4(4)	10.7(4)	-1.6(4)
O15	34.6(5)	19.0(6)	27.0(5)	-0.7(4)	12.9(4)	-1.8(4)
C16	30.0(7)	21.5(8)	22.0(6)	0.9(5)	7.1(5)	-2.5(6)
C17	33.9(7)	27.7(9)	24.5(7)	1.5(6)	7.0(5)	-7.5(6)
C18	44.4(9)	31.2(10)	27.8(7)	-5.1(6)	14.5(6)	-6.7(7)

C19	33.2(8)	40.0(10)	32.1(8)	4.6(7)	3.1(6)	4.8(7)
C20	29.2(7)	23.6(8)	23.0(6)	-0.5(5)	4.7(5)	-4.7(6)
O21	39.4(6)	30.0(7)	43.1(6)	3.7(5)	21.1(5)	-1.5(5)
O22	30.4(5)	28.0(6)	38.8(6)	12.7(5)	7.8(4)	-0.9(4)
C23	41.3(9)	28.7(9)	39.8(8)	12.8(7)	7.3(7)	-4.7(7)

Table S4. Bond Lengths for ***(R)*-5b**.

Atom	Atom	Length/Å	Atom	Atom	Length/Å
N1	C2	1.3851(19)	C9	N10	1.4577(18)
N1	C7A	1.3773(19)	N10	C11	1.469(2)
N1	C8	1.4636(18)	N10	C13	1.341(2)
C2	C3	1.358(2)	C11	C12	1.523(2)
C3	C3A	1.430(2)	C13	O14	1.2215(19)
C3A	C4	1.397(2)	C13	O15	1.3491(17)
C3A	C7A	1.4188(19)	O15	C16	1.4756(18)
C4	C5	1.385(2)	C16	C17	1.517(2)
C5	C6	1.417(2)	C16	C18	1.520(2)
C5	C20	1.485(2)	C16	C19	1.517(2)
C6	C7	1.380(2)	C20	O21	1.2087(19)
C7	C7A	1.393(2)	C20	O22	1.338(2)
C8	C9	1.535(2)	O22	C23	1.4433(19)
C8	C12	1.527(2)			

Table S5. Bond Angles for ***(R)*-5b**.

Atom	Atom	Atom	Angle/°	Atom	Atom	Atom	Angle/°
C2	N1	C8	127.35(13)	N10	C9	C8	102.99(13)
C7A	N1	C2	108.31(12)	C9	N10	C11	113.37(13)
C7A	N1	C8	123.96(13)	C13	N10	C9	120.11(13)
C3	C2	N1	110.18(14)	C13	N10	C11	126.31(11)
C2	C3	C3A	107.07(14)	N10	C11	C12	102.75(12)
C4	C3A	C3	134.14(14)	C11	C12	C8	103.30(12)
C4	C3A	C7A	119.08(14)	N10	C13	O15	110.85(13)
C7A	C3A	C3	106.77(13)	O14	C13	N10	123.27(12)
C5	C4	C3A	119.46(13)	O14	C13	O15	125.87(13)
C4	C5	C6	120.35(13)	C13	O15	C16	120.99(12)
C4	C5	C20	117.61(12)	O15	C16	C17	111.43(11)
C6	C5	C20	122.01(14)	O15	C16	C18	101.57(12)
C7	C6	C5	121.36(14)	O15	C16	C19	109.09(12)
C6	C7	C7A	117.79(13)	C17	C16	C18	110.14(13)
N1	C7A	C3A	107.64(13)	C19	C16	C17	112.54(15)
N1	C7A	C7	130.41(13)	C19	C16	C18	111.55(13)
C7	C7A	C3A	121.94(13)	O21	C20	C5	123.89(15)
N1	C8	C9	113.70(13)	O21	C20	O22	123.16(15)
N1	C8	C12	113.63(12)	O22	C20	C5	112.96(12)
C12	C8	C9	105.07(12)	C20	O22	C23	115.30(12)

Table S6. Torsion Angles for ***(R)*-5b**.

A	B	C	D	Angle/°	A	B	C	D	Angle/°
N1	C2	C3	C3A	0.77(19)	C7A	N1	C2	C3	-1.69(18)
N1	C8	C9	N10	148.70(12)	C7A	N1	C8	C9	154.16(13)
N1	C8	C12	C11	-160.09(13)	C7A	N1	C8	C12	-85.71(17)
C2	N1	C7A	C3A	1.90(16)	C7A	C3A	C4	C5	1.1(2)
C2	N1	C7A	C7	-177.04(15)	C8	N1	C2	C3	-174.79(15)
C2	N1	C8	C9	-33.7(2)	C8	N1	C7A	C3A	175.29(13)
C2	N1	C8	C12	86.38(19)	C8	N1	C7A	C7	-3.7(2)
C2	C3	C3A	C4	179.41(16)	C8	C9	N10	C11	-3.35(16)
C2	C3	C3A	C7A	0.40(18)	C8	C9	N10	C13	-178.35(12)
C3	C3A	C4	C5	-177.87(16)	C9	C8	C12	C11	-35.19(15)
C3	C3A	C7A	N1	-1.42(16)	C9	N10	C11	C12	-18.33(16)
C3	C3A	C7A	C7	177.63(14)	C9	N10	C13	O14	-1.3(2)
C3A	C4	C5	C6	-0.3(2)	C9	N10	C13	O15	179.79(12)
C3A	C4	C5	C20	177.54(13)	N10	C11	C12	C8	32.11(15)
C4	C3A	C7A	N1	179.39(12)	N10	C13	O15	C16	-172.95(11)
C4	C3A	C7A	C7	-1.6(2)	C11	N10	C13	O14	-175.58(14)
C4	C5	C6	C7	0.0(2)	C11	N10	C13	O15	5.5(2)
C4	C5	C20	O21	-6.6(2)	C12	C8	C9	N10	23.84(15)
C4	C5	C20	O22	173.57(13)	C13	N10	C11	C12	156.29(14)
C5	C6	C7	C7A	-0.5(2)	C13	O15	C16	C17	-59.05(16)

C5	C20	O22	C23	178.81(13)	C13	O15	C16	C18	-176.30(12)
C6	C5	C20	O21	171.23(16)	C13	O15	C16	C19	65.82(16)
C6	C5	C20	O22	-8.61(19)	O14	C13	O15	C16	8.2(2)
C6	C7	C7A	N1	-179.93(14)	C20	C5	C6	C7	-177.74(14)
C6	C7	C7A	C3A	1.3(2)	O21	C20	O22	C23	-1.0(2)

Table S7. Hydrogen Atom Coordinates (Å×10^4) and Isotropic Displacement Parameters (Å^2×10^3) for ***(R)*-3b**.

**Atom**	***x***	***y***	***z***	***U*(iso)**
H2	9558	1709.32	4578.45	37
H3	10961.97	3142.82	3350.85	38
H4	10561.58	6581.22	2102.11	30
H6	7161.98	9332.91	2617.56	33
H7	6627.99	7032.5	3779.31	34
H8	6340(20)	4190(30)	4570(15)	25(4)
H9A	6122.74	1122.61	4596.41	34
H9B	7711.52	831.98	5126.81	34
H11A	7074.6	3353.23	7528.21	34
H11B	5734.08	4013.37	6691.24	34
H12A	8519(19)	4190(30)	6371(14)	22(4)
H12B	7351(19)	5840(30)	6091(16)	30(5)
H17A	3767.52	-1688.17	7670.62	43
H17B	4390.13	-3304.08	8472.92	43

H17C	4861.68	-3065.78	7309.96	43
H18A	6071.44	680.91	9720.34	50
H18B	5190.81	-1047.45	9976.67	50
H18C	4492.08	522.1	9171.51	50
H19A	7333.74	-2778.39	8122.92	53
H19B	6993.62	-3099.11	9324.13	53
H19C	7808.39	-1309.34	9066.99	53
H23A	8792.4	11967.58	-64.61	55
H23B	9549.62	12993.54	1010.09	55
H23C	7954.94	13342.72	575.82	55

X-ray analysis of compound (*S*)-5b:

Single crystals of $C_{19}H_{24}N_2O_4$ **(*S*)-5b** were investigated on a Rigaku XtaLAB Synergy Dualflex HyPix diffractometer. The crystal was maintained at 160.0(1) K during data collection. Using Olex2 [1], the structure was solved with the Superflip [2] structure-solution program using Charge Flipping and refined with the ShelXL [3] using Least Squares minimization.

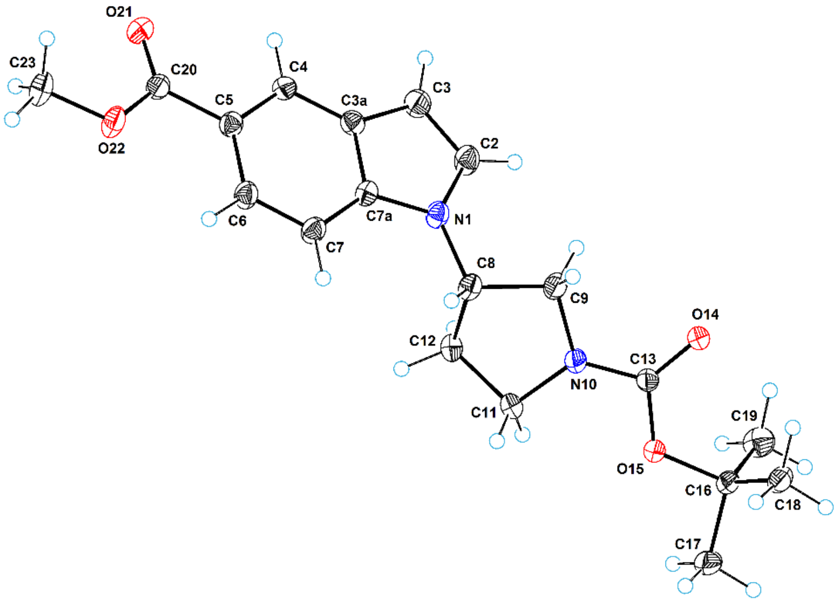

Figure S201. ORTEP view of the asymmetric unit of the crystal **(*S*)-5b**.

Crystal Data for $C_{19}H_{24}N_2O_4$ (M = 344.40 g/mol): monoclinic, space group $P2_1$ (no. 4), a = 9.9263(1) Å, b = 7.3344(1) Å, c = 12.4136(1) Å, β = 99.772(1)°, V = 890.64(2) Å^3, Z = 2, T = 160.0(1) K, μ(CuKα) = 0.738 mm^{-1}, $Dcalc$ = 1.284 g/cm^3, 15828 reflections measured ($2\Theta \leq$ 160°), 3849 unique (R_{int} = 0.0213, R_{sigma} = 0.0166) which were used in all calculations. The final R_1 was 0.0263 ($I > 2\sigma(I)$) and wR_2 was 0.0710 (all data).

Table S8. Crystal data and structure refinement for **(*S*)-5b**.

**Empirical formula**	$C_{19}H_{24}N_2O_4$	**μ/mm^{-1}**	0.738
**Formula weight**	344.40	***F*(000)**	368.0
**Temperature/K**	160.0(1)	**Crystal size/mm^3**	0.23 × 0.14 × 0.11
**Crystal system**	monoclinic	**Radiation**	CuKα (λ = 1.54184 Å)
**Space group**	$P2_1$	**2Θ max. for data collection**	160
***a*/Å**	9.9263(1)	**Index ranges**	$-12 \leq h \leq 12$, $-9 \leq k \leq 9$, $-15 \leq l \leq 14$
***b*/Å**	7.3344(1)	**Reflections collected**	15828
***c*/Å**	12.4136(1)	**Independent reflections**	3849 [R_{int} = 0.0213, R_{sigma} = 0.0166]
**α/°**	90	**Data/restraints/parameters**	3849/1/239
**β/°**	99.772(1)	**Goodness-of-fit on *F*^{2}**	1.067
**γ/°**	90	**Final *R* indexes [*I* > 2σ(*I*)]**	R_1 = 0.0263, wR_2 = 0.0709
**Volume/Å^3**	890.640(17)	**Final *R* indexes [all data]**	R_1 = 0.0265, wR_2 = 0.0710
Z	2	**Largest diff. peak/hole / e Å^{-3}**	0.18/-0.12
**ρ_{calc}mg/mm^3**	1.284	**Flack′s *x* parameter**	0.01(4)

Table S9. Fractional Atomic Coordinates (×10^4) and Equivalent Isotropic Displacement Parameters (Å^2×10^3) for ***(S)*-5b**. U_{eq} is defined as 1/3 of of the trace of the orthogonalised U_{IJ} tensor.

**Atom**	***x***	***y***	***z***	***U*(eq)**
N1	1790.9(13)	6101.7(18)	5749.1(11)	26.4(3)
C2	650.1(16)	7166(2)	5799.3(14)	32.9(3)
C3	-131.5(17)	6383(3)	6468.4(15)	34.3(4)
C3A	527.4(14)	4719(2)	6860.4(12)	25.5(3)
C4	223.0(15)	3338(2)	7553.9(12)	26.6(3)
C5	1080.6(15)	1837(2)	7743.0(12)	25.8(3)
C6	2257.7(16)	1709(2)	7244.1(14)	30.0(3)
C7	2583.7(16)	3063(2)	6557.1(14)	30.1(3)
C7A	1720.4(14)	4574(2)	6381.7(11)	24.0(3)
C8	2828.0(15)	6385(2)	5056.3(12)	26.2(3)
C9	3114.4(18)	8403(2)	4854.0(12)	30.2(3)
N10	3472.4(14)	8386.4(18)	3762.5(10)	27.6(3)
C11	3361.3(17)	6576(2)	3250.3(13)	30.2(3)
C12	2452.9(17)	5550(2)	3917.4(13)	28.5(3)
C13	3761.0(14)	9956(2)	3291.7(12)	22.5(3)
O14	3762.4(11)	11438.4(15)	3740.0(9)	27.5(2)
O15	4060.4(12)	9647.7(14)	2288.4(9)	28.2(2)
C16	4258.8(16)	11170(2)	1555.9(12)	25.9(3)
C17	4661(2)	10171(3)	586.9(14)	36.4(4)
C18	5411.3(17)	12415(2)	2071.9(13)	30.9(3)

C19	2915.6(18)	12180(3)	1236.9(15)	39.0(4)
C20	687.3(16)	361(2)	8450.5(12)	27.2(3)
O21	-383.7(14)	321.6(18)	8789.1(11)	39.5(3)
O22	1644.8(12)	-932.2(17)	8673.3(10)	35.1(3)
C23	1306(2)	-2420(3)	9340.0(16)	40.3(4)

Table S10. Anisotropic Displacement Parameters (Å^2×10^3) for ***(S)*-5b**. The Anisotropic displacement factor exponent takes the form: $-2\pi^2[h^2a^{*2}U_{11}+2hka^*b^*U_{12}+\ldots]$.

**Atom**	U_{11}	U_{22}	U_{33}	U_{23}	U_{13}	U_{12}
N1	24.1(6)	25.0(7)	29.6(6)	5.9(5)	3.5(5)	0.7(5)
C2	29.7(7)	28.9(8)	39.9(9)	10.3(7)	5.3(6)	5.4(6)
C3	30.3(8)	30.7(8)	43.5(9)	5.8(7)	10.5(7)	6.9(7)
C3A	24.5(6)	24.5(7)	27.2(7)	-2.1(6)	3.7(5)	-0.2(6)
C4	27.3(7)	25.9(7)	28.4(7)	-1.6(6)	9.3(6)	-2.0(6)
C5	27.2(7)	24.9(7)	25.2(7)	-0.3(5)	4.1(5)	-3.9(5)
C6	27.9(7)	25.3(8)	37.7(8)	7.4(6)	7.8(6)	2.8(6)
C7	25.3(7)	29.0(8)	37.7(8)	7.0(6)	10.6(6)	1.5(6)
C7A	23.8(6)	24.0(7)	23.5(6)	2.4(6)	2.2(5)	-3.2(5)
C8	23.8(7)	26.1(7)	28.5(7)	5.6(6)	3.6(5)	-0.6(6)
C9	40.2(8)	26.7(7)	24.1(7)	0.1(6)	6.9(6)	-8.0(7)
N10	38.5(7)	20.5(6)	25.2(6)	-1.3(5)	9.2(5)	-3.8(6)
C11	39.0(8)	20.1(7)	33.4(8)	-2.8(6)	11.9(6)	-1.3(6)
C12	32.3(8)	20.6(7)	32.8(8)	0.4(6)	6.2(6)	-2.6(6)
C13	21.6(6)	21.2(6)	25.4(6)	0.0(5)	5.6(5)	0.2(5)

O14	34.9(5)	20.5(5)	29.4(5)	-1.8(4)	12.0(4)	-2.0(4)
O15	38.5(6)	20.7(5)	28.5(5)	-0.8(4)	14.9(4)	-2.2(4)
C16	32.8(7)	23.9(7)	22.3(6)	0.8(6)	8.4(6)	-2.9(6)
C17	49.5(10)	34.5(9)	28.7(8)	-5.6(7)	16.4(7)	-7.6(8)
C18	37.6(8)	30.8(8)	25.2(7)	1.4(6)	8.1(6)	-8.3(7)
C19	37.4(8)	44.6(10)	34.4(8)	5.2(8)	4.3(7)	5.1(8)
C20	32.7(7)	25.7(7)	23.6(7)	-0.3(5)	5.8(6)	-5.0(6)
O21	44.3(7)	32.9(6)	47.4(7)	4.5(5)	24.6(6)	-1.2(5)
O22	33.8(6)	31.1(6)	41.4(7)	14.7(5)	8.9(5)	-1.1(5)
C23	46.6(10)	31.7(8)	42.9(9)	13.9(7)	8.9(8)	-5.2(7)

Table S11. Bond Lengths for ***(S)*-5b**.

Atom	Atom	Length/Å	Atom	Atom	Length/Å
N1	C2	1.386(2)	C9	N10	1.4580(19)
N1	C7A	1.3767(18)	N10	C11	1.4681(19)
N1	C8	1.4640(19)	N10	C13	1.3440(19)
C2	C3	1.357(2)	C11	C12	1.523(2)
C3	C3A	1.430(2)	C13	O14	1.2210(19)
C3A	C4	1.395(2)	C13	O15	1.3478(18)
C3A	C7A	1.416(2)	O15	C16	1.4742(18)
C4	C5	1.387(2)	C16	C17	1.519(2)
C5	C6	1.415(2)	C16	C18	1.518(2)
C5	C20	1.487(2)	C16	C19	1.518(2)
C6	C7	1.383(2)	C20	O21	1.208(2)
C7	C7A	1.395(2)	C20	O22	1.338(2)
C8	C9	1.536(2)	O22	C23	1.444(2)
C8	C12	1.528(2)			

Table S12. Bond Angles for ***(S)*-5b**.

Atom	Atom	Atom	Angle/°	Atom	Atom	Atom	Angle/°
C2	N1	C8	127.44(13)	N10	C9	C8	102.99(13)
C7A	N1	C2	108.17(12)	C9	N10	C11	113.46(12)
C7A	N1	C8	124.04(13)	C13	N10	C9	120.02(13)
C3	C2	N1	110.22(14)	C13	N10	C11	126.33(12)
C2	C3	C3A	107.08(14)	N10	C11	C12	102.66(12)
C4	C3A	C3	134.02(14)	C11	C12	C8	103.37(12)
C4	C3A	C7A	119.26(14)	N10	C13	O15	110.77(12)
C7A	C3A	C3	106.72(13)	O14	C13	N10	123.26(13)
C5	C4	C3A	119.32(13)	O14	C13	O15	125.96(14)
C4	C5	C6	120.45(14)	C13	O15	C16	121.08(11)
C4	C5	C20	117.55(13)	O15	C16	C17	101.74(12)
C6	C5	C20	121.96(14)	O15	C16	C18	111.42(12)
C7	C6	C5	121.30(14)	O15	C16	C19	109.10(13)
C6	C7	C7A	117.70(14)	C18	C16	C17	110.12(14)
N1	C7A	C3A	107.79(13)	C18	C16	C19	112.48(15)
N1	C7A	C7	130.24(13)	C19	C16	C17	111.49(14)
C7	C7A	C3A	121.95(13)	O21	C20	C5	123.89(15)
N1	C8	C9	113.65(13)	O21	C20	O22	123.18(15)
N1	C8	C12	113.66(12)	O22	C20	C5	112.93(13)
C12	C8	C9	104.95(12)	C20	O22	C23	115.36(13)

Table S13. Torsion Angles for ***(S)*-5b**.

A	B	C	D	Angle/°	A	B	C	D	Angle/°
N1	C2	C3	C3A	-0.7(2)	C7A	N1	C2	C3	1.49(19)
N1	C8	C9	N10	-148.51(13)	C7A	N1	C8	C9	-153.90(13)
N1	C8	C12	C11	159.99(13)	C7A	N1	C8	C12	86.14(17)
C2	N1	C7A	C3A	-1.65(16)	C7A	C3A	C4	C5	-1.2(2)
C2	N1	C7A	C7	176.86(16)	C8	N1	C2	C3	174.76(16)
C2	N1	C8	C9	33.8(2)	C8	N1	C7A	C3A	-175.20(13)
C2	N1	C8	C12	-86.1(2)	C8	N1	C7A	C7	3.3(2)
C2	C3	C3A	C4	-179.52(17)	C8	C9	N10	C11	3.18(18)
C2	C3	C3A	C7A	-0.32(19)	C8	C9	N10	C13	178.34(13)
C3	C3A	C4	C5	177.89(17)	C9	C8	C12	C11	35.21(16)
C3	C3A	C7A	N1	1.22(17)	C9	N10	C11	C12	18.53(18)
C3	C3A	C7A	C7	-177.45(15)	C9	N10	C13	O14	1.2(2)
C3A	C4	C5	C6	0.4(2)	C9	N10	C13	O15	-179.80(13)
C3A	C4	C5	C20	-177.52(13)	N10	C11	C12	C8	-32.25(16)
C4	C3A	C7A	N1	-179.44(13)	N10	C13	O15	C16	172.96(12)
C4	C3A	C7A	C7	1.9(2)	C11	N10	C13	O14	175.65(15)
C4	C5	C6	C7	-0.2(2)	C11	N10	C13	O15	-5.3(2)
C4	C5	C20	O21	6.6(2)	C12	C8	C9	N10	-23.73(16)
C4	C5	C20	O22	-173.59(13)	C13	N10	C11	C12	-156.27(15)
C5	C6	C7	C7A	0.8(2)	C13	O15	C16	C17	176.28(13)

C5	C20	O22	C23	-178.81(14)	C13	O15	C16	C18	58.97(18)
C6	C5	C20	O21	-171.28(17)	C13	O15	C16	C19	-65.82(17)
C6	C5	C20	O22	8.5(2)	O14	C13	O15	C16	-8.0(2)
C6	C7	C7A	N1	-179.97(15)	C20	C5	C6	C7	177.66(15)
C6	C7	C7A	C3A	-1.6(2)	O21	C20	O22	C23	1.0(2)

Table S14. Hydrogen Atom Coordinates (Å×10^4) and Isotropic Displacement Parameters (Å^2×10^3) for ***(S)*-5b**.

**Atom**	***x***	***y***	***z***	***U*(iso)**
H2	444.7	8283.59	5419.79	40
H3	-980(30)	6870(40)	6650(20)	50(7)
H4	-563.53	3425.21	7893.1	32
H6	2835.27	673.21	7384.09	36
H7	3368.45	2967.79	6216.29	36
H8	3660(20)	5850(30)	5434(16)	28(5)
H9A	2294.25	9161.73	4875.46	36
H9B	3880.06	8867.78	5401.43	36
H11A	4268.64	5989.04	3306.69	36
H11B	2932.38	6649.64	2471.02	36
H12A	2651.73	4226.81	3931.93	34
H12B	1473.71	5742.34	3618.07	34
H17A	5498.27	9469.83	829.18	55
H17B	4821.08	11056.28	31.15	55
H17C	3923.31	9340.54	275.14	55

H18A	5139.47	13058.43	2692.82	46
H18B	5607.48	13303.07	1529.53	46
H18C	6231.02	11685.97	2326.66	46
H19A	2195.62	11313.26	941.96	59
H19B	3010.73	13097.21	680.52	59
H19C	2673.31	12782.52	1882.41	59
H23A	459.32	-3002.17	8980.65	60
H23B	1180.09	-1953.13	10055.82	60
H23C	2049.32	-3316.54	9436.31	60

X-ray analysis of compound (*R*)-5c:

Single crystals of $C_{19}H_{24}N_2O_4$ **(*R*)-5c** were investigated on a Rigaku XtaLAB Synergy Dualflex HyPix diffractometer. The crystal was maintained at 150.0(1) K during data collection. Using Olex2 [1], the structure was solved with the ShelXT [2] structure-solution program using Intrinsic Phasing and refined with the olex2.refine [3] using the Gauss-Newton minimization package.

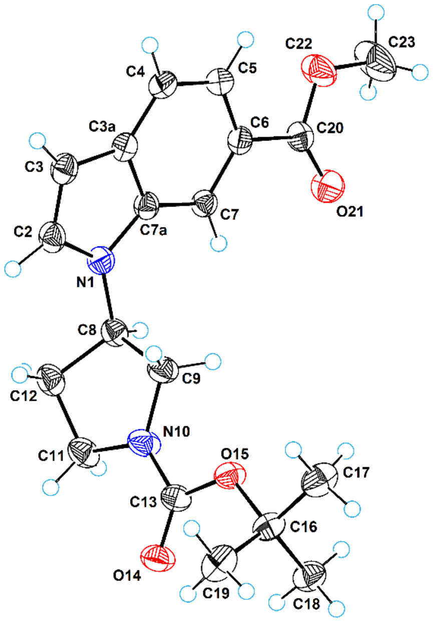

Figure S202. ORTEP view of the asymmetric unit of the crystal **(*R*)-5c**.

Crystal Data for $C_{19}H_{24}N_2O_4$ (M = 344.41 g/mol): monoclinic, space group $P2_1$ (no. 4), a = 10.7848(2) Å, b = 6.4110(1) Å, c = 13.0714(2) Å, β = 98.211(2)°, V = 894.51(3) Å^3, Z = 2, T = 150.0(1) K, μ(Cu Kα) = 0.735 mm^{-1}, $Dcalc$ = 1.2786 g/cm^3, 14738 reflections measured (2Θ ≤ 160°), 3837 unique (R_{int} = 0.0380, R_{sigma} = 0.0361) which were used in all calculations. The final R_1 was 0.0393 ($I > 2\sigma(I)$) and wR_2 was 0.1015 (all data).

Table S15. Crystal data and structure refinement for **(*R*)-5c**.

**Empirical formula**	$C_{19}H_{24}N_2O_4$	**μ/mm^{-1}**	0.735
**Formula weight**	344.41	***F*(000)**	368
**Temperature/K**	150.0(1)	**Crystal size/mm^3**	0.18 × 0.02 × 0.01
**Crystal system**	monoclinic	**Radiation**	Cu Kα (λ = 1.54184 Å)
**Space group**	$P2_1$	**2Θ max. for data collection**	160
***a*/Å**	10.78483(19)	**Index ranges**	-13 ≤ h ≤ 13, -8 ≤ k ≤ 8, -16 ≤ l ≤ 13
***b*/Å**	6.41103(10)	**Reflections collected**	14738
***c*/Å**	13.0714(2)	**Independent reflections**	3837 [R_{int} = 0.0380, R_{sigma} = 0.0361]
**α/°**	90	**Data/restraints/parameters**	3837/1/246
**β/°**	98.2111(16)	**Goodness-of-fit on F^2**	1.079
**γ/°**	90	**Final *R* indexes [$I > 2\sigma(I)$]**	R_1 = 0.0393, wR_2 = 0.1004
**Volume/Å^3**	894.51(3)	**Final *R* indexes [all data]**	R_1 = 0.0409, wR_2 = 0.1015
Z	2	**Largest diff. peak/hole / e Å^{-3}**	0.18/-0.16
**ρ_{calc}mg/mm^3**	1.2786	**Flack's *x* parameter**	0.05(8)

Table S16. Fractional Atomic Coordinates (×10^4) and Equivalent Isotropic Displacement Parameters (Å^2×10^3) for ***(R)*-5c**. U_{eq} is defined as 1/3 of the trace of the orthogonalised U_{IJ} tensor.

**Atom**	***x***	***y***	***z***	***U*(eq)**
N1	3112.4(14)	1730(2)	5377.9(11)	29.3(3)
C2	4227.1(18)	665(3)	5639.3(15)	32.3(4)
C3	4942.5(17)	1627(3)	6446.3(14)	32.9(4)
C3A	4272.6(16)	3432(3)	6704.4(13)	28.1(3)
C4	4521.5(18)	5035(3)	7432.4(14)	31.6(4)
C5	3629.8(17)	6563(3)	7477.5(13)	31.0(4)
C6	2488.3(16)	6543(3)	6799.0(13)	27.9(3)
C7	2232.9(16)	5005(3)	6050.4(13)	26.8(3)
C7A	3131.3(16)	3455(3)	6019.2(12)	26.6(3)
C8	2202.0(17)	1459(3)	4449.3(13)	29.0(3)
C9	2583(2)	2652(3)	3526.0(14)	34.2(4)
N10	1968.3(17)	1472(3)	2644.0(12)	36.9(4)
C11	1385(2)	-448(3)	2945.6(15)	35.7(4)
C12	2008(2)	-773(3)	4057.0(16)	35.0(4)
C13	1929.9(17)	1998(3)	1641.5(14)	30.4(4)
O14	1423.2(14)	948(2)	928.7(10)	38.9(3)
O15	2548.4(13)	3824(2)	1560.4(9)	32.7(3)
C16	2714.5(17)	4612(3)	525.7(14)	33.0(4)
C17	3509(2)	6550(4)	788.2(16)	44.3(5)
C18	1452.7(19)	5224(4)	-70.4(16)	38.8(4)
C19	3416(2)	3018(4)	-26.6(17)	45.0(5)
C20	1522.7(17)	8162(3)	6864.4(14)	30.7(4)
O21	618.0(13)	8454(2)	6219.7(11)	40.4(3)
O22	1739.1(15)	9224(2)	7749.3(12)	49.1(4)
C23	817(3)	10779(4)	7908(3)	67.6(9)

Table S17. Anisotropic Displacement Parameters (Å^2×10^3) for ***(R)*-5c**. The Anisotropic displacement factor exponent takes the form: $-2\pi^2[h^2a^{*2}U_{11}+2hka^*b^*U_{12}+\ldots]$.

**Atom**	U_{11}	U_{22}	U_{33}	U_{12}	U_{13}	U_{23}
N1	29.5(7)	28.9(7)	29.6(7)	3.0(6)	4.9(6)	-0.8(6)
C2	34.4(9)	28.5(9)	35.1(9)	7.2(7)	8.9(7)	-0.3(7)
C3	30.4(8)	31.5(9)	36.9(9)	7.0(8)	5.0(7)	2.3(8)
C3A	28.4(8)	27.9(8)	28.6(8)	3.2(7)	5.9(6)	3.7(7)
C4	30.2(9)	33.5(9)	29.7(8)	2.0(7)	-0.5(7)	2.2(7)
C5	33.8(9)	29.9(8)	28.3(8)	0.2(8)	1.6(7)	-0.5(7)
C6	30.6(8)	24.9(8)	28.7(7)	1.8(7)	5.3(6)	2.6(7)
C7	26.2(8)	27.4(8)	26.9(8)	2.7(7)	4.0(6)	0.7(7)
C7A	28.3(8)	25.0(8)	27.2(8)	1.1(7)	6.7(6)	2.0(7)
C8	30.6(8)	26.3(8)	30.6(8)	-2.3(7)	6.7(7)	-1.3(7)
C9	40.7(10)	33.1(10)	28.5(8)	-10.3(8)	3.6(7)	-1.5(7)
N10	45.9(9)	35.1(8)	29.7(7)	-17.7(8)	5.3(6)	-2.5(7)
C11	38.0(10)	31.9(9)	38.1(10)	-10.5(8)	8.9(8)	-5.1(7)
C12	41.4(10)	26.9(8)	38.2(10)	-5.5(8)	10.4(8)	-0.8(7)
C13	26.9(8)	33.2(10)	30.8(8)	-4.1(7)	3.2(7)	-3.9(7)
O14	40.4(8)	40.7(8)	33.6(7)	-7.4(6)	-1.4(6)	-7.2(6)
O15	33.6(7)	36.5(7)	27.3(6)	-9.9(5)	2.2(5)	1.6(5)
C16	28.8(8)	43.0(11)	27.1(8)	-1.1(8)	3.7(7)	3.8(7)
C17	38.9(10)	53.1(12)	40.4(10)	-13.1(10)	4.2(8)	13.0(10)
C18	33.6(10)	44.7(11)	36.7(10)	3.3(9)	0.2(8)	1.4(9)
C19	39.3(11)	57.9(14)	40.0(10)	12.0(10)	13.4(8)	9.0(10)

C20	29.8(8)	27.8(8)	34.8(9)	1.5(7)	5.6(7)	-0.6(7)
O21	30.5(7)	44.3(8)	45.1(7)	7.8(6)	1.3(6)	-3.9(6)
O22	44.4(8)	45.1(9)	54.2(9)	17.8(7)	-5.1(7)	-20.5(7)
C23	51.7(15)	58.8(16)	89(2)	23.6(13)	-1.9(13)	-36.2(15)

Table S18. Bond Lengths for ***(R)*-5c**.

Atom	Atom	Length/Å	Atom	Atom	Length/Å
N1	C2	1.383(2)	C9	N10	1.457(2)
N1	C7A	1.386(2)	N10	C11	1.462(2)
N1	C8	1.458(2)	N10	C13	1.348(2)
C2	C3	1.362(3)	C11	C12	1.525(3)
C3	C3A	1.430(3)	C13	O14	1.215(2)
C3A	C4	1.400(3)	C13	O15	1.359(2)
C3A	C7A	1.415(2)	O15	C16	1.479(2)
C4	C5	1.380(3)	C16	C17	1.520(3)
C5	C6	1.411(2)	C16	C18	1.520(3)
C6	C7	1.389(2)	C16	C19	1.514(3)
C6	C20	1.482(2)	C20	O21	1.209(2)
C7	C7A	1.393(2)	C20	O22	1.334(2)
C8	C9	1.534(2)	O22	C23	1.444(3)
C8	C12	1.524(3)			

Table S19. Bond Angles for ***(R)*-5c**.

Atom	Atom	Atom	Angle/°	Atom	Atom	Atom	Angle/°
C7A	N1	C2	107.91(15)	N10	C9	C8	102.75(14)
C8	N1	C2	127.22(15)	C11	N10	C9	112.87(15)
C8	N1	C7A	123.28(15)	C13	N10	C9	125.90(15)
C3	C2	N1	110.12(16)	C13	N10	C11	121.22(15)
C3A	C3	C2	107.44(16)	C12	C11	N10	102.93(15)
C4	C3A	C3	134.43(17)	C11	C12	C8	102.28(15)
C7A	C3A	C3	106.38(15)	O14	C13	N10	123.70(17)
C7A	C3A	C4	119.19(16)	O15	C13	N10	110.14(14)
C5	C4	C3A	118.87(16)	O15	C13	O14	126.16(17)
C6	C5	C4	121.17(17)	C16	O15	C13	119.40(14)
C7	C6	C5	121.12(16)	C17	C16	O15	102.18(15)
C20	C6	C5	120.98(16)	C18	C16	O15	109.91(15)
C20	C6	C7	117.90(16)	C18	C16	C17	109.86(18)
C7A	C7	C6	117.29(16)	C19	C16	O15	109.83(15)
C3A	C7A	N1	108.12(15)	C19	C16	C17	110.80(18)
C7	C7A	N1	129.56(16)	C19	C16	C18	113.65(17)
C7	C7A	C3A	122.31(16)	O21	C20	C6	124.89(17)
C9	C8	N1	111.78(15)	O22	C20	C6	111.73(15)
C12	C8	N1	115.83(16)	O22	C20	O21	123.32(17)
C12	C8	C9	104.04(14)	C23	O22	C20	116.00(18)

Table S20. Hydrogen Atom Coordinates (Å×10^4) and Isotropic Displacement Parameters (Å^2×10^3) for ***(R)*-5c**.

Atom	*x*	*y*	*z*	*U*(iso)
H2	4370(20)	-690(40)	5281(17)	36(6)
H3	5740.1(17)	1180(3)	6777.0(14)	39.5(4)
H4	5291.1(18)	5070(3)	7887.7(14)	37.9(4)
H5	3787.3(17)	7647(3)	7974.3(13)	37.2(4)
H7	1476.2(16)	5011(3)	5578.6(13)	32.2(4)
H8	1400(20)	2080(40)	4599(17)	33(6)
H9a	2279(2)	4110(3)	3510.3(14)	41.0(5)
H9b	3504(2)	2653(3)	3544.7(14)	41.0(5)
H11a	467(2)	-280(3)	2910.1(15)	42.8(5)
H11b	1558(2)	-1631(3)	2499.9(15)	42.8(5)
H12a	2810(20)	-1510(40)	4045(18)	41(6)
H12b	1490(30)	-1590(50)	4470(20)	61(8)
H17a	3100(9)	7456(15)	1243(12)	66.4(7)
H17b	4337(6)	6140(4)	1140(13)	66.4(7)
H17c	3603(15)	7302(17)	151(2)	66.4(7)
H18a	910(6)	3993(6)	-168(11)	58.2(7)
H18b	1061(7)	6284(19)	319(6)	58.2(7)
H18c	1573(2)	5790(20)	-746(5)	58.2(7)
H19a	4177(9)	2590(20)	426(6)	67.5(8)
H19b	2880(7)	1798(13)	-203(13)	67.5(8)
H19c	3643(15)	3635(10)	-660(8)	67.5(8)

H23a	931(15)	12013(16)	7489(15)	101.3(13)
H23b	-25(3)	10209(14)	7703(19)	101.3(13)
H23c	920(16)	11170(30)	8640(4)	101.3(13)

X-ray analysis of compound (*S*)-5c:

Single crystals of $C_{19}H_{24}N_2O_4$ **(*S*)-5c** were investigated on a Rigaku XtaLAB Synergy Dualflex HyPix diffractometer. The crystal was maintained at 160.0(2) K during data collection. Using Olex2 [1], the structure was solved with the SHELXT [2] structure solution program using Intrinsic Phasing and refined with the olex2.refine [3] using the Levenberg-Marquardt minimization package.

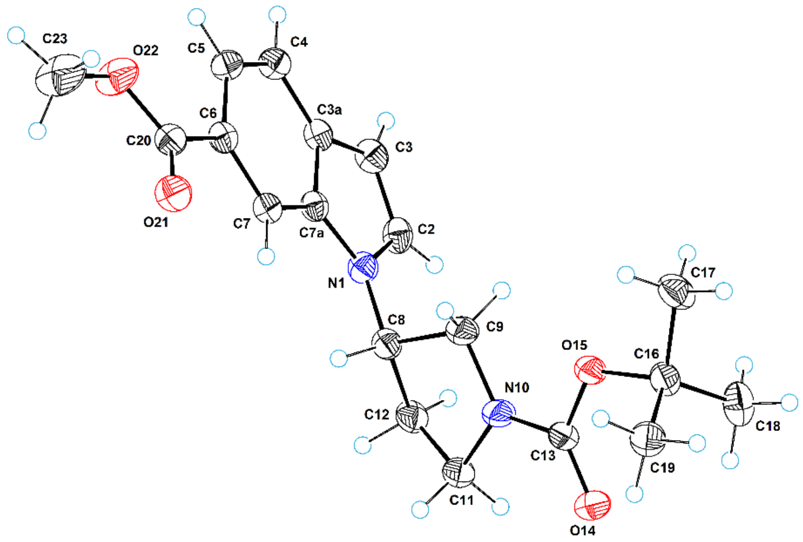

Figure S203. ORTEP view of the asymmetric unit of the crystal **(*S*)-5c**.

Crystal Data for $C_{19}H_{24}N_2O_4$ (M = 344.413 g/mol): monoclinic, space group $P2_1$ (no. 4), a = 10.7917(3) Å, b = 6.4130(2) Å, c = 13.0794(4) Å, β = 98.261(3)°, V = 895.80(5) Å^3, Z = 2, T = 160.0(2) K, μ(Cu Kα) = 0.734 mm^{-1}, $Dcalc$ = 1.277 g/cm^3, 14525 reflections measured (2Θ ≤ 160°), 3815 unique (R_{int} = 0.0510, R_{sigma} = 0.0466) which were used in all calculations. The final R_1 was 0.0424 ($I > 2\sigma(I)$) and wR_2 was 0.0986 (all data).

Table S21. Crystal data and structure refinement for **(*S*)-5c**.

**Empirical formula**	$C_{19}H_{24}N_2O_4$	**μ/mm^{-1}**	0.734
**Formula weight**	344.413	***F*(000)**	368
**Temperature/K**	160.0(2)	**Crystal size/mm^3**	0.11 × 0.08 × 0.01
**Crystal system**	monoclinic	**Radiation**	CuKα (λ = 1.54184 Å)
**Space group**	$P2_1$	**2Θ max. for data collection**	160°
***a*/Å**	10.7917(3)	**Index ranges**	$-13 \leq h \leq 13$, $-8 \leq k \leq 7$, $-16 \leq l \leq 16$
***b*/Å**	6.4130(2)	**Reflections collected**	14525
***c*/Å**	13.0794(4)	**Independent reflections**	3815[*R*(int) = 0.0510]
**α/°**	90	**Data/restraints/parameters**	3815/1/234
**β/°**	98.261(3)	**Goodness-of-fit on F^2**	1.059
**γ/°**	90	**Final *R* indexes [$I > 2\sigma(I)$]**	$R_1 = 0.0424$, $wR_2 = 0.0963$
**Volume/Å^3**	895.80(5)	**Final *R* indexes [all data]**	$R_1 = 0.0470$, $wR_2 = 0.0986$
Z	2	**Largest diff. peak/hole / e Å^{-3}**	0.18/-0.17
**ρ_{calc}mg/mm^3**	1.277	**Flack's *x* parameter**	0.0(1)

Table S22. Fractional Atomic Coordinates (×10^4) and Equivalent Isotropic Displacement Parameters (Å^2×10^3) for **(*S*)-5c**. U_{eq} is defined as 1/3 of the trace of the orthogonalised U_{IJ} tensor.

Atom	*x*	*y*	*z*	*U*(eq)
N1	6884.9(14)	8277(3)	4624.2(13)	32.0(3)
C2	5772.5(19)	9348(3)	4360.3(16)	35.5(4)
C3	5060.3(18)	8382(3)	3556.6(16)	35.6(4)
C3A	5729.5(16)	6584(3)	3298.2(14)	30.1(4)
C4	5486.3(18)	4983(3)	2570.2(16)	33.7(4)
C5	6371.0(18)	3458(3)	2522.4(15)	33.4(4)
C6	7512.0(17)	3471(3)	3206.9(15)	30.4(4)
C7	7765.5(17)	5008(3)	3951.6(15)	29.5(4)
C7A	6868.9(16)	6558(3)	3983.7(14)	28.6(4)
C8	7794.7(17)	8553(3)	5554.9(15)	31.8(4)
C9	7416(2)	7366(3)	6477.5(16)	36.8(4)
N10	8029.5(17)	8538(3)	7357.6(13)	39.3(4)
C11	8609(2)	10463(3)	7052.3(18)	38.7(5)
C12	7992(2)	10778(3)	5946.7(17)	37.5(4)
C13	8065.6(17)	8020(3)	8359.7(16)	33.1(4)
O14	8574.2(14)	9062(2)	9069.2(12)	41.7(4)
O15	7448.1(13)	6198(2)	8442.8(11)	35.6(3)
C16	7285.7(18)	5409(3)	9474.8(16)	36.8(5)
C17	6490(2)	3479(4)	9218.3(19)	48.7(6)
C18	6590(2)	7003(4)	10028.8(19)	48.6(6)
C19	8544(2)	4801(4)	10070.7(18)	42.3(5)

C20	8479.1(18)	1852(3)	3141.6(16)	33.4(4)
O21	9382.5(13)	1565(3)	3785.4(12)	43.5(4)
O22	8262.6(15)	781(3)	2261.6(14)	53.4(5)
C23	9180(3)	-777(5)	2107(3)	73.4(10)

Table S23. Anisotropic Displacement Parameters ($Å^2 \times 10^3$) for ***(S)*-5c**. The Anisotropic displacement factor exponent takes the form: $-2\pi^2[h^2a^{*2}U_{11}+2hka^*b^*U_{12}+\ldots]$.

**Atom**	U_{11}	U_{22}	U_{33}	U_{12}	U_{13}	U_{23}
N1	31.8(7)	29.3(8)	35.4(8)	2.8(7)	6.9(6)	-1.7(7)
C2	37.3(10)	30.8(10)	40.3(11)	7.3(8)	12.8(8)	0.4(8)
C3	31.3(9)	32.2(10)	43.6(11)	6.0(8)	5.9(8)	3.2(9)
C3A	28.4(8)	29.3(9)	33.3(9)	3.2(8)	6.7(7)	4.2(8)
C4	31.6(9)	34.7(10)	33.9(10)	1.8(8)	1.5(7)	3.1(8)
C5	35.0(9)	30.9(9)	33.6(10)	0.3(8)	2.9(7)	-1.6(8)
C6	30.8(8)	27.6(9)	33.5(9)	1.5(8)	7.4(7)	3.7(8)
C7	27.9(8)	30.1(9)	30.6(9)	1.3(7)	4.8(7)	3.0(8)
C7A	30.2(8)	26.2(9)	30.3(9)	-0.0(7)	7.7(7)	3.5(7)
C8	32.0(9)	27.9(9)	36.6(10)	0.3(8)	8.4(7)	-1.2(8)
C9	40.9(11)	33.0(11)	36.7(11)	-11.2(8)	6.3(8)	-2.4(8)
N10	47.2(9)	36.8(9)	34.5(9)	-18.9(8)	7.6(7)	-3.9(8)
C11	42.7(10)	32.3(10)	42.9(11)	-12.3(9)	12.6(9)	-5.8(8)
C12	41.4(10)	29.3(9)	43.8(11)	-7.3(8)	13.0(9)	-0.0(8)
C13	27.1(8)	35.6(11)	37.0(10)	-4.3(7)	5.5(7)	-4.0(8)
O14	42.3(8)	43.3(9)	37.8(8)	-7.7(6)	0.1(6)	-7.3(7)

O15	35.1(7)	39.4(8)	32.2(7)	-10.9(6)	4.4(5)	1.0(6)
C16	30.8(9)	46.1(12)	33.7(10)	-0.6(8)	5.9(8)	5.1(9)
C17	42.1(11)	57.3(14)	46.5(12)	-13.9(11)	5.9(9)	11.7(12)
C18	43.1(12)	61.8(16)	43.6(12)	12.6(11)	15.0(10)	9.3(11)
C19	36.3(10)	48.6(13)	40.7(12)	3.7(9)	1.0(9)	1.8(10)
C20	33.4(9)	28.7(10)	38.6(10)	1.2(8)	6.5(8)	-0.2(8)
O21	33.0(7)	45.9(9)	50.6(9)	10.5(7)	2.4(6)	-2.7(7)
O22	46.2(8)	49.7(10)	60.8(10)	20.1(8)	-4.2(7)	-22.6(8)
C23	58.6(16)	61.2(17)	97(2)	25.0(14)	-0.9(15)	-39.7(17)

Table S24. Bond Lengths for ***(S)*-5c**.

Atom	Atom	Length/Å	Atom	Atom	Length/Å
N1	C2	1.383(3)	C9	N10	1.452(3)
N1	C7A	1.384(3)	N10	C11	1.465(3)
N1	C8	1.461(3)	N10	C13	1.347(3)
C2	C3	1.359(3)	C11	C12	1.516(3)
C3	C3A	1.427(3)	C13	O14	1.210(2)
C3A	C4	1.399(3)	C13	O15	1.357(2)
C3A	C7A	1.414(3)	O15	C16	1.475(2)
C4	C5	1.375(3)	C16	C17	1.516(3)
C5	C6	1.415(3)	C16	C18	1.513(3)
C6	C7	1.385(3)	C16	C19	1.516(3)
C6	C20	1.484(3)	C20	O21	1.207(3)
C7	C7A	1.392(3)	C20	O22	1.332(3)
C8	C9	1.531(3)	O22	C23	1.441(3)
C8	C12	1.521(3)			

Table S25. Bond Angles for ***(S)*-5c**.

Atom	Atom	Atom	Angle/°	Atom	Atom	Atom	Angle/°
C7A	N1	C2	108.04(16)	N10	C9	C8	102.97(16)
C8	N1	C2	127.10(17)	C11	N10	C9	112.57(17)
C8	N1	C7A	123.34(16)	C13	N10	C9	126.12(17)
C3	C2	N1	109.92(18)	C13	N10	C11	121.29(17)
C3A	C3	C2	107.60(17)	C12	C11	N10	103.10(16)
C4	C3A	C3	134.62(18)	C11	C12	C8	102.54(17)
C7A	C3A	C3	106.44(17)	O14	C13	N10	123.81(19)
C7A	C3A	C4	118.94(17)	O15	C13	N10	110.15(16)
C5	C4	C3A	119.30(17)	O15	C13	O14	126.04(19)
C6	C5	C4	120.93(19)	C16	O15	C13	119.52(15)
C7	C6	C5	121.02(18)	C17	C16	O15	102.44(17)
C20	C6	C5	120.99(18)	C18	C16	O15	109.79(17)
C20	C6	C7	117.98(17)	C18	C16	C17	110.69(19)
C7A	C7	C6	117.47(17)	C19	C16	O15	110.07(16)
C3A	C7A	N1	107.98(16)	C19	C16	C17	109.9(2)
C7	C7A	N1	129.72(17)	C19	C16	C18	113.36(19)
C7	C7A	C3A	122.30(18)	O21	C20	C6	124.82(19)
C9	C8	N1	111.90(16)	O22	C20	C6	111.92(17)
C12	C8	N1	116.07(17)	O22	C20	O21	123.22(18)
C12	C8	C9	103.97(16)	C23	O22	C20	116.10(19)

Table S26. Hydrogen Atom Coordinates (Å×10^4) and Isotropic Displacement Parameters (Å^2×10^3) for ***(S)*-5c**.

**Atom**	***x***	***y***	***z***	***U*(iso)**
H2	5540.5(19)	10577(3)	4691.6(16)	42.6(5)
H3	4261.6(18)	8824(3)	3226.8(16)	42.8(5)
H4	4717.8(18)	4951(3)	2113.8(16)	40.5(5)
H5	6214.0(18)	2380(3)	2022.5(15)	40.0(5)
H7	8522.6(17)	5005(3)	4422.7(15)	35.4(5)
H8	8580(20)	7950(40)	5388(17)	30(5)
H9a	6496(2)	7366(3)	6458.8(16)	44.1(5)
H9b	7719(2)	5908(3)	6492.9(16)	44.1(5)
H11a	8432(2)	11646(3)	7495.3(18)	46.4(6)
H11b	9527(2)	10300(3)	7090.3(18)	46.4(6)
H12a	8545(2)	11563(3)	5541.8(17)	45.0(5)
H12b	7186(2)	11527(3)	5919.0(17)	45.0(5)
H17a	5681(7)	3883(5)	8829(13)	73.0(8)
H17b	6920(9)	2526(15)	8801(13)	73.0(8)
H17c	6353(16)	2782(17)	9859.3(19)	73.0(8)
H18a	7119(7)	8238(13)	10187(13)	73.0(9)
H18b	5816(9)	7400(20)	9586(6)	73.0(9)
H18c	6386(16)	6401(11)	10673(7)	73.0(9)
H19a	8935(8)	3740(20)	9683(7)	63.5(7)
H19b	9086(6)	6032(7)	10168(12)	63.5(7)
H19c	8424(3)	4240(30)	10746(6)	63.5(7)

H23a	9048(16)	-1230(30)	1384(5)	110.1(15)
H23b	10022(3)	-186(14)	2280(20)	110.1(15)
H23c	9094(17)	-1976(19)	2556(15)	110.1(15)

Chiral HPLC analysis

Sample Name : RO-D-35
Injection Volume : 10 uL
Column : CHIRAL ART Cellulose SB
Flow : 1.0 mL/min
Eluent : water/acetonitrile

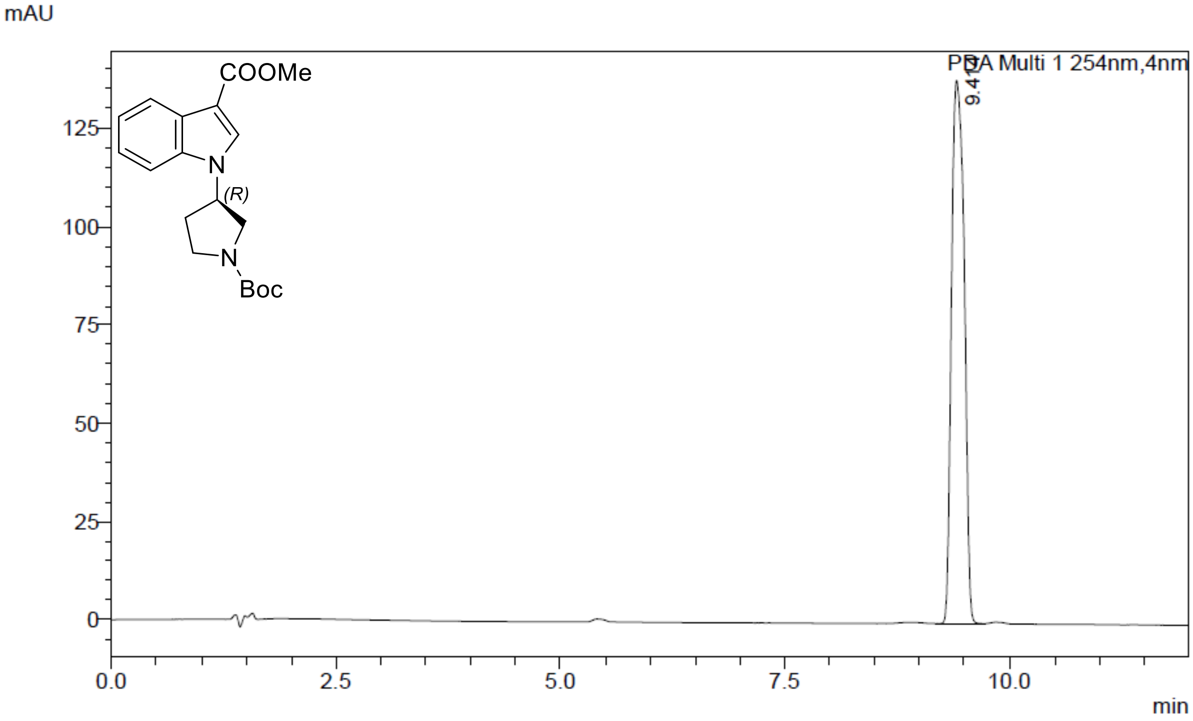

PDA Ch1 254nm

Peak#	Ret. Time	Height	Area	Area%
1	9.414	138144	1359629	100.000

Figure S204. Chiral HPLC chromatogram view of ***(R)*-5a**.

Sample Name : RO-D-30
Injection Volume : 10 uL
Column : CHIRAL ART Cellulose SB
Flow : 1.0 mL/min
Eluent : water/acetonitrile

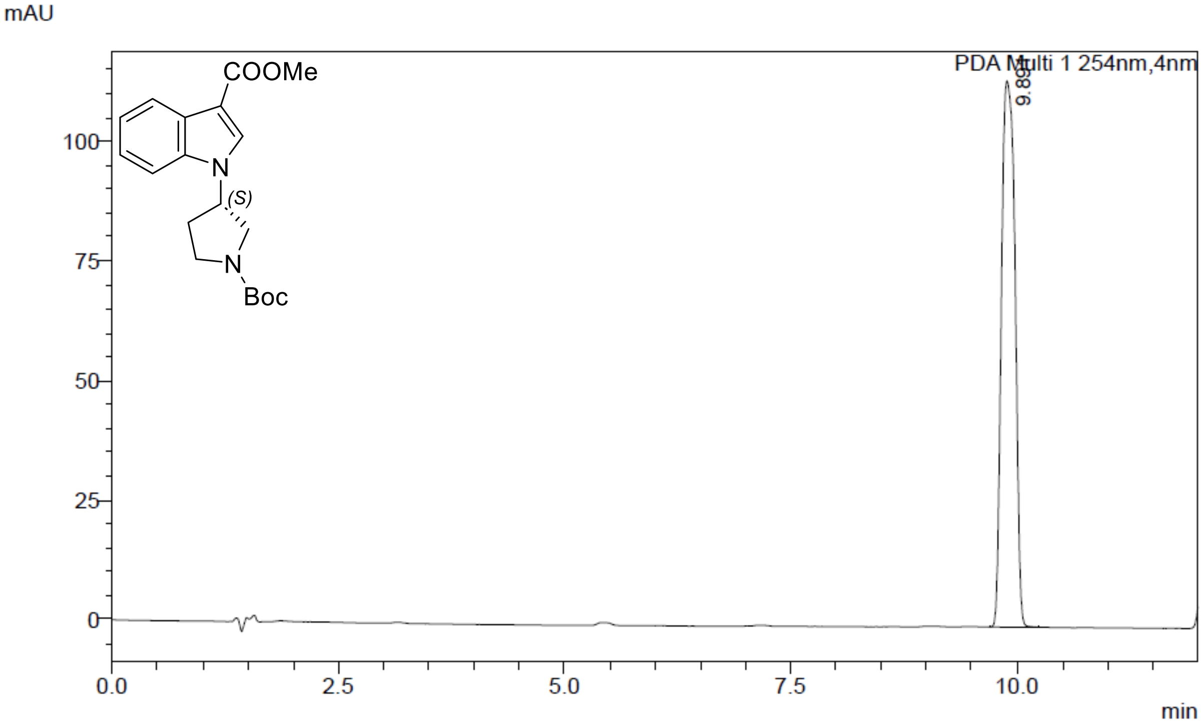

PDA Ch1 254nm

Peak#	Ret. Time	Height	Area	Area%
1	9.891	114006	1185577	100.000

Figure S205. Chiral HPLC chromatogram view of **(*S*)-5a**.

Sample Name : RO-D-37
Injection Volume : 10 uL
Column : CHIRAL ART Cellulose SJ
Flow : 1.0 mL/min
Eluent : water/acetonitrile

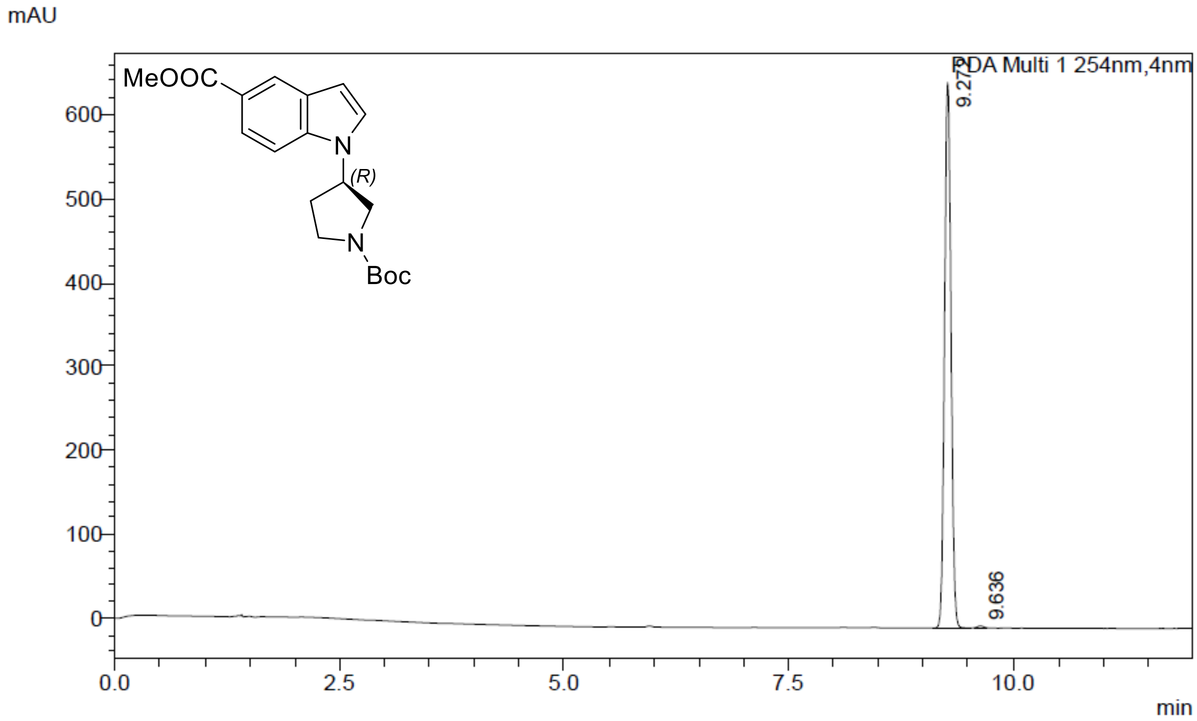

PDA Ch1 254nm

Peak#	Ret. Time	Height	Area	Area%
1	9.272	646967	3265605	99.710
2	9.636	2217	9496	0.290

Figure S206. Chiral HPLC chromatogram view of **(*R*)-5b**.

Sample Name : RO-D-28
Injection Volume : 10 uL
Column : CHIRAL ART Cellulose SJ
Flow : 1.0 mL/min
Eluent : water/acetonitrile

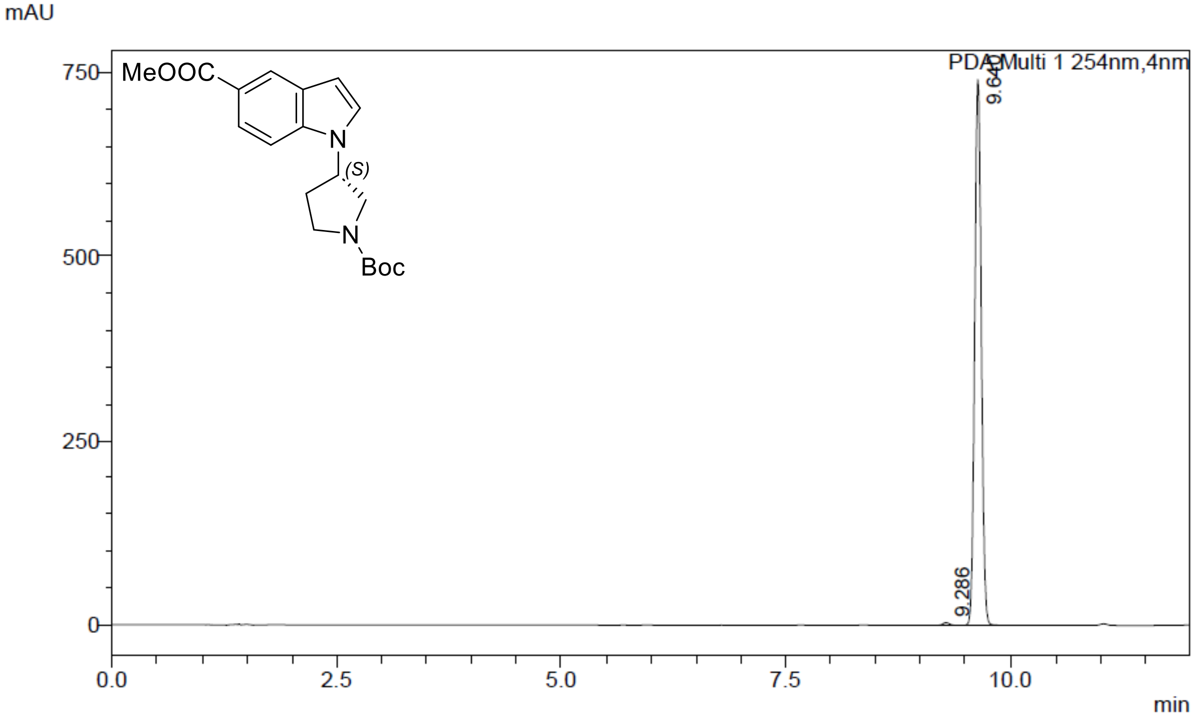

PDA Ch1 254nm

Peak#	Ret. Time	Height	Area	Area%
1	9.286	3748	18160	0.474
2	9.640	738504	3809717	99.526

Figure S207. Chiral HPLC chromatogram view of **(*S*)-5b**.

Sample Name : RO-D-36
Injection Volume : 10 uL
Column : CHIRAL ART Cellulose SJ
Flow : 1.0 mL/min
Eluent : water/acetonitrile

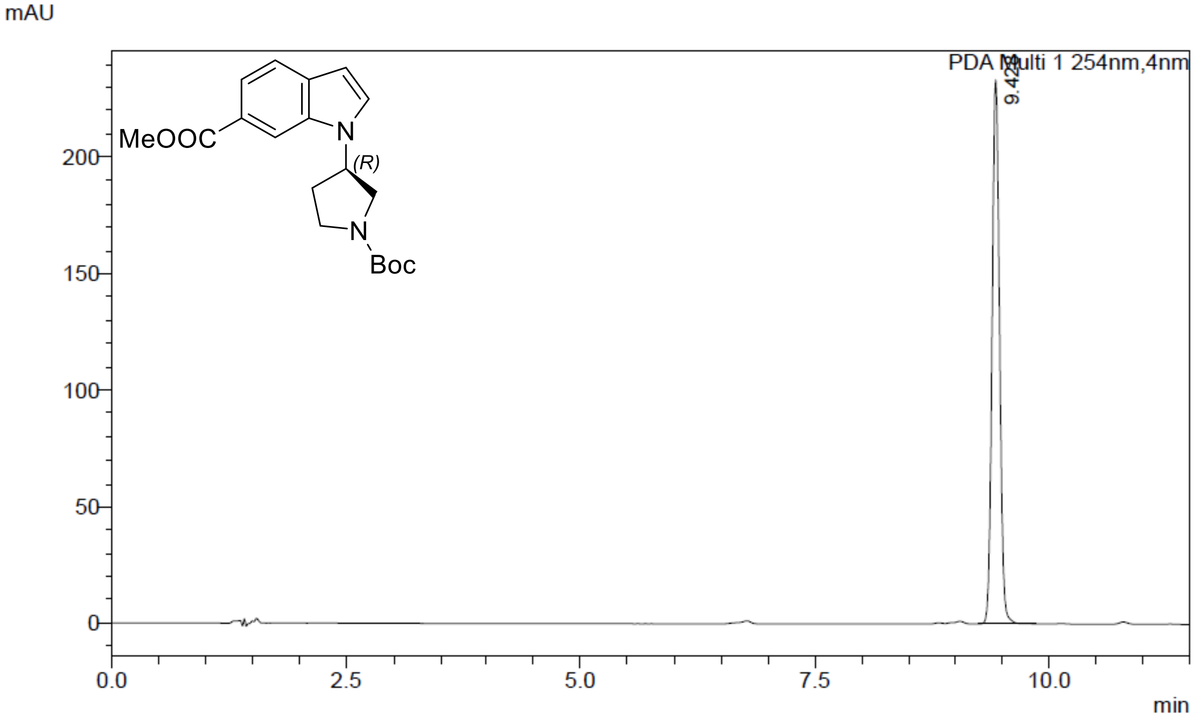

PDA Ch1 254nm

Peak#	Ret. Time	Height	Area	Area%
1	9.428	233040	1295616	100.000

Figure S208. Chiral HPLC chromatogram view of ***(R)*-5c**.

Sample Name : RO-D-27
Injection Volume : 10 uL
Column : CHIRAL ART Cellulose SJ
Flow : 1.0 mL/min
Eluent : water/acetonitrile

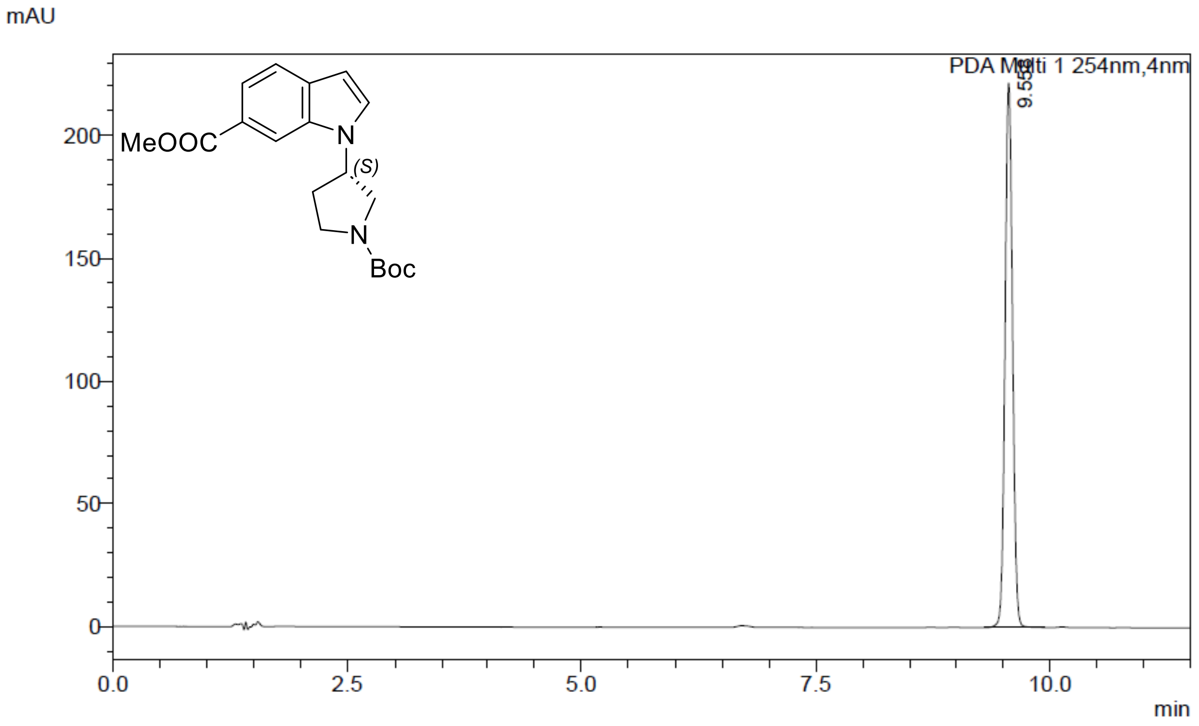

PDA Ch1 254nm

Peak#	Ret. Time	Height	Area	Area%
1	9.558	222001	1238390	100.000

Figure S209. Chiral HPLC chromatogram view of **(*S*)-5c**.

<Sample Information>

Sample Name : MDP-207
Injection Volume : 10 uL
Column : CHIRAL ART Amylose SA
Flow : 1.0 mL/min
Eluent : water/acetonitrile

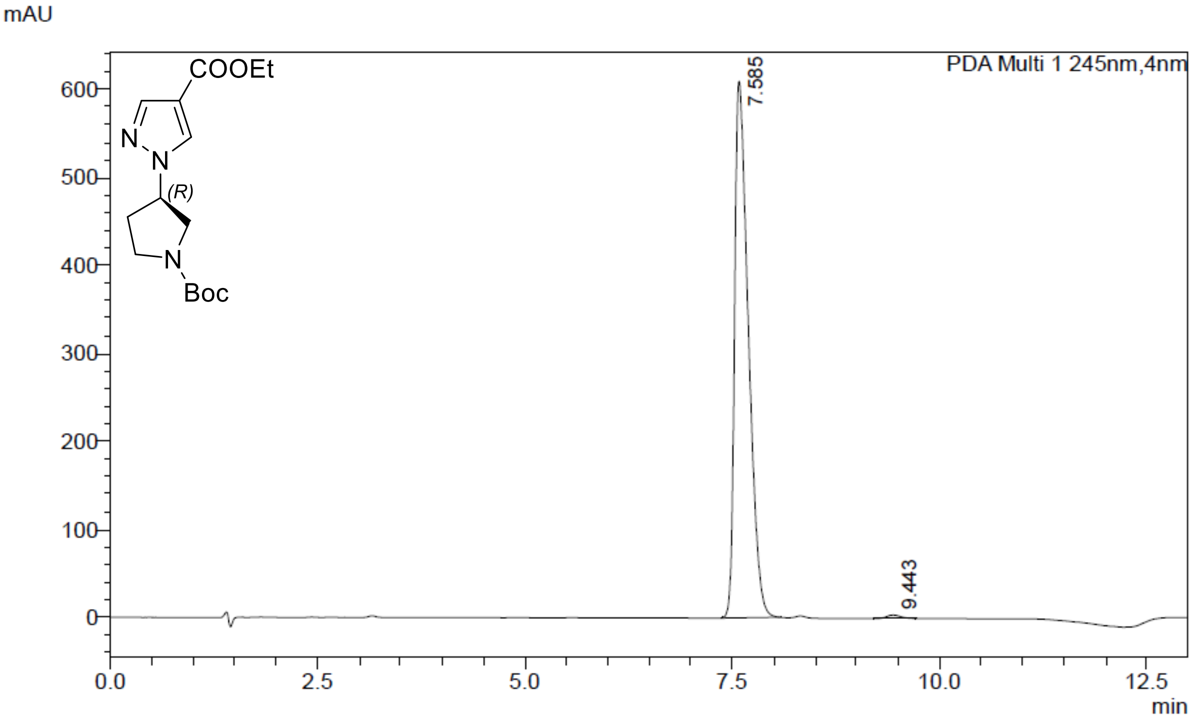

PDA Ch1 245nm

Peak#	Ret. Time	Height	Area	Area%
1	7.585	608898	7054483	99.376
2	9.443	3925	44281	0.624

Figure S210. Chiral HPLC chromatogram view of ***(R)*-14a**.

Sample Name : MDP-209
Injection Volume : 10 uL
Column : CHIRAL ART Amylose SA
Flow : 1.0 mL/min
Eluent : water/acetonitrile

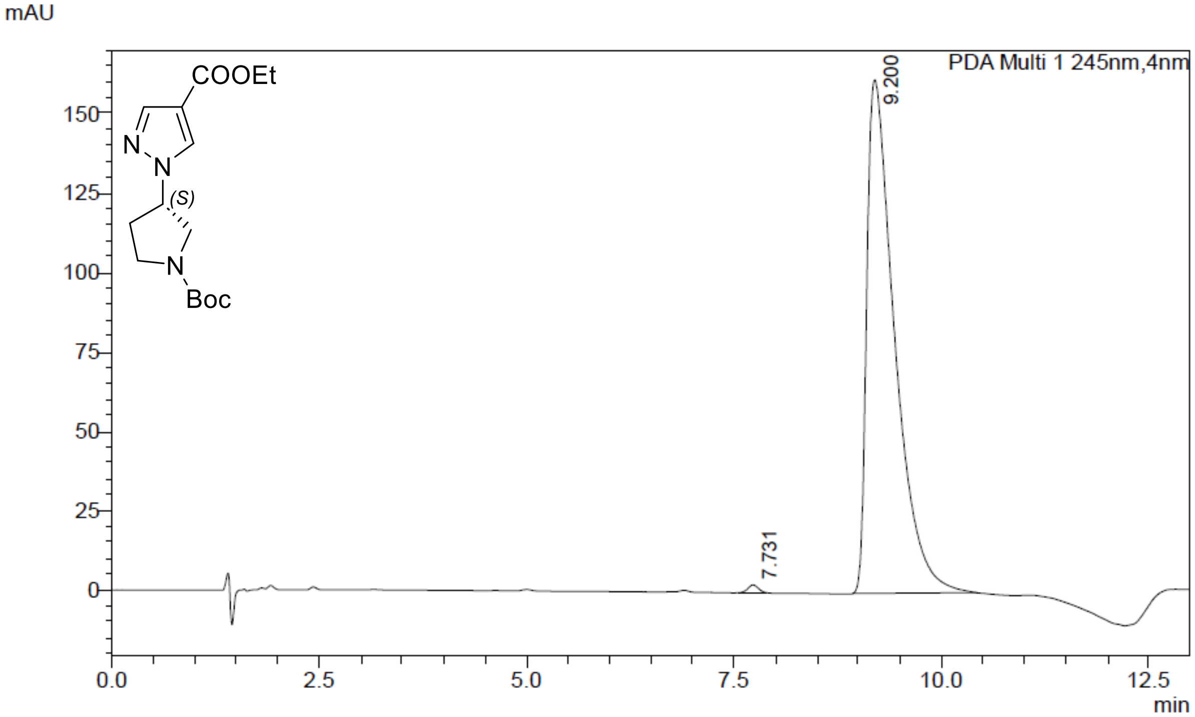

PDA Ch1 245nm

Peak#	Ret. Time	Height	Area	Area%
1	7.731	2429	20634	0.544
2	9.200	161515	3771931	99.456

Figure S211. Chiral HPLC chromatogram view of **(*S*)-14a**.

Sample Name : MDP-215-2
Injection Volume : 5 uL
Column : CHIRAL ART Amylose SA
Flow : 1.0 mL/min
Eluent : water/acetonitrile

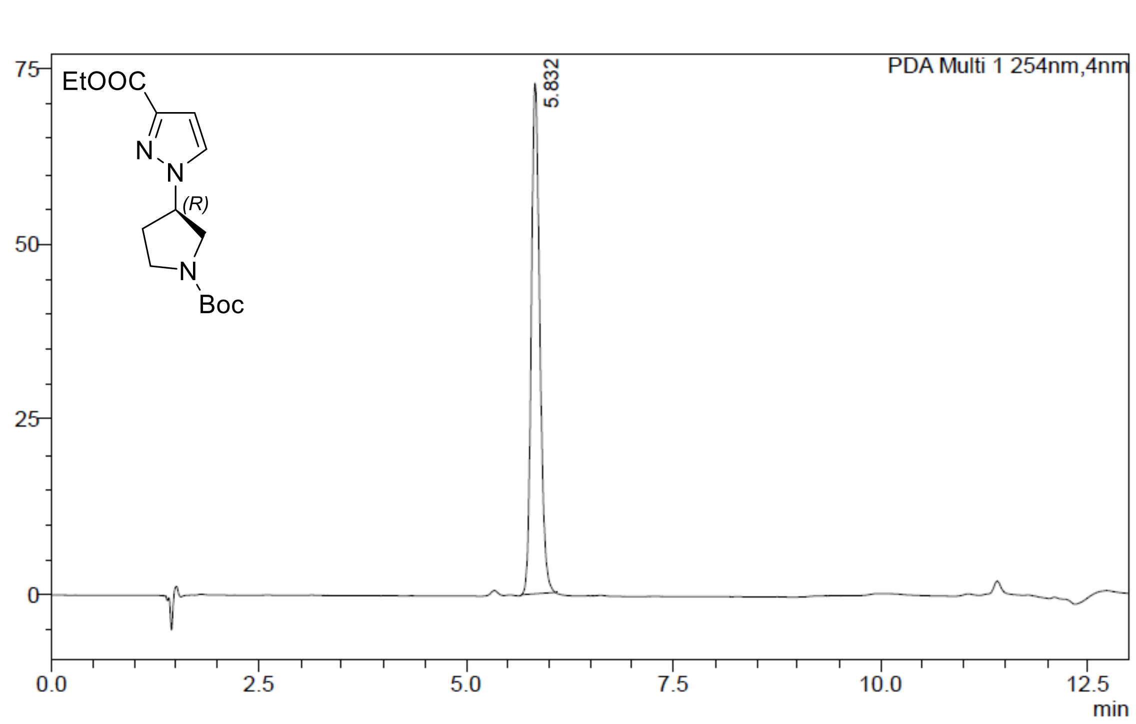

PDA Ch1 254nm

Peak#	Ret. Time	Height	Area	Area%
1	5.832	72620	521102	100.000

Figure S212. Chiral HPLC chromatogram view of ***(R)*-14b**.

Sample Name : MDP-216-2
Injection Volume : 5 uL
Column : CHIRAL ART Amylose SA
Flow : 1.0 mL/min
Eluent : water/acetonitrile

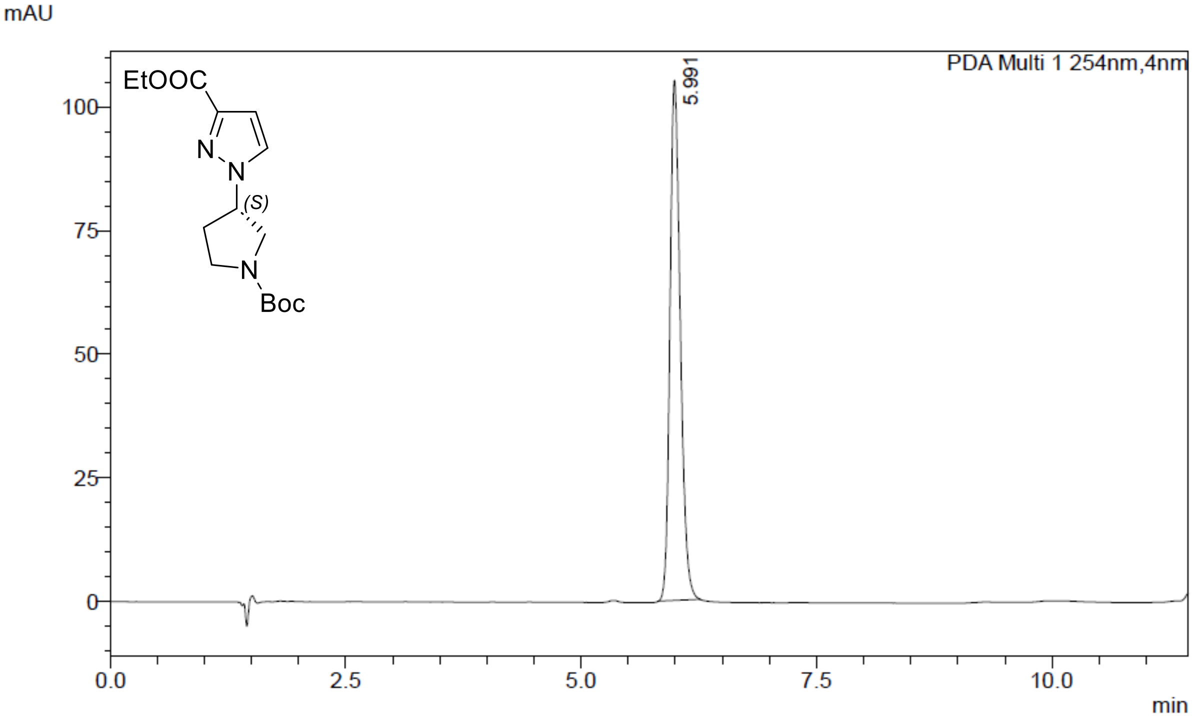

PDA Ch1 254nm

Peak#	Ret. Time	Height	Area	Area%
1	5.991	105105	806378	100.000

Figure S213. Chiral HPLC chromatogram view of ***(S)*-14b**.

Sample Name : MDP-215-1
Injection Volume : 3 uL
Column : CHIRAL ART Amylose SA
Flow : 1.0 mL/min
Eluent : water/acetonitrile

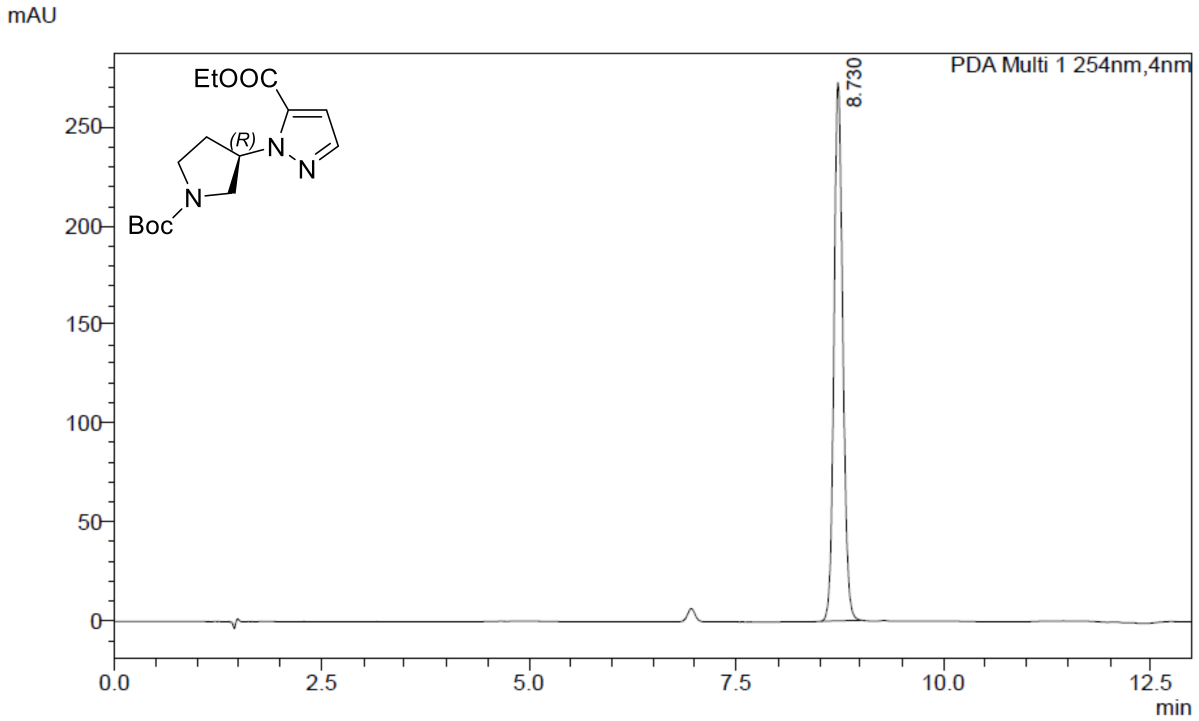

PDA Ch1 254nm

Peak#	Ret. Time	Height	Area	Area%
1	8.730	270944	2017551	100.000

Figure S214. Chiral HPLC chromatogram view of **(*R*)-15b**.

Sample Name : MDP-216-1
Injection Volume : 3 uL
Column : CHIRAL ART Amylose SA
Flow : 1.0 mL/min
Eluent : water/acetonitrile

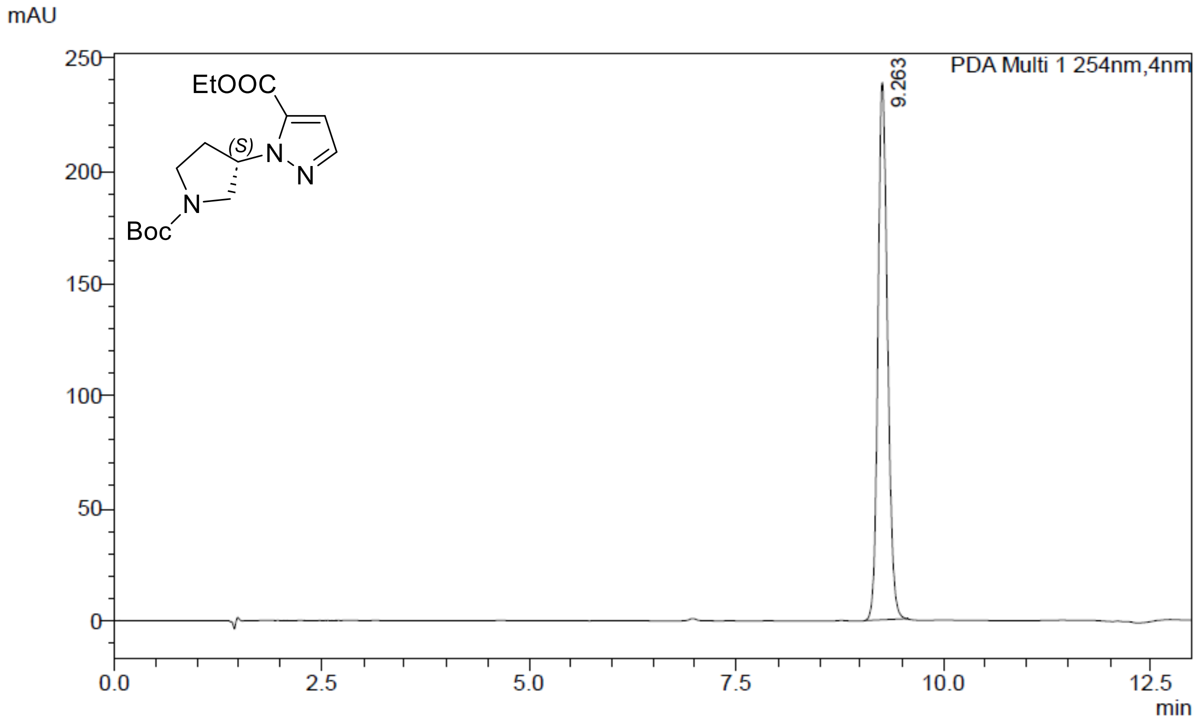

PDA Ch1 254nm

Peak#	Ret. Time	Height	Area	Area%
1	9.263	238723	1928546	100.000

Figure S215. Chiral HPLC chromatogram view of **(*S*)-15b**.

Sample Name : RO-D-34-2
Injection Volume : 10 uL
Column : CHIRAL ART Cellulose SJ
Flow : 1.0 mL/min
Eluent : water/acetonitrile

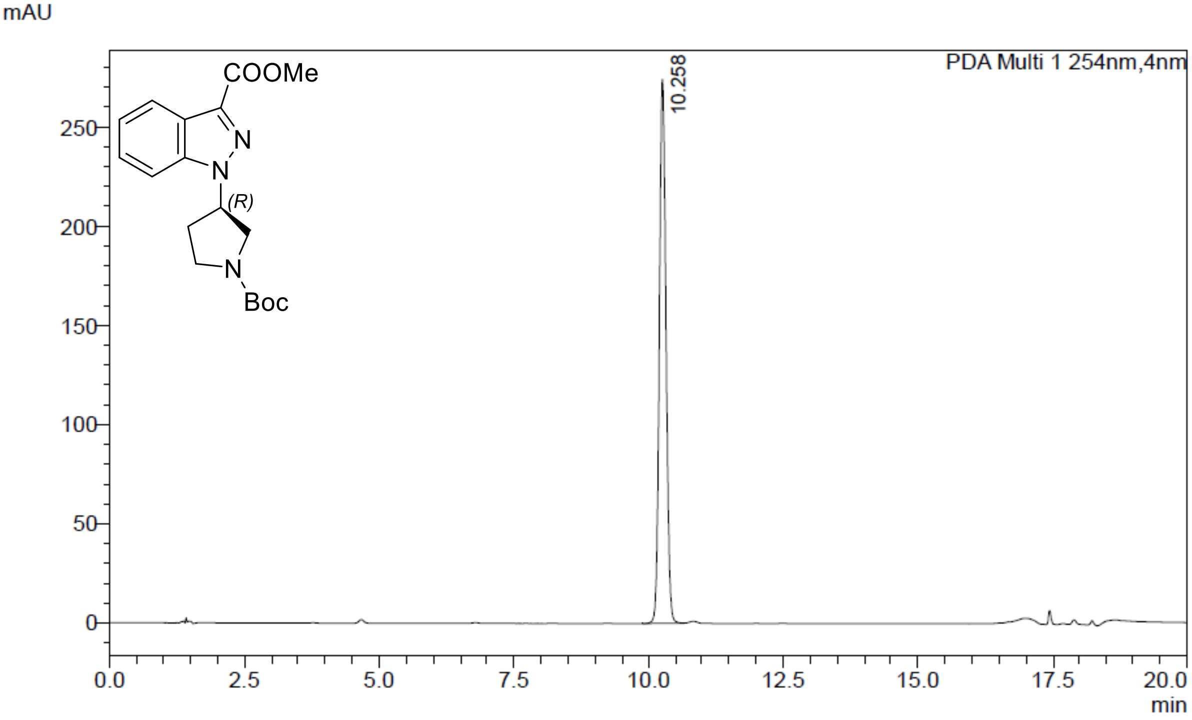

PDA Ch1 254nm

Peak#	Ret. Time	Height	Area	Area%
1	10.258	274135	2358123	100.000

Figure S216. Chiral HPLC chromatogram view of ***(R)*-19a**.

Sample Name : RO-D-29-2
Injection Volume : 10 uL
Column : CHIRAL ART Cellulose SJ
Flow : 1.0 mL/min
Eluent : water/acetonitrile

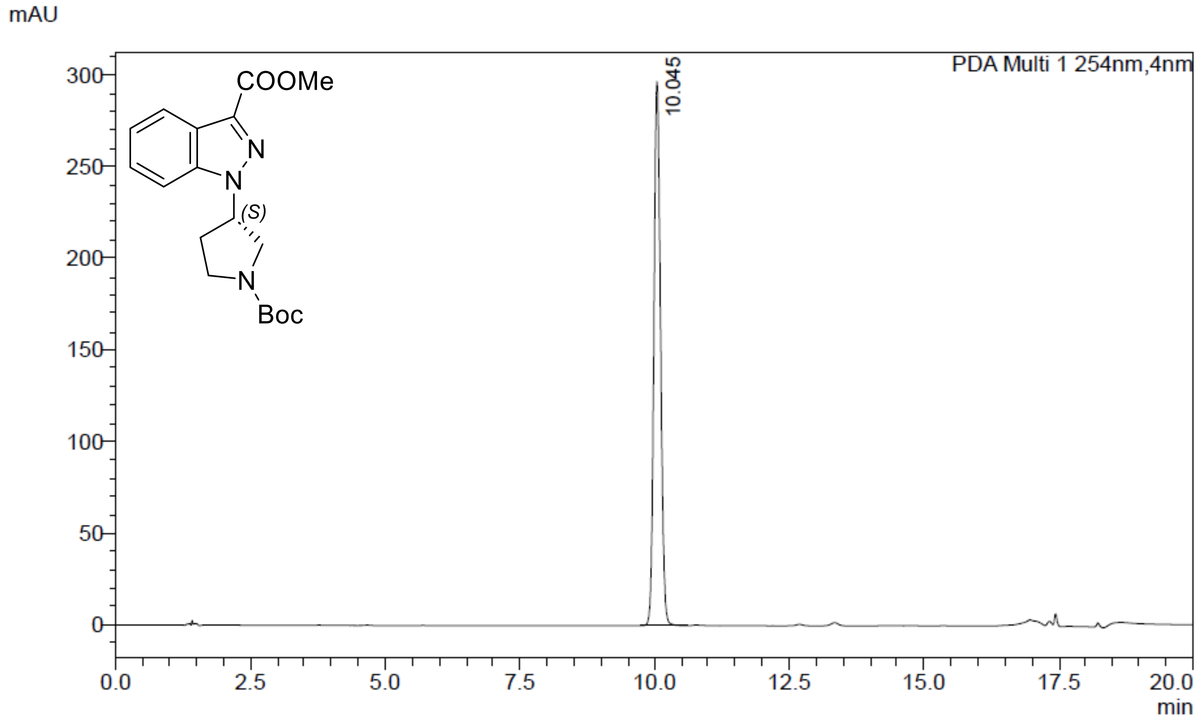

PDA Ch1 254nm

Peak#	Ret. Time	Height	Area	Area%
1	10.045	296418	2537667	100.000

Figure S217. Chiral HPLC chromatogram view of **(*S*)-19a**.

Sample Name : RO-D-34-1
Injection Volume : 10 uL
Column : CHIRAL ART Cellulose SB
Flow : 1.0 mL/min
Eluent : water/acetonitrile

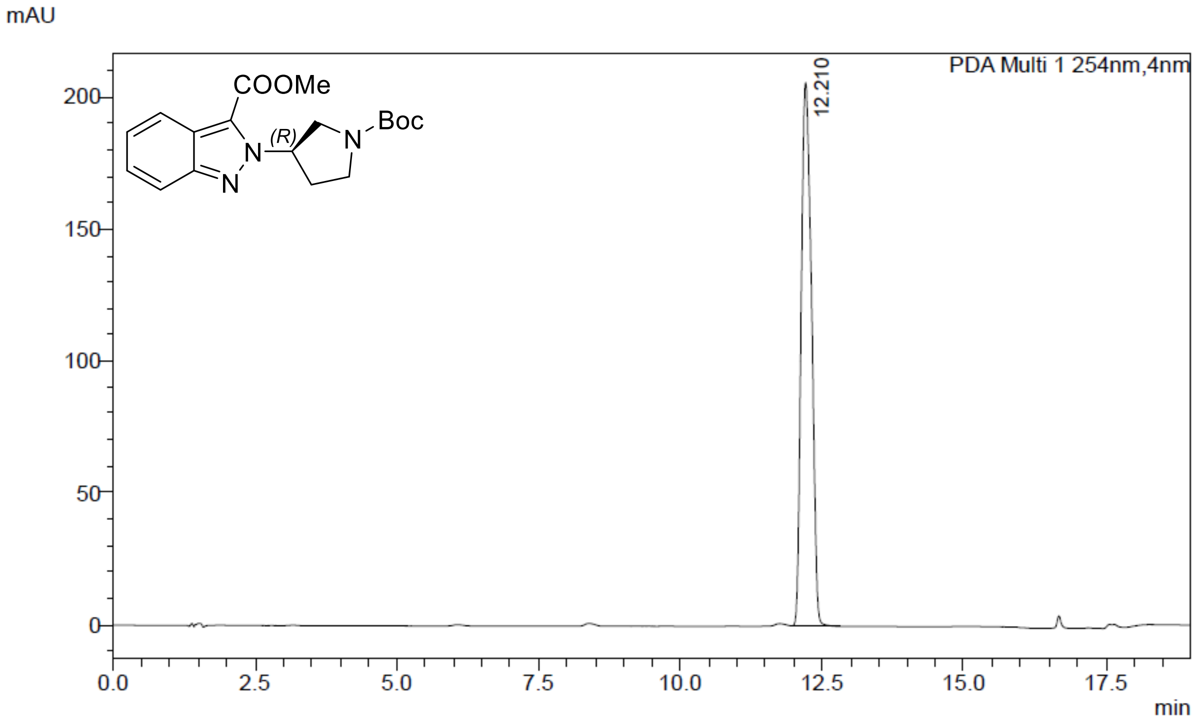

PDA Ch1 254nm

Peak#	Ret. Time	Height	Area	Area%
1	12.210	205431	2597500	100.000

Figure S218. Chiral HPLC chromatogram view of ***(R)*-20a**.

Sample Name : RO-D-29-1
Injection Volume : 10 uL
Column : CHIRAL ART Cellulose SB
Flow : 1.0 mL/min
Eluent : water/acetonitrile

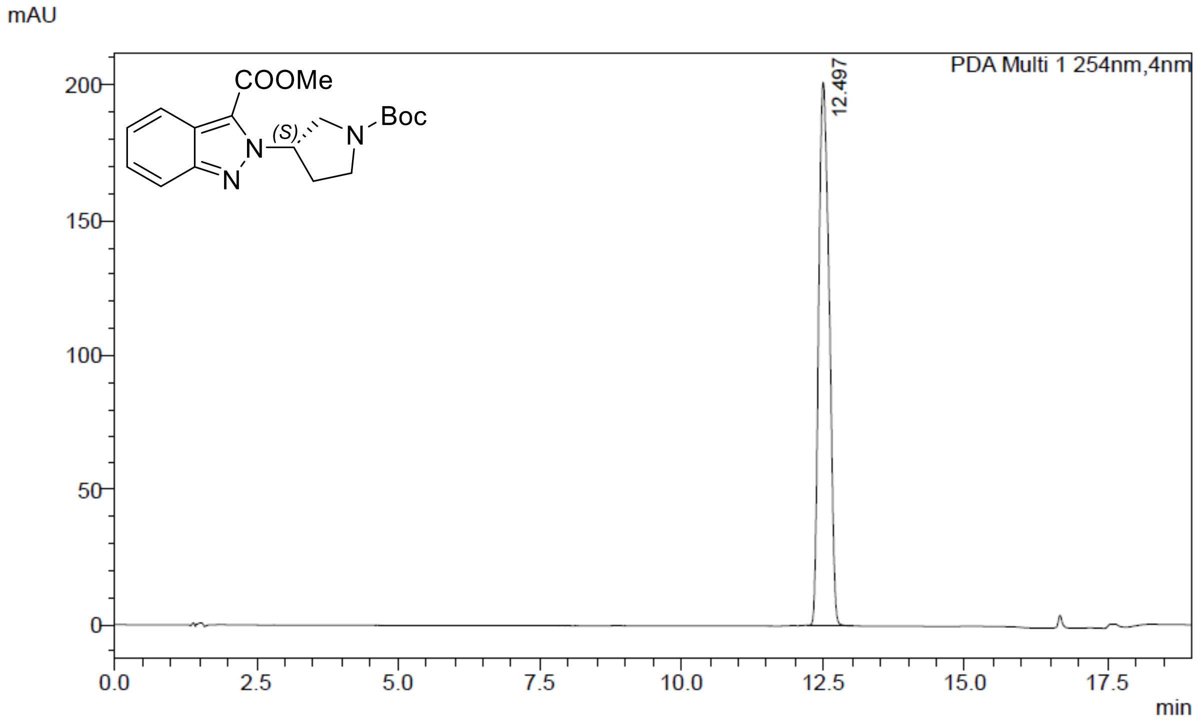

PDA Ch1 254nm

Peak#	Ret. Time	Height	Area	Area%
1	12.497	201488	2591706	100.000

Figure S219. Chiral HPLC chromatogram view of ***(S)*-20a**.

Sample Name : RO-D-33-1
Injection Volume : 10 uL
Column : CHIRAL ART Cellulose SJ
Flow : 1.0 mL/min
Eluent : water/acetonitrile

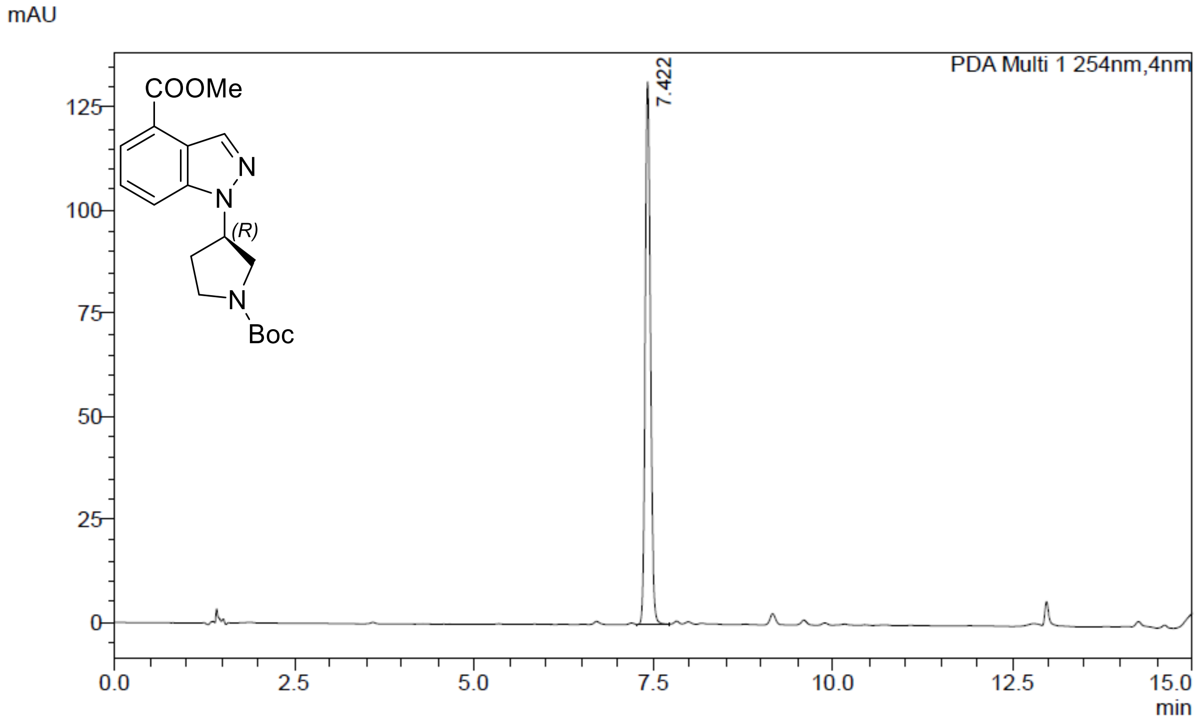

PDA Ch1 254nm

Peak#	Ret. Time	Height	Area	Area%
1	7.422	131762	667997	100.000

Figure S220. Chiral HPLC chromatogram view of **(*R*)-19b**.

Sample Name : RO-D-26-1
Injection Volume : 10 uL
Column : CHIRAL ART Cellulose SJ
Flow : 1.0 mL/min
Eluent : water/acetonitrile

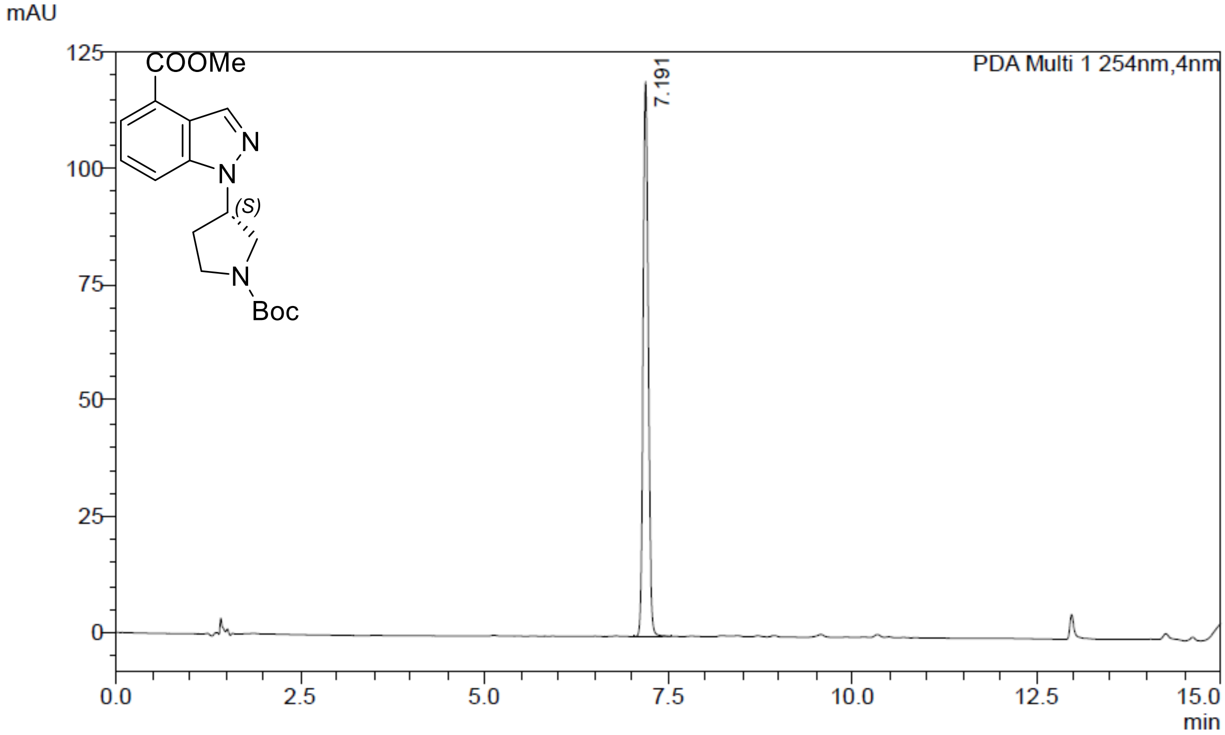

PDA Ch1 254nm

Peak#	Ret. Time	Height	Area	Area%
1	7.191	119103	581702	100.000

Figure S221. Chiral HPLC chromatogram view of ***(S)*-19b**.

Sample Name : RO-D-33-2
Injection Volume : 10 uL
Column : CHIRAL ART Cellulose SJ
Flow : 1.0 mL/min
Eluent : water/acetonitrile

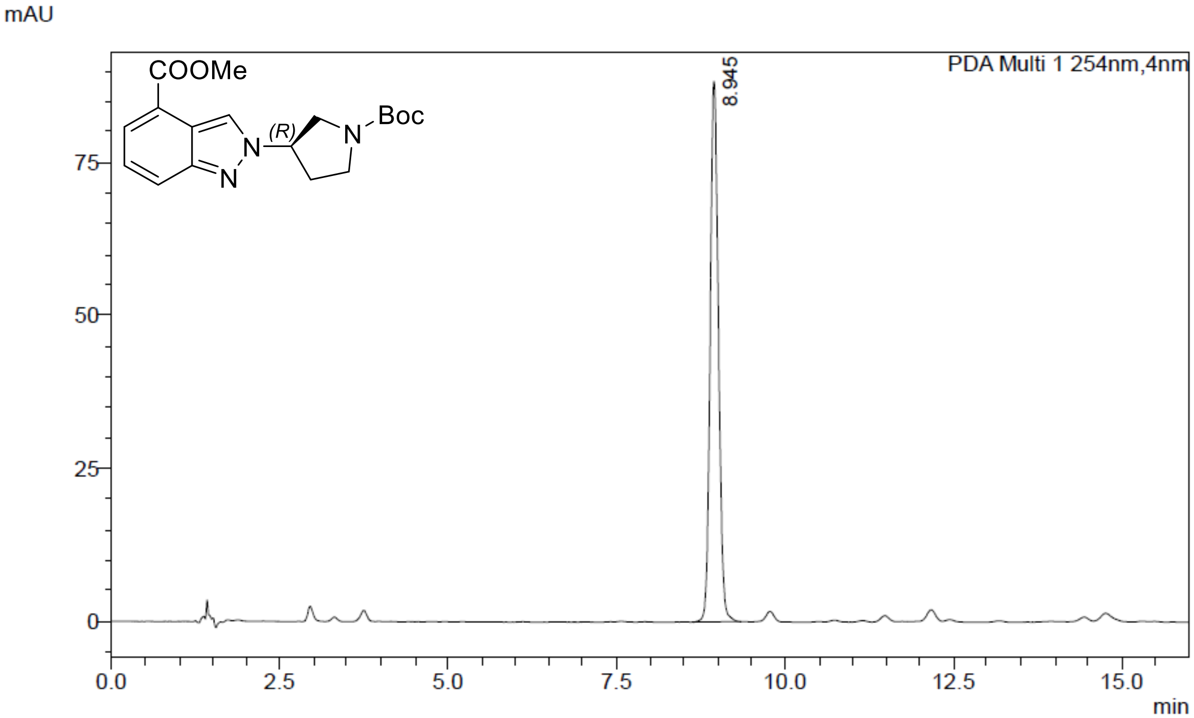

PDA Ch1 254nm

Peak#	Ret. Time	Height	Area	Area%
1	8.945	88111	754505	100.000

Figure S222. Chiral HPLC chromatogram view of ***(R)*-20b**.

Sample Name : RO-D-26-2
Injection Volume : 10 uL
Column : CHIRAL ART Cellulose SJ
Flow : 1.0 mL/min
Eluent : water/acetonitrile

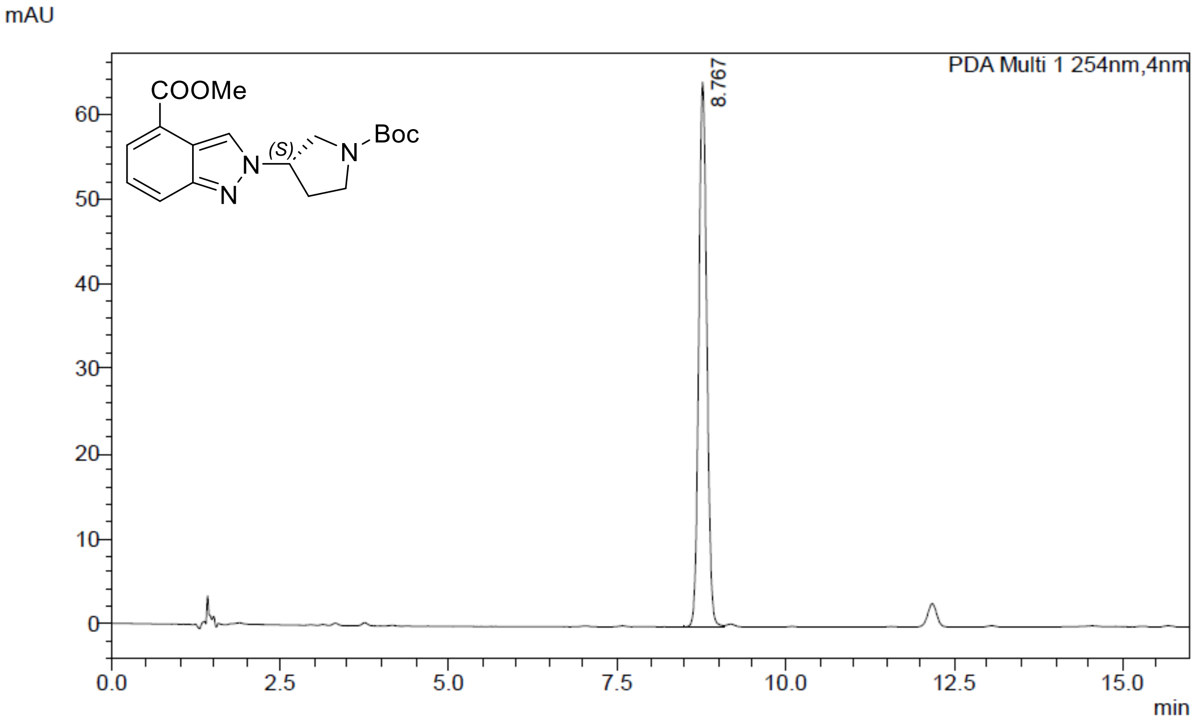

PDA Ch1 254nm

Peak#	Ret. Time	Height	Area	Area%
1	8.767	64115	534105	100.000

Figure S223. Chiral HPLC chromatogram view of ***(S)*-20b**.

Sample Name : RO-D-32-1
Injection Volume : 10 uL
Column : CHIRAL ART Cellulose SB
Flow : 1.0 mL/min
Eluent : water/acetonitrile

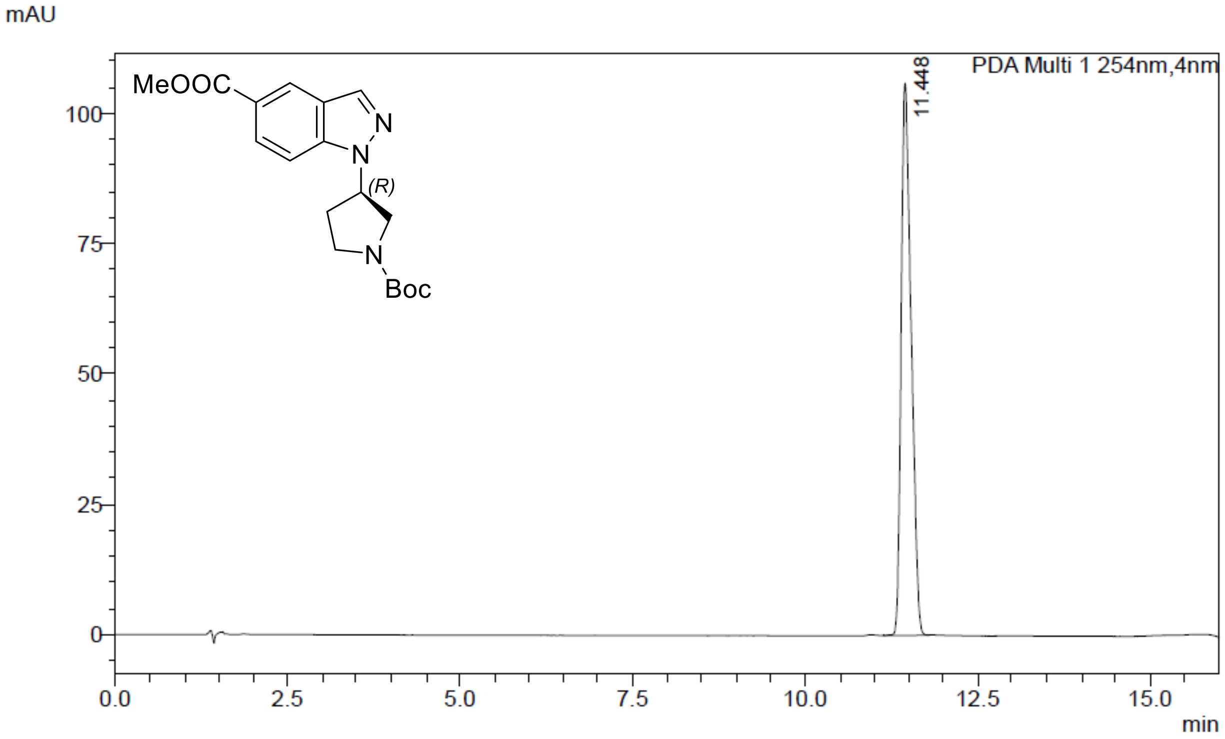

PDA Ch1 254nm

Peak#	Ret. Time	Height	Area	Area%
1	11.448	105876	1069143	100.000

Figure S224. Chiral HPLC chromatogram view of ***(R)*-19c**.

Sample Name : RO-D-25-1
Injection Volume : 10 uL
Column : CHIRAL ART Cellulose SB
Flow : 1.0 mL/min
Eluent : water/acetonitrile

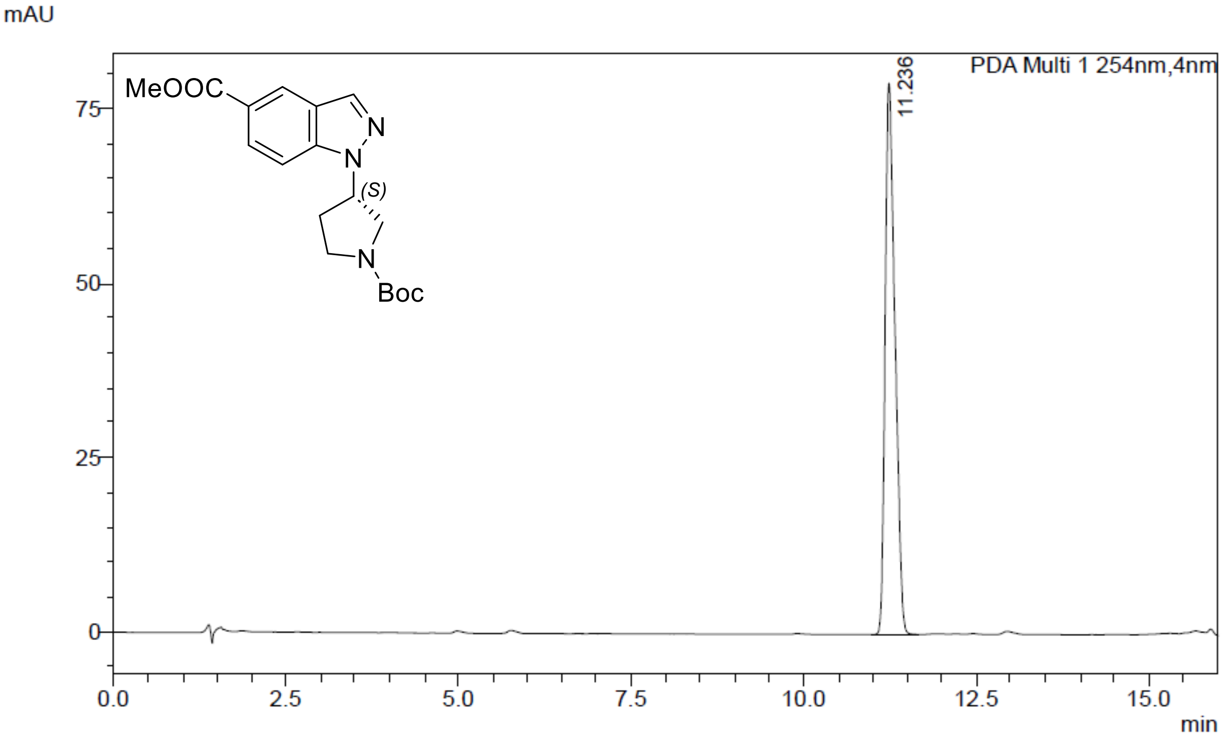

PDA Ch1 254nm

Peak#	Ret. Time	Height	Area	Area%
1	11.236	78876	782844	100.000

Figure S225. Chiral HPLC chromatogram view of ***(S)*-19c**.

Sample Name : RO-D-32-2
Injection Volume : 10 uL
Column : CHIRAL ART Cellulose SJ
Flow : 1.0 mL/min
Eluent : water/acetonitrile

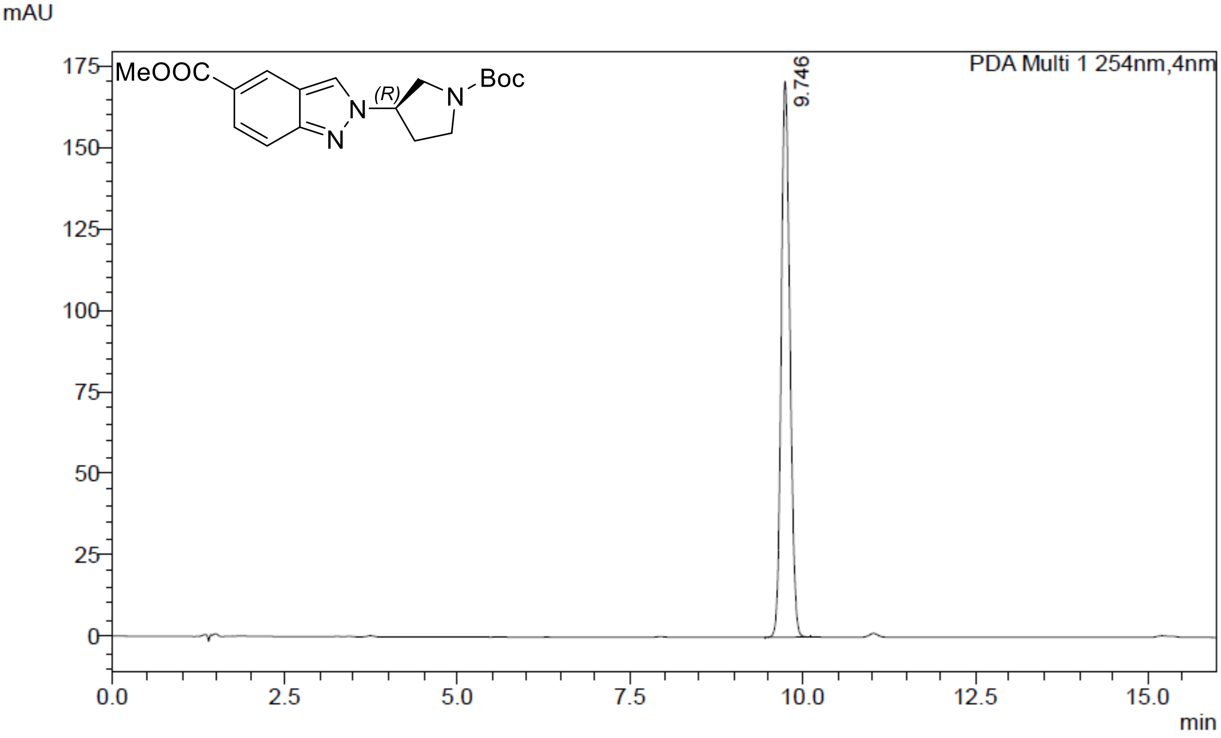

PDA Ch1 254nm

Peak#	Ret. Time	Height	Area	Area%
1	9.746	170036	1490188	100.000

Figure S226. Chiral HPLC chromatogram view of ***(R)*-20c**.

Sample Name : RO-D-25-2
Injection Volume : 10 uL
Column : CHIRAL ART Cellulose SJ
Flow : 1.0 mL/min
Eluent : water/acetonitrile

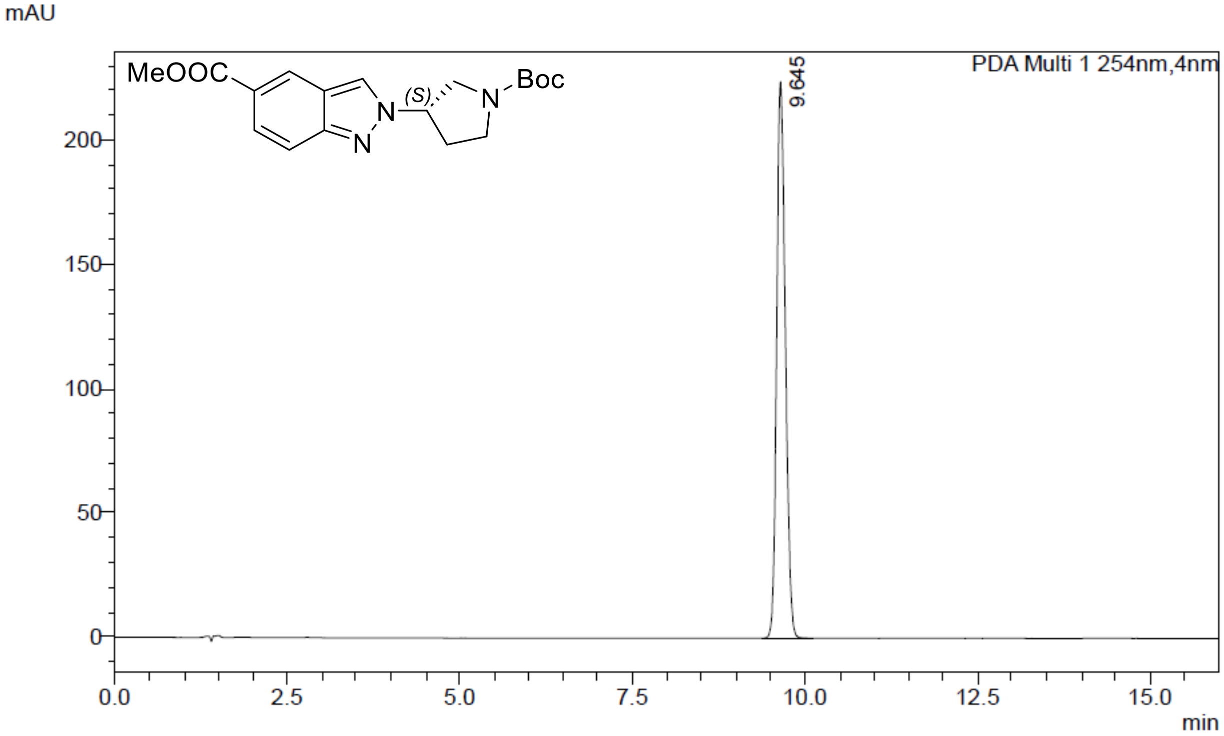

PDA Ch1 254nm

Peak#	Ret. Time	Height	Area	Area%
1	9.645	223624	1962909	100.000

Figure S227. Chiral HPLC chromatogram view of **(*S*)-20c**.
